# Genomic Insights into the Origin of a Thermotolerant Tomato Line and Identification of Candidate Genes for Heat Stress

**DOI:** 10.3390/genes14030535

**Published:** 2023-02-21

**Authors:** Salvatore Graci, Valentino Ruggieri, Silvana Francesca, Maria Manuela Rigano, Amalia Barone

**Affiliations:** 1 Department of Agricultural Sciences, University of Naples Federico II, Portici, 80055 Naples, Italy; 2 Biomeets Consulting, Carrer d’Àlaba, 61, 08005 Barcelona, Spain

**Keywords:** high temperatures, whole-genome resequencing, SNPs, InDels, wild species introgressions, *Solanum pimpinellifolium*, heat shock proteins (HSPs), heat shock factors (HSFs), flower number

## Abstract

Climate change represents the main problem for agricultural crops, and the constitution of heat-tolerant genotypes is an important breeder’s strategy to reduce yield losses. The aim of the present study was to investigate the whole genome of a heat-tolerant tomato genotype (E42), in order to identify candidate genes involved in its response to high temperature. E42 presented a high variability for chromosomes 1, 4, 7 and 12, and phylogenetic analysis highlighted its relationship with the wild *S. pimpinellifolium* species. Variants with high (18) and moderate (139) impact on protein function were retrieved from two lists of genes related to heat tolerance and reproduction. This analysis permitted us to prioritize a subset of 35 candidate gene mapping in polymorphic regions, some colocalizing in QTLs controlling flowering in tomato. Among these genes, we identified 23 HSPs, one HSF, six involved in flowering and five in pollen activity. Interestingly, one gene coded for a flowering locus T1 and mapping on chromosome 11 resides in a QTL region controlling flowering and also showed 100% identity with an *S. pimpinellifolium* allele. This study provides useful information on both the E42 genetic background and heat stress response, and further studies will be conducted to validate these genes.

## 1. Introduction

As sessile organisms, plants are continuously exposed to environmental stresses through their entire life cycle [1]. Climate change and global warming represent the main threats for many agricultural crops [2]. It was reported that by the end of the 21st century, global temperatures are estimated to increase on average 1–3.7 °C [3]. Temperatures higher than 10–15 °C above the optimum for plant growth and development cause heat stress [4]. Tomato is one of the most extensively grown and consumed horticultural crops and can survive in a wide range of climatic conditions. However, high temperatures negatively affect both vegetative growth and reproductive processes (of modern cultivars), resulting in losses of yield and fruit quality traits [5]. Temperature changes alter plant morphology, anatomy and physiology, comporting with protein denaturation and leading to an increased fluidity of membrane lipids, inactivation of enzymes in chloroplasts and mitochondria, disruption of membrane integrity, production and accumulation of reactive oxygen species (ROS) and inhibition of photosystem II (PSII) damage repair [6,7]. Even plant reproductive development is highly sensitive to heat stress, which determines flower and male gametophyte abortion with consequent reduction of the fruit set [8]. It was reported that an increase of few degrees above the optimal daily temperature of 25 °C led to a poor pollen germination and impaired pollen tube development [9,10]. In this context, the constitution of heat-tolerant tomato genotypes represents an important breeder’s challenge to face abiotic stresses and reduce yield losses.

In recent works, different authors have focused their attention on the selection of heat-tolerant plants through agronomical, physiological, qualitative and molecular traits. In particular, Olivieri et al. [11] phenotyped 10 tomato landraces grown under high temperatures for yield-related traits by comparing data recorded during two years in two different environments. Results showed that during the summer season, when temperatures were recorded to exceeded 32 °C with peaks of 38 °C, one genotype, named E42, exhibited a high and stable yield production (2.93 kg/plant) comparable to those of the tomato hybrids DOCET (3.13 kg/plant) and JAG8810 (3.37 kg/plant), reported to be heat-tolerant (Monsanto, unpublished results). Particularly, E42 stands out for the high number of total flowers and total number of fruits in all environments (open field, under tunnel), even when grown with one-month late transplant to increase exposure to high temperatures during reproduction [11,12,13]. These data were also confirmed by using a selection index based on fruit set, total number of fruits and yield production (both by Olivieri et al. [13] and Ruggieri et al. [12]), where the good performances of E42 were compared with 14 and 45 tomato lines, respectively. Francesca et al. [14] compared physiological traits of E42 and the thermotolerant LA3120 (Malintka) genotype (Tomato Genetics Resource Center, TGRC, University of California, Davis, CA, USA) under heat stress. In E42, under high temperatures, a very strong increase (+173%) was observed in stomatal density and transpiration rate, compensated by a decrease in stomatal length and width. Moreover, an increase in stomatal conductance was observed, while the intracellular CO_2_ concentration remained stable. Net photosynthetic rate and Fv/Fm values were not affected by heat stress. All these physiological evaluations allowed the authors to further demonstrate the tolerance of the referred genotype, which was also evaluated under drought and combined stresses [11]. Since high temperatures in tomato fields mainly occur during the reproductive stages, in our laboratory we focused our attention on traits related to high numbers of flowers and flowers/inflorescence. Interestingly, one peculiar trait observed in E42 during reproduction was the high number of flowers and fruits produced, leading to high yield even though the fruit set was usually around 50%. As such, it is likely that the high flowering trait observed in E42 could lead to high plant production, observed also under adverse conditions. Following many years of evaluation (from 2016 to 2022), this trait was always observed both when growing the genotype in standard conditions and at high temperatures, thus leading to the belief that this is a constitutive trait of the genotype E42.

Data obtained using a genotyping by sequencing (GBS) strategy [11,13] evidenced a high genetic variability of E42 respect to other analyzed genotypes. Since this strategy is based on reduced representation sequencing (RRS) approaches through the use of restriction enzymes [15,16], its major limitation is due to the random distribution of restriction enzyme sites on the genome, and thus the inability to target polymorphisms localized within genes or having a functional significance [17]. The aim of the present study was to investigate the whole genome sequence of the E42 genotype using whole genome resequencing data to explore the origin of its genetic variability and to identify candidate genes involved in response to high temperatures. A high number of polymorphisms was detected across the genome when compared to the tomato reference genome of cv. Heinz (Tomato Genome version SL4.0, available at the Solgenomics Network, www.solgenomics.net [18]), with a major concentration on four chromosomes. Phylogenetic analysis evidenced the strong relationship with *S. pimpinellifolium* wild species, from which probably the E42 genotype descends. Since the previously reported phenotypic data evidenced that vegetative growth was not affected by high temperatures, we focused our attention on reproductive stages and consequently on yield-related traits. Among these, the major distinctive trait of this genotype is its high florigen activity. Thus, candidate genes related to flower and pollen development were investigated.

## 2. Materials and Methods

### 2.1. Resequencing of the E42 Tomato Line

Genomic DNA extraction was performed with the Qiagen DNeasy plant mini kit following the standard protocol. Afterwards, DNA was randomly fragmented by sonication, and the fragments were end-polished, A-tailed, and ligated with the full-length adapters of Illumina sequencing, followed by further PCR amplification with P5 and indexed P7 oligos. The PCR products as the final construction of the libraries were purified with the AMPure XP system. Then, libraries were checked for size distribution with an Agilent 2100 Bioanalyzer (Agilent Technologies, Santa Clara, CA, USA), and quantified by real-time PCR (to meet the criteria of 3 nM). Qualifying libraries were fed into Illumina sequencers (Novaseq6000) after pooling according to effective concentration and expected data volume.

### 2.2. Variant Calling and Annotation

Raw FASTQ files were quality-filtered and trimmed using Trimmomatic [19] v.0.39 (http://www.usadellab.org/cms/?page=trimmomatic) with default parameters. Paired trimmed reads were aligned with the *Solanum lycopersicum* reference genome (Tomato Genome version SL4.0, available at the Solgenomics Network, www.solgenomics.net) using Bowtie-2 [20] with default parameters. The resulting SAM and BAM files were sorted, de-duplicated and indexed with Samtools [21]. Finally, the variant calling step was performed by BCFtools mpileup [22] with default parameters. Filtering procedure of variants was performed using VCFtools [23], setting parameters as follows: minQ = 15 and minimum mean of depth of coverage (min–mean DP) = 15. Single-nucleotide polymorphism (SNP) density and distribution across chromosomes were estimated using the snpden function of VCFtools (1 Mb non-overlapping windows). SNP and insertion and/or deletion (InDel) density plots were generated using CMplot of the rMVP package in R [24]. Variants were annotated using SnpEff software [25] using the Tomato SL4.0 genome assembly and ITAG4.1 annotation (available at the Solgenomics Network). A VCF file was generated containing the prediction of the possible effects of SNPs and InDels. These were categorized by putative impact (high, moderate, low and modifier) and effect (i.e., disruptive in-frame insertion, disruptive in-frame deletion, downstream gene variant, frameshift variant, intergenic region, missense variant, stop gained).

### 2.3. Phylogenetic Analysis

In order to investigate the origin of the genetic variability detected in E42, phylogenetic analysis was performed using the neighbor-joining (NJ) method implemented in VCF-kit [26] (http://vcf-kit.readthedocs.io/). A dataset of variants of 82 samples belonging to 13 distinct tomato species was retrieved from a previous study [27] under project number PRJEB5235. Since this study was conducted on a different *Solanum lycopersicum* assembly release (SL2.40), we performed an additional variant calling for E42 on the same tomato genome version SL2.40. This allowed us to have a coherent variant dataset. All the 83 VCF files were indexed and merged through BCFtools using default parameters. Filtering of variants was performed using VCFtools, setting parameters as follows: minQ = 15 and max missing = 0.5. After that, the merged VCF file was filtered for chromosome, thus obtaining 12 VCF files. The phylogenetic analysis was performed for each chromosome separately, and 12 Newick files were generated after running VCF-kit tool with default parameters. Finally, these files were uploaded on iTol [28] to plot the phylogenetic trees.

### 2.4. Identification of Variants in Candidate Genes

In order to identify candidate genes potentially involved in heat stress response, a keyword search was undertaken using the following terms: “heat”, “HSP”, “HSF”, “flower”, “pollen”, “anthesis”, “anther”, and “fruit set”. The Solgenomics database (ITAG4.1 version of tomato genome annotation) was investigated with this aim. Furthermore, a selected number of E42 gene variants were deeply investigated by aligning them with *Solanum lycopersicum* cv. Heinz and *Solanum pimpinellifolium* LA2093 accession [29] (Tomato Genome version SL4.0 and 1.5 respectively, available at the Solgenomics Network) using Clustal Omega [30], in order to identify variants and sequence introgressions from the wild species. In silico promoter analysis using a region of 3000 bp from the gene start site was performed using PlantCare [31]. This allowed us to identify putative cis-acting elements related to the heat stress response and to underline differences among E42 and the tomato reference genome (cv. Heinz) in these regulatory regions.

## 3. Results

### 3.1. Variant Calling

**Figure 1:**
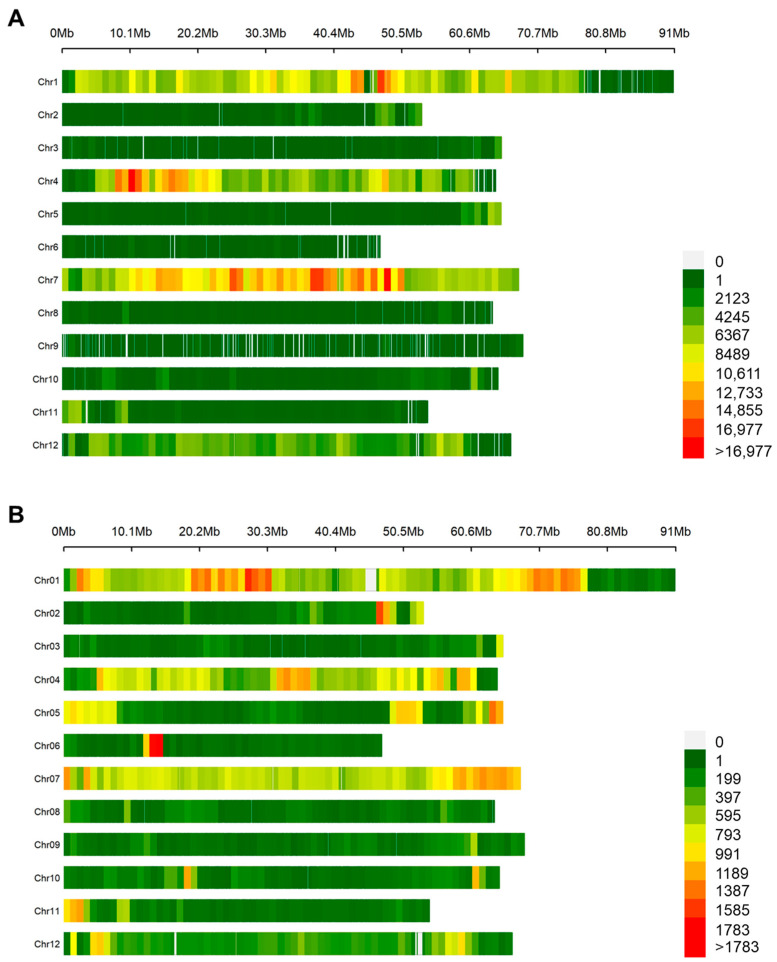
Density distribution of filtered SNPs (**A**) and InDels (**B**) within 1 Mb windows across the 12 tomato chromosomes.

Resequencing of E42 tomato genotype produced about 25 gigabases (Gb) of raw sequence data, (166,461,626 raw reads), representing ~25× coverage. The variant calling analysis evidenced 2,126,253 raw SNPs and InDels. After filtering, 1,992,156 high-quality homozygous variants were maintained: 1,755,606 SNPs and 236,550 InDels. Interestingly, 92% of SNPs and 67% of InDels were mapped on chromosomes 1, 4, 7 and 12 (Supplementary Table S1) as showed in Figure 1A consistent number of InDels (8%) were also mapped on chromosome 5.

As a whole, 18 highly polymorphic chromosome regions were identified when considering both SNP (more than 12,733) and InDel (more than 1189) density variants detected on the whole E42 genome. On chromosome 1, accordingly, with SNP density analysis, two highly polymorphic regions were localized from position 40,000,000 to 44,000,000 (p1_1) and from 46,000,000 to 50,000,000 (p1_2), while from the InDel density plot three regions could be defined from position 2,000,000 to 6,000,000 (p1_3), from 19,000,000 to 30,000,000 (p1_4) and from 67,000,000 to 75,000,000 (p1_5). On chromosome 2, a small region ranging from 46,000,000 to 48,000,000 (p2_1) showed a high number of InDels. On chromosome 4, three highly polymorphic regions could be defined: from 8,000,000 to 21,000,000 (p4_1) showing SNP variants higher than the fold, from 31,000,000 to 36,000,000 (p4_2) and from 57,000,000 to 60,000,000 (p4_3) showing a high number of InDel variants. On chromosome 5, a high number of polymorphisms was localized from position 62,000,000 to the end (p5_1), while on chromosome 6 a high number of InDels mapped from 12,000,000 to 14,000,000 (p6_1). Chromosome 7 presented three large regions with many SNP variants: the first ranging from 14,000,000 to 18,000,000 (p7_1), the second from 22,000,000 to 40,000,000 (p7_2) and the third from 41,000,000 to 50,000,000 (p7_3), while from position 57,000,000 to the end (p7_4) it showed a high number of InDels. Finally, a high number of InDels mapped on chromosome 11 from the start position to 3,000,000 (p11_1), on chromosome 12 from 4,000,000 to 6,000,000 (p12_1) and from 56,000,000 to 58,000,000 (p12_2).

### 3.2. Phylogenetic Analysis

**Figure 2:**
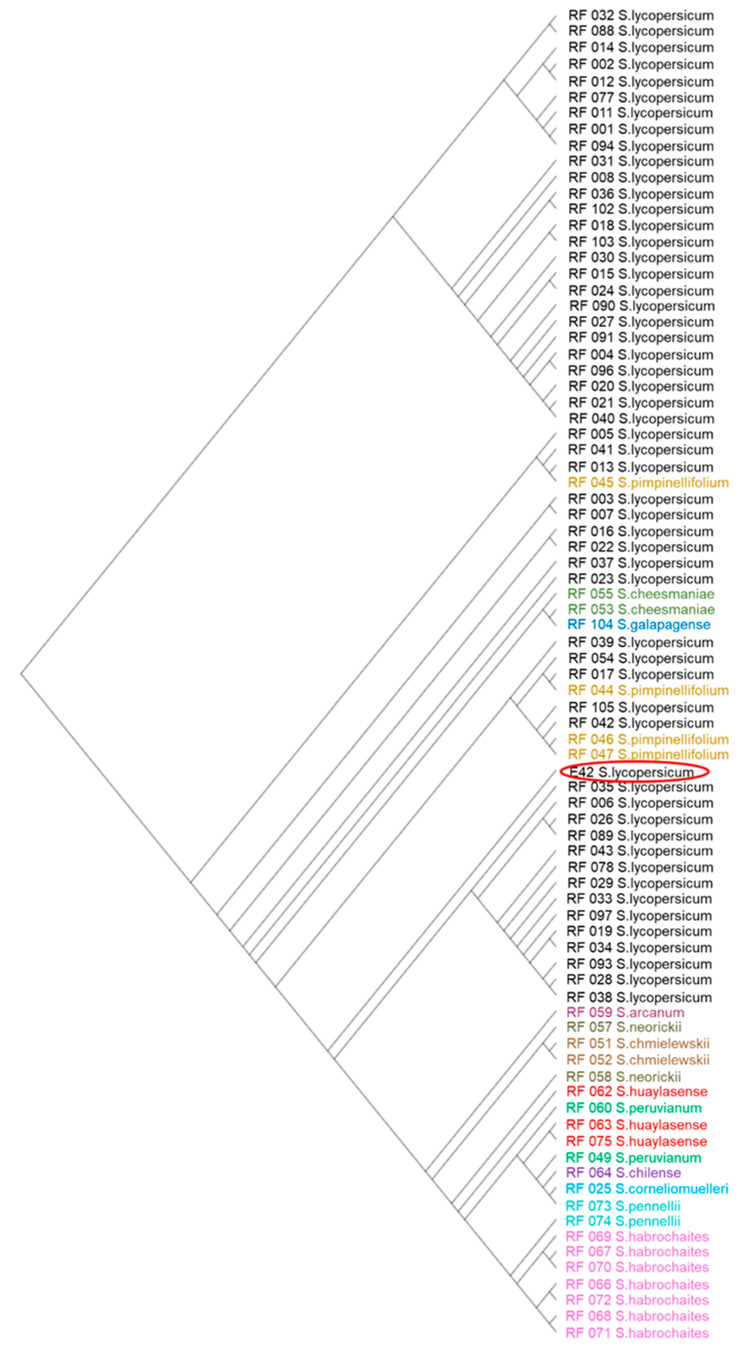
Phylogenetic tree of the tomato chromosome 1 involving the E42 genotype and 82 accessions belonging to 13 tomato species.

To better define the origin of the genetic variability evidenced in E42, a phylogenetic analysis was performed with a comprehensive dataset of 82 accessions belonging to 13 tomato species. The dataset comprised 54 accessions of *S. lycopersicum*, seven of *S. habrochaites*, four of *S. pimpinellifolium*, three of *S. huaylasense*, two of *S. pennellii*, *S. peruvianum*, *S. chmielewskii*, *S. cheesmaniae* and *S. neorickii* respectively, one of *S. corneliomuelleri*, *S. arcanum*, *S. chilense* and *S. galapagense* respectively. The 83 variant calling files were merged and a unique file was generated involving 70,452,665 variants. After filtering, 175,631 raw variants were maintained (Supplementary Table S2). E42 shared the highest number of variants with *S. lycopersicum* accessions and, additionally, with *S. pimpinellifolium*, *S. galapagense* and *S. cheesmaniae* ones. To better understand these relationships, 12 phylogenetic trees were obtained, one for each tomato chromosome (Supplementary Figure S1). A majority of the E42 chromosomes clustered with *S. lycopersicum* accessions. However, on chromosomes 1, 4, 7 and 12, E42 clustered also with *S. pimpinellifolium* accessions, and to a minor extent with *S. galapagense* and *S. cheesmaniae*, as is possible to see for chromosome 1 in Figure 2.

### 3.3. SNPs and InDel Annotation

**Figure 3:**
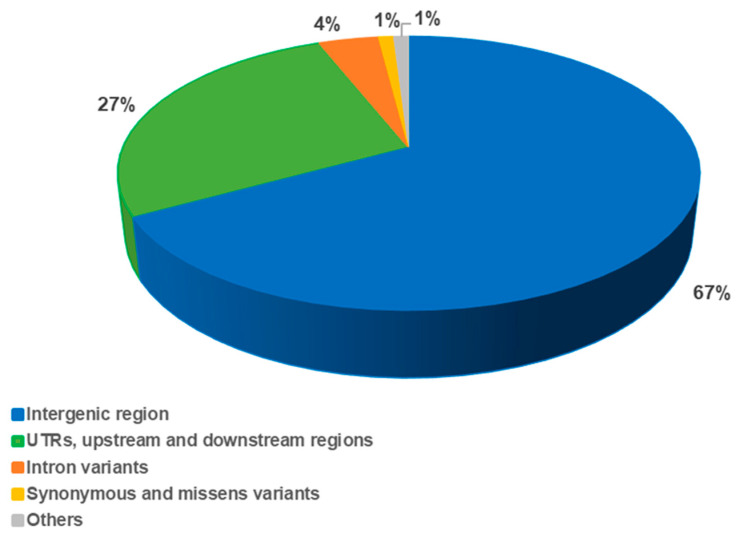
Distribution of 1,992,156 high-confidence homozygous variants based on the predicted type of effect.

SnpEff analysis was performed to estimate the probable impact on proteins of SNPs and InDels detected in E42 compared to Heinz (Supplementary Table S3). As shown in Figure 3, most of the variants were categorized as intergenic regions (67%), followed by UTR variants (including 3’-UTR variants, 5’-UTR premature start codon gain variants, and 5’-UTR variants) and downstream and upstream gene variants (27%), while 6% were mapped in the gene body regions. Among these, 4% were categorized as intron variants and 1% as exonic variants (synonymous and missense variants). Finally, the remaining 1% were categorized as “others”, and together with the missense variants, are responsible of the most interesting effects.

Among the variants evaluated by SnpEff analysis (Supplementary Table S4), most had a putative modifier impact (96.5%), followed by those with moderate (1.8%), low (1.5%) and high (0.3%) impact.

### 3.4. Identification of Variants in Candidate Genes

To investigate the molecular response of E42 genotype to heat stress, the functional descriptions of genes included in the tomato annotation (ITAG4.1) were explored to search for those usually involved in the response to high temperatures, thus obtaining a list of 246 heat-related candidate genes (Supplementary Table S5). Of these, 216 genes were annotated as heat shock proteins (HSPs) and 30 as heat shock factors (HSFs). In particular, four HSFs included variants with a moderate impact. By contrast, a higher variability was observed in the HSPs, where 11 high impact variants affected nine genes (one mapping on chromosomes 2, 8 and 11 respectively, two on chromosome 7 and 4 on chromosome 5), with the following consequent predicted effects: nine InDels produced 8 frameshift variants and one bidirectional gene fusion, while 2 SNPs generated stop gained variants (Supplementary Table S6). As a whole, the moderate impact variants affected 43 genes, producing the following effects: four InDels resulted in one conservative in-frame deletion, one conservative in-frame insertion, one disruptive in-frame deletion and one disruptive in-frame insertion, while all the 92 SNPs generated missense variants (Supplementary Table S6). In addition, since the high number of flowers/inflorescences and fruits were always observed in the heat-tolerant E42 genotype and this could explain the stable production of this genotype under high temperatures, the Solgenomics database was also investigated for detecting reproduction genes related to flowering and pollen development. This second list consisted of 83 genes (Supplementary Table S7), 39 of which were annotated as flower- and 44 as pollen-related. Among these, 21 genes exhibited seven high and 47 moderate impact variants (Supplementary Table S8), with five genes showing only one mutation. The seven high InDels affected six genes (one mapping on chromosomes 1, 7, 11 and 12, and two on chromosome 5), with the consequent predicted functions: six frameshift variants and one frameshift variant and stop lost and splice region variant. The 47 moderate SNPs affected 16 genes, producing the following effect: two missense and splice region variants and 45 missense variants.

Following the analysis of the two lists of genes, we searched for QTLs related to heat tolerance already reported in the literature to detect colocalization between the genes showing variants and these QTLs. In the literature, 172 QTL regions were found [32,33,34,35,36] to be involved in reproduction and production traits related to flowers, pollen and fruits (Supplementary Table S9). These traits were reported to be strongly linked with the response of tomato plants under heat stress. The highest number of QTLs were mapped on chromosomes 2 and 3. Among the QTLs found, 86 were related to reproductive traits, such as anther length, flower number, flowering time, inflorescence number, pollen number and viability, stigma and style exertion (Table 1).

**Figure 4:**
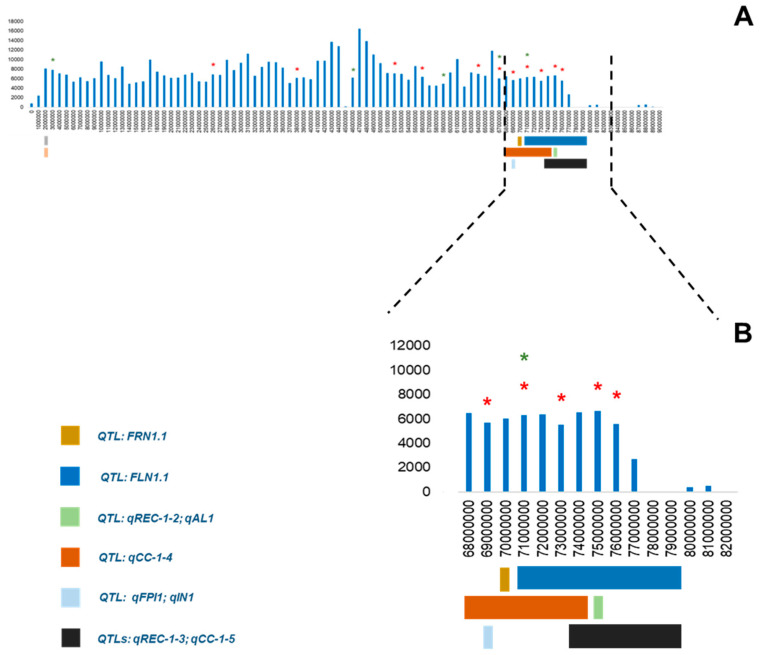
SNP density of 1 Mb representation of E42 chromosome 1 compared with the one of Heinz (Tomato Genome version SL4.0). (**A**) SNP density of the whole chromosome 1; (**B**) detail of a region within 68,000,000 and 82,000,000 bp. Different QTLs are represented by stained boxes (see legend below the figure). The heat shock-(red stars) and reproductive-(green stars) related E42 genes are reported in Supplementary Tables S6 and S8; FLN = numbers of flowers and FRN = numbers of fruits [33]; qAL = anther length, qFPI = flowers per inflorescence and qIN = inflorescence number [36]; qREC = relative electrical conductivity and qCC = chlorophyll content [34].

In most cases, the polymorphic genes in E42, which imply a high or moderate impact on the protein, colocalized with a group of QTLs related to reproductive traits (Supplementary Figure S2). For example, a number of QTLs were mapped on a specific area of chromosome 1 (Figure 4), among which FLN1.1 controlled the number of flowers, qFPI and qIN QTLs controlled the flowers per inflorescence and the inflorescence number. In this region, five polymorphic genes with missense variants were detected in E42. Similarly, on chromosome 4, three polymorphic genes showing missense variants effect colocalized with the QTL FRN4.1, which controls the number of fruits.

Data regarding genes with high and moderate impact variants were combined with those related to the 18 most polymorphic regions evidenced from the density distribution of SNPs and InDels across the E42 genome and those related to the colocalization with QTL regions involved in flower number, thus obtaining a subset of 35 genes mapping on 13 polymorphic regions (Table 2).

Among these, 24 genes were related to heat response and 11 to reproduction traits. Of the 35 genes, seven showed polymorphisms with high impact, which caused six frameshift variants, one frameshift variant and stop lost and splice region variant, one stop gain variant and one bidirectional gene fusion. Four of these genes were described as HSPs and three were related to flowering. One of the latest (Solyc11g008650) mapped in QTL regions involved in the number of flowers and flowering time of the first and second inflorescence. Finally, another 10 genes showing 19 missense variants were mapped in QTL regions involved in reproductive stages, such as flower and inflorescence production, flowering time of the first and second inflorescence.

Since the phylogenetic analysis evidenced the relationship of E42 genotype with the heat-tolerant *Solanum pimpinellifolium* wild species, we decided to focus on the group of 35 genes reported in Table 2 in order to deeply investigate the gene sequences in comparison with the ones of *Solanum lycopersicum* cv. Heinz and *Solanum pimpinellifolium* LA2093 accession (Tomato Genome version SL4.0 and 1.5 respectively) and to identify the putative wild origin of gene variants and/or sequences. Therefore, E42 FASTA gene sequences were aligned with the ones of Heinz and LA2093 (Supplementary Text S1) and the results of these comparisons were reported in Supplementary Table S10. The nucleotide alignments evidenced that eight E42 genes shared variants with LA2093, while in 14 genes the variants were private of the genotype. Interestingly the Solyc11g008650 nucleotide sequence of E42 shared 100% identity with the LA2093 accession, suggesting that this gene could be entirely introgressed from the wild species. In addition, this gene mapped into a genomic region including QTLs related to the flowering time of the first and second inflorescence. By contrast, one reproductive and five heat-related genes showing variants private of the referred genotype mapped into QTL regions involved in the total number of flowers and flowering time of the second inflorescence.

Finally, since the plant response to high temperatures could also be affected by the presence of heat stress elements (HSE) in the promoter regions that control the expression levels of heat stress-inducible genes, an in silico promoter analysis was performed on the 35 selected genes comparing the E42 and Heinz sequences, identifying polymorphisms in putative cis-acting elements (Supplementary Table S11). For most of the genes, the highest number of motifs was related to light-responsive elements, followed by hormone-responsive, environmental stress-related and developmental stress-related elements. The environmental stress-related elements involved ARE (essential for anaerobic induction), DRE (involved in dehydration, low temperatures and salt stress), GC (involved in anoxic specific inducibility), LTR (involved in low-temperature responsiveness), CCAAT-box (MYB binding sites), MBS, and MBSI (MYB binding sites involved in drought-inducibility and flavonoid biosynthetic genes regulation, respectively), TC-rich repeats (involved in defense and stress responsiveness) and WUN motives (wound responsive element) (Supplementary Table S12). Among these, the ARE motifs were the most frequent. Not one of the 35 promoters evidenced the presence of HSE motifs involved in heat stress response. Among the hormone-responsive elements, we found ABRE (involved in abscisic acid responsiveness), AuxRE, AuxRR-core, TGA-box and TGA-elements (involved in auxin responsiveness), CGTCA and TGACG motifs (involved in MeJA responsiveness), GARE, P-box and TATC-box (involved in gibberellin responsiveness) and TCA element (involved in salicylic acid responsiveness) (Supplementary Table S13). The highest frequencies were detected for ABRE, CGTCA and TGACG motifs. The Solyc01g079640 and Solyc11g008650 genes showed the highest number (seven) of mutated motifs in E42 compared to Heinz, followed by Solyc02g088610, Solyc05g053850 and Solyc07g065970 with six polymorphisms. Only Solyc03g122230, Solyc05g050820 and Solyc12g042560 displayed no variation along the promoter sequence.

## 4. Discussion

Temperature change and global warming have a significant impact on tomato yield affecting different aspects of plant development, including seed germination, vegetative growth, and reproduction [37]. Particularly, flowering, pollen viability, fruit set and fruit development are damaged at air temperature higher than 35 °C [38]. In this context, the priority of breeding programs is to develop heat-tolerant varieties that can survive under high temperatures and other biotic and abiotic stresses. In the present study, resequencing data of the E42 tomato thermotolerant genotype were exploited to investigate the whole genome and to identify candidate genes involved in the response of this genotype to heat stress.

The next-generation sequencing (NGS) technologies dramatically reduced the costs of sequencing and were used to understand the genome architecture, to discover SNP mutations and genome variations, and to identify QTLs and candidate genes for biotic and abiotic stresses [39,40]. Several species have been resequenced using a whole genome resequencing approach, including rice [41], maize [42], sorghum [43] and tomato [44,45]. Unlike RRS methods (GBS, single primer enrichment technology SPET, etc.) that screen random or specific fractions of the genome [46], resequencing technology covers the whole genome and aims at comparing genomic variability among individuals or populations when a reference genome is available for read mapping and variant identification [47]. E42 resequencing reads were mapped on the *Solanum lycopersicum* Heinz reference genome (Tomato Genome version SL4.0). Results evidenced a high number of variants across the genome of the referred genotype, most of which mapped on chromosomes 1, 4, 7 and 12 (92% of SNP and 67% of InDel), in accordance with the data of Olivieri et al. [11], who detected the highest number of polymorphisms on the same chromosomes of E42 when analyzing GBS data. In addition, the density distribution of SNPs and InDels through the E42 genome evidenced 18 highest polymorphic regions on eight chromosomes.

These polymorphisms allowed us to deeply investigate the origin of the E42 variability through a phylogenetic analysis of the whole sequenced genome of the referred genotype compared to 82 accessions belonging to 13 tomato species. The analysis evidenced that its four most polymorphic chromosomes (1, 4, 7 and 12) clustered not only with *S. lycopersicum* accessions but also with *S. pimpinellifolium* and with *S. galapagense* and *S. cheesmaniae* ones. These results were in accordance with the evidence of Olivieri et al. [11], who found a group of selected InDels on chromosomes 1 and 7 putatively introgressed from *S. pimpinellifolium* species. The architecture of the phylogenetic trees was in accordance with the classification in tomato clades proposed by Rodriguez et al. [48], who identified five clades: (1) a clade that includes *S. arcanum*, *S. chmielewskii* and *S. neorickii*; (2) a clade conformed by *S. chilense*, *S. corneliomulleri*, *S. peruvianum*, and the sister relationship between *S. corneliomulleri* and *S. peruvianum*; (3) a clade formed by *S. habrochaites* and *S. pennellii*; (4) a clade that includes *S. cheesmaniae* and *S. galapagense* and (5) a clade formed by *S. lycopersicum* and *S. pimpinellifolium*. This evidence suggests that the high variability detected in the E42 genome could be related to its origin, probably due to a breeding activity that involved at least *S. lycopersicum* and *S. pimpinellifolium* tomato species. Indeed, the 18 most polymorphic regions in the E42 genome, highlighted from density distribution of SNPs and InDels, could have been introgressed from the tomato wild species as a consequence of breeding activities and may include genes of interest in the response to high temperatures. It is well known that modern varieties are the result of intensive plant breeding programs, in which wild tomato species strongly contributed to this process as the main source of key genes in response to biotic and abiotic stresses. In particular, accessions from wild *Solanum* spp., such as *S. pimpinellifolium*, L., *S. pennellii* L., *S. habrochaites* L., *S. chmielewskii* L. and *S. cheesmaniae* L., have been demonstrated to be tolerant to high temperatures [49].

The tomato response to heat tolerance is a quantitative trait [34], and many genes are involved in this response, determining a complex of interactions among them, and dissecting this trait is a challenge that remains open. Not only have genomic variations in coding genes may imply a different heat response but also many other regulatory mechanisms been demonstrated to be involved in this response, such as (I) differences in the expression level of HSP genes regulated by HSFs [5]; (II) the noncoding RNAs (ncRNAs) that may regulate the activity of transcriptional factors (TFs) or genes [50]; (III) the epigenetic regulatory system involving DNA methylation, histone modification, and chromatin remodeling, which alter the gene expression pattern and/or epigenetic memory of plants under heat stress [50]. Since the E42 genotype selected in our laboratory was able to face high temperatures in different environmental and growth conditions [11,12,13], in this work we decided to focus on constitutive variations in the genome of E42 that could be responsible of its thermotolerance. Among the number of genes involved in determining its high- and stable-yield performances under heat, some genes and/or promoter regions and/or TFs would be constitutively polymorphic respect to more susceptible genotypes and we explored the genetic variability exhibited by E42 with this hypothesis in mind. First of all, investigating functional descriptions, we searched for polymorphic genes among those related to the heat response, mainly HSPs and HSFs, and those related to flowering, number of flowers, inflorescences and pollen development, which could influence the high number of fruits usually exhibited by the E42 genotype grown under high temperatures. Consequently, we obtained two lists of heat- and reproduction-related candidate genes, among which we identified polymorphisms putatively responsible for high and moderate impact changes on the related protein. Some of the variants occur in noncoding regions and others in the coding ones [51]. The SNPs entailing silent mutations occur in the noncoding regions and do not affect the protein sequence. However, silent mutations could also be found in the coding regions because each amino acid is coded by more than one codon. On the other hand, non-synonymous SNPs cause changes in the protein sequence. Among these, the nonsense mutations result in a premature stop codon allowing the production of nonfunctional proteins, while missense mutations produce the substitution of amino acids in the protein sequence [52]. According to this, we identified 49 heat- and 21 reproductive-related genes showing variants that imply missense variant, conservative in-frame insertion, conservative in-frame deletion, disruptive in-frame insertion, disruptive in-frame deletion, bidirectional gene fusion, stop gained variant and frameshift variant. Among these 70 genes, we decided to focus on those mapped in the 18 most polymorphic genome regions exhibited by E42 and/or QTL regions related to the number of flowers, thus obtaining a list of 35 genes, with 23 heat- and 12 reproductive-related genes. Among the first group of polymorphic genes, 16 genes code for HSP40 and HSP40-like and three for HSP70. It is reported that DnaJ proteins, also known as heat-shock protein 40 (HSP40), work as molecular chaperones independently or as co-chaperones of HSP70s [53]. In addition, the list also involved three short heat shock proteins (sHSPs) and one HSP110, known to be triggered by heat stress stimuli [54,55]. Lastly, variants occurred in a gene (Solyc07g055710) coding for HSFA4b which is reported to be a potent activator of heat stress gene expression [56,57]. The group of reproductive-related genes includes three genes classified as flowering locus T-like, which is the *Arabidopsis thaliana* ortholog of the SINGLE FLOWER TRUSS (SFT), reported to be the main gene involved in florigen activity. It is also reported that the *sft* mutant may disrupt normal tomato sympodial growth and allows the reversion of the inflorescence towards vegetative functioning after the development of one or few flowers [58,59]. In addition, it can interact with the flowering locus D gene to induce the transition of the shoot apical meristem to floral meristem [60]. By contrast, one gene coded for the SELF PRUNING (SP) (Solyc01g009580), which is the orthologue of terminal flower 1 in *Arabidopsis thaliana*, represses the floral transition in the sympodial meristems but does not alone play a role in inflorescence structure. Loss of function of SP gene leads to the shortening of successive sympodial segments up to the ultimate cessation of the iterative process, but does not affect inflorescence architecture [61]. Finally, five genes were found to be related to pollen development and are involved in pollen germination and pollen tube growth [62,63,64,65,66].

Results of sequence alignment showed that eight out of 35 genes shared all the high and moderate impact variants with the ones of the wild species LA2093, while other 13 genes also presented variants private of the referred genotype, in accordance with the phylogenetic and the density distribution of variants analysis. These findings allowed us to suggest that these genes could be strongly involved in the response of E42 to heat stress, since Zhou et al. [67] screened tomato heat-tolerant and heat-sensitive genotypes under heat stress selecting the LA2093 accession as tolerant to high temperatures. In particular, the Solyc11g008650 gene shared 100% identity with the sequence of the LA2093 accession, indicating its putative introgression from the *S. pimpinellifolium* wild ancestor. Song et al. [68] demonstrated that the two genes Solyc05g053850 and Solyc11g008650 were involved in day-neutral flowering time in *S. pimpinellifolium* tomato species. Interestingly, these two genes showed variants in E42 genotype compared with cv. Heinz, and further investigations will be carried out to understand their role in the inflorescence induction in the referred genotype. In addition, among the polymorphic genes in E42, five HSPs, two pollen- and one flowering-related genes also colocalized with the two QTLs FLN and Q-flnS, both involved in determining the number of flowers. These findings are in accordance with data reported by Ruggieri et al. [12] and Olivieri et al. [11,13], who evidenced the high number of flowers in the E42 genotype under both normal and heat stress conditions.

Finally, it is known from the literature that response mechanisms to heat stress also include signal perception, activation of HSFs, and production of HSPs, which are a prerequisite for protection from the stress and maintenance of protein homeostasis [69]. The HSF gene family is one of the most important TF families playing key roles in forming networks in the regulation of gene expression during heat stress response [70]. When exposed to high temperatures, heat shock genes are quickly expressed, resulting in a rapid transcription of HSPs [71]. Polymorphisms in the promoter regions could affect the number and the type of heat shock elements (HSEs), which provide DNA binding sites for HSFs. Given this, the promoter analysis was conducted on the reported 35 genes. Although the results evidenced the absence of the HSE motifs in the 35 selected genes, several E42 genes showed variations in the number of abscisic acid- and methyl jasmonate-related elements, hormones that are known to be involved in plant adaptation to external stimuli and to regulate diverse stress responses, including heat stress [72]. These outcomes suggest that these genes could be involved in ABA- and MeJA mediated regulatory network for plant stress response.

## 5. Conclusions

A plethora of genes, as well as genetic and epigenetic regulators, might affect the heat tolerance response in tomato, which depends on different interacting factors, first of all the specific genotype. In our laboratory, the selection of the stable high-yielding genotype E42 under different high temperatures conditions convinced us that some “constitutive” factors could determine its performance under stress. Therefore, the present investigation of the whole genome of E42, based on genomic and bioinformatic tools, led to the selection of a group of 35 genes, that could act as master regulators for the control of thermotolerance in E42. This group included 23 HSPs and one HSF, among those derived from a heat-related list of genes, six genes involved in flowering and five in pollen action, among those derived from a reproduction-related list. They were selected for their variants with high or moderate impact on the putative protein function, but even because they colocalized with some QTLs controlling flowering in tomato and mapped in polymorphic regions mostly deriving from the wild species *Solanum pimpinellifolium*, a species known to be heat-tolerant. With its list of selected genes, this study also paves the way for further genomics approaches aimed at increasing heat tolerance in tomato. Detailed studies on promoter regions and the expression of the selected genes under stress will be helpful to further reduce the putative number of master genes, whose role in thermotolerance will be validated by various strategies, including genome editing.

## Figures and Tables

**Table 1:** Number and chromosome distribution of the 86 QTLs related to reproduction traits and reported in the literature (2017–2022).

Trait	QTL No.	Chromosome
Flowering time	20	3, 4, 6, 7, 8, 9, 11
Flower number	11	1, 2, 3, 5, 6, 11
Inflorescence number	2	1, 3
Flowers per inflorescence	10	1, 2, 3, 4, 5, 12
Inflorescence with a single cyme	7	2, 5, 10, 12
Stigma length/protrusion/exertion	13	1, 2, 3, 4, 6, 7,
Anther length	3	1, 2, 7,
Pollen number	1	7
Pollen viability	1	11
Fruit set	8	3, 4, 7, 11, 12
Fruit number	10	1, 2, 3, 4, 6, 7, 9, 12

**Table 2:** List of the 35 genes selected from the heat- and reproduction-related lists mapping in the putative introgressed regions evidenced from SNP and InDel density distributions and colocalizing with QTL regions involved in flower number. Number of high and/or moderate variants in the genes, polymorphic regions, QTL colocalizations and gene functions are also reported. FLN = numbers of flowers, qCC = chlorophyll content, qREC = relative electrical conductivity, Q-fcsa = fruit cross-section area, Q-fpt = fruit pericarp thickness, Q-md = maturing time, Q-fo01 = flowering time of the first inflorescence, Q-fo02 = flowering time of the second inflorescence, Q-flnS = flower number per simple inflorescence.

Gene	High Variants, n	Moderate Variants, n	Polymorphic Region	QTL	Protein Function
Solyc01g009580	1	5	p1_3	-	Terminal flower 1
Solyc01g056310	0	2	p1_2	-	Anther-specific LAT51
Solyc01g066680	0	2	p1_5	-	Pollen Ole e 1 allergen and extensin family protein
Solyc01g066770	0	2	p1_5	-	Chaperone protein DnaJ 49
Solyc01g067780	0	14	p1_5	-	DnaJ domain
Solyc01g079610	0	1	p1_5	FLN1.1; qCC-1-4	DnaJ protein ERDJ3B
Solyc01g079640	0	1	p1_5	FLN1.1; qCC-1-4	Pollen-specific LRR extensin-like protein
Solyc01g086740	0	1	p1_5	FLN1.1; qCC-1-4	Chaperone protein DnaJ
Solyc01g088730	0	1	p1_5	FLN1.1; qREC-1-3; qCC-1-5	DnaJ domain-containing protein
Solyc02g088610	0	4	p2_1	Q-fcsa01; Q-fpt01; Q-md01	LeHSP110/ClpB heat shock protein
Solyc02g093600	1	2	-	FLN2.2; Q-flnS01	Class I heat shock protein
Solyc03g122230	0	3	-	Q-flnS02	Pollen receptor-like kinase 3
Solyc04g026280	0	2	p4_1	-	S1 self-incompatibility locus-linked pollen 3.15 protein
Solyc04g076270	0	1	p4_3	-	DnaJ domain-containing protein
Solyc04g077430	0	2	p4_3	-	Chaperone protein DnaJ
Solyc05g050820	0	3	-	FLN5.3	DnaJ homolog
Solyc05g051140	0	3	-	FLN5.3	Protein FLOWERING locus D-like protein
Solyc05g053760	2	0	p5_1	-	Chaperone protein DnaJ
Solyc05g053850	0	5	p5_1	-	Protein FLOWERING LOCUS T
Solyc05g055660	1	1	p5_1	-	Flowering locus T
Solyc07g021000	0	2	p7_1	-	FlowERING LOCUS D
Solyc07g026810	0	2	p7_2	-	Chaperone protein DnaJ
Solyc07g039220	2	12	p7_3	-	DNAJ heat shock N-terminal domain-containing protein
Solyc07g043560	0	2	p7_4	-	Heat shock protein 70 kDa
Solyc07g047690	0	3	p7_4	-	DnaJ domain-containing protein
Solyc07g053615	1	0	p7_4	-	DnaJ like protein
Solyc07g055710	0	1	p7_4	-	Heat stress transcription factor A-5
Solyc07g055720	0	1	p7_4	-	Heat shock protein 20
Solyc07g065970	0	1	p7_4	Q-fo02_03; Q-fo02_04	Chaperone protein DnaJ
Solyc07g066290	0	1	p7_4	Q-fo02_04	Chaperone DnaJ-domain containing protein
Solyc11g005400	0	1	p11_1	-	DnaJ domain
Solyc11g008650	1	1	p11_1	Q-fo01_05; Q_fo02_06	Flowering locus T1
Solyc12g042560	0	1	p12_2	-	Heat shock 70 kDa protein
Solyc12g042830	0	1	p12_2	-	Class I heat shock protein
Solyc12g043120	0	10	p12_2	-	Heat shock protein 70 family

## Data Availability

The data presented in this study are available on request from the corresponding author.

## Supplementary Materials

The following supporting information can be downloaded at https://www.mdpi.com/article/10.3390/genes14030535/s1. Supplementary Table S1: Results obtained from SNP calling analysis for each chromosome of E42 genotype compared with the tomato reference genome (Tomato Genome version SL4.0, www.solgenomics.net). Total number of SNPs, homozygous SNPs, total InDel variants, homozygous InDels are mentioned in the table. Supplementary Table S2: Data regarding the filtered merged VCF file of the 83 tomato accessions with 175,631 total raw variants. The number of variants and common variants of each accession with E42 is reported. Supplementary Table S3: Predicted effects of E42 polymorphisms compared with the tomato reference genome (Tomato Genome version SL4.0, www.solgenomics.net) obtained with SnpEff tool analysis. Supplementary Table S4: Results obtained from SnpEff analysis for each chromosome of E42 tomato genotype compared with the reference tomato genome (Tomato Genome version SL4.0, www.solgenomics.net). Total number of variants and high, moderate, low, and modifier impact variants in the gene regions. Supplementary Table S5: List of the 246 heat-related genes retrieved from Solgenomics database (ITAG4.1 versions of tomato genome annotation). For each gene, the “X” refers to the gene annotation (HSP or HSF). The number of high and moderate variants and the gene function annotation are also reported. Supplementary Table S6: List of the 49 heat-related genes showing high and/or moderate SNP/InDel variants. The mutation, the position referred to Tomato Genome version SL4.0 (www.solgenomics.net), the impact, the predicted effect of the variants and the protein function are also reported. Supplementary Table S7: List of the 83 reproduction-related genes retrieved from Solgenomics database (ITAG4.1 version of the tomato genome annotation). For each gene, the “X” refers to the flowering or pollen related function. The number of high and moderate impact variants and the gene function annotation are also reported. Supplementary Table S8: List of the 21 reproduction-related genes showing high and/or moderate SNP/InDel variants. The mutation, the position referring to the tomato genome version SL4.0 (www.solgenomics.net), the impact, the predicted effect of the variants and the protein function are also reported. Supplementary Table S9: List of QTLs related to reproduction and production under high temperatures reported in the literature in 2017–2022. For each QTL, the chromosome position is indicated by the flanking marker positions reported in the reference. Supplementary Table S10: Results of the multi-FASTA alignment of E42 with Heinz and LA2093 nucleotide and amino acid sequences of the 35 selected genes. E42 high and/or moderate impact variants in common with LA2093 accessions are marked “X”. The type of mutation, the position referred to the *Solanum lycopersicum* cv. Heinz version 4.0, the predicted impact and effect and the amino acid changes are also reported. Supplementary Table S11: In silico promoter analysis performed using PlantCare to identify putative cis-acting elements in the promoter region upstream of 3000 bp from the gene start site of the 35 E42 and Heinz gene sequences. Numbers of light-responsive, environmental stress-related, hormone-responsive, and development-related elements, are reported. Supplementary Table S12: List of the 35 selected genes showing the number of E42 ARE (essential for the anaerobic induction), DRE (involved in dehydration, low temperatures and salt stress), GC (involved in anoxic specific inducibility), LTR (involved in low temperature responsiveness), CCAAT-box (MYB binding sites), MBS, and MBSI (MYB binding sites involved in drought inducibility and flavonoid biosynthetic gene regulation), TC-rich repeats (involved in defense and stress responsiveness) and WUN motives (wound responsive element) environmental stress-related elements in comparison with Heinz obtained from PlantCARE analysis conducted on the promoter region upstream of 3000 bp from the gene start site. Supplementary Table S13: List of the 35 selected genes showing the number of E42 ABRE (involved in the abscisic acid responsiveness), AucRE, AuxRR-core, TGA-box and TGA elements (involved in auxin responsiveness), CGTCA and TGACG motifs (involved in MeJA responsiveness), GARE, P-box and TATC-box (involved in gibberellin responsiveness) and TCA element (involved in salicylic acid responsiveness) hormone-responsive elements in comparison with Heinz obtained from PlantCARE analysis conducted on the promoter region upstream of 3000 bp from the gene start site. Supplementary Figure S1: Phylogenetic trees of the 12 tomato chromosomes involving E42 and 82 accessions belonging to 13 tomato species. Supplementary Figure S2: SNP density of 1 Mb representation of the 12 E42 chromosomes compared with the ones of Heinz (Tomato Genome version SL4.0, available at the Solgenomics Network, www.solgenomics.net). Different QTL regions are indicated with stained boxes (see legend below the figure). Heat shock- and reproductive-related E42 genes with SNPs of high and/or moderate impact involved in high temperature response are indicated by red and green stars, respectively. FLN = numbers of flowers, FRN = numbers of fruits, FRS = fruit set proportion (FRS = 100 × FRN/FLN) and SE = stigma exertion [33]; flw = flowering time, ht = height, diam = stem diameter, fset = fruit set, fw = fruit weight, nflw = number of flowers, nfr = number of fruits, pH = acidity, SSC = soluble solid content, col_a = fruit color [32]; qPV = pollen viability, qPN = pollen number, qSP = style protrusion, qAL = anther length, qSL = style length, FPI = flowers per inflorescence qIN = inflorescence number [36]; qREC = relative electrical conductivity, qCC = chlorophyll content, qFv/Fm = maximum photochemical quantum efficiency of photosystem II [34]; Q_fc = immature fruit color, Q-ab01 = length of the first axillary branch, Q-ab02 = length of the second axillary branch, Q-fcsa = fruit cross section area, Q-fln = flower number per inflorescence, Q-flnS = flower number per simple inflorescence, Q-fmh = fruit maximum height, Q-fmw = Fruit maximum width, Q-fo01 = flowering time of the first inflorescence, Q-fo02 = flowering time of the second inflorescence, Q-fo03 = flowering time of the third inflorescence, Q-fp = fruit perimeter, Q-fpt = fruit pericarp thickness, Q-fs = green fruit shoulder, Q-fw = fruit weight, Q-ins = percentage of inflorescences with single cyme, Q-md = maturing time, Q-sp = stem pubescence [35]. Supplementary Text S1: Multi-FASTA alignment of E42 with Heinz and LA2093 nucleotide sequences of the 35 selected genes. Bold-red characters indicate high and/or moderate variant sites.
