# Supplementary material for: Genomic Insights into the Origin of a Thermotolerant Tomato Line and Identification of Candidate Genes for Heat Stress
Source: Genes (Basel). 2023 Feb 21;14(3):535. doi: 10.3390/genes14030535 (PMC10048601; doi:10.3390/genes14030535)
Supplement: Supplementary file 1 [file genes-14-00535-s001.zip › Supplementary Figure S1.pdf]

Ch01

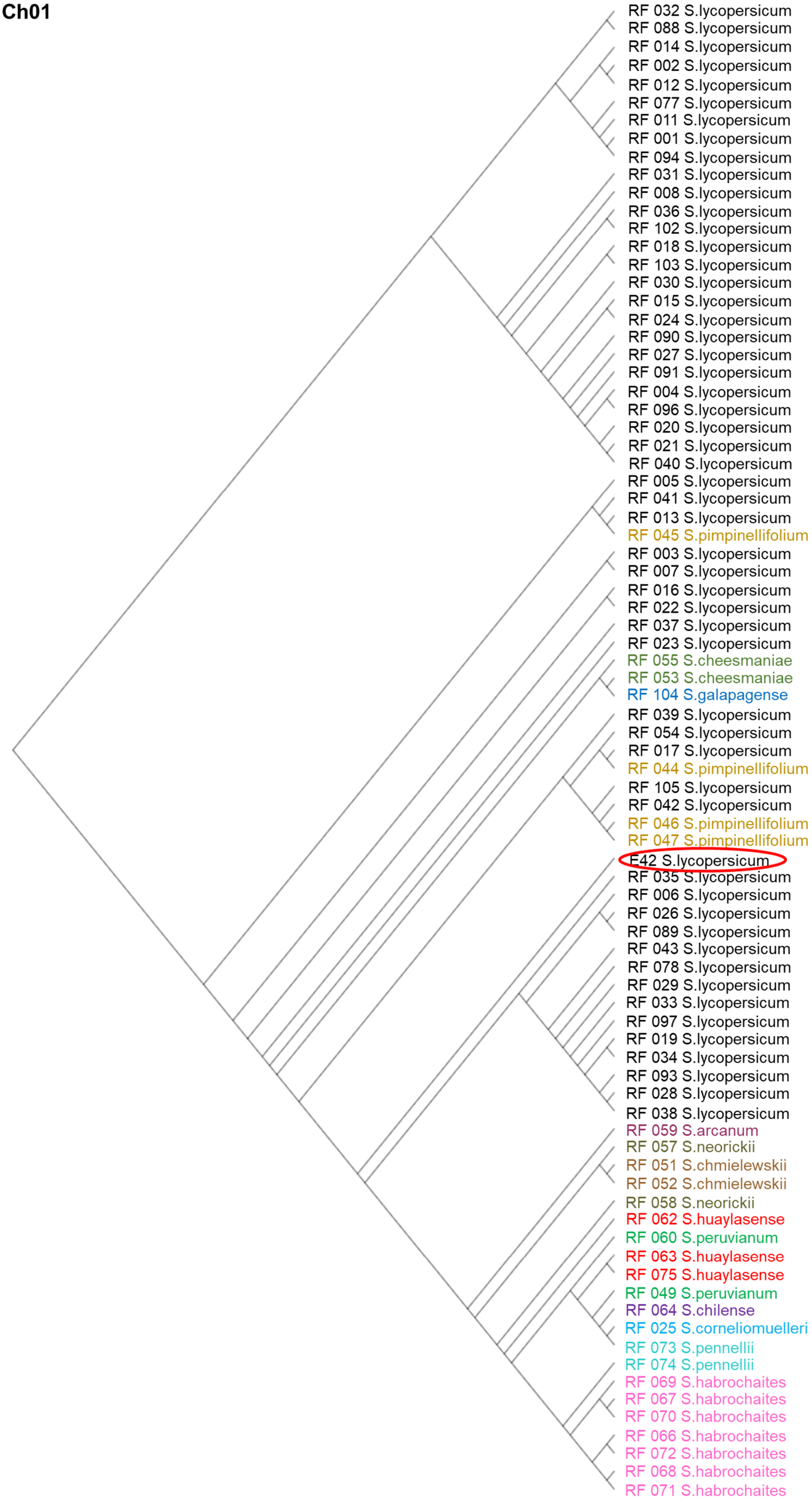

RF 032 S.lycopersicum
RF 088 S.lycopersicum
RF 014 S.lycopersicum
RF 002 S.lycopersicum
RF 012 S.lycopersicum
RF 077 S.lycopersicum
RF 011 S.lycopersicum
RF 001 S.lycopersicum
RF 094 S.lycopersicum
RF 031 S.lycopersicum
RF 008 S.lycopersicum
RF 036 S.lycopersicum
RF 102 S.lycopersicum
RF 018 S.lycopersicum
RF 103 S.lycopersicum
RF 030 S.lycopersicum
RF 015 S.lycopersicum
RF 024 S.lycopersicum
RF 090 S.lycopersicum
RF 027 S.lycopersicum
RF 091 S.lycopersicum
RF 004 S.lycopersicum
RF 096 S.lycopersicum
RF 020 S.lycopersicum
RF 021 S.lycopersicum
RF 040 S.lycopersicum
RF 005 S.lycopersicum
RF 041 S.lycopersicum
RF 013 S.lycopersicum
RF 045 S.pimpinellifolium
RF 003 S.lycopersicum
RF 007 S.lycopersicum
RF 016 S.lycopersicum
RF 022 S.lycopersicum
RF 037 S.lycopersicum
RF 023 S.lycopersicum
RF 055 S.cheesmaniae
RF 053 S.cheesmaniae
RF 104 S.galapagense
RF 039 S.lycopersicum
RF 054 S.lycopersicum
RF 017 S.lycopersicum
RF 044 S.pimpinellifolium
RF 105 S.lycopersicum
RF 042 S.lycopersicum
RF 046 S.pimpinellifolium
RF 047 S.pimpinellifolium
E42 S.lycopersicum
RF 035 S.lycopersicum
RF 006 S.lycopersicum
RF 026 S.lycopersicum
RF 089 S.lycopersicum
RF 043 S.lycopersicum
RF 078 S.lycopersicum
RF 029 S.lycopersicum
RF 033 S.lycopersicum
RF 097 S.lycopersicum
RF 019 S.lycopersicum
RF 034 S.lycopersicum
RF 093 S.lycopersicum
RF 028 S.lycopersicum
RF 038 S.lycopersicum
RF 059 S.arcanum
RF 057 S.neorickii
RF 051 S.chmielewskii
RF 052 S.chmielewskii
RF 058 S.neorickii
RF 062 S.huaylasense
RF 060 S.peruvianum
RF 063 S.huaylasense
RF 075 S.huaylasense
RF 049 S.peruvianum
RF 064 S.chilense
RF 025 S.corneliomuelleri
RF 073 S.pennellii
RF 074 S.pennellii
RF 069 S.habrochaites
RF 067 S.habrochaites
RF 070 S.habrochaites
RF 066 S.habrochaites
RF 072 S.habrochaites
RF 068 S.habrochaites
RF 071 S.habrochaites

Ch02

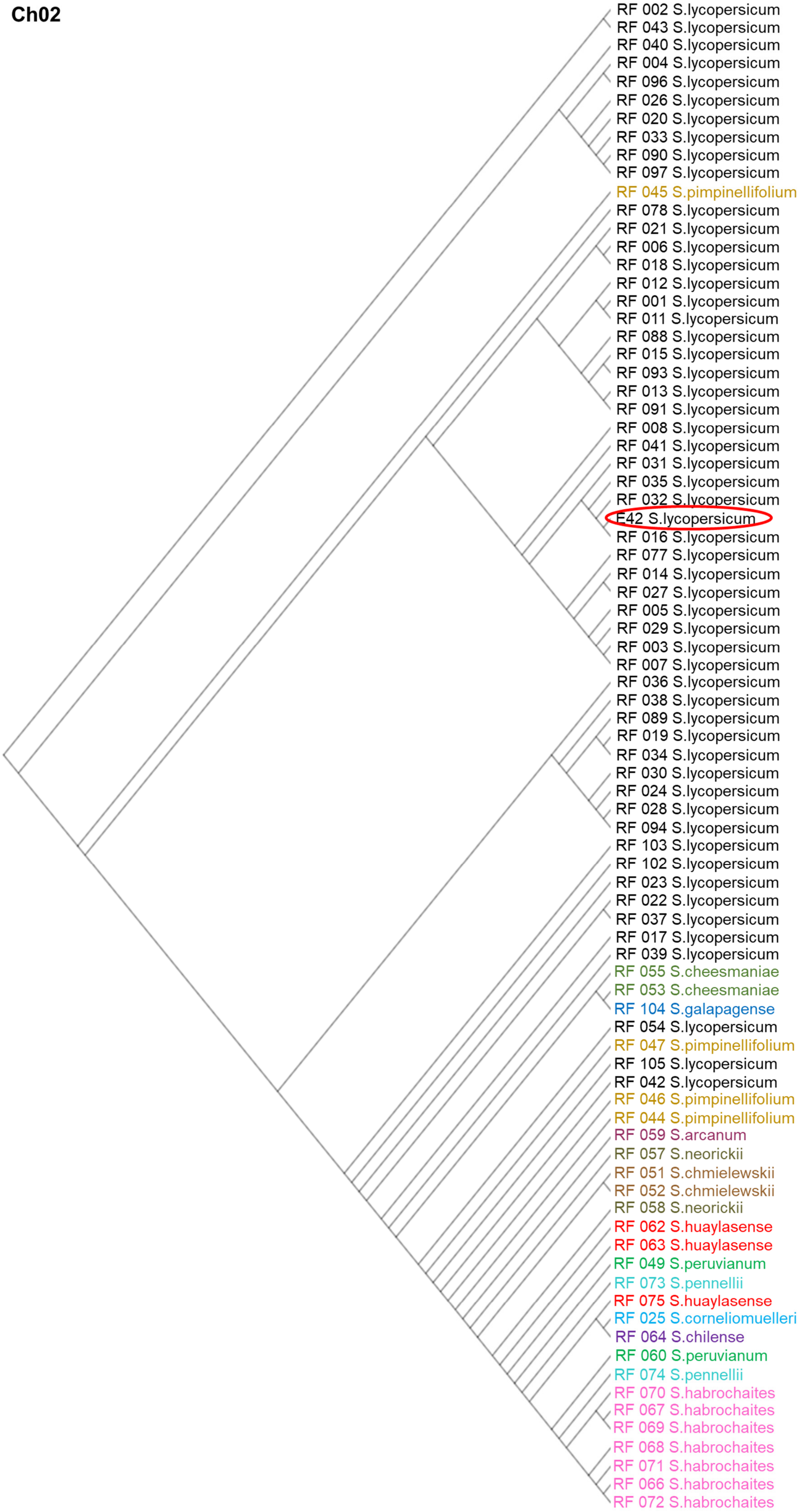
RF 002 S.lycopersicum
RF 043 S.lycopersicum
RF 040 S.lycopersicum
RF 004 S.lycopersicum
RF 096 S.lycopersicum
RF 026 S.lycopersicum
RF 020 S.lycopersicum
RF 033 S.lycopersicum
RF 090 S.lycopersicum
RF 097 S.lycopersicum
RF 045 S.pimpinellifolium
RF 078 S.lycopersicum
RF 021 S.lycopersicum
RF 006 S.lycopersicum
RF 018 S.lycopersicum
RF 012 S.lycopersicum
RF 001 S.lycopersicum
RF 011 S.lycopersicum
RF 088 S.lycopersicum
RF 015 S.lycopersicum
RF 093 S.lycopersicum
RF 013 S.lycopersicum
RF 091 S.lycopersicum
RF 008 S.lycopersicum
RF 041 S.lycopersicum
RF 031 S.lycopersicum
RF 035 S.lycopersicum
RF 032 S.lycopersicum
E42 S.lycopersicum
RF 016 S.lycopersicum
RF 077 S.lycopersicum
RF 014 S.lycopersicum
RF 027 S.lycopersicum
RF 005 S.lycopersicum
RF 029 S.lycopersicum
RF 003 S.lycopersicum
RF 007 S.lycopersicum
RF 036 S.lycopersicum
RF 038 S.lycopersicum
RF 089 S.lycopersicum
RF 019 S.lycopersicum
RF 034 S.lycopersicum
RF 030 S.lycopersicum
RF 024 S.lycopersicum
RF 028 S.lycopersicum
RF 094 S.lycopersicum
RF 103 S.lycopersicum
RF 102 S.lycopersicum
RF 023 S.lycopersicum
RF 022 S.lycopersicum
RF 037 S.lycopersicum
RF 017 S.lycopersicum
RF 039 S.lycopersicum
RF 055 S.cheesmaniae
RF 053 S.cheesmaniae
RF 104 S.galapagense
RF 054 S.lycopersicum
RF 047 S.pimpinellifolium
RF 105 S.lycopersicum
RF 042 S.lycopersicum
RF 046 S.pimpinellifolium
RF 044 S.pimpinellifolium
RF 059 S.arcanum
RF 057 S.neorickii
RF 051 S.chmielewskii
RF 052 S.chmielewskii
RF 058 S.neorickii
RF 062 S.huaylasense
RF 063 S.huaylasense
RF 049 S.peruvianum
RF 073 S.pennellii
RF 075 S.huaylasense
RF 025 S.corneliomuelleri
RF 064 S.chilense
RF 060 S.peruvianum
RF 074 S.pennellii
RF 070 S.habrochaites
RF 067 S.habrochaites
RF 069 S.habrochaites
RF 068 S.habrochaites
RF 071 S.habrochaites
RF 066 S.habrochaites
RF 072 S.habrochaites

Ch03

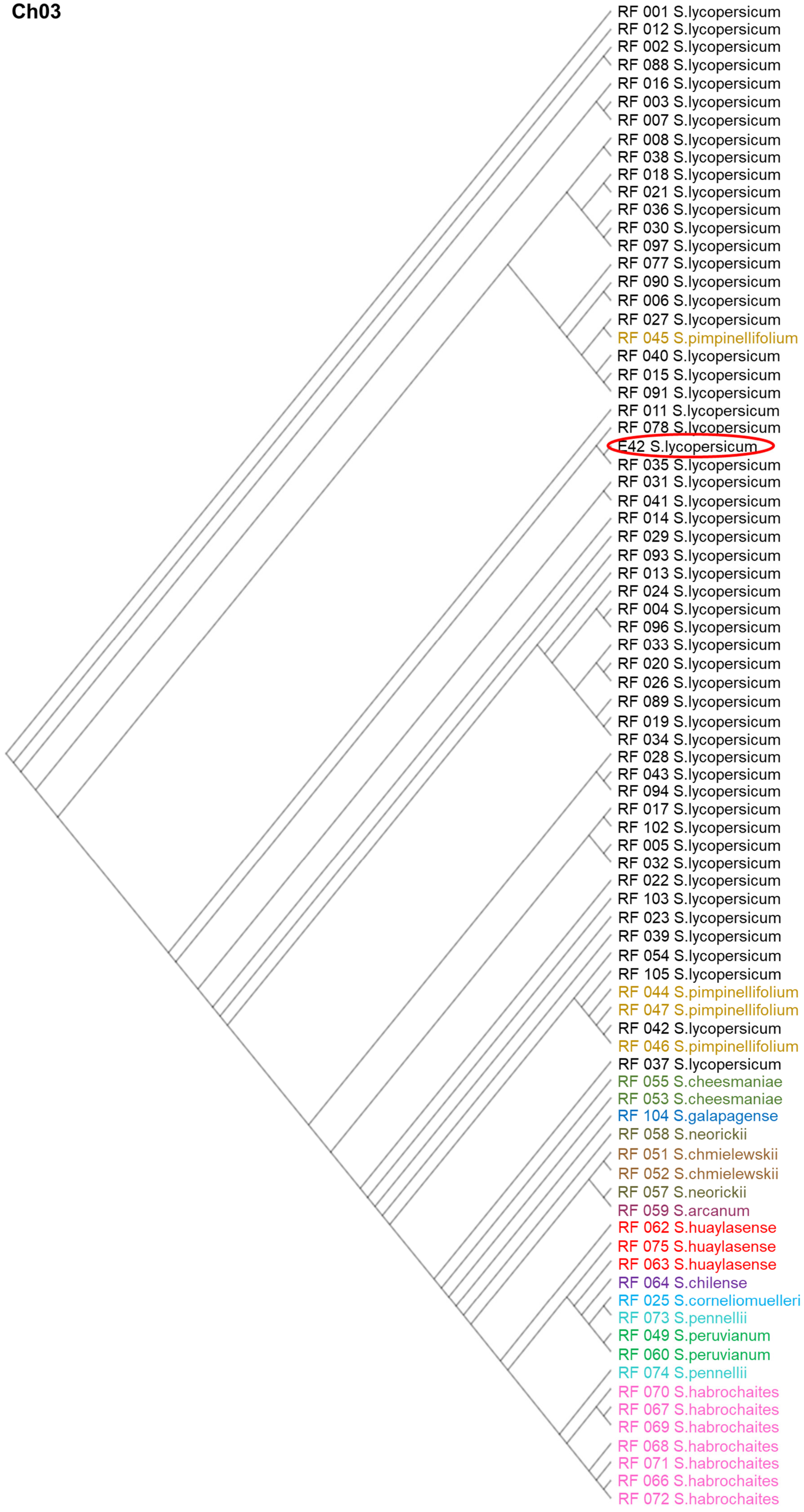
RF 001 S.lycopersicum
RF 012 S.lycopersicum
RF 002 S.lycopersicum
RF 088 S.lycopersicum
RF 016 S.lycopersicum
RF 003 S.lycopersicum
RF 007 S.lycopersicum
RF 008 S.lycopersicum
RF 038 S.lycopersicum
RF 018 S.lycopersicum
RF 021 S.lycopersicum
RF 036 S.lycopersicum
RF 030 S.lycopersicum
RF 097 S.lycopersicum
RF 077 S.lycopersicum
RF 090 S.lycopersicum
RF 006 S.lycopersicum
RF 027 S.lycopersicum
RF 045 S.pimpinellifolium
RF 040 S.lycopersicum
RF 015 S.lycopersicum
RF 091 S.lycopersicum
RF 011 S.lycopersicum
RF 078 S.lycopersicum
F42 S.lycopersicum
RF 035 S.lycopersicum
RF 031 S.lycopersicum
RF 041 S.lycopersicum
RF 014 S.lycopersicum
RF 029 S.lycopersicum
RF 093 S.lycopersicum
RF 013 S.lycopersicum
RF 024 S.lycopersicum
RF 004 S.lycopersicum
RF 096 S.lycopersicum
RF 033 S.lycopersicum
RF 020 S.lycopersicum
RF 026 S.lycopersicum
RF 089 S.lycopersicum
RF 019 S.lycopersicum
RF 034 S.lycopersicum
RF 028 S.lycopersicum
RF 043 S.lycopersicum
RF 094 S.lycopersicum
RF 017 S.lycopersicum
RF 102 S.lycopersicum
RF 005 S.lycopersicum
RF 032 S.lycopersicum
RF 022 S.lycopersicum
RF 103 S.lycopersicum
RF 023 S.lycopersicum
RF 039 S.lycopersicum
RF 054 S.lycopersicum
RF 105 S.lycopersicum
RF 044 S.pimpinellifolium
RF 047 S.pimpinellifolium
RF 042 S.lycopersicum
RF 046 S.pimpinellifolium
RF 037 S.lycopersicum
RF 055 S.cheesmaniae
RF 053 S.cheesmaniae
RF 104 S.galapagense
RF 058 S.neorickii
RF 051 S.chmielewskii
RF 052 S.chmielewskii
RF 057 S.neorickii
RF 059 S.arcanum
RF 062 S.huaylasense
RF 075 S.huaylasense
RF 063 S.huaylasense
RF 064 S.chilense
RF 025 S.corneliomuelleri
RF 073 S.pennellii
RF 049 S.peruvianum
RF 060 S.peruvianum
RF 074 S.pennellii
RF 070 S.habrochaites
RF 067 S.habrochaites
RF 069 S.habrochaites
RF 068 S.habrochaites
RF 071 S.habrochaites
RF 066 S.habrochaites
RF 072 S.habrochaites

Ch04

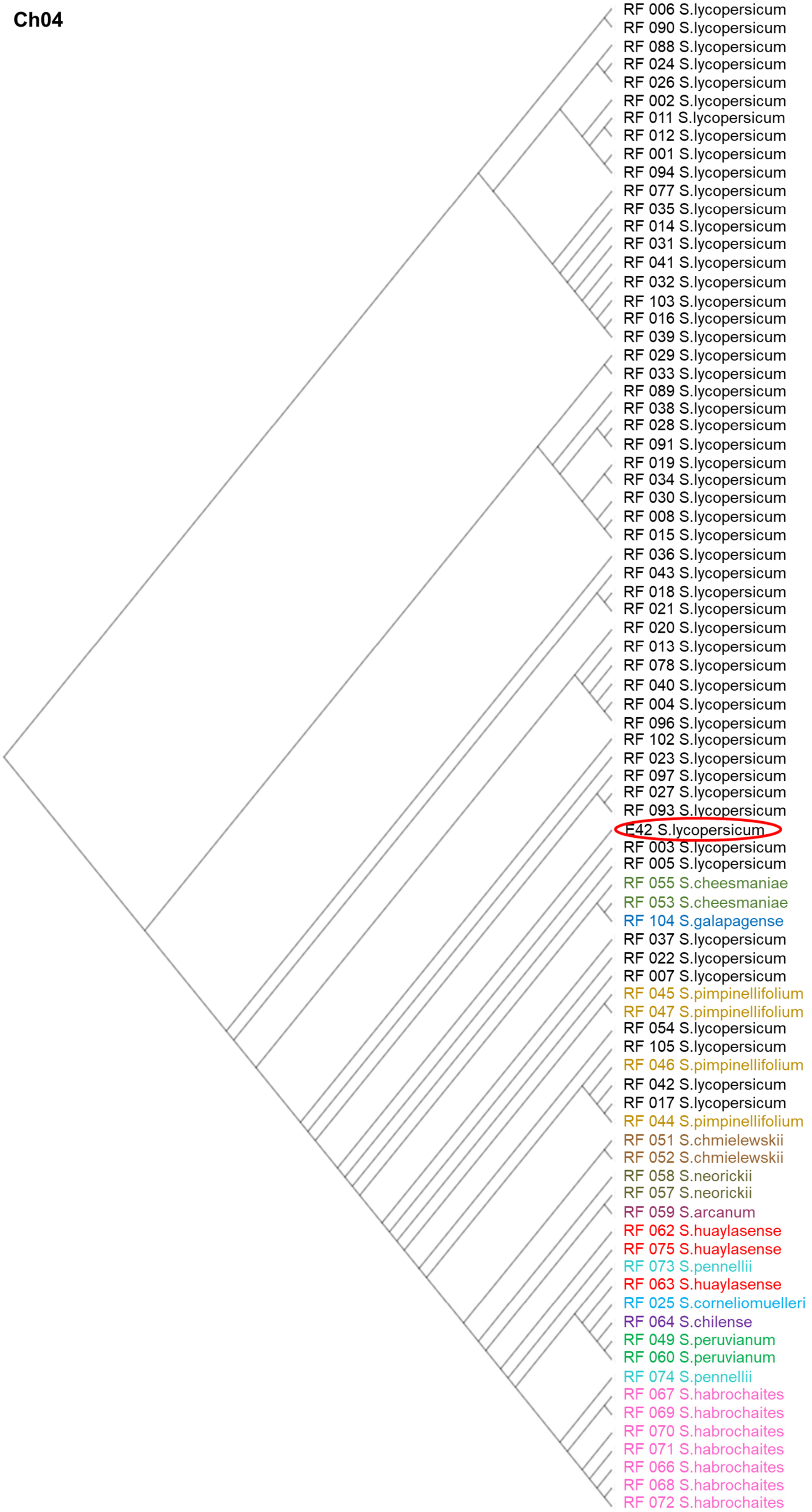
RF 006 S.lycopersicum
RF 090 S.lycopersicum
RF 088 S.lycopersicum
RF 024 S.lycopersicum
RF 026 S.lycopersicum
RF 002 S.lycopersicum
RF 011 S.lycopersicum
RF 012 S.lycopersicum
RF 001 S.lycopersicum
RF 094 S.lycopersicum
RF 077 S.lycopersicum
RF 035 S.lycopersicum
RF 014 S.lycopersicum
RF 031 S.lycopersicum
RF 041 S.lycopersicum
RF 032 S.lycopersicum
RF 103 S.lycopersicum
RF 016 S.lycopersicum
RF 039 S.lycopersicum
RF 029 S.lycopersicum
RF 033 S.lycopersicum
RF 089 S.lycopersicum
RF 038 S.lycopersicum
RF 028 S.lycopersicum
RF 091 S.lycopersicum
RF 019 S.lycopersicum
RF 034 S.lycopersicum
RF 030 S.lycopersicum
RF 008 S.lycopersicum
RF 015 S.lycopersicum
RF 036 S.lycopersicum
RF 043 S.lycopersicum
RF 018 S.lycopersicum
RF 021 S.lycopersicum
RF 020 S.lycopersicum
RF 013 S.lycopersicum
RF 078 S.lycopersicum
RF 040 S.lycopersicum
RF 004 S.lycopersicum
RF 096 S.lycopersicum
RF 102 S.lycopersicum
RF 023 S.lycopersicum
RF 097 S.lycopersicum
RF 027 S.lycopersicum
RF 093 S.lycopersicum
E42 S.lycopersicum
RF 003 S.lycopersicum
RF 005 S.lycopersicum
RF 055 S.cheesmaniae
RF 053 S.cheesmaniae
RF 104 S.galapagense
RF 037 S.lycopersicum
RF 022 S.lycopersicum
RF 007 S.lycopersicum
RF 045 S.pimpinellifolium
RF 047 S.pimpinellifolium
RF 054 S.lycopersicum
RF 105 S.lycopersicum
RF 046 S.pimpinellifolium
RF 042 S.lycopersicum
RF 017 S.lycopersicum
RF 044 S.pimpinellifolium
RF 051 S.chmielewskii
RF 052 S.chmielewskii
RF 058 S.neorickii
RF 057 S.neorickii
RF 059 S.arcanum
RF 062 S.huaylasense
RF 075 S.huaylasense
RF 073 S.pennellii
RF 063 S.huaylasense
RF 025 S.corneliomuelleri
RF 064 S.chilense
RF 049 S.peruvianum
RF 060 S.peruvianum
RF 074 S.pennellii
RF 067 S.habrochaites
RF 069 S.habrochaites
RF 070 S.habrochaites
RF 071 S.habrochaites
RF 066 S.habrochaites
RF 068 S.habrochaites
RF 072 S.habrochaites

Ch05

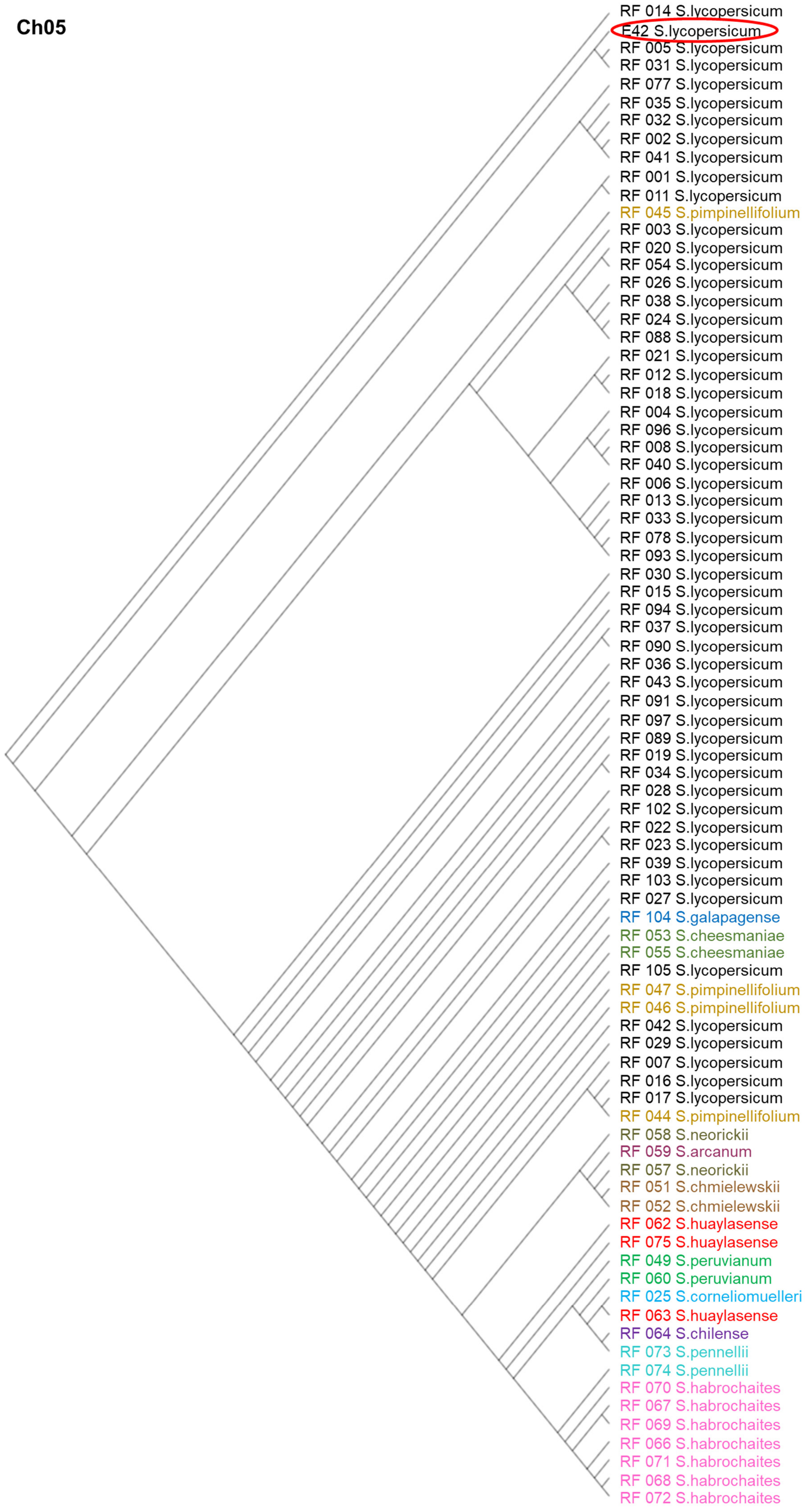

RF 014 S.lycopersicum
E42 S.lycopersicum
RF 005 S.lycopersicum
RF 031 S.lycopersicum
RF 077 S.lycopersicum
RF 035 S.lycopersicum
RF 032 S.lycopersicum
RF 002 S.lycopersicum
RF 041 S.lycopersicum
RF 001 S.lycopersicum
RF 011 S.lycopersicum
RF 045 S.pimpinellifolium
RF 003 S.lycopersicum
RF 020 S.lycopersicum
RF 054 S.lycopersicum
RF 026 S.lycopersicum
RF 038 S.lycopersicum
RF 024 S.lycopersicum
RF 088 S.lycopersicum
RF 021 S.lycopersicum
RF 012 S.lycopersicum
RF 018 S.lycopersicum
RF 004 S.lycopersicum
RF 096 S.lycopersicum
RF 008 S.lycopersicum
RF 040 S.lycopersicum
RF 006 S.lycopersicum
RF 013 S.lycopersicum
RF 033 S.lycopersicum
RF 078 S.lycopersicum
RF 093 S.lycopersicum
RF 030 S.lycopersicum
RF 015 S.lycopersicum
RF 094 S.lycopersicum
RF 037 S.lycopersicum
RF 090 S.lycopersicum
RF 036 S.lycopersicum
RF 043 S.lycopersicum
RF 091 S.lycopersicum
RF 097 S.lycopersicum
RF 089 S.lycopersicum
RF 019 S.lycopersicum
RF 034 S.lycopersicum
RF 028 S.lycopersicum
RF 102 S.lycopersicum
RF 022 S.lycopersicum
RF 023 S.lycopersicum
RF 039 S.lycopersicum
RF 103 S.lycopersicum
RF 027 S.lycopersicum
RF 104 S.galapagense
RF 053 S.cheesmaniae
RF 055 S.cheesmaniae
RF 105 S.lycopersicum
RF 047 S.pimpinellifolium
RF 046 S.pimpinellifolium
RF 042 S.lycopersicum
RF 029 S.lycopersicum
RF 007 S.lycopersicum
RF 016 S.lycopersicum
RF 017 S.lycopersicum
RF 044 S.pimpinellifolium
RF 058 S.neorickii
RF 059 S.arcanum
RF 057 S.neorickii
RF 051 S.chmielewskii
RF 052 S.chmielewskii
RF 062 S.huaylasense
RF 075 S.huaylasense
RF 049 S.peruvianum
RF 060 S.peruvianum
RF 025 S.corneliomuelleri
RF 063 S.huaylasense
RF 064 S.chilense
RF 073 S.pennellii
RF 074 S.pennellii
RF 070 S.habrochaites
RF 067 S.habrochaites
RF 069 S.habrochaites
RF 066 S.habrochaites
RF 071 S.habrochaites
RF 068 S.habrochaites
RF 072 S.habrochaites

Ch06

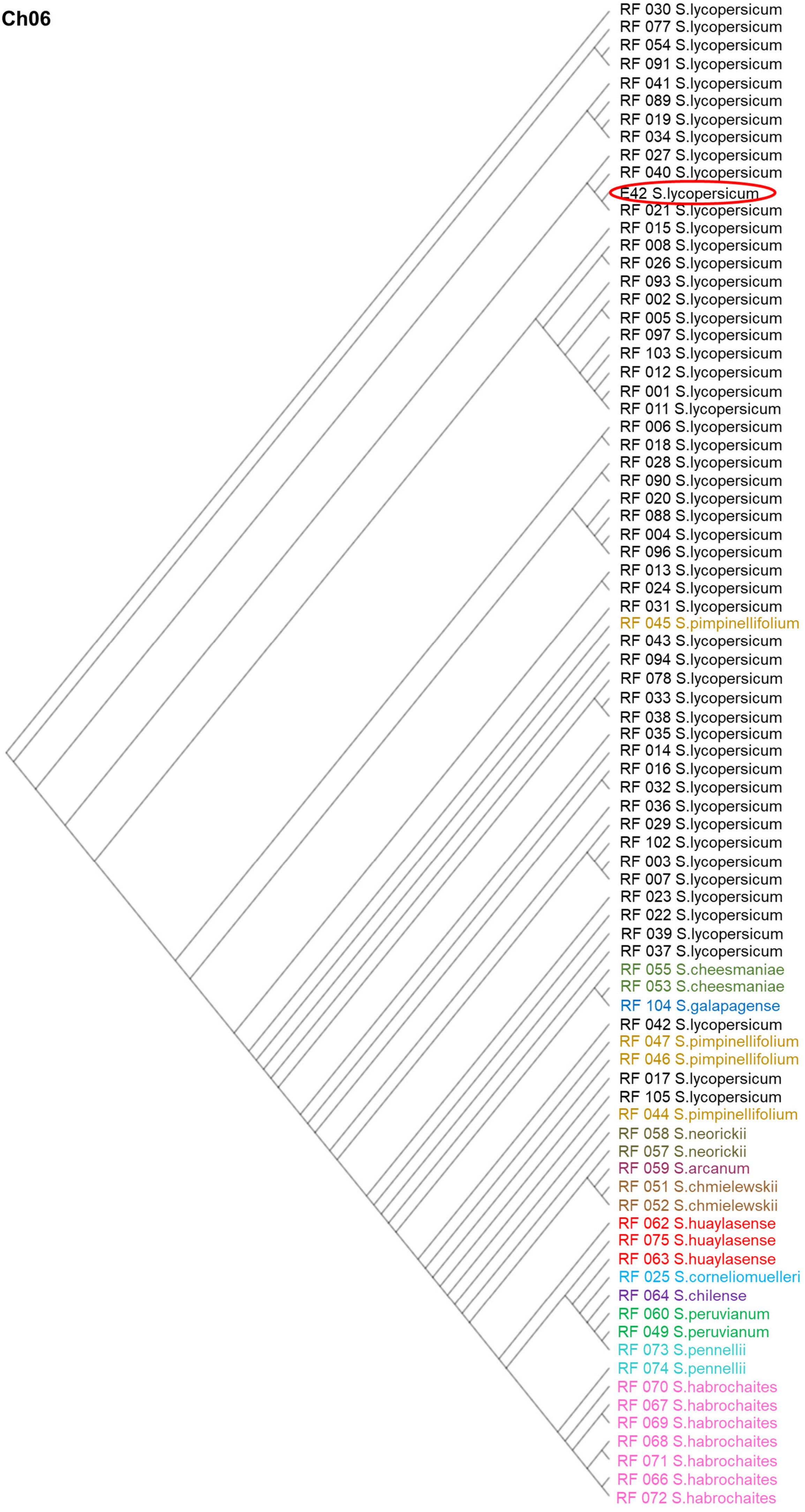
RF 030 S.lycopersicum
RF 077 S.lycopersicum
RF 054 S.lycopersicum
RF 091 S.lycopersicum
RF 041 S.lycopersicum
RF 089 S.lycopersicum
RF 019 S.lycopersicum
RF 034 S.lycopersicum
RF 027 S.lycopersicum
RF 040 S.lycopersicum
E42 S.lycopersicum
RF 021 S.lycopersicum
RF 015 S.lycopersicum
RF 008 S.lycopersicum
RF 026 S.lycopersicum
RF 093 S.lycopersicum
RF 002 S.lycopersicum
RF 005 S.lycopersicum
RF 097 S.lycopersicum
RF 103 S.lycopersicum
RF 012 S.lycopersicum
RF 001 S.lycopersicum
RF 011 S.lycopersicum
RF 006 S.lycopersicum
RF 018 S.lycopersicum
RF 028 S.lycopersicum
RF 090 S.lycopersicum
RF 020 S.lycopersicum
RF 088 S.lycopersicum
RF 004 S.lycopersicum
RF 096 S.lycopersicum
RF 013 S.lycopersicum
RF 024 S.lycopersicum
RF 031 S.lycopersicum
RF 045 S.pimpinellifolium
RF 043 S.lycopersicum
RF 094 S.lycopersicum
RF 078 S.lycopersicum
RF 033 S.lycopersicum
RF 038 S.lycopersicum
RF 035 S.lycopersicum
RF 014 S.lycopersicum
RF 016 S.lycopersicum
RF 032 S.lycopersicum
RF 036 S.lycopersicum
RF 029 S.lycopersicum
RF 102 S.lycopersicum
RF 003 S.lycopersicum
RF 007 S.lycopersicum
RF 023 S.lycopersicum
RF 022 S.lycopersicum
RF 039 S.lycopersicum
RF 037 S.lycopersicum
RF 055 S.cheesmaniae
RF 053 S.cheesmaniae
RF 104 S.galapagense
RF 042 S.lycopersicum
RF 047 S.pimpinellifolium
RF 046 S.pimpinellifolium
RF 017 S.lycopersicum
RF 105 S.lycopersicum
RF 044 S.pimpinellifolium
RF 058 S.neorickii
RF 057 S.neorickii
RF 059 S.arcanum
RF 051 S.chmielewskii
RF 052 S.chmielewskii
RF 062 S.huaylasense
RF 075 S.huaylasense
RF 063 S.huaylasense
RF 025 S.corneliomuelleri
RF 064 S.chilense
RF 060 S.peruvianum
RF 049 S.peruvianum
RF 073 S.pennellii
RF 074 S.pennellii
RF 070 S.habrochaites
RF 067 S.habrochaites
RF 069 S.habrochaites
RF 068 S.habrochaites
RF 071 S.habrochaites
RF 066 S.habrochaites
RF 072 S.habrochaites

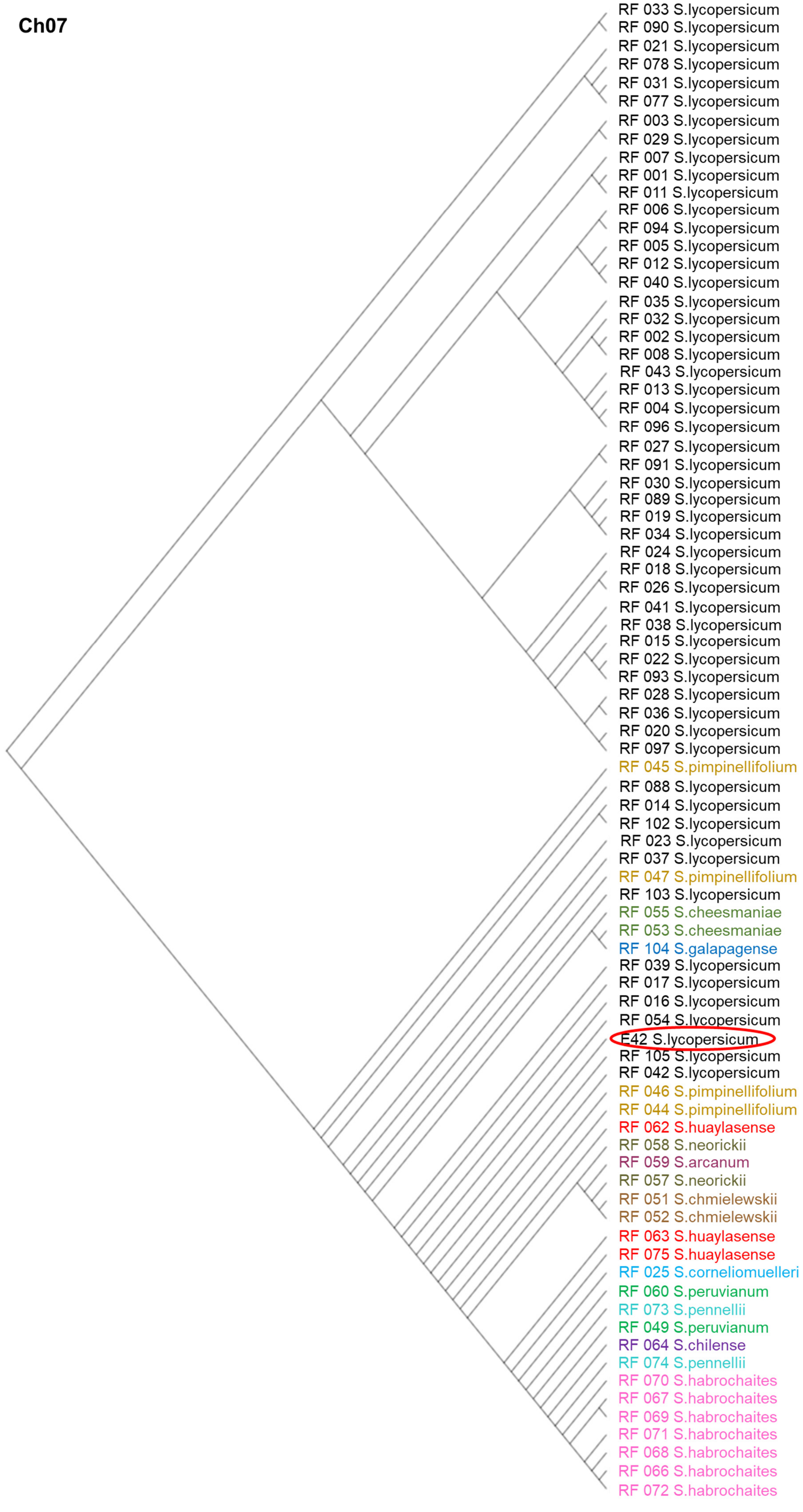
Ch07
RF 033 S.lycopersicum
RF 090 S.lycopersicum
RF 021 S.lycopersicum
RF 078 S.lycopersicum
RF 031 S.lycopersicum
RF 077 S.lycopersicum
RF 003 S.lycopersicum
RF 029 S.lycopersicum
RF 007 S.lycopersicum
RF 001 S.lycopersicum
RF 011 S.lycopersicum
RF 006 S.lycopersicum
RF 094 S.lycopersicum
RF 005 S.lycopersicum
RF 012 S.lycopersicum
RF 040 S.lycopersicum
RF 035 S.lycopersicum
RF 032 S.lycopersicum
RF 002 S.lycopersicum
RF 008 S.lycopersicum
RF 043 S.lycopersicum
RF 013 S.lycopersicum
RF 004 S.lycopersicum
RF 096 S.lycopersicum
RF 027 S.lycopersicum
RF 091 S.lycopersicum
RF 030 S.lycopersicum
RF 089 S.lycopersicum
RF 019 S.lycopersicum
RF 034 S.lycopersicum
RF 024 S.lycopersicum
RF 018 S.lycopersicum
RF 026 S.lycopersicum
RF 041 S.lycopersicum
RF 038 S.lycopersicum
RF 015 S.lycopersicum
RF 022 S.lycopersicum
RF 093 S.lycopersicum
RF 028 S.lycopersicum
RF 036 S.lycopersicum
RF 020 S.lycopersicum
RF 097 S.lycopersicum
RF 045 S.pimpinellifolium
RF 088 S.lycopersicum
RF 014 S.lycopersicum
RF 102 S.lycopersicum
RF 023 S.lycopersicum
RF 037 S.lycopersicum
RF 047 S.pimpinellifolium
RF 103 S.lycopersicum
RF 055 S.cheesmaniae
RF 053 S.cheesmaniae
RF 104 S.galapagense
RF 039 S.lycopersicum
RF 017 S.lycopersicum
RF 016 S.lycopersicum
RF 054 S.lycopersicum
E42 S.lycopersicum
RF 105 S.lycopersicum
RF 042 S.lycopersicum
RF 046 S.pimpinellifolium
RF 044 S.pimpinellifolium
RF 062 S.huaylasense
RF 058 S.neorickii
RF 059 S.arcanum
RF 057 S.neorickii
RF 051 S.chmielewskii
RF 052 S.chmielewskii
RF 063 S.huaylasense
RF 075 S.huaylasense
RF 025 S.corneliomuelleri
RF 060 S.peruvianum
RF 073 S.pennellii
RF 049 S.peruvianum
RF 064 S.chilense
RF 074 S.pennellii
RF 070 S.habrochaites
RF 067 S.habrochaites
RF 069 S.habrochaites
RF 071 S.habrochaites
RF 068 S.habrochaites
RF 066 S.habrochaites
RF 072 S.habrochaites

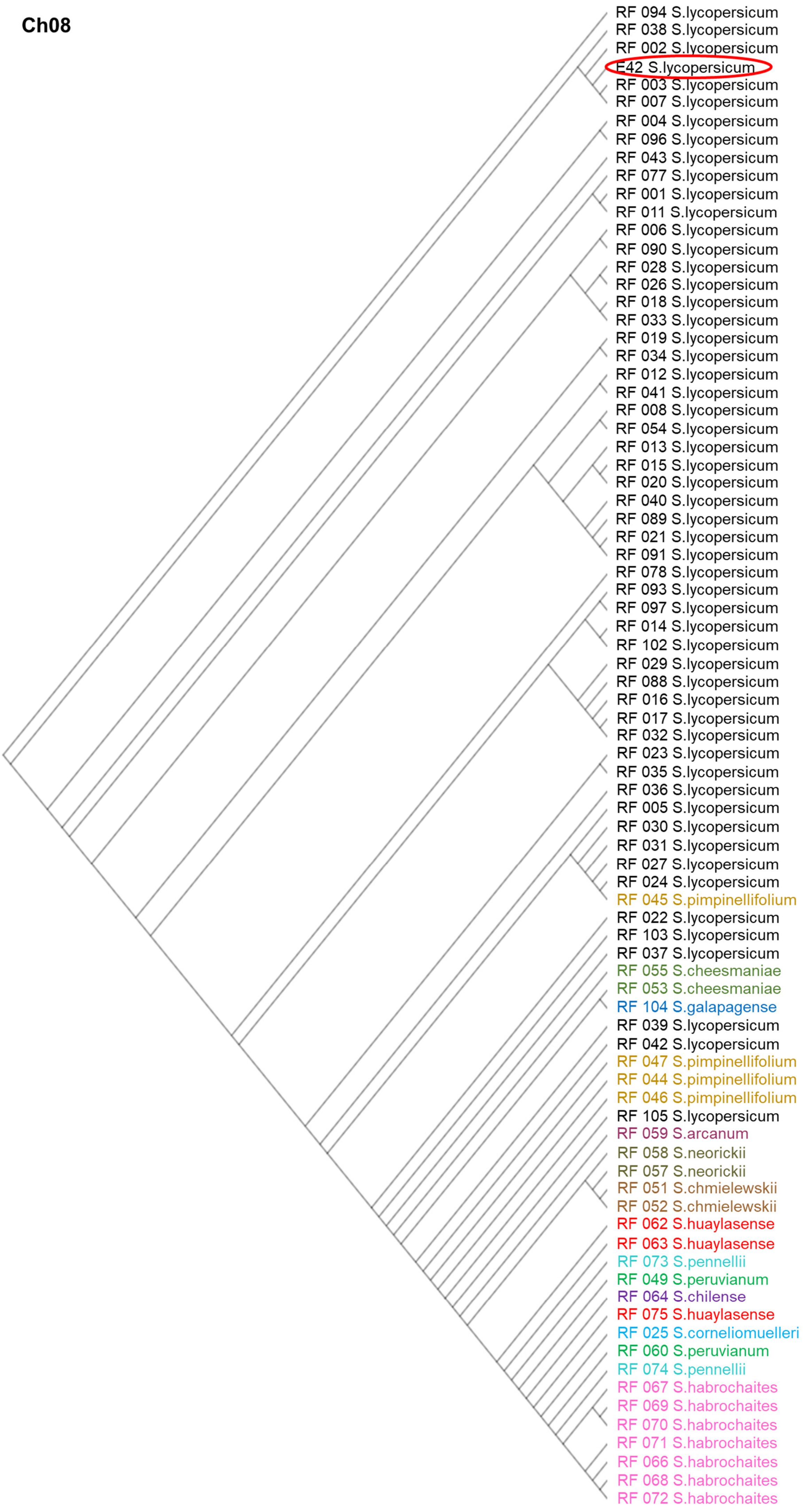
Ch08
RF 094 S.lycopersicum
RF 038 S.lycopersicum
RF 002 S.lycopersicum
E42 S.lycopersicum
RF 003 S.lycopersicum
RF 007 S.lycopersicum
RF 004 S.lycopersicum
RF 096 S.lycopersicum
RF 043 S.lycopersicum
RF 077 S.lycopersicum
RF 001 S.lycopersicum
RF 011 S.lycopersicum
RF 006 S.lycopersicum
RF 090 S.lycopersicum
RF 028 S.lycopersicum
RF 026 S.lycopersicum
RF 018 S.lycopersicum
RF 033 S.lycopersicum
RF 019 S.lycopersicum
RF 034 S.lycopersicum
RF 012 S.lycopersicum
RF 041 S.lycopersicum
RF 008 S.lycopersicum
RF 054 S.lycopersicum
RF 013 S.lycopersicum
RF 015 S.lycopersicum
RF 020 S.lycopersicum
RF 040 S.lycopersicum
RF 089 S.lycopersicum
RF 021 S.lycopersicum
RF 091 S.lycopersicum
RF 078 S.lycopersicum
RF 093 S.lycopersicum
RF 097 S.lycopersicum
RF 014 S.lycopersicum
RF 102 S.lycopersicum
RF 029 S.lycopersicum
RF 088 S.lycopersicum
RF 016 S.lycopersicum
RF 017 S.lycopersicum
RF 032 S.lycopersicum
RF 023 S.lycopersicum
RF 035 S.lycopersicum
RF 036 S.lycopersicum
RF 005 S.lycopersicum
RF 030 S.lycopersicum
RF 031 S.lycopersicum
RF 027 S.lycopersicum
RF 024 S.lycopersicum
RF 045 S.pimpinellifolium
RF 022 S.lycopersicum
RF 103 S.lycopersicum
RF 037 S.lycopersicum
RF 055 S.cheesmaniae
RF 053 S.cheesmaniae
RF 104 S.galapagense
RF 039 S.lycopersicum
RF 042 S.lycopersicum
RF 047 S.pimpinellifolium
RF 044 S.pimpinellifolium
RF 046 S.pimpinellifolium
RF 105 S.lycopersicum
RF 059 S.arcanum
RF 058 S.neorickii
RF 057 S.neorickii
RF 051 S.chmielewskii
RF 052 S.chmielewskii
RF 062 S.huaylasense
RF 063 S.huaylasense
RF 073 S.pennellii
RF 049 S.peruvianum
RF 064 S.chilense
RF 075 S.huaylasense
RF 025 S.corneliomuelleri
RF 060 S.peruvianum
RF 074 S.pennellii
RF 067 S.habrochaites
RF 069 S.habrochaites
RF 070 S.habrochaites
RF 071 S.habrochaites
RF 066 S.habrochaites
RF 068 S.habrochaites
RF 072 S.habrochaites

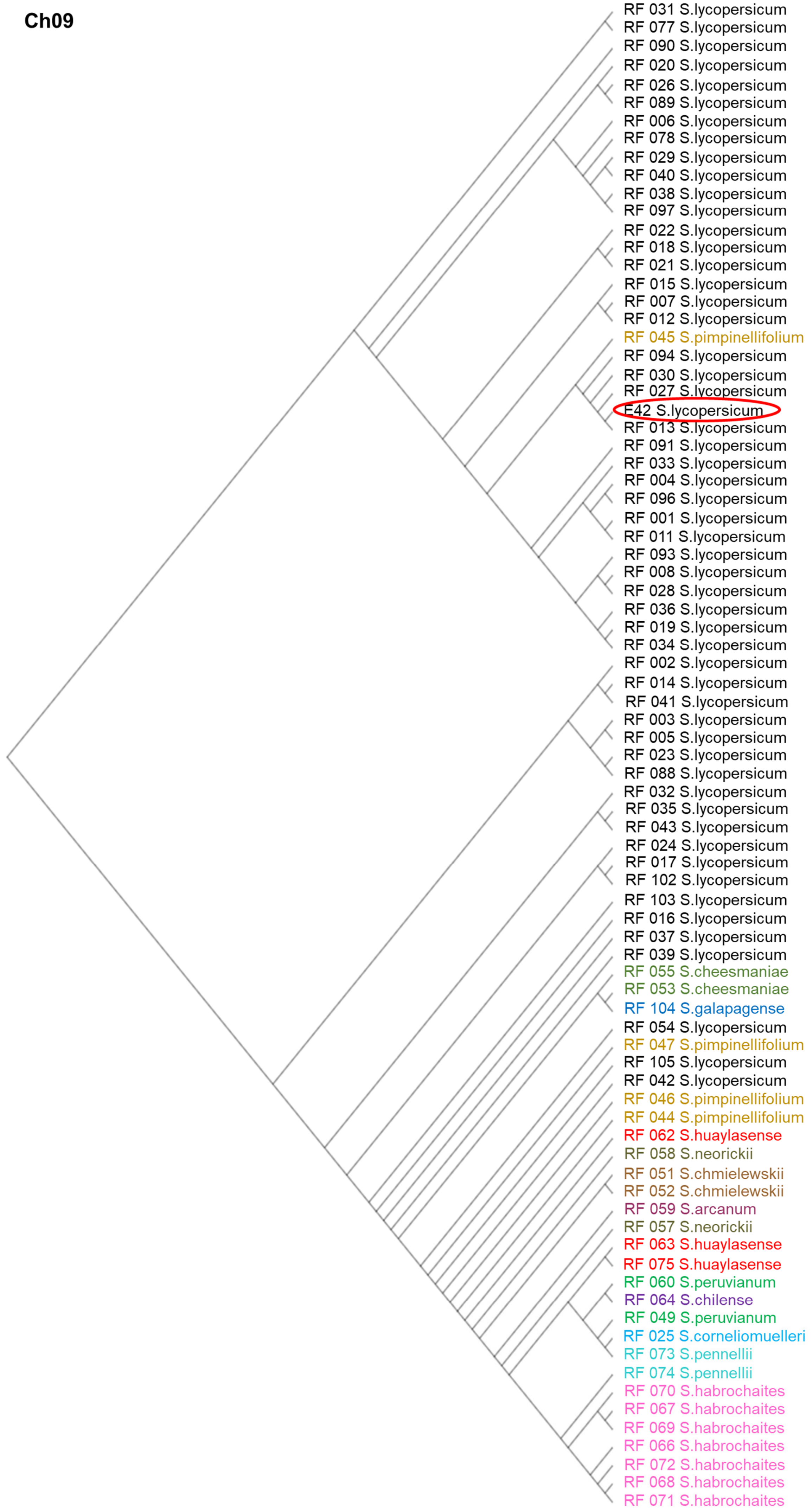
Ch09
RF 031 S.lycopersicum
RF 077 S.lycopersicum
RF 090 S.lycopersicum
RF 020 S.lycopersicum
RF 026 S.lycopersicum
RF 089 S.lycopersicum
RF 006 S.lycopersicum
RF 078 S.lycopersicum
RF 029 S.lycopersicum
RF 040 S.lycopersicum
RF 038 S.lycopersicum
RF 097 S.lycopersicum
RF 022 S.lycopersicum
RF 018 S.lycopersicum
RF 021 S.lycopersicum
RF 015 S.lycopersicum
RF 007 S.lycopersicum
RF 012 S.lycopersicum
RF 045 S.pimpinellifolium
RF 094 S.lycopersicum
RF 030 S.lycopersicum
RF 027 S.lycopersicum
E42 S.lycopersicum
RF 013 S.lycopersicum
RF 091 S.lycopersicum
RF 033 S.lycopersicum
RF 004 S.lycopersicum
RF 096 S.lycopersicum
RF 001 S.lycopersicum
RF 011 S.lycopersicum
RF 093 S.lycopersicum
RF 008 S.lycopersicum
RF 028 S.lycopersicum
RF 036 S.lycopersicum
RF 019 S.lycopersicum
RF 034 S.lycopersicum
RF 002 S.lycopersicum
RF 014 S.lycopersicum
RF 041 S.lycopersicum
RF 003 S.lycopersicum
RF 005 S.lycopersicum
RF 023 S.lycopersicum
RF 088 S.lycopersicum
RF 032 S.lycopersicum
RF 035 S.lycopersicum
RF 043 S.lycopersicum
RF 024 S.lycopersicum
RF 017 S.lycopersicum
RF 102 S.lycopersicum
RF 103 S.lycopersicum
RF 016 S.lycopersicum
RF 037 S.lycopersicum
RF 039 S.lycopersicum
RF 055 S.cheesmaniae
RF 053 S.cheesmaniae
RF 104 S.galapagense
RF 054 S.lycopersicum
RF 047 S.pimpinellifolium
RF 105 S.lycopersicum
RF 042 S.lycopersicum
RF 046 S.pimpinellifolium
RF 044 S.pimpinellifolium
RF 062 S.huaylasense
RF 058 S.neorickii
RF 051 S.chmielewskii
RF 052 S.chmielewskii
RF 059 S.arcanum
RF 057 S.neorickii
RF 063 S.huaylasense
RF 075 S.huaylasense
RF 060 S.peruvianum
RF 064 S.chilense
RF 049 S.peruvianum
RF 025 S.corneliomuelleri
RF 073 S.pennellii
RF 074 S.pennellii
RF 070 S.habrochaites
RF 067 S.habrochaites
RF 069 S.habrochaites
RF 066 S.habrochaites
RF 072 S.habrochaites
RF 068 S.habrochaites
RF 071 S.habrochaites

Ch10

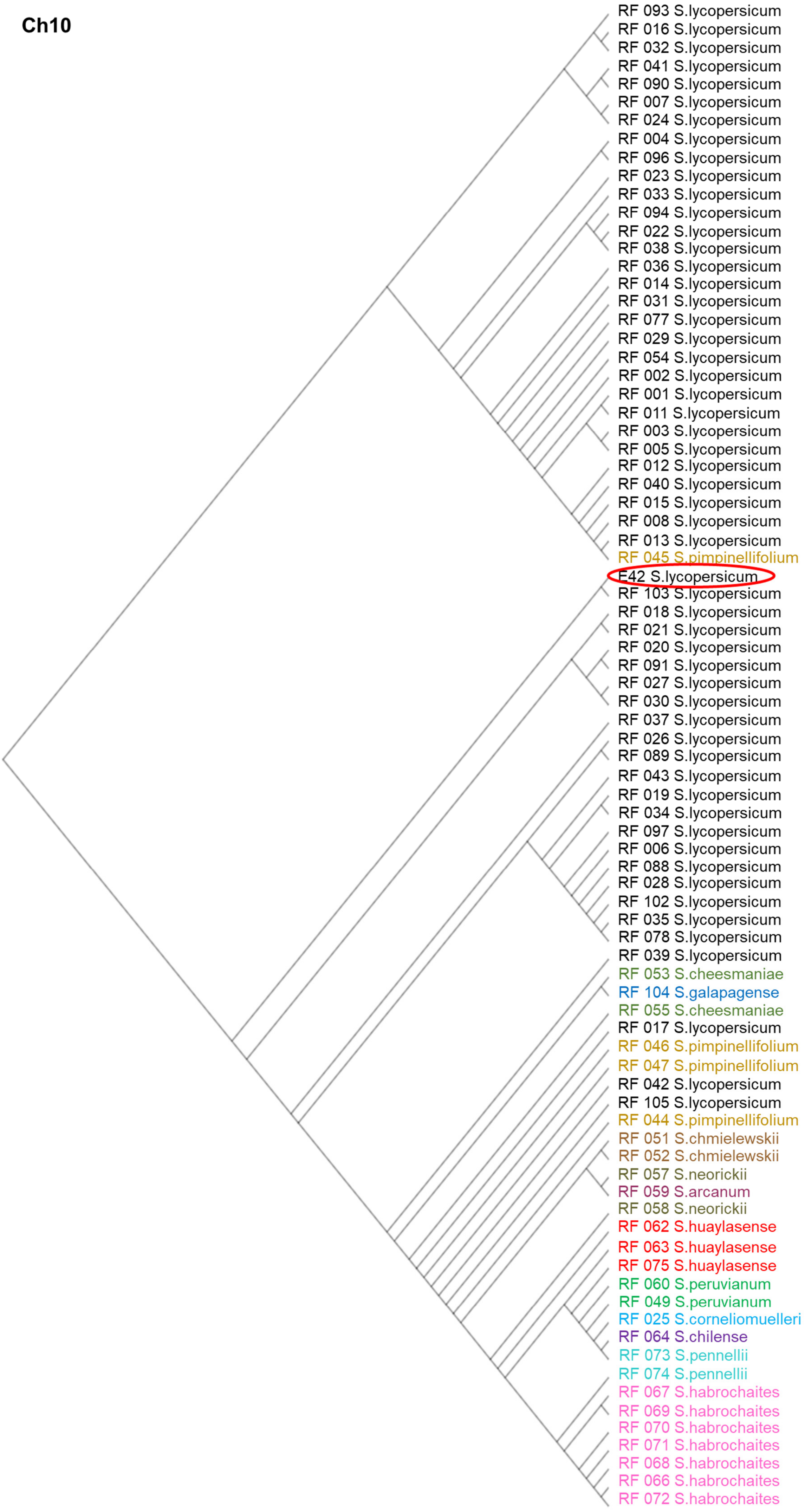
RF 093 S.lycopersicum
RF 016 S.lycopersicum
RF 032 S.lycopersicum
RF 041 S.lycopersicum
RF 090 S.lycopersicum
RF 007 S.lycopersicum
RF 024 S.lycopersicum
RF 004 S.lycopersicum
RF 096 S.lycopersicum
RF 023 S.lycopersicum
RF 033 S.lycopersicum
RF 094 S.lycopersicum
RF 022 S.lycopersicum
RF 038 S.lycopersicum
RF 036 S.lycopersicum
RF 014 S.lycopersicum
RF 031 S.lycopersicum
RF 077 S.lycopersicum
RF 029 S.lycopersicum
RF 054 S.lycopersicum
RF 002 S.lycopersicum
RF 001 S.lycopersicum
RF 011 S.lycopersicum
RF 003 S.lycopersicum
RF 005 S.lycopersicum
RF 012 S.lycopersicum
RF 040 S.lycopersicum
RF 015 S.lycopersicum
RF 008 S.lycopersicum
RF 013 S.lycopersicum
RF 045 S.pimpinellifolium
F42 S.lycopersicum
RF 103 S.lycopersicum
RF 018 S.lycopersicum
RF 021 S.lycopersicum
RF 020 S.lycopersicum
RF 091 S.lycopersicum
RF 027 S.lycopersicum
RF 030 S.lycopersicum
RF 037 S.lycopersicum
RF 026 S.lycopersicum
RF 089 S.lycopersicum
RF 043 S.lycopersicum
RF 019 S.lycopersicum
RF 034 S.lycopersicum
RF 097 S.lycopersicum
RF 006 S.lycopersicum
RF 088 S.lycopersicum
RF 028 S.lycopersicum
RF 102 S.lycopersicum
RF 035 S.lycopersicum
RF 078 S.lycopersicum
RF 039 S.lycopersicum
RF 053 S.cheesmaniae
RF 104 S.galapagense
RF 055 S.cheesmaniae
RF 017 S.lycopersicum
RF 046 S.pimpinellifolium
RF 047 S.pimpinellifolium
RF 042 S.lycopersicum
RF 105 S.lycopersicum
RF 044 S.pimpinellifolium
RF 051 S.chmielewskii
RF 052 S.chmielewskii
RF 057 S.neorickii
RF 059 S.arcanum
RF 058 S.neorickii
RF 062 S.huaylasense
RF 063 S.huaylasense
RF 075 S.huaylasense
RF 060 S.peruvianum
RF 049 S.peruvianum
RF 025 S.corneliomuelleri
RF 064 S.chilense
RF 073 S.pennellii
RF 074 S.pennellii
RF 067 S.habrochaites
RF 069 S.habrochaites
RF 070 S.habrochaites
RF 071 S.habrochaites
RF 068 S.habrochaites
RF 066 S.habrochaites
RF 072 S.habrochaites

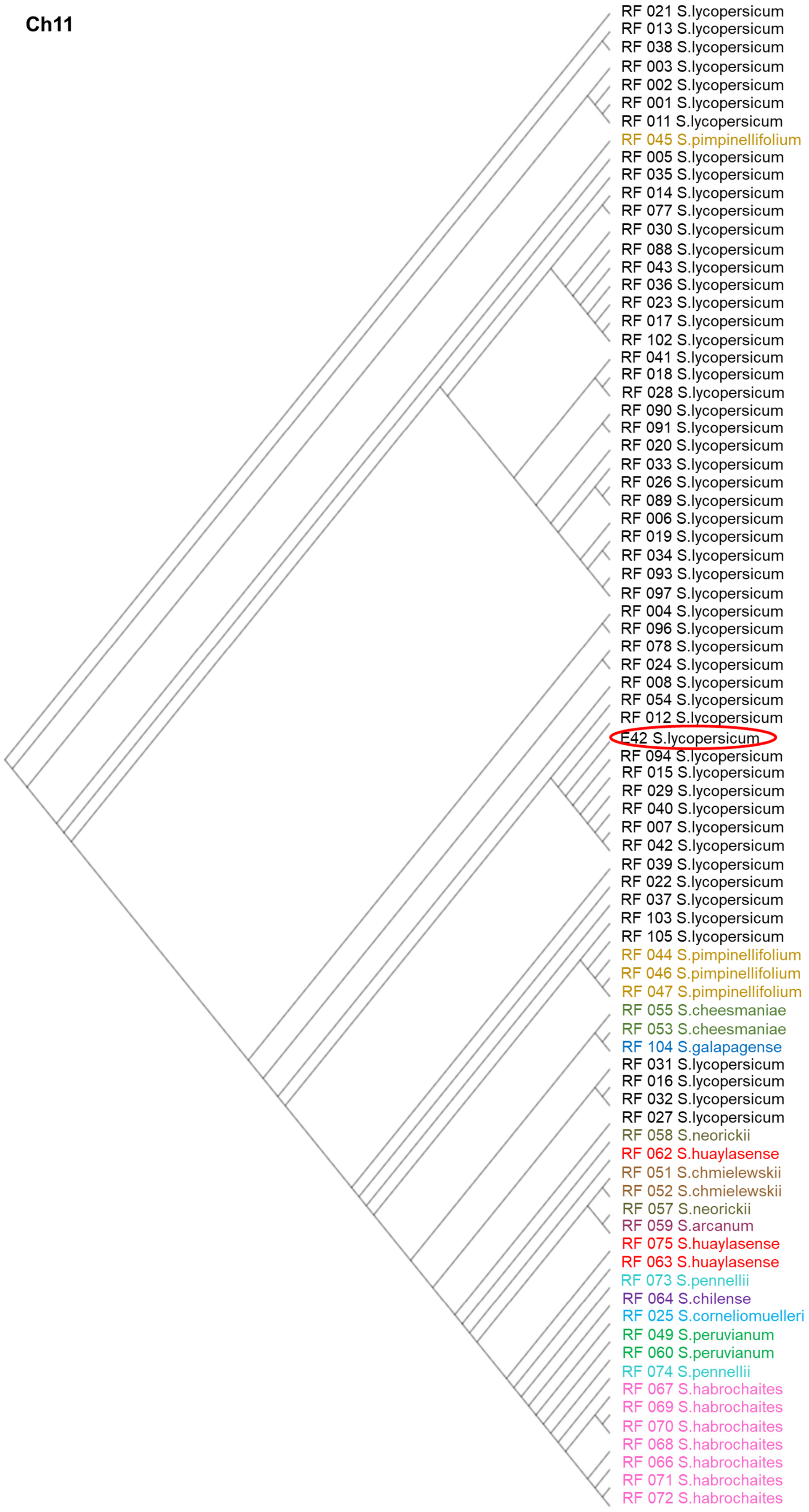
Ch11
RF 021 S.lycopersicum
RF 013 S.lycopersicum
RF 038 S.lycopersicum
RF 003 S.lycopersicum
RF 002 S.lycopersicum
RF 001 S.lycopersicum
RF 011 S.lycopersicum
RF 045 S.pimpinellifolium
RF 005 S.lycopersicum
RF 035 S.lycopersicum
RF 014 S.lycopersicum
RF 077 S.lycopersicum
RF 030 S.lycopersicum
RF 088 S.lycopersicum
RF 043 S.lycopersicum
RF 036 S.lycopersicum
RF 023 S.lycopersicum
RF 017 S.lycopersicum
RF 102 S.lycopersicum
RF 041 S.lycopersicum
RF 018 S.lycopersicum
RF 028 S.lycopersicum
RF 090 S.lycopersicum
RF 091 S.lycopersicum
RF 020 S.lycopersicum
RF 033 S.lycopersicum
RF 026 S.lycopersicum
RF 089 S.lycopersicum
RF 006 S.lycopersicum
RF 019 S.lycopersicum
RF 034 S.lycopersicum
RF 093 S.lycopersicum
RF 097 S.lycopersicum
RF 004 S.lycopersicum
RF 096 S.lycopersicum
RF 078 S.lycopersicum
RF 024 S.lycopersicum
RF 008 S.lycopersicum
RF 054 S.lycopersicum
RF 012 S.lycopersicum
F42 S.lycopersicum
RF 094 S.lycopersicum
RF 015 S.lycopersicum
RF 029 S.lycopersicum
RF 040 S.lycopersicum
RF 007 S.lycopersicum
RF 042 S.lycopersicum
RF 039 S.lycopersicum
RF 022 S.lycopersicum
RF 037 S.lycopersicum
RF 103 S.lycopersicum
RF 105 S.lycopersicum
RF 044 S.pimpinellifolium
RF 046 S.pimpinellifolium
RF 047 S.pimpinellifolium
RF 055 S.cheesmaniae
RF 053 S.cheesmaniae
RF 104 S.galapagense
RF 031 S.lycopersicum
RF 016 S.lycopersicum
RF 032 S.lycopersicum
RF 027 S.lycopersicum
RF 058 S.neorickii
RF 062 S.huaylasense
RF 051 S.chmielewskii
RF 052 S.chmielewskii
RF 057 S.neorickii
RF 059 S.arcanum
RF 075 S.huaylasense
RF 063 S.huaylasense
RF 073 S.pennellii
RF 064 S.chilense
RF 025 S.corneliomuelleri
RF 049 S.peruvianum
RF 060 S.peruvianum
RF 074 S.pennellii
RF 067 S.habrochaites
RF 069 S.habrochaites
RF 070 S.habrochaites
RF 068 S.habrochaites
RF 066 S.habrochaites
RF 071 S.habrochaites
RF 072 S.habrochaites

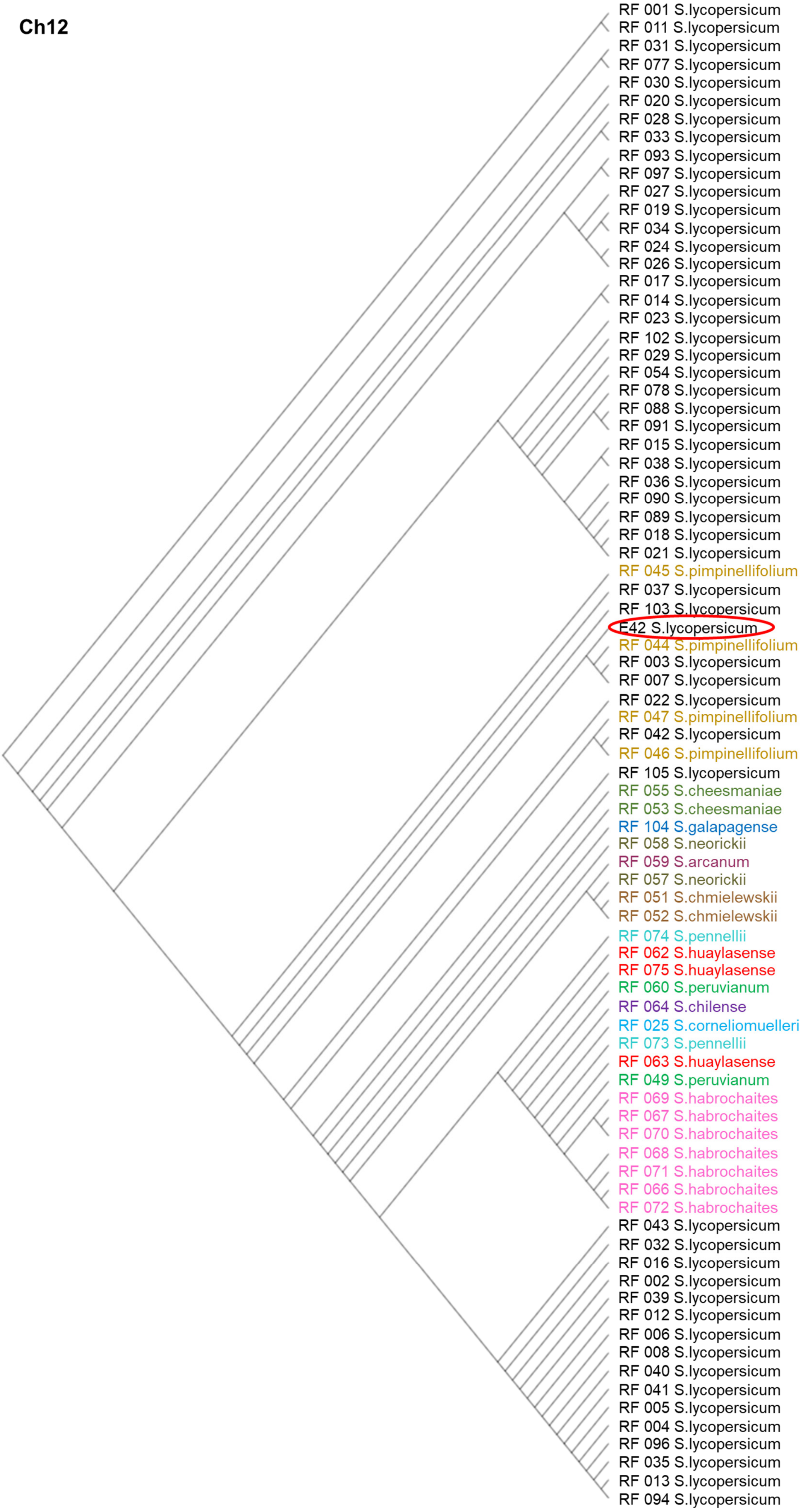

Supplementary Figure S1 – Phylogenetic trees of the 12 tomato chromosomes involving E42 and 82 accessions belonging to 13 tomato species.
