# Supplementary material for: Genomic Insights into the Origin of a Thermotolerant Tomato Line and Identification of Candidate Genes for Heat Stress
Source: Genes (Basel). 2023 Feb 21;14(3):535. doi: 10.3390/genes14030535 (PMC10048601; doi:10.3390/genes14030535)
Supplement: Supplementary file 1 [file genes-14-00535-s001.zip › Supplementary Figure S2.pdf]

Ch01

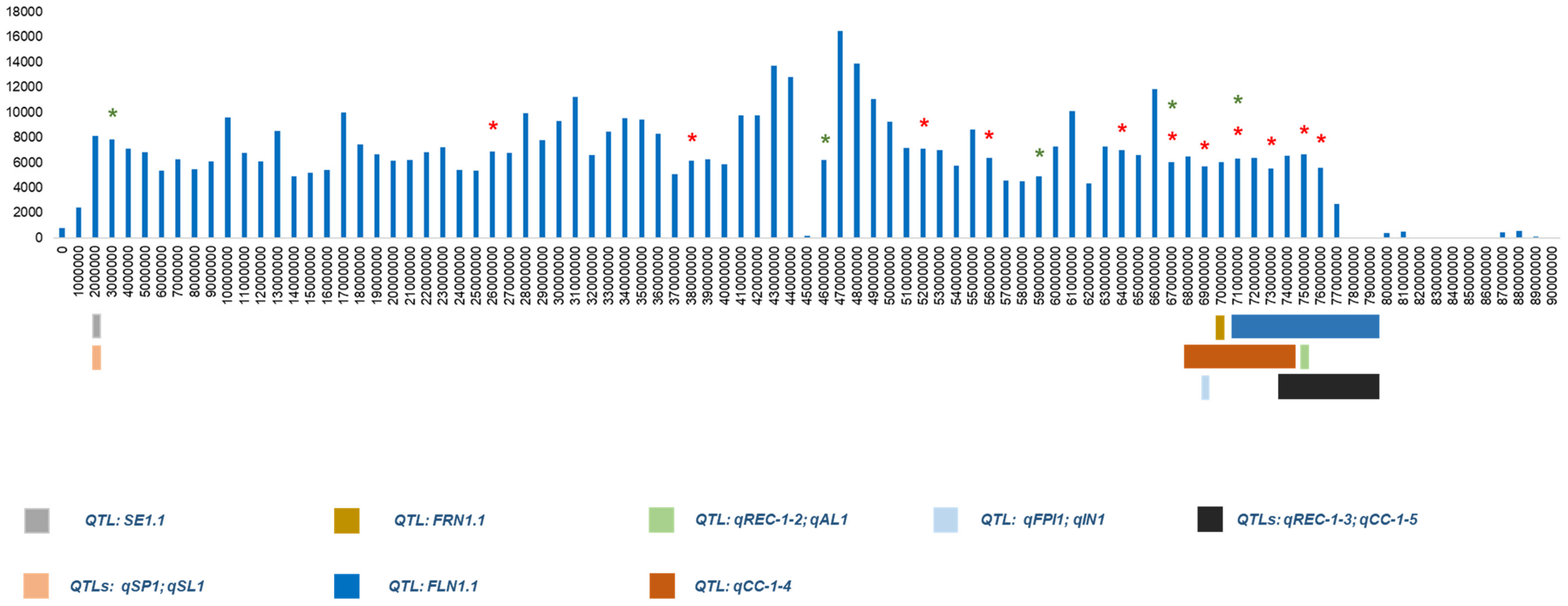

18000
16000
14000
12000
10000
8000
6000
4000
2000
0
QTL: SE1.1
QTL: FRN1.1
QTL: qREC-1-2; qAL1
QTL: qFPI1; qIN1
QTLs: qREC-1-3; qCC-1-5
QTLs: qSP1; qSL1
QTL: FLN1.1
QTL: qCC-1-4

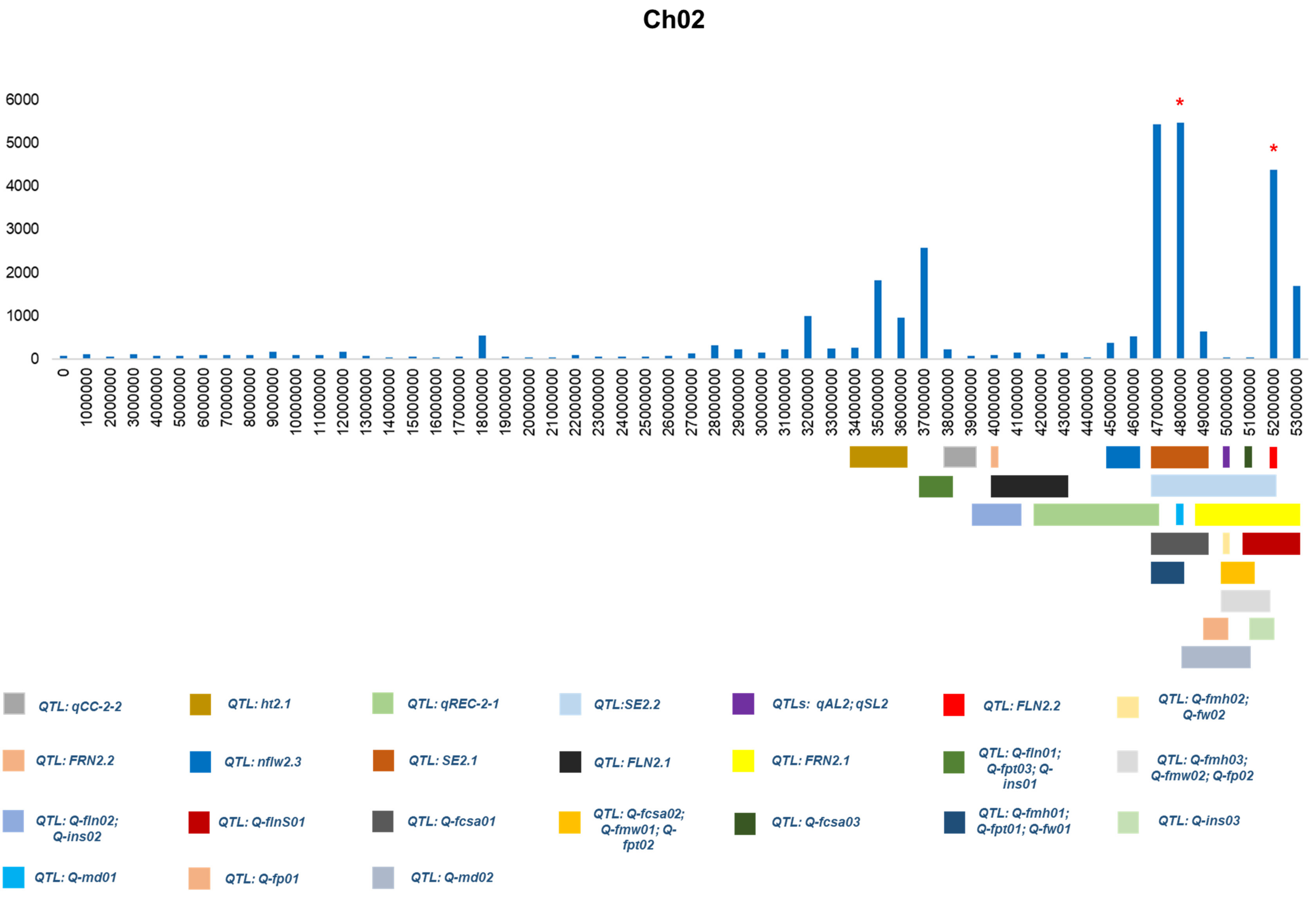
Ch02
6000
5000
4000
3000
2000
1000
0
*
*
QTL: qCC-2-2
QTL: ht2.1
QTL: qREC-2-1
QTL:SE2.2
QTLs: qAL2; qSL2
QTL: FLN2.2
QTL: Q-fmh02; Q-fw02
QTL: FRN2.2
QTL: nflw2.3
QTL: SE2.1
QTL: FLN2.1
QTL: FRN2.1
QTL: Q-fln01; Q-fpt03; Q-ins01
QTL: Q-fmh03; Q-fmw02; Q-fp02
QTL: Q-fln02; Q-ins02
QTL: Q-flnS01
QTL: Q-fcsa01
QTL: Q-fcsa02; Q-fmw01; Q-fpt02
QTL: Q-fcsa03
QTL: Q-fmh01; Q-fpt01; Q-fw01
QTL: Q-ins03
QTL: Q-md01
QTL: Q-fp01
QTL: Q-md02

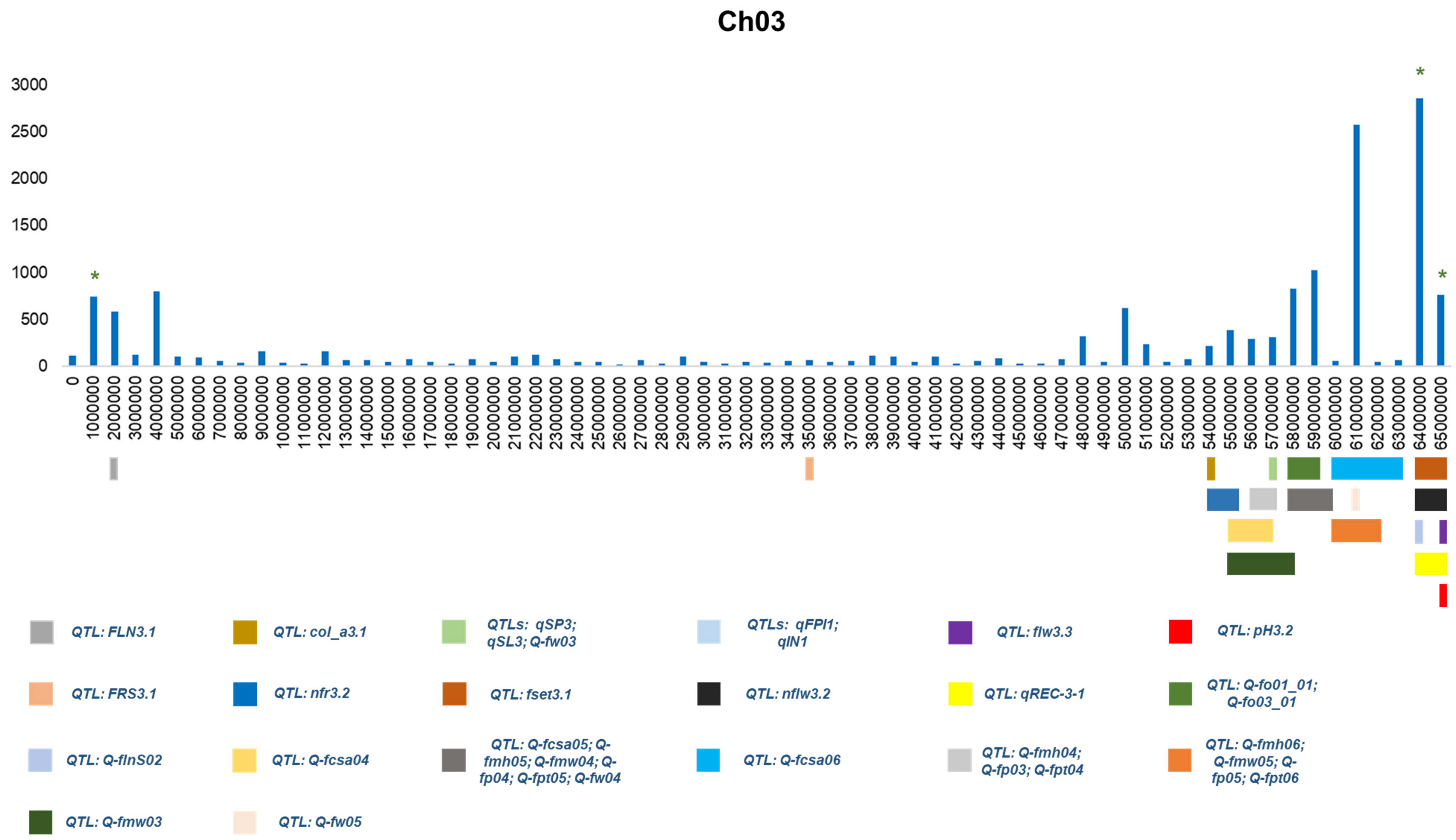

Ch03
3000
2500
2000
1500
1000
500
0
0
1000000
2000000
3000000
4000000
5000000
6000000
7000000
8000000
9000000
10000000
11000000
12000000
13000000
14000000
15000000
16000000
17000000
18000000
19000000
20000000
21000000
22000000
23000000
24000000
25000000
26000000
27000000
28000000
29000000
30000000
31000000
32000000
33000000
34000000
35000000
36000000
37000000
38000000
39000000
40000000
41000000
42000000
43000000
44000000
45000000
46000000
47000000
48000000
49000000
50000000
51000000
52000000
53000000
54000000
55000000
56000000
57000000
58000000
59000000
60000000
61000000
62000000
63000000
64000000
65000000
QTL: FLN3.1
QTL: col_a3.1
QTLs: qSP3; qSL3; Q-fw03
QTLs: qFPI1; qIN1
QTL: flw3.3
QTL: pH3.2
QTL: FRS3.1
QTL: nfr3.2
QTL: fset3.1
QTL: nflw3.2
QTL: qREC-3-1
QTL: Q-fo01_01; Q-fo03_01
QTL: Q-flnS02
QTL: Q-fcsa04
QTL: Q-fcsa05; Q-fmh05; Q-fmw04; Q-fp04; Q-fpt05; Q-fw04
QTL: Q-fcsa06
QTL: Q-fmh04; Q-fp03; Q-fpt04
QTL: Q-fmh06; Q-fmw05; Q-fp05; Q-fpt06
QTL: Q-fmw03
QTL: Q-fw05

Ch04

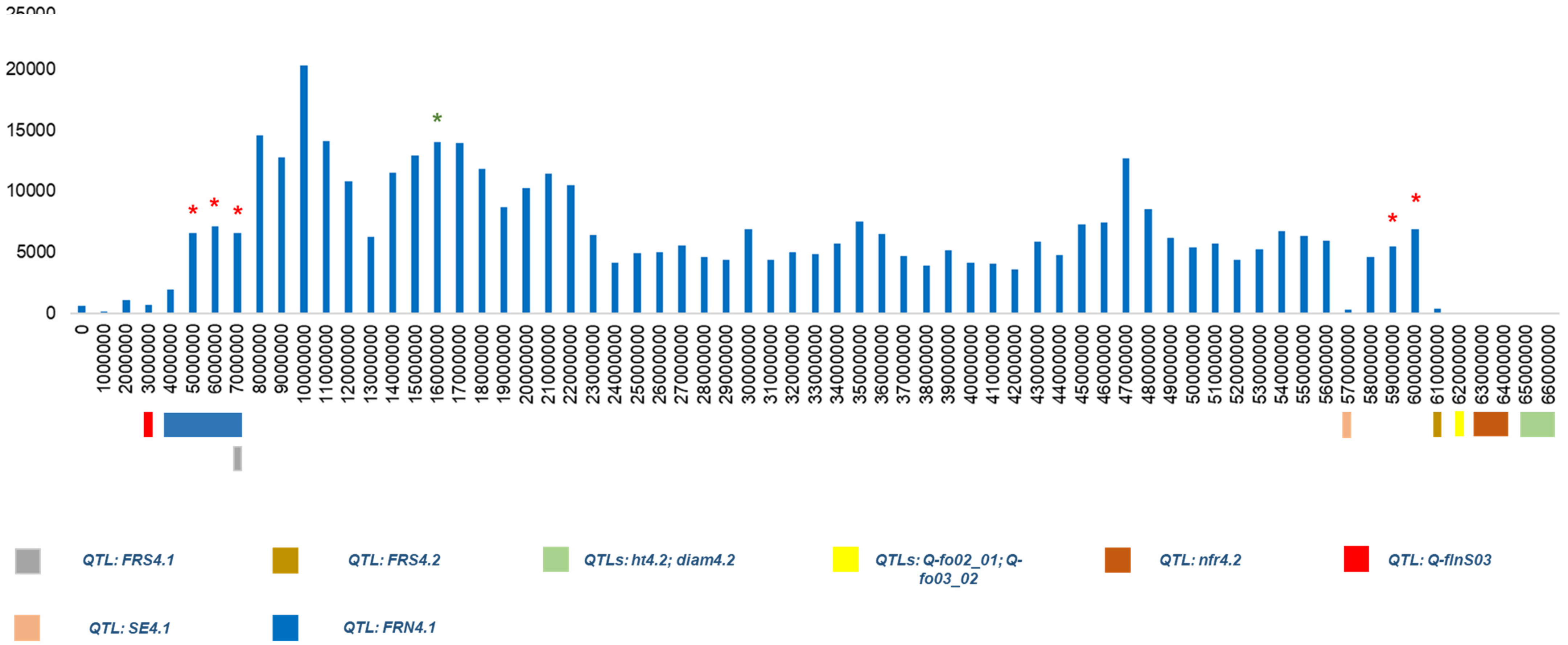

25000
20000
15000
10000
5000
0
0
1000000
2000000
3000000
4000000
5000000
6000000
7000000
8000000
9000000
10000000
11000000
12000000
13000000
14000000
15000000
16000000
17000000
18000000
19000000
20000000
21000000
22000000
23000000
24000000
25000000
26000000
27000000
28000000
29000000
30000000
31000000
32000000
33000000
34000000
35000000
36000000
37000000
38000000
39000000
40000000
41000000
42000000
43000000
44000000
45000000
46000000
47000000
48000000
49000000
50000000
51000000
52000000
53000000
54000000
55000000
56000000
57000000
58000000
59000000
60000000
61000000
62000000
63000000
64000000
65000000
66000000
QTL: FRS4.1
QTL: FRS4.2
QTLs: ht4.2; diam4.2
QTLs: Q-fo02_01; Q-fo03_02
QTL: nfr4.2
QTL: Q-flnS03
QTL: SE4.1
QTL: FRN4.1

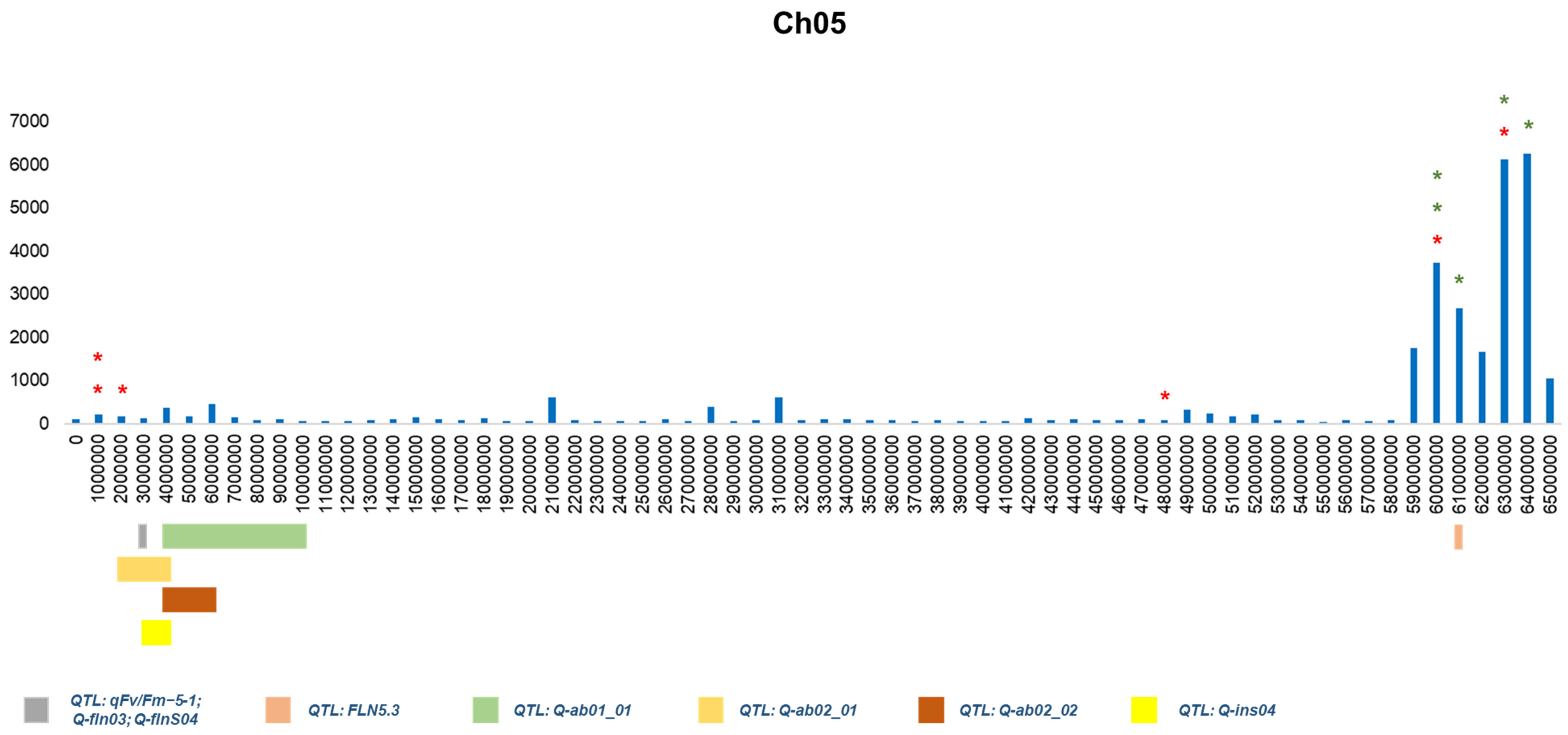
Ch05
7000
6000
5000
4000
3000
2000
1000
0
0
1000000
2000000
3000000
4000000
5000000
6000000
7000000
8000000
9000000
10000000
11000000
12000000
13000000
14000000
15000000
16000000
17000000
18000000
19000000
20000000
21000000
22000000
23000000
24000000
25000000
26000000
27000000
28000000
29000000
30000000
31000000
32000000
33000000
34000000
35000000
36000000
37000000
38000000
39000000
40000000
41000000
42000000
43000000
44000000
45000000
46000000
47000000
48000000
49000000
50000000
51000000
52000000
53000000
54000000
55000000
56000000
57000000
58000000
59000000
60000000
61000000
62000000
63000000
64000000
65000000
QTL: qFv/Fm−5-1; Q-fln03; Q-flnS04
QTL: FLN5.3
QTL: Q-ab01_01
QTL: Q-ab02_01
QTL: Q-ab02_02
QTL: Q-ins04

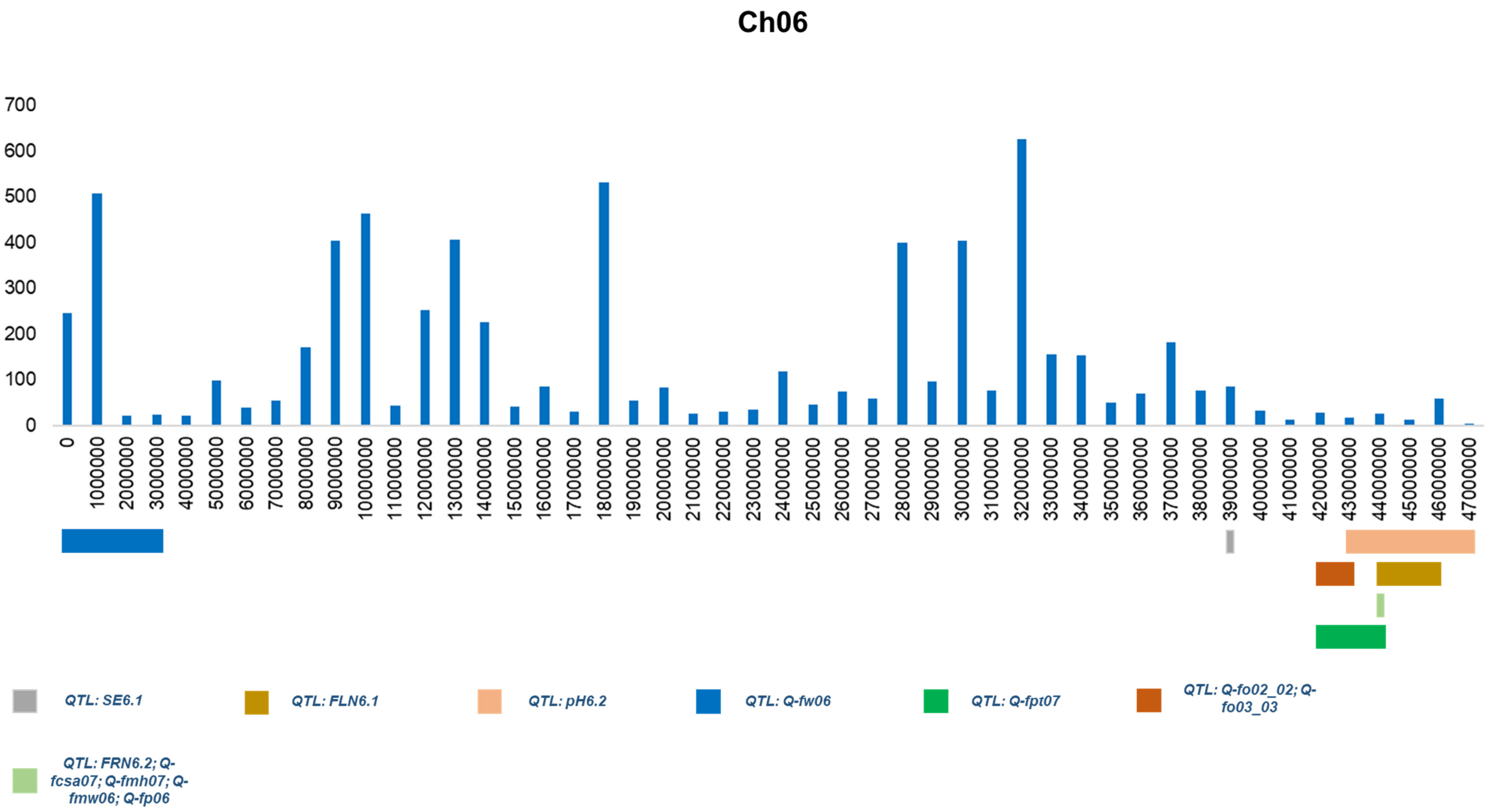
Ch06
700
600
500
400
300
200
100
0
0
1000000
2000000
3000000
4000000
5000000
6000000
7000000
8000000
9000000
10000000
11000000
12000000
13000000
14000000
15000000
16000000
17000000
18000000
19000000
20000000
21000000
22000000
23000000
24000000
25000000
26000000
27000000
28000000
29000000
30000000
31000000
32000000
33000000
34000000
35000000
36000000
37000000
38000000
39000000
40000000
41000000
42000000
43000000
44000000
45000000
46000000
47000000
QTL: SE6.1
QTL: FLN6.1
QTL: pH6.2
QTL: Q-fw06
QTL: Q-fpt07
QTL: Q-fo02_02; Q-fo03_03
QTL: FRN6.2; Q-fcsa07; Q-fmh07; Q-fmw06; Q-fp06

Ch07

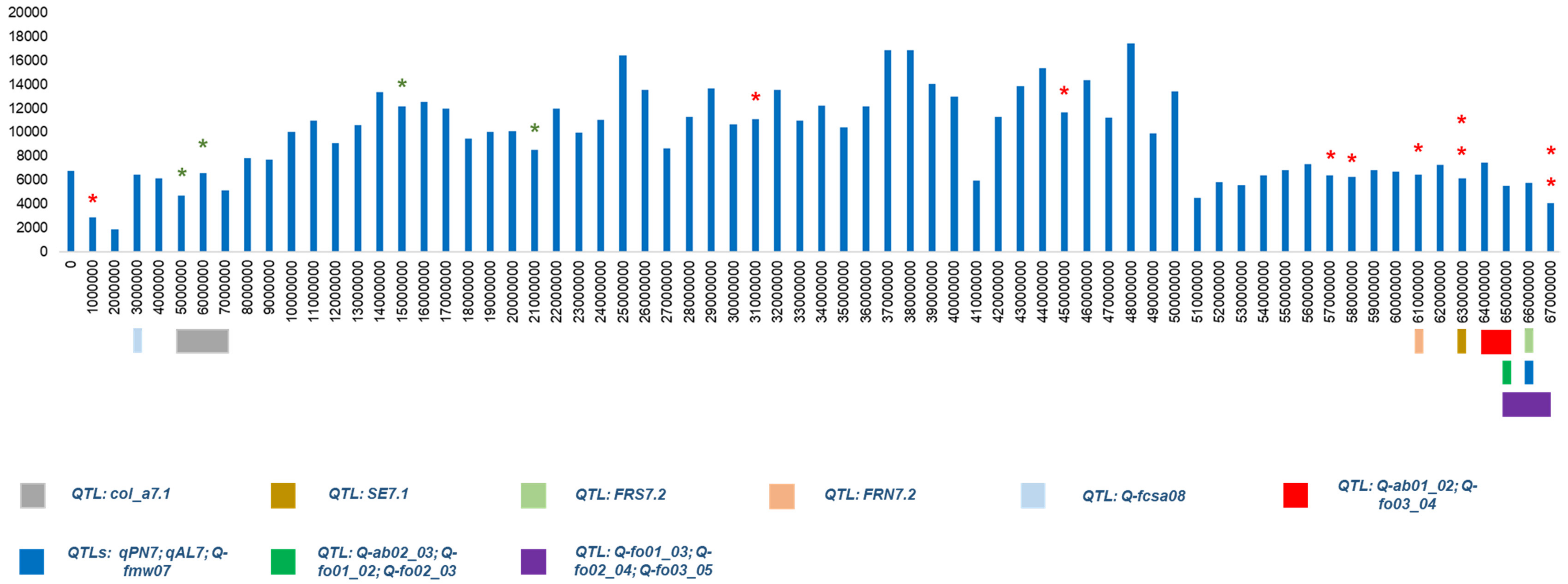

20000
18000
16000
14000
12000
10000
8000
6000
4000
2000
0
0
1000000
2000000
3000000
4000000
5000000
6000000
7000000
8000000
9000000
10000000
11000000
12000000
13000000
14000000
15000000
16000000
17000000
18000000
19000000
20000000
21000000
22000000
23000000
24000000
25000000
26000000
27000000
28000000
29000000
30000000
31000000
32000000
33000000
34000000
35000000
36000000
37000000
38000000
39000000
40000000
41000000
42000000
43000000
44000000
45000000
46000000
47000000
48000000
49000000
50000000
51000000
52000000
53000000
54000000
55000000
56000000
57000000
58000000
59000000
60000000
61000000
62000000
63000000
64000000
65000000
66000000
67000000
QTL: col_a7.1
QTL: SE7.1
QTL: FRS7.2
QTL: FRN7.2
QTL: Q-fcsa08
QTL: Q-ab01_02; Q-fo03_04
QTLs: qPN7; qAL7; Q-fmw07
QTL: Q-ab02_03; Q-fo01_02; Q-fo02_03
QTL: Q-fo01_03; Q-fo02_04; Q-fo03_05

Ch08

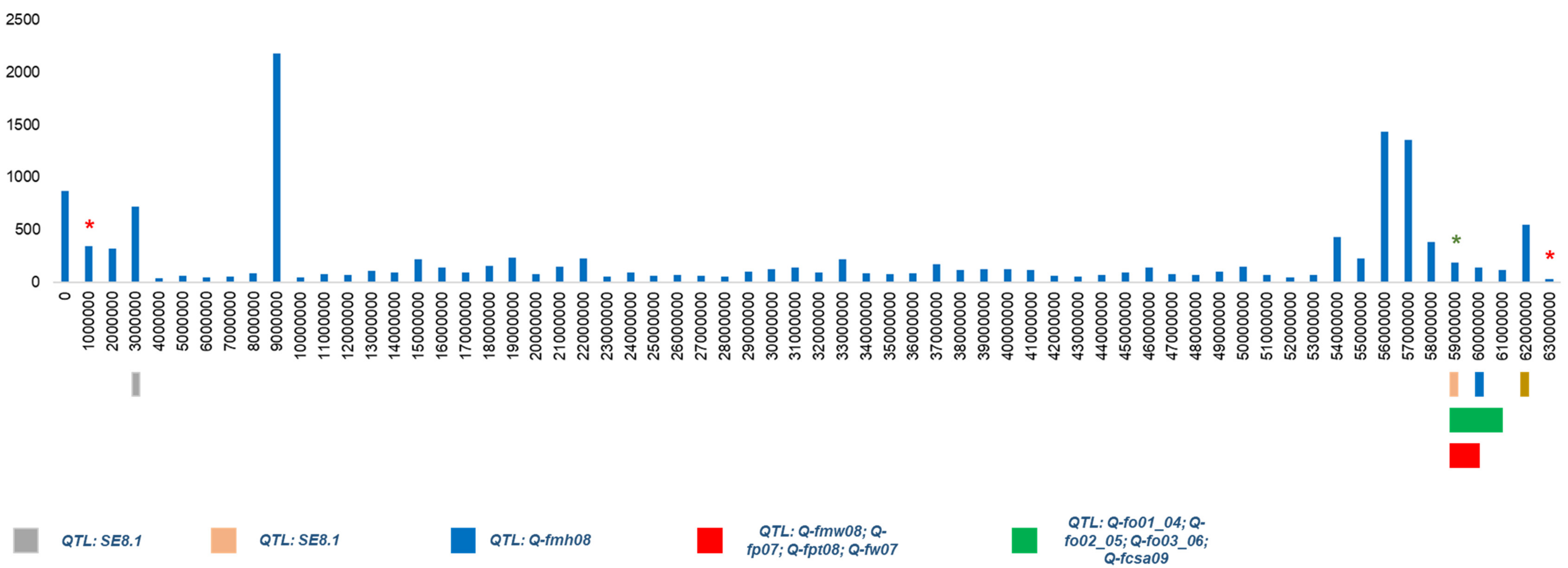

2500
2000
1500
1000
500
0
0
1000000
2000000
3000000
4000000
5000000
6000000
7000000
8000000
9000000
10000000
11000000
12000000
13000000
14000000
15000000
16000000
17000000
18000000
19000000
20000000
21000000
22000000
23000000
24000000
25000000
26000000
27000000
28000000
29000000
30000000
31000000
32000000
33000000
34000000
35000000
36000000
37000000
38000000
39000000
40000000
41000000
42000000
43000000
44000000
45000000
46000000
47000000
48000000
49000000
50000000
51000000
52000000
53000000
54000000
55000000
56000000
57000000
58000000
59000000
60000000
61000000
62000000
63000000
*
*
*
QTL: SE8.1
QTL: SE8.1
QTL: Q-fmh08
QTL: Q-fmw08; Q-fp07; Q-fpt08; Q-fw07
QTL: Q-fo01_04; Q-fo02_05; Q-fo03_06; Q-fcsa09

Ch09

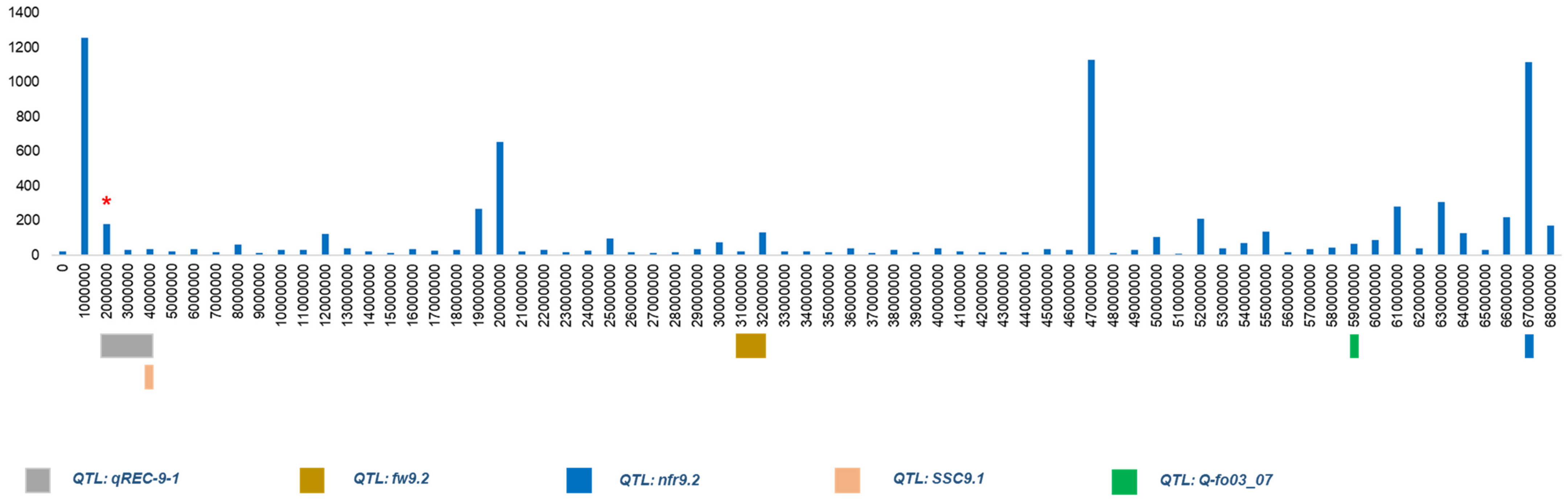

1400
1200
1000
800
600
400
200
0
*
0
1000000
2000000
3000000
4000000
5000000
6000000
7000000
8000000
9000000
10000000
11000000
12000000
13000000
14000000
15000000
16000000
17000000
18000000
19000000
20000000
21000000
22000000
23000000
24000000
25000000
26000000
27000000
28000000
29000000
30000000
31000000
32000000
33000000
34000000
35000000
36000000
37000000
38000000
39000000
40000000
41000000
42000000
43000000
44000000
45000000
46000000
47000000
48000000
49000000
50000000
51000000
52000000
53000000
54000000
55000000
56000000
57000000
58000000
59000000
60000000
61000000
62000000
63000000
64000000
65000000
66000000
67000000
68000000
QTL: qREC-9-1
QTL: fw9.2
QTL: nfr9.2
QTL: SSC9.1
QTL: Q-fo03_07

Ch10

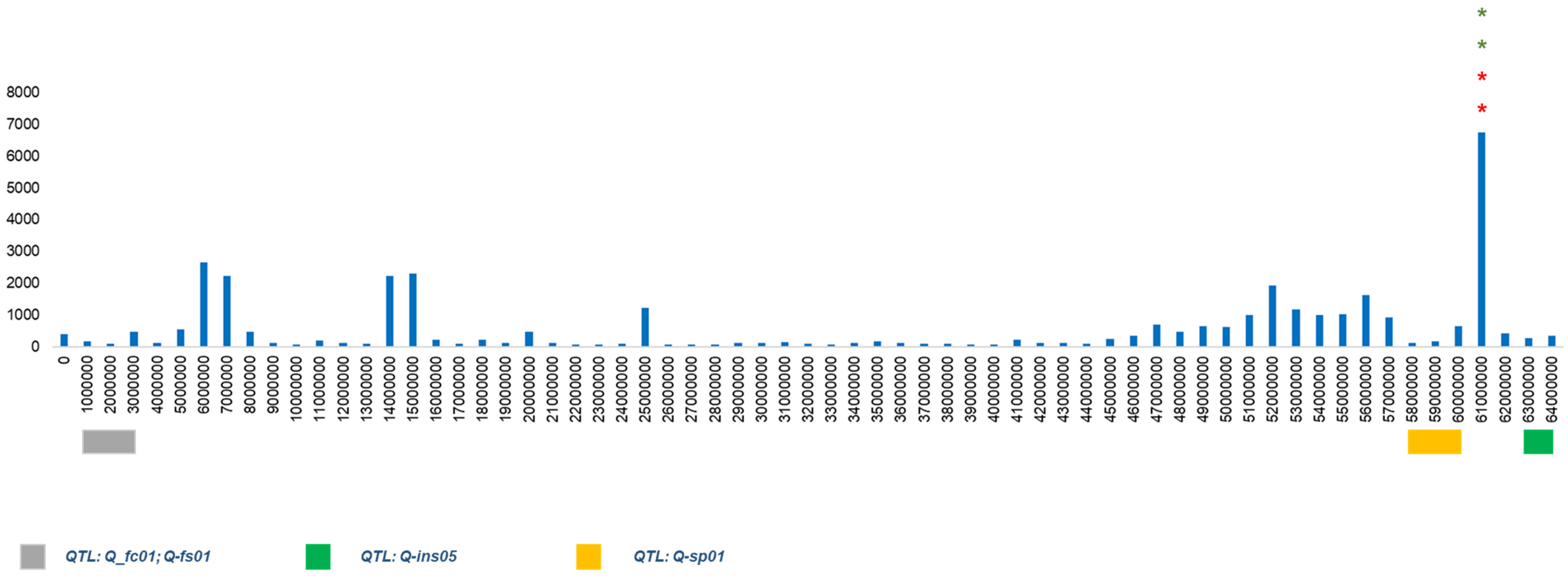

8000
7000
6000
5000
4000
3000
2000
1000
0
QTL: Q_fc01; Q-fs01
QTL: Q-ins05
QTL: Q-sp01

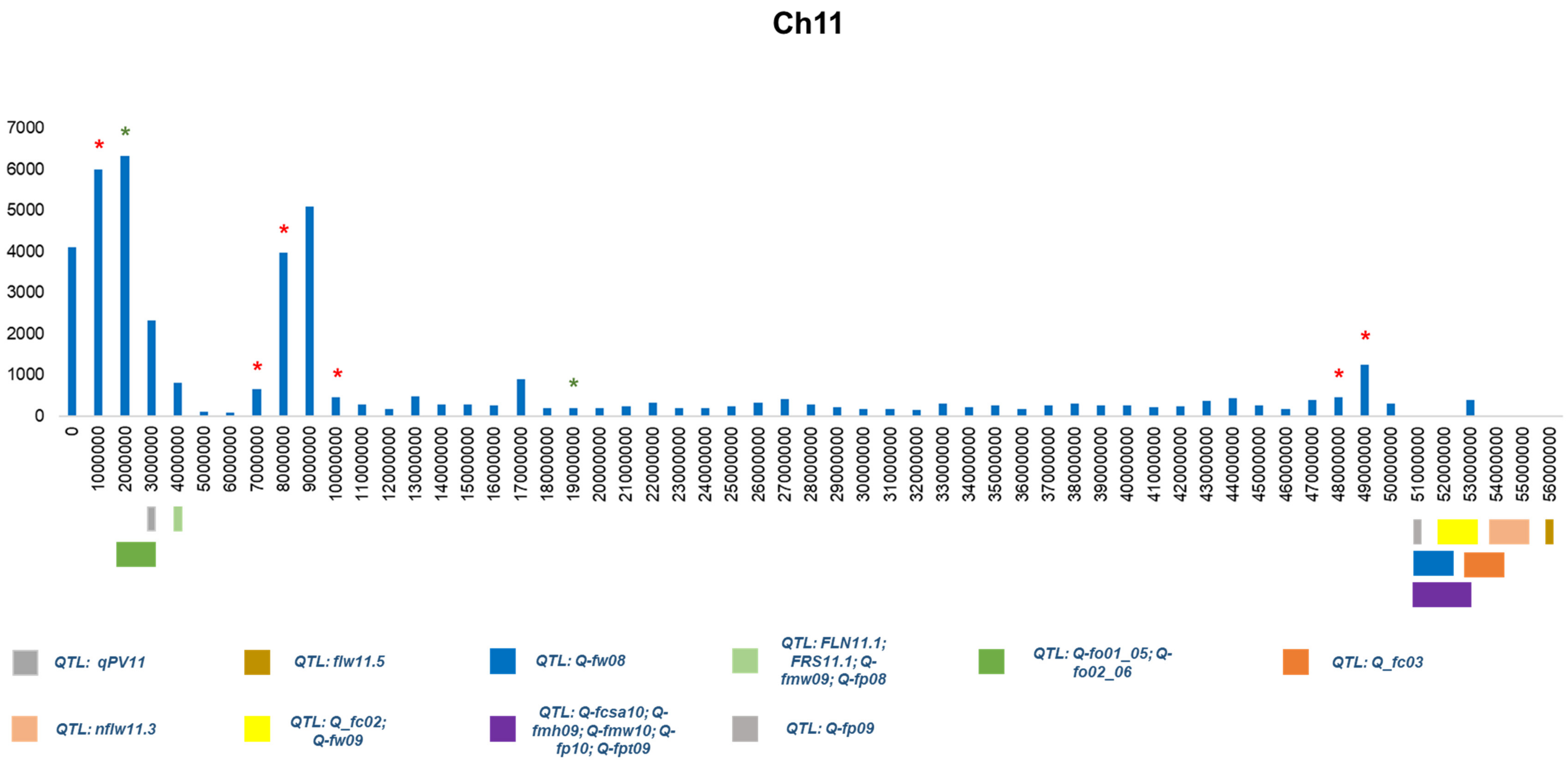
Ch11
7000
6000
5000
4000
3000
2000
1000
0
QTL: qPV11
QTL: flw11.5
QTL: Q-fw08
QTL: FLN11.1; FRS11.1; Q-fmw09; Q-fp08
QTL: Q-fo01_05; Q-fo02_06
QTL: Q_fc03
QTL: nflw11.3
QTL: Q_fc02; Q-fw09
QTL: Q-fcsa10; Q-fmh09; Q-fmw10; Q-fp10; Q-fpt09
QTL: Q-fp09

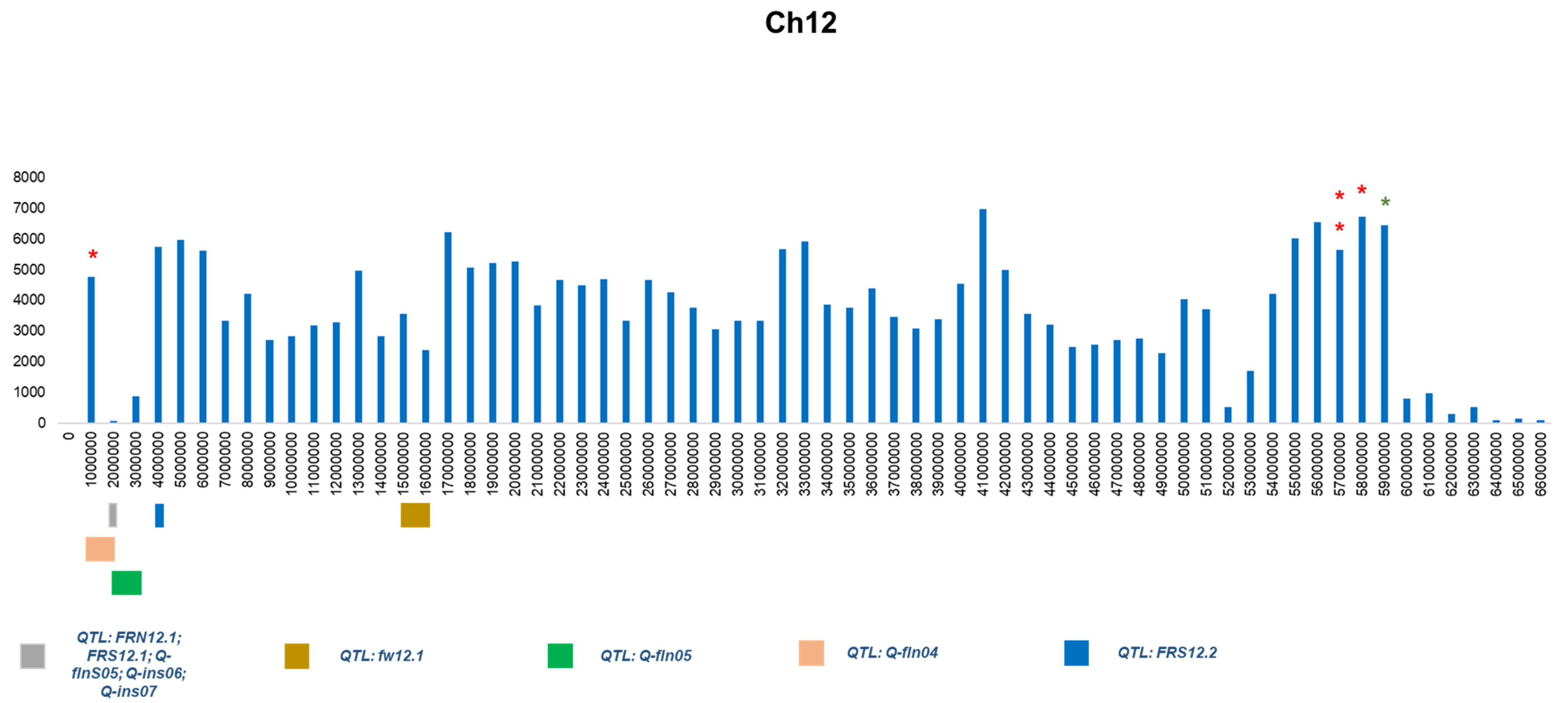

Supplementary Figure S2 – Data regarding the filtered merged VCF file of the 83 tomato accessions with 175,631 total raw variants. The number of variants and common variants of each accession with E42 is reported.
