# Supplementary material for: Genomic Insights into the Origin of a Thermotolerant Tomato Line and Identification of Candidate Genes for Heat Stress
Source: Genes (Basel). 2023 Feb 21;14(3):535. doi: 10.3390/genes14030535 (PMC10048601; doi:10.3390/genes14030535)
Supplement: Supplementary file 1 [file genes-14-00535-s001.zip › Supplementary Text S1.pdf]

Supplementary Text S1 – Multi FASTA alignment of E42 with HEINZ and LA2093 nucleotide sequences of the 35 selected genes. Red bolded characters indicate the HIGH and/or MODERATE variant sites.

Solyc01g009580

```
E42          TGAATAGGCCATCAAAAGCTACCTCCGCCCTCACTCAAAGTGTTGTGTATATAAAGTCCT     60
HEINZ        TGAATACGTCATCAAAAGCTACCTCTGCCATCACTCAAAGTGTTGTGTATATAAAGTCCT     60
LA2093       TGAATACGTCATCAAAAGCTACCTCTGCCATCACTCAAAGTGTTGTGTATATAAAGTCCT     60
             ****** * **************** *** ******************************

E42          CCTTGCAAGTAAAATTGTTAAAACCATCTCACATATATCTCCCCATTGCCTCTATAGCTT     120
HEINZ        CCTTGCAAGTAAAATTGTTAAAACCATCTCACATATATCTCTCCATTGCCTCTATAGCTT     120
LA2093       CCTTGCAAGTAAAATTGTTAAAACCATCTCACATATATCTCTCCATTGCCTCTATAGCTT     120
             ***************************************** ******************

E42          ACACTAAA-AAACACACAATATGTCTTGTAGAGATATTGAACCACTTATTGTTGCAAGAG     179
HEINZ        ACACTAAAAAAACACACAATATGTCTTGTAGGGATATTGAACCACTTATTGTTGCAAGAG     180
LA2093       ACACTAAAAAAACACACAATATGTCTTGTAGGGATATTGAACCACTTATTGTTGCAAGAG     180
             ******** ********************** ****************************

E42          TTATAGGAGAAGTTGTTGATAGTTTCAATCCAAGTGTGAAAATGAATGTGATATATAATG     239
HEINZ        TTATAGGAGAAGTTGTTGATAGTTTCAATCCAAGTGTGAAAATGAATGTGATATATAATG     240
LA2093       TTATAGGAGAAGTTGTTGATAGTTTCAATCCAAGTGTGAAAATGAATGTGATATATAATG     240
             ************************************************************

E42          GAACCAAACAAGTCTTTAATGGTCATGAACTCATGCCTCTTGTCATTGCTTCTAAGCCTC     299
HEINZ        GAACCAAACAAGTCTTTAATGGTCATGAACTCATGCCTCTTGTCATTGCTTCTAAGCCTC     300
LA2093       GAACCAAACAAGTCTTTAATGGTCATGAACTCATGCCTCTTGTCATTGCTTCTAAGCCTC     300
             ************************************************************

E42          GTGTCGAGATTGGTGGCGAAGACATGAGATCTGCTTACACACTCGTACGTTATATTTAAT     359
HEINZ        GTGTCGAGATTGGTGGCGAAGACATGAGATCTGCTTACACACTCGTACGTTATATTTAAT     360
LA2093       GTGTCGAGATTGGTGGCGAAGACATGAGATCTGCTTACACACTCGTACGTTATATTTAAT     360
             ************************************************************

E42          TTATGTGATATTTTTCGTTTCTTGATATTCAAATTAAACTATCTCTGTCATTTCACTAAT     419
HEINZ        TTATGTGATATTTTTCGTTTCTTGATATTCAAATTAAACTATCTCTGTCATTTCACTAAT     420
LA2093       TTATGTGATATTTTTCGTTTCTTGATATTCAAATTAAACTATCTCTGTCATTTCACTAAT     420
             ************************************************************

E42          TTAAGCGTCTTAGTTTGACTGAACACATCTTGTTGTGTTAATTAGATTTTTCATGTATGA     479
HEINZ        TTAAGCGTCTTAGTTTGACTGAACACATCTTGTTGTGTTAATTAGATTTTTCATGTATGA     480
LA2093       TTAAGCGTCTTAGTTTGACTGAACACATCTTGTTGTGTTAATTAGATTTTTCATGTATGA     480
             ************************************************************

E42          TGTTGAAATTTAAAGAACTTATTAAATATAGAAAAGAGACCGTCTTTTTAAGACGGATTA     539
HEINZ        TGTTGAAATTTAAAGAACTTATTAAATATAGAAAAGAGACCGTCTTTTTAAGACGGATTA     540
LA2093       TGTTGAAATTTAAAGAACTTATTAAATATAGAAAAGAGACCGTCTTTTTAAGACGGATTA     540
             ************************************************************

E42          AAAAGAAAAATATGACGATTAAATTGAAAAATAAGAAATATTTATATTTTGATCAAACAT     599
HEINZ        AAAAGAAAAATATGACGATTAAATTGAAAAATAAGAAATATTTATATTTTGATCAAACAT     600
LA2093       AAAAGAAAAATATGACGATTAAATTGAAAAATAAGAAATATTTATATTTTGATCAAACAT     600
             ************************************************************

E42          TTCAAAATATTTTTTTTTATCATATATATTGATAGTTGAAGAATTGCAACTTACTCTGGT     659
HEINZ        TTCAAAATATTTTTTTTTATCATATATATTGATAGTTGAAGAATTGCAACTTACTCTGGT     660
LA2093       TTCAAAATATTTTTTTTTATCATATATATTGATAGTTGAAGAATTGCAACTTACTCTGGT     660
             ************************************************************

E42          TTCATTTTTAGATTATCTTCTTCTTTTTTTTTTTTCCTTCTAACTATGTTGTTTTATTTC     719
HEINZ        TTCATTTTTAGATTATCTTCTTCTTTTTTTTTTTTCCTTCTAACTATGTTGTTTTATTTC     720
LA2093       TTCATTTTTAGATTATCTTCTTCTTTTTTTT-TTTTCTTCTAACTATGTTGTTTTATTTC     719
             ******************************* *** ************************

E42          TTTTGTTTGGAAAAGATCATGACTGATCCAGATGTTCCAGGTCCTAGTGATCCTTACTTA     779
HEINZ        TTTTGTTTGGAAAAGATCATGACTGATCCAGATGTTCCAGGTCCTAGTGATCCTTACTTA     780
LA2093       TTTTGTTTGGAAAAGATCATGACTGATCCAGATGTTCCAGGTCCTAGTGATCCTTACTTA     779
             ************************************************************
```

```
E42        AGGGAACACCTCCACTGGTAAGTATATTAATTGTTGATAAAAAAAATTGAATTT----AG    835
HEINZ      AGGGAACACCTCCACTGGTAAGTATATTAATTGTTGATAAAAAAAATTGAATTTACGTAG    840
LA2093     AGGGAACACCTCCACTGGTAAGTATATTAATTGTTGATAAAAAAAATTGAATTTACGTAG    839
           ******************************************************    **

E42        TTTAATTTCTAAATTATTAAAAGTCTTAAAAATATATTTTTACTTCATTAATTAAACTTA    895
HEINZ      TTTAATTTCTTAATTATTAAAAGTCTTAAAAATATATTTTTACTTGATTAATTAAACTTA    900
LA2093     TTTAATTTCTAAATTATTAAAAGTCTTAAAAATATATTTTTACTTGATTAATTAAACTTA    899
           ********** ********************************** **************

E42        AATACAGTCCTGATACACTTATGATCCGTCACCTGATATAGCAAGTGGTCTCAAATTTTT    955
HEINZ      AATACAGTCCTGATACACTTATGATCCGTCACTTGATATAGCAAGTGGTCTCAAATTTTT    960
LA2093     AATACAGTCCTGATACACTTATGATCCGTCACCTGATATAGCAAGTGGTCTCAAATTTTT    959
           ******************************** ***************************

E42        GTAGGAACATGAATCTTTTCATAAAAAACTGAGAGAATTGCTAAAAACATTCTGATAAGA    1015
HEINZ      GTAGGAACATAAATCTTTTCATAAAAAACTGAGAGAATTGCTAAAAACATTCTGATAAGA    1020
LA2093     GTAGGAACATGAATCTTTTCATAAAAAACTGAGAGAATTGCTAAAAACATTCTGATAAGA    1019
           ********** *************************************************

E42        ATTTAGGGACGTACTTCACTCATGTTACAATAACGGGGGGCGGAGCCACTTGTACAAAGG    1075
HEINZ      ATTTAGGGACGTACTTCACTCATGTTACAATAACGGGGGGCGGAGCCACTTGTACAAAGG    1080
LA2093     ATTTAGGGACGTACTTCACTCATGTTACAATAACGGGGGGCGGAGCCACTTGTACAAAGG    1079
           ************************************************************

E42        GAATTCATCCGAACCCCTTTTGGTGAAAAATTATATTGTTTATACATGGTTAAAATAATT    1135
HEINZ      GAATTCATCCGAACCCCTTTCGGCGAAAAATTATATTGTTTATACATGGTTAAAATAATT    1140
LA2093     GAATTCATCCGAACCCCTTTCGGCGAAAAATTATATTGTTTATACATGGTTAAAATAATT    1139
           ******************** ** ************************************

E42        TTATATGTATATATAGTAGATGTCGGACCTCCTTCGGCTACTGCATGTGTCTATTTCTGC    1195
HEINZ      TTATATGTATATATAGTAGATGTCGGACCTCCTTCGGCTACTGCATGTGTCTATTTCTGC    1200
LA2093     TTATATGTATATATAGTAGATGTCGGACCTCCTTCGGCTACTGCATGTGTCTATTTCTGC    1199
           ************************************************************

E42        AGATTTTGAATCCCCTTATTGAAAATCCTGACTCTACCTCTAATAACAAGAGTGTATTTT    1255
HEINZ      AGATTTTGAATCCCCTTATTGAAAATCCTGACTCTACCTCTAATAACAAGAGTGTATTTT    1260
LA2093     AGATTTTGAATCCCCTTATTAAAAATCCTGACTCTACCTCTAATAACAAGAGTGTATTTT    1259
           ******************** ***************************************

E42        TAAGTTTATTTAATCATATAAATAAGTATTTTTAAAACTATTAATAGTTCGAAAATAAAA    1315
HEINZ      TAAGTTTATTTAATCATATAAATAAGTATTTTTAAAACTATTAATAGTTCGAAAATGAAA    1320
LA2093     TAAGTTTATTTAATCATATAAATAAGTATTTTTAAAACTATTAATAGTTCGAAAATGAAA    1319
           ******************************************************** ***

E42        CCGAACCGCTTACGCCAAATTTGATAGTGTTTCCACTACTTCTCTCAAAGGGTAATCATA    1375
HEINZ      CCGAACCGTTTACGCCAAATTTGATAGTGTTTCCACTACTTCTCTCAAAGGGTAATCATA    1380
LA2093     CCGAACCGTTTACGCCAAATTTGATAGTGTTTCCACTACTTCTCTCAAAGGGTAATCATA    1379
           ******** ***************************************************

E42        TTTGCATGGAAATTAAATTTGGTTTTGTTTTTTCAGGATTGTGACAGATATTCCTGGTTC    1435
HEINZ      TTTGCATGGAAATTAAATTTGGTTTTGTTTTTTCAGGATTGTGACAGATATTCCTGGTTC    1440
LA2093     TTTGCATGGAAATTAAATTTGGTTTTGTTTTTTCAGGATTGTGACAGATATTCCTGGTTC    1439
           ************************************************************

E42        AACTGATGCTTCTTTTGGTAAAAAATAAGCAAATTAACTTTTAACTTTTCCGTTAACATT    1495
HEINZ      AACTGATGTTTCTTTTGGTAAAAAATAAGCAAATTAACTTTTAACTTTTCCGTTAACATT    1500
LA2093     AACTGATGCTTCTTTTGGTAAAAAATAAGCAAATTAACTTTTAACTTTTCCGTTAACATT    1499
           ******** ***************************************************

E42        TTATTTACTTAATTATATCGTTAACAAATGATTAGATATGTAGTAATACGTTAAATTTAT    1555
HEINZ      TTATTTACTTAATTATATCGTTAACAAATGATTAGATATGTAGTAATACGTTAAATTTAT    1560
LA2093     TTATTTACTTAATTATATCATTAACAAATGATTAGATATGTAGTAATACGTTAAATTTAT    1559
           ******************* ****************************************

E42        ACAGGAAAAGAGATAGTGAGCTACGAGAGTCCAAAACCAGTGATTGGAATTCATCGATAC    1615
HEINZ      ACAGGAAAAGAGATAGTGAGCTACGAGAGTCCAAAACCAGTGATTGGAATTCATCGATAC    1620
LA2093     ACAGGAAAAGAGATAGTGAGCTACGAGAGTCCAAAACCAGTGATTGGAATTCATCGATAC    1619
           ************************************************************

E42        GTGTTCATATTATATAAGCAAAATAGAGGAAGACAGACAGTGAAACCACCAGTAACAAGA    1675
HEINZ      GTGTTCATATTATATAAGCAAAATAGAGGAAGACAGACAGTGAAACCACCAGTAACAAGA    1680
```

```
LA2093      GTGTTCATATTATATAAGCAAAATAAAGGAAGACAGACAGTGAAACCACCAGTAACAAGA    1679
            ************************* **********************************

E42         GATCATTTCAACGCTCGAAAATTTGCGGTAGAAAATGGATTGGGTTCCCCTGTTGCTGCT    1735
HEINZ       GATCATTTCAACGCTCGAAAATTTGCGGTAGAAAATGGATTGGGTTCCCCTGTTGCTGCT    1740
LA2093      GATCATTTCAACGCTCGAAAATTTGCGGTAGAAAATGGATTGGGTTCCCCTGTTGCTGCT    1739
            ************************************************************

E42         GTATACTTTAATGCACAAAGAGAAACTGCTGCTAGAAGAAGATGA    1780
HEINZ       GTATACTTTAATGCACAAAGAGAAACTGCTGCTAGAAGAAGATGA    1785
LA2093      GTATACTTTAATGCACAAAGAGAAACTGCTGCTAGAAGAAGATGA    1784
            *********************************************
```

Solyc01g056310

```
HEINZ       AGTGCAAGCTCTAGCAGGAAGAAGAACTAATAAAAAAAAGAAGAAGAAGCCTGACCTCTT    60
E42         AGTGCAAGCTCTAGCAGGAAGAAGAACTAATAAAAAAAAGAAGAAGAAGCCTGACCTCTT    60
LA2093      AGTGCAAGCTCTAGCAGGAAGAAGAACTAATAAAAAAAAGAAGAAGAAGCCTGACCTCTT    60
            ************************************************************

HEINZ       CTTCTTGTGAGAGTAAAAATATATAAAACTCCCAAAAAAATTACTAGAGTGATCAAAAAT    120
E42         CTTCTTGTGAGAGTAAAAATATATAAAACTCCCAAAAAAATTACTAGAGTGATCAAAAAT    120
LA2093      CTTCTTGTGAGAGTAAAAATATATAAAACTCCCAAAAAAATTACTAGAGTGATCAAAAAT    120
            ************************************************************

HEINZ       AGAAAAAAAAAGAAGCATCGTCATGGGGAGTGGTAAAGTGACAGTTGTGGCTTTGCTACT    180
E42         AGAAAAAAAAAGAAGCATCGTCATGGGGAGTGGTAAAGTGACAGTTGTGGCTTTGCTACT    180
LA2093      AGAAAAAAAAAGAAGCATCGTCATGGGGAGTGGTAAAGTGACAGTTGTGGCTTTGCTACT    180
            ************************************************************

HEINZ       TTGCCTCTCAGTAGGGGTAATAGCTGAGGACCCTTACCTCTACTTTAACTGGAACGTTAC    240
E42         TTGCCTCTCAGTAGGGGTAATAGCTGAGGACCCTTACCTCTACTTTAACTGGAACGTTAC    240
LA2093      TTGCCTCTCAGTAGGGGTAATAGCTGAGGACCCTTACCTCTACTTTAACTGGAACGTTAC    240
            ************************************************************

HEINZ       CTATGGCACAGTCTCTCCATTGGGCGTGCCACAACAAGGTATTCTCATCAATGGCCAGTT    300
E42         CTATGGCACAGTCTCTCCATTGGGCGTGCCACAACAAGGTATTCTCATCAATGGCCAGTT    300
LA2093      CTATGGCACAGTCTCTCCATTGGGCGTGCCACAACAAGGTATTCTCATCAATGGCCAGTT    300
            ************************************************************

HEINZ       CCCTGGGCCTAGAATTAATTGTACCTCCAACAACAACATTGTCGTCAATGTCTTCAATAA    360
E42         CCCTGGGCCTAGAATTAATTGTACCTCCAACAACAACATTGTCGTCAATGTCTTCAATAA    360
LA2093      CCCTGGGCCTAGAATTAATTGTACCTCCAACAACAACATTGTCGTCAATGTCTTCAATAA    360
            ************************************************************

HEINZ       TTTGGATGAGCCATTCCTATTTACCTGGAATGGTGTCCAACATAGGAAGAACTCATGGCA    420
E42         TTTGGATGAGCCATTCCTATTTACCTGGAATGGTGTCCAACATAGGAAGAACTCATGGCA    420
LA2093      TTTGGATGAGCCATTCCTATTTACCTGGAATGGTGTCCAACATAGGAAGAACTCATGGCA    420
            ************************************************************

HEINZ       AGATGGTACCCCAGGAACCATGTGCCCAATCATGCCTGGTCAAAATTTTACTTACCGATT    480
E42         AGATGGTACCCCAGGAACCATGTGCCCAATCATGCCTGGTCAAAATTTTACTTACCGATT    480
LA2093      AGATGGTACCCCAGGAACCATGTGCCCAATCATGCCTGGTCAAAATTTTACTTACCGATT    480
            ************************************************************

HEINZ       CCAGGTCAAGGACCAGATTGGTAGCTACTACTACTTCCCTACCACAGCTTTGCACCGAGC    540
E42         CCAGGTCAAGGACCAGATTGGTAGCTACTACTACTTCCCTACCACAGCTTTGCACCGAGC    540
LA2093      CCAGGTCAAGGACCAGATTGGTAGCTACTACTACTTCCCTACCACAGCTTTGCACCGAGC    540
            ************************************************************

HEINZ       AGCAGGTGGTTATGGTGCCATCAATGTCCACAGTCGTGCTCTCATCCCTGTTCCTTTCGA    600
E42         AGCAGGTGGTTATGGTGCCATCAATGTCCACAGTCGTGCTCTCATCCCTGTTCCTTTCGA    600
LA2093      AGCAGGTGGTTATGGTGCCATCAATGTCCACAGTCGTGCTCTCATCCCTGTTCCTTTCGA    600
            ************************************************************

HEINZ       CAATCCTGCCGATGAGTACAATGTCTTTGTCGGTGATTGGTACAACAAGGGCCACAAGAC    660
E42         CAATCCTGCCGATGAGTACAATGTCTTTGTCGGTGATTGGTACAACAAGGGCCACAAGAC    660
LA2093      CAATCCTGCCGATGAGTACAATGTCTTTGTCGGTGATTGGTACAACAAGGGCCACAAGAC    660
            ************************************************************

HEINZ       TTTGAAAAAGGTCTTGGATGGTGGACACACCGTTGGCAGACCTGATGGCATCATCATCAA    720
E42         TTTGAAAAAGGTCTTGGATGGTGGACACACCGTTGGCAGACCTGATGGCATCATCATCAA    720
LA2093      TTTGAAAAAGGTCTTGGATGGTGGACACACCGTTGGCAGACCTGATGGCATCATCATCAA    720
            ************************************************************

HEINZ       TGGTAAGTCTGCTAAGGTTGGAGAGGCAAAAGAGCCACTCTTTACCATGGAGGCTGGCAA    780
E42         TGGTAAGTCTGCTAAGGTTGGAGAGGCAAAAGAGCCACTCTTTACCATGGAGGCTGGCAA    780
LA2093      TGGTAAGTCTGCTAAGGTTGGAGAGGCAAAAGAGCCACTCTTTACCATGGAGGCTGGCAA    780
            ************************************************************

HEINZ       GACCTATAGGTACAGATTCTGTAACCTTGGCATGAGGTCCTCAGTCAACGTTAGATTACA    840
E42         GACCTATAGGTACAGATTCTGTAACCTTGGCATGAGGTCCTCAGTCAACGCTAGATTCCA    840
LA2093      GACCTATAGGTACAGATTCTGTAACCTTGGCATGAGGTCCTCAGTCAACGTTAGATTCCA    840
            ************************************************** ****** **
```

```
HEINZ      AGGTCACCCAATGAAATTAGTCGAGCTAGAGGGATCCCACACCGTACAAAACATCTACGA    900
E42        AGGTCACCCAATGAAATTAGTCGAGCTAGAGGGATCCCACACCGTACAAAACATCTACGA    900
LA2093     AGGTCACCCAATGAAATTAGTCGAGCTAGAGGGATCCCACACCGTACAAAACATCTACGA    900
           ************************************************************

HEINZ      TTCATTGGATATCCATGTTGGTCAGTGCCTCTCAGTATTGGTGACTGCTAATCAGGAGCC    960
E42        TTCATTGGATATCCATGTTGGTCAGTGCCTCTCAGTATTGGTGACTGCTAATCAGGAGCC    960
LA2093     TTCATTGGATATCCATGTTGGTCAGTGCCTCTCAGTATTGGTGACTGCTAATCAGGAGCC    960
           ************************************************************

HEINZ      CAAGGACTATTACTTGGTCGTTTCAAGCAGATTCTTGAAGCAAGAACTCTCCTCTGTGGC    1020
E42        CAAGGACTATTACTTGGTCGTTTCAAGCAGATTCTTGAAGCAAGAACTCTCCTCTGTGGC    1020
LA2093     CAAGGACTATTACTTGGTCGTTTCAAGCAGATTCTTGAAGCAAGAACTCTCCTCTGTGGC    1020
           ************************************************************

HEINZ      CATCATCCGTTATGCCAATGGAAAGGGCCCAGCATCTCCTGAGCTCCCAGCATCCCCACC    1080
E42        CATCATCCGTTATGCCAATGGAAAGGGCCCAGCATCTCCTGAGCTCCCAGCATCCCCACC    1080
LA2093     CATCATCCGTTATGCCAATGGAAAGGGCCCAGCATCTCCTGAGCTCCCAGCATCCCCACC    1080
           ************************************************************

HEINZ      AGACAACACTGAAGGCATTGCCTGGTCCATGAACCAGTTCCGCTCCTTCAGATGGAACTT    1140
E42        AGACAACACTGAAGGCATTGCCTGGTCCATGAACCAGTTCCGCTCCTTCAGATGGAACTT    1140
LA2093     AGACAACACTGAAGGCATTGCCTGGTCCATGAACCAGTTCCGCTCCTTCAGATGGAACTT    1140
           ************************************************************

HEINZ      GACCGCTAGCGCTGCCCGTCCCAACCCTCAGGGTTCCTACCATTACGGACAGATAAACAT    1200
E42        GACCGCTAGCGCTGCCCGTCCCAACCCTCAGGGTTCCTACCATTACGGACAGATAAACAT    1200
LA2093     GACCGCTAGCGCTGCCCGTCCCAACCCTCAGGGTTCCTACCATTACGGACAGATAAACAT    1200
           ************************************************************

HEINZ      CACCCGCACCATCAAGATTGTCAACTCTATGGGCCAAGTAAATGGTAAGCTTAGATATGG    1260
E42        CACCCGCACCATCAAGATTGTCAACTCTATGGGCCAAGTAAATGGTAAGCTTAGATATGG    1260
LA2093     CACCCGCACCATCAAGATTGTCAACTCTATGGGCCAAGTAAATGGTAAGCTTAGATATGG    1260
           ************************************************************

HEINZ      TTTGAACGGCATCTCTCACTCAGATACCGAAACTCCATTGAAGCTTGTAGAGTACTTTGG    1320
E42        TTTGAACGGTATCTCTCACTCAGATACCGAAACTCCATTGAAGCTTGTAGAGTACTTTGG    1320
LA2093     TTTGAACGGTATCTCTCACTCAGATACCGAAACTCCATTGAAGCTTGTAGAGTACTTTGG    1320
           ********* **************************************************

HEINZ      AGCTGCCGATAAGAGTTTCAAGTATGATCTCATGGCTGATGAAGCCCCAGCTGACCCAAG    1380
E42        AGCTGCCGATAAGAGTTTCAAGTATGATCTCATGGCTGATGAAGCCCCAGCTGACCCAAG    1380
LA2093     AGCTGCCGATAAGAGTTTCAAGTATGATCTCATGGCTGATGAAGCCCCAGCTGACCCAAG    1380
           ************************************************************

HEINZ      CAAGCTAACTGTTGCCCCAAATGTGAAAAACACCACCTACCGTAACTTTGTGGAGATCAT    1440
E42        CAAGCTAACTGTTGCCCCAAATGTGAAAAACACCACCTACCGTAACTTTGTGGAGATCAT    1440
LA2093     CAAGCTAACTGTTGCCCCAAATGTGAAAAACACCACCTACCGTAACTTTGTGGAGATCAT    1440
           ************************************************************

HEINZ      CTTTGAGAACCACGAGAAGAGCATCAGGACATATCACCTTGATGGATATTCCTTCTTTGC    1500
E42        CTTTGAGAACCACGAGAAGAGCATCAGGACATATCACCTTGATGGATATTCCTTCTTTGC    1500
LA2093     CTTTGAGAACCACGAGAAGAGCATCAGGACATATCACCTTGATGGATATTCCTTCTTTGC    1500
           ************************************************************

HEINZ      AGTAGCTGTTGAGCCAGGGAGGTGGAGCGCTGAGAAGAGAAAGAACTACAACTTAGTGGA    1560
E42        AGTAGCTGTTGAGCCAGGGAGGTGGAGCGCTGAGAAGAGAAAGAACTACAACTTAGTGGA    1560
LA2093     AGTAGCTGTTGAGCCAGGGAGGTGGAGCGCTGAGAAGAGAAAGAACTACAACTTAGTGGA    1560
           ************************************************************

HEINZ      CGCAACAAGCAGGAACAACATCCAGGTGTATCCAAACTCATGGGCAGCAATAATGTTGAC    1620
E42        CGCAACAAGCAGGAACAACATCCAGGTGTATCCAAACTCATGGGCAGCAATAATGTTGAC    1620
LA2093     CGCAACAAGCAGGAACAACATCCAGGTGTATCCAAACTCATGGGCAGCAATAATGTTGAC    1620
           ************************************************************

HEINZ      ATTTGACAATGCAGGAATGTGGAACTTGAGGTCAGAGATGTGGGAGAAGACTTATTTGGG    1680
E42        ATTTGACAATGCAGGAATGTGGAACTTGAGGTCAGAGATGTGGGAGAAGACTTATTTGGG    1680
LA2093     ATTTGACAATGCAGGAATGTGGAACTTGAGGTCAGAGATGTGGGAGAAGACTTATTTGGG    1680
           ************************************************************

HEINZ      AGAGCAAATGTACTTCAGCGTTCTCTCACCAAGCCGCTCCTTGAGAGATGAATACAACCT    1740
```

```
E42           AGAGCAAATGTACTTCAGCGTTCTCTCACCAAGCCGCTCCTTGAGAGATGAATACAACCT    1740
LA2093        AGAGCAAATGTACTTCAGCGTTCTCTCACCAAGCCGCTCCTTGAGAGATGAATACAACCT    1740
              ************************************************************

HEINZ         CCCTGATAACCATCCTCTCTGCGGTGTTGTCAAGGGCATGCCCCTCCCAGCTCCATACAA    1800
E42           CCCTGATAACCATCCTCTCTGCGGTGTTGTCAAGGGCATGCCCCTCCCAGCTCCATACAA    1800
LA2093        CCCTGATAACCATCCTCTCTGCGGTGTTGTCAAGGGCATGCCCCTCCCAGCTCCATACAA    1800
              ************************************************************

HEINZ         GGCTTAATTGAATCTCTTCTCTTTCTATCTAATAATAAAGGGGACGGCTTAACTATTAAT    1860
E42           GGCTTAATTGAATCTCTTCTCTTTCTATCTAATAATAAAGGGGACGGCTTAACTATTAAT    1860
LA2093        GGCTTAATTGAATCTCTTCTCTTTCTATCTAATAATAAAGGGGACGGCTTAACTATTAAT    1860
              ************************************************************

HEINZ         GGATTCCATGTATTTGTTTTCCTTCCCCCCAAAAAATAATGAATAAAAAGTTTTTTCTTT    1920
E42           GGATTCCATGTATTTGTTTTCCTTCCCCCCAAAAAATAATGAATAAAAAGTTTTTTCTTT    1920
LA2093        GGATTCCATGTATTTGTTTTCCTTCCCCCCAAAAAATAATGAATAAAAAGTTTTTTCTTT    1920
              ************************************************************

HEINZ         CTTTCAAAATTATGCATTTTTCGTTTCTCTCCTACTCCTTCATCATATTATTTGAACTCA    1980
E42           CTTTCAAAATTATGCATTTTTCGTTTCTCTCCTACTCCTTCATCATATTATTTGAACTCA    1980
LA2093        CTTTCAAAATTATGCATTTTTCGTTTCTCTCCTACTCCTTCATCATATTATTTGAACTCA    1980
              ************************************************************

HEINZ         AACACCAAAACATTAATAAATTCATTATCTTGATCCATATG 2021
E42           AACACCAAAACATTAATAAATTCATTATCTTGATCCATATG 2021
LA2093        AACACCAAAACATTAATAAATTCATTATCTTGATCCATATG 2021
              *****************************************
```

Solyc01g066680

```
E42         CATGATAATTTTTTTGCGCAGATCGTATTTGGAAATATATAAAGAAAATAAAAATGAAAA    60
HEINZ       CATGATAATTTTTTTGCGCAGATCGTATTTGGAAATATATAAAGAAAATAAAAATGAAAA    60
LA2093      CATGATAATTTTTTTGCGCAGATCGTATTTGGAAATATATAAAGAAAATAAAAATGAAAA    60
            ************************************************************

E42         CAATACGAGTAGAGTAATATTACACTTAACTTGTATAATGACAAAACATTGAAACAATTA    120
HEINZ       CAATACAAGTAGAGTAATATTACACTTAACTTGTATAATGACAAAACATTGAAACAATTA    120
LA2093      CAATACGAGTAGAGTAATATTACACTTAACTTGTATAATGACAAAACATTGAAACAATTA    120
            ****** *****************************************************

E42         CAGTGGACAGAAAGAAGAATCCTTATTTATGCCATCAGCAGCAACACTAACTAGCTGATG    180
HEINZ       CAGTGGACAGAAAGAAGAATCCTTATTTATGCCATCAGCAGCAACACTAACTAGCTGATG    180
LA2093      CAGTGGACAGGAAGAAGAATCCTTATTTATGCCATCAGCAGCAACACTAACTAGCTGATG    180
            ********** *************************************************

E42         CATCACTAGGAAACATTAATTAATAACGTAACACATAATATAAAGGAGTAGCATTTATTT    240
HEINZ       CATCACTAGGAAACATTAATTAATAACGTAACACATAATATAAAGGAGTAGCATTTATTT    240
LA2093      CATCACTAGGAAACATTAATTAATAACGTAACACATAATATAAAGGAGTAGCATTTATTT    240
            ************************************************************

E42         ATTTAGTTAATAAGTTGAAAAGCACCAGTAACAAGTTTGACAATATTAAGAAGTCCTCCA    300
HEINZ       ATTTAGTTAATAAGTTGAAAAGCACCAGTAACAAGTTTGACAATATTAAGAAGTCCTCCA    300
LA2093      ATTTAGTTAATAAGTTGAAAAGCACCAGTAACAAGTTTGACAATATTAAGAAGTCCTCCA    300
            ************************************************************

E42         CCAGCGGTTTTGCCAACAATCTGCAAAGGTGCCTGCAGAACCCCAACGGATGGTAAGGTA    360
HEINZ       CCAGCGGTTTTGCCAACAATCTGCAAAGGTGCCTGCAGAACCCCAACGGATGGTAAGGTA    360
LA2093      CCAGCGGTTTTGCCAACAATCTGCAAAGGTGCCTGCAGAACCCCAACGGATGGTAAGGTA    360
            ************************************************************

E42         GCGTTACATGTTGAGAGCGGAGTTGTAACCACTATGTTGCAGTCAGACAACAATGATGAT    420
HEINZ       GCGTTACATGTTGAGAGCGGAGTTGTAACCACTATGTTGCAGTCAGACAACAATGATGAT    420
LA2093      GCGTTACATGTTGAGAGCGGAGTTGTAACCACTATGTTGCAGTCAGACAACAATGATGAT    420
            ************************************************************

E42         AATAAACTTTCTACAGGACTCGTCACCAACGAAAACGCTCCTGATCCATTTGTTGTTGTA    480
HEINZ       AATAAACTTTGTACAGGACTCGTCACCAACGAAAACGCTCCTGATCCATTTGTTGTTGTA    480
LA2093      AATAAACTTTGTACAGGACTCGTCACCAACGAAAACGCTCCTGATCCATTTGTTGTTGTA    480
            ********** *************************************************

E42         CTTGATACTACATTTCCTGCTCCACACCGCAGCTGCACCGATGCATCTGCAATAAACAAA    540
HEINZ       CTTGATACTACATTTCCTGCTCCACACCGCAGCTGCACCGATGCATCTGCAATAAACAAA    540
LA2093      CTTGATACTACATTTCCTGCTCCACACCGCAGCTGCACCGATGCATCTGCAATAAACAAA    540
            ************************************************************

E42         CATGCTCACTAACGACATAATTCTTACGTACATGTGTATGTATACTCACAAAAAAGCAAG    600
HEINZ       CATGCTCACTAACGACATAATTCTTACGTACATGTGTATGTATACTCACAAAAAAGCAAG    600
LA2093      CATGCTCACTAACGACATAATTCTTACGTACATGTGTATGTATACTCACAAAAAAGCAAG    600
            ************************************************************

E42         AGTGGGAGACAAGTGAAATGTACATGTATATTGGTTATTATACATATGTATTTGTATATA    660
HEINZ       AGTGGGAGACAAGTGAAATGTACATGTATATTGGTTATTATACATATGTATTTGTATATA    660
LA2093      AGTGGGAGACAAGTGAAATGTACATGTATATTGGTTATTATACATATGTATTTGTATATA    660
            ************************************************************

E42         TGATAAGTCAGATTGGGGAAAGAGGTCAATTGTATATGTATATCGGTTAGATAATTGTAT    720
HEINZ       TGATAAGTCAGATTGGGGAAAGAGGTCAATTGTATATGTATATCGGTTAGATAATTGTAT    720
LA2093      TGATAAGTCAGATTGGGGAAAGAGGTCAATTGTATATGTATATCGGTTAGATAATTGTAT    720
            ************************************************************

E42         ATTATACATATGTGTTTGTATATTCTGGCGAATTATACCTATACAAACGTGGTTTATCAT    780
HEINZ       ATTATACATATGTGTTTGTATATTCTGGCGAATTATACCTATACAAACGTGGTTTATCAT    780
LA2093      ATTATACATATGTGTTTGTATATTCTGGCGAATTATACCTATACAAACGTGGTTTATCAT    780
            ************************************************************

E42         ACAAACTACATGATTAATGTATGATTTTAGTCGCAAGTGATAATTATAGCAAACTATAGT    840
HEINZ       ACAAACTACATGATTAATGTATGATTTTAGTCGCGAGTGATAATTATAGCAAACTATAGT    840
LA2093      ACAAACTACATGATTAATGTATGATTTTAGTCGCGAGTGATAATTATAGCAAACTATAGT    840
            ********************************** *************************
```

```
E42         TATGATGAGTAAATTAGTAGTACAAATTTGTTTAACCGCGTAATTTCCCTTACAAAGAAT    900
HEINZ       TATGATGAGTAAATTAGTAGTACAAATTTGTTTAACCGCGTAATTTCCCTTACAAAGAAT    900
LA2093      TATGATGAGTAAATTAGTAGTACAAATTTGTTTAACCGCGTAATTTCCCTTACAAAGAAT    900
            ************************************************************

E42         TAAGGAACTTACTGGGGAAAATTGGGGTGGTAGCTCCATTGAGGACATCTATTTTGCCGT    960
HEINZ       TAAGGAACTTACCGGGGAAAATTGGGGTGGTAGCTCCATTGAGGACATCTATTTTGCCGT    960
LA2093      TAAGGAACTTACCGGGGAAAATTGGGGTGGTAGCTCCATTGAGGACATCTATTTTGCCGT    960
            ************ ***********************************************

E42         TAAGGCTACAAAACAGAACCCCTTCTATACTTGTTGGGGCAAGAAGGCCACCCAGAAGTC    1020
HEINZ       TAAGGCTACAAAACAGAACCCCTTCTATACTTGTTGGGGCAAGAAGGCCACCCAGAAGTC    1020
LA2093      TAAGGCTACAAAACAGAACCCCTTCTATACTTGTTGGGGCAAGAAGGCCACCCAGAAGTC    1020
            ************************************************************

E42         CTCCTAATTGGGCTACGGCAACAGGGGTTGCAATTAATGCAGCCAAAATGAAAATCAAGA    1080
HEINZ       CTCCTAATTGGGCTACGGCAACAGGGGTTGCAATTAATGCAGCCAAAATGAAAATCAAGA    1080
LA2093      CTCCTAATTGGGCTACGGCAACAGGGGTTGCAATTAATGCAGCCAAAATGAAAATCAAGA    1080
            ************************************************************

E42         AAACTTTTGATGAGGCCATAGCAACTTCTTATATATGTGTGGTATGGCTAATTAATTAAG    1140
HEINZ       AAACTTTTGATGAGGCCATAGCAACTTCTTATATATGTGTGGTATGGCTAATTAATTAAG    1140
LA2093      AAACTTTTGATGAGGCCATAGCAACTTCTTATATATGTGTGGTATGGCTAATTAATTAAG    1140
            ************************************************************

E42         TTGTTGTTGTACGAAT   1156
HEINZ       TTGTTGTTGTACGAAT   1156
LA2093      TTGTTGTTGTACGAAT   1156
            ****************
```

Solyc01g066770

```
LA2093      TGATATAAACATGTTAATATATCACTTGTCAAACATTGATAGAATGCAAAACTCGTACAC    60
E42         TGATATAAACATGTTAATATATCACTTGTCAAACATTGATAGAATGCAAAACTCGTACAC    60
HEINZ       TGATATAAACATGTTAATATATCACTTGTCAAACATTGATAGAATGCAAAACACGTACAC    60
            **************************************************** *******

LA2093      ATGACGAACTTTCTAACAATGAAAACTTGGTTCGCCCAAAAACACTTGTTCTCCCCCACA    120
E42         ATGACGAACTTTCTAACAATGAAAACTTGGTTCGCCCAAAAACACTTGTTCTCCCCCACA    120
HEINZ       ATGACGAACTTTCTAACAATGAAAACTTGGTTCGCCCAAAAACACTTGTTCTCCCCCACA    120
            ************************************************************

LA2093      ACCCTAAATCATAATGGCCTGAAACATGACGCTCCATGGACAGTGTTTCGATCGCAGTTC    180
E42         ACCCTAAATCATAATGGCCTGAAACATGACGCTCCATGGACAGTGTTTCGATCGCAGTTC    180
HEINZ       ACCCTAAATCATAATGGCCTGAAACATGACGCTCCATGGACAGTGTTTCGATCGCAGTTC    180
            ************************************************************

LA2093      TGAATGTCAAGCTATATATTGCGATCCCTCTGCTGGCAGCCTCGTTGATGTTAGTTTCAA    240
E42         TGAATGTCAAGCTATATATTGCGATCCCTCTGCTGGCAGCCTCGTTGATGTTAGTTTCAA    240
HEINZ       TGAATGTCAAGCTATATATTGCGATCCCTCTGCTGGCAGCCTCGTTGATGTTAGTTTCAA    240
            ************************************************************

LA2093      TCGCGCCATGACATGCTATCAAGTGACAATATAACTGGACTATAAGATGGCTGGTCATAC    300
E42         TCGCGCCATGACATGCTATCAAGTGACAATATAACTGGACTATAAGATGGCTGGTCATAC    300
HEINZ       TCGCGCCATGACATGCTATCAAGTGACAATATAACTGGACTATAAGATGGCTGGTCATAC    300
            ************************************************************

LA2093      TTCATCAACCAAGCAGAAGAGCCTATTCCTTCACAGAATACTACAAATCTTCAATTCCCA    360
E42         TTCATCAACCAAGCAGAAGAGCCTATTCCTTCACAGAATACTACAAATCTTCAATTCCCA    360
HEINZ       TTCATCAACCAAGCAGAAGAGCCTATTCCTTCACAGAATACTACAAATCTTCAATTCCCA    360
            ************************************************************

LA2093      CCACCTTATAGCTCCATGCACACAGTCCACCTGCAATACAAGTAGAGCAAACAAACAACT    420
E42         CCACCTTATAGCTCCATGCACACAGTCCACCTGCAATACAAGTAGAGCAAACAAACAACT    420
HEINZ       CCACCTTATAGCTCCATGCACACAGTCCACCTGCAATACAAGTAGAGCAAACAAACAACT    420
            ************************************************************

LA2093      AATTACTAGCCATATCAGAAAATGGTACTTCGAAGATTAGGCCACATTACACACAATCCA    480
E42         AATTACTAGCCATATCAGAAAATGGTACTTCGAAGATTAGGCCACATTACACACAATCCA    480
HEINZ       AATTACTAGCCATATCAGAAAATGGTACTTCGAAGATTAGGCCACATTACACACAATCCA    480
            ************************************************************

LA2093      ACGATAATCCATGTACATGTGGGGACATTACTCGAAAGTTAATAGGA-ATAAATTACTAG    539
E42         ACGATAATCCATGTACATGTGGGGACATTACTCGAAGGTTAATAGGAAATAAATTACTAG    540
HEINZ       ACGATAATCCATGTACATGTGGGGACATTACTCGAAGGTTAATAGGAAATAAATTACTAG    540
            ************************************ ********** ************

LA2093      AGCTGAGATGAAATATGCATATACCATACGAACCACAGGCCAAGGAGGCAAGCCAGAGTA    599
E42         AGCTGAGATGAAATATGCATATACCATACGAACCACAGGCCAAGGAGGCAAGCCAGAGTA    600
HEINZ       AGCTGAGATGAAATATGCATATACCATACGAACCACAGGCCAAGGAGGCAAGCCAGAGTA    600
            ************************************************************

LA2093      AAAAAGTAACACATTTATCAAAATACAAAAGTCATTAGATAAAAACAGAAGCTGTCGTCA    659
E42         AAAAAGTAACACATTTATCAAAATACAAAAGTCATTAGATAAAAACAGAAGCTGTCGTCA    660
HEINZ       AAAAAGTAACACATTTATCAAAATACAAAAGTCATTAGATAAAAACAGAAGCTGTCGTCA    660
            ************************************************************

LA2093      GCCCAACTTGCATTATCTGAAGGTGAAATCAAGATAATCTATGAATAGATGTTACATAAA    719
E42         GCCCAACTTGCATTATCTGAAGGTGAAATCAAGATAATCTATGAATAGATGTTACATAAA    720
HEINZ       GCCCAACTTGCATTATCTGAAGGTGAAATCAAGATAATCTATGAATAGATGTTACATAAA    720
            ************************************************************

LA2093      ATTTTGAAACTCGTCCAAGAAATAAATAGAATCTTGCGGTTTTAAGCAATGCAAGCAAAC    779
E42         ATTTTGAAACTCGTCCAAGAAATAAATAGAATCTTGCGGTTTTAAGCAATGCAAGCAAAC    780
HEINZ       ATTTTGAAACTCGTCCAAGAAATAAATAGAATCTTGCTGTTTTAAGCAATGCAAGCAAAC    780
            ************************************* **********************

LA2093      AATTTCACTCTTTACTTTTTCTTGACAAAATACACCACTACAGCTTTCTTCAGTCAGAAG    839
E42         AATTTCACTCTTTACTTTTTCTTGACAAAATACACCACTACAGCTTTCTTCAGTCAGAAG    840
HEINZ       AATTTCACTCTTTACTTTTTCTTGACAAAATACACCACTACAGCTTTCTTCAGTCAGAAG    840
            ************************************************************
```

```
LA2093      TTTTTT-TTTTTCAAGCATTCAAATTAACTAGAACCCCAACACCCAGGTCACCTATCTCT    898
E42         TTTTTTTATTTTCAAGCATTCAAATTAACTAGAACCCCAACACCCAGGTCACCTATCTCT    900
HEINZ       TTTTTTTTTTTTCAAGCATTCAAATTAACTAGAACCCCAACACCCAGGTCACCTATCTCT    900
            ******  ****************************************************

LA2093      CCATCACCCTATTTTTCCAATTCCTGTACATGTCAGCGGTCAACCAAAAAATATACAAAT    958
E42         CCATCACCCTATTTTTCCAATTCCTGTACATGTCAGCGGTCAACCAAAAAATATACAAAT    960
HEINZ       CCATCACCCTATTTTTCCAATTCCTGTACATGTCAGCGGTCAACCAAAAAATATACAAAT    960
            ************************************************************

LA2093      TTATTAACAATCTGTTAGCATGTTTGCCCGAACACCTGGGAAGCCAAAAATTCTTAATTT    1018
E42         TTATTAACAATCTGTTAGCATGTTTGCCCGAACACCTGGGAAGCCAAAAATTCTTAATTT    1020
HEINZ       TTATTAACAATCTGTTAGCATGTTTGCCCGAACACCTGGGAAGCCAAAAATTCTTAATTT    1020
            ************************************************************

LA2093      TTTAGAGTTGAGGTGCTTAAGCTTTTAGGAAAAAGAAGTGCTTTTGAGAAAAAGTAGCTT    1078
E42         TTTAGAGTTGAGGTGCTTAAGCTTTTAGGAAAAAGAAGTGCTTTTGAGAAAAAGTAGCTT    1080
HEINZ       TTTAGAGTTGAGGTGCTTAAGCTTTTAGGAAAAAGAAGTGCTTTTGAGAAAAAGTAGCTT    1080
            ************************************************************

LA2093      CTCCCTGGGAACACTTTTTGAATCTTGGTCAAGCACAAATAGTTGATCTAACATTGACAA    1138
E42         CTCCCTGGGAACACTTTTTGAATCTTGGTCAAGCACAAATAGTTGATCTAACATTGACAA    1140
HEINZ       CTCCCTGGGAACATTTTTTGAATCATGGTCAAGCACAAATTGTTGATCTAACATTGACAA    1140
            ************* ********** *************** *******************

LA2093      AGGCACTTTCCAAATTAGTCAAACACAAACTACTACTCTCCATTAGTACTTTCTCGAACA    1198
E42         AGGCACTTTCCAAATTAGTCAAACACAAACTACTACTCTCCATTAGTACTTTCTCGAACA    1200
HEINZ       AGGCACTTTCCAAATTAGTCAAACACAAACTACTACTCTCCATTAGTACTTTCTCGAACA    1200
            ************************************************************

LA2093      ACACTTTTCAAAATAAGCAGATTTTAGAAAGATAGGCAAAGAAGATATAACAGTAGTTAT    1258
E42         ACACTTTTCAAAATAAGCAGATTTTAGAAAGATAGGCAAAGAAGATATAACAGTAGTTAT    1260
HEINZ       ACACTTTTCAAAATAAGCAGATTTTAGAAAGATAGGCAAAGAAGATATAACAGTAGTTAT    1260
            ************************************************************

LA2093      GAACCAAAAATTAGAAGAGAGAAAGAAAACAAATCCTGTAAAACAACATTCAACTATAGC    1318
E42         GAACCAAAAATTAGAAGAGAGAAAGAAAACAAATCCTGTAAAACAACATTCAACTATAGC    1320
HEINZ       GAATCAAAAATTAGAAGAGAGAAAGAAAACAAATCCTGTAAAACAACATTCAACTATAGC    1320
            *** ********************************************************

LA2093      ACGTCCAAGTTCCATCATACTTTCAGCATTATATTTCTTGCTGCTCAACAGTGTCACAAT    1378
E42         ACGTCCAAGTTCCATCATACTTTCAGCATTATATTTCTTGCTGCTCAACAGTGTCACAAT    1380
HEINZ       ACGTCCAAGTTCCATCATACTTTCAGCATTATATTTCTTGCTGCTCAACAGTGTCACAAT    1380
            ************************************************************

LA2093      AGATAATACAGGATGCAATAGAATGCACTTAGCCTTCCCTCAGCAGGGAAAAAAGGACTT    1438
E42         AGATAATACAGGATGCAATAGAATGCACTTAGCCTTCCCTCAGCAGGGAAAAAAGGACTT    1440
HEINZ       AGATAATACAGGATGCAATAGAATGCACTTAGCCTTCCCTCAGCAGGGAAAAAAGGACTT    1440
            ************************************************************

LA2093      TAAAAGCACTTTTGCACTAACATTCAAGAAGGTTGAGTTGCAATGGATGGTTCCTCTTCC    1498
E42         TAAAAGCACTTTTGCACTAACATTCAAGAAGGTTGAGTTGCAATGGATGGTTCCTCTTCC    1500
HEINZ       TAAAAGCACTTTTGCACTAACATTCAAGAAGGTTGAGTTGCAATGGATGGTTCCTCTTCC    1500
            ************************************************************

LA2093      AATTGGCAATATTTTCATGCAAACCAAAATTTAATATTTAACAAAGTTGAGATGCAATGG    1558
E42         AATTGGCAATATTTTCATGCAAACCAAAATTTAATATTTAACAAAGTTGAGATGCAATGG    1560
HEINZ       AAATGGCAATATTTTCATGCAAACCAAAATTTAACATTTAACAAAGTTGAGATGCAATGG    1560
            ** ******************************* *************************

LA2093      TTCCGTTACAAATAACATTATTTTCACATAAACGAAGTGAAACCTAAGATGGGATAAAGA    1618
E42         TTCCGTTACAAATAACATTATTTTCACATAAACGAAGTGAAACCTAAGATGGGATAAAGA    1620
HEINZ       TTCCTTTACAAATAACATTATTTTCACATAAACGAAGTGAAACCTAAGATGGGATAAAGA    1620
            **** *******************************************************

LA2093      GAGTAAAAACAAACAACATTCCCATGTTATCTTATTTTCACGAGGAGAAAGGGTAGCTTG    1678
E42         GAGTAAAAACAAACAACATTCCCATGTTATCTTATTTTCACGAGGAGAAAGGGTAGCTTG    1680
HEINZ       GAGTAAAAACAAACAACATTCCCATGTTATCTTATTTTCACGAGGAGAAAGGGTAGCTTG    1680
            ************************************************************

LA2093      AATGATTGCAATCACTGAAGAGAGAATAAGTTGACATGTCCTTACTCATCTCAATCGCTA    1738
```

```
E42         AATGATTGCAATCACTGAAGAGAGAATAAGTTGACATGTCCTTACTCATCTCAATCGCTA    1740
HEINZ       AATGATTGCAATCACTGAAGAGAGAATAAGTTGACATGTCCTTACTCATCTCAATCGCTA    1740
            ************************************************************

LA2093      AATTGTACATAATGTTTTTTTATAATTGTGGTATCCAGGTCAATTTGAGTGCACCTTGAC    1798
E42         AATTGTACATAATGTTTTTTTATAATTGTGGTATCCAGGTCAATTTGAGTGCACCTTGAC    1800
HEINZ       AATTGTACATAATGTTTTTTTATAATTGTGGTATCCAGGTCAATTTGAGTGCACCTTGAC    1800
            ************************************************************

LA2093      TAATTCCACAGAATACTTGCTACTTCCCATCTATCATAGGTGTCGGGTAAACTCTACCCA    1858
E42         TAATTCCACAGAATACTTGCTACTTCCCATCTATCATAGGTGTCGGGTAAACTCTACCCA    1860
HEINZ       TAATTCCACAGAATACTTGCTACTTCCCATCTATCATAGGTGTCGGGTAAACTCTACCCA    1860
            ************************************************************

LA2093      CAAAGACTTGGACAAATTAGAAGGATATCACCTAGTGTCTTTGCCTTTCAGATTTGAATC    1918
E42         CAAAGACTTGGACAAATTAGAAGGATATCACCTAGTGTCTTTGCCTTTCAGATTTGAATC    1920
HEINZ       CAAAGACTTGGACAAATTAGAAGGATATCACCTAGTGTCTTTGCCTTTCAGATTTGAATC    1920
            ************************************************************

LA2093      TGAGACTCATGGTTCTAAACGTACTTCATTGACCACTTGTCCACACAATTAGGTGCGATT    1978
E42         TGAGACTCATGGTTCTAAACGTACTTCATTGACCACTTGTCCACACAATTAGGTGCGATT    1980
HEINZ       TGAGACTCATGGTTCTAAACGTACTTCATTGACCACTTGTCCACACAATTAGGTGCGATT    1980
            ************************************************************

LA2093      ATCCATAATTTATTCTTGACCAAATTCACATTCCACGGTCGAGTTGGAATCCACTTCCTC    2038
E42         ATCCATAATTTATTCTTGACCAAATTCACATTCCACGGTCGAGTTGGAATCCACTTCCTC    2040
HEINZ       ATCCATAATTTATTCTTGACCAAATTCACATTCCACGGTCGAGTTGGAATCCACTTCCTC    2040
            ************************************************************

LA2093      TGGAATGTTCCATTTAGTGTTGTAACAGTAATCAGAATAGAATCACCCCAGAGGAACTCA    2098
E42         TGGAATGCTCCATTTAGTGTTGTAACAGTAATCAGAATAGAATCACCCCAGAGGAACTCA    2100
HEINZ       TGGAATGCTCAATTTAGTGTTGTAACAGTAATCAGAATAGAATCACCCCAGAGGAACTCA    2100
            ******* ** *************************************************

LA2093      AGAAGATAAAATATGAATATAAACAAGATATAAGTCAAACTTTACGGATATTCCCCATTA    2158
E42         AGAAGATAAAATATGAATATAAACAAGATATAAGTCAAACTTTACGGATATTCCCCATTA    2160
HEINZ       AGAAGATAAAATATGAATATAAACAAGATATAAGTCAAACTTTACGGATATTCCCCATTA    2160
            ************************************************************

LA2093      CATTTCCTCACATATTGAGTTTCTAGAGATGGATCAACCTTTGATAATCTAGAAGTTGGT    2218
E42         CATTTCCTCACATATTGAGTTTCTAGAGATGGATCAACCTTTGATAATCTAGAAGTTGGT    2220
HEINZ       CATTTCCTCACATATTGAGTTTCTAGAGATGGATCAACCTTTGATAATCTAGAAGTTGGT    2220
            ************************************************************

LA2093      CATTTTATTGGCTGTATGTTATATGTATATATAATATAATATGAGTGTGTAGAGAGA--G    2276
E42         CATTTTATTGGCTGTATGTTATATGTATATATAATATAATATGAGTGTGTAGAGAGAGAG    2280
HEINZ       CATTTTATTGGCTGTATGTTATATGTATATATAATATAATATGAGTGTGTAGAGAGAGAG    2280
            *********************************************************  *

LA2093      AGAGAGAATAGAAAAGAGAATTCTGGATAAAAGTACTGTTGTAGTTGCGAGAAATAGGGA    2336
E42         AGAGAGAATAGAAAAGAGAATTCTGGATAAAAGTACTGTTGTAGTTGCGAGAAATAGGGA    2340
HEINZ       AGAGAGAATAGAAGAGAGAATTCTGGATAAAAGTACTGTTGTAGTTGCGAGAAATAGGGA    2340
            ************* **********************************************

LA2093      AGCGATGGAAGCTAGATGACACAGAAGTTCACATAACATAATACTCGTGTTTATCAAATA    2396
E42         AGCGATGGAAGCTAGATGACACAGAAGTTCACATAACATAATACTCGTGTTTATCAAATA    2400
HEINZ       AGCGATGGAAGCTAGATGACACAGAAGTTCACATAACATAATACTCGTGTTTATCAAATA    2400
            ************************************************************

LA2093      ATTAGCATTGATCAAAGCAAAAAGAAAAATTCTTCAGCAGAGAGAAGAGTGGAAAGAGCC    2456
E42         ATTAGCATTGATCAAAGCAAAAAGAAAAATTCTTCAGCAGAGAGAAGAGTGGAAAGAGCC    2460
HEINZ       ATTAGCATTGATCAAAGCAAAAAGAAAAATTCTTCAGCAGAGAGAAGAGTGGAAAGAGCC    2460
            ************************************************************

LA2093      AGAAGAAATGAGGCTGACCTTAAGCTACCCCAAAGTTTTGAAGCCTGTCACAATGAGGTG    2516
E42         AGAAGAAATGAGGCTGACCTTAAGCTACCCCAAAGTTTTGAAGCCTGTCACAATGAGGTG    2520
HEINZ       AGAAGAAATGAGGCTGACCTTAAGCTACCCCAAAGTTTTGAAGCCTGTCACAATGAGGTG    2520
            ************************************************************

LA2093      TTGGGTAATTCCTGTTCCATTGCCGCCTCTGGAGTTCTATGTGACAGTAACGACCAAGTG    2576
E42         TTGGGTAATTCCTGTTCCATTGCCGCCTCTGGAGTTCTATGTGACAGTAACGACCAAGTG    2580
HEINZ       TTGGGTAATTCCTGTTCCATTGCCGCCTCTGGAGTTCTATGTGACAGTAACGACCAAGTG    2580
```

```
             ************************************************************

LA2093       TACTTTTGTGATCTCTAATAACATTGTCTTCAATGTTCTCCCGAGCAGGACTACCAAGAG    2636
E42          TACTTTTGTGATCTCTAATAACATTGTCTTCAATGTTCTCCCGAGCAGGACTACCAAGAG    2640
HEINZ        TACTTTTGTGATCTCTAATAACATTGTCTTCAATGTTCTCCCGAGCAGGACTACCAAGAG    2640
             ************************************************************

LA2093       GATAATTCTTATCGAATTCTGCTGATTTTACGAAAAATTCTACACCATATTTATCTGTCA    2696
E42          GATAATTCTTATCGAATTCTGCTGATTTTACGAAAAATTCTACACCATATTTATCTGTCA    2700
HEINZ        GATAATTCTTATAGAATTCTGCTGATTTTACGAAAAATTCTACACCATATTTATCTGTCA    2700
             ************ ***********************************************

LA2093       TCTTCTTAAACTGATAAGAGTAGTTCCTCTGCAAGGAATATTGTTGAGGCGACGAGAAAG    2756
E42          TCTTCTTAAACTGATAAGAGTAGTTCCTCTGCAAGGAATATTGTTGAGGCGACGAGAAAG    2760
HEINZ        TCTTCTTAAACTGATAAGAGTAGTTCCTCTGCAAGGAATATTGTTGAGGCGACGAGAAAG    2760
             ************************************************************

LA2093       GAAAATAAGCAAGTAAAAAGATGATAATAAATGGTAACATTTGAAGGAGGAGAATTAAAT    2816
E42          GAAAATAAGCAAGTAAAAAGATGATAATAAATGGTAACATTTGAAGGAGGAGAATTAAAT    2820
HEINZ        GAAAATAAGCAAGTAAAAAGATGATAATAAATGGTAACATTTGAAGGAGGAGAATTAAAT    2820
             ************************************************************

LA2093       TAGGTCCAGAGGAACCTAAATTCTGCCTCTGGTGGCGGACATTGGTCCTTGACCTGTAGA    2876
E42          TAGGTCCAGAGGAACCTAAATTCTGCCTCTGGTGGCGGACATTGGTCCTTGACCTGTAGA    2880
HEINZ        TAGGTCCAGAGGAACCTAAATTCTGCCTCTGGTGGCGGACATTGGTCCTTGACCTGTAGA    2880
             ************************************************************

LA2093       CATAAGAGGTCCTAAACATTTCCGACTGACCAAAAAATGCCCTTATAATCTCATCATCAT    2936
E42          CATAAGAGGTCCTAAACATTTCCGACTGACCAAAAAATGCCCTTATAATCTCATCATCAT    2940
HEINZ        CATAAGAGGTCCTAAACATTTCCGACTGACCAAAAAATGCCCTTATAATCTCATCATCAT    2940
             ************************************************************

LA2093       CAAAATCATCTTCAAAAAATTCGTGGTCCATTCTTCTCCTTCGACGTCTAAGATTATACT    2996
E42          CAAAATCATCTTCAAAAAATTCGTGGTCCATTCTTCTCCTTCGACGTCTAAGATTATACT    3000
HEINZ        CAAAATCATCTTCAAAAAATTCGTGGTCCATTCTTCTCCTTCGACGTCTAAGATTATACT    3000
             ************************************************************

LA2093       GCTGATTAAACTCAAATTCATCCACCAAACCAGTCTCATCATACTGCCTTCTTGAATCAT    3056
E42          GCTGATTAAACTCAAATTCATCCACCAAACCAGTCTCATCATACTGCCTTCTTGAATCAT    3060
HEINZ        GCTGATTAAACTCAAATTCATCCACCAAACCAGTCTCATCATACTGCCTTCTTGAATCAT    3060
             ************************************************************

LA2093       CATCACTCAAACACTTAAATGCCTTGGATACTTTCTTAAAGGCCTCTTCAGATCCAGGAG    3116
E42          CATCACTCAAACACTTAAATGCCTTGGATACTTTCTTAAAGGCCTCTTCAGATCCAGGAG    3120
HEINZ        CATCACTCAAACACTTAAATGCCTTGGATACTTTCTTAAAGGCCTCTTCAGATCCAGGAG    3120
             ************************************************************

LA2093       CCTTGTTTTTGTCGGGATGAACTTTCAAGGAGAGCTTCCTGTATGCCTTCCTTATCTCCT    3176
E42          CCTTGTTTTTGTCGGGATGAACTTTCAAGGAGAGCTTCCTGTATGCCTTCCTTATCTCCT    3180
HEINZ        CCTTGTTTTTGTCGGGATGAACTTTCAAGGAGAGCTTCCTGTATGCCTTCCTTATCTCCT    3180
             ************************************************************

LA2093       CAACTGAACAGCTCTTTTCTAGACCTAGAATTGCATAATAGTCCTTTATGCTCTTAATTT    3236
E42          CAACTGAACAGCTCTTTTCTAGACCTAGAATTGCATAATAGTCCTTTATGCTCTTAATTT    3240
HEINZ        CAACTGAACAGCTCTTTTCTAGACCTAGAATTGCATAATAGTCCTTTATGCTCTTAATTT    3240
             ************************************************************

LA2093       GTGTAACCAAATGCACGTGTTCTTCAGTATAGTTCCTCTCCTCATCAGATTTGACATGTT    3296
E42          GTGTAACCAAATGCACGTGTTCTTCAGTATAGTTCCTCTCCTCATCAGATTTGACATGTT    3300
HEINZ        GTGTAACCAAATGCACGTGTTCTTCAGTATAGTTCCTCTCCTCATCAGATTTGACATGTT    3300
             ************************************************************

LA2093       TCTCACTCGAGACATCTCCGATCTCACTAGGGTGATCATAAGTTGAGGCATCAAGCTTTT    3356
E42          TCTCACTCGAGACATCTCCGATCTCACTAGGGTGATCATAAGTTGAGGCATCAAGCTTTT    3360
HEINZ        TCTCACTCGAGACATCTCCGATCTCACTAGGGTGATCATAAGTTGAGGCATCAAGCTTTT    3360
             ************************************************************

LA2093       CACAAGCAACCAAAAGATCATCGACGTCTAGTTTACTGTCAAGGCGGCATGCGATTCCAA    3416
E42          CACAAGCAACCAAAAGATCATCGACGTCTAGTTTACTGTCAAGGCGGCATGCGATTCCAA    3420
HEINZ        CACAAGCAACCAAAAGATCATCGACGTCTAGTTTACTGTCAAGGCGGCATGCGATTCCAA    3420
             ************************************************************
```

```
LA2093      TATACTTAAGTGCTTTCTTCTTATTGCCTGACACAATTGCCTCCTTAGCAATACTGATGC    3476
E42         TATACTTAAGTGCTTTCTTCTTATTGCCTGACACAATTGCCTCCTTAGCAATACTGATGC    3480
HEINZ       TATACTTAAGTGCTTTCTTCTTATTGCCTGACACAATTGCCTCCTTAGCAATACTGATGC    3480
            ************************************************************

LA2093      ATCTTAAGGCCTCATCTTTATTACCATCCATTCTTATCTCAACTTCAACTATTATCCAAA    3536
E42         ATCTTAAGGCCTCATCTTTATTACCATCCATTCTTATCTCAACTTCAACTATTATCCAAA    3540
HEINZ       ATCTTAAGGCCTCATCTTTATTACCATCCATTCTTATCTCAACTTCAATTATTATCCAAA    3540
            ************************************************ ***********

LA2093      AAAGATGAAAATTAAGCTTAAAAGCTAACATCCATTGCACAAGAATACATAATGCCAACA    3596
E42         AAAGATGAAAATTAAGCTTAAAAGCTAACATCCATTGCACAAGAATACATAATGCCAACA    3600
HEINZ       AAAGATGAAAATTAAGCTTAAAAGCTAACATCCATTGCACAAGAATACATAATGCCAACA    3600
            ************************************************************

LA2093      GTCATTCAGAATCTTGTTTCGCAATTCAATGTCACCTGACCACCAAAATAGCAAAACCAA    3656
E42         GTCATTCAGAATCTTGTTTCGCAATTCAATGTCACCTGACCACCAAAATAGCAAAACCAA    3660
HEINZ       GTCATTCAGAATCTTGTTTCGCAATTCAATTTCACCTGACCACCAAAATAGCAAAACCAA    3660
            ****************************** *****************************

LA2093      TCACTTATAAT        3667
E42         TCACTTATAAT        3671
HEINZ       TCACTTATAAT        3671
            ***********
```

Solyc01g067780

```
E42          TGGACTGCAACAAAGAAGAGGCCATTAAGGCTAGAGGGATGGCTGAAGTTATGATGCGAA     60
HEINZ        TGGACTGCAACAAAGAAGAGGCCATTAAGGCTAGAGGGATGGCTGAAGTTATGATGCGAA     60
LA2093       TGGACTGCAACAAAGAAGAGGCCATTAAGGCTAGAGGGATGGCTGAAGTTATGATGCGAA     60
             ************************************************************

E42          ATAGAGATTTCGTTGGAGCTAAAAAGTTTGCATCTAAAGCCCAGAAGCTCTTTCCTGATC     120
HEINZ        ATAGAGATTTCGTTGGAGCTAAAAAGTTTGCATCTAAAGCCCAGAAGCTCTTTCCTGATC     120
LA2093       ATAGAGATTTCGTTGGAGCTAAAAAGTTTGCATCTAAAGCCCAGAAGCTCTTTCCTGATC     120
             ************************************************************

E42          TGGAGAACATTACACAGATGGTTTCGATTTGTGAGGTGCATTGCTCTGCAGAGAAGACGT     180
HEINZ        TGGAGAACATTACACAGATGGTTTCGATTTGTGAGGTGCATTGCTCTGCAGAGAAGACGT     180
LA2093       TGGAGAACATTACACAGATGGTTTCGATTTGTGAGGTGCATTGCTCTGCAGAGAAGACGT     180
             ************************************************************

E42          CTTTCGGAAATGAAAAAGATTGGTATAGTATTCTTAAAGTAGAGCCAACAGCTGATGATG     240
HEINZ        CTTTCGGAAATGAAAAAGATTGGTATAGTATTCTTAAAGTAGAGCCAACAGCTGATGATG     240
LA2093       CTTTCGGAAATGAAAAAGATTGGTATAGTATTCTTAAAGTAGAGCCAACAGCTGATGATG     240
             ************************************************************

E42          CTCTTATCAGGAAGCAATATCGCAAGCTTGCTCTCTTGCTACATCCTGATAAGAACAAGT     300
HEINZ        CTCTTATCAGGAAGCAATATCGCAAGTTTGCTCTCTTGCTACATCCTGATAAGAACAAGT     300
LA2093       CTCTTATCAGGAAGCAATATCGCAAGTTTGCTCTCTTGCTACATCCTGATAAGAACAAGT     300
             ************************** *********************************

E42          TCCCTGGTGCAGCTGATGCCTTTTCACTGATTGGTGAAGCTATATCGGTGCTTTTGGATG     360
HEINZ        TCCCTGGTGCAGCTGATGCCTTTTCACTGATTGGTGAAGCTATATCGGTGCTTTTGGATG     360
LA2093       TCCCTGGTGCAGCTGATGCCTTTTCACTGATTGGTGAAGCTATATCGGTGCTTTTGGATG     360
             ************************************************************

E42          GACCGAAACGAATGTTGTACAACAGTAGACACATTCCTTCAGGGAGATTTCAAGTACCAA     420
HEINZ        GACCGAAACGAACGTTGTACAACAGTAGACACATTCCTTCAGGGAGATTTCAAGTACCAA     420
LA2093       GACCGAAACGAACGTTGTACAACAGTAGACACATTCCTTCAGGGAGATTTCAAGTACCAA     420
             ************ ***********************************************

E42          TGCAGCATAAGAGCTGCCAGCCAGATACCAAGAAACATCACAAGGTAACTCAATCAGGAG     480
HEINZ        TGCAGCATAAGAGCTGCCAGCCAGATACCAGGAAACATCACAAGGTAACTCAATCAGGAG     480
LA2093       TGCAGCATAAGAGCTGCCAGCCAGATACCAGGAAACATCACAAGGTAACTCAATCAGGAG     480
             ****************************** *****************************

E42          CTCCAGAAAGCGAACCAACCTATTGGACTATCTGCCCATGCTGTTCTATTAAGTACAAGT     540
HEINZ        CTCCAGAAAGCGAACCAACCTTTTGGACTATCTGCCCATGCTGTTCTATTAAGTACAAGT     540
LA2093       CTCCAGAAAGCGAACCAACCTTTTGGACTATCTGCCCATGCTGTTCTATTAAGTACAAGT     540
             ********************* **************************************

E42          ATCATAAGACATTTCTAAATCAATTGTTGCGGTGCCCCAATAGTAAGAAGTCGTACAGAG     600
HEINZ        ATCATAAGACATTTCTAAATCAATTGTTGCGGTGCCCCAATAGTAAGAAGTCGTACAGAG     600
LA2093       ATCATAAGACATTTCTAAATCAATTGTTGCGGTGCCCCAATAGTAAGAAGTCGTACAGAG     600
             ************************************************************

E42          GTTATGAAGTGAATGATTCAGTTGCAACACCCGGAACCAGTAGGAGTCAGCCTACTTCCA     660
HEINZ        GTTATGAAGTGAATGATTCAGTTGCAACACCCGGAACCAGTAGGAGTCAGCCTACTTCCA     660
LA2093       GTTATGAAGTGAATGATTCAGTTGCAACACCCGGAACCAGTAGGAGTCAGCCTACTTCCA     660
             ************************************************************

E42          GTCAGAAGAAGGGTGCTGATGAGACCCTAGCCAGGAATTCATTTATACAGCCAGAGTTTC     720
HEINZ        GTCAGAAGAAGGGTGCTGATGAGACCCTAGCCAGGAATTCATTTATACAGCCAGAGTTTC     720
LA2093       GTCAGAAGAAGGGTGCTGATGAGACCCTAGCCAGGAATTCATTTATACAGCCAGAGTTTC     720
             ************************************************************

E42          CCTCAGAGGTTAGTCAGGAGTCTAATAGAAATGGAAAGTCGGAAAATGCATATAGAAAAA     780
HEINZ        CCTCAGAGGTTAGTCAGGAGTCTAATAGAAATGGAAAGTCGGATAATGCATATAGAAAAA     780
LA2093       CCTCAGAGGTTAGTCAGGAGTCTAATAGAAATGGAAAGTCGGAAAATGCATATAGAAAAA     780
             ******************************************* ****************

E42          TGAATAAGGAGGGATTATCAGGAGAATATAAAAGGAAAAACACAGAGAGAAAGAAGACAT     840
HEINZ        TGAATAAGGAGGGATTATCAGGGGAATATAAAAGGAAAAACACAGAGAGAAAGAAGATAT     840
LA2093       TGAATAAGAAGGGATTATCAGGGGAATATAAAAGGAAAAACACAGAGAGAAAGAAGACAT     840
             ******** ************* *********************************** **
```

```
E42         CAATAGAATCTAGTGAAAAATGTGATTTATCAGAAGATACAAATTTTGAAGTGGACACTC    900
HEINZ       CAATAGAATCTAGTGAAAAATGTGATTTATCAGAAGATACAAATTTTGAAGTGGACACTC    900
LA2093      CAATAGAATCTAGTGAAAAATGTGATTTATCAGAAGATACAAATTTTGAAGTGGACACTC    900
            ************************************************************

E42         ATGTTCCTGGGCAGAAATCTCAATGTCTCACTAGGGAAAACCAGCGAAGATCTACACGGT    960
HEINZ       ATGTTCCTGGGCAGAAATCTCAATGTCTCACTAGGGAAAACCAGCGAAGATCTACACGGT    960
LA2093      ATGTTCCTGGGCAGAAATCTCAATGTCTCACTAGGGAAAACCAGCGAAGATCTACACGGT    960
            ************************************************************

E42         GTAGACAGCATGTCACTCACCGTGATAATCTTAGTGATGAAGATGAAGAAGAAGGTCCTT    1020
HEINZ       GTAGACAGCATGTCACTCACCGTGATAATCTTAGTGATGAAGATGAAGAAGAAGGTCCTT    1020
LA2093      GTAGACAGCATGTCACTCACCGTGATAATCTTAGTGATGAAGATGAAGAAGAAGGTCCTT    1020
            ************************************************************

E42         CTAAGCGATCTAAGGGAGTTGGATATCCCTCTCCTGCTAAGGAATCCAAGGTTCAGCATT    1080
HEINZ       CTAAGCGATCTAAGGGAGTTGGATATCCCTCTCCTACTAAGGAATCCGAGGTTCAGCATT    1080
LA2093      CTAAGCGATCTAAGGGAGTTGGATATCCCTCTCCTACTAAGGAATCCGAGGTTCAGCATT    1080
            *********************************** *********** ************

E42         TGTCTCATGCAGCAACTCCTAAGGGGAAGGAGAAGAAATTGAAGGACAGTTTGTCCTCTG    1140
HEINZ       TGTCTCATGCAGCAACTCCTAAGGGGAAGGAGAAGAAATTGAAGGACAGTTTGTCCTCTG    1140
LA2093      TGTCTCATGCAGCAACTCCTAAGGGGAAGGAGAAGAAATTGAAGGACAGTTTGTCCTCTG    1140
            ************************************************************

E42         AAGAGCGCTTGCAGAACACAGAGCAGGAAGCTGAGACTGCAAATGGAAGAGTGGATCTAC    1200
HEINZ       AAGAGCGCTTGCAGAACACAGAGCAGGAAGCTGAGACTGCAAATGGAAGAGTGGATCTAC    1200
LA2093      AAGAGCGCTTGCAGAACACAGAGCAGGAAGCTGAGACTGCAAATGGAAGAGTGGATCTAC    1200
            ************************************************************

E42         ATTTAAAAGGAAGTGTTGATTGTCCCTCAGATGTAGGGGCTTCTGCTATGGCTGAGCCAA    1260
HEINZ       CTTTAAAAGGAAGTGTTGATTGTCCCTCAGATGTAGGGGCTTCTGCTATGGCTGAGCCAA    1260
LA2093      CTTTAAAAGGAAGTGTTGATTGTCCCTCAGATGTAGGGGCTTCTGCTATGGCTGAGCCAA    1260
             ***********************************************************

E42         AAATATATCAATGTGCAGATCCAGATTTTAGTGATTTTGACAAGGACAAGGAAGAATCTT    1320
HEINZ       AAATATATCAATGTGCAGATCCAGATTTTAGTGATTTTGACAAGGACAAGGAAGAATCTT    1320
LA2093      AAATATATCAATGTGCAGATCCAGATTTTAGTGATTTTGACAAGGACAAGGAAGAATCTT    1320
            ************************************************************

E42         GCTTTAAGGTTGGACAGGTATGGGCTATTTATGATTCTCTAGACGCCATGCCTAGATTTT    1380
HEINZ       GCTTTAAGGTTGGACAGGTATGGGCTATTTATGATTCTCTAGACGCCATGCCTAGATTTT    1380
LA2093      GCTTTAAGGTTGGACAGGTATGGGCTATTTATGATTCTCTAGACGCCATGCCTAGATTTT    1380
            ************************************************************

E42         ATGCGGTCATCAGCAAGATCGTATCTCCTGCATTTAAGTTGAGCATAACTTGGTTAGAGC    1440
HEINZ       ATGCGGTCATCAGCAAGATTGTATCTCCTGCATTTAAGTTGAGCATAACTTGGTTAGAGC    1440
LA2093      ATGCGGTCATCAGCAAGATTGTATCTCCTGCATTTAAGTTGAGCATAACTTGGTTAGAGC    1440
            ******************* ****************************************

E42         CAGATCCGCTGAATGAAGATGAAACCAAATGGCTGTCTGAGGGCCTTCCAGCTTCTTGTG    1500
HEINZ       CAGATCCGCTGAATGAAGATGAAACCAAATGGCTGTCTGAGGGCCTTCCAGCTTCTTGTG    1500
LA2093      CAGACCCGCTGAATGAAGATGAAACCAAATGGCTGTCTGAGGGCCTTCCAGCTTCTTGTG    1500
            **** *******************************************************

E42         GTAGGTTCAGAAAGGGAAACTTGGAAGACATTGAAGATCTTCCCATGTTCTCCCATTTGG    1560
HEINZ       GTAGGTTCAGAAAGGGAAACTTGGAAGACATTGAAGATCTTCCCATGTTCTCCCATTTGG    1560
LA2093      GTAGGTTCAGAAAGGGAAACTTGGAAGACATTGAAGATCTTCCCATGTTCTCCCATTTGG    1560
            ************************************************************

E42         TATGTGCAATAAATAGACATAGCTGTGGTGCCATAAAGATATTTCCACTGCAAGGAGAAA    1620
HEINZ       TATGTGCAATAAATAGACATAGCTGTGGTGCCATAAAGATATTTCCACTGCAAGGAGAAA    1620
LA2093      TATGTGCAATAAATAGACATAGCTGTGGTGCCATAAAGATATTTCCACTGCAAGGAGAAA    1620
            ************************************************************

E42         CTTGGGCCATCTTTAGAGATTGGGATTTAAACTGGTGTTCTGGTCTTGAGAGGAAGAAGA    1680
HEINZ       CTTGGGCCATCTTTAGAGATTGGGATTTAAACTGGTGTTCTGGTCTTGAGAGGAAGAAGA    1680
LA2093      CTTGGGCCATCTTTAGAGATTGGGATTTAAACTGGTGTTCTGGTCTTGAGAGGAAGAAGA    1680
            ************************************************************

E42         AGTTCAAGTATGATTTTGTTGAGGTCTTGTCAGACTTTGCTGATGCTATTGGTGTGCATG    1740
```

```
HEINZ       AGTTCAAGTATGATTTTGTTGAGGTCTTGTCAGACTTTGCTGATGCTATTGGTGTGCATG    1740
LA2093      AGTTCAAGTATGATTTTGTTGAGGTCTTGTCAGACTTTGCTGATGCTATTGGTGTGCATG    1740
            ************************************************************

E42         TTGTAAAGTTGGTGAAAGCAAATCGTTTCACTTGTCTTTTTCATCGAGCAGGACACCCAT    1800
HEINZ       TTGTAAAGTTGGTGAAAGCAAATGGTTTCACTTGTCTTTTTCATCGAGCAGGACACCCAT    1800
LA2093      TTGTAAAGTTGGTGAAAGCAAATCGTTTCACTTGTCTTTTTCATCGAGCAGGACACCCAT    1800
            *********************** ************************************

E42         TTGTAGTTCCTGCAAAGGAGATGCTTAGATTTTCTCATAGAGTTCCTTCTTTTAAGATGA    1860
HEINZ       TTGTAGTTCCTGCAAAGGAGATGCTTAGATTTTCTCATAGAGTTCCTTCTTTTAAGATGA    1860
LA2093      TTGTAGTTCCTGCAAAGGAGATGCTTAGATTTTCTCATAGAGTTCCTTCTTTTAAGATGA    1860
            ************************************************************

E42         CAGGGATGGAGAGGAATGATGTTCCTGAAGGATCTTTTGAACTAGACCCTGCTTCCTTAC    1920
HEINZ       CAGGGATGGAGAGGAATGATGTTCCTGAAGGATCTTTTGAACTAGACCCTGCTTCCTTAC    1920
LA2093      CAGGGATGGAGAGGAATGATGTTCCTGAAGGATCTTTTGAACTAGACCCTGCTTCCTTAC    1920
            ************************************************************

E42         CTACTGACCAAGTAGGCGTTTCTGCTTCATCTCTAGACGAGAGAGAGAGAGGTAATTTCA    1980
HEINZ       CTACTGACCAAGTAGGCATTTCTGCTTCATCTCTAGACGAGAGAGAGAGAGGTAATTTCA    1980
LA2093      CTACTGACCAAGTAGGCATTTCTGCTTCATCTCTAGACGAGAGAGAGAGAGGTAATTTCA    1980
            ***************** ******************************************

E42         TGGCATACGATCACATGGATTCTGCAGAAAAGTGTGTTGGATCAGTACCTAATCAGGTTG    2040
HEINZ       TGGCATACGATCACATGGATTCTGCAGAAAAGTGTGTTGGATCAGTACCTGATCAGGTTG    2040
LA2093      TGGCATACGATCACATGGATTCTGCAGAAAAGTGTGTTGGATCAGTACCTGATCAGGTTG    2040
            ************************************************** *********

E42         CTGAACCTATATTCTACTGCTTTGATGCTGAGAGATCACCGGAGAAGTTTGAAGTTGGTC    2100
HEINZ       CTGAACCTATATTCTACTGCTTTGATGCTGAGAGATCACCGGAGAAGTTTGAAGTTGGTC    2100
LA2093      CTGAACCTATATTCTACTGCTTTGATGCTGAGAGATCACCGGAGAAGTTTGAAGTTGGTC    2100
            ************************************************************

E42         AATATTGGGCGATGTACAGTGATGAAGACGGCTTACCCAGGTACTATGGGCTGATAAAGA    2160
HEINZ       AATATTGGGCGATGTACAGTGATGAAGACGGCTTACCCAGGTACTATGGGCTGATAAAGA    2160
LA2093      AATATTGGGCGATGTACAGTGATGAAGACGGCTTACCCAGGTACTATGGGCTGATAAAGA    2160
            ************************************************************

E42         AGATAGATCTTCTCCCTGATTTTGTGTTGCATGTGGCATGGCTGTATGCCTGTCCACCCC    2220
HEINZ       AGATAGATCTTCTCCCTGATTTTGTGTTGCATGTGGCATGGCTGTATGCCTGTCCACCCC    2220
LA2093      AGATAGATCTTCTCCCTGATTTTGTGTTGCATGTGGCATGGCTGTATGCCTGTCCACCCC    2220
            ************************************************************

E42         CTAAAGGTACAACACAGTGGCATGATGAAACAATGCCGATTGGGTGTGGACAGTTCAAGT    2280
HEINZ       CTAAAGGTACAACACAGTGGCATGATGAAACAATGCCGATTGGGTGTGGACAGTTCAAGT    2280
LA2093      CTAAAGGCACAACACAGTGGCATGATGAAACAATGCCGATTGGGTGTGGACAGTTCAAGT    2280
            ******* ****************************************************

E42         TTCGAAACAGTAAGCTGAAGCCATATACTGGAACCGCCACCTTTTCACATGAAGTAGCAG    2340
HEINZ       TTCGAAACAGTAAGCTGAAGCCATATACTGGAACCGCCACCTTTTCACATGAAGTAGCAG    2340
LA2093      TTCGAAACAGTAAGCTGAAGCCATATACTGGAACCGCCACCTTTTCACATGAAGTAGCAG    2340
            ************************************************************

E42         CAGAGGTTTTGAAGAAGGGTTTATACAAGATCTTCCCGGGAAAAGGCGAAGTTTGGGCAG    2400
HEINZ       CAGAGGTTTTGAAGAAGGGTTTATACAAGATCTTCCCGGGAAAAGGCGAAGTTTGGGCAG    2400
LA2093      CAGAGGTTTTGAAGAAGGGTTTATACAAGATCTTCCCGGGAAAAGGCGAAGTTTGGGCAG    2400
            ************************************************************

E42         TGTACAAGAACTGGAGCGCTAAGACAAAAGGTAAGAAACTAGAAGATTGCGAGTATGAGA    2460
HEINZ       TGTACAAGAACTGGAGCGCTAAGATAAAAGGTAAGAAACTAGAAGATTGCGAGTATGAGA    2460
LA2093      TGTACAAGAACTGGAGCGCTAAGATAAAAGGTAAGAAACTAGAAGATTGCGAGTATGAGA    2460
            ************************ ***********************************

E42         TTGTTGAAATAGTGGACGTTTCCACTAGCTATATACAAGTGAAGTTGTTAGTGAGGGTAC    2520
HEINZ       TTGTTGAAATAGTGGACGTTTCCACTAGCTATATACAAGTGAAGTTGTTAGTGAGGGTAC    2520
LA2093      TTGTTGAAATAGTGGACGTTTCCACTAGCTATATACAAGTGAAGTTGTTAGTGAGGGTAC    2520
            ************************************************************

E42         AAGGCTTCAAGTCTGTCTACAAGCCTCAAGTGGAAGAAGAAGGAAGAGTGAAAATATCTA    2580
HEINZ       AAGGCTTCAAGTCTGTCTACAAGCCTCAAGTGGAAGAAGAAGGAAGAGTGAAAATATCTA    2580
LA2093      AAGGCTTCAAGTCTGTCTACAAGCCTCAAGTGGAAGAAGAAGGAAGAGTGAAAATATCTA    2580
```

```
            ************************************************************

E42         TGTCCGAGCATCTCAAGTTCTCTCATGGAATCCCTGCTTTTCGCCTGACAGAAGAGAGAG    2640
HEINZ       TGTCCGACCATCTCAAGTTCTCTCATGGAATCCCTGCTTTTCGCCTGACAGAAGAGAGAG    2640
LA2093      TGTCCGACCATCTCAAGTTCTCTCATGGAATCCCTGCTTTTCGCCTGACAGAAGAGAGAG    2640
            ******* ****************************************************

E42         GTGGCAGTCTTCGAGGTTTCTGGGAGCTTGATCCAGCCGCAATGCCGCTTTATTTACTGT    2700
HEINZ       GTGGCAGTCTTCGAGGTTTCTGGGAGCTTGATCCAGCCGCAATGCCGCTTTATTTACTGT    2700
LA2093      GTGGCAGTCTTCGAGGTTTCTGGGAGCTTGATCCAGCCGCAATGCCGCTTTATTTACTGT    2700
            ************************************************************

E42         GCACAGATTGA        2711
HEINZ       GCACAGATTGA        2711
LA2093      GCACAGATTGA        2711
            ***********
```

Solyc01g079610

```
LA2093      TTGAAACCAACATTTCCATATGCCATTATAGAGTAAACCATCTTAAAATAGAGCAGCAGA    60
E42         TTGAAACCAACATTTCCATATGCCATTATAGAGTAAACCATCTTAAAATAGAGCAGCAGA    60
HEINZ       TTGAAACCAACATTTCCATATGCCATTATAGAGTAAACCATCTTAAAATAGAGCAGCAGA    60
            ************************************************************

LA2093      CAATAGGCTACTCAGTCATCCAAATATATGTGAATCACTGGGCTAGTAATATACATATTT    120
E42         CAATAGGCTACTCAGTCATCCAAATATATGTGAATCACTGGGCTAGTAATATACATATTT    120
HEINZ       CAATAGGCTACTCAGTCATCCAAATATATGTGAATCACTGGGCTAGTAATATACATATTT    120
            ************************************************************

LA2093      CCATCTACTCTATGAATCATTGTTCAATACATGTAAATGGGTTCTATTCTATAATCCAAA    180
E42         CCATCTACTCTATGAATCATTGTTCAATACATGTAAATGGGTTCTATTCTATAATCCAAA    180
HEINZ       CCATCTACTCTATGAATCATTGTTCAATACATGTAAATGGGTTCTATTCTATAATCCAAA    180
            ************************************************************

LA2093      TGAGAACCTCCACCCAAAAATTGAGAAAAAAGGAAATATGAATTTGGTACAAGGGCCTTG    240
E42         TGAGAACCTCCACCCAAAAATTGAGAAAAAAGGAAATATGAATTTGGTACAAGGGCCTTG    240
HEINZ       TGAGAACCTCCACCCAAAAATTGAGAAAAAAGGAAATATGAATTTGGTACAAGGGCCTTG    240
            ************************************************************

LA2093      AAA-AAAAACAACGCAGACGCCTGGAAGATTATACCAAGTCTGACGTACAACGCACCTTC    299
E42         AAAAAAAAACAACGCAGACGCCTGGAAGATTATACCAAGTCTGACGTACAACGCACCTTC    300
HEINZ       AAA-AAAAACAACGCAGACGCCTGGAAGATTATACCAAGTCTGACGTACAACGCACCTTC    299
            *** ********************************************************

LA2093      AAAGCAGATGTCCAGAATTCATTACTTGGGACCAAATGATATACTGTATCCTAACCAAGA    359
E42         AAAGCAGATGTCCAGAATTCATTACTTGGGACCAAATGATATACTGTATCCTAACCAAGA    360
HEINZ       AAAGCAGATGTCCAGAATTCATTACTTGGGACCAAATGATATACTGTATCCTAACCAAGA    359
            ************************************************************

LA2093      ACCTCCTTGATCTGTTTCTTTTGGTCCTCCGTCAATGACGTGGGGAAAAGAACCTCAAAT    419
E42         ACCTCCTTGATCTGTTTCTTTTGGTCCTCCGTCAATGACGTGGGGAAAAGAACCTCAAAT    420
HEINZ       ACCTCCTTGATCTGTTTCTTTTGGTCCTCCGTCAATGACGTGGGGAAAAGAACCTCAAAT    419
            ************************************************************

LA2093      TTAACATATAAATCACCCTTCTTGTTACTGTAATGTAGCGGCATCCCTTCACCTTTAAAC    479
E42         TTAACATATAAATCACCCTTCTTGTTACTGTAATGTAGCGGCATCCCTTCACCTTTAAAC    480
HEINZ       TTAACATATAAATCACCCTTCTTGTTACTGTAATGTAGCGGCATCCCTTCACCTTTAAAC    479
            ************************************************************

LA2093      TTCCTCACTTCTTTAGGTTTAGTGATTCCCTAGTGAAGAATATAAATAATGATTTCAGTA    539
E42         TTCCTCACTTCTTTAGATTTAGTGATTCCCTAGTGAAGAATATAAATAATGATTTCAGTA    540
HEINZ       TTCCTCACTTCTTTAGGTTTAGTGATTCCCTAGTGAAGAATATAAATAATGATTTCAGTA    539
            **************** *******************************************

LA2093      CACAGTTATACAGCCAAACATACACAAGTGTACTTGGTTAGCATGCCAGAAATGTACAGA    599
E42         CACAGTTATACAGCCAAACATACACAAGTGTACTTGGTTAGCATGCCAGAAATGTACAGA    600
HEINZ       CACAGTTATACAGCCAAACATACACAAGTGTACTTGGTTAGCATGCCAGAAATGTACAGA    599
            ************************************************************

LA2093      AAAATATACCTTTGAACTAATGTCCACCAGATGATCATCGAGATGTTTGATGGTCTTCTC    659
E42         AAAATATACCTTTGAACTAATGTCCACCAGATGATCATCGAGATGTTTGATGGTCTTCTC    660
HEINZ       AAAATATACCTTTGAACTAATGTCCACCAGATGATCATCGAGATGTTTGATGGTCTTCTC    659
            ************************************************************

LA2093      GAAACCAACCAGAGCTTGCACCTGGATTGAAGTACAAGCATTATATCAACAAAGGAGTTA    719
E42         GAAACCAACCAGAGCTTGCACCTGGATTGAAGTACAAGCATTATATCAACAAAGGAGTTA    720
HEINZ       GAAACCAACCAGAGCTTGCACCTGGATTGAAGTACAAGCATTATATCAACAAAGGAGTTA    719
            ************************************************************

LA2093      GCTTTCTAAGATCCACAGGAATGAAGCTTTCTATCCATATCCGTTGGTTGTTACTGATTA    779
E42         GCTTTCTAAGATCCACAGGAATGAAGCTTTCTATCCATATCCGTTGGTTGTTACTGATTA    780
HEINZ       GCTTTCTAAGATCCACAGGAATGAAGCTTTCTATCCATATCCGTTGGTTGTTACTGATTA    779
            ************************************************************

LA2093      ACCACGTCAACTTCACGAAAAAAGGTTCTGTAATCTCCAAACAGAAATCCAGGAGTTACA    839
E42         ACCACGTCAACTTCACGAAAAAAGGTTCTGTAATCTCCAAACAGAAATCCAGGAGTTACA    840
HEINZ       ACCACGTCAACTTCACGAAAAAAGGTTCTGTAATCTCCAAACAGAAATCCAGGAGTTACA    839
            ************************************************************
```

```
LA2093      GTGAAACAAAGATTGACTATCCTGATAGTGATCTCGACCCAAATAAAGGTAAAGACAATA    899
E42         GTGAAACAAAGATTGACTATCCTGATAGTGATCTCGACCCAAATAAAGGTAAAGACAATA    900
HEINZ       GTGAAACAAAGATTGACTATCCTGATAGTGATCTCGACCCAAATAAAGGTAAAGACAATA    899
            ************************************************************

LA2093      GTTTGCGTCTCAGTGACACCACTTATAATGCACCAAAGGTCTCATGCATAAGTGAAGTTT    959
E42         GTTTGCGTCTCAGTGACACCACTTATAATGCACCAAAGGTCTCATGCATAAGTGAAGTTT    960
HEINZ       GTTTGCGTCTCAGTGACACCACTTATAATGCACCAAAGGTCTCATGCATAAGTGAAGTTT    959
            ************************************************************

LA2093      TAGGTGGGAATCAGATGGG-------------------AGGTCTGCAACCGTAGCTGAAG    1000
E42         TAGGTGGGAATCAGATGGGATTATTTGTTGAGGTAACAAGGTCTGCAACCGTAGCTGAAG    1020
HEINZ       TAGGTGGGAATCAGATGGGATTATTTGTTGAGGTAACAAGGTCTGCAACCGTAGCTGAAG    1019
            *******************                   **********************

LA2093      CTAGGTTACAGCTGACTCATCAACCAAGCATCCTAGAGTAACTACTGTCTGCTATAGCAC    1060
E42         CTAGGTTACAGCTGACTCATCAACCAAGCATCCTAGAGTAACTACTGTCTGCTACAGCAC    1080
HEINZ       CTAGGTTACAGCTGACTCATCAACCAAGCATCCTAGAGTAACTACTGTCTGCTACAGCAC    1079
            ****************************************************** *****

LA2093      CATGTGTTGGTTGGCCATTACTATATGGATAGCATATGCATGCTCAGTAAATGATGAGGC    1120
E42         CATGTGTTGGTTGGCCATTACTATATGGATAGCATATGCATGCTCAGTAAATGATGAGGC    1140
HEINZ       CATGTGTTGGTTGGCCATTACTATATGGATAGCATATGCATGCTCAGTAAATGATGAGGC    1139
            ************************************************************

LA2093      AGACAAGGGCAGAGAACAAGAGGTGAGGGGGAGATAAGGGGAACTATCTCTACTAGCACT    1180
E42         AGACAAGGGCAGAGAACAAGAGGTGAGGGGGAGATAAGGGGAACTATCTCTACTAGCACT    1200
HEINZ       AGACAAGGGCAGAGAACAAGAGGTGAGGGGGAGATAAGGGGAACTATCTCTACTAGCACT    1199
            ************************************************************

LA2093      ATATCAGGTGTCATTTAAGAACCTTCATCTTTTGGTAAATTAAAATGTTCAATTCTATGC    1240
E42         ATATCAGGTGTCATTTAAGAACCTTCATCTTTTGGTAAATTAAAATGTTCAATTCTATGC    1260
HEINZ       ATATCAGGTGTCATTTAAGAACCTTCATCTTTTGGTAAATTAAAATGTTCAATTCTATGC    1259
            ************************************************************

LA2093      CATATGAATAAAAGGGCTAACTACAAGACACTATTGTAAGTAAGAGAATGTCAAGATTAC    1300
E42         CATATGAATAAAAGGGCTAACTACAAGACACTATTGTAAGTAAGAGAATGTCAAGATTAC    1320
HEINZ       CATATGAATAAAAGGGCTAACTACAAGACACTATTGTAAGTAAGAGAATGTCAAGATTAC    1319
            ************************************************************

LA2093      CAGAGTTATAGTAACGGTGGTGCGAAGGTCGTTTACTTCTCTTGTGAATTGGTCATGAGG    1360
E42         CAGAGTTATAGTAACGGTGGTGCGAAGGTCGTTTACTTCTCTTGTGAATTGGTCATGAGG    1380
HEINZ       CAGAGTTATAGTAACGGTGGTGCGAAGGTCGTTTACTTCTCTTGTGAATTGGTCATGAGG    1379
            ************************************************************

LA2093      AGCTGTGCGGATGCGGAACTGTTCCAAGGTATTGAAAGGAACCAATATAAGTCACAGTCA    1420
E42         AGCTGTGCGGATGCGGAACTGTTCCAAGGTATTGAAAGGAACCAATATAAGTCACAGTCA    1440
HEINZ       AGCTGTGCGGATGCGGAACTGTTCCAAGGTATTGAAAGGAACCAATATAAGTCACAGTCA    1439
            ************************************************************

LA2093      AGACATACATCATAATGAAACAAGTCCATTTTTTTCGGCATGGAAAAAATCTAACCTTCA    1480
E42         AGACATACATCATAATGAAACAAGTCCATTTTTTTCGGCATGGAAAAAATCTAACCTTCA    1500
HEINZ       AGACATACATCATAATGAAACAAGTCCATTTTTTTCGGCATGGAAAAAATCTAACCTTCA    1499
            ************************************************************

LA2093      GGTCTCCAGGTTCACCATCTATCTTAGGCTCACCATCCTCATAAAAGACCACTTCCTGTA    1540
E42         GGTCTCCAGGTTCACCATCTATCTTAGGCTCACCATCCTCATAAAAGACCACTTCCTGTA    1560
HEINZ       GGTCTCCAGGTTCACCATCTATCTTAGGCTCACCATCCTCATAAAAGACCACTTCCTGTA    1559
            ************************************************************

LA2093      AAAATGATGCTCTATTATTTCAGGGCTCCAGCATATTCCAAAACTTATCGAGAAACACAC    1600
E42         AAAATGATGCTCTATTATTTCAGGGCTCCAGCATATTCCAAAACTTATCGAGAAACACAC    1620
HEINZ       AAAATGATGCTCTATTATTTCAGGGCTCCAGCATATTCCAAAACTTATCGAGAAACACAC    1619
            ************************************************************

LA2093      AACACAAAAAAAGGCAGGTTCCAGTTGTTCAGACACCAAATTCAGCAAGACATGGCATGAA    1660
E42         AACACAAAAAAAGGCAGGTTCCAGTTGTTCAGACACCAAATTCAGCAAGACATGGCATGAA    1680
HEINZ       AACACAAAAAAAGGCAGGTTCCAGTTGTTCAGACACCAAATTCAGCAAGACATGGCATGAA    1679
            ************************************************************

LA2093      GAAATTGAATTTCTAGGGGAAAACATATTGCCACTCAACTTTATGTCTATTATTCACGCA    1720
```

```
E42         GAAATTGAATTTCTAGGGGAAAACATATTGCCACTCAACTTTATGTCTATTATTCACGCA    1740
HEINZ       GAAATTGAATTTCTAGGGGAAAACATATTGCCACTCAACTTTATGTCTATTATTCACGCA    1739
            ************************************************************

LA2093      AATATATTAGTACATCTAAAAGGGAGAAACCAATTAGAAGATTAAACATGAGATGACTTT    1780
E42         AATATATTAGTACATCTAAAAGGGAGAAACCAATTAGAAGATTAAACATGAGATGACTTT    1800
HEINZ       AATATATTAGTACATCTAAAAGGGAGAAACCAATTAGAAGATTAAACATGAGATGACTTT    1799
            ************************************************************

LA2093      GCTCACAGTTTTAAACAAGAAAAGTTCGATAGATCTAAGGAAATTCTAAAATGTTTCCTT    1840
E42         GCTCACAGTTTTAAACAAGAAAAGTTCGATAGATCTAAGGAAATTCTAAAATGTTTCCTT    1860
HEINZ       GCTCACAGTTTTAAACAAGAAAAGTTCGATAGATCTAAGGAAATTCTAAAATGTTTCCTT    1859
            ************************************************************

LA2093      TATTTCAGAAAAAAGGAAATAAAACTACAGGGTATTAACCTCCTTTAGAGCACGTCAAAG    1900
E42         TATTTCAGAAAAAAGGAAATAAAACTACAGGGTATTAACCTCCTTTAGAGCACGTCAAAG    1920
HEINZ       TATTTCAGAAAAAAGGAAATAAAACTACAGGGTATTAACCTCCTTTAGAGCACGTCAAAG    1919
            ************************************************************

LA2093      ACAAGATATACATTTTAAATGCTAAAACTGTAAATTACATTCATATCAAAGTGCCCCACA    1960
E42         ACAAGATATACATTTTAAATGCTAAAACTGTAAATTACATTCATATCAAAGTGCCCCACA    1980
HEINZ       ACAAGATATACATTTTAAATGCTAAAACTGTAAATTACATTCATATCAAAGTGCCCCACA    1979
            ************************************************************

LA2093      TGCCTGGGGAGCTTTCAAACGAACCACCATCAGATTGTTTCTGAGTGAACTTAGGTGCAC    2020
E42         TGCCTGGGGAGCTTTCAAACGAACCACCATCAGATTGTTTCTGAGTGAACTTAGGTGCAC    2040
HEINZ       TGCCTGGGGAGCTTTCAAACGAACCACCATCAGATTGTTTCTGAGTGAACTTAGGTGCAC    2039
            ************************************************************

LA2093      CTCTTATCAGTATCTAGCAAAGGCTACTACTTGTAAGAGCAAAAAGTACATGGCTTTCGC    2080
E42         CTCTTATCAGTATCTAGCAAAGGCTACTACTTGTAAGAGCAAAAAGTACATGGCTTTCGC    2100
HEINZ       CTCTTATCAGTATCTAGCAAAGGCTACTACTTGTAAGAGCAAAAAGTACATGGCTTTCGC    2099
            ************************************************************

LA2093      CGTTTAGGCAGGCAGCTCTAAGTGGCCAGATGTGGACATCGAACACAAAATTATTTTGCT    2140
E42         CGTTTAGGCAGGCAGCTCTAAGTGGCCAGATGTGGACATCGAACACAAAATTATTTTGCT    2160
HEINZ       CGTTTAGGCAGGCAGCTCTAAGTGGCCAGATGTGGACATCGAACACAAAATTATTTTGCT    2159
            ************************************************************

LA2093      AGTCAAATGAAGTTCCTGAGGGCTATTTTGCTCGGACTCTTCAAAAATGCCAACGGGTGC    2200
E42         AGTCAAATGAAGTTCCTGAGGGCTATTTTGCTCGGACTCTTCAAAAATGCCAACGGGTGC    2220
HEINZ       AGTCAAATGAAGTTCCTGAGGGCTATTTTGCTCGGACTCTTCAAAAATGCCAACGGGTGC    2219
            ************************************************************

LA2093      ATGCAGGATTCTCCAAAAGAAAAGTGTTTTTGGGAATTCCGACATGGGTATGGCAAAGAA    2260
E42         ATGCAGGATTCTCCAAAAGAAAAGTGTTTTTGGGAATTCCGACATGGGTATGGCAAAGAA    2280
HEINZ       ATGCAGGATTCTCCAAAAGAAAAGTGTTTTTGGGAATTCCGACATGGGTATGGCAAAGAA    2279
            ************************************************************

LA2093      AGTGAAGATTTGCGCAACTTAGTCTGAGGGTATTAGCGACAAAAAGGGTGCAAGGAAGTA    2320
E42         AGTGAAGATTTGCGCAACTTAGTCTGAGGGTATTAGCGACAAAAAGGGTGCAAGGAAGTA    2340
HEINZ       AGTGAAGATTTGCGCAACTTAGTCTGAGGGTATTAGCGACAAAAAGGGTGCAAGGAAGTA    2339
            ************************************************************

LA2093      AGTATATCATCATAAATACAGGCTTATTTCAGTGTCTATGACAGAAAGGGATCAGCGCAA    2380
E42         AGTATATCATCATAAATACAGGCTTATTTCAGTGTCTATGACAGAAAGGGATCAGCGCAA    2400
HEINZ       AGTATATCATCATAAATACAGGCTTATTTCAGTGTCTATGACAGAAAGGGATCAGCGCAA    2399
            ************************************************************

LA2093      ACAGATAAAGGGTGTAATAGCTTAACAAACAGCATCAAGCATGCAAGTCAAGAATGTAGA    2440
E42         ACAGATAAAGGGTGTAATAGCTTAACAAACAGCATCAAGCATCCAAGTCAAGAATGTAGA    2460
HEINZ       ACAGATAAAGGGTGTAATAGCTTAACAAACAGCATCAAGCATCCAAGTCAAGAATGTAGA    2459
            ****************************************** *****************

LA2093      AGAAATCTCACCTGGCCATCTTGCATTCCTTTCTCAATATCCACAGTAATATGGTACCCT    2500
E42         AGAAATCTCACCTGGCCATCTTGCATTCCTTTCTCAATATCCACAGTAATATGGTACCCT    2520
HEINZ       AGAAATCTCACCTGGCCATCTTGCATTCCTTTCTCAATATCCACAGTAATATGGTACCCT    2519
            ************************************************************

LA2093      TCCCTTTCAAACTTCACGTTGGGGCATTTGTCACAGACCTAAACAGAACAATTCCAGAGT    2560
E42         TCCCTTTCAAACTTCACGTTGGGGCATTTGTCACAGACCTAAACAGAACAATTCCAGAGT    2580
HEINZ       TCCCTTTCAAACTTCACGTTGGGGCATTTGTCACAGACCTAAACAGAACAATTCCAGAGT    2579
```

```
            ************************************************************

LA2093      TCAAAGGTAAATCGAGACGCTGCACCAAATTGTTTGAAATTGACATCTAGAACTTTGGAG    2620
E42         TCAAAGGTAAATCGAGACGCTGCACCAAATTGTTTGAAATTGACATCTAGAACTTCGGAG    2640
HEINZ       TCAAAGGTAAATCGAGACGCTGCACCAAATTGTTTGAAATTGACATCTAGAACTTCGGAG    2639
            ******************************************************* ****

LA2093      AGAACATATACCTGTTCAGTCATCTGCTGAAACATTCCTGGACCAATTTGTCGATGATAA    2680
E42         AGAACATATACCTGTTCAGTCATCTGCTGAAACATTCCTGGACCAATTTGTCGATGATAA    2700
HEINZ       AGAACATATACCTGTTCAGTCATCTGCTGAAACATTCCTGGACCAATTTGTCGATGATAA    2699
            ************************************************************

LA2093      ACCTCATTTCGGCAATTGCACTTCCTCTTACCAGGGGCCGGCTTTAAAATGTTTTTCTCC    2740
E42         ACCTCATTTCGGCAATTGCACTTCCTCTTACCAGGGGCCGGCTTTAAAATGTTTTTCTCC    2760
HEINZ       ACCTCATTTCGGCAATTGCACTTCCTCTTACCAGGGGCCGGCTTTAAAATGTTTTTCTCC    2759
            ************************************************************

LA2093      CTCCAAACCTATATTTTTCACCAATCATACAAGAAACTTTTCTTGATTAGTACAAAAATG    2800
E42         CTCCAAACCTATATTTTTCACCAATCATACAAGAAACTTTTCTTGATTAGTACAAAAATG    2820
HEINZ       CTCCAAACCTATATTTTTCACCAATCATACAAGAAACTTTTCTTGATTAGTACAAAAATG    2819
            ************************************************************

LA2093      TAGAAGAATTATGAAAACTGGTATCACCAGAAAACAAAAAATTAGGCTTAGGGTAGCCCA    2860
E42         TAGAAGAATTATGAAAACTGGTATCACCAAAAAACAAAAAATTAGGCTTAGGGTAGCCCA    2880
HEINZ       TAGAAGAATTATGAAAACTGGTATCACCAAAAAACAAAAAATTAGGCTTAGGGTAGCCCA    2879
            ***************************** ******************************

LA2093      AGAGTTCAAACATGGTGACTCAAGTAATGCAAGAATGCCTCACTCTAGGATATAATATTA    2920
E42         AGAGTTCAAACATGGTGACTCAAGTAATGCAAGAATGCCTCACTCTAGGATATAATATTA    2940
HEINZ       AGAGTTCAAACATGGTGACTCAAGTAATGCAAGAATGCCTCACTCTAGGATATAATATTA    2939
            ************************************************************

LA2093      TGACCTAAACTATTTTTCAACTTCCTATATCTCTACTTTAAGTCATTCCTGTGTTGTTTG    2980
E42         TGACCTAAACTATTTTTCAACTTCCTATATCTCTACTTTAAGTCATTCCTGTGTTGTTTG    3000
HEINZ       TGACCTAAACTATTTTTCAACTTCCTATATCTCTACTTTAAGTCATTCCTGTGTTGTTTG    2999
            ************************************************************

LA2093      ATGGAAAAAGAAGACATGTGAAAGCATATGATAGCTTTCAACTATCTAGATAGTCATACC    3040
E42         ATGGAAAAAGAAGACATGTGAAAGCATATGATAGCTTTCAACTATCTAGATAGTCATACC    3060
HEINZ       ATGGAAAAAGAAGACATGTGAAAGCATATGATAGCTTTCAACTATCTAGATAGTCATACC    3059
            ************************************************************

LA2093      CCTGCGTGTGGTAAAGGGGCGATGATGTCAATTTGACAAGGAATATTTAGAAGAGATGAG    3100
E42         CCTGCGTGTGGTAAAGGGGCGATGATGTCAATTTGACAAGGAATATTTAGAAGAGATGAG    3120
HEINZ       CCTGCGTGTGGTAAAGGGGCGATGATGTCAATTTGACAAGGAATATTTAGAAGAGATGAG    3119
            ************************************************************

LA2093      CATGTTAGTTAACAGGATTTCACCTTCAAGGAGCCCCCCATGTATAAGTCTTCCAATGTA    3160
E42         CATGTTAGTTAACAGGATTTCACCTTCAAGGAGCCCCCCATGTATAAGTCTTCCAATGTA    3180
HEINZ       CATGTTAGTTAACAGGATTTCACCTTCAAGGAGCCCCCCATGTATAAGTCTTCCAATGTA    3179
            ************************************************************

LA2093      GCATCCAAATCAACAATTACATCATCACCTTTCACAATCTTCTCTTCCTCCTCCTCCATT    3220
E42         GCATCCAAATCAACAATTACATCATCACCTTTCACAATCTTCTCTTCCTCCTCCTCCATT    3240
HEINZ       GCATCCAAATCAACAATTACATCATCACCTTTCACAATCTTCTCTTCCTCCTCCTCCATT    3239
            ************************************************************

LA2093      GAGCCCCCGCCGAAAAACCTTCAGTGGTTATTCATTTTGTCAGTTAGTAAGATAGCACAT    3280
E42         GAGCCCCCGCCGAAAAACCTTCAGTGGTTATTCATTTTGTCAGTTAGTAAGATAGCACAT    3300
HEINZ       GAGCCCCCGCCGAAAAACCTTCAGTGGTTATTCATTTTGTCAGTTAGTAAGATAGCACAT    3299
            ************************************************************

LA2093      GCTACACATTTGATCATCATAAATTAACGAATTGACAACTTCAAGAGAAACCAGACAATC    3340
E42         GCTACACATTTGATCATCATAAATTAACGAATTGACAACTTCAAGAGAAACCAGACAATC    3360
HEINZ       GCTACACATTTGATCATCATAAATTAACGAATTGACAACTTCAAGAGAAACCAGACAATC    3359
            ************************************************************

LA2093      AACAACATATCCAGTGGAATAGTGGAATCCCATAAAGGAGGGTAGAGTGTAGGCAAATCT    3400
E42         AACAACATATCCAGTGGAATAGTGGAATCCCATAAAGGAGGGTAGAGTGTAGGCAAATCT    3420
HEINZ       AACAACATATCCAGTGGAATAGTGGAATCCCATAAAGGAGGGTAGAGTGTAGGCAAATCT    3419
            ************************************************************
```

```
LA2093      TACCTCTACCTCGTGGTCGTAGAGAGGCTGAGAAACCAGACAAACTAAGCAGAATTATTC    3460
E42         TACCTCTACCTCGTGGTTGTAGAGAGGCTGAGAAACCAGACAAACTAAGCAGAATTATTC    3480
HEINZ       TACCTCTACCTCGTGGTCGTAGAGAGGCTGAGAAACCAGACAAACTAAGCAGAATTATTC    3479
            ***************** ******************************************

LA2093      CTTTCAAAAAAGATACTTGAAAATCATGGTACATGTGTGTGCAATGTTTACTTCTCTCAC    3520
E42         CTTTCAAAAAAGATACTTGAAAATCATGGTACATGTGTGTGCAATGTTTACTTCTCTCAC    3540
HEINZ       CTTTCAAAAAAGATACTTGAAAATCATGGTACATGTGTGTGCAATGTTTACTTCTCTCAC    3539
            ************************************************************

LA2093      AAGTTGCAAGAGAAGGCAACAAAAACAACTAAATGAACAGAGAAGGCGGGGAGTGTCATC    3580
E42         AAGTTGCAAGAGAAGGCAACAAAAACAACAAAATGAACAGAGAAGGCGGGGAGTGTCATC    3600
HEINZ       AAGTTGCAAGAGAAGGCAACAAAAACAACTAAATGAACAGAGAAGGCGGGGAGTGTCATC    3599
            ***************************** ******************************

LA2093      GCCTAGTAGAAATAGATGAATGTAAGAGTTATTGTAAACCAATGTTTACAATAACTTGGC    3640
E42         GCCTAGTAGAAATAGATGAATGTAAGAGTTATTGTAAACCAATGTTTACAATAACTTGGC    3660
HEINZ       GCCTAGTAGAAATAGATGAATGTAAGAGTTATTGTAAACCAATGTTTACAATAACTTGGC    3659
            ************************************************************

LA2093      AAAGATTGACGGGAACATGTCCGACAAACATACATGAATGGAGTGTAACTTAGTACCTCA    3700
E42         AAAGATTGACGGGAACATGTCCGACAAACATACATGAATGGAGTGTAACTTAGTACCTCA    3720
HEINZ       AAAGATTGACGGGAACATGTCCGACAAACATACATGAATGGAGTGTAACTTAGTACCTCA    3719
            ************************************************************

LA2093      ACAATACAAATTAAAAAAATTTAACAAACAAAAAGTAGCTAGGCAGAAATTATTACTTGG    3760
E42         ACAATACAAATTAAAAAAATTTAACAAACAAAAAGTAGCTAGGCAGAAATTATTACTTGG    3780
HEINZ       ACAATACAAATTAAAAAAATTTAACAAACAAAAAGTAGCTAGGCAGAAATTATTACTTGG    3779
            ************************************************************

LA2093      AAACCACCAGTGTTCAGTAAAGCAAGCACTTCATCGCTTAAAGCGTGAAGCGATAAAGCG    3820
E42         AAACCACCAGTGTTCAGTAAAGCGAGCACTTCATCGCTTAAAGCGTGAAGCGATAAAGCG    3840
HEINZ       AAACCACCAGTGTTCAGTAAAGCGAGCACTTCATCGCTTAAAGCGTGAAGCGATAAAGCG    3839
            *********************** ************************************

LA2093      GGGCCTTATCGCTTTTTTCCGCTTCACACATTACTTCCATAAAGGAAGTATGTCTATGGA    3880
E42         GGGCCTTATCGCTTTTTTCCGCTTCACGCATTACTTCCATAAAGGAAGTGTGTCTATGGA    3900
HEINZ       GGGCCTTATCGCTTTTTTCCGCTTCACACATTACTTCCATAAAGGAAGTATGTCTATGGA    3899
            *************************** ********************* **********

LA2093      ACTTTTTGCCAGGGCGAGACTCTAGCAAAACGCCTCGAGACTCAAGCGTGGGGCTTAGTT    3940
E42         ACTTTTTGCCAGGGCGAGACTCTAGCAAAACGCCTCGAGACTCAAGCGTGGGGCTTAGTT    3960
HEINZ       ACTTTTTGCTAGGGCGAGACTCTAGCAAAACGCCTCGAGACTCAAGCGTGGGGCTTAGTT    3959
            ********* **************************************************

LA2093      CTGTAAAATATCTGCACTAAGCAAGCTCAACGCTCGGGGCTCACCTAACAAATCTTTCAC    4000
E42         CTGTAAAATATCTGCACTAAGCAAGCTCAACGCTCGGGGCTCACCTAACAAATCTTTCAC    4020
HEINZ       CTGTAAAATATCTGCACTAAGCAAGCTCAACGCTCGGGGCTCACCTAACAAATCTTTCAC    4019
            ************************************************************

LA2093      AACTCATGTGTTAAATTATTTAGTTAACATAGTTAACCCTCATAATCCTAGAATAGATGA    4060
E42         AACTCATGTGTTAAATTATTTAGTTAACATAGTTAACCCTCATAATCCTAGAATAGATGA    4080
HEINZ       AACTCATGTGTTAAATTATTTAGTTAACATAGTTAACCCTCATAATCCTAGAATAGATGA    4079
            ************************************************************

LA2093      ATGATACTTAATAGTTATTTAATCTATAGAAATAAGATCGGAAGTAACTCAA-ATAACAA    4119
E42         ATGATACTTAATAGTTATTTAATCTATAGAAATAAGATCGGAAGTAACTCAAAATAACAA    4140
HEINZ       ATGATACTTAATAGTTATTTAATCTATAGAAATAAGATCGGAAGTAACTCAA-ATAACAA    4138
            **************************************************** *******

LA2093      ATTGTAATATTGCATCTTTCCTATCTGGTAAGACTGTGAGGGTGATTATATATTACGTAA    4179
E42         ATTGTAATATTGCATCTTTCCTATCTGGTAAGACTGTGAGGGTGATTATATATTACGTAA    4200
HEINZ       ATTGTAATATTGCATCTTTCCTATCTGGTAAGACTGTGAGGGTGATTATATATTACGTAA    4198
            ************************************************************

LA2093      CATTTCTTTTAGAAATACATAGCGAAATTTTACTTTTTCACTTATCATTGGTTTCATGTT    4239
E42         CATTTCTTTTAGAAATACATAGCGAAATTTTACTTTTTCACTTATCATTGGTTTCATGTT    4260
HEINZ       CATTTCTTTTAGAAATACATAGCGAAATTTTACTTTTTCACTTATCATTGGTTTCATGTT    4258
            ************************************************************

LA2093      TTTGTCATGTCTCAAAATTATCATATTTTATTTAGCTATTTAAATGTAATTTTATTATTA    4299
E42         TTTGTCATGTCTCAAAATTATCATATTTTATTTAGCTATTTAAATGTAATTTTATTATTA    4320
```

```
HEINZ         TTTGTCATGTCTCAAAATTATCATATTTTATTTAGCTATTTAAATGTAATTTTATTATTA    4318
              ************************************************************

LA2093        TTTATGATGGGGTGATGTTTTTATTATCACATACTTTAATTGATAATTTTCTTTTAGGAG    4359
E42           TTTATGATGGGGTGATGTTTTTATTATCACATACTTTAATTGATAATTTTCTTTTAGGAG    4380
HEINZ         TTTATGATGGGGTGATGTTTTTATTATCACATACTTTAATTGATAATTTTCTTTTAGGAG    4378
              ************************************************************

LA2093        GGTTAATTGTATAGTATGATAATAATATACTTCTAGAAATAACGATTCATTTATTATTAG    4419
E42           GGTTAATTGTATAGTATGATAATAATATACTTCTAGAAATAACGATTCATTTATTATTAG    4440
HEINZ         GGTTAATTGTATAGTATGATAATAATATACTTCTAGAAATAACGATTCATTTATTATTAG    4438
              ************************************************************

LA2093        AATGTTAAGATATTAGATTACTTATACTTGTATAATCATGTTAGAAGTTTTGTTTTCTAT    4479
E42           AATGTTAAGATATTAGATTACTTATACTTGTATAATCATGTTAGAAGTTTTGTTTTCTAT    4500
HEINZ         AATGTTAAGATATTAGATTACTTATACTTGTATAATCATGTTAGAAGTTTTGTTTTCTAT    4498
              ************************************************************

LA2093        GAGTTTCCTTAATCATATTTGTAATTATTTGCTAACATATAAGGAATGTTAATACATCTA    4539
E42           GAGTTTCCTTAATCATATTTGTAATTATTTGCTAACATATAAGGAATGTTAATACATCTA    4560
HEINZ         GAGTTTCCTTAATCATATTTGTAATTATTTGCTAACATATAAGGAATGTTAATACATCTA    4558
              ************************************************************

LA2093        AACATCATTGCAGTACTTTAAAAATATAAAAGTAAAACAAACAAACCACTGACACCATAT    4599
E42           AACATCATTGCAGTACTTTAAAAATATAAAAGTAAAACAAACAAACCACTGACACCCTAT    4620
HEINZ         AACATCATTGCAGTACTTTAAAAATATAAAAGTAAAACAAACAAACCACTGACACCCTAT    4618
              ******************************************************** ***

LA2093        TGCAGGAAAAAGGGTCAGTCATTATATATCAAATATAAACATACAATGGACAAAGT-TTT    4658
E42           TGCAGGAAAAAGGGTCAATCATTATATATCAAATATAAACATACAATGGACAAAGTCTTT    4680
HEINZ         TGCAGGAAAAAGGGTCAATCATTATATATCAAATATAAACATACAATGGACAAAGTCTTT    4678
              ***************** ************************************** ***

LA2093        TGATAGAATAATGTAAGGGAGATAAATTTAACAGCAGCTATAAATCTGTAGATGACAATG    4718
E42           TGATAGAATAATGTAAGGGAGATAAATTCAACAGCAGCTATAAATCTGTAGATGACAATG    4740
HEINZ         TGATAGAATAATGTAAGGGAGATAAATTCAACAGCAGCTATAAATCTGTAGATGACAATG    4738
              **************************** *******************************

LA2093        AAACCAACTATAAACCGTACATGATTAAACTGTAAGGGCAATCAGAGACAGCTTAAGCTT    4778
E42           AAACCAACTATAAACCGTACATGATTAAACTGTAAGGGCAATCAGAGACAGCTTAAGCTT    4800
HEINZ         AAACCAACTATAAACCGTACATGATTAAACTGTAAGGGCAATCAGAGACAGCTTAAGCTT    4798
              ************************************************************

LA2093        ATGCTTGTAATCAGAATTATATAAGCGGTCCATGTGCCTACTGATTGACCTTTGCCATAA    4838
E42           ATGCTTGTAATCAGAATTATATAAGCGGTCCATGTGCCTACTGATTGACCTTTGCCATAA    4860
HEINZ         ATGCTTGTAATCAGAATTATATAAGCGGTCCATGTGCCTACTGATTGACCTTTGCCATAA    4858
              ************************************************************

LA2093        TTAATTCAAGATCAGTTTGTTGAACATCATCATTAAGTTGAAAGGTCCCTAACTTAATCC    4898
E42           TTAATTCAAGATCAGTTTGTTGAACATCATCATTAAGTTGAAAGGTCCCTAACTTAATCC    4920
HEINZ         TTAATTCAAGATCAGTTTGTTGAACATCATCATTAAGTTGAAAGGTCCCTAACTTAATCC    4918
              ************************************************************

LA2093        TACTTCAATCCAGTGGCATTACACAAAAGAGTAAAGAACACAGCTAGAAGTTGCAAAATT    4958
E42           TACTTCAATCCAGTGGCATTACACAAAAGAGTAAAGAACACAGCTAGAAGTTGCAAAATT    4980
HEINZ         TACTTCAATCCAGTGGCATTACACAAAAGAGAAAAGAACACAGCTAGAAGTTGCAAAATT    4978
              ******************************* ****************************

LA2093        AAGATTAACAAAACGCTGAACACTAGACATTGCAATAAGATGCACCTAACCACTCTATCA    5018
E42           AAGATTAACAAAACGCTGAACACTAGACATTGCAATAAGATGCACCTAACCACTCTATCA    5040
HEINZ         AAGATTAACAAAACGCTGAACACTAGACATTGCAATAAGATGCACCTAACCACTCTATCA    5038
              ************************************************************

LA2093        ATCAGATATAATTATAGGGGTCTATTTTTTCGGGTTCATTTCATTCCAAAAAGAATATGC    5078
E42           ATCAGATATAATTATAGGGGTCTATTTTTTCGGGTTCATTTCATTCCAAAAAGAATATGC    5100
HEINZ         ATCAGATATAATTATAGGGGTCTATTTTTTCGGGTTCATTTCATTCCAAAAAGAATATGC    5098
              ************************************************************

LA2093        AACCATTGAATAACTATAATTCGTAGGTGAGCAAACAAAAACAATGTCACAAAATAATAC    5138
E42           AACCATTGAATAACTATAATTCGTAGGTGAGCAAACAAAAACAATGTCACAAAATAATAC    5160
HEINZ         AACCATTGAATAACTATAATTCGTAGGTGAGCAAACAAAAACAATGTCACAAAATAATAC    5158
              ************************************************************
```

```
LA2093      AATTGAGGATAAAGTGAAACTATAGAAACTCAACATACTGGCTAAATATGTCTTGCATGT     5198
E42         AATTGAGGATAAAGTGAAACTATAGAAACTCAACATACTGGCTAAATATGTCTTGCATGT     5220
HEINZ       AATTGAGGATAAAGTGAAACTATAGAAACTCAACATACTGGCTAAATATGTCTTGCATGT     5218
            ************************************************************

LA2093      TCATTCCACCTCCTCTGCCTCCGCTAGCAGCATGTTGTTTAAGACCCTCTTCCCCATATC     5258
E42         TCATTCCACCTCCTCTGCCTCCGCTAGCAGCATGTTGTTTAAGACCCTCTTCCCCATATC     5280
HEINZ       TCATTCCACCTCCTCTGCCTCCGCTAGCAGCATGTTGTTTAAGACCCTCTTCCCCATATC     5278
            ************************************************************

LA2093      GATCATATATATTTCTCTTTTCACTGTCCGAAATCACTTCATAAGCTGAAAATATCCCAA     5318
E42         GATCATATATATTTCTCTTTTCACTGTCCGAAATCACTTCATAAGCTGAAAATATCCCAA     5340
HEINZ       GATCATATATATTTCTCTTTTCACTGTCCGAAATCACTTCATAAGCTGAAAATATCCCAA     5338
            ************************************************************

LA2093      AAAATGAGAGTCAATCACACAATTAGCAATCTAAGCACCAAACTAGTTGCAGATATCTAG     5378
E42         AAAATGAGAGTCAATCACACAATTAGCAATCTAAGCACCAAACTAGTTGCAGATATCTAG     5400
HEINZ       AAAATGAGAGTCAATCACACAATTAGCAATCTAAGCACCAAACTAGTTGCAGATATCTAG     5398
            ************************************************************

LA2093      ATCTATTCAGCATCCTATACTGGTAAGCATATGCATTAGACAATTAATCATATAAAACTA     5438
E42         ATCTATTCAGCATCCTATACTGGTAAGCATATGCATTAGATAATTAATCATATAAAACTA     5460
HEINZ       ATCTATTCAGCATCCTATACTGGTAAGCATATGCATTAGATAATTAATCATATAAAACTA     5458
            **************************************** *******************

LA2093      AACACAACATACCATTACTGATTTCAGCAAATTTCTTATTAGCCTCTTCATTTCCCTGAT     5498
E42         AACACAACATACCATTACTGATTTCAGCAAATTTCTTATTAGCCTCTTCATTTCCCTGAT     5520
HEINZ       AACACAACATACCATTACTGATTTCAGCAAATTTCTTATTAGCCTCTTCATTTCCCTGAT     5518
            ************************************************************

LA2093      TCTTATCAGGGTGATACTTCAAAGCAAGCTTCCTATAAGCTCGTTTGATCTGCTCATCGG     5558
E42         TCTTATCAGGGTGATACTTCAACGCAAGCTTCCTATAAGCTCGTTTGATCTGCTCATCGG     5580
HEINZ       TCTTATCAGGGTGATACTTCAACGCAAGCTTCCTATAAGCTCGTTTGATCTGCTCATCGG     5578
            ********************** *************************************

LA2093      AAGCACCTTTTGGAATTTGCAAAATATCATAGTAACTCTTCCTGCACACATCAAACACAC     5618
E42         AAGCACCTTTTGGAATTTGCAAAATATCATAGTAACTCTTCCTGCACACATCAAACACAC     5640
HEINZ       AAGCACCTTTTGGAATTTGCAAAATATCATAGTAACTCTTCCTGCACACATCAAACACAC     5638
            ************************************************************

LA2093      ATTGTCACAAAAAAGAAAAAAAAAACACAAAACACAACGGAGAAAAAGGAGATGAACTCG     5678
E42         ATTGTCACAAAAAAGAAAAAAAAAACACAAAACACAACGGAGAAAAAGGAGATGAACTCG     5700
HEINZ       ATTGTCACAAAAAAGAAAAAAAAAACACAAAACACAACGGAGAAAAAGGAGATGAACTCG     5698
            ************************************************************

LA2093      TATAAAAAACTCACGCAGCAATGGCAATTGACAAAGTGTAGGATAAACAACACAACAAGA     5738
E42         TATAAAAAACTCACGCAGCAATGGCAATTGACAAAGTGTAGGATAAACAACACAACAAGA     5760
HEINZ       TATAAAAAACTCACGCAGCAATGGCAATTGACAAAGTGTAGGATAAACAACACAACAAGA     5758
            ************************************************************

LA2093      TGAGCAACTTTGATCTTCTATGCGCCATAAGCACACAGATCTAAGGAATCAAAAACTATT     5798
E42         TGAGCAACTTTGATCTTCTATGCGCCATAAGCACACAGATCTAAGGAATCAAAAACTATT     5820
HEINZ       TGAGCAACTTTGATCTTCTATGCGCCATAAGCACACAGATCTAAGGAATCAAAAACTATT     5818
            ************************************************************

LA2093      CCTCACAACAAATTGAAATTCGATTGAATCGATAGCTTCGTTTTTTCTTTAACTAGATCT     5858
E42         CCTCACAACAAATTGAAATTCGATTGAATCGATAGCTTCGTTTTTTCTTTAACTAGATCT     5880
HEINZ       CCTCACAACAAATTGAAATTCGATTGAATCGATAGCTTCGTTTTTTCTTTAACTAGATCT     5878
            ************************************************************

LA2093      ACTAGAACAATCTAGAATTTATGAATATGAAATTTGTGTTAATTTCACGGCAAAAGCTTA     5918
E42         ACTAGAACAATCTAGAATTTATGAATATGAAATTTGTGTTAATTTCACGGCAAAAGCTTA     5940
HEINZ       ACTAGAACAATCTAGAATTTATGAATATGAAATTTGTGTTAATTTCACGGCAAAAGCTTA     5938
            ************************************************************

LA2093      AAGAATATTAAACCGCGGAACAGGGCGGACGTTTGAATATTGAACAAAACGACTGACTTC     5978
E42         AAGAATATTAAACCGCGGAACAGGGCGGACGTTTGAATATTGAACAAAACGACTGACTTC     6000
HEINZ       AAGAATATTAAACCGCGGAACAGGGCGGACGTTTGAATATTGAACAAAACGACTGACTTC     5998
            ************************************************************

LA2093      ACACTGC    5985
```

```
E42          ACACTGC    6007
HEINZ        ACACTGC    6005
             *******
```

Solyc01g079640

```
E42         AATTGCATAAGAGTGCGATTTGGGTGCGCACGTACCAAATGCAAAGTTATA-GGCATATT     59
Heinz       AATTGCATGAGAGTGCGATTTGGGTGCGCACGTACCAAATGCAAAGTCATAGGGCATATT     60
LA2093      AATTGCATGAGAGTGCGATTTGGGTGCGCACGTACCAAATGCAAAGTCATAGGGCATATT     60
            ******** ************************************** *** ********

E42         TTGTAACATGTAAAACATATGTGCCAGTGTTATTTGCTCTTGATGAGAGTCCCTTCCTGC     119
Heinz       TTGTAACATGTAAAACATATGTGCCAGTGTTATTTGCTCTTAATGAGAGTCCCTTCCTGC     120
LA2093      TTGTAACATGTAAAACATATGTGCCAGTGTTATTTGCTCTTAATGAGAGTCCCTTCCTGC     120
            ***************************************** ******************

E42         AAATCCCCTAATGAAAATGAAGCCCAGAATCCCTGCTTCAATTTCTGCTCTAATGGCAGG     179
Heinz       AAATCCCCTAATGAAAATGAAGCCCAGAATCCCTGCTTCAATTTCTGCTCTAATGGCAGG     180
LA2093      AAATCCCCTAATGAAAATGAAGCCCAGAATCCCTGCTTCAATTTCTGCTCTAATGGCAGG     180
            ************************************************************

E42         TAAAACTCTTCCTCTAAAACCCTAAATTTTTATCAAATGGCTCCTTCCAATTCAATTGTC     239
Heinz       TAAAACTCTTCCTCTAAAACCCTAAATTTTTATCAAATGGCTCCTTCCAATTCAATTGTC     240
LA2093      TAAAACTCTTCCTCTAAAACCCTAAATTTTTATCAAATGGCTCCTTCCAATTCAATTGTC     240
            ************************************************************

E42         CCTTCTCCTCCACCGCAGGTGAATGTGCCGGAATTTGCAAAGCCATTTCTGGCCTCTTAC     299
Heinz       CCTTCTCCTCCACCGCAGGTGAATGTGCCGGAATTTGCAAAGCCATTTCTGGCCTCTTAC     300
LA2093      CCTTCTCCTCCACCGCAGGTGAATGTGCCGGAATTTGCAAAGCCATTTCTGGCCTCTTAC     300
            ************************************************************

E42         CGGAATCTCAAACATTAAACGGCTTCGGTCGAAGCTGCAGCAGGTAAATACACTTTAAAC     359
Heinz       CGGAATCTCAAACATTAAACGGCTTCGGTCGAAGCTGCAGCAGGTAAATACACTTTAAAC     360
LA2093      CGGAATCTCAAACATTAAACGGCTTCGGTCGAAGCTGCAGCAGGTAAATACACTTTAAAC     360
            ************************************************************

E42         CTTGTGATTTAACACCAGTTCTATTTGTAAAGATGAATTTGAAGCAGCTAGGAAAAT-TT     418
Heinz       CTTGTGATTTAACACCAGTTCTATTTTTAAAGATGAATTTGAAGCAGCTAGGAAAATTTT     420
LA2093      CTTGTGATTTAACACCAGTTCTATTTTTAAAGATGAATTTGAAGCAGCTAGGAAAATTTT     420
            ************************** ****************************** **

E42         CCTTCATCAGTTTGCCAATAGGTGGAGGATTAATACAGTATCTATATCCGTTTTACAAAT     478
Heinz       CCTGCATCAGTTTGCCAATAGGTGGAGGATTAATACAGTATCTATATCCGTTTTACAAAT     480
LA2093      CCTGCATCAGTTTGCCAATAGGTGGAGGATTAATACAGTATCTATATCCGTTTTACAAAT     480
            *** ********************************************************

E42         TTATCCTGGTTGTTTGTGCTAATATAGGTTTTTTAATGTGAAATCATCAGAACTGTTCAT     538
Heinz       TTATCCTGGTTGTTTGTGCTAATATAGGTTTTTTAATGTGAAATCATCAGAACTGTTCAT     540
LA2093      TTATCCTGGTTGTTTGTGCTAATATAGGTTTTTTAATGTGAAATCATCAGAACTGTTCAT     540
            ************************************************************

E42         TTTAAAAAAAAAATTGTAATTCGTTTTCATTATGGATTGTAGTTCATCTATTCGTTGTGTT     598
Heinz       TTTAAA-AAAAATTGTAATTCGTTTTCATTATGGATTGTAGTTCATCTATTCGTTGTGTT     599
LA2093      TTTAAAAAAAAAATTGTAATTCGTTTTCATTATGGATTGTAGTTCATCTATTCGTTGTGTT     600
            ****** *****************************************************

E42         TACTTAGAGGACGAGTTTGTTGTCAATAGCAAAATTTAAGAGCATGTTTGGATG-GGCGT     657
Heinz       TACTTAGAGGACGAGTTTGTTGTCAATAGCAAAATTTAAGAGCATGTTTGGATGGGGCGT     659
LA2093      TACTTAGAGGACGAGTTTGTTGTCAATAGCAAAATTTAAGAGCATGTTTGGATG-GGCGT     659
            ****************************************************** *****

E42         ATAGCTTACGACTTATAACTAATAGTCATTAAGTTAGGGCAATAGAAATTTTAAAGTTCA     717
Heinz       ATAGCTTACGACTTATAACTAATAGTCATTAAGTTAGGGCAATAGAAATTTTAAAGTTCA     719
LA2093      ATAGCTTACGACTTATAACTAATAGTCATTAAGTTAGGGCAATAGAAATTTTAAAGTTCA     719
            ************************************************************

E42         AGTGAAGAAAAAAAAAAAGATGAGATGATATCTCCTATAGAGAAGGTACAAAAATGAATG     777
Heinz       AGTGAAGAAAAAAAAAAAGATGAGATGATATCTCCTATAGAGAAGGTACAAAAATGAATG     779
LA2093      AGTGAAGAAAAAAAAAAAGATGAGATGATATCTCCTATAGAGAAGGTACAAAAATGAATG     779
            ************************************************************

E42         TCAAGTTTGCAGTATATCTGAATGGATTTAGCTAAAATCTTGTAGGAGTAATTGGTAAAA     837
Heinz       TCAAGTTTGCAGTATATCTGAATGGATTTAGCTAAAATCTTGTAGGAGTAATTGGTAAAA     839
LA2093      TCAAGTTTGCAGTATATCTGAATGGATTTAGCTAAAATCTTGTAGGAGTAATTGGTAAAA     839
            ************************************************************
```

```
E42         AGTTGTGTAGTTTTGCCTCAACATAGATGCTTTTACTACCTTACTGAAAGCACGCCAAGG     897
Heinz       AGTTGTGTAGTTTTGCCTCAACATAGATGCTTTTACTACCTTACTGAAAGCACGCCAAGG     899
LA2093      AGTTGTGTAGTTTTGCCTCAACATAGATGCTTTTACTACCTTACTGAAAGCACGCCAAGG     899
            ************************************************************

E42         CAAAGGTACCTAGGAATATGTATAGAGTAGGAAGTAGTGATGTACTTCCAAAATGCAAAA     957
Heinz       CAAAGGTACCTAGGAATATGTATAGAGTAGGAAGTAGTGATGTACTTCCAAAATGCAAAA     959
LA2093      CAAAGGTACCTAGGAATATGTATAGAGTAGGAAGTAGTGATGTACTTCCAAAATGCAAAA     959
            ************************************************************

E42         TTGGATTACTCCAAGAAACCACATTATAGATTAACCAACATATGATGGATGGACCAAAGA     1017
Heinz       TTGGATTACTCCAAGAAACCACATTATAGATTAACCAACATATGATGGATGGACCAAAGA     1019
LA2093      TTGGATTACTCCAAGAAACCACATTATAGATTAACCAACATATGATGGATGGACCAAAGA     1019
            ************************************************************

E42         AGCAGATTCAATTGTCTTCCTGATACCATTCCCTAATTTAATACATTGGAGATTATTGAA     1077
Heinz       AGCAGATTCAATTGTCTTCCTGATACCATTCCCTAATTTAATACATTGGAGATTATTGAA     1079
LA2093      AGCAGATTCAATTGTCTTCCTGATACCATTCCCTAATTTAATACATTGGAGATTATTGAA     1079
            ************************************************************

E42         CGTGCTCATGAGTTAATTGAAGAATGAAGCTTCATTTTTGTATAGGTGGGCAATTGGGTT     1137
Heinz       CGTGCTCATGAGTTAATTGAAGAATGAAGCTTCATTTTTGTATAGGTGGGCAATTGGGTT     1139
LA2093      CGTGCTCATGAGTTAATTGAAGAATGAAGCTTCATTTTTGTATAGGTGGGCAATTGGGTT     1139
            ************************************************************

E42         GTATTAGGCCGGTTCTGGTTCTAACCACTCTTTTTGCCCTGCTATGCTGAGGAAATCTTG     1197
Heinz       GTATTAGGCCGGTTCTGGTTCTAACCACTCTTTTTGCCCTGCTATGCTGAGGAAATCTTG     1199
LA2093      GTATTAGGCCGGTTCTGGTTCTAACCACTCTTTTTGCCCTGCTATGCTGAGGAAATCTTG     1199
            ************************************************************

E42         TGGACCCTTTGAGGGGTAGATTGATGCCTAATGGTCTAGATTTGTCCTTCGTTAACATTC     1257
Heinz       TGGACCCTTTGAGGGGTAGATTGATGCCTAATGGTCTAGATTTGTCTTTCGTTAACATTC     1259
LA2093      TGGACCCTTTGAGGGGTAGATTGATGCCTAATGGTCTAGATTTGTCCTTCGTTAACATTC     1259
            ********************************************** *************

E42         ATGATCAGATTTACCCTCATCGTTACACTATGGACTAGATTCGCCCCTTTCCTTTGTAAA     1317
Heinz       ATGGTCAGATTTACCCTCATCGTTACACTATGGACTAGATTCGCCCCTTTCCTTTGTAAA     1319
LA2093      ATGGTCAGATTTACCCTCATCGTTACACTATGGACTAGATTCGCCCCTTTCCTTTGTAAA     1319
            *** ********************************************************

E42         AATGTCCATGTAAGCCTTCTTTGTGTCAATAAGGGGAAATTTAGCCCATTTTTGTGACAA     1377
Heinz       AATGTCCATGTAAGCCTTCATTGTGTCAATAAGGGGAAATTTAGCCCATTTTTGTGACAA     1379
LA2093      AATGTCCATGTAAGCCTTCATTGTGTCAATAAGGGGAAATTTAGCCCATTTTTGTGACAA     1379
            ******************* ****************************************

E42         CAGGGTAGATCTGATTCCAACTATTAATGGAGGGAGAACTTAAACAATTTCGCAAACTTC     1437
Heinz       CGGGGTAGATCTGATTCCAACTATTAATGGAGGGCGAACTTAAACAATTTCGCAAACTTC     1439
LA2093      CAGGGTAGATCTGATTCCAACTATTAATGGAGGGCGAACTTAAACAATTTCGCAAACTTC     1439
            * ******************************** *************************

E42         AAGGACAAATTTGGACAATTTCGTACAATTG---GGCTAATTTGACCCTTTTTTAGTTGA     1494
Heinz       AAGGACAAATTTGGACAATTTCGTACAATTGAAGGGCTAATTTGACCCTTTTTTAGTTGA     1499
LA2093      AAGGACAAATTTGGACAATTTCGTACAATTGAAGGGCTAATTTGACCCTTTTTTAGTTGA     1499
            *******************************   **************************

E42         GAAAATGTCCACATTTTGCAAAACCTTCGACGTTAGTCAATAGAGGCAAATTAAACCCAT     1554
Heinz       GAAAATGTCCACATTTTGCAAAACCTTCGACGTTAGTCAATAGAGGCAAATTAAACCCAT     1559
LA2093      GAAAATGTCCACATTTTGCAAAACCTTCGACGTTAGTCAATAGAGGCAAATTAAACCCAT     1559
            ************************************************************

E42         TTTAGTAATAGGAGGGGCGAATCTGACCCAACTATTTATGGAGGGCAAACTTAAGCCTCA     1614
Heinz       TTTAGTAATAGGAGGGGCGAATCTGACCCAACTATTTATGGAGGGCAAACTTAAGCCTCA     1619
LA2093      TTTAGTAATAGGAGGGGCGAATCTGACCCAACTATTTATGGAGGGCAAACTTAAGCCTCA     1619
            ************************************************************

E42         TTTATTTGCACTTATTGGAGGTTTGAATATGAATAATTCAGACATTAGACCATTAAGTGC     1674
Heinz       TTTATTTGCACTTATTGGAGGTTTGAATATGAATAATTCAGACATTAGACCATTAAGTGC     1679
LA2093      TTTATTTGCACTTATTGGAGGTTTGAATATGAATAATTCAGACATTAGACCATTAAGTGC     1679
            ************************************************************

E42         ATTTGTTTTTATTAAGATTTAAGCACTTAACGGGCTTGGATATGTCTTAATCACTAAGAT     1734
```

```
Heinz     ATTTGTTTTTATTAAGATTTAAGCACTTAACGGGCTTGGATATGTCTTAATCACTAAGAT    1739
LA2093    ATTTGTTTTTATTAAGATTTAAGCACTTAACGGGCTTGGATATGTCTTAATCACTAAGAT    1739
          ************************************************************

E42       CTAGAACAGTGTCTTAATATGATTAAGAGGTCTATCCACAATCACCAGCCACCACTCTGC    1794
Heinz     CTAGAACAGTGTCTTAATATGATTAAGAGGTCTATCCACAATCACCAGCCACCACTCTGC    1799
LA2093    CTAGAACAGTGTCTTAATATGATTAAGAGGTCTATCCACAATCACCAGCCACCACTCTGC    1799
          ************************************************************

E42       CATCACAACAACCACCACTACTAACCACCTCTGTCATCACCACCACCACCATTATTGTCA    1854
Heinz     CATCACAACAACCACCACTACTAACCACCTCTGTCATCACCACCACCACCATTATTGTCA    1859
LA2093    CATCACAACAACCACCACTACTAACCACCTCTGTCATCACCACCACCACCATTATTGTCA    1859
          ************************************************************

E42       ACGACCATTGCCTCGTCTACCACCAACCACCACTTGCAAACTATTACTAGCAGGTGCTAC    1914
Heinz     ACGACCATTGCCTCGTCTACCACCAACCACCACTTGCAAACTATTACTAGCAGGTGCTAC    1919
LA2093    ACGACCATTGCCTCGTCTACCACCAACCACCACTTGCAAACTATTACTAGCAGGTGCTAC    1919
          ************************************************************

E42       CACACTTATCTTCTTTATCATTATGATTATATCCAATGCCACCACCACCAGCCACCTTTA    1974
Heinz     CACACTTATCTTCTTTATCATTATGATTATATCCAATGCCACCACCACCAGCCACCTTTA    1979
LA2093    CACACTTATCTTCTTTATCATTATGATTATATCCAATGCCGCCACCACCAGCCACCTTTA    1979
          **************************************** *******************

E42       AATATTTTATTTTTTATTTGAATTGTATATTTATTATGTACATATAAATAAATTATGTAC    2034
Heinz     AATATTTTATTTTTTATTTGAATTGTATATTTATTATGTACATATAAATAAATTATGTAC    2039
LA2093    AATATTTTATTTTTTATTTGAATTGTATATTTATTATGTACATATAAATAAATTATGTAC    2039
          ************************************************************

E42       ATTCAGTTGTTGAAAACAAGCAATCTTAATCATTCAATGTTCAAATCTAGAGATAACATC    2094
Heinz     ATTCAGTTGTTGAAAACAAGCAATCTTAATCATTCAATGTTCAAATCTAGAGATAACATC    2099
LA2093    ATTCAGTTGTTGAAAACAAGCAATCTTAATCATTCAATGTTCAAATCTAGAGATAACATC    2099
          ************************************************************

E42       TTAATTATTTAGATGTGCATTCAGATTCAGACGTCTAAATTTTAATGAAAACAAATGAGG    2154
Heinz     TTAATTATTTAGATGTGCATTCAGATTCAGACGTCTAAATTTTAATGAAAACAAATGAGG    2159
LA2093    TTAATTATTTAGATGTGCATTCAGATTCAGACGTCTAAATTTTAATGAAAACAAATGAGG    2159
          ************************************************************

E42       CCTTAAAACCATTTCGCAAAGTTTAAGGGCAATTTTGGACTTTTTACTTTTTAGAATAAT    2214
Heinz     CCTTAAAACCATTTCGCAAAGTTTAAGGGCAATTTTGGACTTTTTACTTTTTAGAATAAT    2219
LA2093    CCTTAAAACCATTTCGCAAAGTTTAAGGGCAATTTTGGACTTTTTACTTTTTAGAATAAT    2219
          ************************************************************

E42       ATGTTTAGTATGAATAAAGTTGTAAATCTTGCAAAAAACACATCCACAAAATAGGTGAAA    2274
Heinz     ATGTTTAGTATGAATAAAGTTGTAAATCTTGCAAAAAACACACCCACAAAATAGGTGAAA    2279
LA2093    ATGTTTAGTATGAATAAAGTTGTAAATCTTGCAAAAAACACATCCACAAAATAGGTGAAA    2279
          ****************************************** *****************

E42       ATGGGTCCAGAAGTAGAGATAGTATTTATTTGGAAACATTTTAAATTACTTTAGTTCAAA    2334
Heinz     ATGGGTCCAGAAGTAGAGATAGTATTTATTTGGAAACATTTTAAATTACTTTAGTTCAAA    2339
LA2093    ATGGGTCCAGAAGTAGAGATAGTATTTATTTGGAAACATTTTAAATTACTTTAGTTCAAA    2339
          ************************************************************

E42       AATGGGTTGCACAGATAGAATGCTGAAAAGTAATACACTGTCAAAATAAGTTGACAACTT    2394
Heinz     AATGGGTTGCACAGATAGAATGCTGAAAAGTAATACACTGTCAAAATAAGTTGACAACTT    2399
LA2093    AATGGGTTGCACAGATAGAATGCTGAAAAGTAATACACTGTCAAAATAAGTTGACAACTT    2399
          ************************************************************

E42       TGGAATAACCTAAAATAGAAATAGATAGATTATTGGGTATGAAGGAAGTAGTAATAAGTA    2454
Heinz     TGGAATAACCTAAAATAGAAATAGATAGATTATTGGGTATGAAGGAAGTAGTAATAAGTA    2459
LA2093    TGGAATAACCTAAAATAGAAATAGATAGATTATTGGGTATGAAGGAAGTAGTAATAAGTA    2459
          ************************************************************

E42       ATCATCAGAGGGTAACACCTTCCCACGGTAGGGTTCAGGTGAAGGCCAACAAATAACCAG    2514
Heinz     ATCATCAGAGGGTAACACCTTCCCACGGTAGGGTTCAGGTGAAGGCCAACAAATAACCAG    2519
LA2093    ATCATCAGAGGGTAACACCTTCCCACGGTAGGGTTCAGGTAAAGGCCAACAAATAACCAG    2519
          **************************************** *******************

E42       TACACTAACAAAACCAAGGTTAGCAAGCTGCAGAAACTAAAAGGACTGAATGTGTCAATA    2574
Heinz     TACACTAACAAAACCAAGGTTAGCAAGCTGCAGAAACTAATAGGACTGAATGTGTCAATA    2579
LA2093    TACACTAACAAAACCAAGGTTAGCAAGCTGCAGAAACTAATAGGACTGAATGTGTCAATA    2579
```

```
                ****************************************  *******************

E42             GCATAGCAAAGACCTCTAGGCATATAGCTTCTGAAAAAGAATAAACTGCCTTCCTAAAGG    2634
Heinz           GCATAGCAAAGACCTCTAGGCATATAGCTTCTGAAAAAGAATAAACTGCCTTCCTAAAGG    2639
LA2093          GCATAGCAAAGACCTCTAGGCATATAGCTTCTGAAAAAGAATAAACTGCCTTCCTAAAGG    2639
                ************************************************************

E42             CTCATTAGCTTTAAATAAATTATACTTAAATGAACAGTGAAAACTAGAATCTGGAGGTTT    2694
Heinz           CTCATTAGCTTTAAATAAATTATACTTAAATGAACAGTGAAAACTAGAATCTGGAGGTTT    2699
LA2093          CTCATTAGCTTTAAATAAATTATACTTAAATGAACAGTGAAAACTAGAATCTGGAGGTTT    2699
                ************************************************************

E42             TAGGGTAGGATAATTATAATCTGGTAAGATGAGCAGGGACATGAGAAATGCCACTATGGG    2754
Heinz           TAGGGTAGGATAATTATAATCTGGTAAGATGAGCAGGGACATGAGAAATGCCACTATGGG    2759
LA2093          TAGGGTAGGATAATTATAATCTGGTAAGATGAGCAGGGACATGAGAAATGCCACTATGGG    2759
                ************************************************************

E42             TATCAAAGTTGGGAAGCAAAGAAATTAGAAAAAAAAAATTTGGTATTTTGTCTGTACATC    2814
Heinz           TATCAAAGTTGGGAAGCAAAGAAATTAGAAAAAAAAAACTTGGTATTTTGTCTGTACATC    2819
LA2093          TATCAAAGTTGGGAAGCAAAGAAATTAGAAAAAAAAAACTTGGTATTTTGTCTGTACATC    2819
                ************************************** *********************

E42             TCTTGCCTGCTAGCTCAACCTGTGGATTTTCCTGATTATTGATTTTGTAGACTTTATTCT    2874
Heinz           TCTTGCCTGCTAGCTCAACCTGTGGATTTTCCTGATTATTGATTTTGTAGACTTTATTCT    2879
LA2093          TCTTGCCTGCTAGCTCAACCTGTGGATTTTCCTGATTATTGATTTTGTAGACTTTATTCT    2879
                ************************************************************

E42             GTCTATTCAAATCAAACATCATGCTTTATTATTGATTATTATGCTATTCTTACACCTTAA    2934
Heinz           GTCTATTCAAATCAAACATCATACTTTATTATTGATTATTGTGCTATTCTTACACCTTAA    2939
LA2093          GTCTATTCAAATCAAACATCATACTTTATTATTGATTATTGTGCTATTCTTACACCTTAA    2939
                ********************** ***************** *******************

E42             AGTTAACCAGCTTCCAGTTCCAAAGATTTCAGAGCTCAAAGGGTTTACTATTCAAAGTCC    2994
Heinz           AGTTAACCAGCTTCCAGTTCCAAAGATTTCAGAGCTCAAAGGGTTTACTATTCAAAGTCC    2999
LA2093          AGTTAACCAGCTTCCAGTTCCAAAGATTTCAGAGCTCAAAGGGTTTACTATTCAAAGTCC    2999
                ************************************************************

E42             ATAATGATGATAACGACTTCTCAGACCTGGGTCCTCCAGTAGAAAGAGCTTTTGGGCAGC    3054
Heinz           ATAATGATGATAACGACTTCTCAGACCTGGGTCCTCCAGTAGAAAGAGCTTTTGGGCAGC    3059
LA2093          ATAATGATGATAACGACTTCTCAGACCTGGGTCCTCCAGTAGAAAGAGCTTTTGGGCAGC    3059
                ************************************************************

E42             TGAAATTAGTGACGGAGAAGCGCGATCCTTTTCCGGTATGTTTTTCTGATTTTATTTATA    3114
Heinz           TGAAATTAGTGACGGAGAAGCGCGATCCTTTTCCGGTATGTTTTTCTGATTTTATTTATA    3119
LA2093          TGAAATTAGTGACGGAGAAGCGCGATCCTTTTCCGGTATGTTTTTCTGATTTTATTTATA    3119
                ************************************************************

E42             GCATAGGACTTCTAATGGTTGGGTACATGTACTAATTGGCTTGAATATGTTACCATTACC    3174
Heinz           GCATAGGACTTCTAATGGTTGGGTACATGTACTAATTGGTTTGAATATGTTACCATTACC    3179
LA2093          GCATAGGACTTCTAATGGTTGGGTACATGTACTAATTGGCTTGAATATGTTACCATTACC    3179
                *************************************** ********************

E42             TTTTGAGTCACAGAAATTTGATAATGGTCGAGAAGTATTATCTCGCAAGTCTCACAGCAA    3234
Heinz           TTTTGAGTCACAGAAATTTGATAATGGTCGAGAAGTATTATCTCGCAAGTCTCACAGCAA    3239
LA2093          TTTTGAGTCACAGAAATTTGATAATGGTCGAGAAGTATTATCTCGCAAGTCTCACAGCAA    3239
                ************************************************************

E42             AAGCATGCAACACGTAAAGGGAAAAGTCTTAACTCTTGATGAACCTCATCAAAGTGGGTG    3294
Heinz           AAGCATGCAACACGTAAAGGGAAAAGTCTTAACTCTTGATGAACCTCATCAAAGTGGGTG    3299
LA2093          AAGCATGCAACACGTAAAGGGAAAAGTCTTAACTCTTGATGAACCTCATCAAAGTGGGTG    3299
                ************************************************************

E42             TGGTAATGGTGATGATGCTTCTGGGTCGACTTATACAACTTCAGATGCTGCTTGTATGGA    3354
Heinz           TGGTAATGGTGATGATGCTTCTGGGTCGACTTATACAACTTCAGATGCTGCTTGTATGGA    3359
LA2093          TGGTAATGGTGATGATGCTTCTGGGTCGACTTATACAACTTCAGATGCTGCTTGTATGGA    3359
                ************************************************************

E42             CGTCCGGAGGTCAATCTCAGTTGGGAACGTACCTTCTACCATCAGCCTTCCTCAGCTTGT    3414
Heinz           CGTCCGGAGGTCAATCTCAGTTGGGAACGTACCTTCTACCATCAGCCTTCCTCAGCTTGT    3419
LA2093          CGTCCGGAGGTCAATCTCAGTTGGGAACGTACCTTCTATCATCAGCCTTCCTCAGCTTGT    3419
                ************************************** *********************
```

```
E42       AGAAGCAGTTTCAGTCTTCGGAAAGTTCTGTGCTGCTTCAGTCAGGCATCTTCCCGATGG   3474
Heinz     AGAAGCAGTTTCAGTCTTCGGAAAGTTCTGTGCTGCTTCAGTCAGGCATCTTCCCGATGG   3479
LA2093    AGAAGCAGTTTCAGTCTTCGGAAAGTTCTGTGCTGCTTCAGTCAGGCATCTTCCCGATGG   3479
          ************************************************************

E42       GCTTAACTGTTGGGACATCCAATTCAAGGTATGCTATTGATTTCTTGAACTTCTGCCTTC   3534
Heinz     GCTTAACTGTTGGGACATCCAATTCAAGGTATGCTATTGATTTCTTGAACTTCTGCCTTC   3539
LA2093    GCTTAACTGTTGGGACATCCAATTCAAGGTATGCTATTGATTTCTTGAACTTCTGCCTTC   3539
          ************************************************************

E42       AGCCCCCTCAATCAGGAAAAGTTTGTTTATTGACTGTTGTTAACTATTGGCAAGAGCTTA   3594
Heinz     AGCCCCCTCAATCAGGAAAAGTTTGTTTATTAACTGTTGTTAACTATTGGCAAGAGCTTA   3599
LA2093    AGCCCCCTCAATCAGGAAAAGTTTGTTTATTAACTGTTGTTAACTATTGGCAAGAGCTTA   3599
          ******************************* ****************************

E42       GAATCCCGCAATAGAGCAGTTAGAGCTGGTGCGCTTTATCTTGGGAGTTATCGCCTTCCA   3654
Heinz     GAATCCCGCAATAGAGCAGTTAGAGCTGGTGCGCTAAATCTTGGGAGGTATCGCCTTCCA   3659
LA2093    GAATCCCGCAATAGAGCAGTTAGAGCTGGTGCGCTAAATCTTGGGAGTTATCGCCTTCCA   3659
          ***********************************  ********** ************

E42       ATTCAACCCCCACGGGCTTCTATGGTTGTTACTATCAAGATTGAGGACATTCCCGATGAT   3714
Heinz     ATTCAACCCCCACGGGCTTCTATGGTTGTTACTATCAAGATTGAGGACATTCCCGATGAT   3719
LA2093    ATTCAACCCCCACGGGCTTCTATGGTTGTTACTATCAAGATTGAGGACATTCCCGATGAT   3719
          ************************************************************

E42       GCTTCCCTCAGTGAGATGCACTCGATCTGTAAATCTGTTGGCACGACAGAAGGGTTGGCA   3774
Heinz     GCTTCCCTCAGTGAGATGCACTCGATCTGTAAATCTGTTGGCACGACAGAAGGGTTGGCA   3779
LA2093    GCTTCCCTCAGTGAGATGCACTCGATCTGTAAATCTGTTGGCACGACAGAAGGGTTGGCA   3779
          ************************************************************

E42       TGGGTAAGCAAGGATAGTGTAGAAGCCTTATTTACAGTTGAGAATGACAAAGAATCTGAG   3834
Heinz     TGGGTAAGCAAGGATAGTGTAGAAGCCTTATTTACAGTTGAGAATGACAAAGAATCTGAG   3839
LA2093    TGGGTAAGCAAGGATAGTGTAGAAGCCTTATTTACAGTTGAGAATGACAAAGAATCTGAG   3839
          ************************************************************

E42       TCGATCCTCAAAAAGTAAGTGGTGGTTCATCCTGGAACAAAAGGTCTTATTGTTACTGTT   3894
Heinz     TCGATCCTCAAAAAGTAAGTGGTGGTTCATCCTGGAACAAAAGGTCTTATTGTTACTGTT   3899
LA2093    TCGATCCTCAAAAAGTAAGTGGTGGTTCATCCTGGAACAAAAGGTCTTATTGTTACTGTT   3899
          ************************************************************

E42       TTCACTCTTGTAGGCTAGGGGGCTGGTTCTTGTCCATTATTTTGCCATCTCAAAAGTATA   3954
Heinz     TTCACTCTTGTAGGCTAGGGGGCTGGTTCTTGTCCATTATTTTGCCATCTCAAAAGTATA   3959
LA2093    TTCACTCTTGTAGGCTAGGGGGCTGGTTCTTGTCCATTATTTTGCCATCTCAAAAGTATA   3959
          ************************************************************

E42       AAACTGTTCTCACTTCATTTTGGAAGAAGTTGGAGGGGGGGAATCAATCCAAATATGACT   4014
Heinz     AAACTGTTCTCACTTCATTTTGGAAGAAGTTGGAGGGGGGGAATCAATCCAAATATGACT   4019
LA2093    AAACTGTTCTCACTTCATTTTGGAAGAAGTTGGAGGGGGGGAATCAATCCAAATATGACT   4019
          ************************************************************

E42       -TGCTTTATTACAGCAGCATCATTAATCATATTCCTTATTGTTCCATGTAGTTCTTCACT   4073
Heinz     TTGCTTTATTACAGCAGCATCATTAATCATATTCCTTATTGTTCCATGTAGTTCTTCACT   4079
LA2093    TTGCTTTATTACAGCAGCATCATTAATCATATTCCTTATTGTTCCATGTAGTTCTTCACT   4079
           ***********************************************************

E42       TACCATTGCATCCATACTCCATTTTCTTTGCATGTACCTTATTAATAGACAAAAACGAGA   4133
Heinz     TACCATTGCATCCATACTCCATTTTCTTTGCATGTACCTTATTAATAGACAAAAACGAGA   4139
LA2093    TACCATTGCATCCATACTCCATTTTCTTTGCATGTACCTTATTAATAGACAAAAACGAGA   4139
          ************************************************************

E42       AGACTGATATTGGCATCTGCTCCGTTCATTTTCTGCCTTCCTTTTTTCCTTAGCAGAACA   4193
Heinz     AGACTGATATTGGCATCTGCTCCGTTCATTTTCTGCCTTCCTTTTTTCCTTAGCAGAACA   4199
LA2093    AGACTGATATTGGCATCTGCTCCGTTCATTTTCTGCCTTCCTTTTTTCCTTAGCAGAACA   4199
          ************************************************************

E42       CACTGTACTTCCATTATTGGTGGACACTGGTTGAAAACGCATGATATTTCATCTTTATTT   4253
Heinz     CACTGTACTTCCATTATTGGTGGACACTGGTTGAAAACGCATGATATTTCATCTTTATTT   4259
LA2093    CACTGTACTTCCATTATTGGTGGACACTGGTTGAAAACGCATGATATTTCATCTTTATTT   4259
          ************************************************************

E42       GGTCGCAGACTTTAATTATACCACCTGAAATCAAATTGTTGCACATCTTGTTCCCAATTT   4313
Heinz     GGTCACAGACTTTAATTATACCACCTGAAATCAAATTGTTGCACATCTTGTTCCCATTTT   4319
```

```
LA2093     GGTCACAGACTTTAATTATACCACCTGAAATCAAATTGTTGCACATCTTGTTCCCATTTT    4319
           **** *************************************************** ***

E42        AACCTTGCCTTAACTAGAAAGAATATGCTGGTGTAACTTTGTTATGTATTTTTATCTTCT    4373
Heinz      AACCTTGCCTTAACTAGAAAGAATATGCTGGTGTAACTTTGTTATGTATTTTTATCTTCT    4379
LA2093     AACCTTGCCTTAACTAGAAAGAATATGCTGGTGTAACTTTGTTATGTATTTTTATCTTCT    4379
           ************************************************************

E42        TAGGTTGAATGGTGCTATTGTTGGTGGTCATTGCCTATCAGCTTCTTTAGTACCTAGCAA    4433
Heinz      TAGGTTGAATGGTGCTATTGTTGGTGGTCATTGCCTATCAGCTTCTTTAGTACCTAGCAA    4439
LA2093     TAGGTTGAATGGTGCTATTGTTGGTGGTCATTGCCTATCAGCTTCTTTAGTACCTAGCAA    4439
           ************************************************************

E42        CTCATCATCTGCTTCCATGTCTGAAAATAAGGATGATAGATGCAGGATGGCTTTACAAAT    4493
Heinz      CTCATCATCTGCTTCCATGTCTGAAAATAAGGATGATAGATGCAGGATGGCTTTACAAAT    4499
LA2093     CTCATCATCTGCTTCCATGTCTGAAAATAAGGATGATAGATGCAGGATGGCTTTACAAAT    4499
           ************************************************************

E42        TAACAACTACTTGACAGAACTCAAAATGCAACTTGAGGAAAAGGAAATGGATTGGCTTGA    4553
Heinz      TAACAACTACTTGACAGAACTCAAAATGCAACTTGAGGAAAAGGAAATGGATTGGCTTGA    4559
LA2093     TAACAACTACTTGACAGAACTCAAAATGCAACTTGAGGAAAAGGAAATGGATTGGCTTGA    4559
           ************************************************************

E42        ATTGTCGGTGCTGAAATCGTGTATGAAGGATCTTCAGATGCTGCATGAGGAGATAATGCA    4613
Heinz      ATTGTCGGTGCTGAAATCGTGTATGGAGGATCTTCAGATGCTGCATGAGGAGATAATGCA    4619
LA2093     ATTGTCGGTGCTGAAATCGTGTATGGAGGATCTTCAGATGCTGCATGAGGAGATAATGCA    4619
           ************************* **********************************

E42        CCTTGAAGATCTGCCTTCCATCATCGATTCATCTGACAATTGATTCTTGATTTTCAAAAC    4673
Heinz      CCTTGAAGATCTGCCTTCCATCATCGATTCATCTGACAATTGATTCTTGATTTTCAAAAC    4679
LA2093     CCTTGAAGATCTGCCTTCCATCATCGATTCATCTGACAATTGATTCTTGATTTTCAAAAC    4679
           ************************************************************

E42        TTCTCTATTCAAGTATCTTCACTTGCTAACAACAGGATAACTCTCTTTCATTCCCCCTTC    4733
Heinz      TTCTCTATTCAAGTATCTTCACTTGCTAACAACAGGATAACTCTCTTTCATTCCCCCTTC    4739
LA2093     TTCTCTATTCAAGTATCTTCACTTGCTAACAACAGGATAACTCTCTTTCATTCCCCCTTC    4739
           ************************************************************

E42        CTTCATGTGCCTCCTCCTTGGCATTGCTTCTACAATTAACTGCTGAAACCGAACCCGACT    4793
Heinz      CTTCATGTGCCTCCTCCTTGGCATTGCTTCTACAATTAACTGCTGAAACCGAACCCGACT    4799
LA2093     CTTCATGTGCCTCCTCCTTGGCATTGCTTCTACAATTAACTGCTGAAACCGAACCCGACT    4799
           ************************************************************

E42        TTAATTCCTTTAATTTTTACAATTTCGTTTTCATATTTGTTGGACTGATTACCCTTTAAA    4853
Heinz      TTAATTCCTTTAATTTTTACAATTTCGTTTTCATATTTGTTGGACTGATTACCCTTTAAA    4859
LA2093     TTAATTCCTTTAATTTTTACAATTTCGTTTTCATATTTGTTGGACTGATTACCCTTTAAA    4859
           ************************************************************

E42        AGAAGATATTGGTTGATGATCTGTTTGTGGTGATTGTTAATTGATACAATATCGAAATGC    4913
Heinz      AGAAGATATTGGTTGATGATCTGTTTGTGGTGATTGTTAATTGATACAATATCGAAATGC    4919
LA2093     AGAAGATATTGGTTGATGATCTGTTTGTGGTGATTGTTAATTGATACAATATCGAAATGC    4919
           ************************************************************

E42        AGAAATTGAAACCGTAAATTCTAAGAAACAAGAATGAAGATAGATCGACACAAACAAAAG    4973
Heinz      AGAAATTGAACCCGTAAATTCTAAGAAACAAGAATAAAGATAGATCGACACAAACAAAAG    4979
LA2093     AGAAATTGAAACCGTAAATTCTAAGAAACAAGAATGAAGATAGATCGACACAAAGAAAAG    4979
           ********** ************************ ****************** *****

E42        AAGTAATATACAACAATGACTATATCACAGTTACAACCTTCGGTGGAAATTACGTGTGAG    5033
Heinz      AAGTAATATACAACAATGACTATATCACAGTTACAACCTTCGGTGGAAATTACGTGTGAG    5039
LA2093     AAGTAATATACAACAATGACTATATCACAGTTACAACTTTCGGTGGAAATTACGTGTGAG    5039
           ************************************* **********************

E42        AACCAAAGAAAGGAGATTCTGTGATTTGTATTATTCAACCTTTTCCTGAGCTTAAGATTG    5093
Heinz      AACCAAAGAAAGGAGATTCTGTGATTTGTATTATTCAACCTTTTCCTGAGCTTAAGATTG    5099
LA2093     AACCAAAGAAAGGAGATTCTGTGATTTGTATTATTCAACCTTTTCCTGAGCTTAAGATTG    5099
           ************************************************************

E42        AACTGGAAATAAAGTCTCTCTACTTGATGATCAACGACAAACAATAAATCAACCTCTCTG    5153
Heinz      AACTGGAAATAAAGTCTCTCTACTTGATGATCAACGACAAACAATAAATCAACCTCTCTG    5159
LA2093     AACTGGAAATAAAGTCTCTCTACTTGATGATCAACGACAAACAATAAATCAACCTCTCTG    5159
           ************************************************************
```

```
E42       ATGCCTTATCATCCTAATTCATATCACACTATGAATGTATATTTATTTGGCCAATTGCTC    5213
Heinz     ATGCCTTATCATCCTAATTCATATCACACTATGAATGTATATTTATTTGGCCAATTGCTC    5219
LA2093    ATGCCTTATCATCCTAATTCATATCACACTATGAATGTATATTTATTTGGCCAATTGCTC    5219
          ************************************************************

E42       ATAGATTTCCTATACTTGTGCTTTTGTACATGTTTTTATAATTGTAAAACAAGTTTTTTT    5273
Heinz     ATAGATTTCCTATACTTGTGCTTTTGTACATGTTTTTATAATTGCAAAACAAGTTTTTTT    5279
LA2093    ATAGATTTCCTATACTTGTGCTTTTGTACATGTTTTTATAATTGTAAAACAAGTTTTTTT    5279
          ******************************************** ***************

E42       GAGAGTGGATAGGCATGGGGCAGGAAAGTGAAACAAGACAAGGAAAACAATGGCAGAATC    5333
Heinz     GAGAGTGGATAGGCATGGGGCAGGAAAGTGAAACAAGACAAGGAAAACAATGGCAGAATC    5339
LA2093    GAGAGTGGATAGGCATGGGGCAGGAAAGTGAAACAAGACAAGGAAAACAATGGCAGAATC    5339
          ************************************************************

E42       GGGTAAGAGTGTAAACAGAAGGTGGTCATAGAACAGGACTGTTTTCTGAAGTCGTTACCT    5393
Heinz     GGGTAATAGTGTAAACAGAGGGTGGTCATAGAACAGGACTGTTTTCTGAAGTCGTTACCT    5399
LA2093    GGGTAATAGTGTAAACAGAAGGTGGTCATAGAACAGGACTGTTTTCTGAAGTCGTTACCT    5399
          ****** ************ ****************************************

E42       TTGAGCATGAAGGGCTCAATCGATCCTCGAAAGTATAGCAGTAACTGACAATGACATGTA    5453
Heinz     TTGAGCATGAAGGGCTCAATCGATCCTCGAAAGTATAGCAGTAACTGACAATGACATGTT    5459
LA2093    TTGAGCATGAAGGGCTCAATCGATCCTCGAAAGTATAGCAGTAACTGACAATGACATGTA    5459
          *********************************************************** 

E42       CTGTGTTTAATCTACAGATTCACCTTCTTTTGGTCCCATATTCAAAATACTACAGGGATC    5513
Heinz     CTGTGTTTAATCTACAGATTCATCTTCTTTTGGTCCCATATTCAAAATACTACAGGGATC    5519
LA2093    CTGTGTTTAATCTACAGATTCATCTTCTTTTGGTCCCATATTCAAAATACTACAGGGATC    5519
          ********************** *************************************

E42       TTCCCCATTTTCGGGTGCTGATACACTCATTGACGAGCATTGTTCAATCTTATATTCTCT    5573
Heinz     TTCCCCATTTTCGGGTGCTGATACACTCATTGACGAGCATTGTTCAATCTTATATTCTCT    5579
LA2093    TTCCCCATTTTCGGGTGCTGATACACTCATTGACGAGCATTGTTCAATCTTATATTCTCT    5579
          ************************************************************

E42       CTCGTCAACTAAAGACTTCATGTAGGAACAAGGCCGGTCCTCAGTCTCCATATGATTAGG    5633
Heinz     CTCGTCAACTAAAGACTTCATGTAGGAACAAGGCCGGTCCTCAGTCTCCATATGATTAGG    5639
LA2093    CTCGTCAACTAAAGACTTCATGTAGGAACAAGGCCGGTCCTCAGTCTCCATATGATTAGG    5639
          ************************************************************

E42       CTCCTGGCCTGGAGTAAAATGCAATTCAAATGATGATGGTCCAGGAGGTTGAGTCCTCAT    5693
Heinz     CTCCTGGCCTGGAGTAAAATGCAATTCAAATGATGATGGTCCAGGAGGTTGAGTCCTCAT    5699
LA2093    CTCCTGGCCTGGAGTAAAATGCAATTCAAATGATGATGGTCCAGGAGGTTGAGTCCTCAT    5699
          ************************************************************

E42       TTGCACAGACCGCAAAAGTGCAGCTCTCCTTAAGTCAGCAGAGTGGCATATGTCACTGGG    5753
Heinz     TTGCACAGACCGCAAAAGTGCAGCTCTCCTTAAGTCAGCAGAGTGGCATATGTCACTGGG    5759
LA2093    TTGCACAGACCGCAAAAGTGCAGCTCTCCTTAAGTCAGCAGAGTGGCATATGTCACTGGG    5759
          ************************************************************

E42       TGATGATGGAAATCGACCCTCAGAATCAGAGCCTCGTTGACGAGGCTGGGAGGATGTGGC    5813
Heinz     TGATGATGGAAATCGACCCTCAGAATCAGAGCCTCGTTGACGAGGCTGGGAGGATGTGGC    5819
LA2093    TGATGATGGAAATCGACCCTCAGAATCAGAGCCTCGTTGACGAGGCTGGGAGGATGTGGC    5819
          ************************************************************

E42       AGGAGGGAAGTTGCAGGAATGTGACGGATATGACGTGGAGGGAGGGCATGATGAGAACCC    5873
Heinz     AGGAGGGAAGTTGCAGGAATGTGACGGATATGACGTGGAGGGAGGGCATGATGAGAACCC    5879
LA2093    AGGAGGGAAGTTGCAGGAATGTGACGGATATGACGTGGAGGGAGGGCATGATGAGAACCC    5879
          ************************************************************

E42       ATTCATTGGTCTGTGATATCGGTAGGGTGAGACTGGACTAGTTGGTGCACTCTCACGTTG    5933
Heinz     ATTCATTGGTCTGTGATATCGGTAGGGTGAGACTGGACTAGTTGGTGCACTCTCACGTTG    5939
LA2093    ATTCATTGGTCTGTGATATCGGTAGGGTGAGACTGGACTAGTTGGTGCACTCTCACGTTG    5939
          ************************************************************

E42       GGGTGAGCTGGTGTTCGTCAAAGCCTCATAACACCGTGAATCATCTGAATCTTCTGACAT    5993
Heinz     GGGTGAGCTGGTGTTCGTCAAAGCCTCATAACACCGTGAATCATCTGAATCTTCTGACAT    5999
LA2093    GGGTGAGCTGGTGTTCGTCAAAGCCTCATAACACCGTGAATCATCTGAATCTTCTGACAT    5999
          ************************************************************

E42       TGGTGAATATTGAAAGGACAGCTCGTCCCTGAAACAAACATAAAAAATCAAATAAAACTT    6053
```

```
Heinz      TGGTGAATATTGAAAGGACAGCTCGTCCCTGAAACAAACATAAAAAATCAAATAAAACTT    6059
LA2093     TGGTGAATATTGAAAGGACAGCTCGTCCCTGAAACAAACATAAAAAATCAAATAAAACTT    6059
           ************************************************************

E42        GATAACAAATAAGCAAGGAGATAGGAAAACAGGATCTTCATCTGATGCTACATTTCTTAT    6113
Heinz      GATAACAAATAAGCAAGGAGATGGGAAAACAGGATCTTCATCTGATGCTACATTTCTTAT    6119
LA2093     GATAACAAATAAGCAAGGAGATAGGAAAACAGGATCTTCATCTGATGCTACATTTCTTAT    6119
           ********************** *************************************

E42        AAGCTTACAGGTACATTGTCAAGTCACAAATAGTAAAGACATTCAAATCTAAGAAGAATT    6173
Heinz      AAGCTTAGAGGTACATTGTCAAGTCACAAATAGTAAAGACATTCAAATCTAAGAAGAATT    6179
LA2093     AAGCTTACAGGTACATTGTCAAGTCACAAATAGTAAAGACATTCAAATCTAAGAAGAATT    6179
           ******* ****************************************************

E42        CATGGAGTCTATCTCTACATCAAAGAAAAAATTCAGAGAGCTAGAGCAAAATTACATGTTT    6233
Heinz      CATGGAGTCTATCTCTACATCAAAGAAAAAATTCAGAGAGCTAGAGCAAAATTACATGTTT    6239
LA2093     CATGGAGTCTATCTCTACATCAAAGAAAAAATTCAGAGAGCTAGAGCAAAATTACATGTTT    6239
           ************************************************************

E42        ATGTACTCCACAAAAGTAAGCAAAATCCAAATGCATGCATGATGATAGATTGGTCTATCT    6293
Heinz      ATGTACTCCACAAAAGTAAGCAAAATCCAAATGCATGCATGATGATAGATTGGTCTATCT    6299
LA2093     ATGTACTCCACAAAAGTAAGCAAAATCCAAATGCATGCATGATGATAGATTGGTCTATCT    6299
           ************************************************************

E42        ACAGATTCTATGCAGTAGTTTGTGTGGTTCTTTCCCATCATGTGTGGTTCTAAAAGGGAT    6353
Heinz      ACAGATTCTATGCAGTAGTTTGTGTGGTTCTTTCCCATCATGTGTGGTTCTAAAAGGGAT    6359
LA2093     ACAGATTCTATGCAGTAGTTTGTGTGGTTCTTTCCCATCATGTGTGGTTCTAAAAGGGAT    6359
           ************************************************************

E42        TAAGAACTTCTCTTTAGACACTAAACTGGACCAAAAAGACCAAGACTTCGCCGATCATAA    6413
Heinz      TAAGAACTTCTCTTTAGACACTAAACTGGACCAAAAAGACCAAGACTTCGCCGATCATAA    6419
LA2093     TAAGAACTTCTCTTTAGACACTAAACTGGACCAAAAAGACCAAGACTTCGCCGATCATAA    6419
           ************************************************************

E42        CTTGGAAACGTATTTTTGCACGATTATTGCAGTTTCATTTAACAGTAGAATACAGTCCTC    6473
Heinz      CTTGGAAACGTATTTTTGCACGATTATTGCAGTTTCATTTAACAGTAGAATACAGTCCTC    6479
LA2093     CTTGGAAACGTATTTTTGCACGATTATTGCAGTTTCATTTAACAGTAGAATACAGTCCTC    6479
           ************************************************************

E42        AATATTTGCATGGTGCATCAGTTTTCTCTAAACAAAATATGCAAGTCAGCGCCTTTCAAG    6533
Heinz      AATATTTGCATGGTGCATCAGTTTTCTCTAAACAAAATATGCAAGTCAGCGCCTTTCAAG    6539
LA2093     AATATTTGCATGGTGCATCAGTTTTCTCTAAACAAAATATGCAAGTCAGCGCCTTTCAAG    6539
           ************************************************************

E42        ATTTTGGAACTACAAAGCCATACAGCGGTGACCCCTCTATTACGCTTAAAAGTCAAATGA    6593
Heinz      ATTTTGGAACTACAAAGCCATACAGCGGTGACCCCTCTATTACGCTTAAAAGTCAAATGA    6599
LA2093     ATTTTGGAACTACAAAGCCATACAGCGGTGACCCCTCTATTACGCTTAAAAGTCAAATGA    6599
           ************************************************************

E42        CTATACGGCCTGTACTTCCTTGCCAACCATTTTTTGCTCTAAACTCCAAGTGCCATGACA    6653
Heinz      CTATACGGCCTGTACTTCCTTGCCAACCATTTTTTGCTCTAAACTCCAAGTGCCATGACA    6659
LA2093     CTATACGGCCTGTACTTCCTTGCCAACCATTTTTTGCTCTAAACTCCAAGTGCCATGACA    6659
           ************************************************************

E42        GGAGACAGTGAGCCACTGCTTGTTTCATCTGTAAACTTTGATATGTTCCCAATGTCTCTG    6713
Heinz      GGAGACACTGAGCCACTGCTTGTTTCATCTGTAAACTTTGATATGTTCCCAATGTCTCTG    6719
LA2093     GGAGACAGTGAGCCACTGCTTGTTTCATCTGTAAACTTTGATATGTTCCCAATGTCTCTG    6719
           ******* ****************************************************

E42        TATATAAAGCTCTGAACACTTTGCATTTTAACAGAATTGCACTGACAGACACTACAAGGT    6773
Heinz      TATATAAAGCTCTGAACACTTTGCATTTTAACAGAATTGCACTGACAGACACTACAAGGT    6779
LA2093     TATATAAAGCTCTGAACACTTTGCATTTTAACAGAATTGCACTGACAGACACTACAAGGT    6779
           ************************************************************

E42        TTCACAGAATCAAAGTAGTGAATCTAACTCTGATCATCACAGAATCAGCGCAGATTAGTC    6833
Heinz      TTCACAGAATCAAAGTAGTGAATCTAACTCTGATCATCACAGAATCAGGGCAGATTAGTC    6839
LA2093     TTCACAGAATCAAAGTAGTGAATCTAACTCTGATCATCACAGAATCAGCGCAGATTAGTC    6839
           ************************************************ ***********

E42        ACTCTCTAAAGAAAGACAGCTGCCAGATGACATCTACGAATTCTAGTCCTCTGTTATCCA    6893
Heinz      ACTCTCTAAAGAAAGACAGCTGCCAGATGACATCTACGAATTCTAGTCCTCTGTTATCCA    6899
LA2093     ACTCTCTAAAGAAAGACAGCTGCCAGATGACATCTACGAATTCTAGTCCTCTGTTATCCA    6899
```

```
                ************************************************************

E42             TCACTCCAAAAAGAGCTGTCACATTTTGAACGCCCATTTCTACAAGTTTTGATAGGTTCT      6953
Heinz           TCACTCCAAAAAGAGCTGTCACATTTTGAACGCCCATTTCTACAAGTTTTGATAGGTTCT      6959
LA2093          TCACTCCAAAAAGAGCTGTCACATTTTGAACGCCCATTTCTACAAGTTTTGATAGGTTCT      6959
                ************************************************************

E42             GATTATATATATACTGATTCATCTGTCAAAGATGCAGACATGAAACCCGACAAGGAAGAA      7013
Heinz           GATTATATATATACTGATTCATCTGTCAAAGATGCAGACATGAAACCCGACAAGGAAGAA      7019
LA2093          GATTATATATATACTGATTCATCTGTCAAAGATGCAGACATGAAACCCGACAAGGAAGAA      7019
                ************************************************************

E42             TCCCGCACCACAGTATATTCACTTGCAACTATACCCTTAATAATTGCCGCCCCACACTAG      7073
Heinz           TCCCGCACCACAGTATATTCACTTGCAACTATACCCTTAATAATTGCCGTCCCACACTAG      7079
LA2093          TCCCGCACCACAGTATATTCACTTGCAACTATACCCTTAATAATTGCCGCCCCACACTAG      7079
                ************************************************* **********

E42             AGTGTTTCAATAGTTTACAGATCTTAAAGGTTCTATTAACACTCTC-TACCAACTCCCTC      7132
Heinz           AGTGTTTCAATAGTTTACAGATCTTAAAGGTTCTATTAACACTCTCCTACCAACTCCCTC      7139
LA2093          AGTGTTTCAATAGTTTACAGATCTTAAAGGTTCTATTAACACTCTCCTACCAACTCCCTC      7139
                ********************************************** *************

E42             AGCACAACATTATGGCAGTGAAATTCAAGAGGTCAACGATGTGTGTATGTCATGTGAATG      7192
Heinz           AGCATAACATTATGGCAGTGAAATTCAAGAGGTCAACGATGTGTGTATGTCATGTGAATG      7199
LA2093          AGCACAACATTATGGCAGTGAAATTCAAGAGGTCAACGATGTGTGTATGTCATGTGAATG      7199
                **** *******************************************************

E42             AGCATCTTTCTACAAGGGAAATATGAGTAAAAAGGTTTTGAGGATGTAGCAAAAGATTGA      7252
Heinz           AGCATCTTTCTACAAGGGAAATATGAGTAAAAAGGTTTTGAGGATGTAGCAAAAGATTGA      7259
LA2093          AGCATCTTTCTACAAGGGAAATATGAGTAAAAAGGTTTTGAGGATGTAGCAAAAGATTGA      7259
                ************************************************************

E42             GGTGGCTACTCTCCATCCAACCTTGTTTATTTAATGCTTCCCAGTTACAAGGTAACATAC      7312
Heinz           GGTGGCTACTCTCCATCCAACCTTGTTTATTTAATGCTTCCCAGTTACT-----------      7308
LA2093          GGTGGCTACTCTCCATCCAACCTTGTTTATTTAATGCTTCCCAGTTACAAGGTAACATAC      7319
                ************************************************

E42             TAGAAAAAGGAACCATTGAATTCTAAGATATAATAATAATAATAATAACAAGAATAAGGT      7372
Heinz           -AGAAAAAGGAACCATTGAATTCTAAGATATAATAATAATAATAATAACAAGAATAAGGT      7367
LA2093          TAGAAAAAGGAACCATTGAATTCTAAGATATAATAATAATAATAATAACAAGAATAAGGT      7379
                 ***********************************************************

E42             GTGTAGCCGTTAGAGCTTACTTTGAGTGGCATAGGTTGAGGGAGACTTGTGACTAAGGTC      7432
Heinz           GCGTAGCCGTTAGAGCTTACTTTGAGTGGCATAGGTTGAGGGAGACTTGTGACTAAGGTC      7427
LA2093          GTGTAGCCGTTAGAGCTTACTTTGAGTGGCATAGGTTGAGGGAGACTTGTGACTAAGGTC      7439
                * **********************************************************

E42             ACGGGTTCGAGCCTTACACTAAGCCTGGTTTCAGAGGTTGTGTTTGGTCCTAAGGGTAGG      7492
Heinz           ACGGGTTCGAGCCTTACACTAAGCCCGGTTTCAGAGGTTGTGTTTGGTCCTAAGGGTAGG      7487
LA2093          ACGGGTTCGAGCCTTACACTAAGCCTGGTTTCAGAGGTTGTGTTTGGTCCTAAGGGTAGG      7499
                ************************* **********************************

E42             CCAAGATGGATTTTTCGGTCATAAAAAAGAATATTAATAATAAGGCGGGTAAGTTTCAAT      7552
Heinz           CCAAGATGGATTTTTCAGTCATAAAAAAGAATATTAATAATAAGGCGGGTAAGTTTCAAT      7547
LA2093          CCAAGATGGATTTTTCGGTCATAAAAAAGAATATTAATAATAAGGCGGGTAAGTTTCAAT      7559
                **************** *******************************************

E42             TATATATTGGAAATGGACTGGACAGCTTCATATACCATGTAAGTCATGGAGGTTATTGGA      7612
Heinz           TATATATTGGAAATGGACTGGACAGCTTCATAGACCATGTAAGTCATGGAGGTTATTGGA      7607
LA2093          TATATATTGGAAATGGACTGGACAGCTTCATATACCATGTAAGTCATGGAGGTTATTGGA      7619
                ******************************** ***************************

E42             TATTATTTTATTTTTAATGTAAATGTATTTCATTCATGGCCAAAAACTGGCCATTACAAG      7672
Heinz           TATTATTTTATTTTTAATGTAAATGTATTTCATTCATGGCCAAAAACTGGCCATTACAAG      7667
LA2093          TATTATTTTATTTTTAATGTAAATGTATTTCATTCATGGCCAAAAACTGGCCATTACAAG      7679
                ************************************************************

E42             CTAATCAGACATATTATTCGATATTAAAGTCCCCATATCCTTGCTAATCAGGCATACTAT      7732
Heinz           CTAATCAGACATATTATTCGATATTAAAGTCCCCATATCCTTGCTAATCAGGCATACTAT      7727
LA2093          CTAATCAGACATATTATTCGATATTAAAGTCCCCATATCCTTGCTAATCAGGCATACTAT      7739
                ************************************************************
```

```
E42         AACTTACTTGTTGCAGCATACAGTGCTTCTCTTTCATTTTTTATTATATTTTAGAGGAGG    7792
Heinz       AACTTACTTGTTGCAGCATACAGTGCTTCTCTTTCATTTTTTATTTATTTTAGAGGAGG    7787
LA2093      AACTTACTTGTTGCAGCATACAGTGCTTCTCTTTCATTTTTTATTATATTTTAGAGGAGG    7799
            ********************************************* **************

E42         GTCTAGACCAAAAGATGCCAGTAGCATGTTCGATTTACCTTCTAGTAACTTTTAAATTTA    7852
Heinz       GTCTAGACCAAAAGATGCCAGTAGCATGTTCGATTTACCTTCTAGTAACTTTTAAATTTA    7847
LA2093      GTCTAGACCAAAAGATGCCAGTAGCATGTTCGATTTACCTTCTAGTAACTTTTAAATTTA    7859
            ************************************************************

E42         ACATATGATAGATTATAAGTCTAAAGGAAACCTAACCTAAACCAACATAAGCATACACTG    7912
Heinz       ACATATGATAGATTATAAGTCTAAAGGAAACCTAACCTAAACCAACATAAGCATACACTG    7907
LA2093      ACATATGATAGATTATAAGTCTAAAGGAAACCTAACCTAAACCAACATAAGCATACACTG    7919
            ************************************************************

E42         CAGGGAAGCAATTGATATACGTGGGGAAGAAGAAAAGAAAATTCTCTGTCTGCTTTAACT    7972
Heinz       CAGGGAAGCAATTGATATACGTGGGGAAGAAGAAAAGAAAATTCTCTGTCTACTTTAATT    7967
LA2093      CAGGGAAGCAATTGATATACGTGGGGAAGAAGAAAAGAAAATTCTCTGTCTGCTTTAATT    7979
            *************************************************** ****** *

E42         CTTGCTTGTCTCTCCATTTAACATCAGTCCTCTCAAAAGGAGAAGTTCTAACACAATTTA    8032
Heinz       CTTGCTTGTCTCTCCATTTAACATCAGTCCTCTCAAAAGGAGAAATTCTAACACAATTTA    8027
LA2093      CTTGCTTGTCTCTCCATTTAACATCAGTCCTCTCAAAAGGAGAAGTTCTAACACAATTTA    8039
            ******************************************** ***************

E42         TTGCTGTTCTAAGAGAAAGACATTCACGACTAGGTTTTCTAAGCAGTATCTTAATTGTGT    8092
Heinz       TTGCTGTTCTAAGAGAAAGACATTCACGACTAGGTTTTCTAAGCAGTATCTTAATTGTGT    8087
LA2093      TTGCTGTTCTAAGAGAAAGACATTCACGACTAGGTTTTCTAAGCAGTATCTTAATTGTGT    8099
            ************************************************************

E42         TATAACCCAACTGAGCAATAATTGGCATTATTCTGTTATTTCAGAAATTCAACAAAACTT    8152
Heinz       TATAACCCAACTGAGCAATAATTGGCATTATTCTGTTATTTCAGAAATTCAACAAAACTT    8147
LA2093      TATAACCCAACTGAGCAATAATTGGCATTATTCTGTTATTTCAGAAATTCAACAAAACTT    8159
            ************************************************************

E42         TCAACATTAAAACATATCTCTGGGTTTCACACAGTACTGAGAGATTGAGCTAGCACAGGC    8212
Heinz       TCAACATTAAAACATATCTCTGGGTTTCACACAGTACTGAGAGATTGAGCTAGCACAGGC    8207
LA2093      TCAACATTAAAACATATCTCTGGGTTTCACACAGTACTGAGAGATTGAGCTAGCACAGGC    8219
            ************************************************************

E42         TTCTTTGTGAGATCGATAACTAAGCGATCACTGAATTCTGGTAAATTATTTTTTTCTTTA    8272
Heinz       TTCTTTGTGAGATCGATAACTAAGCGATCACTGAATTCTGGTAAATTATTTTTTTCTTTA    8267
LA2093      TTCTTTGTGAGATCGATAACTAAGCGATCACTGAATTCTGGTAAATTATTTTTTTCTTTA    8279
            ************************************************************

E42         GAGTGGGCAAAATTAGTTTAAGAGATGATCTATGCAGGATTAATTACTCAACAAAGAATG    8332
Heinz       GAGTGGGCAAAATTAGTTTAAGAGATGATCTATGCAGGATTAATTACTCAACAAAGAATG    8327
LA2093      GAGTGGGCAAAATTAGTTTAAGAGATGATCTATGCAGGATTAATTACTCAACAAAGAATG    8339
            ************************************************************

E42         ATACCAAGCAGCCACTATGAGTGGTTTTTATAGAAGGTAGGGGTTTAAGTGGTCAATTAA    8392
Heinz       ATACCAAGCAGCCACTATGAGTGGTTTTTATAGAAGGTAGGGGTTTAAGTGGTCAATTAA    8387
LA2093      ATACCAAGCAGCCACTATGAGTGGTTTTTATAGAAGGTAGGGGTTTAAGTGGTCAATTAA    8399
            ************************************************************

E42         CTCAAAAGTAATACTCTCTCAAATGAAACTACTTAAAACCTATGGAACTTCTGTCAACAT    8452
Heinz       CTCAAAAGTAATACTCTCTCAAATGAAACTACTTAAAACCTATGGAACTTCTGTCAACAA    8447
LA2093      CTCAAAAGTAATACTCTCTCAAATGAAACTACTTAAAACCTATGGAACTTCTGTCAACAT    8459
            ***********************************************************

E42         AGGTTGTAAAAGATTACATAGTACATATGAGTAGCATGTCCCGAACACCAATATTTAGGT    8512
Heinz       AGGTTGTAAAAGATTACATAGTACATATGAGTAGCATGTCCCGAACACCAATATTTAGGT    8507
LA2093      AGGTTGTAAAAGATTACATAGTACATATGAGTAGCATGTCCCGAACACCAATATTTAGGT    8519
            ************************************************************

E42         AAAGGTATCAATAGATCTCTCTCTCTGAACAATTAAGTGTTCTCTCAAGATTCACTTTTT    8572
Heinz       AAAGGTATCAATGGATCTCTCTCTCTGAACAATTAAGTGTTCTCTCAAGATTCACTTTTT    8567
LA2093      AAAGGTATCAATAGATCTCTCTCTCTGAACAATTAAGTGTTCTCTCAAGATTCACTTTTT    8579
            ************ ***********************************************

E42         AGGAAGAAAATTTTAAGCTTGGTCTTTTAATGGACTCACAGAGCTTCTTGGGGTCAAGGT    8632
Heinz       AGGAAGAAAATTTTAAGCTTGGTCTTTTAATGGACTCACAGAGCTTCTTGGGGTCAAGGT    8627
```

```
LA2093      AGGAAGAAAATTTTAAGCTTGGTCTTTTAATGGACTCACAGAGCTTCTTGGGGTCAAGGT    8639
            ************************************************************

E42         GTTGATACAGTAATGAGTTCACTGCCAAGGATGTAAGGACTGAAGGAGAGAGATTTCAAC    8692
Heinz       GTTGATACAGTAATGAGCTCACTGCCAAGGATGTAAGGACTGAAGGAGAGAGATTTCA--    8685
LA2093      GTTGATACAGTAATGAGTTCACTGCCAAGGATGTAAGGACTGAAGGAGAGAGATTTCAAC    8699
            ***************** ****************************************  

E42         TAGAATCCCCATCGTCCAGCAAACCTCCTTCGATTTAAGTTAAGAGTAAGACAAACCTAA    8752
Heinz       --AAATCCCCATCGTCCAGCAAACCTCCTTCGATTTAAGTTAAGAGTAAGACAAACCTAA    8743
LA2093      TAGAATCCCCATCGTCCAGCAAACCTCCTTCGATTTAAGTTAAGAGTAAGACAAACCTAA    8759
              **********************************************************

E42         AGCAGTCATACAGACAGAGAGAGAAAGAGTAAAGAACCAAGAAGTAAAGGCCATTAACAG    8812
Heinz       AGCAGTCATACAGACAGAGAGAGAAAGAGTAAAGAACCAAGAAGTAAAGGCAATTAACAG    8803
LA2093      AGCAGTCATACAGACAGAGAGAGAAAGAGTAAAGAACCAAGAAGTAAAGGCCATTAACAG    8819
            *************************************************** ********

E42         GAAAGCAATCTCTCATCTGCAATGGAAAATTCTTCCTTATCTCTCTCTTTACTATTTCTT    8872
Heinz       GAAAGCAATCTCTCATCTGCAATGGAAAATTCTTCCTTATCTCTCTCTTTACTATTTCTT    8863
LA2093      GAAAGCAATCTCTCATCTGCAATGGAAAATTCTTCCTTATCTCTCTCTTTACTATTTCTT    8879
            ************************************************************

E42         TTGTTTGTATAAGAGAAGAAGACTTATAAGCACAAAAGTGCATTAATGTAATAAGTAAAA    8932
Heinz       TTGTTTGTATAAGAGAAGAAGACTTATAAGCACAAAAGTGCATTAATGTAATAAGTAAAA    8923
LA2093      TTGTTTGTATAAGAGAAGAAGACTTATAAGCACAAAAGTGCATTAATGTAATAAGTAAAA    8939
            ************************************************************

E42         ACAGTAAAATATCCACAAGGAACTAAATAATCTAAGCAGTAAATTCTATAAACTAATGCA    8992
Heinz       ACAGTAAAATATCCACAAGGAACTAAATAATCTAAGCAGTAAATTCTATAAACTAATGCA    8983
LA2093      ACAGTAAAATATCCACAAGGAACTAAATAATCTAAGCAGTAAATTCTATAAACTAATGCA    8999
            ************************************************************

E42         AGGTACCTATCGGGGGACTTCTTCACCTTAAACACAATCACATGCTAATGAACTATCATG    9052
Heinz       AGGTACCTATCGGGGGACTTCTTCACCTTAAACACAATCACATGCTAATGAACTATCATG    9043
LA2093      AGGTACCTATCGGGGGACTTCTTCACCTTAAACACAATCACATGCTAATGAACTATCATG    9059
            ************************************************************

E42         AACATGTGAGTCAATAAAGGATTTGCACGTAAAGTATAATATCTCAAGGTCAGAGTGTCA    9112
Heinz       AACATGTGAGTCAATAAAGGATTTGCACGTAAAGCATAATATCTCAAGGTCAGAGTGTCA    9103
LA2093      AACATGTGAGTCAATAAAGGATTTGCACGTAAAGTATAATATCTCAAGGTCAGAGTGTCA    9119
            ********************************** *************************

E42         TTCCAGACTTCCAACAGTAACTGAAGTTGGACTAAAAGCAACTCTCTCCACAATGAGAAA    9172
Heinz       TTCCAGACTTCCAACAGTAACTGAAGTTGGACTAAAAGCAACTCTCTCCACAATGAGAAA    9163
LA2093      TTCCAGACTTCCAACAGTAACTGAAGTTGGACTAAAAGCAACTCTCTCCACAATGAGAAA    9179
            ************************************************************

E42         CCGAACTTGCTCCCATCCTTAACTATGTACTAATATCTGTCTTCCAATGTAGTACAAAAC    9232
Heinz       CCAAACTTGCTCCCATCCTTAACTATGTACTAATATCTGTCTTCCAATGTAGTACAAAAC    9223
LA2093      CCGAACTTGCTCCCATCCTTAACTATGTACTAATATCTGTCTTCCAATGTAGTACAAAAC    9239
            ** *********************************************************

E42         TCATCACCTGATATAGAGCATGCTTTCGGATCTTGATGGAGAATCCACTATATTATCTGG    9292
Heinz       TCATCACCTGATATAGAGCATGCTTTCGGATCTTGATGGAGAATCCACTATATTATCTGG    9283
LA2093      TCATCACCTGATATAGAGCATGCTTTCGGATCTTGATGGAGAATCCACTATATTATCTGG    9299
            ************************************************************

E42         TAGGGTGTCTCCCACTGTCAATCCATTTAAACTTGATGCCATTATCTGCAAAATTCCGCA    9352
Heinz       TAGGGTGTCTCCCACTGTCAATCCATTTAAACTTGATGCCATTATCTGCAAAATTCCGCA    9343
LA2093      TAGGGTGTCTCCCACTGTCAATCCATTTAAACTTGATGCCATTATCTGCAAAATTCCGCA    9359
            ************************************************************

E42         AGTTACTTCAGAAAGCCGAAGAAATTAGCACATGAAGGCTGAAATCACAGCCAAAGAACA    9412
Heinz       AGTTACTTCAGAAAGCCGAAGAAATTAGCACATGAAGGCTGAAATCACAGCCAAAGAACA    9403
LA2093      AGTTACTTCAGAAAGCCGAAGAAATTAGCACATGAAGGCTGAAATCACAGCCAAAGAACA    9419
            ************************************************************

E42         TGCATTTCCGTTGATAGCAACAACAACAAACGCAATTTCAATAATTATATTTAACATAAG    9472
Heinz       TGCATTTCCGTTGATAGCAACAACAACAAACGCAATTTCAATAATTATATTTAACATAAG    9463
LA2093      TGCATTTCCGTTGATAGCAACAACAACAAACGCAATTTCAATAATTATATTTAACATAAG    9479
            ************************************************************
```

```
E42         AATCTACAATAACATTTACACACTTTGTTGACATTATTAAGAAAGCACCATCTTTTAAAA    9532
Heinz       AATCTACAATAACATTTACACACTTTGTTGACATTATTAAGAAAGCACCATCTTTTAAAA    9523
LA2093      AATCTACAATAACATTTACACACTTTGTTGACATTATTAAGAAAGCACCATCTTTTAAAA    9539
            ************************************************************

E42         AGTCTATCAGCCACATCTCAATCCTAAACTAGTTGTAATTAACTATATTTATCCCACCTG    9592
Heinz       AGTCTATCAGCCACATCTCAATCCTAAACTAGTTGTAATTAACTATATTTATCCCACCTG    9583
LA2093      AGTCTATCAGCCACATCTCAATCCTAAACTAGTTGTAATTAACTATATTTATCCCACCTG    9599
            ************************************************************

E42         CTCTGCTCAAATCCGTTTCATTCTAAAAACGCAATTCAATAATGTAAGAAATTTAAACCC    9652
Heinz       CTCTGCTCAAATCCGTTTCATTCTAAAAACGCAATTCAATAATGTAAGAAATTTAAACCC    9643
LA2093      CTCTGCTCAAATCCGTTTCATTCTAAAAACGCAATTCAATAATGTAAGAAATTTAAACCC    9659
            ************************************************************

E42         TCGAAAGGTCTACAAACTTGAAATTGTATGTTAACAAAATCGGCTCTAATTCCACATACA    9712
Heinz       TCGAAAGGTCTACAAACTTGAAATTGTATGTTAACAAAATCGGCTCTAATTCCACATACA    9703
LA2093      TCGAAAGGTCTACAAACTTGAAATTGTATGTTAACAAAATCGGCTCTAATTCCACATACA    9719
            ************************************************************

E42         CATTTCAACAACATAAAAAGATTAGATTTTGAGAAGGGGCAAAACCTCACTAAGATAACG    9772
Heinz       CATTTCAACAACATAAAAAGAGTAGATTTTGAGAAGGGGCAAAACCTCACTAAGATAACG    9763
LA2093      CATTTCAACAACATAAAAAGATTAGATTTTGAGAAGGGGCAAAACCTCACTAAGATAACG    9779
            ********************* **************************************

E42         TTTGTGAGAGTGAAGATTATCGACATAAACCTCGTCTTCTGGGTCTCTATTTCTCTTACC    9832
Heinz       TTTGTGAGAGTGAAGATTATCGACATAAACCTCGTCTTCTGGGTCTCTATTTCTCTTACC    9823
LA2093      TTTGTGAGAGTGAAGATTATCGACATAAACCTCGTCTTCTGGGTCTCTATTTCTCTTACC    9839
            ************************************************************

E42         AGTACCAGTTGGGGACGATGTTGCTGATGATGACGATGCCGCTGCTGCTGTTGCGATCGC    9892
Heinz       AGTACCAGTTGGGGACGATGTTGCTGATGATGACGATGCCGCTGCTGCTGTTGCGATCGC    9883
LA2093      AGTACCAGTTGGGGACGATGTTGCTGATGATGACGATGCCGCTGCTGCTGTTGCGATCGC    9899
            ************************************************************

E42         CGGAGCAGACGACGGCGATGAACCCGTCTCCGAGCCCATATTATAAAAGAGAATAAAAAA    9952
Heinz       CGGAGCAGACGACGGCGATGAACCCGTCTCCGAGCCCATATTATAAAAGAGAATAAAAAA    9943
LA2093      CGGAGCAGACGACGGCGATGAACCCGTCCCCGAGCCCATATTATAAAAGAGAATAAAAAA    9959
            **************************** *******************************

E42         ATCAAATCCCTTTTCTCTCGAGTTCAATGTCAGAGTGAAAAAGTAGAAAACCGATTAAGC    10012
Heinz       ATCAAATCCCTTTTCTCTCGAGTTCAATGTCAGAGTGAAAAAGTAGAAAACCGATTAAGC    10003
LA2093      ATCAAATCCCTTTTCTCTCGAGTTCAATGTCAGAGTGAAAAAGTAGAAAACCGATTAAGC    10019
            ************************************************************

E42         ATTAAACCAAAAGTTAACGGCTTTAATTATCCAAAAAAGTATGAAATTATTATTTTTTTT    10072
Heinz       ATTAAACCAAAAGTTAACGGCTTTAATTATCCAAAAAAGTATGAAATTATTATTTTTTTT    10063
LA2093      ATTAAACCAAAAGTTAACGGCTTTAATTATCCAAAAAAGTATGAAATTATTATTTTTTTT    10079
            ************************************************************

E42         CACCATAGGTTGACAATATGCCAGGTCAGTCTCACTTGTTAGAGCCGGGTC      10123
Heinz       CACCATAGGTTGACAATATGCCAGGTCAGTCTCACTTGTTAGAGCCGGGTC      10114
LA2093      CACCATAGGTTGACAATATGCCAGGTCAGTCTCACTTGTTAGAGCCGGGTC      10130
            ***************************************************
```

Solyc01g086740

```
E42         GCCTAATATCTAATTAGGCCCTGTGAAAGGGCTTCCACAAAAGAAGGAGCCCCTGATACT    60
HEINZ       GCCTAATATCTAATTAGGCCCTGTGAAAGGGCTTCCACAAAAGGAGGAGCCCCTGATACT    60
LA2093      GCCTAATATCTAATTAGGCCCTGTGAAAGGGCTTCCACAAAAGGAGGAGCCCCTGATACT    60
            ******************************************* ****************

E42         ACTTCCGCCATTAAACTAGAACCTTCCATTGCAAAGCTCGCAAAGAAGCTTGAGGCTTGT    120
HEINZ       ACTTCCGCCATTAAACTAGAACCTTCCATTGCAAAGCTCGCAAAGAAGCTTGAGGCTTGT    120
LA2093      ACTTCCGCCATTAAACTAGAACCTTCCATTGCAAAGCTCGCAAAGAAGCTTGAGGCTTGT    120
            ************************************************************

E42         GTGGGTAGACGTCTATATTCACTATCAAAACCCCATCATTTGAAAA-AAAAAATCAATCT    179
HEINZ       GTGGGTAGACGTCTATATGCTCTATCGAAACCCCATCAATTGAAAAA-AAAAATCAATCT    179
LA2093      GTGGGTAGACGTCTATATGCTCTATCGAAACCCCATCAATTGAAAAAAAAAAAATCAATCT   180
            ****************** * ***** *********** *******  ************

E42         TTTTTCACTTGACAAACTAGGGTTTTTCTAGGGTTTATTATAGTCGAGAACTGTTTTGCT    239
HEINZ       TTTTTCACTTGACAAACTAGGGTTTTTCTAGGGTTTATTATAGTCGAGAACTGTTTTGCT    239
LA2093      TTTTTCACTTGACAAACTAGGGTTTTTCTAGGGTTTATTATAGTCGAGAACTGTTTTGCT    240
            ************************************************************

E42         TTAGCTTTTTTCAGTGTTACTAAAATGGTCAGCTCAAATGGTCTTCGTCTTGCTCACCGT    299
HEINZ       TTAGCTTTTTTCAGTGTTACTAAAATGGTCAGCTCAAATGGTCTTCGTCTTGCTCACCGT    299
LA2093      TTAGCTTTTTTCAGTGTTACTAAAATGGTCAGCTCAAATGGTCTTCGTCTTGCTCACCGT    300
            ************************************************************

E42         CTTGCTCGCTGCACCTTTTCTTCTGTATTGTCCTCTCCTTCTTTTATCTCAATCGCTCTC    359
HEINZ       CTTGCTCGCTGCACCTTTTCTTCTGTATTATCCTCTC---CTTTTATCTCAGTCGCTCTC    356
LA2093      CTTGCTCGCTGCACCTTTTCTTCTGTATTGTCCTCTCCTTCTTTTATCTCAGTCGCTCTC    360
            ***************************** *******   *********** ********

E42         TGCATTTTTTTCTTTTTTGTATTGATGTGTGAGCAATGTTGAATTTGTAGACTACTGGAT    419
HEINZ       TGCATTTTTTTCTTTTTTGTATTGATGTGTGAGCAATGTTGAATTTGTAGACTACTGGAT    416
LA2093      TGCATTTTTTTCTTTTTTGTATTGATGTGTGAGCAATGTTGAATTTGTAGACTACTGGAT    420
            ************************************************************

E42         CTTTGTTGAAGGAGGGTTGTAGGGGATATAACACGGCAGTATGCAATCAAACAAGGGGAT    479
HEINZ       CTTTGTTGAAGGAGGGTTGTAGGGGATATAACACGGCAGTATGCAATCAAACAAGGGGAT    476
LA2093      CTTTGTTGAAGGAGGGTTGTAGGGGATATAACACGGCAGTATGCAATCAAACAAGGGGAT    480
            ************************************************************

E42         TGTTTTATTTCAATTCAAAGAATGGTATGTATGAATTGACCTTCTGTAATTTATTTGCTT    539
HEINZ       TGTTTTATTTCAATTCAAAGAATGGTATGTATGAATTGACCTTCTGTAATTTATTTGCTT    536
LA2093      TGTTTTATTTCAATTCAAAGAATGGTATGTATGAATTGACCTTCTGTAATTTATTTGCTT    540
            ************************************************************

E42         AAAAAAACTTGAATTGAATTTTTGATATTGTGAACTCATAGGGGGCAGCGAAAGGGATTG    599
HEINZ       AAAAAAACTTGAATTGAATTTTTGATATTGTGAACTCATAGGGGGCAGCGAAAGGGATTG    596
LA2093      AAAAAAACTTGAATTGAATTTTTGATATTGTGAACTCATAGGGGGCAGCGAAAGGGATTG    600
            ************************************************************

E42         GTTAAGACTTGGGCAATTCAAATCCAATTTTGGTGCCACAAGATCAATTCATGGAACAGG    659
HEINZ       GTTAAGACTTGGGCAATTCAAATCCAATTTTGGTGCCACAAGATCAATTCATGGAACAGG    656
LA2093      GTTAAGACTTGGGCAATTCAAATCCAATTTTGGTGCCACAAGATCAATTCATGGAACAGG    660
            ************************************************************

E42         TACCAGTTAACCACTCCCTTCCGAGTATTGAGCTTCCGTTATCTCGCTTCCGTATATGGC    719
HEINZ       TACCAGTTAACCACTCCCTTCCGAGTATTGAGCTTCCGTTATCTCGCTTCCGTATATGGC    716
LA2093      TACCAGTTAACCACTCCCTTCCGAGTATTGAGCTTCCGTTATCTCGCTTCGGTATATGGC    720
            ************************************************** *********

E42         CGGTTGCGGGTTATGTCACTCTATTAGTTGCTTCATTTGATAATCTTGAGGAATTATCTC    779
HEINZ       TGGTTGCGGGTTATGTCACTCTATTAGTTGCTTGATTTGATAATCTTGAGGAATTATCTC    776
LA2093      CGGTTGCGGGTTATGTCACTCTATTAGTTGCTTCATTTGATAATCTTGAGGAATTATCTC    780
             ******************************** **************************

E42         TTTTATGTTAGTCAGTTAGTCTACTGCTCTACTTGAATGAGGTAAATTTTAGGTCTTTAT    839
HEINZ       TTTTATGTTAGTCAGTTAGTCTACTGCTCTACTTGAATGAGGTAAATTTTAGGTCTTTAT    836
LA2093      TTTTATGTTAGTCAGTTAGTCTACTGCTCTACTTGAATGAGGTAAATTTTAGGTCTTTAT    840
            ************************************************************
```

```
E42        GGACTCTAAATTGGAGTTCTTGTGTTAATTAGATATTTAATGGGTAACATATTTCTGTAG    899
HEINZ      GGACTCTAAATTGGAGTTCTTGTGTTAATTAGATATTTAATGGGTAACATATTTCTGTAG    896
LA2093     GGACTCTAAATTGGAGTTCTTGTGTTAATTAGATATTTAATGGGTAACATATTTCTGTAG    900
           ************************************************************

E42        ATTTTTCTCATTAGGCTAATCGGACCAGATAAAATACCTTGTTTTCCCAACATGTTCCTT    959
HEINZ      ATTTTTCTCATTAGGCTAATCGGACCAGATAAAATACCTTGTTTTCCCAACATGTTCCTT    956
LA2093     ATTTTTCTCATTAGGCTAATCGGACCAGATAAAATACCTTGTTTTCCCAACATGTTCCTT    960
           ************************************************************

E42        TTTATCAATCGATAGAACAGATTGTGGCAATCCAGCTATAATTTCTTCTGGAT---TTTA    1016
HEINZ      TTTATCAATCGATAGAACAGCTTGTGGCAATCCAGCTATAATTTCTTCTGGATGTGTTTA    1016
LA2093     TTTATCAATCGATAGAACAGATTGTGGCAATCCAGCTATAATTTCTTCTGGATGTGTTTA    1020
           ******************** ********************************   ****

E42        ATGTTATTTTTAGGGGTTAGTTTCATTAGGTAAATCGGTTGTTAGCTTGCTCTCCTTTGA    1076
HEINZ      ATGTTATTTTTAGGGGTTAGTTTCATTAGGTAAATCGGTTGTTAGCTTGCTCTCCTTTGA    1076
LA2093     ATGTTATTTTTAGGGGTTAGTTTCATTAGGTAAATCGGTTGTTAGCTTGCTCTCCTTTGA    1080
           ************************************************************

E42        CTCTTTTTCCATGTTAATTGAAATACATTTGGCCTCTAAAACCTGATGAGTACTCATTAT    1136
HEINZ      CTCTTTTTCCATGTTAATTGAAATACATTTGGCCTCTAAAACCTGATGAGTACTCATTAT    1136
LA2093     CTCTTTTTCCATGTTAATTGAAATACATTTGGCCTCTAAAACCTGATGAGTACTCATTAT    1140
           ************************************************************

E42        GCATGTTCATACAGACCGGTAAGACAGAGGTAGAAATTGCAAAACTTCCACGGCATCTCT    1196
HEINZ      GCATGTTCATACAGACCGGTAAGACAGAGGTAGAAATTGCAAAACTTCCACGGCATCTCT    1196
LA2093     GCATGTTCATACAGACCGGTAAGACAGAGGTAGAAATTGCAAAACTTCCACGGCATCTCT    1200
           ************************************************************

E42        TGGTTTCTTCTGTAAATTGCAAGTCTGCTACTATTTTCCAAGAAGAGGTAAAAAAAGCGG    1256
HEINZ      TGGTTTCTTCTGTAAATTGCAAGTCTGCTACTATTTTCCAAGAAGAGGTAAAAAAAGCGG    1256
LA2093     TGGTTTCTTCTGTAAATTGCAAGTCTGCTACTATTTTCCAAGAAGAGGTAAAAAAAGCGG    1260
           ************************************************************

E42        TACAGAAGCTCTAGCTAGCAGGCAGAGCTTGTTTATGCCTTCTATGGAGAATGATGGTAG    1316
HEINZ      TACAGAAGCTCTAGCTAGCAGGCAGAGCTTGTTTATGCCTTCTATGGAGAATGATGGTAG    1316
LA2093     TACAGAAGCTCTAGCTAGCAGGCAGAGCTTGTTTATGCCTTCTATGGAGAATGATGGTAG    1320
           ************************************************************

E42        CCCGATAAATAGGGTGTGTATGTGTGTATTTAGAGAGAGAGGGGGGACGGTGTTTGGGGG    1376
HEINZ      CCCGATAAATAGGGTGTGTATGTGTGTATTTAGAGAGAGAGGGGGGACGGTGTTTGGGGG    1376
LA2093     CCCGATAAATAGGGTGTGTATGTGTGTATTTAGAGAGAGAGGGGGGACGGTGTTTGGGGG    1380
           ************************************************************

E42        GGAGGGGGAGAACAGCTTGTGTATATTGGAAGAGGAAAGCTGATAGAAGAGACCGTCTAA    1436
HEINZ      GGAGGGGGAGAACAGCTTGTGTATATTGGAAGAGGAAAGCTGATAGAAGAGACAGTCTAA    1436
LA2093     GGAGGGGGAGAACAGCTTGTGTATATTGGAAGAGGAAAGCTGATAGAAGAGACAGTCTAA    1440
           ***************************************************** ******

E42        ATTTATTTTTCTTATTCCCTATGGTGCATTCTGAGTTTCTCTCAGAGAGTGGAGGTGAGG    1496
HEINZ      ATTTATTTTTCTTATTCCCTATGGTGCATTCTGAGTTTCTCTCAGAGAGTGGAGGTGAGG    1496
LA2093     ATTTATTTTTCTTATTCCCTATGGTGCATTCTGAGTTTCTCTCAGAGAGTGGAGGTGAGG    1500
           ************************************************************

E42        TGAAGATTAGTCACATCTACTGGTTTCTTCCTTTAAACCGGTAC---TCCTTTTTAATTG    1553
HEINZ      TGAAGATTAGTCACATCTACTGGTTTCTTCCTTTAAACCGGTATATCTCCTTTTTAATTG    1556
LA2093     TGAAGATTAGTCACATCTACTGGTTTCTTCCTTTAAACCGGTATATCTCCTTTTTAATTG    1560
           *******************************************    *************

E42        TGAAAAAAAAAGACTTTTAATTGCTCTCTGTTTTCCCCTCCAAAAAGCATTGTGCAGCCAG    1613
HEINZ      TGAAAAAAAAAGACTTTTAATTGCTCTCTGTTTTCCCCTCCAAAAAGCATTGTGCAGCCAG    1616
LA2093     TGAAAAAAAAAGACTTTTAATTGTTCTCTGTTTTCCCCTCCAAAAAGCATTGTGCAGCCAG    1620
           *********************** ************************************

E42        TGTAGAATGAGATCTGTTATTATGTATGCTAGTAGGTGAATTATTCTTTCTAAATAATAA    1673
HEINZ      TGTAGAATGAGATCTGTTATTATGTATGCTAGTAGGTGAGTTATTCTTTCTAAATAATAA    1676
LA2093     TGTAGAATGAGATCTGTTATTATGTATGCTAGTAGGTGAATTATTCTTTCTAAATAATAA    1680
           *************************************** ********************

E42        ATAAGTAAATTGTTGAAGTAACAACTTGATATAAAACTTTCCATCTCACAAAAAAGTAAA    1733
```

```
HEINZ      ATAAGTAAATTGTTGAAGTAACAACTTGATATAAAACTTTCCATCTCACAAAAAAGTAAA    1736
LA2093     ATAAGTAAATTGTTGAAGTAACAACTTGATATAAAACTTTCCATCTCACAAAAAAGTAAA    1740
           ************************************************************

E42        AATTGTGCTCTCAGGGCTATGGTCTAGTTGTAAGAGTGCAGCGTGCGTTTTGTGGGTTAA    1793
HEINZ      AATTGTGCTCTCAGGGCTATGGTCTAGTTGTAAGAGTGCAGCGTGCGTTTTGTGGGTTAA    1796
LA2093     AATTGTGCTCTCAGGGCTATGGTCTAGTTGTAAGAGTGCAGCGTGCGTTTTGTGGGTTAA    1800
           ************************************************************

E42        CCGCACGTCGTAAGTTCGAACCCTTCACTAGGCAAAAGTCAGGTATTTAACTGAAGAAGG    1853
HEINZ      CCGCACGTCGTAAGTTCGAACCCTTCACTAGGCAAAAGTCAGGTATTTAACTGAAGAAGG    1856
LA2093     CCGCACGTCGTAAGTTCGAACCCTTCACTAGGCAAAAGTCAGGTATTTAACTGAAGAAGG    1860
           ************************************************************

E42        GGTTAGAGGGGTGGGTCCATTATCCAATTTATATAAACGTTATCAAAAGTAACAAATTCC    1913
HEINZ      GGTTAGAGGGGTGGGTCCATTATCCAATTTATATAAACGTTATCAAAAGTAACAAATTCC    1916
LA2093     GGTTAGAGGGGTGGGTCCATTATCCAATTTATGTAAACGTTATCAAAAGTAACAAATTCC    1920
           ******************************* ****************************

E42        TGGTTGAAAATGTTTCATATAGGCTAAGTTGTGCGGACTCTTCACTTACATTGCCGCACC    1973
HEINZ      TGGTTGAAAATGTTTCATATAGGCTAAGTTGTGCGGACTCTTCACTTACATTGCCGCACC    1976
LA2093     TGGTTGAAAATGTTTCATATAGGCTAAGTTGTGCGGACTCTTCACTTACATTGCCGCACC    1980
           ************************************************************

E42        TGTGCCGGGTTCTTCAAAAATACACTACTTATGGAGAATCTGACATGCACATCTAGACAT    2033
HEINZ      TGTGCCGGGTTCTTCAAAAATACACTACTTGTGGAGAATCTGACACGCACATGTAGACAT    2036
LA2093     TGTGCCGGGTTCTTCAAAAATACACTACTTATGGAGAATCTGACACGCACATGTAGACAT    2040
           ****************************** ************** ****** *******

E42        TTTTGATGAGTCCGAGCAACATAGCATATAAGGCATGCTTTCATTATCTTGGTTTCCAGG    2093
HEINZ      TTTTGATGAGTCCGAGCAACATAGCATATAAGGCATGCTTTCATTATCTTGGTTTCCAGG    2096
LA2093     TTTTGATGAGTCCGAGCAACATAGCATATAAGGCATGCTTTCATTATCTTGGTTTCCAGG    2100
           ************************************************************

E42        ATGTGCAATGGTTACATCTTACTCTGTTGATAACATATATGCGACTCATTTACCCACTGT    2153
HEINZ      ATGTGCAATGGTTACATCTTACTCTGTTGATAACATATATGCGACTCATTTACCCACTGT    2156
LA2093     ATGTGCAATGGTTACATCTTACTCTGTTGATAACATATATGCGACTCATTTACCCACTGT    2160
           ************************************************************

E42        GAATGGTATATCTGTGTATTTGGAAGCGGATAAGATTTGTATTATAAAACATTGTTGTTC    2213
HEINZ      GAATGGTATATCTGTGTATTTGGAAGCGGATAAGATTTGTATTATAAAACATTGTTGTTC    2216
LA2093     TAATGGTATATCTGTGTATTTGGAAGCGGATAAGATTTGTATTATAAAACATTGTTGTTC    2220
            ***********************************************************

E42        ACTATGAATTGCTTCCCATATCTGTATATCTGGAAGCAGTTAATATTTGTATTGTAAAAC    2273
HEINZ      ACTATGAATTGCTTCCCATATCTGTATATCTGGAAGCAGTTAATATTTGTATTGTAAAAC    2276
LA2093     ACTATGAATTGCTTCCCATATCTGTATATCTGGAAGCAGTTAATATTTGTATTGTAAAAC    2280
           ************************************************************

E42        ATTATTGTTCACTGTGAAATGCTTTCCATATGATGTTTCATCTTGGAAATTTTGGTGGTA    2333
HEINZ      ATTATTGTTCACTGTGAAATGCTTTCCATATGATGTTTCATCTTGGAAATTTTGGTGGTA    2336
LA2093     ATTATTGTTCACTATGAAATGCTTTCCATATGATGTTTCATCTTGGAAATTTTGGTGGTA    2340
           ************* **********************************************

E42        ATCTTTCAAAGTGGTAATGGAGGTATTTGTTAGAATTAGTTTTTGATTCTCTCGTATGTA    2393
HEINZ      ATCTTTCAAAGTGGTAATGGAGGTATTTGTTAGAATTAGTTTTTGATTCTCTCGTATGTA    2396
LA2093     ATCTTTCAAAGTGGTAATGGAGGTATTTGTTAGAATTAGTTTTTGATTCTCTCGTATGTA    2400
           ************************************************************

E42        TTTTCATATTAGACTGGTTATGTATTTTAACTAACATAACCTCTTCGCTGTTCAATGGTT    2453
HEINZ      TTTTCATATTAGACTGGTTATGTATTTTAACTAACATAACCTCTTCGCTGTTCAATGGTT    2456
LA2093     TTTTCATATTAGACTGGTTATGTATTTTAACTAACATAACCTCTTCGCTGTTCAATGGTT    2460
           ************************************************************

E42        GGTTAGGCATGAGTATGAAAGATTTTTATGAGGTTCTTGGCGTCAATCGGAATGCAACTG    2513
HEINZ      GGTTAGGCATGAGTATGAAAGATTTTTATGAGGTTCTTGGCGTCAATCGGAATGCAACTG    2516
LA2093     GGTTAGGCATGAGTATGAAAGATTTTTATGAGGTTCTTGGCGTCAATCGGAATGCAACTG    2520
           ************************************************************

E42        CCTCCGAAATAAAAAAGGCTTATTATGGGGTATGTTATCATCCCCTTCTGGATTAGTTAA    2573
HEINZ      CCTCCGAAATAAAAAAGGCTTATTATGGGGTATGTTATCATCCCCTTCTGGATTAGTTAA    2576
LA2093     CCTCCGAAATAAAAAAGGCTTATTATGGGGTATGTTATCATCCCCTTCTGGATTAGTTAA    2580
```

```
            ************************************************************

E42         TATTGTTTGCAAATATAAGATTGGCTAAGTTCTAGTCAAGGAGGACTTTGTAATGACTGA    2633
HEINZ       TATTGTTTGCAAATATAAGATTGGCTAAGTTCTAGTCAAGGAGGACTTTGTAATGACTGA    2636
LA2093      TATTGTTTGCAAATATAAGATTGGCTAAGTTCTAGTCAAGGAGGACTTTGTAATGACTGA    2640
            ************************************************************

E42         ACTAGCATCTTGCAATTAAAAGTTTCTGGTGTTAGTATCACCTTATCTTTGTTACTGATT    2693
HEINZ       ACTAGCATCTTGCAATTAAAAGTTTCTGGTGTTAGTATCACCTTATCTTTGTTACTGATT    2696
LA2093      ACTAGCATCTTGCAATTAAAAGTTTCTGGTGTTAGTATCACCTTATCTTTGTTACTGATT    2700
            ************************************************************

E42         ATACATTCGCTATGCTTAGCTTGCAAAGCAACTGCATCCAGATATGAACAAAGATGACCC    2753
HEINZ       ATACATTCGCTATGCTTAGCTTGCAAAGCAACTGCATCCAGATATGAACAAAGATGACCC    2756
LA2093      ATACATTCGCTATGCTTAGCTTGCAAAGCAACTGCATCCAGATATGAACAAAGATGACCC    2760
            ************************************************************

E42         AGATGCTGAAAAAAAGTTTCAAGAAGTCTCAAAGGCTTATGAGGTACTAGCCACTTTGGA    2813
HEINZ       AGATGCTGAAAAAAAGTTTCAAGAAGTCTCAAAGGCTTATGAGGTACTAGCCACTTTGGA    2816
LA2093      AGATGCTGAAAAAAAGTTTCAAGAAGTCTCAAAGGCTTATGAGGTACTAGCCACTTTGGA    2820
            ************************************************************

E42         GAAGTTTTTTGCTCTGTAAAGAAGTTATTGCTGAAGCCATTACTGTATGGAATTTTGGAT    2873
HEINZ       GAAGTTTTTTGCTCTGTAAAGAAGTTATTGCTGAAGCCATTACTGTATGGAATTTTGGAT    2876
LA2093      GAAGTTTTTTGCTCTGTAAAGAAGTTATTGCTGAAGCCATTACTGTATGGAATTTTGGAT    2880
            ************************************************************

E42         GACCAATTGTTTGTTCATCTATGTGGCAAATGCAGGTTTTGAAGGATGATAGTACACGTG    2933
HEINZ       GACCAATTGTTTGTTCATCTATGTGGCAAATGCAGGTTTTGAAGGATGATAGTACACGTG    2936
LA2093      GACCAATTGTTTGTTCATCTATGTGGCAAATGCAGGTTTTGAAGGATGATAGTACACGTG    2940
            ************************************************************

E42         AACAATATGATCAGGTTTGACATGCCTTATGGGTTACTTTGCACATGAACCTGGCAAACA    2993
HEINZ       AACAATATGATCAGGTTTGACATGCCTTATGGGTTACTTTGCACATGAACCTGGCAAACA    2996
LA2093      AACGATATGATCAGGTTTGACATGCCTTATGGGTTACTTTGCACATGAACCTGGCAAACA    3000
            *** ********************************************************

E42         TTTGGTTCTCTGTTTGTTTTTATTTGCATATTAGGGGTTTATTTGCCTATAAACATTCAT    3053
HEINZ       TTTGGTTCTCTGTTTGTTTTTATTTGCATATTAGGGGTTTATTTGCCTATAAACATTCAT    3056
LA2093      TTTGGTTCTCTGTTTGTTTTTATTTGCATATTAGGGGTTTATTTGCCTATAAACATTCAT    3060
            ************************************************************

E42         GACTTTGATTATGTTTCCTCTTGAATGAGAGGCATTCTGATGCATTGTTGAATATACAGC    3113
HEINZ       GACTTTGATTATGTTTCCTCTTGAATGAGAGGCATTCTGATGCATTGTTGAATATGCAGC    3116
LA2093      GACTTTGATTATGTTTCCTCTTGAATGAGAGGCATTCTGATGCATTGTTGAATATGCAGC    3120
            ******************************************************* ****

E42         TTGGGCATGATGCATTTAATAATATGAACAGTGGTGGAGGTGGAGGTCCTGGATTTGATC    3173
HEINZ       TTGGGCATGATGCATTTAATAATATGAACAGTGGTGGAGGTGGAGGTCCTGGATTTGATC    3176
LA2093      TTGGGCATGATGCATTTAATAATATGAACAGTGGTGGAGGTGGAGGTCCTGGATTTGATC    3180
            ************************************************************

E42         CATTTGGTGGTTTCAAAAGTCCATTTGAAGACATGTTTAAAAATGCTGATGTAAGGACCT    3233
HEINZ       CATTTGGTGGTTTCAAAAGTCCATTTGAAGACATGTTTAAAAATGCTGATGTAAGGACCT    3236
LA2093      CATTTGGTGGTTTCAAAAGTCCATTTGAAGACATGTTTAAAAATGCTGATGTAAGGACCT    3240
            ************************************************************

E42         TATCTCCTGGTTTAGTTCTCTGATAATTCATTTGCATTGGAAAATTTCTATGGTGGTATT    3293
HEINZ       TCCTG----GTTTAGTTCTCTGATAATTCATTTGCATTGGAAAATTTCTATGGTGGTATT    3292
LA2093      TATCC----TGGTTTAGTTCTGATAATTCATTTGCATTGGAAAATTTCTATGGTGGTATT    3296
            *           *     ******************************************

E42         CTAGATTTTTACTTTTTTCTCTAGCTGTCTGTTAAGCATTGAATTCTACTAATTCACCTC    3353
HEINZ       CTAGATTTTTACTTTTTTCTCTAGCTGTCTGTTAAGCATTGAATTCTACTAATTCACCTC    3352
LA2093      CTAGATTTTTACTTTTTTCTCTAGCTGTCTGTTAAGCATTGAATTCTACTAATTCACCTC    3356
            ************************************************************

E42         TGATGTTGGCCTCTACAAAAAGTTGGTTCTTGAGCTAGATACAAAGTTTAGTAATGTGCT    3413
HEINZ       TGATGTTGGCCTCTACAAAAAGTTGGTTCTTGAGCTAGATACAAAGTTTAGTAATGTGCT    3412
LA2093      TGATGTTGGCCTCTACAAAAAGTTGGTTCTTGAGCTAGATACAAAGTTTAGTAATGTGCT    3416
            ************************************************************
```

```
E42             CTTCATGAGAGAGGTCCTCTGGTGAATTGATATGACCCTCTGTTCTCTCTTCCATAGATA     3473
HEINZ           CTTCATGAGAGAGGTCCTCTGGTGAATTGATATGACCCTCTGTTCTCTCTTCCATAGATA     3472
LA2093          CTTCATGAGAGAGGTCCTCTGGTGAATTGATATGACCCTCTGTTCTCTCTTCCATAGATA     3476
                ************************************************************

E42             TTTCAAGAGATTATTGGTTGTGTTTTTGCTAAACAGTATCACAGCCGGCATTAGAGTGTG     3533
HEINZ           TTTCAAGAGATTATTGGTTGTGTTTTTGCTAAACAGTATCACAGCCGGCATTAGAGTGTG     3532
LA2093          TTTCAAGAGATTATTGGTTGTGTTTTTGCTAAACAGTATCACAGCCGGCATTAGAGTGTG     3536
                ************************************************************

E42             TGCGCACCTGATTGCCAAACAGTATCACACCCTCTGTTCTCTCCTTTCCATGAATTTTAG     3593
HEINZ           TGCGCACCTGATTGCCAAACAGTATCACACCCTCTGTTCTCTCCTTTCCATGAATTTTAG     3592
LA2093          TGCGCACCTGATTGCCAAACAGTATCACACCCTCTGTTCTCTCCTTTCCATGAATTTTAG     3596
                ************************************************************

E42             CCCCATTTATAGAAAGCTATTTGAGTCTATGACGTTTGAGGGAGTTTGTAGAAGGATAGA     3653
HEINZ           CCCCATTTATAGAAAGCTATTTGAGTCTATGACGTTTGAGGGAGTTTGTAGAAGGATAGA     3652
LA2093          CCCCATTTATAGAAAGCTATTTGAGTCTATGACGTTTGAGGGAGTTTGTAGAAGGATAGA     3656
                ************************************************************

E42             GTCTACAAAGACAAATATTTGTGAAAGTTACCATTATCAAAAGAAAAAAAGCCAAATGTT     3713
HEINZ           GTCTACAAAGACAAATATTTGTGAAAGTTACCATTATCAAAAGAAAAAAAGCCAAATGTT     3712
LA2093          GTCTACAAAGACAAATATTTGTGAAAGTTACCATTATCAAAAGAAAAAAAGCCAAATGTT     3716
                ************************************************************

E42             TGTGAAGAGGTACTTGTTCGGCAGCGAATTAAGTATTACTCCCCTCTATACCAATTTATG     3773
HEINZ           TGTGAAGAGGTACTTGTTCGGCAGCGAATTAAGTATTACTCCCCTCTATACCAATTTATG     3772
LA2093          TGTGAAGAGGTACTTGTTCGGCAGCGAATTAAGTATTACTCCCCTCTATACCAATTTATG     3776
                ************************************************************

E42             TGGCACTGTTTGACTGGCGATTGAATTTATGGAAGAAGTGAAGACTTTTGGAACTTGTAG     3833
HEINZ           TGGCACTGTTTGACTGGCGATTGAATTTATGGAAGAAGTGAAGACTTTTGGAACTTGTAG     3832
LA2093          TGGCACTGTTTGACTGGCGATTGAATTTATGGAAGAAGTGAAGACTTTTGGAACTTGTAG     3836
                ************************************************************

E42             TCTAAAACAAGCCTTAGACATTTGTGTGGCTATAAATAATCTCTTTAATGGTAGAATGAG     3893
HEINZ           TCTAAAACAAGCCTTAGACATTTGTGTGGCTATAAATAATCTCTTTAATGGTAGAATGAG     3892
LA2093          TCTAAAACAAGCCTTAGACATTTGTGTGGCTATAAATAATCTCTTTAATGGTAGAATGAG     3896
                ************************************************************

E42             ATTTCAAACAACTTTTTTGGGACATACTAAAATGTAAATTGGGACAGAAAGTAACAAACA     3953
HEINZ           ATTTCAAACAACTTTTTTGGGACATACTAAAATGTAAATTGGGACAGAAAGTAACAAACA     3952
LA2093          ATTTCAAACAACTTTTTTGGGACATACTAAAATGTAAATTGGGACAGAAAGTAACAAACA     3956
                ************************************************************

E42             CAAATATTGGAAAACACAGGAGTAATAAATAGCTGATGTCATTTTTACCTATTGAGATGT     4013
HEINZ           CAAATATTGGAAAACACAGGAGTAATAAATAGCTGATGTCATTTTTACCTATTGAGATGT     4012
LA2093          CAAATATTGGAAAACACAGGAGTAATAAATAGCTGATGTCATTTTTACCTATTGAGATGT     4016
                ************************************************************

E42             TCGATGAGGTACTGTGAAAACAGATGCTTGATAGCTCTTCTTAACCTCTTAGGCATCAAC     4073
HEINZ           TCGATGAGGTACTGTGAAAACAGATGCTTGATAGCTCTTCTTAACCTCTTAGGCATCAAC     4072
LA2093          TCGATGAGGTACTGTGAAAACAGATGCTTGATAGCTCTTCTTAACCTCTTAGGCATCAAC     4076
                ************************************************************

E42             TTTTATGGTCTCTTCAGATGCATCACGACTGTAAAAGCTTAAACGTGATTTTATGCTTAA     4133
HEINZ           TTTTATGGTCTCTTCAGATGCATCACGACTGTAAAAGCTTAAACGTGATTTTATGCTTAA     4132
LA2093          TTTTATGGTCTCTTCAGATGCATCACGACTGTAAAAGCTTAAACGTGATTTTATGCTTAA     4136
                ************************************************************

E42             CATGACACAATATTAGGTGAACCTGCAACAGGTTACTCTGTTATGCCCATTTCTTTTGCA     4193
HEINZ           CATGACACAATATTAGGTGAACCTGCAACAGGTTACTCTGTTATGCCCATTTCTTTTGCA     4192
LA2093          CATGACACAATATTAGGTGAACCTGCAACAGGTTACTCTGTTATGCTCATTTCTTTTGCA     4196
                ********************************************** *************

E42             TTCTTACTTCCAATCCTTGAGATCTAACCCTTCTCCATTTTCTGTTGGCATTTATTGGAG     4253
HEINZ           TTCTTACTTCCAATCCTTGAGATCTAACCCTTCTCCATTTTCTGTTGGCATTTATTGGAG     4252
LA2093          TTCTTACTTCCAATCCTTGAGATCTAACCCTTCTCCATTTTCTGTTGGCATTTATTGGAG     4256
                ************************************************************

E42             ATTAGATTTTTGGCAACATCTTTAACAGAGACATGGGTGGAGAGGATGTCAAGGTTTTCT     4313
HEINZ           ATTAGATTTTTGGCAACATCTTTAACAGAGACATGGGTGGAGAGGATGTCAAGGTTTTCT     4312
```

```
LA2093     ATTAGATTTTTGGCAACATCTTTAACAGAGACATGGGTGGAGAGGATGTCAAGGTTTTCT   4316
           ************************************************************

E42        CCTTTTCTGTCTCCTTCATCTGTTGTTGTGGCTATTTTTTTTTATGCACTTCCGCCATTT   4373
HEINZ      CCTTTTCTGTCTCCTTCATCTGTTGTTGTGGCTATTTTTTTTTATGCACTTCCGCCATTT   4372
LA2093     CCTTTTCTGTCTCCTTCATCTGTTGTTGTGGCTATTTT-TTTTATGCACTTCCGCCATTT   4375
           ************************************** *********************

E42        ATCTTTTTAACAGTTTTTGTCCCTTTACTGCAGGTTCCGATTGAACTATCCTTCATGGAA   4433
HEINZ      ATCTTTTTAACAGTTTTTGTCCCTTTACTGCAGGTTCCGATTGAACTATCCTTCATGGAA   4432
LA2093     ATCTTTTTAACAGTTTTTGTCCCTTTACTGCAGGTTCCGATTGAACTATCCTTCATGGAA   4435
           ************************************************************

E42        GCTGTTCAGGGGTGCACTAAGACCATAACATTCCAAACTGATTTGCCTTGCACTGCTTGT   4493
HEINZ      GCTGTTCAGGGGTGCTCTAAGACCATAACATTCCAAACTGATTTGCCTTGCACTGCTTGT   4492
LA2093     GCTGTTCAGGGGTGCTCTAAGACCATAACATTCCAAACTGATTTGCCTTGCACTGCTTGT   4495
           *************** ********************************************

E42        GGTATATATACACTTCCAGCGATTTGCTTTTTGCCTTTTTGCCTCTTTTTTTTTTTTGGG   4553
HEINZ      GGTATATATACACTTCCAGCGATTTGCTTTTTGCCTTTTTGCCTCTTTTTTTTTTTTGGG   4552
LA2093     GGTATATATACACTTCCAGCGATTTGCTTTTTGCCTTTTTGCCTCTTTTTTTTTTTTTTG   4555
           *********************************************************  *

E42        ---TGGGGTGGGGGGTGGGGGGGAGGGGGAGGAATCTAAATAGGGGATCAATATGTCTTA   4610
HEINZ      ---TGGGGTGGGGGGTGGGGGGGAGGGGGAGGAATCTAAATAGGGGATCAATATGTCTTA   4609
LA2093     GGTGGGGTGGGGGGGTGGGGGGGAGGGGGAGGAATCTAAATAGGGGATCAATATGTCTTA   4615
               ***  ***************************************************

E42        AGTACTACTGTGTGATACATGAGCATTAAAGTGCCTACAAGTTGATCAAAGTCTGGTTAT   4670
HEINZ      AGTACTACTGTGTGATACATGAGCATTAAAGTGCCTACAAGTTGATCAAAGTCTGGTTAT   4669
LA2093     AGTACTACTGTGTGATACATGAGCATTAAAGAGCCTACAAGTTGATCAAAGTCTGGTTAT   4675
           ******************************* ****************************

E42        TAACTAGATTAAATAAATAACTAGATTAAATAAGTTTTTTTCCTGATAAG------GACA   4724
HEINZ      TAACTAGATTAAATAAATAACTAGATTAAATAAGTTTTTTTCCTGATAAGGAACAAGACA   4729
LA2093     TAACTAGATTAAATAAATAACTAGATTAAATAAGTTTTTTTCCTGATAAGGAACAAGACA   4735
           **************************************************      ****

E42        TTGAATAAGTTACTGCAGGGTTTTGATGTGAGCTGTGACAGGTGATATCTTGTGAATTTG   4784
HEINZ      TTAAATAAGTTACTGCAGGGTTTTGATGTGAGCTGTGACAGGTGATATCTTGTGAATTTG   4789
LA2093     TTAAATAAGTTACTGCAGGGTTTTGATGTGAGCTGTGACAGGTGATATCTTGTGAATTTG   4795
           ** *********************************************************

E42        CTTAACATAGTTTGTCTAGCCCCTTTCAACCTATAAAATTTATTTCAAGTAGTCCACACC   4844
HEINZ      CTTAACATAGTTTGTCTAGCCCCTTTCAACCTATAAAATTTATTTCAAGTAGTCCACACC   4849
LA2093     CTTAACATAGTTTGTCTAGCCCCTTTCAACCTATAAAATTTATTTCAAGTAGTCCACACC   4855
           ************************************************************

E42        TTCTAGCTCATGTTAGTGAGTTGGACTCCACTCTACTTGGTACTTACTGTAGTTCCTTTT   4904
HEINZ      TTCTAGCTCATGTTAGTGAGTTGGACTCCACTCTACTTGGTACTTACTGTAGTTCCTTTT   4909
LA2093     TTCTAGCTCATGTTAGTGAGTTGGACTCCACTCTACTTGGTACTTACTGTAGTTCCTTTT   4915
           ************************************************************

E42        CTTCTTCCCTTGTTTCTCTTTTAGGTGGAAGTGGTGTGCCCCCTGGCACTAAACCTGAAA   4964
HEINZ      CTTCTTCCCTTGTTTCTCTTTTAGGTGGAAGTGGTGTGCCCCCTGGCACTAAACCTGAAA   4969
LA2093     CTTCTTCCCTTGTTTCTCTTTTAGGTGGAAGTGGTGTGCCCCCTGGCACTAAACCTGAAA   4975
           ************************************************************

E42        CTTGTAGGCGCTGCAAAGGTTCAGGCGTGGTTAGTACCCTTTGCTTATTTCCATTCTTTA   5024
HEINZ      CTTGTAGGCGCTGCAAAGGTTCAGGCGTGGTTAGTACCCTTTGCTTATTTCCATTCTTTA   5029
LA2093     CTTGTAGGCGCTGCAAAGGTTCAGGCGTGGTTAGTACCCTTTGCTTATTTCCATTCTTTA   5035
           ************************************************************

E42        CTTCCAGCAACATGCATGTTTCTTTGTTATAATGTTGGCTCCTTTTATCTTATATTTTCA   5084
HEINZ      CTTCCAGCAACATGCATGTTTCTTTGTTATAATGTTGGCTCCTTTTATCTTATATTTTCA   5089
LA2093     CTTCCAGCAACATGCATGTTTCTTTGTTATAATGTTGGCTCCTTTTATCTTATATTTTCA   5095
           ************************************************************

E42        ATATCCCTTTACAGTCAATCTCGCAAACTGGTCCTTTCACACTTCAGACGACTTGTCCTT   5144
HEINZ      ATATCCCTTTACAGTCAATCTCGCAAACTGGTCCTTTCACACTTCAGACGACTTGTCCTT   5149
LA2093     ATATCCCTTTACAGTCAATCTCGCAAACTGGTCCTTTCACACTTCAGACGACTTGTCCTT   5155
           ************************************************************
```

```
E42       CATGCAAAGGAACAGGGAAAATCGTATCGGTATGACATCCTGTCTACAAAAAACAGAGTT   5204
HEINZ     CATGCAAAGGAACAGGGAAAATCGTATCGGTATGACATCCTGTCTACAAAAAACAGAGTT   5209
LA2093    CATGCAAAGGAACAGGGAAAATCGTATCGGTATGACATCCTGTCTACAAAAAACAGAGTT   5215
          ************************************************************

E42       GTTAGTTATGCATGCAAATAACTGATTGTTCTTTCCTTTTTAAATGGAAAATGATCATT   5264
HEINZ     GTTAGTTATGCATGCAAATAACTGATTGTTCTTTCCTTTTTAAATGGAAAATGATCATT   5269
LA2093    GTTAGTTATGCATGCAAATAACTGATTGTTCTTTCCTTTTTAAATGGAAAATGATCATT   5275
          ************************************************************

E42       GTGCTTGGTTTGCTACATAAATAGGTATAGTTATTATGCTTAACAGATGTCATGACCATC   5324
HEINZ     GTGCTTGGTTTGCTACATAAATAGGTATAGTTATTATGCTTAACAGATGTCATGACCATC   5329
LA2093    GTGCTTGGTTTGCTACATAAATAGGTATAGTTATTATGCTTAACAGATGTCATGACCATC   5335
          ************************************************************

E42       ACAGGAGGAATCTTTAATTTCACTTCATCCCAGTAGGGCAAAAATATAACCATTTTAATT   5384
HEINZ     ACAGGAGGAATCTTTAATTTCACTTCATCCCAGTAGGGCAAAAATATAACCATTTTAATT   5389
LA2093    ACAGGAGGAATCTTTAATTTCACTTCATCCCAGTAGGGCAAAAATATAACCATTTTAATT   5395
          ************************************************************

E42       TTTTAGAAGTTGGAACTTGGATAAGATAAAGTTCCTAAATGTACATCCTATACAAAGAGT   5444
HEINZ     TTTTAGAAGTTGGAACTTGGATAAGATAAAGTTCCTAAATGTACATCCTATACAAAGAGT   5449
LA2093    TTTTAGAAGTTGGAACTTGGATAAGATAAAGTTCCTAAATGTACATCCTATACAAAGAGT   5455
          ************************************************************

E42       GTCTTCAGTCGGGTGATATTTGGAGGAATATTAGTCTTAGTTTTTTTTTTTTTAACAAGG   5504
HEINZ     GTCTTCAGTCGGGTGATATTTGGAGGAATATTAGTCTTAGTTTTTTT-TTTTTAATAAGG   5508
LA2093    GTCTTCAGTCGGGTGATATTTGGAGGAATATTAGTCTTAGTTTTTTT-TTTTTAACAAGA   5514
          *********************************************** ******* ***

E42       AAGAATACTACTCTAGTTTACGGAGGAAGTGTCTGTTCTCAAGTGTTCTATATTCCATAA   5564
HEINZ     AAGAATACTACTCTAGTTTACGGAGGAAGTGTCTCTTCTCAAGTGTTCTATATTCCATAA   5568
LA2093    AAGAATACTACTCTAGTTTACGGAGGAAGTGTCTGTTCTCAAGTGTTCTATATTCCATAA   5574
          ********************************** *************************

E42       GATGATTGAAGTCTTGCTCTTGAAACAATGAACTGTTAAAAAATTTTCTTTTAACTCTTC   5624
HEINZ     GATGATTGAAGTCTTGCTCTTGAAACAATGAACTGTTAAAAAATTTTCTTTTAACTCTTC   5628
LA2093    GATGATTGAAGTCTTGCTCTTGAAACAATGAACTGTTAAAAAATTTTCTTTTAACTCTTC   5634
          ************************************************************

E42       AGCTGACACTTTTAGTCCTATAATTATGTAATGCGTACATCACTTAGAGGAGGAGTGTAT   5684
HEINZ     AGCCGACACTTTTAGTCCTATAATTATGTAATGCGTACATCACTTAGAGGAGGAGTGTAT   5688
LA2093    AGCCGACACTTTTAGTCCTATAATTATGTAATGCGTACATCACTTAGAGGAGGAGTGTAT   5694
          *** ********************************************************

E42       CTTTGGACTCTTAACCACTGCCAGTTGGAGTATTAACTAGCATTTCAATTTTTACTTTGA   5744
HEINZ     CTTTGGACTCTTAACCACTGCCAGTTGGAGTATTAACTAGCATTTCAATTTTTACTTTGA   5748
LA2093    CTTTGGACTCTTAACCACTGCCAGTTGGAGTATTAACTAGCATTTCAATTTTTACTTTGA   5754
          ************************************************************

E42       TGAATTGGCTTTGTTTGATGCATAGTTGTGTTAGAGATTGAAACATTAATAGAATTATTC   5804
HEINZ     TGAATTGGCTTTGTTTGATGCATAGTTGTGTTAGAGATTGAAACATTAATAGAATTATTC   5808
LA2093    TGAATTGGCTTTGTTTGATGCATAGTTGTGTTAGAGATTGAAACATTAATAGAATTATTC   5814
          ************************************************************

E42       TTGGCCTACAGAGTTTCTGCAAGTCTTGCAAAGGAAACCGTGTACTACGAGGACCAAAGA   5864
HEINZ     TTGGCCTACAGAGTTTCTGCAAGTCTTGCAAAGGAAACCGTGTACTACGAGGACCAAAGA   5868
LA2093    TTGGCCTACAGAGTTTCTGCAAGTCTTGCAAAGGAAACCGTGTACTACGAGGACCAAAGA   5874
          ************************************************************

E42       CAGTGAAAGTGGATATCATGCCTGGTATGCTGTAACCACAGATATATCTATGCATGAAGT   5924
HEINZ     CAGTGAAAGTGGATATCATGCCTGGTATGCTGTAACCACAGATATATCTATGCATGAAGT   5928
LA2093    CAGTGAAAGTGGATATCATGCCTGGTATGCTGTAACCACAGATATATCTATGCATGAAGT   5934
          ************************************************************

E42       TATTATGGTGTGAAATATACCTGATTTGTCAACATAACTATCTGATGCATTTTTTGTAAT   5984
HEINZ     AATTATGGTGTGAAATATACCTGATTTGTCAACATAACTATCTGATGCATTTTTTGTAAT   5988
LA2093    AATTATGGTGTGAAATATACCTGATTTGTCAACATAACTATCTGATGCATTTTTTGTAAT   5994
           ***********************************************************

E42       CTGCTGTTTTGAAATCCATTTGTTAGAGTCCGTTCATTATTGATTTAGCAACTTAAGTTG   6044
```

```
HEINZ        CTGCTGTTTTGAAATCCATTTGTTAGAGTCCGTTCATTATTGATTTAGCAACTTAAGTTG    6048
LA2093       CTGCTGTTTTGAAATCCATTTGTTAGAGTCCGTTCATTATTGATTTAGCAACTTAAGTTG    6054
             ************************************************************

E42          ATGTAACCCATGTTATAAAACTATAGCATAGTTGAGACTTGTGCACCACAATTACCCGTT    6104
HEINZ        ATGTAACCCATGTTATAAAACTATAGCATAGTTGAGACTTGTGCACCACAATTGCCCGTT    6108
LA2093       ATGTAACCCATGTTATAAAACTATAGCATAGTTGAGACTTGTGCACCACAATTACCCGTT    6114
             ***************************************************** ******

E42          TTGCCGTTCTCCAAAAATATGCCAAAGAAACCAAAATGGGGAAAATAATGTCAGATGAAA    6164
HEINZ        TTGCCCTTCTCCAAAAATATGCCAAAGAAACCAAAATGGGGAAAATAATGTCAGATGAAA    6168
LA2093       TTGCCCTTCTCCAAAAATATGCCAAAGAAACCAAAATGGGGAAAATAATGTCAGATGAAA    6174
             ***** ******************************************************

E42          GCACCAGCATCTTATTGCTAAAATATGCATGTGGGCTGTTCTGAATTTACTTCCACTCAA    6224
HEINZ        GCACCAGCATCTTATTGCTAAAATATGCATGTGGGCTGTTCTGAATTTACTTCCACTCAA    6228
LA2093       GCACCAGCATCTTATTGCTAAAATATGCATGTGGGCTGTTCTGAATTTACTTCCACTCAA    6234
             ************************************************************

E42          CT----TTTCTTTCACTAATGGATGCCCATACAGTGCTTTTAGCATTAGAGCTTCTATAT    6280
HEINZ        CT----TTTCTTTCACTAATGGATGCCCATACAGTGCTTTTAGCATTAGAGCTTCTATAT    6284
LA2093       ACCTTTTTTCTTTCACTAATGGATGCCCATACAGTGCTTTTAGCATTAGAGCTTCTATAT    6294
                   ******************************************************

E42          AGCACAGGGTAAAACTCTCTGTGTTGAACAACTTTTGCCTCCGCTGTTTTCTCTATTTAT    6340
HEINZ        AGCACAGGGTAAAACTCTCTGTGTTGAACAACTTTTGCCTCCGCTGTTTTCTCTATTTAT    6344
LA2093       AGCACAGGGTAAAACTCTGTGTGTTGAACAACTTTTGCCTCCACTGTTTTCTCTATTTAT    6354
             ****************** ********************** ******************

E42          TATGTGATGTTTAATGGTTCATTGCCTCTTCCGTGAAACCGAAGAAACAGATGAAACCAT    6400
HEINZ        TATGTGATGTTTAATGGTTCATTGCCTCTTCCGTGAAACCGAAGAAACAGATGAAACCAT    6404
LA2093       TATGTGATGTTTAATGGTTCATTGCCTCTTCCGTGAAACCGAAGAAACAGATGACACCAT    6414
             ****************************************************** *****

E42          AAAAACACACATGGACTAGTATCTTGTCCATAAAAACACACATATACAAGTATTCTTGTC    6460
HEINZ        AAAAACACACATGGACTAGTATCTTGTCCATAAAAACACACATATACAATTATTCTTGTC    6464
LA2093       AAAAACACACATGGACTAGTATCTTGTCCATAAAAACACACATATACAAGTATTCTTGTC    6474
             ************************************************* **********

E42          TACATACAGATCAATTCCAAAAGGGGAAACCCATACATGCTGTAGCAAGGGACATAATAT    6520
HEINZ        TACATACAGATCAATTCCAAAAGGGGAAACCGATACATGCTGTAGCAAGGGACATAATAT    6524
LA2093       TACATACAGATCAATTCCAAAAGGGGAAACCCATACATGCTGTAGCAAGGGACATAATAT    6534
             ******************************* ****************************

E42          GTTTTGGCTTGTGCCTGTGCATGCCCCTAGATATTCTGTATGACTCGTTAATTTTCATCA    6580
HEINZ        GTTTTGGCTTGTGCCTGTGCATGCCCCTAGATATTCTGTATGACTCGTTAATTTTCATCA    6584
LA2093       GTTTTGGCTTGTGCCTGTGCATGCCCCTAGATATTCTGTATGACTCGTTAATTTTCATCA    6594
             ************************************************************

E42          ACAGGAGTTGACACTGACGAGACACTCAAGGTGTATGGAAGTGGTGGAGCAGATCCTGAA    6640
HEINZ        ACAGGAGTTGACACTGACGAGACACTCAAGGTGTATGGAAGTGGTGGAGCAGATCCTGAA    6644
LA2093       ACAGGAGTTGACACTGACGAGACACTCAAGGTGTATGGAAGTGGTGGAGCCGATCCTGAA    6654
             ************************************************** *********

E42          GGCAATCGACCTGGAGATCTTTACGTTGTTCTTAAGGTTCGGTTGAAACATGGTTCTTTT    6700
HEINZ        GGCAATCGACCTGGAGATCTTTACGTTGTTCTTAAGGTTCGGTTGAAACATGGTTCTTTT    6704
LA2093       GGCAATCGACCTGGAGATCTTTACGTTGTTCTTAAGGTTCGGTTGAAACATGGTTCTTTT    6714
             ************************************************************

E42          GTATGTCAGGTTTTATTGTTCTCAAATTTGATTCATGTTATTCATAGAGGCTTCTGGTAT    6760
HEINZ        GTATGTCAGGTTTTATTGTTCTCAAATTTGATTCATGTTATTCATAGAGGCTTCTGGTAT    6764
LA2093       GTATGTCAGGTTTTATTGTTCTCAAATTTGATTCATGTTATTCATAGAGGCTTCTGGTAT    6774
             ************************************************************

E42          TCCTTTTTTAGGTCAGAGAAGACCCTGTTTTCCGGAGAGAAGGCTCTGATATTCATGTAG    6820
HEINZ        TCCTTTTTTAGGTCAGAGAAGACCCTGTTTTCCGGAGAGAAGGCTCTGATATTCATGTAG    6824
LA2093       TCCTTTTTTAGGTCAGAGAAGACCCTGTTTTCCGGAGAGAAGGCTCTGATATTCATGTAG    6834
             ************************************************************

E42          ATGCTGTTTTGAGTATCACCCAGGTACTATTTCCATAACTAAATATAAGCTTTGTTGATC    6880
HEINZ        ATGCTGTTTTGAGTATCACCCAGGTACTATTTCCATAACCAAATATAAGCTTTGTTGATC    6884
LA2093       ATGCTGTTTTGAGTATCACCCAGGTACTATTTCCATAACCAAATATAAGCTTTGTTGATC    6894
```

```
               *************************************** ********************

E42            TAAACAAATTTGTTGCTGCAGTATATTAAGGAATATAGAGTAACTACAGTCAACTATTCC     6940
HEINZ          TAAACAAATTTGTTGCTGCAGTATATTAAGGAATATAGAGTAACTACAGTCAACTATTCC     6944
LA2093         TAAACAAATTTGTTGCTGCAGTATATTAAGGAATATAGAGTAACTACAGTCAACTATTCC     6954
               ************************************************************

E42            CTCGTAATTCTTGAGTAGATTTTTGCTTACTGTTTTCAGTGTTATTTTTGGCATCTAAAT     7000
HEINZ          CTCGTAATTCTTGAGTAGATTTTTGCTTACTGTTTTCAGTGTTATTTTTGGCATCTAAAT     7004
LA2093         CTCGTAATTCTTGAGTAGATTTTTGCTTACTGTTTTCAGTGTTATTTTTGGCATCTAAAT     7014
               ************************************************************

E42            TGTCATCCTCATTTCACTGTTTCCTGGCGTTTTTTTGTCTTCAAAATCTTCTTCTTGTGA     7060
HEINZ          TGTCATCCTCATTTCACTGTTTCCTGGCGTTTTTTTGTCTTCAAAATCTTCTTCTTGTGA     7064
LA2093         TGTCATCCTCATTTCACTGTTTCCTGGCGTTTTTTTGTCTTCAAAATCTTCTTCTTGTGA     7074
               ************************************************************

E42            TGCTAATAATCTTTTAAACCTTTCAATTCCATTTACCTAAGTTCTTGACGTAGATTACCA     7120
HEINZ          TGCTAATAATCTTTTAAACCTTTCAATTCCATTTACCTAAGTTCTTGACGTAGATTACCA     7124
LA2093         TGCTAATAATCTTTTAAACCTTTCAATTCCATTTACCTAAGTTCTTGACGTAGATTACCA     7134
               ************************************************************

E42            CTGATTAAAAACTATATGTACTTAGTGGATCGAGTATACCAGATGAAACATTGTGCATTA     7180
HEINZ          CTGATTAAAAACTATATGTACTTAGTGGATCGAGTATACCAGATGAAACATTGTGCATTA     7184
LA2093         CTGATTAAAAACTATATGTACTTAGTGGATCGAGTATACCAGATGAAACATTGTGCATTA     7194
               ************************************************************

E42            ATATTCTACACATGAGCTAATTGACAAATTTTATGACATTAACACCTGCTGGGAACTCAG     7240
HEINZ          ATATTCTACACATGAGCTAATTGACAAATTTTATGACATTAACACCTGCTGGGAACTCAG     7244
LA2093         ATATTCTACACATGAGCTAATTGACAAATTTTATGACATTAACACCTGCTGGGAACTCAG     7254
               ************************************************************

E42            GGACCAAAAAGTAACAATGCATCATTTATGGACATCTGATAGCCATCACAGTTGTCAACC     7300
HEINZ          GGACCAAAAAGTAACAATGCATCATTTATGGACATCTGATAGCCATCACAGTTGTCAACC     7304
LA2093         GGACCAAAAAGTAACAATGCATCATTTATGGACATCTGATAGCCATCACAGTTGTCAACC     7314
               ************************************************************

E42            TGAGGCCACATGCTTCTGCCATTTTGTTGTTTCTTTTTGTGGTTCCTAAAGCTATGAATT     7360
HEINZ          TGAGGCCACATGCTTCTGCCATTTTGTTGTTTCTTTTTGTGGTTCCTAAAGCTATGAATT     7364
LA2093         TGAGGCCACATGCTTCTGCCATTTTGTTGTTTCTTTTTGTGGTTCCTAAAGCTATGAATT     7374
               ************************************************************

E42            ATGAATCTTCTCTATTTATGATGGTTAGATTCTAATGAATACTTAACTGCAGGCAATCTT     7420
HEINZ          ATGAATCTTCTCTATTTATGATGGTTAGATTCTAATGAATACTTAACTGCAGGCAATCTT     7424
LA2093         ATGAATCTTCTCTATTTATGATGGTTAGATTCTAATGAATACTTAACTGCAGGCAATCTT     7434
               ************************************************************

E42            GGGAGGAACAATCCAAGTCCCAACTCTGACAGGAGATGTTGTTGTTAAGGTACTAAAATA     7480
HEINZ          GGGAGGAACAATCCAAGTCCCAACTCTGACAGGAGATGTTGTTGTTAAGGTACTAAAATA     7484
LA2093         GGGAGGAACAATCCAAGTCCCAACTCTGACAGGAGATGTTGTTGTTAAGGTACTAAAATA     7494
               ************************************************************

E42            CTATTAAGCTTATCATGGTTAACCTTTATCCTTCTATTTTCCTTCTTAGAATGTTTAAAA     7540
HEINZ          CTATTAAGCTTATCATGGTTAACCTTTATCC-TCTATTTTCCTTCTTAGAATGTTTAAAA     7543
LA2093         CTATTAAGCTTATCATGGTTAACCTTTATCC-TCTATTTTCCTTCTTAGAATGTTTAAAA     7553
               ******************************* ****************************

E42            GTTTGATTCTTATGCACTTTGATTGTCCCTACAACTTGGTAATGTGAGATGTTCCTCCTA     7600
HEINZ          GTTTGATTCTTATGCACTTTGATTGTCCCTACAACTTGGTAAAGTGAGATGTTCCTCCTA     7603
LA2093         GTTTGATTCTTATGCACTTTGATTGTCCCTACAACTTGGTAAAGTGAGATGTTCCTCCTA     7613
               ****************************************** *****************

E42            GCATATCTCAGCATGTCTAATACTCATTGTGAAGGCAATGTTCAAGTAACACTATAAGAG     7660
HEINZ          GCATATCTCAGCATGTCTAATACTCATTGTGAAGGCAATGTTCAAGTAACACTATAAGAG     7663
LA2093         GCATATCTCAGCATGTCTAATACTCATTGTAAAGGCAATGTTCAAGTAACACTATAAGAG     7673
               ****************************** *****************************

E42            TAGAAATATATTGGGTGATACTGGTTTTATGATGGTTGGGCTTAGAGTTGAAAATATAGC     7720
HEINZ          TAGAAATATATTGGGTGATACTGGTTTTATGATGGTTGGGCTTAGAGTTGAAAA--TAGC     7721
LA2093         TAGAAATATATTGGGTGATACTGGTTTTATGATGGTTGGGCTTAGAGTTGAAAA--TAGC     7731
               ******************************************************  ****
```

```
E42         AGGAAGAGATGAACCACAGGGAGCGCTCAGGGACTACTGGGAGGCAATGTTGAAAGTCGG    7780
HEINZ       AGGAAGAGATGAACCACAGGGAGCGCTCAGGGACTACTGGGAGGCAATGTTGAAAGTCGG    7781
LA2093      AGGAAGAGATGAACCACAGGGAGCGCTCAGGGACTACTGGGAGGCAATGTTGAAAGTCGG    7791
            ************************************************************

E42         CAGGGAGCTTGGATTTTTTTCCGCATTGCTCAGAATTTCATGAATTGAAAAGTGAGAGTA    7840
HEINZ       CAGGGAGCTTGGATTTTTTTCCGCATTGCTCAGAATTTCATGAATTGAAAAGTGAGAGTA    7841
LA2093      CAGGGAGCTTGGATTTTTTTCCGCATTGCTCAGAATTTCATGAATTGAAAAGTGAGAGTA    7851
            ************************************************************

E42         GCATGATGTTATCATGAAATATAAACACTAATGCCTGTCATACAAACTTATTGTACTACT    7900
HEINZ       GCATGATGTTATCATGAAATATAAACACTAATGCCTGTCATACAAACTTATTGTACTACT    7901
LA2093      GCATGATGTTATCATGAAATATAAACACTAATGCCTGTCATACAAACTTATTGTACTACT    7911
            ************************************************************

E42         TCTTTTTGCCAAAGTTCTAGTGAGGGTCCACCTAAACTATTCAAAGAAAATCAAATTAAG    7960
HEINZ       TCTTTTTGCCAAAGTTCTAGTGAGGGTCCACCTAAACTATTCAAAGAAAATCAAATTAAG    7961
LA2093      TCTTTTTGCCAAAGTTCTAGTGAGGGTCCACCTAAACTATTCAAAGAAAATCAAATTAAG    7971
            ************************************************************

E42         TGGAAAATCTCTTGACAAATGAGCTTATTTGACCTCTATAACAGCAACTGCCAAAGTACC    8020
HEINZ       TGGAAAATCTCCTGACAAATGAGCTTATTTGACCTCTATAACAGCAACTGCCAAAGTACC    8021
LA2093      TGGAAAATCTCCTGACAAATGAGCTTATTTGACCTCTATAACAGCAACTGCCAAAGTACC    8031
            *********** ************************************************

E42         ACTTCGTCAGAAAGTTCCTGGAGTTGGAATTTAAGGCGAAGAAGTAGATCAAATATTCTG    8080
HEINZ       ACTTCGTCAGAAAGTTCCTGGAGTTGGAATTTAAGGCGAAGAAGTAGATCAAATATTCTG    8081
LA2093      ACTTCGTCAGAAAGTTCCTGGAGTTGGAATTTAAGGCGAAGAAGTAGATCAAATATTCTG    8091
            ************************************************************

E42         CACTGATACTAGGTGGTAAATTAGGCAAGCTCTTGTTTGATCTGTTTCCAGATATTTATT    8140
HEINZ       CACTGATACTAGGTGGTAAATTAGGCAAGCTCTTGTTTGATCTGTTTCCAGATATTTATT    8141
LA2093      CACTGATACTAGGTGGTAAATTAGGCAAGCTCTTGTTTGATCTGTTTCCAGATATTTATT    8151
            ************************************************************

E42         AATTCCTTTTTTATTTGGGTTAAGAATCTTCTACTACTTGACCAGAAATTTCCCAACATG    8200
HEINZ       AATTCCTTTTTTATTTGGGTTAAGAATCTTCTACTACTTGACCAGAAATTTCCCAACATG    8201
LA2093      AATTCCTTTTTTATTTGGGTTAAGAATCTTCTACTACTTGACCAGAAATTTCCCAACATG    8211
            ************************************************************

E42         AAAAGTTTAGAGCATAAAGTAGATTCTATTTTCTGCAGTGATTAGGTTAACTAAGCAATT    8260
HEINZ       AAAAGTTTAGGGCATAAAGTAGATTCTATTTTCTGCAGTGATTAGGTTAACTAAGCAATT    8261
LA2093      AAAAGTTTAGGGCATAAAGTAGATTCTATTTTCTGCAGTGATTAGGTTAACTAAGCAATT    8271
            ********** *************************************************

E42         ATCGTACTTGTTCAATCTACATTGAGATAGTACCTTGTCAGTTTTTGAGTCTTCAAATCA    8320
HEINZ       ATCGTACTTGTTCAATCTACATTGAGATAGTACCTTGTCAGTTTTTGAGTCTTCAAATCA    8321
LA2093      ATCGTACTTGTTCAATCTACATTGAGATAGTACCTTGTCAGTTTTTGAGTCTTCAAATCA    8331
            ************************************************************

E42         TTCGTACCCTGCATTATATGAGTTCTGAGAAGTAGTTACTGGTCTGTTTCTTGTGGGCAA    8380
HEINZ       TTCGTACCCTGCATTATATGAGTTCTGAGAAGTAGTTACTGGTCTGTTTCTTGTGGGCAA    8381
LA2093      TTCGTACCCTGCATTATTTGAGTTCTGAGAAGTAGTTACTGGTCTGTTTCTTGTGGGCAA    8391
            ***************** ******************************************

E42         GGGTCATTGCAAGAATGTTAAGGCTATTTATACAAGCCAATTTGCTTTATTGTCTGTTCA    8440
HEINZ       GGGTCATTGCAAGAATGTTAAGGCTATTTATACAAGCCAATTTGCTTTATTGTCTGTTCA    8441
LA2093      GGGTCATTGCAAGAATGTTAAGGCTATTTATACAAGCCAATTTGCTTTATTGTCTGTTCA    8451
            ************************************************************

E42         AAATAAAGGAGGAATTAATTTTATTATTTGTCCTTTGATAGCTTGATCTCCGTGCCCAAG    8500
HEINZ       AAATAAAGGAGGAATTAATTTTATTATTTGTCCTTTGATAGCTTGATCTCCGTGCCCAAG    8501
LA2093      AAATAAAGGAGGAATTAATTTTATTATTTGTCCTTTGATAGCTTGATCTCCGTGCCCAAG    8511
            ************************************************************

E42         TTTTGACATCCTGTTGTTTGTAGGTTCGTTCTGGCACTCAACCTGGCCAAAAGGTTGTCC    8560
HEINZ       TTTTGACATCCTGTTGTTTGTAGGTTCGTTCTGGCACTCAACCTGGCCAAAAGGTTGTCC    8561
LA2093      TTTTGACATCCTGTTGTTTGTAGGTTCGTTCTGGCACTCAACCTGGCCAAAAGGTTGTCC    8571
            ************************************************************

E42         TTAAAAAGAAAGGTAAGAACTTCATTTACTTTGTTTGATGCAGATTTTTTCTTCTGGTTT    8620
HEINZ       TTAAAAAGAAAGGTAAGAACTTCATTTACTTTGTTTGATGCAGATTTTTTCTTCTGGTTT    8621
```

```
LA2093          TTAAAAAGAAAGGTAAGAACTTCATTTACTTTGTTTGATGCAGATTTTTTCTTCTGGTTT     8631
                ************************************************************

E42             ACTTATTTTGATGAATGCTCAAGTTATACTTGAATGTCAGTTGATTTGGGTATTTTCCAT     8680
HEINZ           ACTTATTTTGATGAATGCTCAAGTTATACTTGAATGTCAGTTGATTTGGGTATTTTCCAT     8681
LA2093          ACTTATTTTGATGAATGCTCAAGTTATACTTGAATGTCAGTTGATTTGGGTATTTTCCAT     8691
                ************************************************************

E42             ATCAAGTCACAGTGTGAGAAGT---TGGAGTTTTATCAATTTACAATCTGATGTGTGTAA     8737
HEINZ           ATCAAGTCACAGTGTGAGACGTAGTTGGAGTTTTATCAATTTACAATCTGATGTGTGTAA     8741
LA2093          ATCAAGTCACAGTGTGAGACGT---TGGAGTTTTATCAATTTACAATCTGATGTGTGTAA     8748
                ******************* **   ***********************************

E42             TAACGAATAACTACATTGTTCGCCAGGCCTCCCAGCTTCTCAAAGCATTTTTCTTTAATG     8797
HEINZ           TAACGAATAACTACATTGTTCGCCAGGCCTCCCAGCTTCTCAAAGCATTTTTCTTTAATG     8801
LA2093          TAACGAATAACTACATTGTTCGCCAGGCCTCCCAGCTTCTCAAAGCATTTTTCTTTAATG     8808
                ************************************************************

E42             TGCACAGAGTCGGTTAACCTTGTTATAAACTTTCTTGTTCAAGCTTATAACATTTCTGTG     8857
HEINZ           TGCACAGAGTCGGTTAACCTTGTTATAAACTTTCTTGTTCAAGCTTATAACATTTCTGTG     8861
LA2093          TGCACAGAGTCGGTTAACCTTGTTATAAACTTTCTTGTTCAAGCTTATAACATTTCTGTG     8868
                ************************************************************

E42             TGCTCATGCAGGAATAAAAGGACGGAATTCCTACTCATTTGGGGATCAGTTTGTTCACTT     8917
HEINZ           TGCTCATGCAGGAATAAAAGGACGGAATTCCTACTCATTTGGGGATCAGTTTGTTCACTT     8921
LA2093          TGCTCATGCAGGAATAAAAGGACGGAATTCCTACTCATTTGGGGATCAGTTTGTTCACTT     8928
                ************************************************************

E42             CAATGTCAGCATTCCCACGTAGGTGCCCTTTTGTGTAAAATATGGAAATGACTGCCAACT     8977
HEINZ           CAATGTCAGCATTCCCACGTAGGTGCCCTTTTGTGTAAAATATGGAAATGACTGCCAACT     8981
LA2093          CAATGTCAGCATTCCCACGTAGGTGCCCTTTTGTGTAAAATATGGAAATGACTGCCAACT     8988
                ************************************************************

E42             ACTGCACCTTTTTTACTACTCTAGTCTCTTTCTTAAGCCTGTTATTTAGAATTGAGGAGA     9037
HEINZ           ACTGCACCTTTTTTACTACTCTAGTCTCTTTCTTAAGCCTGTTATTTAGAATTGAGGAGA     9041
LA2093          ACTGCACCTTTTTTACTACTCTAGTCTCTTTCTTAAGCCTGTTATTTAGAATTGAGGAGA     9048
                ************************************************************

E42             TTTATAATGGCCTCAAAATACTCTTGAAACATCTTCTACTAGAGAAGGTACACACACCCC     9097
HEINZ           TTTATAATGGCCTCAAAATACTCTTGAAACATCTTCTACTAGAGAAGGTACACACACCCC     9101
LA2093          TTTATAATGGCCTCAAAATACTCTTGAAACATCTTCTACTAGAGAAGGTACACACACCCC     9108
                ************************************************************

E42             TGAAGCTATCCTGTATTACACTATCTAATGATGAAATTTGCCAACTTGGGTGCAGGAATT     9157
HEINZ           TGAAGCTATCCTGTATTACACTATCTAATGATGAAATTTGCCAACTTGGGTGCAGGAATT     9161
LA2093          TGAAGCTATCCTGTATTACACTATCTAATGATGAAATTTGCCAACTTGGGTGCAGGAATT     9168
                ************************************************************

E42             TGACACCAAGACAGCGTCAATTGATTGAAGAGTTTGCCAAGGAGGAGCAAGGGGAGTATG     9217
HEINZ           TGACACCAAGACAGCGTCAATTGATTGAAGAGTTTGCCAAGGAGGAGCAAGGGGAGTATG     9221
LA2093          TGACACCAAGACAGCGTCAATTGATTGAAGAGTTTGCCAAGGAGGAGCAAGGGGAGTATG     9228
                ************************************************************

E42             ATAAAGGGGCCGCAGCAGGAGCCTCGAGGTGAGGGCATGGCACAGTGCTTACAAGGTAGG     9277
HEINZ           ATAAAGGGGCCGCAGCAGGAGCCTCGAGGTGAGGGCATGGCACAGTGCTTACAAGGTAGG     9281
LA2093          ATAAAGGGGCCGCAGCAGGAGCCTCGAGGTGAGGGCATGGCACAGTGCTTACAAGGTAGG     9288
                ************************************************************

E42             TTAGGGGATATAGGTTTGAGTCAAAAGTGTGTACTATGACGTATTCTTTCTCTCATGAGC     9337
HEINZ           TTAGGGGATATAGGTTTGAGTCAAAAGTGTGTACTATGACGTATTCTTTCTCTCATGAGC     9341
LA2093          TTAGGGGATATAGGTTTGAGTCAAAAGTGTGTACTATGACGTATTCTTTCTCTCATGAGC     9348
                ************************************************************

E42             TGGGGAACTTAGCCAGAGCAATTTTGGGGTTTGGTCATTACCATTTTATCAGTTTCCTTT     9397
HEINZ           TGGGGAACTTAGCCAGAGCAATTTTGGGGTTTGGTCATTACCATTTTATCAGTTTCCTTT     9401
LA2093          TGGGGAACTTAGCCAGAGCAATTTTGGGGTTTGGTCATTACCATTTTATCAGTTTCCTTT     9408
                ************************************************************

E42             ACATTCGAGCGAGGATTTAATTGTTGCTTGATGGAGGAGGGGACAGGTTGGTTATAGTAT     9457
HEINZ           ACATTCGAGCGAGGATTTAATTGTTGCTTGATGGAGGAGGGGACAGGTTGGTTATAGTAT     9461
LA2093          ACATTCGAGCGAGGATTTAATTGTTGCTTGATGGAGGAGGGGACAGGTTGGTTATAGTAT     9468
                ************************************************************
```

```
E42         TATTATTGTGACATTGAGATAATGTAGCACTTGATCTGCAACTTAGTGTTGTATATATAAAT    9517
HEINZ       TATTATTGTGACATTGAGATAATGTAGCACTTGATCTGCAACTTAGTGTTGTATATATAAAT    9521
LA2093      TATTATTGTGACATTGAGATAATGTAGCACTTGATCTGCAACTTAGTGTTGTATATATAAAT    9528
            ************************************************************

E42         ATGATGAAACAAATAAATGTTTCTCAAGGGAAGTGGAATGTTACCTGCCATTCATCCTT     9576
HEINZ       ATGATGAAACAAATAAATGTTTCTCAAGGGAAGTGGAATGTTACCTGCCATTCATCCTT     9580
LA2093      ATGATGAAACAAATAAATGTTTCTCAAGGGAAGTGGAATGTTACCTGCCATTCATCCTT     9587
            ***********************************************************
```

Solyc01g088730

```
E42             ATGAATAGCGTGCAGAAAGGATTCCTCTATTCTAATTGAAAAGATATATGTGCTTCAGTG     60
HEINZ           ATGAATAGCGTGCAGAAAGGATTCCTCTATTCTAATTGAAAAGATATATGTGCTTCAGTG     60
LA2093          ATGAATAGCGTGCAGAAAGGATTCCTCTATTCTAATTGAAAAGATATATGTGCTTCAGTG     60
                ************************************************************

E42             ACCTCAATACAAGACTTTGAGGCTACAAAAGGACTTAACGCAGTAAACCTCAGAGAGTCG     120
HEINZ           ACCTCAATACAAGACTTTGAGGCTACAAAAGGACTTAACGCAGTAAACCTCAGAGAGTCA     120
LA2093          ACCTCAATACAAGACTTTGAGGCTACAAAAGGACTTAACGCAGTAAACCTCAGAGAGTCA     120
                ***********************************************************

E42             TATCGCTAGCGTAGTGATGAATCACATAACACCGATAAACACATAACAGAATCCAAAAGA     180
HEINZ           TATCGCTAGCGTAGTGATGAATCACATAACACCGATAAACACATAACAGAATCCAAAAGA     180
LA2093          TATCGCTAGCGTAGTGATGAATCACATAACACCGATAAACACATAACAGAATCCAAAAGA     180
                ************************************************************

E42             ATAATATAAAATACAACGATGACTTAAACACCCCACTTTAGTATGTATCCATCCGTCTTG     240
HEINZ           ATAATATAAAATACAACGATGACTTAAACACCCCACTTTAGTATGTATCCATC-GTCTTG     239
LA2093          ATAATATAAAATACAACGATGACTTAAACACCCCACTTTAGTATGTATCCATC-GTCTTG     239
                ***************************************************** ******

E42             GCATATTGCAACTTTTCTGAAAAATATTCATGTATCCATGGCACACCAAGTGATTCGTAT     300
HEINZ           GCATATTGCAACTTTTCTGAAAAATATTCATGTATCCATGGCACACTAAGTGATTCGTAT     299
LA2093          GCATATTGCAACTTTTCTGAAAAATATTCATGTATCCATGGCACACTAAGTGATTCGTAT     299
                ********************************************** *************

E42             TGCAAACACCGTGTACAGAATACCAGCATTGCATAATTTAGATGAATCTCTCCTGAGCCC     360
HEINZ           TGCAAACACCGTGTACAGAATACCAGCATTGCATAATTTAGATGAATCTCTCCTGAGCCC     359
LA2093          TGCAAACACCGTGTACAGAATACCAGCATTGCATAATTTAGATGAATCTCTCCTGAGCCC     359
                ************************************************************

E42             TATCTTGTGCCAGAAATACGCGTCAGACAACAAACCTGCTACAGAGATACCAGCAAAAAG     420
HEINZ           TATCTTGTGCCAGAAATACGCGTCAGACAACAAACCTGCTACAGAGATACCAGCAAAAAG     419
LA2093          TATCTTGTGCCAGAAATACGCGTCAGACAACAAACCTGCTACAGAGATACCAGCAAAAAG     419
                ************************************************************

E42             AAATTTAAACTTTCTTGAAATCAATCTTACATGATTACAAGGAACTATCCTTCAGGACTG     480
HEINZ           AAATTTAAACTTTCTTGAAATCAATCTTACATGATTACAAGGAACTATTCTTCAGAACTG     479
LA2093          AAATTTAAACTTTCTTGAAATCAATCTTACATGATTACAAGGAACTATTCTTCAGAACTG     479
                ************************************************ ****** ****

E42             GAATGACACCTCCATACTGTGTAAAATATGCAAGAAATATATGAAACAAGATAAAAGAAT     540
HEINZ           GAATGACAGCCCCATACTGTGTAAAATATGCAAGAAATATATGAAACAAGATAAAAGAAT     539
LA2093          GAATGACAGCCCCATACTGTGTAAAATATGCAAGAAATATATGAAACAAGATAAAAGAAT     539
                ******** * *************************************************

E42             TACTATTAAATTCTGTCTTTCTTCTTTATCTGTGACAATCTAGACATGCCATCATCTCAG     600
HEINZ           TACTATTAAATTCTGTCTTTCTTCTTTATCTGTGACAATCTAGACATGCCATCATCTCAG     599
LA2093          TACTATTAAATTCTGTCTTTCTTCTTTATCTGTGACAATCTAGACATGCCATCATCTCAG     599
                ************************************************************

E42             CACTTGAACAGAATGGAGCTTCTGCATGCTATTTCTTCCTTTGAGCAGCCTGTGATAAGT     660
HEINZ           CACTTGAACAGAATGGAGCTTCTGCATGCTATTTCTTCCTTTGAGCAGCCTGTGATAAGT     659
LA2093          CACTTGAACAGAATGGAGCTTCTGCATGCTATTTCTTCCTTTGAGCAGCCTGTGATAAGT     659
                ************************************************************

E42             GAAACCATACATGTAAGGAAGTTCCTTAAAAAAATTTATTTCAAGGATGGACAAACGACGT     720
HEINZ           GAAACCGTACATGTAAGGAAGTTCCTTAAAAAAATTTATTTCAAGGATGGACAAACGACGT     719
LA2093          GAAACCGTACATGTAAGGAAGTTCCTTAAAAAAATTTATTTCAAGGATGGACAAACGACGT     719
                ****** *****************************************************

E42             ACTGAAAAAATTTCTTTCTAAAATATAAAGGTAAGGAGAGATGATTAAAATGGAGCAGTTT     780
HEINZ           ACTGAAAAAATTTCTTTCTAAAATATAAAGGTAAGGAGAGATGATTAAAATGGAGCAGTTT     779
LA2093          ACTGAAAAAATTTCTTTCTAAAATATAAAGGTAAGGAGAGATGATTAAAATGGAGCAGTTT     779
                ************************************************************

E42             CTAACAATAACAGTAACAATTCCACAAAGGAGAAGTGAAATTAGAAACTATTTTAACTCC     840
HEINZ           CTAACAATAACAGTAACAATTCCACAAAGGAGAAGTGAAATTAGAAACTATTTTAACTCC     839
LA2093          CTAACAATAACAGTAACAATTCCACAAAGGAGAAGTGAAATTAGAAACTATTTTAACTCC     839
                ************************************************************
```

```
E42         TCAAATCAACTCAATCTGTCCTCCTTTCTTTTCCCTTCCATTTTTCAGTACAAGCATGGA    900
HEINZ       TCAAATCAACTCAATCTGTCCTCCTTTCTTTTCCCTTCCATTTTTCAGTACAAGCATGGA    899
LA2093      TCAAATCAACTCAATCTGTCCTCCTTTCTTTTCCCTTCCATTTTTCAGTACAAGCATGGA    899
            ************************************************************

E42         CACTTTTTATTGAGAAGAAGCATGGATATTTTCTTTTAGAGCACCAGCGAAGTTGTATCA    960
HEINZ       CACTTTTTATTGAGAAGAAGCATGGATATTTTCTTTTAGAGCACCAGCGAAGTTGTATCA    959
LA2093      CACTTTTTATTGAGAAGAAGCATGGATATTTTCTTTTAGAGCACCAGCGAAGTTGTATCA    959
            ************************************************************

E42         GACCTCAAAATTAGGTAATATATGTAAAAGATGAAACCCAATTGACGGCTTCTCACTGAT    1020
HEINZ       GACCTCAAAATTAGGTAATATATGTAAAAGATGAAACCCAATTGACGGCTTCTCACTGAT    1019
LA2093      GACCTCAAAATTAGGTAATATATGTAAAAGATGAAACCCAATTGACGGCTTCTCACTGAT    1019
            ************************************************************

E42         TTGGTTGTGTTCCACTCCATTCATGCACCTCTCGACCCTCCGGACATAGCAAAGCACATT    1080
HEINZ       TTGATTGTGTTCCACTCCATTCATGCACCTCTCGACCCTCCGGACATAGCAAAGCACATT    1079
LA2093      TTGATTGTGTTCCACTCCATTCATGCACCTCTCGACCCTCCGGACATAGCAAAGCACATT    1079
            *** ********************************************************

E42         CTTTTTTCTTTCTTTTTTTTAAAGAAATCAGTTCATTTCTCCAATAACATCATCTTGTGT    1140
HEINZ       CTTTTTTCTTTCTTTTTTTTAAAGAAATCAGTTCATTTCTCCAATAACATCATCTTGTGT    1139
LA2093      CTTTTTTCTTTCTTTTTTTTAAAGAAATCAGTTCATTTCTCCAATAACATCATCTTGTGT    1139
            ************************************************************

E42         GGTTGAATGTTGTCAAAGGAGCAGTAAATGGACAGGTTATACCACCTAATTTGATTAGAA    1200
HEINZ       GGTTGAATGTTGTCAAAAGAGCAGTAAATGGACAGGTTATACCACCTAATTTGATTAGAA    1199
LA2093      GGTTGAATGTTGTCAAAAGAGCAGTAAATGGACAGGTTATACCACCTAATTTGATTAGAA    1199
            ***************** ******************************************

E42         CCTCACGTGTTGACAAAATAGAAAAGTTAATTGAAGTCATCCATCAAGCTTTCCTGCAGT    1260
HEINZ       CCTCACGTGTAGACAAAATAGAAAAGTTAATTGAAGTCATCCATCAAGCTTTCCTGCAGT    1259
LA2093      CCTCACGTGTAGACAAAATAGAAAAGTTAATTGAAGTCATCCATCAAGCTTTCCTGCAGT    1259
            ********** *************************************************

E42         CTAGTAGTCTCAGTAGCAAATAGTAGCAGAATCAGATTTTACTGAAGTCTATATAGCAAC    1320
HEINZ       CTAGTAGTCTCAGTAGCAAATAGTAGCAGAATCAGATTTTACTGAAGTCTATATAGCAAC    1319
LA2093      CTAGTAGTCTCAGTAGCAAATAGTAGCAGAATCAGATTTTACTGAAGTCTATATAGCAAC    1319
            ************************************************************

E42         TTAGCAGTAAAAACAACCTTACTGAAATACTACTTCCTTCTATCACTTAGCAAAGTCAAG    1380
HEINZ       TTAGCAGTAAAAACAACCTTACTGAAATACTACTTCCTTCTATCACTTAGCAAAGTCAAG    1379
LA2093      TTAGCAGTAAAAACAACCTTACTGAAATACTACTTCCTTCTATCACTTAGCAAAGTCAAG    1379
            ************************************************************

E42         CCCTATCAGATTTCTTCAGCAGAAAACATTCATAAATACCACTTTTTTTTAAATAGAATT    1440
HEINZ       CCCTATCAGATTTCTTCAGCAGAAAACATTCACAAATACCACTTTTTTTTAAATAGAATT    1439
LA2093      CCCTATCAGATTTCTTCAGCAGAAAACATTCATAAATACCACTTTTTTTTAAATAGAATT    1439
            ******************************** ***************************

E42         TAAGATCCCTACCCAGAAAACTTCACTAATATATTTAGTCACAAACAAAGAAACAACTTC    1500
HEINZ       TAAGATCCCTACCCAGAAAACTTCACTAATATATTTAGTCACAAACAAAGAAACAACTTC    1499
LA2093      TAAGATCCCTACCCAGAAAACTTCACTAATATATTTAGTCACAAACAAAGAAACAACTTC    1499
            ************************************************************

E42         TGCTGGAAGCGCACAGGCATAACTACAAAAACTCTTTACAATTTTTTTAAAAGCCTTTTT    1560
HEINZ       TGCTGGAAGCGCACAGGCATAACTTCAAAAACTCTTTACAATTTTTTTAAAAGCCTTTTT    1559
LA2093      TGCTGGAAGCGCACAGGCATAACTTCAAAAACTCTTTACAATTTTTTTAAAAGCCTTTTT    1559
            ************************ ***********************************

E42         ACATAAAAGGTTCCCCTCATAGTTACAGATGCAATTACTACTCTTAATAAGATTAACTTT    1620
HEINZ       ACATAAAAGGTTCCCCTCATAGTTACAGATGCAATTACTACTCTTAATAAGATTAACTTT    1619
LA2093      ACATAAAAGGTTCCCCTCATAGTTACAGATGCAATTACTACTCTTAATAAGATTAACTTT    1619
            ************************************************************

E42         GAGTTCTTCTCAACCATAGATACATATTCAGAAGGATTCAACTTGAGGTTAAAAAAAAGA    1680
HEINZ       GAGTTCTTCTCAACCATAGATACATATTCAGAAGGATTCCACTTGAGGTTAAAAAAAAGA    1679
LA2093      GAGTTCTTCTCAACCATAGATACATATTCAGAAGGATTCCACTTGAGGTTAAAAAAAAGA    1679
            *************************************** ********************

E42         CAACAAATTCAGATAGAATAGACCTATTTGCGAATTCATTACAATCTACAGCACTGTATA    1740
```

```
HEINZ          CAACAAATTCAGATAGAATAGACCTATTTGCGAATTCATTACAATCTACAGCACTGTATA     1739
LA2093         CAACAAATTCAGATAGAATAGACCTATTTGCGAATTCATTACAATCTACAGCACTGTATA     1739
               ************************************************************

E42            AAAGGATATATCAGAATGTAAAACAGCAGTTTAAAGCAGTTCATACCTTCATCATTTTTT     1800
HEINZ          AAAGGATATATCAGAATGTAAAACAGCAGTTTAAAGCAGTTCATACCTTCATCATTTTTT     1799
LA2093         AAAGGATATATCAGAATGTAAAACAGCAGTTTAAAGCAGTTCATACCTTCATCATTTTTT     1799
               ************************************************************

E42            CCAATACTGAGTCTTCAAATTTCTGGTATCCAGCACCTACAAGATTACTCAAACTCGGGC     1860
HEINZ          CCAATACTGAGTCTTCAAATTTCTGGTATCCAGCACCTACAAGATTACTCAAACTCGGGC     1859
LA2093         CCAATACTGAGTCTTCAAATTTCTGGTATCCAGCACCTACAAGATTACTCAAACTCGGGC     1859
               ************************************************************

E42            CCTCAGATTCTCCATTTTTCTCAGCATTAGGAAACGGAGAGGGCATTGGAGGCTGGAGAG     1920
HEINZ          CCTCAGATTCTCCATTTTTCTCAGCGTTAGGAAACGGAGAGGGCATTGGAGGCTGGAGAG     1919
LA2093         CCTCAGATTCTCCATTTTTCTCAGCGTTAGGAAACGGAGAGGGCATTGGAGGCTGGAGAG     1919
               ************************* **********************************

E42            GCACAATTAAGAGAGGATTTGATAGATCCCCCAAGACATTTCCACAAGAAGCTCTCTAAA     1980
HEINZ          GCACAATTAAGAGAGGATTTGATAGATCCCCCAAGACATTTCCACAAGAAGCTCTCTAAA     1979
LA2093         GCACAATTAAGAGAGGATTTGATAGATCCCCCAAGACATTTCCACAAGAAGCTCTCTAAA     1979
               ************************************************************

E42            AAGGAGAAAAAAAAACGTTACTGATGTATTCCAAACAGTAGAGATGATCAATTAAAATCG     2040
HEINZ          AAGGAGAAAAAAAAACGTTACTGATGTATTCCAAACAGTAGAGATGATCAATTAAAATCG     2039
LA2093         AAGGAGAAAAAAAAACGTTACTGATGTATTCCAAACAGTAGAGATGATCAATTAAAATCG     2039
               ************************************************************

E42            ACACTCAATTTCCTTATATTAATCAAGAATCAGTACTTAATTTCATAAAGTACTCACAGC     2100
HEINZ          ACACTCAATTTCCTTGTATTAATCAAGAATCAGTACTTAATTTCATAAAGTACTCACAGC     2099
LA2093         ACACTCAATTTCCTTGTATTAATCAAGAATCAGTACTTAATTTCATAAAGTACTCACAGC     2099
               *************** ********************************************

E42            TGCTTCTTTAGATGACATGACAACAAGAGCAGACCCTTTCTTCTTAGAACTCTTGATTAC     2160
HEINZ          TGCTTCTTTAGATGACATGACAACAAGAGCAGACCCTTTCTTCTTAGAACTCTTGATTAC     2159
LA2093         TGCTTCTTTAGATGACATGACAACAAGAGCAGACCCTTTCTTCTTAGAACTCTTGATTAC     2159
               ************************************************************

E42            TACATCTTCAACCTCACCAAACTCACTAAACACCTCTCTTAGTCTTTGGGCAGTATAGTC     2220
HEINZ          TACATCTTCAACCTCACCAAACTCACTAAACACCTCTCTTAGTCTTTGGGCAGTATAGTC     2219
LA2093         TACATCTTCAACCTCACCAAACTCACTAAACACCTCTCTTAGTCTTTGGGCAGTATAGTC     2219
               ************************************************************

E42            TTCACCGATCTTCTCCCAAGATACCTTAAGCACCTTTTCCCTGTCTAAACTACTCCCATT     2280
HEINZ          TTCACCGATCTTCTCCCAAGATACCTTAAGCACCTTTTCCCTGTCTACACTACTCCCATT     2279
LA2093         TTCACCGATCTTCTCCCAAGATACCTTAAGCACCTTTTCCCTGTCTACACTACTCCCATT     2279
               *********************************************** ************

E42            GCCTTCTGTACAACTCTCCTTAGCTCTAGCATGTGCCTCTTTCTGTGAAGGATCTATAGG     2340
HEINZ          GCCTTCTGTACAACTCTCCTTAGCTCTAGCATGTGCCTCTTTCTGTGAAGGATCTATAGG     2339
LA2093         GCCTTCTGTACAACTCTCCTTAGCTCTAGCATGTGCCTCTTTCTGTGAAGGATCTATAGG     2339
               ************************************************************

E42            AGTAAACACTTTCTTTGAATGCATTGCACGAATTCGAGCAATCTCCTCCTTAAGTTTTCT     2400
HEINZ          AGTAAACACTTTCTTTGAATGCATTGCACGAATTCGAGCAATCTCCTCCTTAAGTTTTCT     2399
LA2093         AGTAAACACTTTCTTTGAATGCATTGCACGAATTCGAGCAATCTCCTCCTTAAGTTTTCT     2399
               ************************************************************

E42            AGCAATTCGTTCTTCCTCTTCTCGAGCTAGAACACTTGCATCAGGTGAAAAAGCTGCACG     2460
HEINZ          AGCAATTCGTTCTTCCTCTTCTCGAGCTAGAACACTTGCATCAGGTGAAAAAGCTGCACG     2459
LA2093         AGCAATTCGTTCTTCCTCTTCTCGAGCTAGAACACTTGCATCAGGTGAAAAAGCTGCACG     2459
               ************************************************************

E42            CTCTCTTGCATCGAGATCTGACATCATCTTTCTACGCTTTGAATCGTGTTGTGATTGGCG     2520
HEINZ          CTCTCTTGCATCGAGATCTGACATCATCTTTCTACGCTTTGAATCGTGTTGTGATTGGCG     2519
LA2093         CTCTCTTGCATCGAGATCTGACATCATCTTTCTACGCTTTGAATCGTGTTGTGATTGGCG     2519
               ************************************************************

E42            TTGGATCTTCTCACGTTTCACACGAAGTAGATCATCAAATAGCTTCCTCGCTTTCTCGTC     2580
HEINZ          TTGGATCTTCTCACGTTTCACACGAAGTAGATCATCAAATAGCTTCCTCGCTTTCTCGTC     2579
LA2093         TTGGATCTTCTCACGTTTCACACGAAGTAGATCATCAAATAGCTTCCTCGCTTTCTCGTC     2579
```

```
            ************************************************************

E42         CTTTAGAATCTCGTATGAAGTTTTCAGCTTTTGAAAGTTCAAGTGAGCATTTGGGTCATC    2640
HEINZ       CTTTAGAATCTCGTATGAAGTTTTCAGCTTTTGAAAGTTCAAGTGAGCATTTGGGTCATC    2639
LA2093      CTTTAGAATCTCGTATGAAGTTTTCAGCTTTTGAAAGTTCAAGTGAGCATTTGGGTCATC    2639
            ************************************************************

E42         ACGCCTCTTGTCTGGATGCAACTCTAATGCTTTCTTCTTATAGGCTTTAGATATGTCTTT    2700
HEINZ       ACGCCTCTTGTCTGGATGCAACTCTAATGCTTTCTTCTTATAGGCTTTAGATATGTCTTT    2699
LA2093      ACGCCTCTTGTCTGGATGCAACTCTAATGCTTTCTTCTTATAGGCTTTAGATATGTCTTT    2699
            ************************************************************

E42         CTCAGAAAGTTGGGACCCTTCCTCACCAGAGGGTAAACCCAAAGCAGCATAATGATCAAC    2760
HEINZ       CTCAGAAAGTTGGGACCCTTCCTCACCAGAGGGTAAACCCAAAGCAGCATAATGATCAAC    2759
LA2093      CTCAGAAAGTTGGGACCCTTCCTCACCAGAGGGTAAACCCAAAGCAGCATAATGATCAAC    2759
            ************************************************************

E42         TTCAATATCCATTTTCTACCCCGGAATTTGAACTGTGCAAC 2801
HEINZ       TTCAATATCCATTTTCTACCCCGGAATTTGAACTGTGCAAC 2800
LA2093      TTCAATATCCATTTTCTACCCCGGAATTTGAACTGTGCAAC 2800
            *****************************************
```

Solyc02g088610

```
E42         CTAAGTCGACCTTCATTCTTATATATGATTAAAAAAAAACATGGGTGAATAACCGTTAAC    60
HEINZ       CTAAGTCGACCTTCATTCTTATATATGATTAAAAAAAAACATGGGTGAATAACCGTTAAC    60
LA2093      CTAAGTCGACTTTCATTCTTATATATGATTAAAAAAAAACATGGGTGAATAACCGTTAAA    60
            ********** ************************************************ 

E42         TTTCTATAGACTAATAAGCTATTTATTATACGAAAACAGAACTCCACACTGTTCAACTGT    120
HEINZ       TTTCTATAGACTAATAAGCTATTTATTATACGAAAACAGAACTCCACACTGTTCAACTGT    120
LA2093      TTTCTATAGACTAATAAGCTATTTATTATACGAAAACAGAACTCCACACTGTTCAACTGT    120
            ************************************************************

E42         GTAGGTATTTGCTATTCAAAGCCGTGAAATATGTATATCCATAATCTAGATACAAGAGCT    180
HEINZ       GTAGGTATTTGCTATTCAAAGCCGTGAAATATGTATATCCATAATCTAGATACAAGAGCT    180
LA2093      GTAGGTATTTGCTATTCAAAGCCGTGAAATATGTATATCCATAATCTAGATACAAGAGCT    180
            ************************************************************

E42         ACTACTAGTTCGATGCTCGAAATCATAGTATGTGTCTAAACAAGATGTTGAGGTGTAGTC    240
HEINZ       ACTACTAGTTCGATGCTCGAAATCATAGTATGTGTCTAAACAAGATGTTGAGGTGTAGTC    240
LA2093      ACTACTAGTTCGATGCTCGAAATCATAGTATGTGTCTAAACAAGATGTTGAGGTGTAGTC    240
            ************************************************************

E42         TCATAGTTTCTGAGAGAAAGCTTCTTGGTTCTCAGCTGGAGAATCTGAACCAGACTCTTG    300
HEINZ       TCATAGTTTCTGAGAGAAAGCTTCTTGGTTCTCAGCTGGAGAATCTGAACCAGACTCTTG    300
LA2093      TCATAGTTTCTGAGAGAAAGCTTCTTGGTTCTCAGCTGGAGAATCTGAACCAGACTCTTG    300
            ************************************************************

E42         TCTTTTAAACACTAGCTTCTGCTGAGGAAGTTGTCCATTCGAAAATGCGCTGACCTCCGT    360
HEINZ       TCTTTTAAACACTAGCTTCTGCTGAGGAAGTTGTCCATTCGAAAATGCGCTGACCTCCGT    360
LA2093      TCTTTTAAACACTAGCTTCTGCTGAGGAAGTTGTCCATTGGAAAATGCGCTGACCTCCGT    360
            *************************************** ********************

E42         GTCTACTAAAATAGTATCCTCATCCTTGAATTCTCCTCTTAATATTCCTTTGGCCAATTC    420
HEINZ       GTCTACTAAAATAGTATCCTCATCCTTGAATTCTCCTCTTAATATTCCTTTGGCCAATTC    420
LA2093      GTCTACTAAAATAGTATCCTCATCCTTGAATTCTCCTCTTAATATTCCTTTGGCCAATTC    420
            ************************************************************

E42         GTTCTCAACATTCTGCTGGATCACCCGCTTCACTGGCCGTGCTCCATAGTTGGGGTCATA    480
HEINZ       GTTCTCAACATTCTGCTGGATCACCCGCTTCACTGGCCGTGCTCCATAGTTGGGGTCATA    480
LA2093      GTTCTCAACATTCTGCTGGATCACCCGCTTCACTGGCCGTGCTCCATAGTTGGGGTCATA    480
            ************************************************************

E42         ACCAAGACTTCCAAGCAATTGAATAGCAGCTTCACTCACTTGAATCTTCATTTTACGATC    540
HEINZ       ACCAAGACTTCCAAGCAATTGAATAGCAGCTTCACTCACTTGAATCTTCATTTTACGATC    540
LA2093      ACCAAGACTTCCAAGCAATTGAATAGCAGCTTCACTCACTTGAATCTTCATTTTACGATC    540
            ************************************************************

E42         AGCTAATCTCTGCTGCACTCTTTCAAGCTGCAACAACAAAAAATAGCTCAAACTCAGAGA    600
HEINZ       AGCTAATCTCTGCTGCACTCTTTCAAGCTGCAACAACAAAAAATAGCTCAAACTCAGAGA    600
LA2093      AGCTAATCTCTGCTGAACTCTTTCAAGCTGCAACAACAAAAAATAGCTCAAACTCAGAGA    600
            *************** ********************************************

E42         CATACATTTATATCCAGACACAGCATCAACTTCCAGCATTGACATTACCAAGAAGTATAT    660
HEINZ       CATACATTTATATCCAGACACAGCATCAACTTCCAGCATTGACATTACCAAGAAGTATAT    660
LA2093      CATACATTTATATCCAGACACAGCATCAACTTCCAGCATTGACACTACCAGAAAGTATAT    660
            ******************************************** *****  ********

E42         GCAAACGCAAACAAACATGTGTATTTGAGATTGGAAAAGGTATGTTACCTGTAATCGCAC    720
HEINZ       GCAAACGCAAACAAACATGTGTATTTGAGATTGGAAAAGGTATGTTACCTGTAATCGCAC    720
LA2093      GCAAACGCAAACAAGCATGTGTTTTTAAGATTGGAAAAGGTATGTTACCTGCAATCGCAC    720
            ************** ******* *** ************************ ********

E42         AATGCTACTGATCTGGTCACGATCAAGAGGCTGGAATACTATATATTCATCAACCCGGTT    780
HEINZ       AATGCTACTGATCTGGTCACGATCAAGAGGCTGGAATACTATATATTCATCAACCCGGTT    780
LA2093      AATGCTACTGATTTGGTCACGATCAAGAGGCTGGAATACTATATATTCATCAACCCGGTT    780
            ************ ***********************************************

E42         CATGAACTCAGGGCGGAAAACTGCACGTGCAGCATCCATCACCCTTTGCTTTATTGTTTG    840
HEINZ       CATGAACTCAGGGCGGAAAACTGCACGTGCAGCATCCATCACCCTTTGCTTTATTGTTTG    840
LA2093      CATGAACTCGGGGCGGAAAACTGCACGTGCAGCATCCATCACCCTTTGCTTTATTGTTTG    840
            ********* **************************************************
```

```
E42         ATAAGTCGCTTCCTTCAATGAATCATCATCATTATCATCTGTATTTAGGATATATTGTGA    900
HEINZ       ATAAGTCGCTTCCTTCGATGAATCATCATCATTATCATCTGTATTTAGGATATATTGTGA    900
LA2093      ATAAGTCGCTTCCTTCGATGAATCATCATCATTATCATCTGTATTTAGGATATATTGTGA    900
            **************** *******************************************

E42         ACCAACATTTGATGTCATGATGATGACAGTATTGGTGAAACTCACTGTGCGTCCTTGCGA    960
HEINZ       ACCAACATTTGATGTCATGATGATGACAGTATTGGTGAAACTCACTGTGCGTCCTTGCGA    960
LA2093      ACCAACATTTGATGTCATGATGATGACAGTATTGGTGAAACTCACTGTGCGTCCTTGCGA    960
            ************************************************************

E42         GTCGGTCACCCTACCATCATCTAAGATTTGCAGGAACACATTAAATACATCCGAATGGGC    1020
HEINZ       GTCGGTCACCCTACCATCATCTAAGATTTGCAGGAACACATTAAATACATCCGAATGGGC    1020
LA2093      GTCGGTCACCCTACCATCATCTAAGATTTGCAGGAACACATTAAATACATCCGAATGGGC    1020
            ************************************************************

E42         CTTCTCAATTTCATCAAACAATATAACTGCATAAGGTCTTCGGCGAACAATCTCTGTCAA    1080
HEINZ       CTTCTCAATTTCATCAAACAATATAACTGCATAAGGTCTTCGGCGAACAATCTCTGTCAA    1080
LA2093      CTTCTCAATTTCATCAAACAATATAACTGCATACGGTCTTCGGCGAACAATCTCTGTCAA    1080
            ********************************* **************************

E42         CTGCCCCCCTTCTTCATACCCGACATAACCAGGGGGAGCTCCTATCAATCTTGAAACTGC    1140
HEINZ       CTGCCCCCCTTCTTCATACCCGACATAACCAGGGGGAGCTCCTATCAATCTTGAAACTGC    1140
LA2093      CTGACCCCCTTCTTCATACCCGACATAACCAGGGGGAGCTCCTATCAATCTTGAAACTGC    1140
            *** ********************************************************

E42         ATGCTTCTCCATGTATTCACTCATATCGATGCGCACAAGTGCCTCTTCAGTATTGAACAA    1200
HEINZ       ATGCTTCTCCATGTATTCACTCATATCGATGCGCACAAGTGCCTCTTCAGTATTGAACAA    1200
LA2093      ATGCTTCTCCATGTATTCACTCATATCGATGCGCACAAGTGCCTCTTCAGTATTGAACAA    1200
            ************************************************************

E42         ATAGTTAGCAAGGGCCTTAGCTAACTCCGTCTTTCCAACTCCAGTGGGACCCATGAACAT    1260
HEINZ       ATAGTTAGCAAGGGCCTTAGCTAACTCCGTCTTTCCAACTCCAGTGGGACCCATGAACAT    1260
LA2093      ATAGTTAGCAAGGGCCTTAGCTAACTCCGTCTTTCCAACTCCAGTGGGACCCATGAACAT    1260
            ************************************************************

E42         AAAGCTTGCAATAGGACGATGAGGATCTGAAAGTCCAGCACGCGAGCGCTGGATTGCCTC    1320
HEINZ       AAAGCTTGCAATAGGACGATGAGGATCTGAAAGTCCAGCACGCGAGCGCTGGATTGCCTC    1320
LA2093      AAAGCTTGCAATAGGACGATGAGGATCTGAAAGTCCAGCACGCGAGCGCTGGATTGCCTC    1320
            ************************************************************

E42         TGCTACTGCTCTTACTGCTGGATCTTGACCAACAACACGTTTGTGCAACTCTTCCTCTAA    1380
HEINZ       TGCTACTGCTCTTACTGCTGGATCTTGACCAACAACACGTTTGTGCAACTCTTCCTCTAA    1380
LA2093      TGCTACTGCTCTTACTGCTGGATCTTGACCAACAACACGTTTGTGCAACTCTTCCTCTAA    1380
            ************************************************************

E42         ATGCAACAACTTTTCCCTCTCCGACTGTTGTAGCTTAGAAACAGGAATCCCAGTCCACTT    1440
HEINZ       ATGCAACAGCTTTTCCCTCTCCGACTGTTGTAGCTTAGAAACAGGAATCCCAGTCCACTT    1440
LA2093      ATGCAACAGCTTTTCCCTCTCCGACTGTTGTAGCTTAGAAACAGGAATCCCAGTCCACTT    1440
            ******** ***************************************************

E42         ACTGACAATCTCTGCAATATCATTCCCAGTTACTTCTTCTCTGAGCATCGATTTTCCAGA    1500
HEINZ       ACTAACAATCTCTGCAACATCATTCCCAGTTACTTCTTCTCTGAGCATCGATTTTCCAGA    1500
LA2093      ACTGACAATCTCTGCAATATCATTCCCAGTTACTTCTTCTCTGAGCATCGATTTTCCAGA    1500
            *** ************* ******************************************

E42         CTTCATATAATCACTCAGTTCTTTTTCTGAAGCTTCAAGTTGACGCTGCAAGGTGTTTAG    1560
HEINZ       CTTCATATAATCACTCAGTTCTTTTTCTGAAGCTTCAAGTTGACGCTGCAAGGTGTTTAG    1560
LA2093      CTTCATATAATCACTCAGTTCTTTTTCTGAAGCTTCAAGTTGACGCTGCAAGGTGTTTAG    1560
            ************************************************************

E42         ACTCCCATACTTAAGTTCAGCAGCACGATTAAGATCATATTCCCGCTCTGCTTGCTGGAT    1620
HEINZ       ACTCCCATACTTAAGTTCAGCAGCACGATTAAGATCATATTCCCGCTCTGCTTGCTGGAT    1620
LA2093      ACTCCCGTACTTAAGTTCAGCAGCACGATTAAGATCATATTCCCGCTCTGCTTGCTGGAT    1620
            ****** *****************************************************

E42         CTCAAGATTTACCCGGTCGATCTGTATTTAGGAAGAAAGTTAATCAAGCTCCACTGCAAT    1680
HEINZ       CTCAAGATTTACCCGGTCGATCTGTATTTAGGAAGAAAGTTAATCAAGCTCCACTGCAAT    1680
LA2093      CTCAAGATTTACCCGGTCGATCTGTATTTAGGAAGAAAGTTAATCAAGCTCCACTGCAAT    1680
            ************************************************************

E42         CTAATTCATTTACATACGAATTTCTCCTTAGCTAATTCAATTAACAGCTCAGAATATAAA    1740
```

```
HEINZ          CTAATTCATTTACATACGAATTTCTCCTTAGCTAATTCAATTAACAGCTCAGAATATAAA     1740
LA2093         CTAATTCATTTACATACGAATTTCTCCTTAGCTAATTCAATTAACAGCTCAGAATATAAA     1740
               ************************************************************

E42            TGCACTTCCACTCAAGAGTAGTTATTAACAAAAGAAACAACAGTTATGAACAAACTTCAG     1800
HEINZ          TGCACTTCCACTCAAGAGTAGTTATTAACAAAAGAAACAACAGTTATGAACAAACTTCAG     1800
LA2093         TGCACTTCCACTCAAGAGTAGTTAAA--CAAAAGAAACACCAGTTATGAACAAACTTTAG     1798
               ************************    *********** ***************** **

E42            CTGCGTACCTCTTCCTTAATGGACTGCAAGCGAGTCATCACACTCTTTTCATGCTCCCAC     1860
HEINZ          CTGCGTACCTCTTCCTTAATGGACTGCAAGCGAGTCATCACACTCTTTTCATGCTCCCAC     1860
LA2093         CTGCGTACCTCTTCCTTAATGGACTGCAAGCGAGTCATCACACTCTTTTCATGCTCCCAC     1858
               ************************************************************

E42            TGCTCAGTCAGCTCAGCTTGCCTCTCCTTCAGAAGAGACAATTCAGTCTCAAGACGGTTT     1920
HEINZ          TGCTCAGTCAGCTCAGCTTGCCTCTCCTTCAGAAGAGACAATTCAGTCTCAAGACGGTTT     1920
LA2093         TGCTCAGTCAGCTCAGCTTGCCTCTCCTTCAAAAGAGACAACTCAGTCTCAAGACGGTTT     1918
               ******************************* ********* ******************

E42            AACCGGTCTTTCGATGCTTTATCTGTATCATTAGTTAGAGATAGCCTCTCCATCTCCAAC     1980
HEINZ          AACCGGTCTTTCGATGCTTTATCTGTATCATTAGTTAGAGATAGCCTCTCCATCTCCAAC     1980
LA2093         AACCGGTCTTTCGATGCTCTATCTGTATCATTAGTTAGAGATAGCCTCTCCATCTCCAAC     1978
               ****************** *****************************************

E42            TTCAAAACTGCACGATTGATCTCGTCAAGGGCTGTAGGTTTTGAGGTGATCTCCATTTTC     2040
HEINZ          TTCAAAACTGCACGATTGATCTCGTCAAGGGCTGTAGGTTTTGAGGTGATCTCCATTTTC     2040
LA2093         TTCAAAACTGCACGATTGATCTCGTCAAGGGCTGTAGGTTTTGAGGTGATCTCCATTTTC     2038
               ************************************************************

E42            AGTTTTGCAGCAGCTTCATCAACTAGGTCAATAGCTGCAATTTGAAAACCAGCACTTTTA     2100
HEINZ          AGTTTTGCAGCAGCTTCATCAACTAGGTCAATAGCTGCAATTTGAAAACCAGCACTTTTA     2100
LA2093         AGTTTTGCAGCAGCTTCATCAACTAGGTCAATAGCTGCAATTTGAAAACCAGCACTTTTA     2098
               ************************************************************

E42            GCATAGACTATAAGTTCAATAAGA-ACTAGTCTATTATACTCCACATATTGTGCAGGTCA     2159
HEINZ          GCATAGACTATAAGTTCAATAAGA-ACTCGTCTATTATACTCCACATATTGTGCAGGTCA     2159
LA2093         GCATAGACTATAAGTTCAATAAGAACCTAGTCTATTATACTCCACATTTTGTGCAGGTCA     2158
               ************************  ** ****************** ************

E42            AGTGCATGAGCACTTGGTGTATGCTTACTGGACCAAGGGAAACAACAATGTGGTGATGAA     2219
HEINZ          AGTGCATGAGCACTTGGTGTATGCTTACTGGACCAAGGGAAACAACAATGTGGTGATGAA     2219
LA2093         AGTGCATGAGCACTTGGAGCATGCTTACTGAC-CAAGGGAAACAATAATGTGGTGATGGA     2217
               ***************** * **********   ************ ************ *

E42            AGACTGAACGGTATGCTTTGATATGATCCAGAAAATGTGGCTACATCACCGCTCAAGTTG     2279
HEINZ          AGACTGAACGGTATGCTTTGATATGATCCAGAAAATGTGGCTACATCACCGCTCAAGTTG     2279
LA2093         AGACTGAACGGTATGCTTTGATATGATCCAGAAAATGTGGCTACATCACCGCTCAAGTTG     2277
               ************************************************************

E42            TTCCTTTCTATAGTATTTTGAGAAGGTCACGCTCAAATGTCGAAGTGATGATTAGTAGTT     2339
HEINZ          TTCCTTTCTATAGTATTTTGAGAAGGTCACGCTCAAATGTCGAAGTGATGATTAGTAGTT     2339
LA2093         TTCCTTTCTATAGTATTTTGAGAAGGCCACGCTCAAATGTTGAAGTGATGATTAGTAGTT     2337
               ************************** ************* *******************

E42            TGATAGGATTAAAAATCCACCAGCAGGACTAAGGTTAGGCCAAACAAATGCAAAATTGTT     2399
HEINZ          TGATAGGATTAAAAATCCACCAGCAGGACTAAGGTTAGGCCAAACAAATGCAAAATTGTT     2399
LA2093         TGATAGGATTAATAATCCACCAGCAGGACTAAGGTTAGGCCAAACAAATGCAAAATTGTC     2397
               ************ ********************************************** 

E42            TCCTAAATTTTAAATCAAAATTACACAAGCAGGACCACTATAGTCGCCCAATTGAGTACT     2459
HEINZ          TCCTAAATTTTAAATCAAAATTACACAAGCAGGACCACTATAGTCGCCCAATTGAGTACT     2459
LA2093         TCCTAAATTTTAAATCAAAATTACACAAGCAGGGCCACCGCAGTCACCTAATTGGGTACT     2457
               ********************************* ****   **** ** ***** *****

E42            AGAACTTTCACCTTGTTGATAAGAGTTCGGTTCCCTTCTGGTAATCCCTCCCCCGCCAGC     2519
HEINZ          AGAACTTTCACCTTGTTGATAAGAGTTCGGTTCCCTTCTGGTAATCCCTCCCCCGCCAGC     2519
LA2093         AGAACTTTCACCTTGTTGATAAGAGTTCGGTTCCCTTCTGGTAATCCCTCCCCCGCCCGC     2517
               ********************************************************* **

E42            TCTATTTGTAATAAATTAAAATAAAAACTAGACAATAAAGTTATATCACCATGAACTATA     2579
HEINZ          TCTATTTGTAATAAATTAAAATAAAAACTAGACAATAAAGTTATATCACCATGAACTATA     2579
LA2093         TCTATTTGTAGTAAATTAAAATAAAAACTAGACAATAAAGTTATATCACCATGAACGATA     2577
```

```
                **********  ********************************************* ***

E42             CAGTTGCTCTACAGTGAACAGCTTTTCAGTTTCTCACCTTTGTCAGGTAGAAAACGTCCA    2639
HEINZ           CAGTTGCTCTACAGTGAACAGCTTTTCAGTTTCTCACCTTTGTCAGGTAGAAAACGTCCA    2639
LA2093          CAGTTGCTCTATAGTGAACAGCTTTTCAGTTTCTCACCTTTGTCAGGTAGAAAACGTCCA    2637
                *********** ************************************************

E42             CTAATATAACGGTCCGAAAGAATAGCTGCGTCGACAAGCGCAGTGTCTGAAATGCGAACC    2699
HEINZ           CTAATATAACGGTCCGAAAGAATAGCTGCGTCGACAAGCGCAGTGTCTGAAATGCGAACC    2699
LA2093          CTAATATAACGGTCCGAAAGAATAGCTGCGTCGACAAGCGCAGTGTCTGAAATGCGAACC    2697
                ************************************************************

E42             CCGTGATGCAGCTCATATCTTTCACGTAATCCGCGAAGTATGGAGACAGTGTCTTCAACT    2759
HEINZ           CCGTGATGCAGCTCATATCTTTCACGTAATCCGCGAAGTATGGAGACAGTGTCTTCAACT    2759
LA2093          CCGTGATGCAGCTCATATCTTTCACGTAATCCGCGAAGTATGGAGACAGTGTCTTCAACT    2757
                ************************************************************

E42             GTAGGCTGGTCAACATAAACTTGCTGGAAACGACGCTCCAGGGCAGGATCTTTCTCAATA    2819
HEINZ           GTAGGCTGGTCAACATAAACTTGCTGGAAACGACGCTCCAGGGCAGGATCTTTCTCAATA    2819
LA2093          GTAGGCTGGTCAACATAAACTTGCTGGAAACGACGCTCCAGGGCAGGATCTTTCTCAATA    2817
                ************************************************************

E42             TATTTACGGTATTCATCCAAGGTGGTCGCACCAATACATCGTAGTTCTCCTCGACCAAGC    2879
HEINZ           TATTTACGGTATTCATCCAAGGTGGTCGCACCAATACATCGTAGTTCTCCTCGACCAAGC    2879
LA2093          TATTTACGGTATTCATCCAAGGTGGTTGCACCAATACATCGTAGTTCTCCTCGACCAAGC    2877
                ************************** *********************************

E42             ATCGGCTTCAATAGATTCCCAGCATCCATTGCACCATTAGTAGCACCTGATGAAAACAAA    2939
HEINZ           ATCGGCTTCAATAGATTCCCAGCATCCATTGCACCATTAGTAGCACCTGATGAAAACAAA    2939
LA2093          ATCGGCTTCAATAGATTCCCAGCATCCATTGCACCATTAGTAGCACCTGACGAAAACAAA    2937
                ************************************************** *********

E42             TGATTTTGTTTTTTCACAAACCAAACCAGAAAAAAATATATCCAAACTTGGATGAACCCA    2999
HEINZ           TGATTTTGTTTTTTCACAAACCAAACCAGAAAAAAATATATCCAAACTTGGATGAACCCA    2999
LA2093          TGATTTTGTTTTTTCACAAACCAAACCAGAAAAGAATATATCCAAACTTGGATGAACCCA    2997
                ********************************* **************************

E42             AATAATGGTACGGAATACAACTTCTGGTGTAGGATGAAATCATAATGATCCCAGTATGTA    3059
HEINZ           AATAATGGTACGGAATACAACTTCTGGTGTAGGATGAAATCATAATGATCCCAGTATGTA    3059
LA2093          AATGATGGTATGGAATACAACTTCTGGTGTAGGATGAAATCATAATGATCCCAGTATGTA    3057
                *** ****** *************************************************

E42             GCAGACGTATAGGCCAATAACATGGTTAATTATAGGTCCATGCTCAAGTGAAAGGGCAAC    3119
HEINZ           GCAGACGTATAGGCCAATAACATGGTTAATTATAGGTCCATGCTCAAGTGAAAGGGCAAC    3119
LA2093          GCAGACGTATAGGCCAATAACATGGGCAATTATAGGTCCATGCTCAAGTGAAAGGGCAAC    3117
                ************************  **********************************

E42             AAGTATTAATTTTTAATCACCGGCATTCCAACGTATAAAACAGCATTTTAACATCTGGTG    3179
HEINZ           AAGTATTAATTTTTAATCACCGGCATTCCAACGTATAAAACAGCATTTTAACATCTGGTG    3179
LA2093          AAGTATTAATTTTTAATCACCGGCATTCCTACGTATAAAACAGCATTTTAACATCTGGTG    3177
                ***************************** ******************************

E42             ATGTTCCCTAAGAACATACCTGCACCAACAACAGTATGTATCTCATCAATAAAAAGGATG    3239
HEINZ           ATGTTCCCTAAGAACATACCTGCACCAACAACAGTATGTATCTCATCAATAAAAAGGATG    3239
LA2093          ATGTTCCCTAAGAACATACCTGCACCAACAACAGTATGTATCTCATCAATAAAAAGGATG    3237
                ************************************************************

E42             ATCTGTCCTTCAGATTCTGTCACTTCCTTAAGCACTGCCTTCAATCTGTCTTCAAATTCA    3299
HEINZ           ATCTGTCCTTCAGATTCTGTCACTTCCTTAAGCACTGCCTTCAATCTGTCTTCAAATTCA    3299
LA2093          ATCTGTCCTTCTGATTCTGTCACTTCCTTAAGCACTGCCTTCAATCTGTCTTCAAATTCA    3297
                *********** ************************************************

E42             CCACGATATTTTGCACCAGCTATGAGCGCACCCATATCAAGAGATATCAACTGCACAGAG    3359
HEINZ           CCACGATATTTTGCACCAGCTATGAGCGCACCCATATCAAGAGATATCAACTGCACAGAG    3359
LA2093          CCACGATATTTTGCACCAGCTATGAGCGCACCCATATCAAGAGATATCAACTGCACAGAG    3357
                ************************************************************

E42             GACGCAAACATGTTAATTGATAAAATACTTTCTTTCATAAGTCAGGTGAATGCAACAGAA    3419
HEINZ           GACGCAAACATGTTAATTGATAAAATACTTTCTTTCATAAGTCAGGTGAATGCAACAGAA    3419
LA2093          AACGCAAACATGTTAATTCATAAAATAGTTTCTTTCACAAGTCAGGTGAATGCAACAGAA    3417
                 ***************** ******** ********* **********************
```

```
E42         ATTCCCTTCATCACTAACCCTTCGATTCATCAAAGCCTGAGGTACATCTCCTTGCACAAT    3479
HEINZ       ATTCCCTTCATCACTAACCCTTCGATTCATCAAAGCCTGAGGTACATCTCCTTGCACAAT    3479
LA2093      ATCCCCTTCATCACAAACCCTTCGATTCATCAAAGCCTGAGGTACATCTCCTTGCACAAT    3477
            ** *********** *********************************************

E42         TCTCTGAGCAAGCCTATCAAACAGGAAGAGAAGAATATAAGTTGTACGCAA---------    3530
HEINZ       TCTCTGAGCAAGCCTATCAAACAGGAAGAGAAGAATATAAGTTATACGCAA---------    3530
LA2093      CCTCTGAGCAAGCCTATCAAACAGGAAGAGAAGAATATAAGTTAAGCCTATCAAACAGGA    3537
             ******************************************   *  *

E42         --------------------------TGGATAAGTATTTTTTGAAAAAATATAACAAATT    3564
HEINZ       --------------------------TGGATAAGTATTTTTTGAAAAAATATAACAAATT    3564
LA2093      AGAGAAGAATATAAGCTATACGCAATGAATGAAGTGCTTTTTGAAAAAATATAACAAATT    3597
                                           ****  ***********************

E42         GAGACCAATGGAACATGTTCCAAATGAAACATACAAATAAAAATTTAAAATCACATGCTC    3624
HEINZ       GAGACCAATGGAACATGTTCCAAATGAAACATACAAATAAAAATTTAAAATCACATGCTC    3624
LA2093      GAGACCAATGGAACATGTTCCAAATGAGACATACAAATAAAAAATTAAAATCACATGCTC    3657
            *************************** *************** ****************

E42         CAACTCCCGCTCTACTGCTCAATAAGAAGAATTTACTTACCCTTCAGAGATTGCAGTTTT    3684
HEINZ       CAACTCCCGCTCTACTGCTCAATAAGAAGAATTTACTTACCCTTCAGAGATTGCAGTTTT    3684
LA2093      CAACTCCCGCTCTACTGCTCAATAAGTAAAATTTACTTACCCTTCAGAGATTGCAGTTTT    3717
            ************************** * ******************************

E42         CCCAACTCCAGGCTCACCAATTAACACTGGGTTATTCTTTGTTCTCCTTGAGAGGATTTG    3744
HEINZ       CCCAACTCCAGGCTCACCAATTAACACTGGGTTATTCTTTGTTCTCCTTGAGAGGATTTG    3744
LA2093      CCCAACTCCAGGCTCACCAATTAACACTGGGTTATTCTTTGTTCTCCTTGAGAGGATTTG    3777
            ************************************************************

E42         TATGCATCTACGTATCTCATCATCTCTTCCTATAACTGGGTCAAGCTTCCCCGCTCTAGC    3804
HEINZ       TATGCATCTACGTATCTCATCATCTCTTCCTATAACTGGGTCAAGCTTCCCCGCTCTAGC    3804
LA2093      TATGCATCTACGTATCTCATCATCTCTTCCTATAACTGGGTCAAGCTTCCCCGCTCTAGC    3837
            ************************************************************

E42         CATAGCAGTCAGGTCTTTTCCATATTTTTCTAGTGATTCATACTTCCCTTCAGGATCTGC    3864
HEINZ       CATAGCAGTCAGGTCTTTTCCATATTTTTCTAGTGATTCATACTTCCCTTCAGGATCTGC    3864
LA2093      CATAGCAGTCAGGTCTTTTCCATATTTTTCTAGTGATTCATACTTCCCTTCAGGATCTGC    3897
            ************************************************************

E42         AAGAATGATAAACAAGATATTTGAGAAATGGAAAAGGCTAACAGGATATATTGCAGACGA    3924
HEINZ       AAGAATGATAAACAAGATATTTGAGAAATGGAAAAGGCTAACAGGATATATTGCAGACGA    3924
LA2093      AAGAATGATAAACAAGATATTCGAGAAATGGAAAAAGCTAACAGGATATATTGCAGACGA    3957
            ********************* ************** ***********************

E42         GCATGTGTTGGGAAAGTAAGGTTGTTGTTAACAAATAATCTAATGGTGTATACCAGTCAA    3984
HEINZ       GCATGTGTTGGGAAAGTAAGGTTGTTGTTAACAAATAATCTAATGGTGTATACCAGTCAA    3984
LA2093      GCAAGTGTTGGGA-----------------ACAAATAATCTAATGGTGTATACCATTCAA    4000
            *** *********                 ************************* ****

E42         ACTACGTATAAAACATTAAGGACTACACTAGGAAGAAGCTCCCTGTATCACATGAAAGGG    4044
HEINZ       ACTACGTATAAAACATTAAGGACTACACTAGGAAGAAGCTCCCTGTATCACATGAAAGGG    4044
LA2093      ACTACGTATAAAACATT-AAGGCTACACTAGGAAGAAGCTCCCTGTATCACATGAAAGGG    4059
            ***************** * * **************************************

E42         AGGAGATCCAAGTGTAATGAACATAATATAGTCCATAAAGGGGCAACTACGTCTTAATTC    4104
HEINZ       AGGAGATCCAAGTGTAATGAACATAATATAGTCCATAAAGGGGCAACTACGTCTTAATTC    4104
LA2093      AGGAGATCCAAGTGTAATGAACATAATATAGTCCATAAAGGGGTAACTACGTCTTAATTC    4119
            ******************************************* ****************

E42         TTGCACTTATTGACAAGACCCACATATGCAAGTAGTCCACCAATTATAGAAAGAACTAAT    4164
HEINZ       TTGCACTTATTGACAAGACCCACATATGCAAGTAGTCCACCAATTATAGAAAGAACTAAT    4164
LA2093      TTGCACTTATTGACAAGACCCACATATGCAAGTAGTCCACCAATTATAGAAAGACCTTAT    4179
            ****************************************************** ** **

E42         TACCTTGATCAATAACATTTTGGCGTCCCCTTATGGATTCAATGGCAGTTTTCAGTGTCT    4224
HEINZ       TACCTTGATCAATAACATTTTGGCGTCCCCTTATGGATTCAATGGCAGTTTTCAGTGTCT    4224
LA2093      TACCTTGATCAATAACATTTTGGCGTCCCCTTATGGATTCAATGGCAGTTTTCAGTGTCT    4239
            ************************************************************

E42         TCAGGGAAATCTGGAAATCATTAAACAGTTGCTTCCCAAATCGTTTATCTTGAATGAAGC    4284
HEINZ       TCAGGGAAATCTGGAAATCATTAAACAGTTGCTTCCCAAATCGTTTATCTTGAATGAAGC    4284
```

```
LA2093      TCAGGGAAATCTGGAAATCATTAAACAGTTGCTTCCCAAATCGTTTATCTTGAATGAAGC    4299
            ************************************************************

E42         CAAGCACCAAATGCTCAACTGACACAAAGGAATCACCATACTCTTTCTTGTACTCCCTGG    4344
HEINZ       CAAGCACCAAATGCTCAACTGACACAAAGGAATCACCATACTCTTTCTTGTACTCCCTGG    4344
LA2093      CAAGCACCAAATGCTCAATTGACACAAAGGAATCACCATACTCTTTCTTGTACTCCCTGG    4359
            ****************** *****************************************

E42         CTCTCTGCATCAGACCTTCCAACTCTCGGCCTAACATAGAACCTGCTGTCTCACCAATAA    4404
HEINZ       CTCTCTGCATCAGACCTTCCAACTCTCGGCCTAACATAGAACCTGCTGTCTCACCAATAA    4404
LA2093      CTCTCTGCATCAAACCTTCCAACTCTCGGCCTAACATAGAACCTGCTGTCTCACCAATAA    4419
            ************ ***********************************************

E42         CCTGGAAAGATGT-GTAACCAAGAAGATGATTCAGCAATCTATAGTACATGAGAGGACAA    4463
HEINZ       CCTGGAAAGATGT-GTAACCAAGAAGATGATTCAGCAATCTATAGTACATGAGAGGACAA    4463
LA2093      CCTGGAAAGATATTGTAACCAGGAAGATGATTCAGCAATCTATAGTACATGAGAGGACAA    4479
            *********** * ******* **************************************

E42         TGTATTTAGCAGCTTGTTGTTTGATTGTTATTAGAAGTGATCTAAAGAGAGAGGAAGTAA    4523
HEINZ       TGTATTTAGCAGCTTGTTGTTTGATTGTTATTAGAAGTGATCTAAAGAGAGAGGAAGTAA    4523
LA2093      TGTATTTAGCAGCTTGTTGTTTGATTGCTATTAGAAGTGATCTAAAGAGAGAGGAAGTAA    4539
            *************************** ********************************

E42         TATCATAAAAGAACTAATTTGAGTGACTCAGCATCACTGGTGGTTGGAAGCTTCCTATAA    4583
HEINZ       TATCATAAAAGAACTAATTTGAGTGACTCAGCATCACTGGTGGTTGGAAGCTTCCTATAA    4583
LA2093      TATCATAAAAAAACTAATTCGAATGACTCAGCATCACTGGTGGTTGGAAGCTTCCTATAA    4599
            ********** ******** ** *************************************

E42         TATG--TGTGCTCTCTTAAAGAACAATGTACAAGAATCAGTTGACGAAACTACCATGGAA    4641
HEINZ       TATG--TGTGCTCTCTTAAAGAACAATGTACAAGAATCAGTTGACGAAACTACCATGGAA    4641
LA2093      TATATGTGTGCTCTCTTAAAGAACAATGTACGAGAATCAGTTGACGAAACTACCATGGAA    4659
            ***   ************************* ****************************

E42         TCGAAACGTAAGTGCATAGCACTGACTAAGTTTCCATACGTTCTGCATACATCAACTATA    4701
HEINZ       TCGAAACGTAAGTGCATAGCACTGACTAAGTTTCCATACGTTCTGCATACATCAACTATA    4701
LA2093      TCGAAACAAAAGTGCATAGCACTGAGTAAATTTCCATACGTTCTGCATACATCAACTATA    4719
            *******  **************** *** ******************************

E42         CGAGCTGAGTCAAGCTCCGCTATAGACGAGAAAGAGCCAAATTCTAGCTTGCTTGGAGAT    4761
HEINZ       CGAGCTGAGTCAAGCTCCGCTATAGACGAGAAAGAGCCAAATTCTAGCTTGCTTGGAGAT    4761
LA2093      CGAGCTGAGCCAAGCTCAGCTATAGATGAGAAAGAGCCAAATTCTAGCTTGCTTGGAGAT    4779
            ********* ******* ******** *********************************

E42         ATGGTGTTCAGATATAGTTGATATCAAGCTGGATTATAAGCTCATCTATAGCCAGGGGCA    4821
HEINZ       ATGGTGTTCAGATATAGTTGATATCAAGCTGGATTATAAGCTCATCTATAGCCAGGGGCA    4821
LA2093      ATGGTGTTTAGATATAGTTGATATCAAGCTGGATTATAAGCTCATCTATAGCCAGG--CA    4837
            ******** ***********************************************  **

E42         AGCTCCTTTTGTTGAATTATTTAGCTGAATCACATCCTAAATAGAAATAATTATTTTTCC    4881
HEINZ       AGCTCCTTTTGTTGAATTATTTAGCTGAATCACATCCTAAATAGAAATAATTATTTTTCC    4881
LA2093      AGCTCCTTTAGTTGAATTACTTAGCTGAATCACATCCTAAATAGAAATAATTATTTTTCC    4897
            ********* ********* ****************************************

E42         ATCCAATCAATTATGTTGGTTATATAGAACCCTGAAAGTGGAGTGTAAATACCCTTTTAT    4941
HEINZ       ATCCAATCAATTATGTTGGTTATATAGAACCCTGAAAGTGGAGTGTAAATACCCTTTTAT    4941
LA2093      ATCCAATCAATT------------------------------------------------    4909
            ************

E42         AGTTCTGGAGAAAAACACATATGATGTTTTGTTAACACTAAATGAGCCAGGATAAGCTCC    5001
HEINZ       AGTTCTGGAGAAAAACACATATGATGTTTTGTTAACACTAAATGAGCCAGGATAAGCTCC    5001
LA2093      -----------------------------ATTAAACACTAAATGAGCCAGGATAAGCTCC    4940
                                           * ***************************

E42         CTGAGCTCATGTCTTTTCAAAGTTGAGCTACAAAGTGTGCTCGATTCAGCTAGGATGTGA    5061
HEINZ       CTGAGCTCATGTCTTTTCAAAGTTGAGCTACAAAGTGTGCTCGACACAGCTAGGATGTGA    5061
LA2093      CTGAGCTCATGTCTTTTCAAAGTTGAGCTACAAAGTGTGCTCGATTCAGCTAGGATGTGA    5000
            ********************************************  **************

E42         ACTTCCTATGCAAAAACATGATTGTTTTTAGCTCTAAAAGTGGATATGTACGTTAACCTA    5121
HEINZ       ACTTCCTATGCAAAAACATGATTGTTTTTAGCTCTAAAAGTGGATATGTACGTTAACCTA    5121
LA2093      ACTTCCTTTGCAAAAACATGATTGTTTTTAGCTCTAAAAGTGGATATGTACGTTAACCTA    5060
            ******* ****************************************************
```

```
E42         TTGACTTCTTGAATTTTTAACTACCAACTATTTTCTTGTTGGTTAAACTCCAGTGGAATA    5181
HEINZ       TTGACTTCTTGAATTTTTAACTACCAACTATTTTCTTGTTGGTTAAACTCCAGTGGAATA    5181
LA2093      T-AACTTCTTGAATTTTTAACTACCAACTATTTTCTTGTTGGTTAAACTCCAGTGGAATA    5119
            *  *********************************************************

E42         TGATACACAATACAAAACAACACAAGAGACCTCTTGGTAAATCATGCAGAAGAAATAGTC    5241
HEINZ       TGATACACAATACAAAACAACACAAGAGACCTCTTGGTAAATCATGCAGAAGAAATAGTC    5241
LA2093      TGATACACAATACAAAACAACACAAGAGACCTCTTGGTAAATCATGCAGTAGAAATAGTC    5179
            ************************************************* **********

E42         CATAAACTTAAGTTATATGCAGA-ACATACAACTCATCATCATAATAATTAAGTAACACT    5300
HEINZ       CATAAACTTAAGTTATATGCAGA-ACATACAACTCATCATCATAATAATTAAGTAACACT    5300
LA2093      CATAAACTTAAGTTATATGCAGAAACATACAACTCATCATCATAATAATTAAGTAACACT    5239
            *********************** ************************************

E42         GACAACTCTTTACAACAATAAAGTGCCCTGTACCTGAGAACAAATTGAAGTGCATGCACA    5360
HEINZ       GACAACTCTTTACAACAATAAAGTGCCCTGTACCTGAGAACAAATTGAAGTGCATGCACA    5360
LA2093      GACGACATATTACAACGATAAAGTGTCCTGTACCTGAGAACAAATTGAAGTGCATGCACA    5299
            *** **   ******* ******** **********************************

E42         TATTCAGATAAATTCTTGAGAAATGAACATTTTACAAGAAAAACCTAGCATGCTTGAAAA    5420
HEINZ       TATTCAGATAAATTCTTGAGAAATGAACATTTTACAAGAAAAACCTAGCATGCTTGAAAA    5420
LA2093      TATTCAGATAAATTCTTGAGAAATGAACATTTTACAAGGAAAACCTAGCATGCTTGAAAA    5359
            ************************************** *********************

E42         TGGTCATGTGTGGACTAAATGGTTCATTAGATCCTTTTAAGATAAAATGACTGTCACTAA    5480
HEINZ       TGGTCATGTGTGGACTAAATGGTTCATTAGATCCTTTTAAGATAAAATGACTGTCACTAA    5480
LA2093      TGGTCATGCGTGGACTAAATGGTTCATTAGATCCTTTTAAGATAAAATGACTGTCACTAA    5419
            ******** ***************************************************

E42         CATGAAAACAGCAGAGCTGATCAAAGCAGAATGTTGTGCTAAACTCCAATGGATTGCAAA    5540
HEINZ       CATGAAAACAGCAGAGCTGATCAAAGCAGAATGTTGTGCTAAACTCCAATGGATTGCAAA    5540
LA2093      CATGAAAATGGCAGAGCTGATCAAAGCAGAATGTCGTGCTAAACTCCAATGGATTGCAAA    5479
            ********  ************************ *************************

E42         TGAAGAAGAAGCAAAAATAAAATATTGGCCATAATTCGATTTTGATCCTGAAAACACCGA    5600
HEINZ       TGAAGAAGAAGCAAAAATAAAATATTGGCCATAATTCGATTTTGATCCTGAAAACACCGA    5600
LA2093      TGAAGAAGAAGCAAAAATAAAATATTGGCCATAATTCGATTTTGATCCTGAAAACACCGA    5539
            ************************************************************

E42         TTTGATAATTGAACATCAAATCATATCTAAGCAGGAGTAAGAAAAGAACCAATGATGCAC    5660
HEINZ       TTTGATAATTGAACATCAAATCATATCTAAGCAGGAGTAAGAAAAGAACCAATGATGCAC    5660
LA2093      TTTGATAATTGAACATCAAATCATATCTAAGCAGGAGTAAGAAAAGAACCAATGATGCAC    5599
            ************************************************************

E42         AAATGAACATGGCATATAATTAGCAGAAGACTT-TATGTTGATCTGAAGCATACCTTTGG    5719
HEINZ       AAATGAACATGGCATATAATTAGCAGAAGACTT-TATGTTGATCTGAAGCATACCTTTGG    5719
LA2093      AAATGAACATGGCATATAATTAGCAGAAGACTTTTGAGTTGATCTGAAGCATACCTTTGG    5659
            ********************************* *  ***********************

E42         TTGTTGTCTAATAAACTTATCAGTAGCCTCTAAAAGGCGGGTATTATCTACACCCGCCTT    5779
HEINZ       TTGTTGTCTAATAAACTTATCAGTAGCCTCTAAAAGGCGGGTATTATCTACACCCGTCTT    5779
LA2093      TTGTTGCCTAATAAACTTATCAGTAGCCTCTAAAAGGCGGGTATTATCTACACCTGCCTT    5719
            ****** ******************************************** * ***

E42         GGAGAATATGCGACGAGCCAGCCCATTCTTTTGCTCCAACAGTGCCTTCATCAAGTGCTC    5839
HEINZ       GGAGAATATGCGACGAGCCAGCCCATTCTTTTGCTCCAACAGTGCCTTCATCAAGTGCTC    5839
LA2093      GGAGAATATGCGACGAGCCAGCCCATTCTTTTGCTCCAACAGTGCCTTCATCAAGTGCTC    5779
            ************************************************************

E42         AGTCTCTACTATTTGATGCTTGTTCTCTTTAGCTATTTCTGGCGATGCAACAATTGCTTG    5899
HEINZ       AGTCTCTACTATTTGATGCTTGTTCTCTTTAGCTATTTCTGGCGATGCAACAATTGCTTG    5899
LA2093      AGTCTCTACTATTTGATGCTTGTTCTCTTTAGCTATTTCTGGCGATGCAACAATTGCTTG    5839
            ************************************************************

E42         CCATGCCATTTCTGTAAAATCTTGCTGAGTGATCTGCCATTACAAAACCAATTAATTGGA    5959
HEINZ       CCATGCCATTTCTGTAAAATCTTGCTGAGTGATCTGCCATTACAAAACCAATTAATTGGA    5959
LA2093      CCATGCCATTTCTGTAAAATCTTGCTGAGTTATCTGCCATTACAAAACCAATTAATTGGA    5899
            ****************************** *****************************

E42         TCGAGTGAAATATATCAGTCAAAACGGTTATATAAATCATCCATAACCACCTCACTCTAC    6019
```

```
HEINZ       TCGAGTGAAATATATCAGTCAAAACGGTTATATAAATCATCCATAACCACCTCACTCTAC    6019
LA2093      TCGAGTGAAATATATCAGTCAAAACGGTTATATAAATCATCCATAACCACCCCACTCTAC    5959
            *************************************************** ********

E42         TTGGACTGAAATATATCAGTCAGAACGATTATATAAATCATCCATATCCATTCCACTCTA    6079
HEINZ       TTGGACTGAAATATATCAGTCAGAACGATTATATAAATCATCCATATCCATTCCACTCTA    6079
LA2093      TTGGACTGAAATATATCAGTCAGAACGATTATATAAATCATCCATATCCACTCCACTCTA    6019
            ************************************************** *********

E42         CTTAGATATATTTCATTCCAAGTTGTATAATTTTCCCCTTAAGGTAATTAAGGGTTCTCC    6139
HEINZ       CTTAGATATATTTCATTCCAAGTTGTATAATTTTCCCCTTAAGGTAATTAAGGGTTCTCC    6139
LA2093      CTTAGATATATTTCATTCCAAGATGTATAATTTTCCCCTTAAGGTAATTAAGGGTTCTCC    6079
            ********************** *************************************

E42         CTCCATGGGTAAATAGTTGAGTCTCAACTAACTGGTATTGCTATCTCAACACTTTGCTTA    6199
HEINZ       CTCCATGGGTAAATAGTTGAGTCTCAACTAACTGGTATTGCTATCTCAACACTTTGCTTA    6199
LA2093      CTACATGGATAACTAGTTTAGTCTCAACTAACTGGTATTGCTATCTCAACACTTTGCTTA    6139
            ** ***** *** ***** *****************************************

E42         CAGATCATAGATTACAAGAGATATTAGGGAACGACTCTATATGCCTAACCATTCGATCAC    6259
HEINZ       CAGATCATTGATTCCAAGAGATATTAGGGAACGACTCTATATGCCTAACCATTCGATCAC    6259
LA2093      CAGATCATTGATTCCAAGAGATATTAGGGAACGACTCTATATGCCTAACCATTCGATCAC    6199
            ******** **** **********************************************

E42         ATTTGCCAAACAAGTATCTCACTCTGCAAGCAAATAGACATGCCATAACACCCAAACGGA    6319
HEINZ       ATTTGCCAAACAAGTATCTCACTCTGCAAGCAAATAGACATGCCATAACACCCAAACGGA    6319
LA2093      ATTTGCCAAACAAGTATCTCACTCTGCAAGCAAATAGACATGCCATAACACCCAAACGGA    6259
            ************************************************************

E42         AAAATGTGCAAGCACAAAGAATTCAATGCATTTTGATTGATTCATTCAATGAGAGTGTGA    6379
HEINZ       AAAATGTGCAAGCACAAAGAATTCAATGCATTTTGATTGATTCATTCAATGAGAGTGTGA    6379
LA2093      AAAATGTGCAAGCACAAAGAATTCAATGCATTTTGCTTGATTCATTCAATGAGAGTGTGA    6319
            *********************************** ************************

E42         CTTATCAACTGCTTGAGTACCACTTCAAATCTTCCTATTATGGATATCAATCAAGTGACT    6439
HEINZ       CTTATCAACTGCTTGAGTACCACTTCAAATCTTCCTATTATGGATATCAATCAAGTGACT    6439
LA2093      CTTATCAACTGCTTGAGTACCACTTCAAATCTTCCTATTATGGATATCAATCAAGTGACT    6379
            ************************************************************

E42         AATTGGCACCACTTCCCTCAATAAATTGATTAGATAAAGTGGCACTCAATTCAGAACCAA    6499
HEINZ       AATTGGCACCACTTCCCTCAATAAATTGATTAGATAAAGTGGCACTCAATTCAGAACCAA    6499
LA2093      AATTGGCACCACTTCCCTCAATAAATTGATTAGATAAAGTGGCACTCAATTCAGAACCAA    6439
            ************************************************************

E42         TAAATTGATCTGATAAAGCGGTGAGCATCTAAATACCATTTTGCACACTTCCACTATAGA    6559
HEINZ       TAAATTGATCTGATAAAGCGGTGAGCATCTAAATACCATTTTGCACACTTCCACTATAGA    6559
LA2093      TAAATTGATCTGATAAAGCGGTGAGCATCTAAATACCATTTTGCACACTTCCACTATAGA    6499
            ************************************************************

E42         AAGCAATTGCATTTACAATAAAGTAAAATCCATAAGTAAATGAATAAGTAGTATTACCCT    6619
HEINZ       AAGCAATTGCATTTACAATAAAGTAAAATCCATAAGTAAATGAATAAGTAGTATTACCCT    6619
LA2093      AAGCAATTGCATTTACAATAAAGTAAAATCCATAAGTAAATGAATAAGTAGTATTACCCT    6559
            ************************************************************

E42         TCCATTTGAAGCATCACAACGTACGGTAAGCCTTGAGCCCTGAGAAAGTTTCTCAGTTTT    6679
HEINZ       TCCATTTGAAGCATCACAACGTACGGTAAGCCTTGAGCCCTGAGAAAGTTTCTCAGTTTT    6679
LA2093      TCCATTTGAAGCATCACAACGTACGGTAAGCCTTGAGCCCTGAGAAAGTTTCTCAGTTTT    6619
            ************************************************************

E42         CCTACTGAAGAACACATCCTTCCTCTTCAGTTTCAACGAACTACACTTGCCAAGAACTCG    6739
HEINZ       CCTACTGAAGAACACATCCTTCCTCTTCAGTTTCAACGAACTACACTTGCCAAGAACTCG    6739
LA2093      CCTACTGAAGAACACATCCTTCCTCTTCAGTTTCAACGAACTACACTTGCCAAGAACTCG    6679
            ************************************************************

E42         CGATTTGCCTGAGAAGTTCAGGTACGGCACTGAATGGGATGAAAATAAAGCAACTCTGTT    6799
HEINZ       CGATTTGCCTGAGAAGTTCAGGTACGGCGCTGAATGGGATGAAAATAAAGCAACTCTGTT    6799
LA2093      CGATTTGCCTGAGAAGTTCAGGTACGGCGCTGAATGGGATGAAAATAAAGCAACTCTGTT    6739
            **************************** *******************************

E42         TGAAGAATTGGAAGAAGAAGAAGGAACGCAAAACTGTACGCCGGAAAATGACGTCACGGT    6859
HEINZ       TGAAGAATTGGAAGAAGAAGAAGGAACGCAAAACTGTACGCCGGAAAATGACGTCACGGT    6859
LA2093      TGAAGAATTGGAAGAAGAAGAAGGAACGCAAAACTGTACGCCGGAAAATGACGTCACGGT    6799
```

```
            ************************************************************

E42         AGACATACCGGTGAACCAAATGTACTATTCTGTTATCTCAACGTTATTGAAGAAGGAAGA    6919
HEINZ       AGACATACCGGTGAACCAAATGTACTATTCTGTTATCTCAACGTTATTGAAGAAGGAAGA    6919
LA2093      AGACATACCGGTGAACCAAATGTACTATTCTGTTATCTCAACGTTATTGAAGAAGGAAGA    6859
            ************************************************************

E42         AAAACGACAAAGTTTCAGCGGATAGGAGAGAGTGTTTTGCCGGAAAAGTTTGGGGTTTGC    6979
HEINZ       AAAACGACAAAGTTTCAGCGGATAGGAGAGAGTGTTTTGCCGGAAAAGTTTGGGGTTTGC    6979
LA2093      AAAACGACAAAGTTTCAGCGGATAGGAGAGAGTGTTTTGCCGGAAAAGTTTGGGGTTTGC    6919
            ************************************************************

E42         GTGAGAATATAAGGAACCGGTGGACTCTTCTCGTAACTTCCCGAACGTGAGAGCGCGTGT    7039
HEINZ       GTGAGAATATAAGGAACCGGTGGACTCTTCTCGTAACTTCCCGAACGTGAGAGCGCGTGT    7039
LA2093      GTGAGAATATAAGGAACCGGTGGACTCTTCTCGTAACTTCCCGAACGTGAGAGCGCGTGT    6979
            ************************************************************

E42         GAAAATTTTTACTATGATATCTTTCATTTTTTAACGTGTATTTTATATATTTTTTTTATG    7099
HEINZ       GAAAATTTTTACTATGATATCTTTCATTTTTTAACGTGTATTTTATATATTTTTTTTATG    7099
LA2093      GAAAATTTTTACTATGATATCTTTCATTTTTTAACGTGTATTTTATATATTTTTTTTATG    7039
            ************************************************************

E42         AAAATTAATATATA    7113
HEINZ       CAAATTAATATATA    7113
LA2093      CAAATTAATATATA    7053
             *************
```

Solyc02g093600

```
E42          CACGTTAAGACGATCCAAGAAGTTTCAAGACCCATTTGAACTGTCTTTTTTCACCAACTA    60
HEINZ        CACGTTAAGACGATCCAAGAAGTTTCAAGACCCATTTGAACTGTCTTTTTTCACCAACTA    60
LA2093       CACGTTAAGACGATCCAAGAAGTTTCAAGACCCATTTGAACTGTCTTTTTTCACCAACTA    60
             ************************************************************

E42          TATATACTTGTGCAAAGTTTCAATTTATTCCACAGAAGACAACAAGGAAATACAATTAAT    120
HEINZ        TATATACTTGTGCAAAGTTTCAATTTATTCCACAGAAGACAACAAGGAAATACAATTAAT    120
LA2093       TATATACTTGTGCAAAGTTTCAATTTATTCCACAGAAGACAACAAGGAAATACAATTAAT    120
             ************************************************************

E42          TAGCTGATTTAAGTGGGGAAAACAAAGATGTGCAGCTTATTGAGGAGCAGTGATCCAATT    180
HEINZ        TAGCTGATTTAAGTGGGGAAAACAAAGATGTGCAGCTTATTGAGGAGCAGTGATCCAATT    180
LA2093       TAGCTGATTTAAGTGGGGAAAACAAAGATGTGCAGCTTATTGAGGAGCAGTGATCCAATT    180
             ************************************************************

E42          GTTGGGATGATGAATATGTGTCCTGTTTTGAGTACTCCAATTGACTGGAAAGAAACACCT    240
HEINZ        GTTGGGATGATGAATATGTGTCCTGTTTTGAGTACTCCAATTGACTGGAAAGAAACATCT    240
LA2093       GTTGGGATGATGAATATGTGTCCTGTTTTGAGTACTCCAATTGACTGGAAAGAAACACCT    240
             ********************************************************* **

E42          CAAGCTCACTGCTTCTTCGTCGATCTTCCAGGTATTAATTTAAACGATTTAACAATTTAA    300
HEINZ        CAAGCTCACTGCTTCTTCGTCGATCTTCCAGGTATTAATTTAAACGATTTAACAATTTAA    300
LA2093       CAAGCTCACTGCTTCTTCGTCGATCTTCCAGGTATTAATTTAAACGATTTAACAATTTAA    300
             ************************************************************

E42          TAGGGATAATACATATATTGGATTTTAAACTGGTTTTATATTTTAACTTTAATCTTCGAT    360
HEINZ        TAGGGATAATACATATATTGGATTTTAAACTGGTTTTATATTTTAACTTTAATCTTCGAT    360
LA2093       TAGGGATAATACATATATTGGATTTTAAACTGGTTTTATATTTTAACTTTAATCTTCGAT    360
             ************************************************************

E42          TTTCATATTTTAATTATTTAATTTTTAAATAAATAAACACATGAATCCTATATCCTACAA    420
HEINZ        TTTCATATTTTAATTATTTAATTTTTAAATAAATAAACACATGAATCCTATATGACACAA    420
LA2093       TTTCATATTTTAATTATTTAATTTTTAAATAAATAAACACATGAATCCTATATGACACAA    420
             *****************************************************   ****

E42          TACATGTAGGATAAAAATTGACATGTAGTATGATATGTAGGACACGTGTATTTATTTATT    480
HEINZ        TACATGTAGGATAAAAATTGACATGTAGTATGATATGTAGGACACGTGTATTTATTTATT    480
LA2093       TACATGTAGGATAAAAATTGACATATAGTATGATATGTAGGACACGTGTATTTATTTATT    480
             ************************ ***********************************

E42          AAAAATGTTAGAAAGAGTATATATTGTGTACATACAGGGCTCAGCAAAGAGGATGTGAAA    540
HEINZ        AAAAATGTTAGAAAGAGTATATATTGTGTACATACAGGGCTCAGCAAAGAGGATGTGAAA    540
LA2093       AAAAATGTTAGAAAGAGTATATATTGTGTACATACAGGGCTCAGCAAAGAGGATGTGAAA    540
             ************************************************************

E42          GTGGAAGTGGACAATGGAAGAGTAGTGA--------GGTAAGTGGAAAGCAGAAGAAGAA    592
HEINZ        GTGGAAGTGGACAATGGAAGAGTAGTGAAAATTAGTGGTAAGTGGAAAGCAGAAGAAGAA    600
LA2093       GTGGAAGTGGACAATGGAAGAGTAGTGAAAATTAGTGGTAAGTGGAAAGCAGAAGAAGAA    600
             ****************************        ************************

E42          ATTGGAGATGAAAATGAGAAGAAGAATTTATGGCATAGAGTGGAACGTAATAGGGGTGAT    652
HEINZ        ATTGGAGATGAAAATGAGAAGAAGAATTTATGGCATAGAGTGGAACGTAATAGGGGTGAT    660
LA2093       ATTGGAGATGAAAATGAGAAGAAGAATTTATGGCATAGAGTGGAACGTAATAGGGGTGAT    660
             ************************************************************

E42          TTCTGCAGGAAGTTTAGGCTTCCAAAAAAATATAATGGCAGATCGACTCGAGGCATCCATG    712
HEINZ        TTCTGCAGGAAGTTTAGGCTTCCAAAAAAATATAATGGCAGATCGACTCGAGGCATCCATG    720
LA2093       TTCTGCAGGAAGTTTAGGCTTCCAAAAAAATATAATGGCAGATCGACTCGAGGCATCCATG    720
             ************************************************************

E42          GAAAATGGTGTCCTTGTTCTCACCGTACCTAAACAAGAACTCAAAAAACCCTTCTCCAAA    772
HEINZ        GAAAATGGTGTCCTTGTTCTCACCGTACCTAAACAACAACTCAAAAAACCCTTCTCCAAA    780
LA2093       GAAAATGGTGTCCTTGTTCTCACCGTACCTAAACAACAACTCAAAAAACCCTTCTCCAAA    780
             ************************************ ***********************

E42          GTAATTGAAATTGAAGAAAAATAAACATGAACTTTAAGTTATTATTATTGTGGATTGCAA    832
HEINZ        GTAATTGAAATTGAAGAAAAATAAACATGAACTTTAAGTTATTATTATTGTGGATTGCAA    840
LA2093       GTAATTGAAATTGAAGAAAAATAAACATGAACTTTAAGTTATTATTATTGTGGATTGCAA    840
             ************************************************************
```

```
E42         TTCGAATGAATAAATAAATCGTCTGTTGATTCCATGTTTTTATATGTTTTCTATGATTTT    892
HEINZ       TTCGAATGAATAAATAAATCGTCTGTTGATTCCATGTTTTTATATGTTTTCTATGATTTT    900
LA2093      TTCGAATGAATAAATAAATCGTCTGTTGATTCCATGTTTTTATATGTTTTCTATGATTTT    900
            ************************************************************

E42         CCCTGTGATTAATAAATGAATATTAGCATCTGAATTTGTTATAT    936
HEINZ       CCCTGTGATTAATAAATGAATATTAGCATCTGAATTTGTTATAT    944
LA2093      CCCTGTGATTAATAAATGAATATTAGCATCTGAATTTGTTATAT    944
            ********************************************
```

Solyc03g122230

```
LA2093      TGGACATTTTTCGTGTTATGTGTCTTGTTTGCATCGTCTTGTTGATGATTTACCCCGTGA    60
E42         TGGACATTTTTCGTGTTATGTGTCTTGTTTGCATCGTCTTGTTGATGATTTACCCCGTGA    60
HEINZ       TGGACATTTTTCGTGTTATGTGTCTTGTTTGCATCGTCTTGTTGATGATTTACCCCGTGA    60
            ************************************************************

LA2093      AAATAATATCAGTTTCTGATACTGATGCTTTGTTGAAGCTGAAACAATCATTTACAAATG    120
E42         AAATAATATCAGTTTCTGATACTGATGCTTTGTTGAAGCTGAAACAATCATTTACAAATG    120
HEINZ       AAATAATATCAGTTTCTGATACTGATGCTTTGTTGAAGCTGAAACAATCATTTACAAATG    120
            ************************************************************

LA2093      CAGCTTCATTGGAATCATGGAAACCTGGAACGGATCCGTGTGATAAAAATATACGTTGGT    180
E42         CAGCTTCATTGGAATCATGGAAACCTGGAACGGATCCGTGTGATAAAAATATACGTTGGT    180
HEINZ       CAGCTTCATTGGAATCATGGAAACCTGGAACGGATCCGTGTGATAAAAATATACGTTGGT    180
            ************************************************************

LA2093      TGGGTGTTTTTTGTCAGAAAAAAAATAGTTACTGGACTTCTTCTTGCAGAAACGAATTTAT    240
E42         TGGGTGTTTTTTGTCAGAAAAAAAATAGTTACTGGACTTCTTCTTGCAGAAACGAATTTAT    240
HEINZ       TGGGTGTTTTTTGTCAGAAAAAAAATAGTTACTGGACTTCTTCTTGCAGAAACGAATTTAT    240
            ************************************************************

LA2093      CTGGAATTATAGACGTTGAGGCATTATCACAAATGCCTGGTCTGCGTACACTTAGTTTTC    300
E42         CTGGAATTATAGACGTTGAGGCATTATCACAAATGCCTGGTCTGCGTACACTTAGTTTTC    300
HEINZ       CTGGAATTATAGACGTTGAGGCATTATCACAAATGCCTGGTCTGCGTACACTTAGTTTTC    300
            ************************************************************

LA2093      AGAGCAATTTATTTTCTGGACCAATGCCGGAATTCAATCGAATGGGTGCTTTAAAAGGTC    360
E42         AGAGCAATTTATTTTCTGGACCAATGCCGGAATTCAATCGAATGGGTGCTTTAAAAGGTC    360
HEINZ       AGAGCAATTTATTTTCTGGACCAATGCCGGAATTCAATCGAATGGGTGCTTTAAAAGGTC    360
            ************************************************************

LA2093      TTTATTTGTCGGAAAATCAATTTTCTGGTGAAATTCCTTCAAATTACTTCGCAAAAATGT    420
E42         TTTATTTGTCGGAAAATCAATTTTCTGGTGAAATTCCTTCAAATTACTTCGCAAAAATGT    420
HEINZ       TTTATTTGTCGGAAAATCAATTTTCTGGTGAAATTCCTTCAAATTACTTCGCAAAAATGT    420
            ************************************************************

LA2093      TGTCCTTGAAGAAGTTATGGCTCTCTGACAACAAATTTTCCGGTGAAATTCCGGCGTCAT    480
E42         TGTCCTTGAAGAAGTTATGGCTCTCTGACAACAAATTTTCCGGTGAAATTCCGGCGTCAT    480
HEINZ       TGTCCTTGAAGAAGTTATGGCTCTCTGACAACAAATTTTCCGGTGAAATTCCGGCGTCAT    480
            ************************************************************

LA2093      TAATGGAGCTGCAATATCTCATTGAATTGCACCTGGAGAACAATCAGTTCACAGGTCCAA    540
E42         TAATGGAGCTGCAATATCTCATTGAATTGCACCTGGAGAACAATCAGTTCACAGGTCCAA    540
HEINZ       TAATGGAGCTGCAATATCTCATTGAATTGCACCTGGAGAACAATCAGTTCACAGGTCCAA    540
            ************************************************************

LA2093      TACCCCCAATGTCGAAGCAGTCAACGTTAGAATCAATCGATTTTTCTAATAATAACTTGA    600
E42         TACCCCCAATGTCGAAGCAGTCAACGTTAGAATCAATCGATTTTTCTAATAATAACTTGA    600
HEINZ       TACCCCCAATGTCGAAGCAGTCAACGTTAGAATCAATCGATTTTTCTAATAATAACTTGA    600
            ************************************************************

LA2093      AAGGGGAAATCCCTGTTTCATTATCAAGATTCAATGAGAGTTCGTTTAAGGGGAATTCCG    660
E42         AAGGGGAAATCCCTGTTTCATTATCAAGATTCAATGAGAGTTCGTTTAAGGGGAATTCCG    660
HEINZ       AAGGGGAAATCCCTGTTTCATTATCAAGATTCAATGAGAGTTCGTTTAAGGGGAATTCCG    660
            ************************************************************

LA2093      AGCTTTGTGGGGAGAAATTAGGCAAACCTTGTAATCAAGCCACAAATAGCGACAACACTA    720
E42         AGCTTTGTGGGGAGAAATTAGGCAAACCTTGTAATCAAGCCACAAATAGCGACAACACTA    720
HEINZ       AGCTTTGTGGGGAGAAATTAGGCAAACCTTGTGATCAAGCCACAAATAGCGACAACACTA    720
            ******************************** ***************************

LA2093      ATAATAATGGCACTCAACAAAGTCCGGATAGTAATTCTAATAAAAGCATCTCAATGTGGA    780
E42         ATAATAATGGCTCTCAACAAAGTCCGGATAGTAATTCTAATAAAAGCATCTCAATGTGGA    780
HEINZ       ATAATAATGGCACTCAACAAAGTCCGGATAGTAATTCTAATAAAAGCATCTCAATGTGGA    780
            *********** ************************************************

LA2093      TTGTGATATCATTAGTTGTTCTGTTATTGATCGTGATCATAGTAATATACCTCATATGTC    840
E42         TTGTGATATCATTAGTTGTTCTGTTATTGATCGTGATCATAGTAATATACCTCATATGTC    840
HEINZ       TTGTGATATCATTAGTTGTTCTGTTATTGATCGTGATCATAGTAATATACCTCATATGTC    840
            ************************************************************
```

```
LA2093      GCTACCAACAAAGTCGTCGTGCATCCATTGAAAGTTTTGATGAGCCATCACTTGGGAGGC    900
E42         GCTACCAACAAAGTCGTCGTGCATCCATTGAAAGTTTTGATGAGCCATCACTTGGGAGGC    900
HEINZ       GCTACCAACAAAGTCGTCGTGCATCCATTGAAAGTTTTGATGAGCCATCACTTGGGAGGC    900
            ************************************************************

LA2093      GAATTTCAAGTGATAGCAAGAAGTCATTTGAACTAAGTAGACGTGGAAGTTCGATTAGAA    960
E42         GAATTTCAAGTGATAGCAAGAAGTCATTTGAACTAAGTAGACGTGGAAGTTCGATTAGAA    960
HEINZ       GAATTTCAAGTGATAGCAAGAAGTCATTTGAACTAAGTAGACGTGGAAGTTCGATTAGAA    960
            ************************************************************

LA2093      AAGGGTCGATAATGGGAAAACGTGTAGGAGATTTGACAATGGTGAATGATGATAAAGGTG    1020
E42         AAGGGTCGATAATGGGAAAACGTGTAGGAGATTTGACAATGGTGAATGATGATAAAGGTG    1020
HEINZ       AAGGGTCGATAATGGGAAAACGTGTAGGAGATTTGACAATGGTGAATGATGATAAAGGTG    1020
            ************************************************************

LA2093      AGTTTGGTTTAGCAGATTTGATGAAAGCTGCTGCAGAGGTGTTAGGGAATGGGCCATTGG    1080
E42         AGTTTGGTTTAGCAGATTTGATGAAAGCTGCTGCAGAGGTGTTAGGGAATGGGCCATTGG    1080
HEINZ       AGTTTGGTTTAGCAGATTTGATGAAAGCTGCTGCAGAGGTGTTAGGGAATGGGCCATTGG    1080
            ************************************************************

LA2093      GTTCTTCTTATAAGGCTATGATGTCAAATGGATTGACAGTTGTGGTAAAGAGGATCAAAG    1140
E42         GTTCTTCTTATAAGGCTATGATGTCAAATGGATTGACAGTTGTGGTAAAGAGGATCAAAG    1140
HEINZ       GTTCTTCTTATAAGGCTATGATGTCAAATGGATTGACAGTTGTGGTAAAGAGGATCAAAG    1140
            ************************************************************

LA2093      AAATGAACAAGATCGGAAAAGAGGGTTTCGATGCAGAGGTTAAACGTTTAGGAAGTTTGA    1200
E42         AAATGAACAAGATCGGAAAAGAGGGTTTCGATGCAGAGGTTAAACGTTTAGGAAGTTTGA    1200
HEINZ       AAATGAACAAGATCGGAAAAGAGGGTTTCGATGCAGAGGTTAAACGTTTAGGAAGTTTGA    1200
            ************************************************************

LA2093      GACATAAAAATATTCTAACACTTTTGGCTTACCATCATCGCAAGGAAGAAAAGTTGTTTG    1260
E42         GACATAAAAATATTCTAACACTTTTGGCTTACCATCATCGCAAGGAAGAAAAGTTGTTTG    1260
HEINZ       GACATAAAAATATTCTAACACTTTTGGCTTACCATCATCGCAAGGAAGAAAAGTTGTTTG    1260
            ************************************************************

LA2093      TATATGAGTACATTCCTAAAGGAAGCTTGCTGTACGTATTGCACGGTAAAGTACCTACCT    1320
E42         TATATGAGTACATTCCTAAAGGAAGTTTGCTGTACGTATTGCACGGTAAAGTACCTACCT    1320
HEINZ       TATATGAGTACATTCCTAAAGGAAGTTTGCTGTACGTATTGCACGGTAAAGTACCTACCT    1320
            ************************* **********************************

LA2093      CTAATTTTATCAAGTCATGCTAGAAATGCATGGTAAGACTGCGTACAATAAGCTCATTTT    1380
E42         CTAATTTTATCAAGTCATGCTAGAAATGCATGGTAAGACTGCGTACAATAAGCTCATTTT    1380
HEINZ       CTAATTTTATCAAGTCATGCTAGAAATGCATGGTAAGACTGCGTACAATAAGCTCATTTT    1380
            ************************************************************

LA2093      TGTTAATTTTGTGCATGTGCTTAGAATAGGAGGCCAAAAATTTCTACAAGTCGTATGTTT    1440
E42         TGTTAATTTTGTGCATGTGCTTAGAATAGGAGGCCAAAAACTTCTACAAGTCGTGTGTTT    1440
HEINZ       TGTTAATTTTGTGCATGTGCTTAGAATAGGAGGCCAAAAACTTCTACAAGTCGTGTGTTT    1440
            **************************************** ************* *****

LA2093      ACATTGTATTAGTAGTTTAGTCGTCAAGTGTTTCATATTAGTTGAGGAACGGAAAAGTAG    1500
E42         ACATTGTATTAGTAGTTTAGTCGTCAAGTGTTTCATATTAGTTGAGGAACGG-AAAGTAG    1499
HEINZ       ACATTGTATTAGTAGTTTAGTCGTCAAGTGTTTCATATTAGTTGAGGAACGG-AAAGTAG    1499
            **************************************************** *******

LA2093      TCTCCTAGTATGATCTTGGCAAACTATACGTCTATATCCATGAACTAGCTTTGAAGTTGA    1560
E42         TCTCCTAGTATGATCATGGCAAACTATACGTCTATATCCATGAACTAGCTTTGAAGTTGA    1559
HEINZ       TCTCCTAGTATGATCATGGCAAACTATACGTCTATATCCATGAACTAGCTTTGAAGTTGA    1559
            *************** ********************************************

LA2093      GTGAGGTCTAAAGGTCCGTTTGCGTCACAAGTTATCGAAGCCAGACTCATCTGTACTCTT    1620
E42         GTGAGGTCTAAAGGTCCGTTTGCTTCACAAGTTATCGAAGCCAGACTCATCTGTACTCTT    1619
HEINZ       GTGAGGTCTAAAGGTCCGTTTGCTTCACAAGTTATCGAAGCCAGACTCATCTGTACTCTT    1619
            *********************** ************************************

LA2093      GACATGTAGAGCCCCCATGTTATATTGGGATTATTTGCACCTTATGGTCTTTTCCAGGCC    1680
E42         GACATGTAGAGCCCCCATGTTATATTGGGATTATTTGCACCTTATGGTCTTTTCCAGGCC    1679
HEINZ       GACATGTAGAGCCCCCATGTTATATTGGGATTATTTGCACCTTATGGTCTTTTCCAGGCC    1679
            ************************************************************

LA2093      ACTTATTGAATTATGTTCCCGTTAGCCCGTGTGTTAAGGGGAATGTAACATAAATAGAGA    1740
```

```
E42             ACTTATTGAATTATGTTCCCGTTAGCCCGTGTGTTAAGGGGAATGTAACATAAATAGAGA    1739
HEINZ           ACTTATTGAATTATGTTCCCGTTAGCCCGTGTGTTAAGGGGAATGTAACATAAATAGAGA    1739
                ************************************************************

LA2093          CCTCAAGAAAGGTCTTTTCTACAGTTGTCCTGTTGGGAGTTAAGGGACAGATTTGAGCTA    1800
E42             CCTCAAGAAAGGTCTTTTCTACAGTTGTCCTGTTGGGAGTTAAGGGACAGATTTGAGCTA    1799
HEINZ           CCTCAAGAAAGGTCTTTTCTACAGTTGTCCTGTTGGGAGTTAAGGGACAGATTTGAGCTA    1799
                ************************************************************

LA2093          TGCTTAACTCATGAACTAGTTTTTGAGGTTTAATTAGGCCAAATACTCAATTTTCTTTAC    1860
E42             TGCTCAACTCATGAACTAGTTTTTGAGGTTTAATTAGGCCAAATACTCAATTTTCTTTAC    1859
HEINZ           TGCTCAACTCATGAACTAGTTTTTGAGGTTTAATTAGGCCAAATACTCAATTTTCTTTAC    1859
                **** *******************************************************

LA2093          GCCAATTAGTTTGTGTATGATTACTTTACTTATAAAGATTGCTGCTGCTTTATGAATGAT    1920
E42             GCCAACTAGTTTGTGTATGATTACTTTACTTATAAAGATTGCTGCTGCTTTATGAATGAT    1919
HEINZ           GCCAACTAGTTTGTGTATGATTACTTTACTTATAAAGATTGCTGCTGCTTTATGAATGAT    1919
                ***** ******************************************************

LA2093          CACAGGTGAAAGAGGGCTGCCCCATGCTGAGCTCACCTGGCCAGTGCGTCTAAAAATTAT    1980
E42             CACAGGTGAAAGAGGGCTGCCCCATGCTGAGCTCACCTGGCCAGTGCGTCTAAAAATTAT    1979
HEINZ           CACAGGTGAAAGAGGGCTGCCCCATGCTGAGCTCACCTGGCCAGTGCGTCTAAAAATTAT    1979
                ************************************************************

LA2093          ACAAGGAGTAGCTCAAGGACTAAATTATCTTCACACTGAACTTGCTTCTTCTGATTTGCC    2040
E42             ACAAGGAGTAGCTCAAGGACTAAATTATCTTCACACTGAACTTGCTTCTTCTGATTTGCC    2039
HEINZ           ACAAGGAGTAGCTCAAGGACTAAATTATCTTCACACTGAACTTGCTTCTTCTGATTTGCC    2039
                ************************************************************

LA2093          CCACGGCGACTTGAAATCTAGCAATATACTCATAAACACAAACCACGAGCCAATACTCAC    2100
E42             CCACGGCGACTTGAAATCTAGCAATATACTCATAAACACAAACCACGAGCCAATACTCAC    2099
HEINZ           CCACGGCGACTTGAAATCTAGCAATATACTCATAAACACAAACCACGAGCCAATACTCAC    2099
                ************************************************************

LA2093          TGGTTACGGATTCTGCACCTTAATGAACAACGCGCATGCAGTTCAAGCATTGATCGCGTT    2160
E42             TGGTTACGGATTCTGCACCTTAATGAACAACGCGCATGCAGTTCAAGCATTGATCGCGTT    2159
HEINZ           TGGTTACGGATTCTGCACCTTAATGAACAACGCGCATGCAGTTCAAGCATTGATCGCGTT    2159
                ************************************************************

LA2093          CAAGTCACCAGAAGCAGTACAAAATAACCAGGTTACACCCAAGTGCGACGTTTATTGCCT    2220
E42             CAAGTCACCAGAAGCAGTACAAAATAACCAGGTTACACCCAAGTGTGACGTTTATTGCCT    2219
HEINZ           CAAGTCACCAGAAGCAGTACAAAATAACCAGGTTACACCCAAGTGTGACGTTTATTGCCT    2219
                ********************************************* **************

LA2093          TGGAATTGTGATTCTTGAGATAATAACAGGGAAGTACCCGTCTATATATCTTAATGCCGG    2280
E42             TGGAATTGTGATTCTTGAGATAATAACAGGGAAGTACCCGTCTATATATCTTAATGCCGG    2279
HEINZ           TGGAATTGTGATTCTTGAGATAATAACAGGGAAGTACCCGTCTATATATCTTAATGCCGG    2279
                ************************************************************

LA2093          TAAAGGAGGGATAGACATAGCACAATGGGCGAAATCCGCGATTGCTGAGGGAAGAGAAGC    2340
E42             TAAAGGAGGGATAGACATAGCACAATGGGCGAAATCCGCGATTGCTGAGGGAAGAGAAGC    2339
HEINZ           TAAAGGAGGGATAGACATAGCACAATGGGCGAAATCCGCGATTGCTGAGGGAAGAGAAGC    2339
                ************************************************************

LA2093          TGAATTATGTGATCCAGATATAACAGCAAGCGCGAAAGATTCAATGAGTTCAATTAAGGA    2400
E42             TGAATTATGTGATCCAGATATAACAGCAAGCGCGAAAGATTCAATGAGTTCAATTAAGGA    2399
HEINZ           TGAATTATGTGATCCAGATATAACAGCAAGCGCGAAAGATTCAATGAGTTCAATTAAGGA    2399
                ************************************************************

LA2093          ACTAATTCACATTAGTGCAGCATGTGTAGAGAGTAATCCACAACAGAGGATAAGTATTAG    2460
E42             ACTAATTCACATTAGTGCAGCATGTGTAGAGAGTAATCCACAACAGAGGATAAGTATTAG    2459
HEINZ           ACTAATTCACATTAGTGCAGCATGTGTAGAGAGTAATCCACAACAGAGGATAAGTATTAG    2459
                ************************************************************

LA2093          AGAAGTCATTAGGAGGATTGAAGAAATACAACAACAAAACGGAGTAGGACAAACAACAAC    2520
E42             AGAAGTCATTAGGAGGATTGAAGAAATACAACAACAAAACGGAGTAGGACGAACAACAAC    2519
HEINZ           AGAAGTCATTAGGAGGATTGAAGAAATACAACAACAAAACGGAGTAGGACGAACAACAAC    2519
                ************************************************** *********

LA2093          GTCTCAGTCTCAAACGGGACAAGCAATTGAGATAATGCCGTCTCCAAGAGATGGAGATGC    2580
E42             GTCTCAGTCTCAAACGGGACAAGCAATTGAGATAATGCCGTCTCCAAGAGATGGAGATGC    2579
HEINZ           GTCTCAGTCTCAAACGGGACAAGCAATTGAGGTAATGCCGTCTCCAAGAGATGGAGATGC    2579
```

```
             ******************************* ****************************

LA2093       TGAAATACAAGTTTCAGAATCCCAACAAGGAATAACAAATCATTAG   2626
E42          TGAAATACAAGTTTCAGAATCCCAACAAGGAATAACAAATCATTAG   2625
HEINZ        TGAAATACAAGTTTCAGAATCCCAACAAGGAATAACAAATCATTAG   2625
             **********************************************
```

Solyc04g026280

```
LA2093      ACTTAAAAGACGATGATTGCAACAAAATAGCTTTACATAGGTTCCATCCAAAGAAGGTTT     60
E42         ACTTAAAAGACGATGATTGCAACAAAATAGCTTTACATAGGTTCCATCCAACGAAGGTTT     60
HEINZ       ACTTAAAAGACGATGATTGCAACAAAATAGCTTTACATAGGTTCCATCCAAAGAAGGTTT     60
            *************************************************** ********

LA2093      ACACGTTTTACTTCAATTCACATTTACATACGATTAAGTAAACCAAACAAAAACCAAATA     120
E42         ACACGTTTTACTTCAATTCACATTTACATACGATTAAGTAAACCAAACAAAAACCAAATA     120
HEINZ       ACACGTTTTACTTCAATTCACATTTACATACGATTAAGTAAACCAAACAAAAACCAAATA     120
            ************************************************************

LA2093      ATAATGAGTTTTTAGCGATTCATCAACTACAAAATTCTCTACAACTTCTACATATCTGTC     180
E42         ATAATGAGTTTTTAGCGATTCATCAACTACAAAATTCTCTACAACTTCTACATATCTGTC     180
HEINZ       ATAATGAGTTTTTAGCGATTCATCAACTACAAAATTCTCTACAACTTCTACATATCTGTC     180
            ************************************************************

LA2093      TCCACACATTGACAGCCTCAGTCCTTAGCGTTACTTCTTCATTCTCATGGATAAACAGTG     240
E42         TCCACAAATTGACAGCCTCAGTCCTTAGCGTTACTTCTTCATTCTCATGGATAAACAGTG     240
HEINZ       TCCACACATTGACAGCCTCAGTCCTTAGCGTTACTTCTTCATTCTCATGGATAAACAGTG     240
            ****** *****************************************************

LA2093      TTTTCGGGATATTTCTCCCTTGTGCTTATGCTGCCAACATTTCAGCTGCCATCTGCATCT     300
E42         TTTTCGGGATATTTCTCCCTTGTGCTTATGCTGCCAACATTTCAGCTGCCATCTGCATCT     300
HEINZ       TTTTCGGGATATTTCTCCCTTGTGCTTATGCTGCCAACATTTCAGCTGCCATCTGCATCT     300
            ************************************************************

LA2093      ATTCAGGATTTTGGCTGGCATGAGTTTCTTGTCCAGTAAAAACTTGACCATGCTCTCTTA     360
E42         ATTCAGGATTTTGGCTGGCATGAGTTTCTTGTCCAGTAAAAACTTGACCATGCTCTCTTA     360
HEINZ       ATTCAGGATTTTGGCTGGCATGAGTTTCTTGTCCAGTAAAAACTTGACCATGCTCTCTTA     360
            ************************************************************

LA2093      CATCTCGAAATATAGAGAGAAAGGAGTTTAGACCTTCAGAAGAAGCACCCCCTAATATCC     420
E42         CATCTCGAAATATAGAGAGAAAGGAGTTTAGACCTTCAGAAGAAGCACCCCCTAATATCC     420
HEINZ       CATCTCGAAATATAGAGAGAAAGGAGTTTAGACCTTCAGAAGAAGCACCCCCTAATATCC     420
            ************************************************************

LA2093      ACAGCAGCAAGGAGCTGAATGAATTCATAGACCCCGGGTTAGCTCCACCTTCAGACTGGC     480
E42         ACAGCAGCAAGGAGCTGAATGAATTCATAGACCCCGGGTTAGCTCCACCTTCAGACTGGC     480
HEINZ       ACAGCAGCAAGGAGCTGAATGAATTCATAGACCCCGGGTTAGCTCCACCTTCAGACTGGC     480
            ************************************************************

LA2093      GGGTATCCACTTGCCCTCCAAGTTGAAGAGAATTACCAAACTCATCTTGGTTTCCATTAC     540
E42         GGGTATCCACTTGCCCTCCAAGTTGAAGAGAATTACCAAACTCATCTTGGTTTCCATTAC     540
HEINZ       GGGTATCCACTTGCCCTCCAAGTTGAAGAGAATTACCAAACTCATCTTGGTTTCCATTAC     540
            ************************************************************

LA2093      CTTGTCCATCTGCAACTCCCGGAAATTCATTGGCACCATTTCTTGGCAATGACGGATCAA     600
E42         CTTGTCCATCTGCAACTCCCGGAAATTCATTGGCACCATTTCTTGGCAATGACCGATCAA     600
HEINZ       CTTGTCCATCTGCAACTCCCGGAAATTCATTGGCACCATTTCTTGGCAATGACGGATCAA     600
            ***************************************************** ******

LA2093      TTCTAGGCCCAGGACCAGGAGCCATATCAACATTAGTTGGAGCAGGGCTCCTTGCATTTA     660
E42         TTCTAGGCCCAGGACCAGGAGCCATATCAACATTAGTTGGAGCAGGGCTCCTTGCATTTA     660
HEINZ       TTCTAGGCCCAGGACCAGGAGCCATATCAACATTAGTTGGAGCAGGGCTCCTTGCATTTA     660
            ************************************************************

LA2093      ATCTTGTAATGTGCAAAGTTGATGCAAGAATCGTAGAGGAGGTGATATGAAAGTCAGGAT     720
E42         ATCTTGTAATGTGCAAAGTTGATGCAAGAATCGTAGAGGAGGTGATATGAAAGTCAGGAT     720
HEINZ       ATCTTGTAATGTGCAAAGTTGATGCAAGAATCGTAGAGGAGGTGATATGAAAGTCAGGAT     720
            ************************************************************

LA2093      GCTGCTGAAAATGTGACCGTCTGCTTTGCATAAATCTCTGCAAAGCTGGAATCTGAACAA     780
E42         GCTGCTGAAAATGTGACCGTCTGCTTTGCATAAATCTCTGCAAAGCTGGAATCTGAACAA     780
HEINZ       GCTGCTGAAAATGTGACCGTCTGCTTTGCATAAATCTCTGCAAAGCTGGAATCTGAACAA     780
            ************************************************************

LA2093      CAAACATACATGGAAAAATCCCAAAATATATTTAGGTTGCAAAAACAAGGAAATTATTTT     840
E42         CAAACATACATGGAAAAATCCCAAAATATATTTAGGTTGCAAAAACAAGGAAATTATTTT     840
HEINZ       CAAACATACATGGAAAAATCCCAAAATATATTTAGGTTGCAAAAACAAGGAAATTATTTT     840
            ************************************************************
```

```
LA2093      CGTTTTAAAATAAGTATTTACAGACGTAGATGAACATCCATCTAATCTGAAATAAAGCTA    900
E42         CGTTTTAAAATAAGTATTTACAGACGTAGATGAACATCCATCTAATCTGAAATAAAGCTA    900
HEINZ       CGTTTTAAAATAAGTATTTACAGACGTAGATGAACATCCATCTAATCTGAAATAAAGCTA    900
            ************************************************************

LA2093      GGAATTTGAACTGAGATCAAGTTTAAGCAAATATGCTCAGGATCAACCAGTTGTAAACCT    960
E42         GGAATTTGAACTGAGATCAAGTTTAAGCAAATATGCTCAGGATCAACCAGTTGTAAACCT    960
HEINZ       GGAATTTGAACTGAGATCAAGTTTAAGCAAATATGCTCAGGATCAACCAGTTGTAAACCT    960
            ************************************************************

LA2093      AATTCAATGAGTGTTAAGATTTGCAAGGGCAGCCATCTAGTCAGCAAAATATACATCAAG    1020
E42         AATTCAATGAGTGTTAAGATTTGCAAGGGCAGCCATCTAGTCAGCAAAATATACATCAAG    1020
HEINZ       AATTCAATGAGTGTTAAGATTTGCAAGGGCAGCCATCTAGTCAGCAAAATATACATCAAG    1020
            ************************************************************

LA2093      CATGGAATACTCAAAGAAAATACATTCAAGCATGATTTTGTCATTATCATTCTGAAATAT    1080
E42         CATGGAATACTCAAAGAAAATACATTCAAGCATGATTTTGTCATTATCATTCTGAAATAT    1080
HEINZ       CATGGAATACTCAAAGAAAATACATTCAAGCATGATTTTGTCATTATCATTCTGAAATAT    1080
            ************************************************************

LA2093      CATAGTCAATTTGAGAAAAGTCAGCAAGTTATTAGGCGTTTAAGTTGAGAGGAACTGTAA    1140
E42         CATAGTCAATTTGAGAAAAGTCAGCAAGTTATTAGGCGTTTAAGTTGAGAGGAACTGTAA    1140
HEINZ       CATAGTCAATTTGAGAAAAGTCAGCAAGTTATTAGGCGTTTAAGTTGAGAGGAACTGTAA    1140
            ************************************************************

LA2093      GTGTACTCTAACTTCAATTAATTATTCCTCTAGCGTTTCTGTTAGTTGAATACATTCCTA    1200
E42         GTGTACTCTAACTTCAATTAATTATTCCTCTAGCATTTCTGTTAGTTGAATACATTCCTA    1200
HEINZ       GTGTACTCTAACTTCAATTAATTATTCCTCTAGCGTTTCTGTTAGTTGAATACATTCCTA    1200
            ********************************** *************************

LA2093      AAGACATAAACCATGATTCAACATCCTCTCCATGGGGAGAAAAAACAAAGATGTGCTCAA    1260
E42         AAGACATAAACCATGATTCAACATCCTCTCCATGGGGAGAAAAAACAAAGATGTGCTCAA    1260
HEINZ       AAGACATAAACCATGATTCAACATCCTCTCCATGGGGAGAAAAAACAAAGATGTGCTCAA    1260
            ************************************************************

LA2093      AATGTTTAAGAAATCATTGTTTTTTCTCCTCTGTTTCCTAGCACACCAGCAACACCTGGA    1320
E42         AATGTTTAAGAAATCATTGTTTTTTCTCCTCTGTTTCCTAGCACACCAGCAACACCTGGA    1320
HEINZ       AATGATTAAGAAATCATTGTTTTTTCTCCTCTGTTTCCTAGCACACCAGCAACACCTGGA    1320
            **** *******************************************************

LA2093      CATGGCTGTATAGGATCAAGAGAGTATAATAGGCATCCTCAAACATTCCAAAACAGGTAA    1380
E42         CATGGCTGTATAGGATCAAGAGAGTATAATAGGCATCCTCAAACATTCCAAAACAGGTAA    1380
HEINZ       CATGGCTGTATAGGATCAAGAGAGTATAATAGGCATCCTCAAACATTCCAAAACAGGTAA    1380
            ************************************************************

LA2093      CACATCTTTCAGATGGACTGTACTGTTAAGTCAGGGAAGATATATTCACACCCACCAGGC    1440
E42         CACATCTTTCAGATGGACTGTACTGTTAAGTCAGGGAAGATATATTCACACCCACCAGGC    1440
HEINZ       CACATCTTTCAGATGGACTGTACTGTTAAGTCAGGGAAGATATATTCACACCCACCAGGC    1440
            ************************************************************

LA2093      AGAAATATTATAAGGTTTTCTAGTGTCTAAACATGTTTAATTTTTCAAAGCCCCTAAAAC    1500
E42         AGAAATATTATAAGGTTTTCTAGTGTCTAAACATGTTTAATTTTTCAAAGCCCCTAAAAC    1500
HEINZ       AGAAATATTATAAGGTTTTCTAGTGTCTAAACATGTTTAATTTTTCAAAGCCCCTAAAAC    1500
            ************************************************************

LA2093      AAAAAGAATCTAACTGCGAAAAAATATCAAAAGTTGCATTCTACCTCAAAACGGTTCCAAA    1560
E42         AAAAAGAATCTAACTGCAAAAAAATATCAAAAGTTGCATTCTACCTCAAAACGGTTCCAAA    1560
HEINZ       AAAAAGAATCTAACTGCGAAAAAATATCAAAAGTTGCATTCTACCTCAAAACGGTTCCAAA    1560
            ***************** ******************************************

LA2093      AGTATAAAATAAGATGTTGCATAAATGCAGCTGTTGTTGAGAGCGCTAGATAAGAAAAAC    1620
E42         AGTATAAAATAAGATGTTGCATAAATGCAGCTGTTGTTGAGAGCGCTAGATAAGAAAAAC    1620
HEINZ       AGTATAAAATAAGATGTTGCATAAATGCAGCTGTTGTTGAGAGCGCTAGATAAGAAAAAC    1620
            ************************************************************

LA2093      CTGAGAAGATAGACCATGTTGTCAGATAAGGAAGACTGGAAAAGTAAGCAAAAGCTGAAC    1680
E42         CTGAGAAGATAGACCATGTTGTCAGATAAGGAAGACTGGAAAAGTAAGCAAAAGCTGAAC    1680
HEINZ       CTGAGAAGATAGACCATGTTGTCAGATAAGGAAGACTGGAAAAGTAAGCAAAAGCTGAAC    1680
            ************************************************************

LA2093      CTTAAGTAATTTGGACATACCATAACTATAAGAAAAGAAGTAAATGTGGAAAACCAGAAA    1740
```

```
E42         CTTAAGTAATTTGGACATACCATAACTATAAGAAAAGAAGTAAATGTGGAAAACCAGAAA    1740
HEINZ       CTTAAGTAATTTGGACATACCATAACTATAAGAAAAGAAGTAAATGTGGAAAACCAGAAA    1740
            ************************************************************

LA2093      ATACAGCAAAAAGAAGCGTGGAAAGTACTTCATGGATATTGGTGTGCGTACGCTGCAAAA    1800
E42         ATACAGCAAAAAGAAGCGTGGAAAGTACTTCATGGATATTGGTGTGCGTACGCTGCAAAA    1800
HEINZ       ATACAGCAAAAAGAAGCGTGGAAAGTACTTCATGGATATTGGTGTGCGTACGCTGCAAAA    1800
            ************************************************************

LA2093      GATAACCAACATGGTTAAAATAGAGAAAACAAGGTAAACAAGAAATTAAATTGTTGAGAA    1860
E42         GATAACCAACATGGTTAAAATAGAGAAAACAAGGTAAACAAGAAATTAAATTGTTGAGAA    1860
HEINZ       GATAACCAACATGGTTAAAATAGAGAAAACAAGGTAAACAAGAAATTAAATTGTTGAGAA    1860
            ************************************************************

LA2093      TATCAATAAATAATAAAAGTGTGCTCTCTCTAACAACTTAAGCTTTTAGATGAGATGGTC    1920
E42         TATCAATAAATAATAAAAGTGTGCTCTCTCTAACAACTTAAGCTTTTAGATGAGATGGTC    1920
HEINZ       TATCAATAAATAATAAAAGTGTGCTCTCTCTAACAACTTAAGCTTTTAGATGAGATGGTC    1920
            ************************************************************

LA2093      ATACACTTCATCATGGTAGCACAGCAGGCCATCTCACCCAATGTTGGAGCCT-AAAAAAA    1979
E42         ATACACTTCATCATGGTAGCACAGCAGGCCATCTCACCCAATGTTGGAGCCTAAAAAAAA    1980
HEINZ       ATACACTTCATCATGGTAGCACAGCAGGCCATCTCACCCAATGTTGGAGCCT-AAAAAAA    1979
            **************************************************** *******

LA2093      ATCAAAATTGCCACTGGGCATGAGGTGGGTAAAAATTGCCCAAAATTTCCCGAGCATTAG    2039
E42         ATCAAAATTGCCACTGGGCATGAGGTGGGTAAAAATTGCCCAAAATTTCCCGAGCATTAG    2040
HEINZ       ATCAAAATTGCCACTGGGCATGAGGTGGGTAAAAATTGCCCAAAATTTCCCGAGCATTAG    2039
            ************************************************************

LA2093      ATGTTAAACACTGGGCGTGAGGTGGGGTGTTAAAAGCAAAAAGCCTCGCAGTGATGGTTA    2099
E42         ATGTTAAACACTGGGCGTGAGGTGGGGTGTTAAAAGCAAAAAGCCTCGCAGTGATGGTTA    2100
HEINZ       ATGTTAAACACTGGGCGTGAGGTGGGGTGTTAAAAGCAAAAAGCCTCGCAGTGATGGTTA    2099
            ************************************************************

LA2093      ATGAGATGTGTGGGCTTCTTATATAGGCTTGGACAATCCTCTTCCCTTTGAGCTATCTTT    2159
E42         ATGAGATGCGTGGGCTTCTTATATAGGCTTGGACAATCCTCTTCCCTTTGAGCTATTTTT    2160
HEINZ       ATGAGATGCGTGGGCTTCTTATATAGGCTTGGACAATCCTCTTCCCTTTGAGCTATCTTT    2159
            ******** *********************************************** ***

LA2093      TGGGGTATGAGTTAATCACATGACCTAATTTAACATGGTATCTCAGCAGACAGAAGTCAT    2219
E42         TGGGGTATGAGTTAATCACATGACCTAATTTAACATGGTATCTCAGCAGACAGAAGTCAT    2220
HEINZ       TGGGGTATGAGTTAATCACATGACCTAATTTAACATGGTATCTCAGCAGAGAGAAGTCAT    2219
            ************************************************** *********

LA2093      GATATTGAATTTCACCGCCACCCT-AAATTTAAAAGAATTTCCACGTGTTTGGCCCATGA    2278
E42         GATATTGAATTTCACTGCCACCCTAAAATTTAAAAGAATTTCCACGTGTTTGGCACATGA    2280
HEINZ       GATATTGAATTTCACCGCCACCCT-AAATTTAAAAGAATTTCCACGTGTTTGGCCCATGA    2278
            *************** ******** ***************************** *****

LA2093      AAAAGAATCAAGCGCATGTGAGGGGCGTGTTGAGAATATCATTAAATAATAAAAGTGTGC    2338
E42         AAAAGAATCAAGCGCATGTGAGGGGCGTGTTGAGAATATCATTAAATAATAAAAGTGTGC    2340
HEINZ       AAAAGAATCAAGCGCATGTGAGGGGCGTGTTGAGAATATCATTAAATAATAAAAGTGTGC    2338
            ************************************************************

LA2093      TCTGCCTAACAGTTTAAGCTTTTAGATGAGATAATCACATTTTTAATTGAACCTATTTAA    2398
E42         TCTGCCTAACAGTTTAAGCTTTTAGATGAGATAATCACATTTTTAATTGAACCTATTTAA    2400
HEINZ       TCTGCCTAACAGTTTAAGCTTTTAGATGAGATAATCACATTTTTAATTGAACCTATTTAA    2398
            ************************************************************

LA2093      GATAGCAAAATACAGACCGGGGTGAGGGGAGT--------------AGAGTTGTATGCCC    2444
E42         GATAGCAAAATACAGACCGGGGTGAGGGGAGTAGAGTTGTACGCCCAGAGTTGTATGCCC    2460
HEINZ       GATAGCAAAATACAGACCGGGGTGAGGGGAGT--------------AGAGTTGTATGCCT    2444
            ********************************              ************* 

LA2093      ATAGGCACCTTGCCCGTGGATGGCACTTACCAACCTTTCTTTTTCAGTGGTACCACAATT    2504
E42         ATAGGCACCTTGCCCGTGGATGGCACTTACCAACCTTTCTTTTTCAGTGGTACCACAATT    2520
HEINZ       ATAGGCACCTTGCCCGTGGATGGCACTTACCAACCTTTCTTTTTCAGTGGTACCACAATT    2504
            ************************************************************

LA2093      TAAGAGGGGTTTCAAGCTTCGGCCGGAATTTATCAGTTTCAACCTTTCAAAGTACAGAGT    2564
E42         TAAGAGGGGTTTCAAGCTTCGGCCGGAATTTATCAGTTTCAACCTTTCAAAGTACAGAGT    2580
HEINZ       TAAGAGGGGTTTCAAGCTTCGGCCGGAATTTATCAGTTTCAACCTTTCAAAGTACAGAGT    2564
```

```
            ************************************************************

LA2093      AAATAGCTTGTTGAGATGGAAGACAACACAGTTTATTTTGTTGTGGATCTCAAATCCGAT    2624
E42         AAATAGCTTGTTGAGATGGAAGACAACACAGTTTATTTTGTTGTGGATCTCAAATCCGAT    2640
HEINZ       AAATAGCTTGTTGAGATGGAAGACAACACAGTTTATTTTGTTGTGGATCTCAAATCCGAT    2624
            ************************************************************

LA2093      GAGGTGATGTATGTGAAGATAAATTTCCCTTTTCTGATCTTGTTAAAAACATTGAATATA    2684
E42         GAGGTGATGTATGTGAAGATAAATTTCCCTTTTCTGATCTTGTTAAAAACATTGAATATA    2700
HEINZ       GAGGTGATGTATGTGAAGATAAATTTCCCTTTTCTGATCTTGTTAAAAACATTGAATATA    2684
            ************************************************************

LA2093      CGTCTTGTCAGTATTACTGACTTTTTTGAGTGTACATCCAAGTCGGGTCTTTCTTTTGAT    2744
E42         CGTCTTGTCAGTATTACTGACTTTTTTGAGTGTACATCCAAGTCGGGTCTTTCTTTTGAT    2760
HEINZ       CGTCTTGTCAGTATTACTGACTTTTTTGAGTGTACATCCAAGTCGGGTCTTTCTTTTGAT    2744
            ************************************************************

LA2093      CTTCTGACGTCATTTACAATCTTTAAGAGCAGAATGAAAATTCTGCGACTGCAATATTTT    2804
E42         CTTCTGACGTCATTTACAATCTTTAAGAGCAGAATGAAAATTCTGCGACTGCAATATTTT    2820
HEINZ       CTTCTGACGTCATTTACAATCTTTAAGAGCAGAATGAAAATTCTGCGACTGCAATATTTT    2804
            ************************************************************

LA2093      AAAATAAAACCACTGAAATGGACTATTAGGATAGAATTGAAATTCAGCTACTTTAATTGA    2864
E42         AAAATAAAACCACTGAAATGGACTATTAGGATAGAATTGAAATTCAGCTACTTTAATTGA    2880
HEINZ       AAAATAAAACCACTGAAATGGACTATTAGGATAGAATTGAAATTCAGCTACTTTAATTGA    2864
            ************************************************************

LA2093      AATTTTTTTACTGAGACTGGAAGATTTGAAAAACTTTTAATCTCAGCTACTATAATTAAC    2924
E42         AATTTTTTTACTGAGACTGGAAGATTTGAAAAACTTTTAATCTCAGCTACTATAATTAAC    2940
HEINZ       AATTTTTTTACTGAGACTGGAAGATTTGAAAAACTTTTAATCTCAGCTACTATAATTAAC    2924
            ************************************************************

LA2093      CAAAAAAAAATAGCAGCAAAAGAAAGAGCAAAGCAGGGCAGAGAAACAGTAAAACAGGGC    2984
E42         CAAAAAAAAATAGCAGCAAAAGAAAGAGCAAAGCAGGGCAGAGAAACAGTAAAACAGGGC    3000
HEINZ       C-AAAAAAAATAGCAGCAAAAGAAAGAGCAAAGCAGGGCAGAGAAACAGTAAAACAGGGC    2983
            * **********************************************************

LA2093      AAGAAGAAAAAGAAGAAAACAGGCGAAGGAAACAGAGGAAAGAAGAGAAGAAAAAAAACC    3044
E42         AAGAAGAAAAAGAAGAAAACAGGCGAAGGAAACAGAGGAAAGAAGAGAAGAAAAAAAACC    3060
HEINZ       AAGAAGAAAAAGAAGAAAACAGGCGAAGGAAACAGAGGAAAGAAGAGAAGAAAAAAAACC    3043
            ************************************************************

LA2093      ATAGGCTTAAGTCGAGAAGCAGAAGAGAGAATGAAAGAAGGATTGCTACGAAGTCGAGAA    3104
E42         ATAGGCTTAAGTCGAGAAGCAGAAGAGAGAATGAAAGAAGGATTGCTACGAAGTCGAGAA    3120
HEINZ       ATAGGCTTAAGTCGAGAAGCAAAAGAGAGAATGAAAGAAGGATTGCTACGAAGTCGAGAA    3103
            ********************* **************************************

LA2093      GCAAAGAATTGTGTCAATACCCTAGGCTCTACAATGTCGAGTACTCCAGGTAATGTCGTA    3164
E42         GCAAAGAATTGTGTCAATACCCTAGGCTCTAAAATGTCGAGTACTCCAGGTAATGTCGTA    3180
HEINZ       GCAAAGAATTGTGTCAATACCCTAGGCTCTACAATGTCGAGTACTCCAGGTAATGTCGTA    3163
            ******************************* ****************************

LA2093      ACTTGTATTTACAATTAAAATCATAGTTTCGAAAAAACAAAAGCAATTTTTTTGTGCGCT    3224
E42         ACTTGTATTTACAATTAAAATCATAGTTTCGAAAAAACAAAAGCAATTTTTTTGTGCGCT    3240
HEINZ       ACTTGTATTTACAATTAAAATCATAGTTTCGAAAAAACAAAAGCAATTTTTTTGTGCGCT    3223
            ************************************************************

LA2093      TCACACTTCACGCTCTGCCATTGTAGCGGTTGCTAATCTTCTCCGAGGTCGCTTCAGGTT    3284
E42         TCACACTTCACGCTCTGCCATTGTAGCGGTTGCTAATCTTCTCCGAGGTCGCTTCAGGTC    3300
HEINZ       TCACACTTCACGCTCTGCCATTGTAGCGGTTGTTAATCTTCTCCGAGGTCGCTTCAGGTT    3283
            ******************************** ************************** 

LA2093      GCTTCTCTTGGCTTCACCGCTATAAGCGTTTTAAATAGTTATTCCTTTAAGAACTGCCCA    3344
E42         GCTTCTCTTGGCTTCACCGCTATAAGCGTTTTAAATAGTTATTCCTTTAAGAACTGCCCA    3360
HEINZ       GCTTCTCTTGGCTTCACCGCTATAAGCGTTTTAAATAGTTATTCCTTTAAGAACTGCCCA    3343
            ************************************************************

LA2093      GTTTTTCAGCTGCCGTTATAGTTTGTAGTTTCAATAGTTTTCAGATTTAGTTTCAGACTC    3404
E42         GTTTTTCAGCTGCCGTTATAGTTTGTAGTTTCAATAGTTTTCAGATTTAGTTTCAGACTC    3420
HEINZ       GTTTTTCAGCTGCCGTTATAGTTTGTAGTTTCAATAGTTTTCAGATTTAGTTTCAGACTC    3403
            ************************************************************
```

```
LA2093      AGTTTTGAAATTCAATTTTAGCTCACAGGTTCACAATATTTATTATGTAAATTACTTGCG    3464
E42         AGTTTTGAAATTCAATTTTAGCTCACAGGTTCACAATATTTATTATGTAAATTACTTGCG    3480
HEINZ       AGTTTTGAAATTCATTTTTAGCTCACAGGTTCACAATATTTATTATGTAAATTACTTGCG    3463
            ************** *********************************************

LA2093      TTACTTTTGTCATTTCATTATATTACATAATTATTCCAGACTAGTCTCGTCCTCAACTCA    3524
E42         TTACTTTTGTCATTTCATTATATTACATAATTATTCCAGACTAGTCTCGTCCTCAACTCA    3540
HEINZ       TTACTTTTGTCATTTCATTATATTACATAATTATTCCAGACTAGTCTCGTCCTCAACTCA    3523
            ************************************************************

LA2093      GGGAGTAACCAAACTTATTGGTATCCTCCAGAGGTAGTTACCCGTTTGGTGTCGCATTGT    3584
E42         GGGAGTAACCAAACTTATTGGTATCCTCCAGAGGTAGTTACCCGTTTGGTGTCGCATTGT    3600
HEINZ       GGGAGTAACCAAACTTATTGGTATCCTCCAGAGGTAGTTACCCGTTTGGTGTCGCATTGT    3583
            ************************************************************

LA2093      ACGGTAGGTGTTGAGTGCGCAGGTGAAGAAGCATGTGGCTAGGGGCAGACTTTCTAAGTT    3644
E42         ACGGTAGGTGTTGAGTGCGCAGGTGAAGAAGCATGTGGCTAGGGGCAGACTTTCTAAGTT    3660
HEINZ       ACGGTAGGTGTTGAGTGCGCAGGTGAAGAAGCATGTGGCTAGGGGCAGACTTTCTAAGTT    3643
            ************************************************************

LA2093      TATTGACAGGCTCCGTTGATTTGTTCGCAGCAGTAGTAGACCGTTCCAAGAGTTTCATTT    3704
E42         TATTGACAGGCTCCGTTGATTTGTTCGCAGCAGTAGTAGACCGTTCCAAGAGTTTCATTT    3720
HEINZ       TATTGACAGGCTCCGTTGATTTGTTCGCAGCAGTAGTAGACCGTTCCAAGAGTTTCATTT    3703
            ************************************************************

LA2093      GTTTAAAACGCTATGTATTTCCTGGGAATGCCCCAAAGGCATTGTTATAGATTGCAGTCC    3764
E42         GTTTAAAACGCTATGTATTTCCTGGGAATGCCCCAAAGGCATTGTTATAGATTGCAGTCC    3780
HEINZ       GTTTAAAACGCTATGTATTTCCTGGGAATGCCCCAAAGGCATTGTTATAGATTGCAGTCC    3763
            ************************************************************

LA2093      TTAGAAGCTCATGACTTATCAGTGGATTAGTTTTCAGAGGTTTTAACGTAATGTTTGTAT    3824
E42         TTAGAAGCTCATGACTTATCAGTGGATTAGTTTTCAGAGGTTTTAACGTAATGTTTGTAT    3840
HEINZ       TTAGAAGCTCATGACTTATCAGTGGATTAGTTTTCAGAGGTTTTAACGTAATGTTTGTAT    3823
            ************************************************************

LA2093      TTCAACTTTTCAAATCATTGATTACATACAAGCTTTTATTGTTATGATTAGTTTCTTAAT    3884
E42         TTCAACTTTTCAAATCATTGATTACATACCAGCTTTTATTGTTATGATTAGTTTCTTAAT    3900
HEINZ       TTCAACTTTTCAAATCATTGATTACATACAAGCTTTTATTGTTATGATTAGTTTCTTAAT    3883
            ***************************** ******************************

LA2093      AGCCATCAGACAGGACAGGGTTTACTCGACGGGTATTACGAGTTGGGTGCCAATCATTTG    3944
E42         AGCCATCAGACAGGACAGGGTTTACTCGACGGGTATTACGAGTTGGGTGCCAATCATTTG    3960
HEINZ       AGCCATCAGACAGGACAGGGTTTACTCGACGGGTATTACGAGTTGGGTGCCAATCATTTG    3943
            ************************************************************

LA2093      GCACACTAATTTGGGAACATCAGACTACAATTCTTTTTCTCCACCGAACATCTTCTGTCT    4004
E42         GCACACTAATTTGGGAACATCAGACTACAATTCTTTTTCTCCACCGAACATCTTCTGTCT    4020
HEINZ       GCACACTAATTTGGGAACATCAGACTACAATTCTTTTTCTCCACCGAACATCTTCTGTCT    4003
            ************************************************************

LA2093      TGGCAAATAAATATTAGAAATT-AAAACAAAAAATGTAAAACTATGCTCACAAATAGTGA    4063
E42         TGGCAAATAAATATTAGAAATTAAAAACAAAAAATGTAAAACTATGCTCACAAATAGTGA    4080
HEINZ       TGGCAAATAAATATTAGAAATT-AAAACAAAAAATGTAAAACTATGCTCACAAATAGTGA    4062
            ********************** *************************************

LA2093      TAAATACAAGAGTACAGGTTTATTCTTCTGACCAATACATAATTGGCAGAAGTTGCTGAT    4123
E42         TAAATACAAGAGTACAGGTTTATTCTTCTGACCAATACATAATTGGCAGAAGTTGCTGAT    4140
HEINZ       TAAATACAAGAGTACAGGTTTATTCTTCTGACCAATACATAATTGGCAGAAGTTGCTGAT    4122
            ************************************************************

LA2093      CAAGAAACTAGCAGACTAACAAAATGCTGAAGGGATCTTCCAAGCAGGAAATGTGTGGCT    4183
E42         CAAGAAACTAGCGGACTAACAAAATGCTGAAGGGATCTTCCAAGCAGGAAATGTGTGGCT    4200
HEINZ       CAAGAAACTAGCAGACTAACAAAATGCTGAAGGGATCTTCCAAGCAGGAAATGTGTGGCT    4182
            ************ ***********************************************

LA2093      GTTATTAAGGGAGCACCAGATCATTTTCAAATATTAGGCAAAATCTCAGGCAGTATGAAT    4243
E42         GTTATTAAGGGAGCACCAGATCATTTTCAAATATTAGGCAAAATCTCAGGCAGTATGAAT    4260
HEINZ       GTTATTAAGGGAGCACCAGATCATTTTCAAATATTAGGCAAAATCTCAGGCAGTATGAAT    4242
            ************************************************************

LA2093      AGGGACTTCATATTTCAAGCAGCTTTAAGAGGTGTTGAAACAGTTTTCCTTCAATCAACT    4303
E42         AGGGACTTCATATTTCAAGCAGCTTTAAGAGGTGTTGAAACAGTTTTCCTTCAATCAACT    4320
```

HEINZ AGGGACTTCATATTTCAAGCAGCTTTAAGAGGTGTTGAAACAGTTTTCCTTCAATCAACT 4302
****

LA2093 AGATATAGAGCCTGACAATCATCACCCAAAAATTTTTAGGACCCATCTGGATGTACACAA 4363
E42 AGATATAGAGCCTGACAATCATCACCCAAAAATTTTTAGGACCCATCTGGATGTACACAA 4380
HEINZ AGATATAGAGCCTGACAATCATCACCCAAAAATTTTTAGGACCCATCTGGATGTACACAA 4362
****

LA2093 CTATAGACGTTTCTGAGGTTCCAAACTTAAATCATACTAGTATGAAAAGGGGAAGACCAA 4423
E42 CTATAGACGTTTCTGAGGTTCCAAACTTAAATCATACTAGTATGAAAAGGGGAAGACCAA 4440
HEINZ CTATAGACGTTTCTGAGGTTCCAAACTTAAATCATACTAGTATGAAAAGGGGAAGACCAA 4422
****

LA2093 AGCATGTGTTACATAGGACAGTCAAGTAGCATTAGCACTTAGATTATCAGGAAGTTAAAT 4483
E42 AGCATGTGTTACATAGGACAGTCAAGTAGCATTAGCACTTAGATTATCAGGAAGTTAAAT 4500
HEINZ AGCATGTGTTACATAGGACAGTCAAGTAGCATTAGCACTTAGATTATCAGGAAGTTAAAT 4482
****

LA2093 AACTTGGGTCGCTACCAAGTACAATCTGAATGTCATCTTTTAACTAATAGGGAGTAGTAA 4543
E42 AACTTGGGTCGCTACCAAGTACAATCTGAATGTCATGTTTTAACTAATAGGGAGTAGTAA 4560
HEINZ AACTTGGGTCGCTACCAAGTACAATCTGAATGTCATCTTTTAACTAATAGGGAGTAGTAA 4542
************************************ ***********************

LA2093 TCCATTTCTATGTGCAGGACAGCACATGTGACACACACACACACACACACAGAGAGAGAG 4603
E42 TCCATTTCTATCTGCAGGACAGCACATGTGACACACACACACACACACAGAGAGAGAGAG 4620
HEINZ TCCATTTCTATGTGCAGGACAGCACATGTGACACACACACACACACACAGAGAGAGAGAG 4602
*********** ************************************* **********

LA2093 AGAGAGAGAGACCTGATTAATGTGAAAAGTTCGCACAGCCAAACAAAAATTAACACCATG 4663
E42 AGAGAGAGAGACCTGATTAATGTGAAAAGTTCGCACAGCCAAACAAAAATTAACACCATG 4680
HEINZ AGAGAGAGAGACCTGATTAATGTGAAAAGTTCGCACAGCCAAACAAAAATTAACACCATG 4662
****

LA2093 AAAGCCAGCAGCTGATCATCATAGAACTCGAACAAAAAAAATAGGATGCCTATCATGATC 4723
E42 AAAGCCAGCAGCTGATCATCATAGAACTCGAACAAAAAAAATAGGATGCCTATCATGATC 4740
HEINZ AAAGCCAGCAGCTGATCATCATAGAACTCGAACAAAAAAAATAGGATGCCTATCATGATC 4722
****

LA2093 TGCAGGAACTCTAAGTTATACCTGAATGGCCTTGAATTTATAACCAACATTCACCGACTA 4783
E42 TGCAGGAACTCTAAGTTATACCTGAATGGCCTTGAATTTATAACCAACATTCACCGACTA 4800
HEINZ TGCAGGAACTCTAAGTTATACCTGAATGGCCTTGAATTTATAACCAACATTCACCGACTA 4782
****

LA2093 CAAAATATACATCGGATCAACTGGGAAATCTTACCGGTACAAAAACAAGGGACTCAATTA 4843
E42 CAAAATATACATCGGATCAACTGGGAAATCTTACCGGTACAAAAACAAGGGACTCAATTA 4860
HEINZ CAAAATATACATCGGATCAACTGGGAAATCTTACCGGTACAAAAACAAGGGACTCAATTA 4842
****

LA2093 CGTGCACAAAGATCAATTGAAATGTTGGAAGCCTATGCCGAGCATGATGCTGTAGCTGCA 4903
E42 CGTGCACAAAGATCAATTGAAATGTTGGAAGCCTATGCCGAGCATGATGCTGTAGCTGCA 4920
HEINZ CGTGCACAAAGATCAATTGAAATGTTGGAAGCCTATGCCGAGCATGATGCTGTAGCTGCA 4902
****

LA2093 CTGCACATATTTTTACAGTAGAAATGTCTAAGAAAGGAAAGGAAGGCTACATCAAACAAA 4963
E42 CTGCACATATTTTTACAGTAGAAATGTCTAAGAAAGGAAAGGAAGGCTACATCAAACAAA 4980
HEINZ CTGCACATATTTTTACAGTAGAAATGTCTAAGAAAGGAAAGGAAGGCTACATCAAACAAA 4962
****

LA2093 GTATAGACAACAAGCAGTAGAGGCTCAACAAATCAACAATTAAACAGCTTGTTTAGAATT 5023
E42 GTATAGGCAACAAGCAGTAGAGGCTCAACAAATCAACAATTAAACAGCTTGTTTAGAATA 5040
HEINZ GTATAGACAACAAGCAGTAGAGGCTCAACAAATCAACAATTAAACAGCTTGTTTAGAATA 5022
****** **

LA2093 CAAGATCAACAGAATATTTGTATATGAAAGTTGGGTGCTTCATACTTTGACAACAGCATG 5083
E42 CAAGATCAACAGAATATTTGTATATGAAATTTGGGTGCTTCATACTTTGACAACAGCATG 5100
HEINZ CAAGATCAACAGAATATTTGTATATGAAAGTTGGGTGCTTCATACTTTGACAACAGCATG 5082
***************************** ******************************

LA2093 ACCATACCTTTCTTCTTTGTTTTCATGTTGACATGTAATTTAAGCAATAGAACATGATTG 5143
E42 ACCATACCTTTCTTCTTTGTTTTCATGTTGACATGTAATTTAAGCAATAGAACATGATTG 5160
HEINZ ACCATACCTTTCTTCTTTGTTTTCATGTTGACATGTAATTTAAGCAATAGAACATGATTG 5142
****

```
LA2093      AGAAGTATCCAGATGTGCAAGGCATTATAATTTCACGATATAAAAACACCACTGCAGTCT    5203
E42         AGAAGTATCCAGATGTGCAAGGCATTATAATTTCACGATATAAAAACACCACTGCAGTCT    5220
HEINZ       AGAAGTATCCAGATGTGCAAGGCATTATAATTTCACGATATAAAAACACCACTGCAGTCT    5202
            ************************************************************

LA2093      GTGGCATCAAATATACAAAAGGGAACCTACCCGTAAACTTCAGCATGCGTGTTTGGGTCT    5263
E42         GTGGCATCAAATATACAAAAGGGAACCTACCCGTAAACTTCAGCATGCGTGTTTGGGTCT    5280
HEINZ       GTGGCATCAAATATACAAAAGGGAACCTACCCGTAAACTTCAGCATGCGTGTTTGGGTCT    5262
            ************************************************************

LA2093      CTCTCAATGTAAATGAAACTGACATTGTCGTGGTAAAGAAAACAAAGAGTGACATCATGA    5323
E42         CTCTCAATGTAAATGAAACTGACATTGTCGTGGTAAAGAAAACAAAGAGTGACATCATGA    5340
HEINZ       CTCTCAATGTAAATGAAACTGACATTGTCGTGGTAAAGAAAACAAAGAGTGACATCATGA    5322
            ************************************************************

LA2093      GTACACCACACTTTGTGACAAGGTAGTCTGCAAGAAATATGAAGCAAATATTCAAATGGG    5383
E42         GTACACCACACTTTGTGACAAGGTAGTCTGCAAGAAATATGAAGCAAATATTCAAATGGG    5400
HEINZ       GTACACCACACTTTGTGACAAGGTAGTCTGCAAGAAATATGAAGCAAATATTCAAATGGG    5382
            ************************************************************

LA2093      ACCTAATGATACGTTAGGAAGTGCTCACAACAAATCTAACATGTATTTAAGGTGATCATC    5443
E42         ACCTAATGATACGTTAGGAAGTGCTCACAACAAATCTAACATGTATTTAAGGTGATCATC    5460
HEINZ       ACCTAATGATACGTTAGGAAGTGCTCACAACAAATCTAACATGTATTTAAGGTGATCATC    5442
            ************************************************************

LA2093      ATGACTTCATATGATCAATATACATGCAAGTGCATGTACCAGTATGTCAGATGGAATGTT    5503
E42         ATGACTTCATATGATCAATATACATGCAAGTGCATGTACCAGTATGTCAGATGGAATGTT    5520
HEINZ       ATGACTTCATATGATCAATATACATGCAAGTGCATGTACCAGTATGTCAGATGGAATGTT    5502
            ************************************************************

LA2093      TTGTTTTTTTGATGAAGTAAGAAGAATGTCATTAACAAAGCATCAAGCAGATGCACAGAT    5563
E42         TTGTTTTTTTGATGAAGTAAGAAGAATGTCATTAACAAAGCATCAAGCAGATGCACAGAT    5580
HEINZ       TTGTTTTTTTGATGAAGTAAGAAGAATGTCATTAACAAAGCATCAAGCAGATGCACAGAT    5562
            ************************************************************

LA2093      TACAAAAGAAAGAAATATCAGCTCCCAGAAAGG-AAAAAAAAGCTATCTAAACAGCTAAC    5622
E42         TACAAAAGAAAGAAATATCAGCTCCCAGAAAGG-AAAAAAAAGCTATCTAAACAGCTAAC    5639
HEINZ       TACAAAAGAAAGAAATATCAGCTCCCAGAAAGGAAAAAAAAAGCTATCTAAACAGCTAAC    5622
            ********************************* **************************

LA2093      AAGAGAGATCTATGGAGCTGATAAATTCTAGGTAAGAATCGACACTAAGTACAGGAGAGA    5682
E42         AAGAGAGATCTATGGAGCTGATAAATTCTAGGTAAGAATCGACACTAAGTACAGGAGAGA    5699
HEINZ       AAGAGAGATCTATGGAGCTGATAAATTCTAGGTAAGAATCGACACTAAGTACAGGAGAGA    5682
            ************************************************************

LA2093      GATTAACCCAAAAAAATAATAATAATCAAACATCTTGCTTTAACGGTAGAGATGGGAGTT    5742
E42         GATTAACCCAAAAAAATAATAATAATCAAACATCTTGCTTTAACGGTAGAGATGGGAGTT    5759
HEINZ       GATTAACCCAAAAAAATAATAATAATCAAACATCTTGCTTTAACGGTAGAGATGGGAGTT    5742
            ************************************************************

LA2093      GGGGTGCCATCAAAACATCTTTTGTTCCTCTCCGTCCATAAGCACCACAAAATACAGGCT    5802
E42         GGGGTGCCATCAAAACATCTTTTGTTCCTCTCCGTCCATAAGCACCACAAAATACAGGCT    5819
HEINZ       GGGGTGCCATCAAAACATCTTTTGTTCCTCTCCGTCCATAAGCACCACAAAATACAGGCT    5802
            ************************************************************

LA2093      GGAACCAAAGACCAGATCCGCTTGATGGGCTTCCCAACTTTCCATGAACTCCAACAGACA    5862
E42         GGAACCAAAGACCAGATCCGCTTGATGGGCTTCCCAACTTTCCATGAACTCCAACAGACA    5879
HEINZ       GGAACCAAAGACCAGATCCGCTTGATGGGCTTCCCAACTTTCCATGAACTCCAACAGACA    5862
            ************************************************************

LA2093      AAGGCAAGACCAAGAGAGTCCAAAAAGTGAGAAGATAATGTTTCATAGGCCTGTTGCAAC    5922
E42         AAGGCAAGACCAAGTGAGTCCAAAAAGTGAGAAGATAATGTTTCATAGGCCTGTTGCAAC    5939
HEINZ       AAGGCAAGACCAAGAGAGTCCAAAAAGTGAGAAGATAATGTTTCATAGGCCTGTTGCAAC    5922
            ************** *********************************************

LA2093      TGTGCAATGAAGGAACAAGTGATTAATAGATTCTGAGTTGCACAAGCACATGTAGCATCT    5982
E42         TGTGCAATGAAGGAACAAGTGATTAATAGATTCTGAGTTGCACAAGCACATGTAGCATCT    5999
HEINZ       TGTGCAATGAAGGAACAAGTGATTAATAGATTCTGAGTTGCACAAGCACATGTAGCATCT    5982
            ************************************************************

LA2093      GTTGACTATTTGGAATTTCCTCCTCATGAGATTATTCTGAGTTAAGCAAGAGTCTGTTAG    6042
```

```
E42         GTTGACTATTTGGAATTTCCTCCTCATGAGATTATTCTGAGTTAAGCAAGAGTCTGTTAG    6059
HEINZ       GTTGACTATTTGGAATTTCCTCCTCATGAGATTATTCTGAGTTAAGCAAGAGTCTGTTAG    6042
            ************************************************************

LA2093      AGTAATCCAACTGAAACAAATAACTTTAGGTGGCAACTTAGTTTTCCAGATTAATATTCA    6102
E42         AGTAATCCAACTGAAACAAATAACTTTAGGTGGCAACTTAGTTTTCCAGATTAATATTCA    6119
HEINZ       AGTAATCCAACTGAAACAAATAACTTTAGGTGGCAACTTAGTTTTCCAGATTAATATTCA    6102
            ************************************************************

LA2093      TGGTCATTGATCAATTAAGGTTTTCTTGGAGCTTTGTTGAAGGTAGCATGCTTTAACTGA    6162
E42         TGGCCATTGATCAATTAAGGTTTTCTTGGAGCTTTGTTGAAGGTAGCATGCTTTAACTGA    6179
HEINZ       TGGTCATTGATCAATTAAGGTTTTCTTGGAGCTTTGTTGAAGGTAGCATGCTTTAACTGA    6162
            *** ********************************************************

LA2093      GTAGGATACGTCCGTGCTATGTCTCCAGATAAGTTTGTCCCTGCGTTGACTATTGATTGC    6222
E42         GTAGGATACGTCCGTGCTATGTCTCCAGATAAGTTTGTCCCTGCGTTGACTATTGATTGC    6239
HEINZ       GTAGGATACGTCCGTGCTATGTCTCCAGATAAGTTTGTCCCTGCGTTGACTATTGATTGC    6222
            ************************************************************

LA2093      ACTAGTCTGAATGGTGGCTATAAGGCTAAAAACCTCTTCCAACTCCCAGTCATTAAGATT    6282
E42         ACTAGTCTGAATGGTGGCTATAAGGCTAAAAACCTCTTCCAACTCCCAGTCATTAAGATT    6299
HEINZ       ACTAGTCTGAATGGTGGCTATAAGGCTAAAAACCTCTTCCAACTCCCAGTCATTAAGATT    6282
            ************************************************************

LA2093      TCTCCTTAGGATCAGAATCCACACATTGTCTTCCCTGTACTGCGAGATGGTTGAGTCTGG    6342
E42         TCTCCTTAGGATCAGAATCCACACGTTGTCTTCCCTGTACTGCGAGATGGTTGAGTCTGG    6359
HEINZ       TCTCCTTAGGATCAGAATCCACACGTTGTCTTCCCTGTACTGCGAGATGGTTGAGTCTGG    6342
            ************************ ***********************************

LA2093      ATGAGATGCAATCTGGAAAATGTTTGGAAAAACATTCATTAGAGGAGTATCACCTAGCCA    6402
E42         ATGAGATGCAATCTGGAAAATGTTTGGAAAAACATTCATTAGAGGAGTATCACCTAGCCA    6419
HEINZ       ATGAGATGCAATCTGGAAAATGTTTGGAAAAACATTCATTAGAGGAGTATCACCTAGCCA    6402
            ************************************************************

LA2093      AATATCTTTCCAAAACTGAATATGTTCCCTATTTCCCACCATAAGTGAGGCATCACTGAA    6462
E42         AATATCTTTCCAAAACTGAATATGTTCCCTATTTCCCACCATAAGTGAGGCATCACTGAA    6479
HEINZ       AATATCTTTCCAAAACTGAATATGTTCCCTATTTCCCACCATAAGTGAGGCATCACTGAA    6462
            ************************************************************

LA2093      GAATATTTCCCACAGTTTCCTTGTATCTCTCCGAACCCCGTACCATAAGAGGTACTGACT    6522
E42         GAATATTTCCCACAGTTTCCTTGTATCTCTCCGAACCCCGTACCATAAGAGGTACTGACT    6539
HEINZ       GAATATTTCCCACAGTTTCCTTGTATCTCTCCGAACCCCGTACCATAAGAGGTACTGACT    6522
            ************************************************************

LA2093      CTATTAGAGCACCAATGACTATCACTACCATACTTTGCTTGGATCACTTCCTTCCACAAG    6582
E42         CTATTAGAGCACCAATGACTATCACTACCATACTTTGCTTGGATCACTTCCTTCCACAAG    6599
HEINZ       CTATTAGAGCACCAATGACTATCACTACCATACTTTGCTTGGATCACTTCCTTCCACAAG    6582
            ************************************************************

LA2093      CCAGCCCCTCTTGATTGTATCTCCAGTGCCACTTCAAAAGCATACACTTGTTATGCAAGG    6642
E42         TCAGCCCCTCTTGATTGTATCTCCAGTGCCACTTCAATAGCATACACTTGTTATGCAAGG    6659
HEINZ       CCAGCCCCTCTTGATTGTATCTCCAGTGCCACTTCAATAGCATACACTTGTTATGCAAGG    6642
             ************************************ **********************

LA2093      CCAAGTCTTTTATCCCCAATCCTCCACATTGTTTTGGCAAAGTGACTTTGGCCCATTTAA    6702
E42         CCAAGTCTTTTATCCCCAATCCTCCACATTGTTTTGGCAAAGTGACTTTGGCCCATTTAA    6719
HEINZ       CCAAGTCTTTTATCCCCAATCCTCCACATTGTTTTGGCAAAGTGACTTTGGCCCATTTAA    6702
            ************************************************************

LA2093      CAAGGTGAATTTTGTGGTCCATGTTGTTGCCTTCCCAGAGAGAGTCTCTCCTGATTTTGT    6762
E42         CAAGGTGAATTTTGTGGTCCATGTTGTTGCCTTCCCAGAGAGAGTCTCTCCTGATTTTGT    6779
HEINZ       CAAGGTGAATTTTGTGGTCCATGTTGTTGCCTTCCCAGAGAGAGTCTCTCCTGATTTTGT    6762
            ************************************************************

LA2093      CAAGTTGTTGTTTCACCTGAGCTGGCATAAGGAGTAAGGACATAAAATAGGTGGGAATGT    6822
E42         CAAGTTGTTGTTTCACCTGAGCTGGCATAAGGAGTAAGGACATAAAATAGGTGGGAATGT    6839
HEINZ       CAAGTTGTTGTTTCACCTGAGCTGGCATAAGGAGTAAGGACATAAAATAGGTGGGAATGT    6822
            ************************************************************

LA2093      TGTCCAATACACTACTGATGAGTGTTGATCTTCCTCATAGAGAGAGGTATTGTTGTTGTG    6882
E42         TGTCCAATACACTACTGATGAGTGTTGATCTTCCTCATAGAGAGAGGTATTGTTGTTGCG    6899
HEINZ       TGTCCAATACACTACTGATGAGTGTTGATCTTCCTCATAGAGAGAGGTATTGTTGTTGTG    6882
```

```
            ********************************************************** *

LA2093      AGGAGGCTAACCTTCTCTCAAACTTTTCCACAATCACATTCCAAACTTAAGATGATCTAT     6942
E42         AGGAGGCTAACCTTCTCTCAAACTTTTCCACAATCACATTCCAAACTTAAGATGATCTAT     6959
HEINZ       AGGAGGCTAACCTTCTCTCAAACTTTTCCACAATCACATTCCAAACTTAAGATGATCTAT     6942
            ************************************************************

LA2093      TCATGGCATCCAAAGGCATACCCAAGTAGGTGGTGGGAAAGGAGCCATTAGGGCAACACA     7002
E42         TCATGGCATCCAAAGGCATACCCAAGTAGGTAATGGGAAAGGAGCCATTAGGGCAACACA     7019
HEINZ       TCATGGCATCCAAAGGCATACCCAAGTAGGTGGTGGGAAAGGAGCCATTAGGGCAACACA     7002
            *******************************  ***************************

LA2093      TGATATCTGCCATATCTTCCAGATCAGGGACCTCATTTACAGGGTATACACGCTCTTGGA     7062
E42         TGATATCTGCCATATCTTCCAGATCAGGGACCTCATTTACAGGGTATACACGCTCTTGGA     7079
HEINZ       TGATATCTGCCATATCTTCCAGATCAGGGACCTCATTTACAGGGTATACACGCTCTTGGA     7062
            ************************************************************

LA2093      CATGTTGATGTGGAGACCTGACAATGCTTCAAAGAGCATAAGGGTGAGACTAAGATATTG     7122
E42         CATGTTGATGTGGAGACCTGACAATGCTTCAAAGAGCATAAGGGTGAGACTAAGATATTG     7139
HEINZ       CATGTTGATGTGGAGACCTGACAATGCTTCAAAGAGCATAAGGGTGAGACTAAGATATTG     7122
            ************************************************************

LA2093      CACTTGAGATTTCTTAGATCCACAGAAAACCAAGGTATCATCAACAAATAAAAGATGTGA     7182
E42         CACTTGAGATTTCTTAGCTCCACAGAAAACCAAGGTGTCATCAACAAATAAAAGATGTGA     7199
HEINZ       CACTTGAGATTTCTTAGATCCACAGAAAACCAAGGTGTCATCAACAAATAAAAGATGTGA     7182
            ***************** ******************* ***********************

LA2093      GATCGAGACTGGTGGACCATAACCATTGCCCACCTCAAAACCTTATATCCATTGAAGTTG     7242
E42         GATCGAGACTGGTGGACCATAACCATTGCCCACCTCAAAACCTTATATCCATTGAAGTTG     7259
HEINZ       GATCGAGACTGGTGGACCATAACCATTGCCCACCTCAAAACCTTATATCCATTGAAGTTG     7242
            ************************************************************

LA2093      TTTAGCCTTCTCCAACATCCTACTCAAACCTCCATAGCCAAAACAAAAAGGAAGAGTGAT     7302
E42         TTTAGCCTTCTCCAACATCCTACTCAAACCTCCATAGCCAAAACAAAAAGGAAGAGTGAT     7319
HEINZ       TTTAGCCTTCTCCAACATCCTACTCAAACCTCCATAGCCAAAACAAAAAGGAAGAGTGAT     7302
            ************************************************************

LA2093      AAGGGGTCCCCTTGCCTGATGCCTCTATCGGGAGAGAAAACCCACAGGACTTCTATTCAC     7362
E42         AAGGGGTCCCCTTGCCTGATGCCTCTATCGGGAGAGAAAACCCACAGGACTTCTATTCAC     7379
HEINZ       AAGGGGTCCCCTTGCCTGATGCCTCTATCGGGAGAGAAAACCCACGGGACTTCTATTCAC     7362
            ********************************************* **************

LA2093      TAGTACAAAGTACATTGTGGTGGAGAAGCTATACTTGATCCACCTAATCCTTCTCTCTCC     7422
E42         TAGTACAAAGTACATTGTGGTGGAGAAGCTATACTTGATCCACCTAATCCTTCTCTCTCC     7439
HEINZ       TAGTACAAAGTACATTGTGGTGGAGAAGCTATACTTGATCCACCTAATCCTTCTCTCTCC     7422
            ************************************************************

LA2093      AAATCCCATTTGCCTAGGATTGAACCAAGGAAGGACCAACTCAATTTATCAAACACCTTC     7482
E42         AAATCCCATTTGCCTAGGATTGAACCAAGGAAGGACCAACTCAATTTATCAAACACCTTC     7499
HEINZ       AAATCCCATTTGCCTAGGATTGAACCAAGGAAGGACCAACTCAATTTATCAAACACCTTC     7482
            ************************************************************

LA2093      TCAATATCAAGTTTAAAAGGCAGACCTGGAGTACCACTTTTTTGCTTCCAATCTAGAACT     7542
E42         TCAATATCAAGTTTAAAAGGCAGACCTGGAGTACCACTTTTTTGCTTCCAATCTAGAACT     7559
HEINZ       TCAATATCAAGTTTAAAAGGCAGACCTGGAGTACCACTTTTTTGCTTCCAATCTAGAACT     7542
            ************************************************************

LA2093      TCATTGGTAATAAGTGTTGCATCAGTGATTTGCCTTCCCTGAACAAAAGCATTCTAAAAA     7602
E42         TCATTGGTAATAAGTGTTGCATCAGTGATTTGCCTTCCCTGAACAAAAGCATTCTAAAAA     7619
HEINZ       TCATTGGTAATAAGTGTTGCATCAGTGATTTGCCTTCCCTGAACAAAAGCATTCTAAAAA     7602
            ************************************************************

LA2093      CCAGAGACTAGATTCCCAATCACCTTCTTTAGCCTTTCTGAAAGCACTTTGGCAAAATTT     7662
E42         CCAGAGACTAGATTCCCAATCACCTTCTTTAGCCTTTCTGAAAGCACTTTGGCAAAATTT     7679
HEINZ       CCAGAGACTAGATTCCCAATCACCTTCTTTAGCCTTTCTGAAAGCACTTTGGCAAAATTT     7662
            ************************************************************

LA2093      TGTACACACTACCAATATGACTGATAGGCCTAAAATCTTTAAGTTCTATCGCACCTTTCA     7722
E42         TGTACACACTACCAATATGACTGATAGGCCTAAAATCTTTAAGTTCTATCGCACCTTTCA     7739
HEINZ       TGTACACACTACCAATATGACTGATAGGCCTAAAATCTTTAAGTTCTATCGCACCTTTCA     7722
            ************************************************************
```

```
LA2093      TTAGGGACAAGTGCAATAAAAGAGGCATTAAAAGATTTCACCATGTGGCAGTTGTTGTGG    7782
E42         TTAGGGACAAGTGCAATAAAAGAGGCATTAAAAGATTTCACCATGTGGCAGTTGTTGTGG    7799
HEINZ       TTAGGGACAAGTGCAATAAAAGAGGCATTAAAAGATTTCACCATGTGGCAGTTGTTGTGG    7782
            ************************************************************

LA2093      AAGTGATTGAGCGCACCTAAAATATCTTGCTTGATAATTCCCCAGCACTTTTGATAAAAC    7842
E42         AAGTGATTGAGCGCACCTAAAATATCTTGCTTGATAATTCCCCAGCACTTTTGATAAAAC    7859
HEINZ       AAGTGATTGAGCGCACCTAAAATATCTTGCTTGATAATTCCCCAGCACTTTTGATAAAAC    7842
            ************************************************************

LA2093      CCCATAGAGAAGCCACTTGGCCCTGGACTTTCTGGGGCACATGAGTTGATTGCATTCAAT    7902
E42         CCCATAGAGAAGCCACTTGGCCCTGGACTTTCTGGGGCACATGAGTTGATTGCATTCAAT    7919
HEINZ       CCCATAGAGAAGCCACTTGGCCCTGGACTTTCTGGGGCACATGAGTTGATTGCATTCAAT    7902
            ************************************************************

LA2093      ATTTATTTTTCTTCAAAGGTTTGCTCCAAAGCAGTCTTCTCTTCAAAGCTCAAACTGCCC    7962
E42         ATTTATTTTTCTTCAAAGGTTTGCTCCAAAGCAGTCTTCTCTTCAAAGCTCAACCTGCCC    7979
HEINZ       ATTTATTTTTCTTCAAAGGTTTGCTCCAAAGCAGTCTTCTCTTCAAAGCTCAAACTGCCC    7962
            ***************************************************** ******

LA2093      AGACCCTCAAAAGAAGCAGTAATCCTCCATGATACGTTTTCAAAGTATATCCTGGTAGAA    8022
E42         AGACCCTCAAAAGAAGCAGTAATCCTCCATGATTCGTTTTCAAAGTATATCCTGGTAGAA    8039
HEINZ       AGACCCTCAAAAGAAGCAGTAATCCTCCATGATTCGTTTTCAAAGTATATCCTGGTAGAA    8022
            ********************************* **************************

LA2093      GTCAAGAATTACTTCTTTCACCTGCTCTTTTCTTTCAATAAGGTCATTTCCTATCTCCAA    8082
E42         GTCAAGAATTACTTCTTTCACCTGCTCTTTTCTTTCAATAAGGTCATTTCCTATCTCCAA    8099
HEINZ       GTCAAGAATTACTTCTTTCACCTGCTCTTTTCTTTCAATAAGGTCATTTCCTATCTCCAA    8082
            ************************************************************

LA2093      TCTGTCAATGTGGTTGTATCTTTTGTGGGAGTTGGCTATTCTTTGAAAGTATTTTGTGTT    8142
E42         TCTGTCAATGTGGTTGTATCTTTTGTGGGAGTTGGCTATTCTTTGAAAGTATTTTGTGTT    8159
HEINZ       TCTGTCAATGTGGTTGTATCTTTTGTGGGAGTTGGCTATTCTTTGAAAGTATTTTGTGTT    8142
            ************************************************************

LA2093      GCTATCCCACTCTTTTAACCATAGACACCACTCTTTGAAAGTATCTGCCTTTGCTAATGG    8202
E42         GCTATCCCACTCTTTTAACCATAGACACCACTCTTTGAAAGTATCTGCCTTTGCTAATGG    8219
HEINZ       GCTATCCCACTCTTTTAACCATAGACACCACTCTTTGAAAGTATCTGCCTTTGCTAATGG    8202
            ************************************************************

LA2093      TTGAATCTCTAGTTGTCATTGCAAAATTTTGTTCTTTTAGACTTGAGATCAGACCCTTCC    8262
E42         TTGAATCTCTAGTTGTCATTGCAAAATTTTGTTCTTTTAGACTTGAGATCAGACCCTTCC    8279
HEINZ       TTGAATCTCTAGTTGTCATTGCAAAATTTTGTTCTTTTAGACTTGAGATCAGACCCTTCC    8262
            ************************************************************

LA2093      TTCAGTTGCTTTATCTGTCAACAGAAGATCATCCATGGCTTACTGATTCTGGAATTTAGA    8322
E42         TTCAGTTGCTTTATCTGTCAACAGAAGATCATCCATGGCTTACTGATTCTGGAATTTAGA    8339
HEINZ       TTCAGTTGCTTTATCTGTCAACAGAAGATCATCCATGGCTTACTGATTCTGGAATTTAGA    8322
            ************************************************************

LA2093      TTACCAAGGACCTCCTTGTTCCAATTAACAAGGTCTTTCTTCAAAAATTTAATTTCTGG    8382
E42         TTACCAAGGACCTCCTTGTTCCAATTAACAAGGTCTTTCTTCAAAAATTTAATTTCTGG    8399
HEINZ       TTACCAAGGACCTCCTTGTTCCAATTAACAAGGTCTTTCTTCAAAAATTTAATTTCTGG    8382
            ************************************************************

LA2093      GAGAGTATAAAGTCGGGGAGCCATTTATTATATAACCTTGCCACCATTTTTCGACCATAT    8442
E42         GAGAGTATAAAGTCGGGGAGCCATTTATTATATAACCTTGCCACCATTTTTCGACCATAT    8459
HEINZ       GAGAGTATAAAGTCGGGGAGCCATTTATTATATAACCTTGCCACCATTTTTCGACCATAT    8442
            ************************************************************

LA2093      CATGAAAACCTTCAGGCTACAGCCACATGTTCTCGAATTTGAAGT---ACGGATCAGAAG    8499
E42         CATGAAAACCTTCATGCTACAGCCACATGTTCTCGAATTTGAAGTAGGACGGATCAGAAG    8519
HEINZ       CATGAAAACCTTCATGCTACAGCCACATGTTCTCGAATTTGAAGT---ACGGATCAGAAG    8499
            ************** ******************************   ************

LA2093      TCCAATCACCACTCTCTAGGGCTAAGGGACTGGTCGAAGAGAACCCTTGGTAGGGCCACC    8559
E42         TCCAATCACCACTCTCTAGGGCTAAGGGACTGGTCGAAGAGAACCCTTGGTAGGGCCACC    8579
HEINZ       TCCAATCACCACTCTCTAGGGCTAAGGGACTGGTCGAAGAGAACCCTTGGTAGGGCCACC    8559
            ************************************************************

LA2093      TGCTTCACAACTCGAAATATTTCATTTCATTCAGTGGAAATTAAGAACCTATCAATTCTG    8619
E42         TGCTTCACAACTCGAAATATTTCATTTCATTCAGTGGAAATTAAGAACCTATCAATTCTG    8639
```

```
HEINZ      TGCTTCACAACTCGAAATATTTCATTTCATTCAGTGGAAATTAAGAACCTATCAATTCTG    8619
           ************************************************************

LA2093     GAAGCCTGCAGAAATTCCTCTCCCCTTCTCCATGTATAAGTTGCACCATGTAAGGAAAGA    8679
E42        GAAGCCTGCAGAAATTCCTCTCCCCTTCTCCATGTATAAGTTGCACCATGTAAGGAAAGA    8699
HEINZ      GAAGCCTGCAGAAATTCCTCTCCCCTTCTCCATGTATAAGTTGCACCATGTAAGGAAAGA    8679
           ************************************************************

LA2093     TCAATAAGACCCATGTCTTGAATAAATACAGACAAGTCCTTCATGTCCTGGGATCTCCTT    8739
E42        TCAATAAGACCCATGTCTTGAATAAATACAGACAAGTCCTTCATGTCCTGGGATCTCCTT    8759
HEINZ      TCAATAAGACCCATGTCTTGAATAAATACAGACAAGTCCTTCATGTCCTGGGATCTCCTT    8739
           ************************************************************

LA2093     ATACAATTGTGCCTCTCATGTTCATATCTACATACATTAAAGGCACCGCCTATGACCCAA    8799
E42        ATACAATTGTGCCTCTCATGTTCATATCTACATACATTAAAGGCACCGCCTATGACCCAA    8819
HEINZ      ATACAATTGTGCCTCTCATGTTCATATCTACATACATTAAAGGCACCGCCTATGACCCAA    8799
           ************************************************************

LA2093     CATCCATCCCAAATGCCTCTGACAGCAGCCAACTCATCTCATAGGTCACACCTTTCTAGA    8859
E42        CATCCATCCCAAATGCCTCTGACAGCAGCCAACTCATCTCATAGGTCACACCTTTCTAGA    8879
HEINZ      CATCCATCCCAAATGCCTCTGACAGCAGCCAACTCATCTCATAGGTCACACCTTTCTAGA    8859
           ************************************************************

LA2093     GTTGAGTGAGGTCCGTACACCCCAGAGAAACAGAATCTGAAGTCCACCTGCAAACCCTCT    8919
E42        GTTGAGTGAGGTCCGTACACCCTAGAGAAACAGAATCTGAAGTCCACCTGCAAACCCTCT    8939
HEINZ      GTTGAGTGAGGTCCGTACACCCCAGAGAAACAGAATCTGAAGTCCACCTGCAAACCCTCT    8919
           ********************** *************************************

LA2093     CACATGGCCAAAAGAGTATGTTGCCCTTGGTAAGAGTCTAAGCATTTCCATTCTCTTTTG    8979
E42        CACATGGCCAAAAGAGTATGTTGCCCTTGGTAAGAGTCTAAGCATTTCCATTCTCTTTTG    8999
HEINZ      CACATGGCCAAAAGAGTATGTTGCCCTTGGTAAGAGTCTAAGCATTTCCATTCTCTTTTG    8979
           ************************************************************

LA2093     TCCCACAAAATAATAATGCCACCCCTAGTACCACTTGCTTTCAATTCTACCCATTCGACC    9039
E42        TCCCACAAAATAATAATGCCACCCCTAGTACCACTTGCTTTCAATTCTACCCATTCGACC    9059
HEINZ      TCCCACAAAATAATAATGCCACCCCTAGTACCACTTGCTTTCAATTCTACCCATTCGACC    9039
           ************************************************************

LA2093     CATCTTGATCCCCAAATATGTCTTGCTATTGCCACAGAACAACACTCTGTCTTTTGTTTC    9099
E42        CATCTTGATCCCCAAATATGTCTTGCTATTGCCACAGAACAACACTCTGTCTTTTGTTTC    9119
HEINZ      CATCTTGATCCCCAAATATGTCTTGCTATTGCCACAGAACAACACTCTGTCTTTTGTTTC    9099
           ************************************************************

LA2093     CTGTAAACACAAGATATCAGGCTTCCATTTTTGAATAAGTAGCTTGATGGTGTTTCTTTT    9159
E42        CTGTAAACACAAGATATCAGGCTTCCATTTTTGAATAAGTAGCTTGATGGTGTTTCTTTT    9179
HEINZ      CTGTAAACACAAGATATCAGGCTTCCATTTTTGAATAAGTAGCTTGATGGTGTTTCTTTT    9159
           ************************************************************

LA2093     GCTTATATCAAGCCCCCTTATATTCCAACTGATAAGTTTTGCTTTCATTCCGAGAAAGCC    9219
E42        GCTTATATCAAGCCCCCTTATATTCCAACTGATAAGTTTTGCTTTCATTCCGAGAAAGCC    9239
HEINZ      GCTTATATCAAGCCCCCTTATATTCCAACTGATAAGTTTTGCTTTCATTCCGAGAAAGCC    9219
           ************************************************************

LA2093     TTGTGGGACCTGCCCCTGCTTGACCCTTCTCCATTCTCATAATTAATTGTGCACTGCAAT    9279
E42        TTGTGGGACCTGCCCCTGCTTGACCCTTCTCCATTCTCATAATTAATTGTGCACTGCAAC    9299
HEINZ      TTGTGGGACCTGCCCCTGCTTGACCCTTCTCCATTCTCATAATTAATTGTGCACTGCAAC    9279
           ***********************************************************

LA2093     TTCTTGGCCACAATCTCACCCTTTTCCTTTGATCTTTTCTTTTGTTGCTCTTTCCCCTTT    9339
E42        TTCTTGGCCACAATCTCACCCTTTTCCTTTGATCTTTTCTTTTGTTGCTCTTTCCCCTTT    9359
HEINZ      TTCTTGGCCACAATCTCACCCTTTTCCTTTGATCTTTTCTTTTGTTGCTCTTTCCCCTTT    9339
           ************************************************************

LA2093     GATTTCTGCAGCTGTAAATTCCTCTTTGTTCCTTTCGCAATATCATATCTAAGATTTCGT    9399
E42        GATTTCTGCAGCTGTAAATTCCTCTTTGTTCCTTTCGCAATATCATATCTAAGATTTCGT    9419
HEINZ      GATTTCTGCAGCTGTAAATTCCTCTTTGTTCCTTTCGCAATATCATATCTAAGATTTCGT    9399
           ************************************************************

LA2093     GCTCAAACCCCACTTAATTGACCCCAAAGGCTTTTTACAAGCTTTGGTCATCACTATTTT    9459
E42        GCTCAAACCCCACTTAATTGACCCCAAAGGC--TTTACAAGCTTTGGTCATCACTATTTT    9477
HEINZ      GCTCAAACCCCACTTAATTGACCCCAAAGGCTTTTTACAAGCTTTGGTCATCACTATTTT    9459
           *******************************  ***************************
```

```
LA2093      GGTCCAATGAGAGGTTTCGTTAGCTGTCGGGTGTGAAATAACATGTTCCTCCAAGCTGTC    9519
E42         GGTCCAATGAGAGGTTTCGTTAGCTGTCGGGTGTGAAATAACATGTTCCTCCAAGCTGTC    9537
HEINZ       GGTCCAATGAGAGGTTTCGTTAGCTGTCGGGTGTGAAATAACATGTTCCTCCAAGCTGTC    9519
            ************************************************************

LA2093      AACGGACCAGGGTAGTGACAAAGTATCGCTACTAATTGGGAAGAGAAACCTGAATCTTGA    9579
E42         AACGGACCAGGGTAGTGATAAAGTATCGCTACTAATTGGGAAGAGAAACCTGAATCTTGA    9597
HEINZ       AACGGACCAGGGTAGTGACAAAGTATCGCTACTAATTGGGAAGAGAAACCTGAATCTTGA    9579
            ****************** *****************************************

LA2093      GAACACCCAATAGAGAAATTAGAGTGGATAGGAGGAAGAGAGAGAAGAGATAGAGGTCGC    9639
E42         GAACACCCAATAGAGAAATTAGAGTGGATAGGAGGAAGAGAGAGAAGAGATAGAGGTCGC    9657
HEINZ       GAACACCCAATAGAGAAATTAGAGTGGATAGGAGGAAGAGAGAGAAGAGATAGAGGTCGC    9639
            ************************************************************

LA2093      AAGCATTCGGCTTCGTCGTCTCCACCTCCCTCGCACTTCTCAAGAAAATCAGTTTGTGTT    9699
E42         AAGCATTCGGCTTCGTCGTCTCCACCTCCCTCGCACTTCTCAAGAAAATCAGTTTGTGTT    9717
HEINZ       AAGCATTCGGCTTCGTCGTCTCCACCTCCCTCGCACTTCTCAAGAAAATCAGTTTGTGTT    9699
            ************************************************************

LA2093      TGGGAAGCCAACAACAGCTTTTAAGATAACTTAGCATTCGACTGTCGTTCAGAAAGCTTT    9759
E42         TGGGAAGCCAACAACAGCTTTTAAGATAACTTAGCATTCGACTGTCGTTCAGAAAGCTTT    9777
HEINZ       TGGGAAGCCAACAACAGCTTTTAAGATAACTTAGCATTCGACTGTCGTTCAGAAAGCTTT    9759
            ************************************************************

LA2093      TGTGTCTCTGCCACTGTAGATTGGGCTTGGGCTGGCCTAATAGCCTTCCACTCCTTTTAT    9819
E42         TGTGTCTCTGCCACTGTAGATTGGGCTTGGGCTGGCCTAATAGCCTTCCACTCCTTTTAT    9837
HEINZ       TGTGTCTCTGCCACTGTAGATTGGGCTTGGGCTGGCCTAATAGCCTTCCACTCCTTTTAT    9819
            ************************************************************

LA2093      TGAGATGCAAGTTCCCCTTTTTCCTCCTTGAATAATAAAGATGGTCTGAAAACTTTTGCT    9879
E42         TGAGATGCAAGTTCCCCTTTTTCCTCCTTGAATAATAAAGATGGTCTGAAAACTTTTGCT    9897
HEINZ       TGAGATGCAAGTTCCCCTTTTTCCTCCTTGAATAATAAAGATGGTCTGAAAACTTTTGCT    9879
            ************************************************************

LA2093      GCAACCGGGCCTTTGCCTTAAGTTGGCCCAAATTTGCATTAGAACTTTTAGGCCCAGACC    9939
E42         GCAACCGGGCCTTTGCCTTAAGTTGGCCCAAATTTGCATTAGAACTTTTAGGCCCAGACC    9957
HEINZ       GCAACCGGGCCTTTGCCTTAAGTTGGCCCAAATTTGCATTAGAACTTTTAGGCCCAGACC    9939
            ************************************************************

LA2093      TAGGATCTTCAGCTGATTTTATATTTGGATCAATAGGCTGCTCATTCTTCAAAACATAAG    9999
E42         TAGGATCTTCAGCTGATTTTATATTTGGATCAATAGGCTGCTCATTCTTCAAAACATAAG    10017
HEINZ       TAGGATCTTCAGCTGATTTTATATTTGGATCAATAGGCTGCTCATTCTTCAAAACATAAG    9999
            ************************************************************

LA2093      CTTGGTCTGAAGTCTTCTGCCACAACAGGGCCTTTGCTTTAAGTTGGCCCAAGGTTGTCT    10059
E42         CTTGGTCTGAAGTCTTCTGCCACAACAGGGCCTTTGCTTTAAGTTGGCCCAACGTTGTCT    10077
HEINZ       CTTGGTCTGAAGTCTTCTGCCACAACAGGGCCTTTGCTTTAAGTTGGCCCAAGGTTGTCT    10059
            **************************************************** *******

LA2093      TTTTGGGCCCAACCTAGGGGCATTTTCAAATTTGAATTGATAGACTGCTCAGTTTTCGAG    10119
E42         TTTTGGGCCCAACCTAGGGGCATTTTCAAATTTGAATTGATAGACTGCTCAGTTTTCGAG    10137
HEINZ       TTTTGGGCCCAACCTAGGGGCATTTTCAAATTTGAATTGATAGACTGCTCAGTTTTCGAG    10119
            ************************************************************

LA2093      ACACAAACTTGTTTGGAAAAACCCTGTTCCTCAGAATCTTTGTGTCCTCATCTGCATCAA    10179
E42         ACACAAACTTGTTTGGAAAAACCCTGTTCCTCAGAATCTTTGTGTCCTCATCTGCATCAA    10197
HEINZ       ACACAAACTTGTTTGGAAAAACCCTGTTCCTCAGAATCTTTGTGTCCTCATCTGCATCAA    10179
            ************************************************************

LA2093      TATAACCACTACAAGAGTCCCCGATAAATTTCAGAGACTTTTCCGACCATGTGTGAAGCG    10239
E42         TATAACCACTACAAGAGTCCCCGATAAATTTCAGAGACTTTTCCGACCATGTGTGAAGCG    10257
HEINZ       TATAACCACTACAAGAGTCCCCGATAAATTTCAGAGACTTTTCCGACCATGTGTGAAGCG    10239
            ************************************************************

LA2093      GGATGCAAAAGCCCTAATCCACCTCTGATTTGCTACTAACCCTTCTTTAGAGTTATTTGT    10299
E42         GGATGCAAAAGCCCTAATCCACCTCTGATTTGCTACTAACCCTTCTTTAGAGTTATTTGT    10317
HEINZ       GGATGCAAAAGCCCTAATCCACCTCTGATTTGCTACTAACCCTTCTTTAGAGTTATTTGT    10299
            ************************************************************

LA2093      CCCGATTCTGGAGATCACCACTCAAGAGATAATCTTCTCCCATTCAAAAACCATTCCCCC    10359
```

```
E42          CCCGATTCTGGAGACCACCACTCAAGAGATAATCTTCTCCCATTCAAAAACCATTCCCCC    10377
HEINZ        CCCGATTCTGGAGATCACCACTCAAGAGATAATCTTCTCCCATTCAAAAACCATTCCCCC    10359
             ************** *********************************************

LA2093       ATTATTATCCTCTCCGCTTCTTGCCTAGATGGAGACTTGAACATCATCTGATTATGTGTG    10419
E42          ATTATTATCCTCTCCGCTTCTTGCCTAGATGGAGACTTGAACATCATCTGATTATGTGTG    10437
HEINZ        ATTATTATCCTCTCCGCTTCTTGCCTAGATGGAGACTTGAACATCATCTGATTATGTGTG    10419
             ************************************************************

LA2093       AGCTGTAAGACGTGCAACCCTGCTGTCATCTTCCACCTTTTCAGGGACCACTGATGGATG    10479
E42          AGCTGTAAGACGTGCAACCCTGCTGTCATCTTCCACCTTTTCAGGGACCACTGATGGATG    10497
HEINZ        AGCTGTAAGACGTGCAACCCTGCTGTCATCTTCCACCTTTTCAGGGACGACTGATGGATG    10479
             ************************************************ ***********

LA2093       ACTGTTGGATTTGGACTAGAGCTGTAAGGGTCATTGAAGGTTCCAACTAAGCAACACTGC    10539
E42          ACTGTTGGATTTGGACTAGAGCTGTAAGGGTCATTGAAGGTTCCAACTAAGCAACACTGC    10557
HEINZ        ACTGTTGGATTTGGACTAGAGCTGTAAGGGTCATTGAAGGTTCCAACTAAGCAACACTGC    10539
             ************************************************************

LA2093       AAGTATTCCTATAGATTCTCCAATTCAGAATGAGACTTAGAGCTCATGACCGGCCATTTC    10599
E42          AAGTATTCCTATAGATTCTCCAATTCAGAATGAGACTTAGAGCTCATGACCGGCCATTTC    10617
HEINZ        AAGTATTCCTATAGATTCTCCAATTCAGAATGAGACTTAGAGCTCATGACCGGCCATTTC    10599
             ************************************************************

LA2093       TGGATATTTGCAGCATCCATAAAAGATTTCCCTTGGGTGACAAACTTCCGGAGAGCTGGG    10659
E42          TGGATATTTGCAGCATCCATAAAAGATTTCCCTTGGGTGACAAACTTCCGGAGAGCTGGG    10677
HEINZ        TGGATATTTGCAGCATCCATAAAAGATTTCCCTTGGGTGACAAACTTCCGGAAAGCTGGG    10659
             **************************************************** *******

LA2093       TTAACCAGAGATCTGATAATTTTGTCGGCTAAATCACTCCACCCAACATTGTAATCCATC    10719
E42          TTAACCAGAGATCTGATAATTTTGTCGGCTAAATCACTCCACCCAACATTGTAATCCATC    10737
HEINZ        TTAACCAGAGATCTGATAATTTTGTCGGCTAAATCACTCCACCCAACATTGTAATCCATC    10719
             ************************************************************

LA2093       TCCGGGATGATAATAGCTGATTTTCTTTGGCCCCTAATCTCATAACACTGCATACCCATC    10779
E42          TCCGGGATGATAATAGCTGATTTTCTTTGGCCCCTAATCTCATAACACTGCATACCCATC    10797
HEINZ        TCCGGGATGATAATAGCTGATTTTCTTTGGCCCCTAATCTCATAACACTGCATACCCATC    10779
             ************************************************************

LA2093       TTAAAATGACTTCGTCGATCTTAATCTTACTGGCAAACCGACTACTGAACTCCTTGAACA    10839
E42          TTAAAATGACTTCGTCGATCTTAATCTTACTGGCAAACCGACTACTGAACTCCTTGAACA    10857
HEINZ        TTAAAATGACTTCGTCGATCTTAATCTTACTGGCAAACCGACTACTGAACTCCTTGAACA    10839
             ************************************************************

LA2093       AGAACCACCTAAAAGAACCTTCACTGATATCATTTATTTCAAAAGACTTATCACTGGAAA    10899
E42          AGAACCACCTAAAAGAACCTTCACTGATATCATTTATTTCAAAAGACTTATCACTGGAAA    10917
HEINZ        AGAACCACCTAAAAGAACCTTCACTGATATCATTTATTTCAAAAGACTTATCACTGGAAA    10899
             ************************************************************

LA2093       TGAAAAAGCACACCCTGTCACCCATACTAAGAGATCTTATGATTAAAATTCCACCGCAAA    10959
E42          TGAAAAAGCACACCCTGTCACCCATACTAAGAGATCTTATGATTAAAATTCCACCGCAAA    10977
HEINZ        TGAAAAAGCACACCCTGTCACCCATACTAAGAGATCTTATGATTAAAATTCCACCGCAAA    10959
             ************************************************************

LA2093       GTAAATAATTTAGTCCCCCATGGTAAAAAAGAAGCTAATTCTGTTAGGAGTTGCCGGAAA    11019
E42          GTAAATAATTTAGTCCCCCATGGTAAAAAAGAAGCTAATTCTGTTAGGAGTTGCCGGAAA    11037
HEINZ        GTAAATAATTTAGTCCCCCATGGTAAAAAAGAAGCTAATTCTGTTAGGAGTTGCCGGAAA    11019
             ************************************************************

LA2093       AGGATCAAACAGAGTACGAGAGAGAGCCTAGGACAGATTCCCCTTGGGAACTGTGCCTGG    11079
E42          AGGATCAAACAGAGTACGAGAGAGAGCCTAGGACAGATTCCCCTTGGGAACTGTGCCTGG    11097
HEINZ        AGGATCAAACAGAGTACGAGAGAGAGCCTAGGACAGATTCCCCTTGGGAACTGTGCCTGG    11079
             ************************************************************

LA2093       GAGCATATACGACTCTTAGGTGGAATCTTACATAACAGTTGCACAAATTTTAAATTCTGA    11139
E42          GAGCATATACGACTCTTAGGTGGAATCTTACATAACAGTTGCACAAATTTTAAATTCTGA    11157
HEINZ        GAGCATATACGACTCTTAGGTGGAATCTTACATAACAGTTGCACAAATTTTAAATTCTGA    11139
             ************************************************************

LA2093       CAAAGAAGAGAAGCAACATCTAACTTTCAAGAAATACAATTCTGTGCATCTGTGAGTTAA    11199
E42          CAAAGAAGAGAAGCAACATCTAACTTTCAAGAAATACAATTCTGTGCATCTGTGAGTTAA    11217
HEINZ        CAAAGAAGAGAAGCAACATCTAACTTTCAAGAAATACAATTCTGTGCATCTGTGAGTTAA    11199
```

```
            ************************************************************

LA2093      CTAGCTAAGATAAAAATTTCAACACTCCGCCACTCAATTCTATTCTATATAATAGAATAT    11259
E42         CTAGCTAAGATAAAAATTTCAACACTCCGCCACTCAATTCTATTCTATATGATAGAATAT    11277
HEINZ       CTAGCTAAGATAAAAATTTCAACACTCCGCCACTCAATTCTATTCTATATAATAGAATAT    11259
            ************************************************** *********

LA2093      GAATTGTAACACTTCATTTGCTAACAAACTACAGAAAACCCCATTGTTTGAAGTCACTAG    11319
E42         GAATTGTAACACTTCATTTGCTAACAAACTACAGAAAACCCCATTCTTTGAAGTCACTAG    11337
HEINZ       GAATTGTAACACTTCATTTGCTAACAAACTACAGAAAACCCCATTGTTTGAAGTCACTAG    11319
            ********************************************* **************

LA2093      ATGGCGTACCCTGCTGTTATTCAGGGTGAAACAACCTTGAATGAGTATAAGGAAAAGGAA    11379
E42         ATGGCGTACCCTGCTGTTATTCAGGGTGAAACAACCTTGAATGAGTATAAGGAAAAGGAA    11397
HEINZ       ATGGCGTACCCTGCTGTTATTCAGGGTGAAACAACCTTGAATGAGTATAAGGAAAAGGAA    11379
            ************************************************************

LA2093      CATCCAATTTTCACAATAAAATTCAATTGAATTCAGTAAAGGATGCAGTAGCCCACAATA    11439
E42         CATCCAATTTTCACAATAAAATTCAATTGAATTCAGTAAAGGATGCAGTAGCCCACAATA    11457
HEINZ       CATCCAATTTTCACAATAAAATTCAATTGAATTCAGTAAAGGATGCAGTAGCCCACAATA    11439
            ************************************************************

LA2093      TAAACACGACATTAGACTTGGCAGAGAATAGAAAAGAAGAATAAACCTCCAAATCTCGCA    11499
E42         TAAACACGACATTAGACTTGGCAGAGAATAGAAAAGAAGAATAAACCTCCAAATCTCGCA    11517
HEINZ       TAAACACGACATTAGACTTGGCAGAGAATAGAAAAGAAGAATAAACCTCCAAATCTCGCA    11499
            ************************************************************

LA2093      GAACTTTCAGGTTGCTCACGTGCATAAGTAAGATTATAGAACTCCTTTGTTTGGTGATTA    11559
E42         GAACTTTCAGGTTGCTCACGTGCATAAGTAAGATTATAGAACTCCTTTGTTTGGTGATTA    11577
HEINZ       GAACTTTCAGGTTGCTCACGTGCATAAGTAAGATTATAGAACTCCTTTGTTTGGTGATTA    11559
            ************************************************************

LA2093      TAAAGATAACCTGCATAACATATCATAAAGCATCAGTACAGTAAAAGGCAACAAGTGAAA    11619
E42         TAAAGATAACCTGCATAACATATCATAAAGCATCAGTACAGTAAAAGGCAACAAGTGAAA    11637
HEINZ       TAAAGATAACCTGCATAACATATCATAAAGCATCAGTACAGTAAAAGGCAACAAGTGAAA    11619
            ************************************************************

LA2093      GTAAACTATGCCAAAGAGACTTGAATACTAGAATCTTCATAAATAAAGAGGCTATGGGAT    11679
E42         GTAAACTATGCCAAATAGACTTGAATACTAGAATCTTCATAAATAAAGAGGCTATGGGAT    11697
HEINZ       GTAAACTATGCCAAAGAGACTTGAATACTAGAATCTTCATAAATAAAGAGGCTATGGGAT    11679
            *************** ********************************************

LA2093      TCTCCCAACCCAAATATAGAGACACTGACAGAATGCAGAAGTTTAGATCTGCTACACATT    11739
E42         TCTCCCAACCCAAATATAGAGAAACTGACAGAATGCAGAAGTTTAGATCTGCTACACATT    11757
HEINZ       TCTCCCAACCCAAATATAGAGACACTGACAGAATGCAGAAGTTTAGATCTGCTACACATT    11739
            ********************** *************************************

LA2093      TTTTGTGTGGGGGAGTGTGTGTGTGTGTGCGCGCGT----GGGGGGGGGGGGATAACCAA    11795
E42         TTTTGTGTGGGGGAGTGTGTGTGTGTGTGCGCGCGTGGGGGGGGGGGGGGGGATAACCAA    11817
HEINZ       TTTTGTGTGGGGGAGTGTGTGTGTGTGTGCGCGCGTGGGGGGGGGGGGGGGGATAACCAA    11799
            ************************************    ********************

LA2093      GCACTAAGAAATAATATATCAGTTTTGCAAATGAAGGTATCACAAGAAGATGCCTGAAAT    11855
E42         GCACTAAGAAATAATATATCAGTTTTGCAAATGAAGGTATCATAAGAAGATGCCTGAAAT    11877
HEINZ       GCACTAAGAAATAATATATCAGTTTTGCAAATGAAGGTATCATAAGAAGATGCCTGAAAT    11859
            ****************************************** *****************

LA2093      TAACACAAAAATGTAATAAGTACGAGAGTTTATTGGACCTTATAGTTATTCGAAAAAATA    11915
E42         TAACACAAAAATGTAATAAGTACGAGAGTTTATTGGACCTTATAGTTATTCGAAAAAATA    11937
HEINZ       TAACACAAAAATGTAATAAGTACGAGAGTTTATTGGACCTTATAGTTATTCGAAAAAATA    11919
            ************************************************************

LA2093      CAAAAATGAAGGTAAACAAAACAGAAGCTGATGTTAAGTAAATTAACCATGCCTACTGTT    11975
E42         CAAAAATGAAGGTAAACAAAACAGAAGCTGATGTTAAGTAAATTAACCATGCCTACTGTT    11997
HEINZ       CAAAAATGAAGGTAAACAAAACAGAAGCTGATGTTAAGTAAATTAACCATGCCTACTGTT    11979
            ************************************************************

LA2093      TCTATCGATTTTTCCGTTATGTTAGGGTGACATGGTTTTTAATTTTGGAAAACCATCCAG    12035
E42         TCTATCGATTTTTCCGTTATGTTAGGGTGACATGGTTTTTAATTTTGGAAAACCATCCAG    12057
HEINZ       TCTATCGATTTTTCCGTTATGTTAGGGTGACATGGTTTTTAATTTTGGAAAACCATCCAG    12039
            ************************************************************
```

```
LA2093      AATTTGGTTTTGGCATTGAGAACAAAAATCAGACAAAATGCTGTACTTTAAATAAACTAT    12095
E42         AATTTGGTTTTGGCATTGAGAACAAAAATCAGACAAAATGCTGTACTTTAAATAAACTAT    12117
HEINZ       AATTTGGTTTTGGCATTGAGAACAAAAATCAGACAAAATGCTGTACTTTAAATAAACTAT    12099
            ************************************************************

LA2093      ACATTTCTGCCTAACTCCAGCTTTAGAATCTCTCCTGA----------------GAACAT    12139
E42         ACATTTCTGCCTAACTCCAGCTTTAGAATCTCTCCTGACCCTGTTTAGTACATGAACAT    12177
HEINZ       ACATTTCTGCCTAACTCCAGCTTTAGAATCTCTCCTGA----------------GAACAT    12143
            **************************************                ******

LA2093      CTAAATCATCATACTCTACCAAATTTACTGTGTACAACATGGACTTCTAGATCTTCATAT    12199
E42         CTAAATCATCATACTCTACCAAATTTACTGTGTACAACATGGACTTCTAGATCTTCATAT    12237
HEINZ       CTAAATCATCATACTCTACCAAATTTACTGTGTACAACATGGACTTCTAGATCTTCATAT    12203
            ************************************************************

LA2093      TCTATGTAATTTGCTAATTTACAGGCTTCAGTTTTTAAGATTTGCTTTCCTAATTGTCAG    12259
E42         TCTATGTAATTTGCTAATTTACAGGCTTCAGTTTTTAAGATTTGCTTTCCTAATTGTCAG    12297
HEINZ       TCTATGTAATTTGCTAATTTACAGGCTTCAGTTTTTAAGATTTGCTTTCCTAATTGTCAG    12263
            ************************************************************

LA2093      CTGGAAGAAACTAAACTGTTGTGGCCAATTTGCCTTTTACTTTAATCTACATAAAATACC    12319
E42         CTGGAAGAAACTAAACTGTTGTGGCCAATTTGCCTTTTACTTTAATCTACATAAAATACC    12357
HEINZ       CTGGAAGAAACTAAACTGTTGTGGCCAATTTGCCTTTTACTTTAATCTACATAAAATACC    12323
            ************************************************************

LA2093      TGGAATCATTTCTTTAGTACTTGGACTCTTAATATTGTTATTTTCCATCATGTTCTCTTT    12379
E42         TGGAATCATTTCTTTAGTACTTGGACTCTTAATATTGTTATTTTCCATCATGTTCTCTTT    12417
HEINZ       TGGAATCATTTCTTTAGTACTTGGACTCTTAATATTGTTATTTTCCATCATGTTCTCTTT    12383
            ************************************************************

LA2093      ACCATGTCAACTAATATAACAGTAACAACTCTTCAATCCCAAACTAGTTGGGATCAAATC    12439
E42         ACCATGTCAACTAATATAACAGTAACAACTCTTCAATCCCAAACTAGTTGGGATCAAGTC    12477
HEINZ       ACCATGTCAACTAATATAACAGTAACAACTCTTCAATCCCAAACTAGTTGGGATCAAATC    12443
            ********************************************************* **

LA2093      CATGTTAACTTTTCAAAGGAGAAAAAGTTTTGTACAAATAGATACTCCTTACACCCCATT    12499
E42         CATGTTAACTTTTCAAAGGAGAAAAAGTTTTGTACAAATAGATACTCCTTACACCCCATT    12537
HEINZ       CATGTTAACTTTTCAAAGGAGAAAAAGTTTTGTACAAATAGATACTCCTTACACCCCATT    12503
            ************************************************************

LA2093      TTATGGGGCACCATTTGACTTGACACGGTATTAAAGAAAAAAAAAGACCTTTAAACTTGT    12559
E42         TTATGGGGCACCATTTGACTTGACACGGTATTAAAGAAAAAAAAAGACCTTTAAACTTGT    12597
HEINZ       TTATGGGGCACCATTTGACTTGACACGGTATTAAAGAAAAAAAAAGACCTTTAAACTTGT    12563
            ************************************************************

LA2093      AGTCTAAAACAATCCTTAGATATTTGTGTGGCATAATCCGTTCATTAAGGGTAAAAGAGG    12619
E42         AGTCTAAAACAATCCTTAGATATTTGTGTGGCGTAATCAGTTCATTAAGGGTAAAAGAGG    12657
HEINZ       AGTCTAAAACAATCCTTAGATATTTGTGTGGCGTAATCAGTTCATTAAGGGTAAAAGAGG    12623
            ******************************** ***** *********************

LA2093      AAATTTTAAAGATAAAATCCTTTCTAATTATAGTAAAGTGACATTCTTTTTGGGACGGAC    12679
E42         AAATTTTAAAAATAAAATCCTTTCTAATTATAGTAAAGTGACATTCTTTTTGGGACGGAC    12717
HEINZ       AAATTTTAAAGATAAAATCCTTTCTAATTATAGTAAAGTGACATTCTTTTTGGGACGGAC    12683
            ********** *************************************************

LA2093      TAAAAAGGGAAGTGTGACACATAAAATGGGACGGGGGGAGTATTTTTTTTCAAACATGTG    12739
E42         TAAAAAGAAAAGTGTGACACATAAAATGGGACGGGGGGAGTATTTTTTTTCAAACATGTG    12777
HEINZ       TAAAAAGGAAAGTGTGACACATAAAATGGGACGGGGGGAGTATTTTTTTTCAAACATGTG    12743
            *******  ***************************************************

LA2093      GAGTTCAAAAAACGTTCTACAGAGTAGTGGTTAGACCGACTTTGTAGAATGACGCGGAGT    12799
E42         GAGTTCAAAAAACGTTCTACAGAGTAGTGGTTAGACCGACTTTGTAGAATGACACGGAGT    12837
HEINZ       GAGTTCAAAAAACGTTCTACAGAGTAGTGGTTAGACCGACTTTGTAGAATGACGCGGAGT    12803
            ***************************************************** ******

LA2093      TTTGGCCAGTCAAAAACTCTCATGTTTCGAAGATGCAAGTTGTGGAAATGAGGATGTTGA    12859
E42         TTTGGCCAGTCAAAAACTCTCATGTTTCGAAGATGCAAGTTGTGGAAATGAGGATGTTGA    12897
HEINZ       TTTGGCCAGTCAAAAACTCTCATGTTTCGAAGATGCAAGTTGTGGAAATGAGGATGTTGA    12863
            ************************************************************

LA2093      GATAGAAGTGGGCATACTATGAGCGTTAAGATTAGGAATTGACGATATATGGGACAAGGT    12919
E42         GATAGAAGTGGGCATACTATGAGCGTTAAGATTAGGAATTGACGATATATGGGACAAGGT    12957
```

```
HEINZ        GATAGAAGTGGGCATACTATGAGCGTTAAGATTAGGAATTGACGATATATGGGACAAGGT    12923
             ************************************************************

LA2093       GGGAGTGGCCACAGTGGTGGATAAGAAGAGGAAAGCGAGATTGATATGGTTTGGGCATGT    12979
E42          GGGAGTGGCCACAGTGGTGGATAAGAAGAGGAAAGCGAGATTGATATGGTTTGGGCATGT    13017
HEINZ        GGGAGTGGCCACAGTGGTGGATAAGAAGAGGAAAGCGAGATTGATATGGTTTGGGCATGT    12983
             ************************************************************

LA2093       GAAGAGGAGATCATGCAAATATAAAAGAAATGCTATCTCTTGGTAAACACACCTTTCTCT    13039
E42          GAAGAGGAGATCATGCAAATATAAAAGAAATGTTATCTCTTGGTAAACACACCTTTCTCT    13077
HEINZ        GAAGAGGAGATCATGCAAATATAAAAGAAATGCTATCTCTTGGTAAACACACCTTTCTCT    13043
             ******************************** ***************************

LA2093       CTCTCGTTTCCTCCCTTTTTTTCTTTCTCAGTTCATAACTACAGCGAGTGTTTTACCGAT    13099
E42          CTCTCGTTTCCTCCCTTTTTTTCTTTCTCAGTTCATAACTACAGCGAGTGTTTTACCGAT    13137
HEINZ        CTCTCGTTTCCTCCCTTTTTTTCTTTCTCAGTTCATAACTACAGCGAGTGTTTTACCGAT    13103
             ************************************************************

LA2093       GGAATTCCTACTTTGCCCTAAGTCATGGATTAGAGATTCTCTTTTAGCACGAGTAGGAAG    13159
E42          GGAATTCCTACTTTGCCCTAAGTCATGGATTAGAGATTCTCTTTTAGCACGAGTAGGAAG    13197
HEINZ        GGAATTCCTACTTTGCCCTAAGTCATGGATTAGAGATTCTCTTTTAGCACGAGTAGGAAG    13163
             ************************************************************

LA2093       TTTTTCAATTTAAGCATTTTAGTTAAAAGATGGTTCCTTTTAACAGAAGTAGTAGAAGCT    13219
E42          TTTTTCAATTTAAGCATTTTAGTTAAAAGATGGTTCCTTTTAACAGAAGTAGTAGAAGCT    13257
HEINZ        TTTTTCAATTTAAGCATTTTAGTTAAAAGATGGTTCCTTTTAACAGAAGTAGTAGAAGCT    13223
             ************************************************************

LA2093       TCACTAGCAACATCAAAATCGATGGCAATGATCCCCAATTGATCTGCAAAGCCTTGAAAC    13279
E42          TCACTAGCAACATCAAAATCGATGGCAATGATCCCCAATTGATCTGCAAAGCCTTGAAAC    13317
HEINZ        TCACTAGCAACATCAAAATCGATGGCAATGATCCCCAATTGATCTGCAAAGCCTTGAAAC    13283
             ************************************************************

LA2093       ATGCTTCTTTACAACAAGGCAACAAGTGTAGAAGGTGGGAAGATAAGTCCAAGCCTACAC    13339
E42          ATGCTTCTTTACAACAAGGCAACAAGTGTAGAAGGTGGGAAGATAAGTCCAAGCCTACAC    13377
HEINZ        ATGCTTCTTTACAACAAGGCAACAAGTGTAGAAGGTGGGAAGATAAGTCCAAGCCTACAC    13343
             ************************************************************

LA2093       TTTAAGGTTGATTCATGAGAATAGAAAGGGATAGGCTTAAGTCATCTGCGACCTTTAAAG    13399
E42          TTTAAGGTCGATTCATGAGAATAGAAAGGGATAGGCTTAAGTCATCTGCGACCTTTAAAG    13437
HEINZ        TTTAAGGTTGATTCATGAGAATAGAAAGGGATAGGCTTAAGTCATCTGCGACCTTTAAAG    13403
             ******** ***************************************************

LA2093       TTGTTCGCATATTTCTCTTGGATACCTGATCTCAGGTTTGTTCCAATTGAACACCTTTAT    13459
E42          TTGTTCGCATATTTCTCTTGGATACCTGATCTCAGGTTTGTTCCAATTGAACACCTTTAT    13497
HEINZ        TTGTTCGCATATTTCTCTTGGATACCTGATCTCAGGTTTGTTCCAATTGAACACCTTTAT    13463
             ************************************************************

LA2093       TAGTCAAAAAAAATAGCTATCAAACATTTTTTGACAATAAACAAAAATTAGAGATTGTGT    13519
E42          TAGTCAAAAAAATTACCTATCAAACATTTTTTGACAATAAACAAAAATTAGAGATTGTGT    13557
HEINZ        TAGTCAAAAAAAATACCTATCAAACATTTTTTGACAATAAACAAAAATTAGAGATTGTGT    13523
             ************ ** ********************************************

LA2093       GTCATACACTCGCCACTGACGTGGCATAATAACTCATGAAAAAATGACACATGGCATTGG    13579
E42          GTCATACACTCGCCACTGACGTGGCATAATAACTCATGAAAAAATGACACATGGCATTGG    13617
HEINZ        GTCATACACTCGCCACTGACGTGGCATAATAACTCATGAAAAAATGACACATGGCATTGG    13583
             ************************************************************

LA2093       GGCCATAATAACAATTATTAAAAATAAATTTGAAGTACTCTAAAAGTTATGTTTTCAGCA    13639
E42          GCCCATAATAACAATTAGTAAAAATAAATTTGAAGTACTCCAAAAGTTATGTTTTCAGCA    13677
HEINZ        GCCCATAATAACAATTAGTAAAAATAAATTTGAAGTACTCTAAAAGTTATGTTTTCAGCA    13643
             * *************** *********************** *******************

LA2093       AAAAAAATTAAAACATTCCCTTTTCTTCTTTGCAGAAGCCAAGAACACCCCACCCACCCT    13699
E42          AAAAAAATAAAAACATTCCCTTTTCTTCTTTGCAGAAGCCAAGAACACCCCACCCACCCT    13737
HEINZ        AAAAAAATTAAAACATTCCCTTTTCTTCTTTGCAGAAGCCAAGAACACCCCACCCACCCT    13703
             ******** ***************************************************

LA2093       CGCGGCCATTCTTCCCCATTTTTTTCTGCCAAAACATACAATCTTCAACGGTTAAAACCA    13759
E42          CGCGGCCATTCTTCCCCATTTTTTTCTGCCAAAACATACAATCTTCAACGGTTAAAACCA    13797
HEINZ        CGCGGCCATTCTTCCCCATTTTTTTCTGCCAAAACATACAATCTTCAACGGTTAAAACCA    13763
             ************************************************************
```

```
LA2093      TGTGCACTTCGATATCATCCTCACTCCACCTTTTTGAATTCTTTAACGATTTCAAAGAAA     13819
E42         TGTGCACTTCGATATCATCCTCACTCCACCTTTTTGAATTCTTTAACGATTTCAAAGAAA     13857
HEINZ       TGTGCACTTCGATATCATCCTCACTCCACCTTTTTGAATTCTTTAACGATTTCAAAGAAA     13823
            ************************************************************

LA2093      ATAATTTCATCGGTATCAGTTTTATCGGAAAAAAATGGAACTTCGGCAGTATTCGCCGGGT     13879
E42         ATAATTTCATCGGTATCAGTTTTATCGG-AAAAATGGAACTTCGGCAGTATTCGCCGGGT     13916
HEINZ       ATAATTTCATCGGTATCAGTTTTATCGGAAAAAAATGGAACTTCGGCAGTATTCGCCGGGT     13883
            **************************** *******************************

LA2093      AAAACCATGTGCACTCCGATAATCATCCTCACTCCACCTTTTTGAATTTTTAACGATTTC     13939
E42         AAAACCATGTGCACTCCGATAATCATCCTCACTCCACCTTTTTGAATTTTTAACGATTTC     13976
HEINZ       AAAACCATGTGCACTCCGATAATCATCCTCACTCCACCTTTTTGAATTTTTAACGATTTC     13943
            ************************************************************

LA2093      AAAGAAAATAATTTCATTCGTATTAGTTTTATCGGAAAAAAATGGAGTTTCGTCGGAATTA     13999
E42         AAAGAAAATAATTTCATTCGTATTAGTTTTATCGGAAAAAAATGGAGTTTCGTCGGAATTA     14036
HEINZ       AAAGAAAATAATTTCATTCGTATTAGTTTTATCGGAAAAAAATGGAGTTTCGTCGGAATTA     14003
            ************************************************************

LA2093      TTTTCAACTCAAATTGATTTTTAATTTTGATGAAATTCTCTTCCACAACTCAAATGAAAA     14059
E42         TTTTCAACTCAAATTGATTTTTAATTTTGATGAAATTCTCTTCCACAACTCAAATGAAAA     14096
HEINZ       TTTTCAACTCAAATTGATTTTTAATTTTGATGAAATTCTCTTCCACAACTCAAATGAAAA     14063
            ************************************************************

LA2093      --TTTATCAATAAACTCTTCTGATTTTTTGTGTTTTGACTGATTATTTATTTAACTGATG     14117
E42         TTTTTATCAATAAACTCTTCTGATTTTTTGTGTTTTGACTGATTATTTATTTAACTGATG     14156
HEINZ       --TTTATCAATAAACTCTTCTGATTTTTTGTGTTTTGACTGATTATTTATTTAACTGATG     14121
              **********************************************************

LA2093      TTTTTCCAATGAAGATAACAACAATGATGATATTGATGATGGTTGAATGATGCTAGAAAG     14177
E42         TTTTTCCAATGAAGATAACAACAATGATGATATTGATGATGGTTGAATGATGCTAGAAAG     14216
HEINZ       TTTTTCCAATGAAGATAACAACAATGATGATATTGATGATGGTTGAATGATGCTAGAAAG     14181
            ************************************************************

LA2093      AAAGAATGATGAATTATTTGAGAAAGGGGTGTGTCGATGGTGGGAAAGGAATGGCGGACG     14237
E42         AAAGAATGATGAATTATTTGAGAAAGGGGTGTGTCGATGGTGCGAAAGGAATGGCGGACG     14276
HEINZ       AAAGAATGATGAATTATTTGAGAAAGGGGTGTGTCGATGGTGGGAAAGGAATGGCGGACG     14241
            ****************************************** *****************

LA2093      AGGGTTCTAGGTATCTTATTGAAAAGAGAAGTCTCACTGGGGAAAAACTGCCAAGAATTT     14297
E42         AGGGTTCTAGGTATCTTATTGAAAAGAGAAGTCCCACTGGGGAAAAACTGCCAAGAATTT     14336
HEINZ       AGGGTTCTAGGTATCTTATTGAAAAGAGAAGTCTCACTGGGGAAAAACTGCCAAGAATTT     14301
            ********************************* **************************

LA2093      TTTTTAAAAAAAATTATGTTCATACTTCATAATATGATTTTGTTTTTGTTGACATGTTCA     14357
E42         TTTTAAAAAAAAATTATGTTCATACTTCATAATATGATTTTGTTTTTGTTGACATGTTCA     14396
HEINZ       TTTTTTAAAAAAATTATGTTCATACTTCATAATATGATTTTGTTTTTGTTGACATGTTCA     14361
            ****  ******************************************************

LA2093      TTTTTTATTGGTCCATAAAAAGATTTATTATACTTATAATGTCATGCCACCAAAAAGTAT     14417
E42         TTTTTTATTGGTCCATAAAAAGATTTATTATACTTATAATGTCATGCCACCAAAAAGTAT     14456
HEINZ       TTTTTTATTGGTCCATAAAAAGATTTATTATACTTATAATGTCATGCCACCAAAAAGTAT     14421
            ************************************************************

LA2093      TAAATAGATACATGTATTGAAGGGTAGGGTGTTCAATAGGTACAACCCCTAGTTGAGGTG     14477
E42         TAAATAGATACATGTATTGAAGGGTAGGGTGTTCAATAGGTACAACCCCTAGTTGAGGTG     14516
HEINZ       TAAATAGATACATGTATTGAAGGGTAGGGTGTTCAATAGGTACAACCCCTAGTTGAGGTG     14481
            ************************************************************

LA2093      TCTAAGTGAATTACACGAACAACTTTAGCGGGCAGCAGATGACTTAAGCCGAAAGGGATA     14537
E42         TCTAAGTGAATTACACGAACAACTTTAGCGGGCAGCAGATGACTTAAGCCGAAAGGGATA     14576
HEINZ       TCTAAGTGAATTACACGAACAACTTTAGCGGGCAGCAGATGACTTAAGCCGAAAGGGATA     14541
            ************************************************************

LA2093      TAAAGGATGCAATCATCATCCCAAAAGAAGCTATAGCAAAGGGTGGACCAACACAACAGT     14597
E42         TAAAGGATGCAATCATCATCCCAAAAGAAGCTATAGCAAAGGGTGGACCAACACAACAGT     14636
HEINZ       TAAAGGATGCAATCATCATCCCAAAAGAAGCTATAGCAAAGGGTGGACCAACACAACAGT     14601
            ************************************************************

LA2093      AAAGATTCAAAATTCTTTGGGTCCTTGCCTAAACCCAAGGCTCCTTCTTTATGCTGATAA     14657
```

```
E42           AAAGATTCAGAATTCTTTGGGTCCTTGCCTAAACCCAAGGCTCCTTCTTTATGCTGATAA     14696
HEINZ         AAAGATTCAAAATTCTTTGGGTCCTTGCCTAAACCCAAGGCTCCTTCTTTATGCTGATAA     14661
              ********* **************************************************

LA2093        GTCGAGATCCTCTAAGGAGGCCGCAAGAATATCAAACTGCCAATCACAGCCAATACTGAG     14717
E42           GTCGAGATCCTCTAAGGAGGCCGCAAGAATATCAAACTGCCAATCACAGCCAATACTGAG     14756
HEINZ         GTCGAGATCCTCTAAGGAGGCCGCAAGAATATCAAACTGCCAATCACAGCCAATACTGAG     14721
              ************************************************************

LA2093        GTCCGGTCTAGGAACATAGAGAATTATCTTTCAAATATTAGCCCTAAAAAGGATGTCATT     14777
E42           GTCCGGTCTAGGAACATAGAGAATTATCTTTCAAATATTAGCCCTAAAAAGGATGTCATT     14816
HEINZ         GTCCGGTCTAGGAACATAGAGAATTATCTTTCAAATATTAGCCCTAAAAAGGATGTCATT     14781
              ************************************************************

LA2093        CAGGGTTGGTTCGTCGGGAAATGGTAAGTCACTGTAGGGCTCGAAGTCACCCCTATCAAG     14837
E42           CAGGGTTGGTTCGTTGGGAAATGTTAAGTCACTGTAGGGCTCGAAGTCACCCCTATCAAG     14876
HEINZ         CAGGGTTGGTTCGTCGGGAAATGGTAAGTCACTGTAGGGCTCGAAGTCACCCCTATCAAG     14841
              ************** ******** ************************************

LA2093        AACAACCAATTTTAATTTGAGCTCCCCTCTCAGATTGAAGCAGCAAGGATAAAGGTCGGT     14897
E42           AACAACCAATTTTAATTTGAGCTCCCCTCTCAGATTGAAGCAGCAAGGATAAAGGTCGGT     14936
HEINZ         AACAACCAATTTTAATTTGAGCTCCCCTCTCAGATTGAAGCAGCAAGGATAAAGGTCGGT     14901
              ************************************************************

LA2093        GAATGGTTTAGGAAGAGAGAACAAGAAACATTGAACCTATCGTTATTGGGCTCGAATTTG     14957
E42           GAATGGTTTAGGAAGAGAGAACAAGAAACATTGAACCTATCGTTATTGGGCTCGAATTTG     14996
HEINZ         GAATGGTTTAGGAAGAGAGAACAAGAAACATTGAACCTATCGTTATTGGGCTCGAATTTG     14961
              ************************************************************

LA2093        TGTCAGGAACAACATACAAGAAATTCTGAGGCAATTAGAAATTCTAAGAGAGATGGGGGT     15017
E42           TGTCAGGAACAACATACAAGAAATTCTGAGGCAATTAGAAATTCTAAGAGAGATGGGGGT     15056
HEINZ         TGTCAGGAACAACATACAAGAAATTCTGAGGCAATTAGAAATTCTAAGAGAGATGGGGGT     15021
              ************************************************************

LA2093        ATGAAATCATGATTCTAGAAGATTTGCACACAAAAATCAGGCTAGCCGGAAGAAACAATG     15077
E42           ATGAAATCATGATTCTAGAAGATTTGCACACAAAAATCAGGCTAGCCGGAAGAAACAATG     15116
HEINZ         ATGAAATCATGATTCTAGAAGATTTGCACACAAAAATCAGGCTAGCCGGAAGAAACAATG     15081
              ************************************************************

LA2093        GCGGCAACGAAGGTTAGATGTTCGAAGTGGATGACACATCAGAGCACATCATTAGAGAAA     15137
E42           GCGGCAACGAAGGTTAGATGTTCGAAGTGGATGACACATCAGAGCACATCATTAGAGAAA     15176
HEINZ         GCGGCAACGAAGGTTAGATGTTCGAAGTGGATGACACATCAGAGCACATCATTAGAGAAA     15141
              ************************************************************

LA2093        GGACAAGTTGGCAGCTTTAAATTGAAAGTAGAAGCACAGCTTTGTCCAGCAGATAACGGC     15197
E42           GGACAAGTTGGCAGCTTTAAATTGAAAGTAGAAGCACAGCTTTGTCCAGCAGATAACGGC     15236
HEINZ         GGACAAGTTGGCAGCTTTAAATTGAAAGTAGAAGCACAGCTTTGTCCAGCAGATAACGGC     15201
              ************************************************************

LA2093        GCAAGGGTAAAAGCAGATAATTAGTAAGAATGGAGGTGGGCCATTCTTGGGCCTAGAGGC     15257
E42           GCAAGGGTAAAAGCAGATAATTAGTAAGAATGGAGGTGGGCCATTCTTGGGCCTAGAGGT     15296
HEINZ         GCAAGGGTAAAAGCAGATAATTAGTAAGAATGGAGGTGGGCCATTCTTGGGCCTAGAGGC     15261
              *********************************************************** 

LA2093        TGATAAAGAGTGCTATTTTTAAGGCCCAAGAAGGAGAAAAGGCAAAGCAAAAGTCTCAAA     15317
E42           TGATAAAGTGTGCTATTTTTAAGGCCCAAGAAGGAGAAAAGGCAAAGCAAAAGTCTCAAA     15356
HEINZ         TGATAAAGAGTGCTATTTTTAAGGCCCAAGAAGGAGAAAAGGCAAAGCAAAAGTCTCAAA     15321
              ******** ***************************************************

LA2093        AGAGGTCAGTCTCTTACTTCAAAAGTCACAAATTTAATGTGCATGCATCTGAGATACAAG     15377
E42           AGAGGTCAGTCTCTTACTTCAAAAGTCACAAATTTAATGTGCATGCATCTGAGATACAAG     15416
HEINZ         AGAGGTCAGTCTCTTACTTCAAAAGTCACAAATTTAATGTGCATGCATCTGAGATACAAG     15381
              ************************************************************

LA2093        AGACACGGAACTGAAAAGACCCCAGCAACTTCATGCAAAAACAATTGCCAATTCTCTCCT     15437
E42           AGACACGGAACTGAAAAGACCCCAGCAACTTCATGCAAAAACAATTGCCAATTCTCTCCT     15476
HEINZ         AGACACGGAACTGAAAAGACCCCAGCAACTTCATGCAAAAACAATTGCCAATTCTCTCCT     15441
              ************************************************************

LA2093        CAGAAAATTCACCCTAAGACGCTGAGAATGAAGGCTATCAAGAAGTAAGAATACAAAGTG     15497
E42           CAGAAAATTCACCCTAAGACACTGAGAATGAAGGCTATCAAGAAGTAAGAATACAAAGCG     15536
HEINZ         CAGAAAATTCACCCTAAGACGCTGAGAATGAAGGCTATCAAGAAGTAAGAATACAAAGCG     15501
```

```
          ******************** ************************************* *

LA2093    TGCATGGACTTAATCTTTGTTTCCAGCTCAGATACAGACTCAAACAGAGTGTGCGATGCT    15557
E42       TGCATGGACTTAATCTTTGTTTCCAGCTCAGATACAGACTCAAACAGAGTGTGCGATGCT    15596
HEINZ     TGCATGGACTTAATCTTTGTTTCCAGCTCAGATACAGACTCAAACAGAGTGTGCGATGCT    15561
          ************************************************************

LA2093    CTCCCTTTAACATGGTATAAAGGAGTTACTTGTCCCCAGGTTCAAGCCTAATTCCATTAA    15617
E42       CTCCCTTTAACATGGTATAAAGGAGTTACTTGTCCCCAGGTTCAAGCCTAATTCCATTAA    15656
HEINZ     CTCCCTTTAACATGGTATAAAGGAGTTACTTGTCCCCAGGTTCAAGCCTAATTCCATTAA    15621
          ************************************************************

LA2093    AACATCAAAGTGGACGAAAATCACCAAGTTGAAGTCTTGCAAAGCGTTTGGTGTTAATGC    15677
E42       AACATCAAAGTGGACGAAAATCACCAAGTTGAAGTCTTGCAAAGCGTTTGGTGTTAATGC    15716
HEINZ     AACATCAAAGTGGACGAAAATCACCAAGTTGAAGTCTTGCAAAGCGTTTGGTGTTAATGC    15681
          ************************************************************

LA2093    AGTAAGATTCAAACATGACCTCGTGGGTTTATTTCTTAGAATGGAGTAAAAAAGGCAAAG    15737
E42       AGCAAGATTCAAACATGACCTCGTGGATTTATTTCTTAGAATGGAGTAAAAAAGGCAAAG    15776
HEINZ     AGCAAGATTCAAACATGACCTCGTGGATTTATTTCTTAGAATGGAGTAAAAAAGGCAAAG    15741
          ** *********************** *********************************

LA2093    TCAAATAGAACAACATGAATCCAAGAAGCAGAGGTTAGGGAAAGAGAAGAAGAACAAAAG    15797
E42       TCAAATAGAACAACATGAATCCAAGAAGCAGAGGTTAGGGAAAGAGAAGAAGAACAAAAG    15836
HEINZ     TCAAATAGAACAACATGAATCCAAGAAGCAGAGGTTAGGGAAAGAGAAGAAGAACAAAAG    15801
          ************************************************************

LA2093    AGAAGGGGAAAGGGAAGGAAAGAGACTAATATGTGGGATCAATTATGATAGAAGCAGGCA    15857
E42       AGAAGGGGAAAGGGAAGGAAAGAGACTAATATGTGGGATCAATTATGATAGAAGCAGGCA    15896
HEINZ     AGAAGGGGAAAGGGAAGGAAAGAGACTAATATGTGGGATCAATTATGATAGAAGCAGGCA    15861
          ************************************************************

LA2093    ACAATCACATGAAGTCTAAGGGTAAGCAGTACAACTCATTGTCTGGATGAACTTTAAAAT    15917
E42       ACAATCACATGAAGTCTAAGGGTAAACAGTACAACTCATTGTCTGGATGAACTTTAAAAT    15956
HEINZ     ACAATCACATGAAGTCTAAGGGTAAGCAGTACAACTCATTGTCTGGATGAACTTTAAAAT    15921
          ************************* **********************************

LA2093    CTTGAGTTGGAATATAAAGGGGCTGAATGAGGAGAGGAACAGATGCACAATCAAATTTCT    15977
E42       CTTGAGTTGGAATATAAAGGGGCTGAATGAGGAGAGGAACAGATGCACAATCAAATCTCT    16016
HEINZ     CTTGAGTTGGAATATAAAGGGGCTGAATGAGGAGAGGAACAGATGCACAATCAAATCTCT    15981
          ******************************************************** ***

LA2093    CATTCCAAAGTGGAAAGTCGGTATCTTTTTTTTGCAACAGGAAACCAAAATTGAAGAATG    16037
E42       CATTCCGAAGTGGAAAGTCGGTATCTTTTTTTTGCAACAGGAAACCAAAATTGAAGAATG    16076
HEINZ     CATTCCGAAGTGGAAAGTCGGTATCTTTTTTTTGCAACAGGAAACCAAAATTGAAGAATG    16041
          ****** *****************************************************

LA2093    TTCAGGCTTACTAATCCAACAGTTGTGGGGCAATAGATAGGCAGATTGGGTGGATCTTCA    16097
E42       TTCAGGCTTACTAATCCAACAGTTGTGGGGCAATAGATAGGCAGATTGGGTGGATCTTCA    16136
HEINZ     TTCAGGCTTACTAATCCAACAGTTGTGGGGCAATAGATAGGCAGATTGGGTGGATCTTCA    16101
          ************************************************************

LA2093    AGCATCAGGCACTTGTGTGTG-------GGGGGGGGGGGGGAAGGGGGGTATTCTTGTGA    16150
E42       AGCATCAGGCACTTGTGTGTGT--------GGGGGGGGGGGAAGGGGGGTATTCTTGTGA    16188
HEINZ     AGCATCAGGCACTTGTGTGTGTGTGTGGGGGGGGGGGGGGGGAAGGGGGGTATTCTTGTGA    16161
          *********************         ******************************

LA2093    TCGGGAACAAAAGGAAATGGGTTATGGTGGATACTTTGCTAGGTTGTCATTCTATTTCTT    16210
E42       TCAGGAACAAAAGGAAATGGGTTATGGTGGATACTTTGCTAGGTTGTCATTCTATTTCTT    16248
HEINZ     TCGGGAACAAAAGGAAATGGGTTATGGTGGATACTTTGCTAGGTTGTCATTCTATTTCTT    16221
          ** *********************************************************

LA2093    GTGT--TGGAATGTTCTAATGAGAACTTCAGATGGTGCTTTAATAGTTTCTATGGTCCTC    16268
E42       GTGTACTGGAATGTTCTAATGAGAACTTCAGATGGTGCTTTAATAGTTTCTATGGTCCTC    16308
HEINZ     GTGT--TGGAATGTTCTAATGAGAACTTCAGATGGTGCTTTAATAGTTTCTATGGTCCTC    16279
          ****  ******************************************************

LA2093    ACACAAACCCGCAAAGGGAGGAGATGTGGGATGTATTAGCAGGAGCCAACCGCTCTATCA    16328
E42       ACACAAACCCGCAAAGGGAGGAGATGTGGGATGTATTAGCAGGAGCCAACCGCTCTATCA    16368
HEINZ     ACACAAACCCGCAAAGGGAGGAGATGTGGGATGTATTAGCAGGAGCCAACCGCTCTATCA    16339
          ************************************************************
```

```
LA2093      CTGAGCTACTGAAGAAAAACTGGAGATTCAATCTCATCGATTTCTATTCCTAACAAGCTT     16388
E42         CTGAGCTACTGAAGAAAAACTGGAGATTCAATCTCATCGATTTCTATTCCTAACAAGCTT     16428
HEINZ       CTGAGCTACTGAAGAAAAACTGGAGATTCAATCTCATCGATTTCTATTCCTAACAAGCTT     16399
            ************************************************************

LA2093      CAAGAATAGACAGATTTCTTTTTTTGAAATAAGAATAGACAGATTTCTTATTTCACCAAA     16448
E42         CAAGAATAGACAGATTTCTTTTTTTGAAATAAGAATAGACAGATTTCTTATTTCACCAAA     16488
HEINZ       CAAGAATAGACAGATTTCTTTTTTTGAAATAAGAATAGACAGATTTCTTATTTCACCAAA     16459
            ************************************************************

LA2093      ATGGAGTGATTCCTTTAGAGCTGTGAGACATCCTTGGGTGTAGCATTGCAGCACTTTCAA     16508
E42         ATGGAGTGATTCCTTTAGAGCTGTGAGACATCCTTGGGTGTAGCATTGCAGCACTTTCAA     16548
HEINZ       ATGGAGTGATTCCTTTAGAGCTGTGAGACATCCTTGGGTGTAGCATTGCAGCACTTTCAA     16519
            ************************************************************

LA2093      CCACCAACTTAGGAATTTCTCTTGGAGCTAAGTTTAAAGCAACAAGGTATCTGGAGTTGG     16568
E42         CCACCAACTTAGGAATTTCTCTTGGAGCTAAGTTTAAAGCAACAAGGTATCTGGAGTTGG     16608
HEINZ       CCACCAACTTAGGAATTTCTCTTGGAGCTAAGTTTAAAGCAACAAGGTATCTGGAGTTGG     16579
            ************************************************************

LA2093      CTTCTTGGTAAATCCAATTTATCTATGGGAGGAAGACTTACTATGATCAACAGGTGCTAG     16628
E42         CTTCTTGGTAAATCCAATTTATCTATGGGAGGAAGACTTACTATGATCAACAGGTACTAG     16668
HEINZ       CTTCTTGGTAAATCCAATTTATCTATGGGAGGAAGACTTACTATGATCAACAGGTGCTAG     16639
            ******************************************************* ****

LA2093      ATAGAATTCCAACTTATAATACGTGTCTCCACCCCATCCTGGCAAGGTCTTGGGAAAACT     16688
E42         ATAGAATTCCAACTTATAATACGTGTCTCCACCCCATCCTGGCAAAGTCTTGGGAAAACT     16728
HEINZ       ATAGAATTCCAACTTATAATACGTGTCTCCACCCCATCCTGGCAAGGTCTTGGGAAAACT     16699
            ********************************************* **************

LA2093      TAACAAAATAAGAAGGGATTTCCTTCGGGAAGGAAACAACAATGAGCACAAATTTCACCT     16748
E42         TAACAAAATAAGAAGGGATTTCCTTCGGGAAGGAAACAACAATGAGCACAAATTTCACCT     16788
HEINZ       TAACAAAATAAGAAGGGATTTCCTTCGGGAAGGAAACAACAATGAGCACAAATTTCACCT     16759
            ************************************************************

LA2093      AGTCAAGAGGGACAAAGTCATATTGCCTGAGAGACATGCATACAAAATTTAGCCTTGCAT     16808
E42         AGTCAAGAGGGACAAAGTCATATTGCCTGAGAGACATGCATACAAAATTTAGCCTTGCAT     16848
HEINZ       AGTCAAGAGGGACAAAGTCATATTGCCTGAGAGACATGCATACAAAATTTAGCCTTGCAT     16819
            ************************************************************

LA2093      GACAAGTGTCTCCAGATGAAGTCGCTATAGATATGCACACGGGAGGAGCAAAGTTTATGG     16868
E42         GACAAGTGTCTCCAGATGAAGTCGCTATGGATATGCACACGGGAGGAGCAAAGCTTATGG     16908
HEINZ       GACAAGTGTCTCCAGATGAAGTCGCTATAGATATGCACACGGGAGGAGCAAAGTTTATGG     16879
            **************************** ************************ ******

LA2093      AAGGAAATTATAATTACCAAGCATGGAACATTGAATCACTGGAGATCAAAGCTGTAAAGT     16928
E42         AAGGAAATTATAATTACCAAGCATGGAACACTGAATCACTAGAGATCAAAGCTGTAAAGT     16968
HEINZ       AAGGAAATTATAATTACCAAGCATGGAACATTGAATCACTGGAGATCAAAGCTGTAAAGT     16939
            ****************************** ********* *******************

LA2093      GAACCATATGGGGTTAGACTATGGAAGTATATGAGTAAGAAACGGGAAGAGTTCTCACCG     16988
E42         GAACCATATGGGGTTAGACTATGGAAGTATATGAGTAAGAAACGGGAAGAGTTCTCACCG     17028
HEINZ       GAACCATATGGGGTTAGACTATGGAAGTATATGAGTAAGAAACGGGAAGAGTTCTCACCG     16999
            ************************************************************

LA2093      TTCAACCATCTTGCAATAGGAGATGGCCAGAGAATAAATAATTGGGAAGAAAAATGGCTT     17048
E42         TTCAACCATCTTGAAATAGGAGATGGCCAGAGAATAAATAATTGGGAAGAAAAATGGCTT     17088
HEINZ       TTCAACCATCTTGCAATAGGAGATGGCCAGAGAATAAATAATTGGGAAGAAAAATGGCTT     17059
            ************* **********************************************

LA2093      GAAATTTCTCCTTTAATGTCTGATATCCCCAACATTTTTCATATAGAAGTGAACCTGACT     17108
E42         GAAATTTCTCCTTTAATGTCTGATATCCCCAACATTTTTCATATAGAAGTGAACCTGACT     17148
HEINZ       GAAATTTCTCCTTTAATGTCTGATATCCCCAACATTTTTCATATAGAAGTGAACCTGACT     17119
            ************************************************************

LA2093      CAACAGTACAACAGATCAGGGAAGGAAATGCGTCTCAGAAGGAACCTACAAAACTGGGAG     17168
E42         CAACAGTACAACAGATCAGGGAAGGAAATGCGTCTCAGAAGGAACCTACAAAACTGGGAG     17208
HEINZ       CAACAGTACAACAGATCAGGGAAGGAAATGCGTCTCAGAAGGAACCTACAAAACTGGGAG     17179
            ************************************************************

LA2093      TGGAAATGATAAATCTGCTACCAGCACTTCACTATGGAAACATCAACCCCTACACCAAGA     17228
E42         TGGAAATGATAAATCTGCTACCAGCACTTCACTATGGAAACATCAACCCCTACACCAAGA     17268
```

```
HEINZ       TGGAAATGATAAATCTGCTACCAGCACTTCACTATGGAAACATCAACCCCTACACCAAGA    17239
            ************************************************************

LA2093      CCAATTCAAATGGGGAGTTGCAGATAAAAAGAAATTCTTAGTGAAGGCAACCTACAAACT    17288
E42         CCAATTCAAATGGGGAGTTGCAGATAAAAAGAAATTCTTAGTGAAGGCAACCTACAAACT    17328
HEINZ       CCAATTCAAATGGGGAGTTGCAGATAAAAAGAAATTCTTAGTGAAGGCAACCTACAAACT    17299
            ************************************************************

LA2093      TCTAGGTCCACTGAGAACAATCAGAGAGAGTTGGTCATGAAATCTTTTTTGAAAAAGCAA    17348
E42         TCTAGGTCCACTGAGAACAATCAGAGAGAGTTGGTCATGAAATCTTTTTTGAAAGAGCAA    17388
HEINZ       TCTAGGTCCACTGAGAACAATCAGAGAGAGTTGGTCATGAAATCTTTTTTGAAAAAGCAA    17359
            ****************************************************** *****

LA2093      ATTCCCCCCAAGATAAGTTGCATCAGTTGGACTGCCATGCATGAAGCATGTTTGACACCA    17408
E42         ATTCCCCCCAAGATAAGTTGCATCAGTTGGACTGCCATGCATGAAGCATGTTTGACACAA    17448
HEINZ       ATTCCCCCCAAGATAAGTTGCATCAGTTGGACTGCCATGCATGAAGCATGTTTGACACAA    17419
            ********************************************************** *

LA2093      GAGAACCTAGCGAAGTCGAAGTTTCAGAATTCAGATGGTCAACAGATGCTATATGTGCCT    17468
E42         GAGAACCTAGCGAAGTCGAAGTTTCAGAATTCAGATGGTCAACAGATGCTATATGTGCCT    17508
HEINZ       GAGAACCTAGCGAAGTCGAAGTTTCAGAATTCAGATGGTCAACAGATGCTATATGTGCCT    17479
            ************************************************************

LA2093      GAGGGAAACAAAAACACAGCCACATTTATAAACAATGCCCTGTGGCATCGGATCAATGGA    17528
E42         GAGGGAAACAGAAACACAGCCACATTTATAAACAATGCCCTGTGGCATCGGATCAATGGA    17568
HEINZ       GAGGGAAACAGAAACACAGCCACATTTATAAACAATGCCCTGTGGCATCGGATCAATGGA    17539
            ********** *************************************************

LA2093      GCATGTCCATAACACTTTTTGGTCTCAAATGGGTCATGTCACAAAACATCAAAGATGCAT    17588
E42         GCATGTCCATAACACTTTTTGGTCTCAAATGGGTCATGTCACAAAACATCAAAGATGCAT    17628
HEINZ       GCATGTCCATAACACTTTTTGGTCTCAAATGGGTCATGTCACAAAACATCAAAGATGCAT    17599
            ************************************************************

LA2093      TCAAAAGCTAGAGCTAAATGCATTTTGTATCTGCTTAGTTGGGTTTATTTGTCCCCTATA    17648
E42         TCAAAAGCTAGAGCTAAATGCATTTTGTATCTGCTTAGTTGGGTTTATTTGTCCCCTATA    17688
HEINZ       TCAAAAGCTAGAGCTAAATGCATTTTGTATCTGCTTAGTTGGGTTTATTTGTCCCCTATA    17659
            ************************************************************

LA2093      GATTTCCCAGATACCCTTTTTGAACTTTGCTAGTTCCTTGTCGTTAGTATACTGTTCGTA    17708
E42         GATTTCCCAGATACCCTTTTTGAACTTTGCTAGTTCCTTGTCGTTAGTATACTGTTCGTA    17748
HEINZ       GATTTCCCAGATACCCTTTTTGAACTTTGCTAGTTCCTTGTCGTTAGTATACTGTTCGTA    17719
            ************************************************************

LA2093      ATGGAGCTTCTTAGTTATTTTTGCTACAACTCTCTTGTAACTCGTATGCATCACCTTGAT    17768
E42         ATGGAGCTTCTTAGTTATTTTTGCTACAACTCTCTTGTAACTCGTGTGCATCACCTTGAT    17808
HEINZ       ATGGAGCTTCTTAGTTATTTTTGCTACAACTCTCTTGTAACTCGTATGCATCACCTTGAT    17779
            ********************************************* **************

LA2093      GCAAGCAATGAAAGCCTTAAGTTCATCAGAAAGTAGGATATGTGCAAATGCCCCCAAAAG    17828
E42         GCAAGCAATGAAAGCCTTAAGTTCATCAGAAAATAGGATATGTGCAAATGCCCCCAAAAG    17868
HEINZ       GCAAGCAATGAAAGCCTTAAGTTCATCAGAAAGTAGGAAATGTGCAAATGCCCCCAAAAG    17839
            ******************************** ***** *********************

LA2093      GAAGTGTGGGAGGTTGGGTACAGGGGGTACGAGGAGAGGTAGAGGTAGGCCAAAGAAGCA    17888
E42         GAAGTGTGGGAGGTTGGGTACAGGGGGTACGAGGAGAGGTAGAGGTAGGCCAAAGAAGCA    17928
HEINZ       GAAGTGTGGGAGGTTGGGTACAGGGGGTGCGAGGAGAGGTAGAGGTAGGCCAAAGAAGCA    17899
            **************************** *******************************

LA2093      TTGGAAGAGGTGATTAGATTACATGGCGCAACTTCAGCTTACCAAGGAAAATACGAAGAT    17948
E42         TTGGAAGAGGTGATTAGATTACATGGCGCAACTTCAGCTTACCAAGGAAAATACGAAGAT    17988
HEINZ       TTGGAAGAGGTGATTAGATTACATGGCGCAACTTCAGCTTACCAAGGAAAATACGAAGAT    17959
            ************************************************************

LA2093      GTGGAGGTCGTGAATTAGGGTAGAAGATTAGAGTAGGTAGTGCAGCTTTTTCTTGATTTT    18008
E42         GTGGAGGTCGTGAATTAGGGTAGAAGATTAGAGTAGGTAGTGCAGCTTTTTCTTGATTTT    18048
HEINZ       GTGGAGGTCGTGAATTAGGGTAGAAGATTAGAGTAGGTAGTGCAGCTTTTTCTTGATTTT    18019
            ************************************************************

LA2093      CAACGATATTACGCTTAGTGGCAGTCCATAGCAATGTGCCAATCATAGTATTAGTGTAAT    18068
E42         CAACGATATTACGCTTAGTGGCAGTCCATAGCAATGTGTCAATCATAGTATTAGTGTAAT    18108
HEINZ       CAACGATATTACGCTTAGTGGCAGTCCATAGCAATGTGTCAATCATAGTATTAGTGTAAT    18079
            ************************************** *********************
```

```
LA2093     TCGTGTAGTTTTTAGCTTTTTGTTATCTGTTACCATCTGTTATTTATGGTGTTCCGGTGA     18128
E42        TCGTGTAGTTTTTAGCTTTTTGTTATCTGTTACCATCTGTTATTTATGGTGTTCCGGTGA     18168
HEINZ      TCGTGTAGTTTTTAGCTTTTTGTTATCTGTTACCATCTGTTATTTATGGTGTTCCGGTGA     18139
           ************************************************************

LA2093     TCACACTATTTTGTGGATGTTACTTTTCCTTTCCTGAATGTTCTACACTCCTTCTCTATT     18188
E42        TCACACTATTTTGTGGATGTTACTTTTCCTTTCCTGAATGTTCTACACTCCTTCTCTATT     18228
HEINZ      TCACACTATTTTGTGGATGTTACTTTTCCTTTCCTGAATGTTCTACACTCCTTCTCTATT     18199
           ************************************************************

LA2093     AGTTGGCATGCTGTCTTCACTACTGTTTTCATTTTTCATTACTACTTTGATTTGCTACAC     18248
E42        AGTTGGCATGCTGTCTTCACTACTGTTTTCATTTTTCATTACTACTTTGATTTGCTACAC     18288
HEINZ      AGTTGGCATGCTGTCTTCACTACTGTTTTCATTTTTCAT---TACTTTGATTTGCTACAC     18256
           ***************************************   ******************

LA2093     TTAAGATGAGGGCCCTTTGAAAATAGTTGCTCTACTTCCATGAGGTAGGGGTAAGATCTA     18308
E42        TTAAGATGAGGGCCCTTTGAAAATAGTTGCTCTACTTCCATGAGGTAGGGGTAAGATCTA     18348
HEINZ      TTAAGATGAGGGCCCTTTGAAAATAGTTGCTCTACTTCCATGAGGTAGGGGTAAGATCTA     18316
           ************************************************************

LA2093     TGTACACTCTACCCTCCCCAAACCCTACTTGTGGAATTTCACGAGGTATGTTGTCATTGA     18368
E42        TGTACACTCTACCCTCCCCAAACCCTACTTGTGGAATTTCACGAGGTATGTTGTCATTGA     18408
HEINZ      TGTACACTCTACCCTCCCCAAACCCTACTTGTGGAATTTCACGAGGTATGTTGTCATTGA     18376
           ************************************************************

LA2093     GAGTTTGGAGAACAACTTTGCCCATGTGATTTTTCCATATTGATTCCAAGCCTAGATAAA     18428
E42        GAGTTTGGAGAACAACTTTGCCCATGTGATTTTTCCATATTGATTCCAAGCCTAGATAAA     18468
HEINZ      GAGTTTGGAGAACAACTTTGCCCATGTGATTTTTCCATATTGATTCCAAGCCTAGATAAA     18436
           ************************************************************

LA2093     AGAAGGAAGTAGTAATAGGTTGATGATCACATAAAAAACGAGTTTATAACACAAACCCAT     18488
E42        AGAAGGAAGTAGTAATAGGTTGATGATCACATAAAAAACGAGTTTATAACACAAACCCAT     18528
HEINZ      AGAAGGAAGTAGTAATAGGTTGATGATCACATAAAAAACGAGTTTATAACACAAACCCAT     18496
           ************************************************************

LA2093     TTTGCAAGTTTGTTATTATGGGAAGTTCCTAAAAAGAATCGGACCAATGACATATACAAT     18548
E42        TTTGCAAGTTTGTTATTATGGGAAGTTCCTAAAAAGAATCGGACCAATGACATATACATT     18588
HEINZ      TTTGCAAGTTTGTTATTATGGGAAGTTCCTAAAAAGAATCGGACCAATGACATATACAAT     18556
           ********************************************************** *

LA2093     ACTTGAATTCTTAATATAGATGCTACATAGTTAGTTCTCATCCATAGTTACGGTAGGTTG     18608
E42        ACTTGAATTCTTAATATAGATGCTACATAGTTAGTTCTCATCCATAGTTACGGTAGGTTG     18648
HEINZ      ACTTGAATTCTTAATATAGATGCTACATAGTTAGTTCTCATCCATAGTTACGGTAGGTTG     18616
           ************************************************************

LA2093     AGCCCTTTTTTCAAAGGAATTAATTGTTACTATTTCAAGGTCTATTCATTCACCCACCTT     18668
E42        AGCCCTTTTTTCAAAGGAATTAATTGTTACTATTTCAAGGTCTATTCATTCACCCACCTT     18708
HEINZ      AGCCCTTTTTTCAAAGGAATTAATTGTTACTATTTCAAGGTCTATTCATTCACCCACCTT     18676
           ************************************************************

LA2093     GATAATTTTTTTCTTGTTCGCTAATAAGTCGACTAAATTAAACTCATGTTTCAATAGTCA     18728
E42        GATAATTTTTTTGTTGTTCGCTAATAAGTCGACTAAATTAAACTCATGTTTCAATAGTCA     18768
HEINZ      GATAATTTTTTTCTTGTTCGCTAATAAGTCGACTAAATTAAACTCATGTTTCAATAGTCA     18736
           ************ ***********************************************

LA2093     ATTCAATCTCCCAATTGAATCGAGGACAAACACCTACAAAAAGATTGCTTGAATTCAAGG     18788
E42        ATTCAATCTCCCAATTGAATCGAGGACAAACACCTACAAAAAGATTGCTTGAATTCAAGG     18828
HEINZ      ATTCAATCTCCCAATTGAATCGAGGACAAACACCTACAAAAAGATTGCTTGAATTCAAGG     18796
           ************************************************************

LA2093     AGGTCCCTTTGATTGTCTTTTCTAAGTGACAAGAAGAGGGGAAAAAAGTGGGTCAGAAGA     18848
E42        AGGTCCCTTTGATTGTCCTTTCTAAGTGACAAGAAGAGGGGGAAAAAGTGGGTCAGAAGA     18888
HEINZ      AGGTCCCTTTGATTGTCTTTTCTAAGTGACAAGAAGAGGGGGAAAAAGTGGGTCAGAAGA     18856
           ***************** *********************** ******************

LA2093     AACTGTCATTTTGGACTTACCCAATCAAAGTCCCGAGATGTTTATCAAGTAAATTCAATA     18908
E42        AACTGTCATTTTGGACTTACCCAATCAAAGTCCCGAGATGTTTATCAAGTAAATTCAATA     18948
HEINZ      AACTGTCATTTTGGACTTGCCCAATCAAAGTCCCGAGATGTTTATCAAGTAAATTCAATA     18916
           ****************** *****************************************

LA2093     TAACAGACACCTGTTGGCATTCAAGACTTCTCCTTTCATGGTCAATCCAAAACATAATTA     18968
```

```
E42         TAACAGACACCTGTTGGCATTCAAGACTTCTCCTTTCATGGTCAATCCAAAACATAATTA   19008
HEINZ       TAACAGACACCTGTTGGCATTCAAGACTTCTCCTTTCATGGTCAATCCAAAACATAATTA   18976
            ************************************************************

LA2093      GGTACTTCTTGATCGTAAATATTGTCATTTATTATTCAAGAATTCTTGTTTAGGTGGTTG   19028
E42         GGTACTTCTTGATCGTAAATATTGTCATTTATTATTCAAGAATTCTTGTTTAGGTGGTTG   19068
HEINZ       GGTACTTCTTGATCGTAAATATTGTCATTTATTATTCAAGAATTCTTGTTTAGGTGGTTG   19036
            ************************************************************

LA2093      GTAGGAGCAAAGGGTCTAACAAAGAGTGAAATGCATACAGATCGGAAAGAACACCATTGG   19088
E42         GTAGGAGCAAAGGGTCTAACAAAGAGTGAAATGCATACAGATCGGAAAGAACACCATTGG   19128
HEINZ       GTAGGAGCAAAGGGTCTAACAAAGAGTGAAATGCATACAGATCGGAAAGAACACCATTGG   19096
            ************************************************************

LA2093      TGAATCACAGTAACTGGTCCCAAACATTGTGGGTAGGCCAACTTTGTCCCTAAAATATAA   19148
E42         TGAATCACGGTAACTGGTCCCAAACATTGTGGGTAGGCCAACTTTGTCCCTAAAATATAA   19188
HEINZ       TGAATCACGGTAACTGGTCCCAAACATTGTGGGTAGGCCAACTTTGTCCCTAAAATATAA   19156
            ******** ***************************************************

LA2093      ACTTGAGCACTTCTGATCTTTTTTTACTTTAATAATAAATTTTGTTTGTTGAAAGAGTCG   19208
E42         ACTTGAGCACTTCTGATCTTTTTTTACTTTAATAATAAATTTTGTTTGTTGAAAGAGTCG   19248
HEINZ       ACTTGAGCACTTCTGATCTTTTTTTACTTTAATAATAAATTTTGTTTGTTGAAAGAGTCG   19216
            ************************************************************

LA2093      TTATGAGAGAATTCGTGTAGATGGAAAGTCACAAGCAATACATATGCTCATATCTAAAGA   19268
E42         TTATGAGAGAATTCATGTAGATGGAAAGTCACAAGCAATGCATATGCTCATATCTAAAGA   19308
HEINZ       TTATGAGAGAATTCATGTAGATGGAAAGTCACAAGCAATACATATGCTCATATCTAAAGA   19276
            ************** ************************ ********************

LA2093      CACACTAGCAAGAGTCAAAGCTAATTGTGTCGGCTCTTTTGTATTGGAAAGTTATGTGTC   19328
E42         CACACTAGCAAGAGTCAAAGCTAATTGTGTCGGCTCTTTTGTATTGGAAAGTTATGTGTC   19368
HEINZ       CACACTAGCAAGAGTCAAAGCTAATTGTGTCGGCTCTTTTGTATTGGAAAGTTATGTGTC   19336
            ************************************************************

LA2093      ATATTGTGGTCAGATGAATGTATGTTTTCTCAACCAAAACAGTATGGGTGTGGTGTTTGC   19388
E42         ATATTGTGGTCAGATGAATGTATGTTTTCTCAACCAAAACAGTATGGGTGTGGTGTTTGC   19428
HEINZ       ATATTGTGGTCAGATGAATGTATGTTTTCTCAACCAAAACAGTATGGGTGTGGTGTTTGC   19396
            ************************************************************

LA2093      GTCAGAGTATGAATGCGTGCCTCTAAGAGCTTTCTTCTTGGATGCAATGAATAAACTCTG   19448
E42         GTCAGAGTATGAATGCGTGCCTCTAAGAGCTTTCTTCTTGGATGCAATGAATAAACTCTG   19488
HEINZ       GTCAGAGTATGAATGCGTGCCTCTAAGAGCTTTCTTCTTGGATGCAATGAATAAACTCTG   19456
            ************************************************************

LA2093      AAATTAGAGGGGAACTGGGCACCAATTGTCATTATGGTAAGCTCTTGCATGTCTCTATTG   19508
E42         AAATTAGAGGGGAACTGGGCACCAATTGTCATTATGGTAAGCTCTTGCATGTCTCTATTG   19548
HEINZ       AAATTAGAGGGGAACTGGGCACCAATTGTCATTATGGTAAGCTCTTGCATGTCTCTATTG   19516
            ************************************************************

LA2093      TTGCGGTATGGAGAAAAAGAGTGAGGGAATGTGAAATATATGTCATTTTTTGGGGATGAG   19568
E42         TTGCGGTATGGAGAAAAAGAGTGAGGGAATGTGAAATATATGTCATTTTTTGGGGATGAG   19608
HEINZ       TTGCGGTATGGAGAAAAAGAGTGAGGGAATGTGAAATATATGTCATTTTTTGGGGATGAG   19576
            ************************************************************

LA2093      TGAATGTGTGTGTATTTGGCTAGTCTGCTCTCCCGGTTGTGATAAGAATGAGAGGGTTGT   19628
E42         TGAATGTGTGTGTATTTGGCTAGTCTGCTCTCCCGGTTGTGATAAGAATGAGAGGGTTGT   19668
HEINZ       TGAATGTGTGTGTATTTGGCTAGTCTGCTCTCCCGGTTGTGATAAGAATGAGAGGGTTGT   19636
            ************************************************************

LA2093      TTGTGTATCGTAAAGCTCTTTCTACTTTATTATATACATATATTCTCGTTATGTTGTATT   19688
E42         TTGTGTATCGTAAAGCTCTTTCTACTTTATTATATACATATATTCTCGTTATGTTGTATT   19728
HEINZ       TTGTGTATCGTAAAGCTCTTTCTACTTTATTATATACATATATTCTCGTTATGTTGTATT   19696
            ************************************************************

LA2093      GTATGTGTTGTTCTCTATTACTGGATTTATAGAAGATCAAGAAGGAATGTAAAGATGACT   19748
E42         GTATGTGTTGTTCTCTATTACTGGATTTATAGAAGATCAAGAAGGAATGTAAAGATGACC   19788
HEINZ       GTATGTGTTGTTCTCTATTACTGGATTTATAGAAGATCAAGAAGGAATGTAAAGATGACT   19756
            *********************************************************** 

LA2093      AAAAATACGAGTTAGGTGTAAGATTCTATAGGGTGAGGTAGAAGAGGTCCATGGTGTAGT   19808
E42         AAAAATAGGAGTTAGGTGTAAGATTCTATAGGGTGAGGTAGAAGAGGTCCATGGTGTAGT   19848
HEINZ       AAAAATACGAGTTAGGTGTAAGATTTTATAGGGTGAGGTAGAAGAGGTCCATGGTGTAGT   19816
```

```
                ******* ***************** **********************************

LA2093          ATGTGCAGGAAGAATTGTGTGACACTTTGTTCTACAACGATGAAAAAAA-GGTGGGGTGT     19867
E42             ATGTGCAGGAAGAATTGTGTGACACTTTGTTCTACAACGATGAAAAAAAAGGTGGGGTGT     19908
HEINZ           ATGTGCAGGAAGAATTGTGTGACACTTTGTTCTACAACGATGAAAAAAA-GGTGGGGTGT     19875
                ************************************************* **********

LA2093          TTTTTATAGGTAAAAGAAACAAGAGTTTATTTGGCTCTTTTCTCAAGGAGTAGAGTTGAG     19927
E42             TTTTTATAGGTAAAAGAAACAAGAGTTTATTTGGCTCTTTTCTCAAGGAGTAGAGTTGAG     19968
HEINZ           TTTTTATAGGTAAAAGAAACAAGAGTTTATTTGGCTCTTTTCTCAAGGAGTAGAGTTGAG     19935
                ************************************************************

LA2093          GTTATTAAAGTGATTCTACATGAGTTTTAAAAAACATAATTTGACATTTTACTAAACAAG     19987
E42             GTTATAAAAGTGATTCTACATGAGTTTTAAAAAACATAATTTGACATTTTACTAAACAAG     20028
HEINZ           GTTATAAAAGTGATTCTACATGAGTTTTAAAAAACATAATTTGACATTTTACTAAACAAG     19995
                ***** ******************************************************

LA2093          ACTAAACATACTTAGTTTTTGAAAACTTGAATGACTAAATATGTTCCGTTGTGATATTTC     20047
E42             AATAAACATACTTAGTTTTCGAAAACTTGAATGACTAAATATGTTCCGTTGTGATATTTC     20088
HEINZ           ACTAAACATACTTAGTTTTCGAAAACTTGAATGACTAAATATGTTCCGTTGTGATATTTC     20055
                * ***************** ****************************************

LA2093          GAGGAAAATGGACTTACCCAAACTTAAGAGACATTGTGTAAGAAACTCATAAATTAGCCA     20107
E42             GAGGAAAATGGACTTACCCAAACTTAAGAGACATTGTGTAAGAAACTCATAAATTAGCCA     20148
HEINZ           GAGGAAAATGGACTTACCCAAACTTAAGAGACATTGTGTAAGAAACTCATAAATTAGCCA     20115
                ************************************************************

LA2093          CAATGTGATTACCCTTTAATTATTTTATAGTCTAGTTAGCATACATCTGTATGTAGGGAA     20167
E42             CAATGTGATTACCCTTTAATTATTTTATAGTCTAGTTAGCATACATCTGTATGTAGGGAA     20208
HEINZ           CAATGTGATTACCCTTTAATTATTTTATAGTCTAGTTAGCATACATCTGTATGTAGGGAA     20175
                ************************************************************

LA2093          AGGCATGTTGGTACCTAATTATAATAGTAAATTACATGTATGTTGCTCGGGCTCTTCAAA     20227
E42             AGGCATGTTGGTACCTAATTATAATAGTAAATTACATGTATGTTTCTCGGGCTCTTCAAA     20268
HEINZ           AGGCATGTTGGTACCTAATTATAATAGTAAATTACATGTATGTTGCTCGGGCTCTTCAAA     20235
                ******************************************** ***************

LA2093          AATGTCAACCGG--GTGTGTTGGATCCTCCAAAAATAGTGTTTTTTTAAGGATCCAACAC     20285
E42             AATGTCAACCGGGTGTGTGTTGGATCCTCCAAAAATAGTGTTTTTTTAAGGATCCAACAC     20328
HEINZ           AATGTCAACCGG--GTGTGTTGGATCCTCCAAAAATAGTGTTTTTTTAAGGATCCAACAC     20293
                ************  **********************************************

LA2093          GAATGTGGCAACATTTTTGAAAAGGCCGAGCAACTTAATTACGCTACCTACTATTTTTTT     20345
E42             GAATGTGGCAACATTTTTGAAGAGGCCGAGCAACTTAATTACGCTACCTACTATTTTTTT     20388
HEINZ           GAATGTGGCAACATTTTTGAAGAGGCCGAGCAACTTAATTACGCTACCTACTATTTTTTT     20353
                ********************* **************************************

LA2093          TTCTTATTTTTTGTGACAAGGAGAACTCGTAGTCGCTACCCTTTGGGTGCACACAAGGTA     20405
E42             TTCTTATTTTTTGTGACAAGGAGAACTCGTAGTCGCTACCCTTTGGGTGCACACAAGGTA     20448
HEINZ           TTCTTATTTTTTGTGACAAGGAGAACTCGTAGTCGCTACCCTTTGGGTGCACACAAGGTA     20413
                ************************************************************

LA2093          AAACCTCCGCTCCTATGCAATAGCTCGCAAACCACACAGGAGAGGTAACTCACACTAGGC     20465
E42             AAACCTCCGCTCCTATGCAATAGCTCGCAAACCACACAGGAGAGGTAACTCACACTAGGC     20508
HEINZ           AAACCTCCGCTCCTATGCAATAGCTCGCAAACCACACAGGAGAGGTAACTCACACTAGGC     20473
                ************************************************************

LA2093          AAGCCCGATGCAACGAGCTCGACCCAAAAGGCAATTACTCTACCGACTATATTTACATCT     20525
E42             AAGCCCGATGCAACGAGCTCGACCCAAAAGGCAATTACTCTACCGACTATATTTACATCT     20568
HEINZ           AAGCCCGATGCAACGAGCTCGACCCAAAAGGCAATTACTCTACCGACTATATTTACATCT     20533
                ************************************************************

LA2093          ATTATAGAATATTCACAAGGACTCTTACACACTGATTCTAGAGAAACACTAATATGTCTT     20585
E42             ATTATAGAATATTCACAAGGACTCTTACACACTGATTCTAGAGAAACACTAATATGTCTT     20628
HEINZ           ATTATAGAATATTCACAAGGACTCTTACACACTGATTCTAGAGAAACACTAATATGTCTT     20593
                ************************************************************

LA2093          GAAAGAAAATATTTAGTATTCTAATGAAGTTATCCCAAATAGAAGATGTTTGAAGACTCT     20645
E42             GAAAGAAAATATTTAGTATTCTAATGAAGTTATCCCAAATAGAAGATGTTTGAAGACTCT     20688
HEINZ           GAAAGAAAATATTTAGTATTCTAATGAAGTTATCCCAAATAGAAGATGTTTGAAGACTCT     20653
                ************************************************************
```

```
LA2093      GCAAGCTCTACCAAAAGGTTAAAGTCTTAATTGTATATTTTGTTTTGCTTATGGTGTAGG    20705
E42         GCAAGCTCTACCAAAAGGTTAAAGTCTTAATTGTATATTTTGTTTTGCTTATGGTGTAGG    20748
HEINZ       GCAAGCTCTACCAAAAGGTTAAAGTCTTAATTGTATATTTTGTTTTGCTTATGGTGTAGG    20713
            ************************************************************

LA2093      ATGGAACATGTATATGAGGCTGAAATAGTTCTACAATCATAAGACTCTTAACAAGATTAG    20765
E42         ATGGAACATGTATATGAGGCTGAAACAGTTCTACAATCATAAGACTCTTAACAAGATTAG    20808
HEINZ       ATGGAACATGTATATGAGGCTGAAATAGTTCTACAATCATAAGACTCTTAACAAGATTAG    20773
            ************************* **********************************

LA2093      ACAGGGCACAGTTTTGTTGAGTGTTCTTTTGCTAATGGTACATCCTGATAGCATATCCTT    20825
E42         ACAGGGCACAGTTTTGTTGAGTGTTCTTTTGCTAATGGTACATCCTGATAGCATATCCTT    20868
HEINZ       ACAGGGCACAGTTTTGTTGAGTGTTCTTTTGCTAATGGTACATCCTGATAGCATATCCTT    20833
            ************************************************************

LA2093      TGCGCTGATCTAATTTATAAAATTACTTTACCTTCTCGAATATATTAGTCCAAATAGAGA    20885
E42         TGCGCTGATCTAATTTATAAAATTACTTTACCTTCTCGAATATATTAGTCCAAATAGAGA    20928
HEINZ       TGCGCTGATCTAATTTATAAAATTACTTTACCTTCTCGAATATATTAGTCCAAATAGAGA    20893
            ************************************************************

LA2093      ACACTTGAAATAGAGGATCTTTTCATGGATATAGAAGATTCATATACTACGGCTTTGAGG    20945
E42         ACACTTGAAATAGAGGATCTTTTCATGGATATAGAAGATTCATATACTACGGCTTTGAGG    20988
HEINZ       ACACTTGAAATAGAGGATCTTTTCATGGATATAGAAGATTCATATACTACGGCTTTGAGG    20953
            ************************************************************

LA2093      CATAGTAATTATTTGATTGTAGTGCTAGGAAATTATTGATCTTCCACGTGATGTAATTTA    21005
E42         CATAGTAATTATTTGATTGTAGTGCTAGGAAATTATTGATCTTCCACGTGATGTAATTTA    21048
HEINZ       CATAGTAATTATTTGATTGTAGTGCTAGGAAATTATTGATCTTCCACGTGATGTAATTTA    21013
            ************************************************************

LA2093      TGTACAAATACTGACTTTTCAATGTCAAATGTAGGCAACAAATATGAGGTGACTAGTGTG    21065
E42         TGTACAAATACTGACTTTTCAATGTCAAATGTAGGCAACAAATATGAGGTGACTAGTGTG    21108
HEINZ       TGTACAAATACTGACTTTTCAATGTCAAATGTAGGCAACAAATATGAGGTGACTAGTGTG    21073
            ************************************************************

LA2093      CAACTGTTTTGAACCATAAATTTCCATTCTACAATTGCGCACAAGTCTTTTCTATAAGCT    21125
E42         CAACTGTTTTGAACCATAAATTTCCATTCTACAATTGCGCACAAGTCTTTTCTATAAGCT    21168
HEINZ       CAACTGTTTTGAACCATAAATTTCCATTCTACAATTGCGCACAAGTCTTTTCTATAAGCT    21133
            ************************************************************

LA2093      GCCTAAGCCAGTCACAATATGTTTCAAGCTGTTCAAGCCAAGTTTCTGTGCAGGTAGTTC    21185
E42         GCCTAAGCCAGTCACAATATGTTTCAAGCTGTTCAAACCAAGTTTCTGTGCAGGTAGTTC    21228
HEINZ       GCCTAAGCCAGTCACAATATGTTTCAAGCTGTTCAAGCCAAGTTTCTGTGCAGGTAGTTC    21193
            ************************************ ***********************

LA2093      TGGTTCTAGTTCAGCACAGTATCAAGTATGATTTGATTTTTCCATTTATAACGAAACTTC    21245
E42         TGGTTCTAGTTCAGCACAGTGTCAAGTATGATTTGATTTTTCCATTTATAACGAAACTTC    21288
HEINZ       TGGTTCTAGTTCAGCACAGTATCAAGTATGATTTGATTTTTCCATTTATAACGAAACTTC    21253
            ******************** ***************************************

LA2093      ACCTACTTATTTTGTTCTAGCTGAAGTATGCAGATGGGGCCTCCTGGTTTTTCATACAGT    21305
E42         ACCTACTTATTTTGTTCTAGCTGAAGTATGCAGATGGGGCCTCCTGGTTTTTCATACAGT    21348
HEINZ       ACCTACTTATTTTGTTCTAGCTGAAGTATGCAGATGGGGCCTCCTGGTTTTTCATACAGT    21313
            ************************************************************

LA2093      ACTATGGCCAACGTCATGGAATATAGTAGTGGAACACAGAACCTCAGGATATGCTGTTGA    21365
E42         ACTATGGCCAACGTCATGGAATATAGTAGTGGAACACAGAACCTCAGGATATGCTGTTGA    21408
HEINZ       ACTATGGCCAACGTCATGGAATATAGTAGTGGAACACAGAACCTCAGGATATGCTGTTGA    21373
            ************************************************************

LA2093      GAGCAGATACACAGATAAAAGGTTTTGAGGTCTTGAATACTTTATACCTTCACCAGGAGA    21425
E42         GAGCAGATACACAGATAAAAGGTTTTGAGGTCTTGAATACTTTATACCTTCACCAGGAGA    21468
HEINZ       GAGCAGATACACAGATAAAAGGTTTTGAGGTCTTGAATACTTTATACCTTCACCAGGAGA    21433
            ************************************************************

LA2093      AGTCAACAAACTATTCATTAATATAGTGTCATATCCCACAAGTCTGTTTATAAGAAGCTG    21485
E42         AGTCAACAAACTATTCATTAATATAGTGTCATATCCCACAAGTCTGTTTATAAGAAGCTG    21528
HEINZ       AGTCAACAAACTATTCATTAATATAGTGTCATATCCCACAAGTCTGTTTATAAGAAGCTG    21493
            ************************************************************

LA2093      CTGCCACCTGGATAAGAAAAAAGAAGTTTTCCATTAAAATATGAAGTTTTACCTAAATAA    21545
E42         CTGCCACCTGGATAAGAAAAAAGAAGTTTTCCATTAAAATATGAAGTTTTACCTAAATAA    21588
```

```
HEINZ     CTGCCACCTGGATAAGAAAAAAGAAGTTTTCCATTAAAATATGAAGTTTTACCTAAATAA     21553
          ************************************************************

LA2093    GTTATCAGAACTCAATTGTATTTAAATCTTCAAATGTGAACTGAATTCAATAAGAAAAAC     21605
E42       GTTATCAGAACTCAATTGTATTTAAATCTTCAAATGTGAACTGAATTCAATAAGAAAAAC     21648
HEINZ     GTTATCAGAACTCAATTGTATTTAAATCTTCAAATGTGAACTGAATTCAATAAGAAAAAC     21613
          ************************************************************

LA2093    AATCTTTGACCAACCAAGTCGTAATCACAAATAAGAGGGCATAAGAAAGAAAAGGAAGTT     21665
E42       AATCTTTGACCAACCAAGTCGTAATCACAAATAAGAGGGCATAAGAAAGAAAAGGAAGGT     21708
HEINZ     AATCTTTGACCAACCAAGTCGTAATCACAAATAAGAGGGCATAAGAAAGAAAAGGAAGTT     21673
          ********************************************************** *

LA2093    TTTGGGTAAAGGAATCCTTGCAGTTGTAATTTGTTGATGAATGACTGGTGTTACAGCAAA     21725
E42       TTTGGGTAAAGGAATCCTTGCAGTTGTAATTTGTTGATGAATGACTGGTGTTACAGCAAA     21768
HEINZ     TTTGGGTAAAGGAATCCTTGCAGTTGTAATTTGTTGATGAATGACTGGTGTTACAGCAAA     21733
          ************************************************************

LA2093    AGTGTACTCATAGGAGTAAAAGATTTACTAATCAAGCAGGAAATTGAAAGAGAAAAAAGG     21785
E42       AGTGTACTCATAGGAGTAAAAGATTTACTAATCAAGCAGGAAATTGAAAGAGAAAAAAGG     21828
HEINZ     AGTGTACTCATAGGAGTAAAAGATTTACTAATCAAGCAGGAAATTGAAAGAGAAAAAAGG     21793
          ************************************************************

LA2093    GAAAGGAACATTATTTGCATTGTAAGACCGATCTGTCCCATGTTTTTCCACCACGTCTGT     21845
E42       GAAAGGAACATTATTTGCATTGTAAGACCGATCTGTCCCATGTTTTTCCACCACGTCCGT     21888
HEINZ     GAAAGGAACATTATTTGCATTGTAAGACCGATCTGTCCCATGTTTTTCCACCACGTCTGT     21853
          ********************************************************* **

LA2093    TCTAAGAGATAACAAAACAACATCAGTAAATGTAAAATGGGAGATAAAACGAGGGGGGGG     21905
E42       TCTAAGAGATAACAAAACAACATCAGTAAATGTAAAATGGGAGATAAAACGA--------     21940
HEINZ     TCTAAGAGATAACAAAACAACATCAGTAAATGTAAAATGGGAGATAAAACGA--------     21905
          ****************************************************

LA2093    GGGGGGGGGAGATAAAACAAAGATGAGAGTTTTTTTTGTGTGTGATAAAGTTAAAGTTAA     21965
E42       GGGGGGGGGAGATAAAACAAAGATGAGAGTTTTTTTTGTGTGTGATAAAGTTAAAGTTAA     22000
HEINZ     GGGGGGGGGAGATAAAACAAAGATGAGAGTTTTTTTTGTGTGTGATAAAGTTAAAGTTAA     21965
          ************************************************************

LA2093    AACAGAGATGAGAGTTCTTCATCAATATCTGACATTTGCTGGGTAACCTGCAATCTCCCA     22025
E42       AACAGAGATGAGAGTTCTTCATCAATATCTGACATTTGCTGGGTAACCTGCAATCTCCCA     22060
HEINZ     AACAGAGATGAGAGTTCTTCATCAATATCTGACATTTGCTGGGTAACCTGCAATCTCCCA     22025
          ************************************************************

LA2093    GTAACCTTCGGCAAGAACTATCTCCGGCAAACTCCTCAGAGTAATAGAACCGGCCAGCGA     22085
E42       GTAACCTTCGGCAAGAACTATCTCCGGCAAACTCCTCAGAGTAATAGAACCGGCCAGCGA     22120
HEINZ     GTAACCTTCGGCAAGAACTATCTCCGGCAAACTCCTCAGAGTAATAGAACCGGCCAGCGA     22085
          ************************************************************

LA2093    CTATGACTGAATAAGGAAAATACTGCTATGGTCTTCTTCTTCTTCTTTCCTTCATTTCCT     22145
E42       CTATGACTGAATAAGGAAAATACTGCTATGGTCTTCTTCTTCTTCTTTCCTTCATTTCCT     22180
HEINZ     CTATTACTGAATAAGGAAAATACTGCTATGGTCTTCTTCTTCTTCTTTCCTTCATTTCCT     22145
          **** *******************************************************

LA2093    TGGCAAGTAGTCCACCTGCTTTCTGTGTAAGGTTCCATGACACCGAATTCCACATTGGCT     22205
E42       TGGCAAGTAGTCCACCTGCTTTCTGTGTAAGGTTCCATGACACCGAATTCCACATTGGCT     22240
HEINZ     TGGCAAGTAGTCCACCTGCTTTCTGTGTAAGGTTCCATGACACCGAATTCCACATTGGCT     22205
          ************************************************************

LA2093    CATGTGGTCTATGTCGGTTAAGGCAAGATGTTCCCGAAAGCAAAATGACGTAATAAAGTG     22265
E42       CATGTGGTCTATGTCGGTTAAGGCAAGATGTTCCCGAAAGCAAAATGACGTAATAAAGTG     22300
HEINZ     CATGTGGTCTATGTCGGTTAAGGCAAGATGTTCCCGAAAGCAAAATGACGTAATAAAGTG     22265
          ************************************************************

LA2093    AACCCTAACTAAGATGACTTAAGGGGTTTGACTTAGTCTAGGTAGGGGTATGGG-ACTCT     22324
E42       AACCCTAACTAAGATGACTTGAGGGGTTTGACTTAGTCTAGGTAGGGGTATGGGAACTCT     22360
HEINZ     AACCCTAACTAAGATGACTTGAGGGGTTTGACTTAGTCTAGGTAGGGGTATGGG-ACTCT     22324
          ******************** ********************************* *****

LA2093    ACCTATGCATTGCACTAGTTAGCCTTGAGGGAAGTCTTAGAAGGATGTTCTTATGTGCTT     22384
E42       ACCTATGCATTGCACTAGTTAGCCTTGAGGGAAGTCTTAGAAGGATGTTCTTATGTGCTT     22420
HEINZ     ACCTATGCATTGCACTAGTTAGCCTTGAGGGAAGTCTTAGAAGGATGTTCTTATGTGCTT     22384
          ************************************************************
```

```
LA2093      ATATGTTTATAAATGTAAATGATATGTATATTTTGATCTTACATGTTAATGACACTATGT    22444
E42         ATATGTTTATAAATGTAAATGATATGTATATTTTGATCTTACATGTTAATGACACTATGT    22480
HEINZ       ATATGTTTATAAATGTAAATGATATGTATATTTTGATCTTACATGTTAATGACACTATGT    22444
            ************************************************************

LA2093      GATATTGGTTTCACCTATGAACATGTGGTTAATCTCTATTGCTAAAGTATGATGATGTAT    22504
E42         GATATTGGTTTCACCTATGAACATGTGGTTAATCTCTATTGCTAAAGTATGATGATGTAT    22540
HEINZ       GATATTGGTTTCACCTATGAACATGTGGTTAATCTCTATTCCTAAAGTATGATGATGTAT    22504
            **************************************** *******************

LA2093      ACGCTTAATAATAAAGTATATAAAGTTGGACATTTCTTATAGTTCTTGTGGGTTTTACTT    22564
E42         ACGCTTAATAATAAAGTATATAAAGTTGGACATTTCTTATAGTTCTTGTGGGTTTTACTT    22600
HEINZ       ACGCTTAATAATAAAGTATATAAAGTTGGACATTTCTTATAGTTCTTGTGGGTTTTACTT    22564
            ************************************************************

LA2093      GGTTGAGTGAGGTTATGGGACCTTACTTAGTCATTGCACTAGTGGACTTAATGGGGGTGT    22624
E42         GGTTGAGTGAGGTTATGGGACCTTACTTAGTCATTGCACTAGTGGACTTAATGGGGGTGT    22660
HEINZ       GGTTGAGTGAGGTTATGGGACCTTACTTAGTCATTGCACTAGTGGACTTAATGGGGGTGT    22624
            ************************************************************

LA2093      ATGAGTGATGGTCTTGCTTAGGTTTTTATGTTATGAACTCTTATGCATGCTTGTTGATAT    22684
E42         ATGAGTGATGGTCTTGCTTAGGTTTTTATGTTATGAACTCTTATGCATGCTTGTTGATAT    22720
HEINZ       ATGAGTGATGGTCTTGCTTAGGTTTTTATGTTATGAACTCTTATGCATGCTTGTTGATAT    22684
            ************************************************************

LA2093      TATAACTCGATGATAGAGTTGTCTTGATTATGTGCTCAATTATGTTGATATGTATGTTGA    22744
E42         TATAACTCGATGATAGAGTTGTCTTGATTATGTGCTCAATTATGTTGATATGTATGTTGA    22780
HEINZ       TATAACTCGATGATAGAGTTGTCTTGATTATGTGCTCAATTATGTTGATATGTATGTTGA    22744
            ************************************************************

LA2093      CTTGACTAGACTTTGTATTGTTGGTCTTTTGATGTACATGTCTTGACTTAAGTAATATGT    22804
E42         CTTGACTAGACTTTGTATTGTTGGTCTTTTGATGTACATGTCTTGACTTAAGTAATATGT    22840
HEINZ       CTTGACTAGACTTTGTATTGTTGGTCTTTTGATGTACATGTCTTGACTTAAGTAATATGT    22804
            ************************************************************

LA2093      GCTTGTTGACTTGTATCGGTTCTCGAATGTGCATAAGGTATTTCCAAAGTGACTGCATGC    22864
E42         GCTTGTTGACTTGTATCGGTTCTCGAATGTGCATAAGGTATTTCCAAAGTGACTGCATGC    22900
HEINZ       GCTTGTTGACTTGTATCGGTTCTCGAATGTGCATAAGGTATTTCCAAAGTGACTGCATGC    22864
            ************************************************************

LA2093      TTTTGAATAAATGTCCCTCTTTGGCATGATTTATATGTTGTATGTACATATGATTCCATA    22924
E42         TTTTGAATAAATGTCCCTCTTTGGCATGATTTATATGTTGTATGTACATATGATTCCATA    22960
HEINZ       TTTTGAATAAATGTCCCTCTTTGGCATGATTTATATGTTGTATGTACATATGATTCCATA    22924
            ************************************************************

LA2093      CTTAGTACTAGTGATGTGCTAAACCATTTCTCCATTTTTCCCCAACATTTTAGGTTCCGG    22984
E42         CTTAGTACTAGTGATGTGCTAAACCATTTCTCCATTTTTCCCCAACATTTTAGGTTCCGG    23020
HEINZ       CTTAGTACTAGTGATGTGCTAAACCATTTCTCCATTTTTCCCCAACATTTTAGGTTCCGG    22984
            ************************************************************

LA2093      TCATTGAAGGGTTTGTGACCACTTTGAAGGAAGGCTTGGATCATTTCTCTAAGTGTGGGT    23044
E42         TCATTGAAGGGTTTGTGACCACTTTGAAGGAAGGCTTGGATCATTTCTCTAAGTGTGGGT    23080
HEINZ       TCATTGAAGGGTTTGTGACCACTTTGAAGGAAGGCTTGGATCATTTCTCTAAGTGTGGGT    23044
            ************************************************************

LA2093      ATGTCGTCATGTTCCGAGGAAGATGTCTTTCTATCACTCTACCTATGTTACATTGTTATA    23104
E42         ATGTCGTCATGTTCCGAGGAAGATGTCTTTCTAT--------------------------    23114
HEINZ       ATGTCGTCATGTTCCGAGGAAGATGTCTTTCTAT--------------------------    23078
            **********************************

LA2093      GTCTAAAGACACTCATCTCACTGATGTTGTTTCAAGACATTGTTGTAAGCCCTAAGTGGC    23164
E42         ---------CACTCATCTCACTGATGTTGTTTCAAGACATTGTTGTAAGCCCTAAGTGGC    23165
HEINZ       ---------CACTCATCTCACTGATGTTGTTTCAAGACATTGTTGTAAGCCCTAAGTGGC    23129
                     ***************************************************

LA2093      ACTTTTACATTTGTGTAAGGGTTATGTCCATATTGTTCTATTCTGTTTAGATGGTTTAAT    23224
E42         ACTTTTACATTTGTGTAAGGGTTATGTCCATATTGTTCTATTCTGTTTAGATGGTTTAAT    23225
HEINZ       ACTTTTACATTTGTGTAAGGGTTATGTCCATATTGTTCTATTCTGTTTAGATGGTTTAAT    23189
            ************************************************************

LA2093      ATGAGACATCCCTTTTGTTAGAATAGGAATAAGTATTTCTTACTTAGAATAGAAATTAGA    23284
```

```
E42        ATGAGACATCCCTTTTGTTAGAATAGGAATAAGTATTTCTTACTTAGAATAGAAATTAGA    23285
HEINZ      ATGAGACATCCCTTTTGTTAGAATAGGAATAAGTATTTCTTACTTAGAATAGAAATTAGA    23249
           ************************************************************

LA2093     ATAGGAATAAGAATAGGAATTCTAGTTGGAAAAGGTTTTCAATGTAGTGTGTATAAATAG    23344
E42        ATAGGAATAAGAATAGGAATTCTAGTTGGAAAAGGTTTTCAATGTAGTGTCTATAAATAG    23345
HEINZ      ATAGGATTAAGAATAGGAATTCTAGTTGGAAAAGGTTTTCAATGTAGTGTGTATAAATAG    23309
           ****** ******************************************* *********

LA2093     GGTCTTCATTAACAATTTAGATGTGCAATTCAATAATATTTTCTCCAATATTTCTCACAT    23404
E42        GGTCTTCATTAACAATTTAGATGTGCAATTCAATAATATTTTCTCCAATATTTCTCACAT    23405
HEINZ      GGTCTTCATTAACAATTTAGATGTGCAATTCAATAATATTTTCTCCAATATTTCTCACAT    23369
           ************************************************************

LA2093     GGTATCAGAGCAATTATGAGAAAACAAAATT-AAAAAAAAATATTTTCCGGTAACCTAGT    23463
E42        GGTATCAGAGCAATTGTGAGAAAACAAAATTAAAAAAAAAATATTTTCCGGTAACCTAGT    23465
HEINZ      GGTATCAGAGCAATTGTGAGAAAACAAAATT-AAAAAAAAATATTTTCCGGTAACCTAGT    23428
           *************** *************** ****************************

LA2093     TGCTGTCCGGATAATGATTATTTTCCGTCACTGTTTTCATAAAATTGACACCACTACTAG    23523
E42        TGCTGTCCGGATAATGATTATTTTCCGTCACTGTTTTCATAAAATTGACACCACTACTAG    23525
HEINZ      TGCTGTCCGGATAATGATTATTTTCCGTCACTGTTTTCATAAAATTGACACCACTACTAG    23488
           ************************************************************

LA2093     CTGCGGAATTTCCCGGCGAGCAAAGCCCAGTCATCCCCACCGAAAACCAGTGCTACAGTG    23583
E42        CTGCGGAATTTCCCGGCGAGCAAAGCCCAATCATCCCCACCGAAAACCAGTGCTACAGTG    23585
HEINZ      CTGCGGAATTTCCCGGCGAGCAAAGCCCAGTCATCCCCACCGAAAACCAGTGCTACAGTG    23548
           ***************************** ******************************

LA2093     TCGTCACGCGCTGCCTATTCAATTGCCGGAATCAGGTTTCCGACTATTTTCAAGTTGTTT    23643
E42        TCGTCACGCGCCGCCTATTCAATTGCCGGAATCAGGTTTCCGACTATTTTCAAGCTGTTT    23645
HEINZ      TCGTCACGCGCTGCCTATTCAATTGCCGGAATCAGGTTTCCGACTATTTTCAAGTTGTTT    23608
           *********** ****************************************** *****

LA2093     CCGACAAAAAAAATTTCGAGGTTTGAATTTTCCGGCGATCAAATCTCAATAATCAAGATTT    23703
E42        CTGACAAAAAAAATTTCGAGGTTTGAATTTTCCGACGATCAAATCTCAATAATCAAGATTT    23705
HEINZ      CCGACAAAAAAAATTTCGAGGTTTGAATTTTCCGGCGATCAAATCTCAATAATCAAGATTT    23668
           * ******************************** *************************

LA2093     TTTTGTTGTTCCCAAGACAAATTTAGCATAATGTCTTTTGGATGTGATGTTTTTGGTTCT    23763
E42        TTTTGTTGTTCCCAAGACAAATTTAGCATAATGTCTTTTGGATGTGATGTTTTTGGTTCT    23765
HEINZ      TTTTGTTGTTCCCAAGACAAATTTAGCATAATGTCTTTTGGATGTGATGTTTTTGGTTCT    23728
           ************************************************************

LA2093     ACGAATATAGGGATTAGAATTCTAGCCCTATAATCACTTCAGAACTTCAATTGGGAAGTT    23823
E42        ACGAATATAGGGATTAGAATTCTAGCCCTATAATCACTTCAGAACTTCAATTGGGAAGTT    23825
HEINZ      ACGAATATAGGGATTAGAATTCTAGCCCTATAATCACTTCAGAACTTCAATTGGGAAGTT    23788
           ************************************************************

LA2093     CAAACTATTTGTCTTGGGCTTCGTCAGTTGAATTGTGGTGCAAGGGTCAAGGTGTGCACG    23883
E42        CAAACTATTTGTCTTGGGCTTCGTCAGTTGAATTGTGGTGCAAGGGTCAAGGTGTGCACG    23885
HEINZ      CAAACTATTTGTCTTGGGCTTCGTCAGTTGAATTGTGGTGCAAGGGTCAAGGTGTGCACG    23848
           ************************************************************

LA2093     ATTACATAACGAACAAGGTTACAAGACTTATGTGATAGATGTAAAGGCTATGACTAGTGA    23943
E42        ATTACTTAACGAACAAGGTTACAAGACTTATGTGATAGATGTAAAGGCTATGACTAGTGA    23945
HEINZ      ATTACATAACGAACAAGGTTACAAGACTTATGTGATAGATGTAAAGGCTATGACTAGTGA    23908
           ***** ******************************************************

LA2093     GGAAGATGCAAAAGTCAAAGCACATAAGGAGAAAGTAGATGTTCAATTATGTAGTCTCCT    24003
E42        GGAAGATGCAAAAGTCAAAGCACATAAGGAGAAAGTAGATGTTCAATTATGTAGTCTCCT    24005
HEINZ      GGAAGATGCAAAAGTCAAAGCACATAAGGAGAAAGTAGATGTTCAATTATGTAGTCTCCT    23968
           ************************************************************

LA2093     ATGGTGTTCGACTGATTCCTAATTGATGCCCTTGTTTCGCCCATTCCAAACATGTTATAC    24063
E42        ATGGCGTTCGACTGATTCCTAATTGATGCCCTTGTTTCGCCCATTCCAAACATGTTATAC    24065
HEINZ      ATGGCGTTCGACTGATTCCTAATTGATGCCCTTGTTTCGCCCATTCCAAACATGTTATAC    24028
           **** *******************************************************

LA2093     AGTTTGGGAAAAGGCGTGTGCTTTATACACTAATGACATCTCGATTCTATGATGTGATAT    24123
E42        AGTTTGGGAAAAGGCGTGT-CTTTATACACTAATGACATCTCGATTCTATGATGTGATAT    24124
HEINZ      AGTTTGGGAAAAGGCGTGTGCTTTATACACTAATGACATCTCGATTCTATGATGTGATAT    24088
```

```
              ******************* ****************************************

LA2093        CTCGATTGACCAACTTAAAGAAAAACGAATCTGATGTCTACTTACTTGGGACAGGTGCAG     24183
E42           CTCGATTGACCAACTTAAAGAAAAACGAATCTGATGTCTACTTACTTGGGACAGGTACAG     24184
HEINZ         CTCGATTGACCAACTTAAAGAAAAACGAATCTGATGTCTACTTACTTGGGACAGGTACAG     24148
              ******************************************************** ***

LA2093        GCAGTCATGGAGGAATTCGACATATTGATGTCAGTAACTACGGATTTGGAAAAACAACAA     24243
E42           GCAGTCATGGAGGAATTCGACATATTGATGTCAGTAACTACGGATTTGGAAAAACAACAA     24244
HEINZ         GCAGTCATGGAGGAATTCGACATATTGATGTCAGTAACTACGGATTTGGAAAAACAACAA     24208
              ************************************************************

LA2093        GAACATAGACAAACATTGTTTCTAGTTCTTACTCTTGCTGGACTTCCTCCTGATAATGAT     24303
E42           GAACATAGACAAACATTGTTTCTAGTTCTTACTCTTGCTGGACTTCCTCCTGATAATGAT     24304
HEINZ         GAACATAGACAAACATTGTTTCTAGTTCTTACTCTTGCTGGACTTCCTCCTGATAATGAT     24268
              ************************************************************

LA2093        TCTGTGCGTGATCAGATTTTAGCTAGCCCTACAGTTCCTACAATCAATGAATTATTCTCT     24363
E42           TCTGTGCGTGATCAGATTTTAGCTAGCCCTACAGTTCCTACAATCAATGAATTATTCTCT     24364
HEINZ         TCTGTGCGTGATCAGATTTTAGCTAGCCCTACAGTTCCTACAATCAATGAATTATTCTCT     24328
              ************************************************************

LA2093        CACCTCCTTCATCTTGCGGCACCTCCTAGTCACAAAGTCGTTTCATCACCCACTGTTGAC     24423
E42           CGCCTCCTTCATCTTGCGGCACCTCCTAGTCACAAAGTCGTTTCATCACCCACTGTTGAC     24424
HEINZ         CGCCTCCTTCATCTTGCGGCACCTCCTAGTCACAAAGTCGTTTCATCACCCACTGTTGAC     24388
              * **********************************************************

LA2093        TACTCTATTCTCGCATCTCAAACCTTTGAAAAGCGTACATATCAGTCTACGGAGAATCGG     24483
E42           TCCTCTATTCTCGCATCTCAAACCTTTGAAAAGCGTACATATCAGTCTACGGAGAATCGG     24484
HEINZ         TACTCTATTCTCGCATCTCAAACCTTTGAAAAGCGTACATATCAGTCTACGGAGAATCGG     24448
              * **********************************************************

LA2093        CGAGGGGGAGGTCGTTTTGGGAAACCTCGATCCAAGTGTAGTCATTATCATAAGTTTGGA     24543
E42           CGAGGGGGAGGACGTTTTGGGAAACCTCGATCCAAGTGTAGTCATTATCATAAGTTTGGA     24544
HEINZ         CGAGGGGGAGGTCGTTTTGGGAAACCTCGATCCAAGTGTAGTCATTATCATAAGTTTGGA     24508
              *********** ************************************************

LA2093        CACACTCGTGACATATGCCATATTTTGCATGGTTCACCATCCAATTATGATCCTCTTGTT     24603
E42           CACACTCGTGACATATGCCATATTTTGCATGGTTCACCACCCAATTATGATCCTCTTGTT     24604
HEINZ         CACACTCGTGACATATGCCATATTTTGCATGGTTCACCATCCAATTATGATCCTCTTGTT     24568
              *************************************** ********************

LA2093        CTAAAGGAATATAATGAGTTCCTTCGAAATCGCGCAAGTAAATAGTCATCTCCACGAGTA     24663
E42           CTAAAGGAATATAATGAGTTCCTTCGAAATCGCGCAAGTAAATAGTCATCTCCACGAGTA     24664
HEINZ         CTAAAGGAATATAATGAGTTCCTTCGAAATCGCGCAAGTAAATAGTCATCTCCACGAGTA     24628
              ************************************************************

LA2093        GCATATGGTGCTCAACCTAATCAACCATCCAATAATGCTCATATTGCTCAGATAGAATAT     24723
E42           GCATATGGTGCTCAACCTAATCAACCATCCAATAATGCTCATATTGCTCAGATAGAATAT     24724
HEINZ         GCATATGGTGCTCAACCTAATCAACCATCCAATAATGCTCATATTGCTCAGATAGAATAT     24688
              ************************************************************

LA2093        GATGAGTTCCTTCAGTATCGTGCAAATGACAAACGTCTCCATAAGTAGTTTCGATTACAC     24783
E42           GATGAGTTCCTTCAGTATCGTGCAAATGACAAACGTCTCCATAAGTAGTTTCGATTGCAC     24784
HEINZ         GATGAGTTCCTTCAGTATCGTGCAAATGACAAACGTCTCCATAAGTAGTTTCGATTGCAC     24748
              ******************************************************** ***

LA2093        AACCTGTTGTGTCTGCCACGGGTAATTCTTTTGCTTGTGTGTCGCAATCTAGTACTGTTG     24843
E42           AACCTGATGTGTCTGCCACGGGTAATTCTTTTGCTTGTGTGTCGCAATCTAGTACTGTTG     24844
HEINZ         AACCTGATGTGTCTGCCACGGGTAATTCTTTTGCTTGTGTGTCGCAATCTAGTACTGTTG     24808
              ****** *****************************************************

LA2093        GAACATGGGTCGTGGACTCTGGGGCTTCTGATCATATTTCTGGTAATAAATCACTTTTAT     24903
E42           GAACATGGGTCGTGGACTCTGGGGCTTCTGATCATATTTCTGGTAATAAATCACTTTTAT     24904
HEINZ         GAACATGGGTCGTGGACTCTGGGGCTTCTGATCATATTTCTGGTAATAAATCACTTTTAT     24868
              ************************************************************

LA2093        TTGATATTGTTTATTCGCAATCTCTTCCACCTATTACTTTAGCCAATGGGATCTAGACAA     24963
E42           TTGATATTGTTTATTCGCAATCTCTTCCACCTATTACTTTAGCCAATGGGATCTAGACAA     24964
HEINZ         TTGATATTGTTTATTCGCAATCTCTTCCACCTATTACTTTAGCCAATGGGATCTAGACAA     24928
              ************************************************************
```

```
LA2093      AACCAAAAGGGGTTGGAAAAGCCAAACCCCTATCTTCTGTCACCCTAGACTTTGTTTTTT    25023
E42         AACCAAAAGGGGTTGGAAAAGCCAAACCCCTATCTTCTGTCACCCTAGACTTTGTTCTTT    25024
HEINZ       AACCAAAAGGGGTTGGAAAAGCCAAACCCCTATCTTCTGTCACCCTAGACTTTGTTTTTT    24988
            ******************************************************** ***

LA2093      ATGTTCCTGGTTCTCCTTTTAATCTAGCATCTGTTAGTCATTTGATGAAATCCTGACATT    25083
E42         ATGTTCCTGGTTCTCCTTTTAATCTAGCATCTGTTAGTCATTTGATGAAATCCTGACATT    25084
HEINZ       ATGTTCCTGGTTCTCCTTTTAATCTAGCATCTGTTAGTCATTTGATGAAATCCTGACATT    25048
            ************************************************************

LA2093      GTAGCATAACTTTTTTTGATGATTTTTTTCTCATACAGGACCGCAGTACAGGACAGATTA    25143
E42         GTAGCATAACTTTTTTTGATGATTTTTTTCTCATACAGGACCGCAGTACAGGACAGATTA    25144
HEINZ       GTAGCATAACTTTTTTTGATGATTTTTTTCTCATACAGGACCGCAGTACAGGACAGATTA    25108
            ************************************************************

LA2093      TTGTAACACGACATGAATCACAAGGCCTTTACTATCTTACCTCTTCAAATTCCTTCACAG    25203
E42         TTGTAACACGACATGAATCACAAGGCCTTTACTATCTTACCTCTTCAAATTCCTTGACAG    25204
HEINZ       TTGTAACACGACATGAATCACAAGGCCTTTACTATCTTACCTCTTCAAATTCCTTGACAG    25168
            ******************************************************* ****

LA2093      CATGCTCCGTTACAGATTCCCCAGATCTAATTCACAAACGGCTGGACATCCGAGTATCGT    25263
E42         CATGCTCCGTTACAGATTCCCCAGATCTAATTCACAAACGGCTGGACATCCGAGTATCGT    25264
HEINZ       CATGCTCCGTTACAGATTCCCCAGATCTAATTCACAAACGGCTGGACATCCGAGTATCGT    25228
            ************************************************************

LA2093      TGTTATTCACATGATCTTCATCGTTACGTTATGTCCGCTGATGTTACATTTTTTGAGTCT    25323
E42         TGTTATTCACATGATCTTCATCGTTACGTTATGTCCGCTGATGTTACATTTTTTGAGTCT    25324
HEINZ       TGTTATTCACATGATCTTCATCGTTACGTTATGTCCGCTGATGTTACATTTTTTGAGTCT    25288
            ************************************************************

LA2093      CAGCCTTACTATACATCTTTTGATCATACTGATGTCTCTATGGTCTTACCCATACCTCAA    25383
E42         CAGCCTTACTATACATCTTTTGATCATACTGATGTCTCTATGGTCTTACCCATACCTCAA    25384
HEINZ       CAGCCTTACTATACATCTTTTGATCATACTGATGTCTCTATGGTCTTACCCATACCTCAA    25348
            ************************************************************

LA2093      GTTTTACATGTGCCAACATTTGAGGGATCTACAGTTACGTCTACATCTCCAGTTGCAGTG    25443
E42         GTTTTACATGTGCCAACCTTTGAGGGATCTACAGTTACGTCTACATCTCCAGTTGCAGTG    25444
HEINZ       GTTTTACATGTGCCAACATTTGAGGGATCTACAGTTACGTCTACATCTCCAGTTGCAGTG    25408
            ***************** ******************************************

LA2093      CCACCACTACTAACTTATCATCGCCGTCCACGTCCAACACTAGTCCCAGATGATTATGTC    25503
E42         CCATCACTACTAACTTATCATCGCCGTCCACGTCCAACCCTAGTCCCAGATGATTATGTC    25504
HEINZ       CCACCACTACTAACTTATCATCGCCGTCCACGTCCAACACTAGTCCCAGATGATTATGTC    25468
            *** ********************************** *********************

LA2093      ATGGGCCAGATCCTGCTTCTATTGTGGACTGCCTCTACCTAGCCCGCCGCTTGCACTTCA    25563
E42         ATGGGCCAGATCCTGCTTCTATTGCGGACTGCCTCTACCTAGCCCGCCGCTTGCACTTCA    25564
HEINZ       ATGGGCCAGATCCTGCTTCTATTGCGGACTGCCTCTACCTAGCCCGCCGCTTGCACTTCA    25528
            ************************ ***********************************

LA2093      AAAAGGTATACGATCTACTCGAAATACTAACCCGCATTATACCTTCTTAAGTTATCATCA    25623
E42         AAAAGGTATACGATCTACTTGAAATACTAACCCGCATTATACCTTCTTAAGTTATCATCA    25624
HEINZ       AAAAGGTATACGATCTACTCGAAATACTAACCCGCATTATACCTTCTTAAGTTATCATCA    25588
            ******************* ****************************************

LA2093      CCCCATTATGCCTTTGTGACTTCTTTATCCTCTGTTTCCATTTCTAAAACTACAGGTGAA    25683
E42         CCCCATTATGCCTTTGTGACTTCTTTATCCTCTGTTTCCATTTCTAAAACTACAGGTGAA    25684
HEINZ       CCCCATTATGCCTTTGTGACTTCTTTATCCTCTGTTTCCATTTCTAAAACTACAGGTGAA    25648
            ************************************************************

LA2093      GCACTTTCTCATTCTGGATGGAGGCAGGCTATGGTTGATGAGATGTCTACTTTACATAAG    25743
E42         GCACTTTCTCATTCTGGATGGAGGCAGGCTATGGTTGATGAGATGTCTACTTTACATAAG    25744
HEINZ       GCACTTTCTCATTCTGGATGGAGGCAGGCTATGGTTGATGAGATGTCTACTTTACATAAG    25708
            ************************************************************

LA2093      AGTGGTACTTGGGAGCTTGTCTCCCTTGTTACAGGTAAATCTACTATTAGTTGTCGTTGG    25803
E42         AGTGGTACTTGGGAGCTTGTCTCCCTTGTTACAGGTAAATCTACTATTGGTTGTCGTTGG    25804
HEINZ       AGTGGTACTTGGGAGCTTGTCTCCCTTGTTACAGGTAAATCTACTATTGGTTGTCGTTGG    25768
            ************************************************ ***********

LA2093      GTTTATGCAGTC-AAATTGGTCCAGATGGTCAGGTTGATCGACTTAAGGCTCGCCTTGTC    25862
E42         GTTTATGCAGTCAAAATTGGTCCAGATGGTCAGGTTGATCGACTTAAGGCTCGCCTTGTC    25864
```

```
HEINZ       GTTTATGCAGTCAAAATTGGTCCAGATGGTCAGGTTGATCGACTTAAGGCTCGCCTTGTC    25828
            ************ ***********************************************

LA2093      GCCAAAGGGTATGTATACTCAGATATTTGGGCTAGATTATAGTGACACTTTCGCTCCTGC    25922
E42         GCCAAAGGGTATGTATACTCAGATATTTGGGCTAGATTATAGTGACACTTTCGCTCCCGT    25924
HEINZ       GCCAAAGGGTATGTATACTCAGATATTTGGGCTAGATTATAGTGACACTTTCGCTCCTGC    25888
            ********************************************************* * 

LA2093      GGCTAAAATAGCATTTGTTCGTCTTTTTCTATCTATGGTTGTCGTTCGTCATTGGCCTCT    25982
E42         GGCTAAAATAGCATTTGTTCGTCTTTATCTATCTATGGTTGTCGTTCGTCATTGGCCTCT    25984
HEINZ       GGCTAAAATAGCATTTGTTCGTCTTTTTCTATCTATGGTTGTCGTTCGTCATTGGCCTCT    25948
            ************************** *********************************

LA2093      TTATCAGTTGCACATAAAGAATGCTTTTCTGCATAGTGATCTTGAGGAAGAAGTCTATAT    26042
E42         TTATCAGTTGCACATAAAGAATGCTTTTCTGCATAGTGATCTTGAGGAAGAAGTCTATAT    26044
HEINZ       TTATCAGTTGCACATAAAGAATGCTTTTCTGCATAGTGATCTTGAGGAAGAAGTCTATAT    26008
            ************************************************************

LA2093      GGAGCAACCACCTGGTTTTGTTGCTCAGGGGGAGTCTAGTAGCCTTGTATGTCGATTGTG    26102
E42         GGAGCAACCACCTGGTTTTGTTGCTCAGGGGGAGTCTAGTAGCCTTGTATGTCGATTGTG    26104
HEINZ       GGAGCAACCACCTGGTTTTGTTGCTCAGGGGGAGTCTAGTAGCCTTGTATGTCGATTGTG    26068
            ************************************************************

LA2093      TAGGTCACTCTATGGTCTGAAACAGTCTCCTCGAGCTTGGTTTGGGAAGTTCAGCATAGT    26162
E42         TAGGTCACTCTATGGTCTGAAACAGTCTCCTCGAGCTTGGTTTGGGAAGTTCAGCATAGT    26164
HEINZ       TAGGTCACTCTATGGTCTGAAACAGTCTCCTCGAGCTTGGTTTGGGAAGTTCAGCATAGT    26128
            ************************************************************

LA2093      AATTATGGAGTTTGGCATGACTCGTAGTGGAGCTAATCACTCTGTATTTTATCGACATTC    26222
E42         AATTATGGAGTTTGGCATGACTCGTAGTGGAGCTAATCACTCTGTATTTTATCGACATTC    26224
HEINZ       AATTATGGAGTTTGGCATGACTCGTAGTGGAGCTAATCACTCTGTATTTTATCGACATTC    26188
            ************************************************************

LA2093      TGCACAAGGTCGATGTATATATTTGGTTGTTTATGTTGATGATATTGTTATCACCGATAA    26282
E42         TGCACCAGGTCGATGTATATATTTGGTTGTTTATGTTGATGATATTGTTATCACCGATAA    26284
HEINZ       TGCACCAGGTCGATGTATATATTTGGTTGTTTATGTTGATGATATTGTTATCACCGATAA    26248
            ***** ******************************************************

LA2093      TGATCAAGATGGTATCACCGATTTAAAGCAACATCTTTTTAAGCACTTTCAGACTAAAGA    26342
E42         TGATCAAGATGGTATCACCGATTTAAAGCAACATCTTTTTAAGCACTTTCAGACTAAAGA    26344
HEINZ       TGATCAAGATGGTATCACCGATTTAAAGCAACATCTTTTTAAGCACTTTCAGACTAAAGA    26308
            ************************************************************

LA2093      CCTTGGCAGATTGAAGTATTTTTGGGGAATTGAGGTTGCTCAGTCTAGATCAGGCATTGT    26402
E42         CCTTGGCAGATTGAAGTATTTTTTGGGAATTGAGGTTGCTCAGTCTAGATCAGGCATTGT    26404
HEINZ       CCTTGGCAGATTGAAGTATTTTTGGGGAATTGAGGTTGCTCAGTCTAGATCAGGCATTGT    26368
            *********************** ************************************

LA2093      TATCTCTCAACGCAAGTATGCCTTAGACATTCTTGAGGAGACAGGAATGATGGGATGTAG    26462
E42         TATCTCTCAACGCAAGTATGCCTTAGACATTCTTGAGGAGACAGGAATGATGGGATGTAG    26464
HEINZ       TATCTCTCAACGCAAGTATGCCTTAGACATTCTTGAGGAGACAGGAATGATGGGATGTAG    26428
            ************************************************************

LA2093      ACCTATTGACACTCCGATGAATCCGAATGTTAAACTTCTTCCGAGACAAGGGGAGCCACT    26522
E42         ACCTATTGACACTCCGATGAATCCGAATGTTAAACTTCTTCCGAGACAAGGGGAGCCACT    26524
HEINZ       ACCTATTGACACTCCGATGAATCCGAATGTTAAACTTCTTCCGAGACAAGGGGAGCCACT    26488
            ************************************************************

LA2093      TAGTAATCCTGAAAGGTATAGACGACTTGTTGGAAAGTTGAATTATCTCACATTGACTAG    26582
E42         TAGTAATCCTGAAAGGTATAGACGACTTGTTGGAAAGTTGAATTATCTCACATTGACTAG    26584
HEINZ       TAGTAATCCTGAAAGGTATAGACGACTTGTTGGAAAGTTGAATTATCTCACATTGACTAG    26548
            ************************************************************

LA2093      ACCAGACATCTCTTTTCCCGTGAGTGTTGTAAGTCAGTTTATGACTTCCCCTTGTGATAG    26642
E42         ACCAGACATCTCTTTTCCCGTGAGTGTTGTAAGTCAGTTTATGACTTCCCCTTGTGATAG    26644
HEINZ       ACCAGACATCTCTTTTCCCGTGAGTGTTGTAAGTCAGTTTATGACTTCCCCTTGTGATAG    26608
            ************************************************************

LA2093      TCATTGGGAAGCAGTTGTTCGTATTCTTCGATATTTGTAACAAGCTAGGTGCATATGATT    26702
E42         TCATTGGGAAGCAGTTGTTCGTATTCTTCGATATTTGTAACAAGCTAGGTGCATATGATT    26704
HEINZ       TCATTGGGAAGCAGTTGTTCGTATTCTTCGATATTTGTAACAAGCTAGGTGCATATGATT    26668
            ************************************************************
```

```
LA2093      TGTATGCACCAACTTGAGGGGGAGTGTTAAAATAGGAATAAGTATTTCTTACTTAAAATA    26762
E42         TGTATGCACCAACTTGAGGGGGAGTGTTAAAATAGGAATAAGTATTACTTACTTAAAATA    26764
HEINZ       TGTATGCACCAACTTGAGGGGGAGTGTTAAAATAGGAATAAGTATTTCTTACTTAAAATA    26728
            ********************************************** *************

LA2093      TGAATTAGAATAGGAAAAAAATTAGGAATTCTAGTTGGAAAAGGTTTTCAATGTTGTGTC    26822
E42         TGAATTAGAATAGGAATAAAATTAGGAATTCTAGTTGGAAAAGGTTTTCAATGTTGTGTC    26824
HEINZ       TGAATTAGAATAGGAATAAAATTAGGAATTCTAGTTGGAAAAGGTTTTCAATGTTGTGTC    26788
            **************** *******************************************

LA2093      TATAAATAGGGTCTTCATGTAACAATTTAGATGTCCAATTCAATAATATTTTCTCCAATA    26882
E42         TATAAATAGGGTCTTCATGTAACAATTTAGATGTCCAATTCAATAATATTTTCTCCAATA    26884
HEINZ       TATAAATAGGGTCTTCATGTAACAATTTAGATGTCCAATTCAATAATATTTTCTCCAATA    26848
            ************************************************************

LA2093      TTTCTCACACCTTTTAAGACTATATTATGTATGTTGTCTATATGTGGCATAAAAGTAGAA    26942
E42         TTTCTCACACCTTTTAAGACTATATTATGTATGTTGTCTATATGTGGCATAAAAGTAGAA    26944
HEINZ       TTTCTCACACCTTTTAAGACTATATTATGTATGTTGTCTATATGTGGCATAAAAGTAGAA    26908
            ************************************************************

LA2093      GGCTATTTAATCTTCTTATACGAGGAGTCTATGTTTACTCTCTATGAATATATTATGTGT    27002
E42         GGCTATTTAATCTTCTTATACGAGGAGTCTATGTTTACTCTCTATGAATATATTATGTGT    27004
HEINZ       GGCTATTTAATCTTCTTATACGAGGAGTCTATGTTTACTCTCTATGAATATATTATGTGT    26968
            ************************************************************

LA2093      AAGCGAGAGGTCGAAGAAAACCTCATGAAGTAGATGTAGATCAAAAAGTTTTAATTTTAA    27062
E42         AAGCGAGAGGTCGAAGAAAACCTCATGAAGTAGATGTAGATCAAAAAGTTTTAATTTTAA    27064
HEINZ       AAGCGAGAGGTCGAAGAAAACCTCATGAAGTAGATGTAGATCAAAAAGTTTTAATTTTAA    27028
            ************************************************************

LA2093      GCACGTTTTTAACCTATGAATGTAATGACGGATGCTAAGAGGCTAGTCTTAGTCCTCTCC    27122
E42         GCACGTTTTTAACCTATGAATGTAATGACGAATGCTAAGAGGCTAGTCTTAGTCCTCTCC    27124
HEINZ       GCACGTTTTTAACCTATGAATGTAATGACGGATGTTAAGAGGCTAGTCTTAGTCCTCTCC    27088
            ****************************** *** *************************

LA2093      AAGGACAACGACGCCGGTTACGTCTAGGGGGTACTTCCCGGATGTGATAGATTGGCTCCG    27182
E42         AAGGACAACGACGCCGGTTACGTCTAGGGGGTACTTCCCGGATGTGATAGATTGGCTCCG    27184
HEINZ       AAGGACAACGACGCCGGTTACGTCTAGGGGGTACTTCCCGGATGTGATAGATTGGCTCCG    27148
            ************************************************************

LA2093      ACAGCTTTAAGTCAGGGATCATTTGATCTTTTTCCCTATACGGTGGACACCTAAACTATG    27242
E42         ACAGCTTTAAGTCAGGGATCATTTGATCTTTTTCCCTATACGATGGACACCTAAACTATG    27244
HEINZ       ACAGCTTTAAGTCAGGGATCATTTGATCTTTTTCCCTATACGGTGGACACCTAAACTATG    27208
            ****************************************** *****************

LA2093      TCGTTTAACCCTTAAACTACGTTGTTTTGATCAGTTCTAGTCTAGTAACTTAATCAGACT    27302
E42         TCGTTTAACCCTTAAACTACGTTGTTTTGATCAGTTCTAGTCTAGTAACTTAATCAGACT    27304
HEINZ       TCGTTTAACCCTTAAACTACGTTGTTTTGATCAGTTCTAGTCTAGTAACTTAATCAGACT    27268
            ************************************************************

LA2093      TACATTAAGCGATTCATTCACAAAAAAAATCTGAAATCTTAGAGCAAAAAGGGAGAAAGAG    27362
E42         TACATTAAGCGATTCATTCACAAAAAAAATCTGAAATCTTAGAGCAAAAAGGGAGAAAGAG    27364
HEINZ       TACATTAAGCGATTCATTCACAAAAAAAATCTGAAATCTTAGAGCAAAAAGGGAGAAAGAG    27328
            ************************************************************

LA2093      TCGACCAACCTCTCAATAACAAGCAAAAAGTTTCTTCAGTTCCAGCCTTGAAATCGAAAG    27422
E42         TCGACCAACCTCTCAAGAACAAGCAAAAGGTTTCTTCAGTTCCAGCCTTGAAATCGAAAG    27424
HEINZ       TCGACCAACCTCTCAATAACAAGCAAAAAGTTTCTTCAGTTCCAGCCTTGAAATCGAAAG    27388
            **************** *********** *******************************

LA2093      ATTTCTTTATGAAATTTGTCACCAGGTATTTGGGGTTTCACTAATGGGTTCCTTTCACCC    27482
E42         ATTTCTTTGTGAAATTTGTCACCGGGTATTTGGGGTTTCACTAATGGGTTCCTTTCACCC    27484
HEINZ       ATTTCTTTGTGAAATTTGTCACCAGGTATTTGGGGTTTCACTAATGGGTTCCTTTCACCC    27448
            ******** ************** ************************************

LA2093      ATTAGGTCCCTAGAATTCAGTCAAATTCTTGTTTCTCTATATCATGTTTAGACTTAGGGT    27542
E42         ATTAGGTCCCTAGAATTCAGTCAAATTCTTGTTTCTCTATATCATGTTTAGACTTAGGGT    27544
HEINZ       ATTAGGTCCCTAGAATTCAGTCAAATTCTTGTTTCTCTATATCATGTTTAGACTTAGGGT    27508
            ************************************************************

LA2093      TTCTAGAACTTCATATATTTGTTGTGATTTAGATACTAGAGTAATCCTAATCAAATTATT    27602
```

```
E42          TTCTAGAACTTCATATAATTGTTGTGATTTAGATACTAGAGTAATCCTAATCAAATTACT     27604
HEINZ        TTCTAGAACTTCATATAATTGTTGTGATTTAGATACTAGAGTAATCCTAATCAAATTACT     27568
             ***************** **************************************** *

LA2093       ATGTTTTCATAACTACGTTGATTAATCCTTGCATGAAATTCAGAACCCTAGTTTTGTAGA     27662
E42          ATGTTTTCATAACTACGTTGATTAATCCTTGCATGAAATTCAGAACCCTAGTTTTGTAGA     27664
HEINZ        ATGTTTTCATAACTACGTTGATTAATCCTTGCATGAAATTCAGAACCCTAGTTTTGTAGA     27628
             ************************************************************

LA2093       TTCGTTTAGTTCATCAATTACACTTTCTAGGCTAGTTTATGAAGATATATACATGCATCA     27722
E42          TTCGTTTAGTTCATCAATTACACTTGCTAGGCTAGTTTATGAAGATATATACATGCATCA     27724
HEINZ        TTCGTTTAGTTCATCAATTACACTTTCTAGGCTAGTTTATGAAGATATATACATGCATCA     27688
             ************************* **********************************

LA2093       ATTTTCAAATGTTTCATTATCAATACTTAATGTTGCATTTTTAGTTAAAATGTTAGAATC     27782
E42          ATTTTCAAATGTTTCATTATCAATACTTAATGTTGCATTTTTAGTTAAAATGTTAGAATC     27784
HEINZ        ATTTTCAAATGTTTCATTATCAATACTTAATGTTGCATTTTTAGTTAAAATGTTAGAATC     27748
             ************************************************************

LA2093       TTAAGCTATCCAGTGTATTGAATAGTACACATTCTAGTTGGGAGTAGGCTTAATATTGAG     27842
E42          TTAAGCTATCCAGTGTATTGAATAGTACACATTCTAGTTGGGAGTAGGCTTAATATTGAG     27844
HEINZ        TTAAGCTATCCAGTGTATTGAATAGTACACATTCTAGTTGGGAGTAGGCTTAATATTGAG     27808
             ************************************************************

LA2093       TTGGACTAGGGTCAGTCACCCTCATAATCCTAGAACTACGTGCCCCTATAGGTTAAGTTC     27902
E42          TTGGACTAGGGTCAGTCACCCTCATAATCCTAGAACTACGTGCCCCTATAGGTTAAGTTC     27904
HEINZ        TTGGACTAGGGTCAGTCACCCTCATAATCCTAGAACTACGTGCCCCTATAGGTTAAGTTC     27868
             ************************************************************

LA2093       CCTCTGTGGGCATCAATTTTAGTGATCACGCTAGCCATGCCTTTATACCCTAGCAAGGCA     27962
E42          CCTCTGTGGGCATCAATTTTAGTGATCACGCTAGCCATGCCTTTATACCCTAGCAAGGCA     27964
HEINZ        CCTCTGTGGGCATCAATTTTAGTGATCACGCTAGCCATGCCTTTATACCCTAGCAAGGCA     27928
             ************************************************************

LA2093       TATTGGGTATTCTCGATGAGGCGTATACATTGGACTCCACGTTTAGCTCACGTGGTTTTA     28022
E42          TATTGGGTATTCTCGATGAGGCGTATACATTGGACTCCACATTTAGCTCACGTGGTTTTA     28024
HEINZ        TATTGGGTATTCTCGATGAGGCGTATACATTGGACTCCACGTTTAGCTCACGTGGTTTTA     27988
             **************************************** *******************

LA2093       TGTCAGTTATTAGTAGATTAGTCAGATTCTAGTGCATTGACCAGGTTATCAATCACAATA     28082
E42          TGTCAGTTATTAGTAGATTAGTCAGATTCTAGTGCATTGACCAGGTTATCAATCACAATA     28084
HEINZ        TGTCAGTTATTAGTAGATTAGTCAGATTCTAGTGCATTGACCAGGTTATCAATCACAATA     28048
             ************************************************************

LA2093       TAGTATTCAATTTCAGCAAGTTATGATTTGGTGATTGCATTTAGTTTAGTTCATAATTAG     28142
E42          TAGTATTCAATTTCAGCAAGTTATGATTTGGTGATTGCATTTAGTTTAGTTCATAATTAG     28144
HEINZ        TAGTATTCAATTTCAGCAAGTTATGATTTGGTGATTGCATTTAGTTTAGTTCATAATTAG     28108
             ************************************************************

LA2093       TATGTTCTAATATATTCAATATTCATGATGTTCATCTGATATCATTGCTCAATATGATTT     28202
E42          TATGTTCTAATATATTAAATATTCATGATGTTCATCTGATATCATTGCTCAATATGATTT     28204
HEINZ        TATGTTCTAATATATTCAATATTCATGATGTTCATCTGATATCATTGCTCAATATGATTT     28168
             **************** *******************************************

LA2093       GTTCAATTATTTATATTGTCCAGCTTTATCCTATCGTGCATGCTCAATACCTTTCAAATA     28262
E42          GTTCAATTATTTATATTGTCCAGCTTTATCCTATCTTGCATGCTCAATACCTTTCAAATA     28264
HEINZ        GTTCAATTATTTATATTGTCCAGCTTTATCCTATCGTGCATGCTCAATACCTTTCAAATA     28228
             *********************************** ************************

LA2093       CTGACACATACTTAGCGCATCTTCTTGTGATGTAGGTTCAGGCCTACAGCATTCTGATCG     28322
E42          CTGACACATACTTAGCGCATCTTCTTGTGATGTAGGTTCATGCCTACAGCATTCTGATCG     28324
HEINZ        CTGACACATACTTAGCGCATCTTCTTGTGATGTAGGTTCAGGCCTACAGCATTCTGATCG     28288
             **************************************** *******************

LA2093       CGTGTAGATCGGTTCCTAATCCATATACAGCATTATCAGTGGTGAGTTCTCATTCTTTGA     28382
E42          CGTGTAGATCGGTTCCTAATCCATATACAGCATTATCAGTGGTGAGTTCTCATTCTTCGA     28384
HEINZ        CGTGTAGATCGGTTCCTAATCCATATACAGCATTATCAGTGGTGAGTTCTCATTCTTCAA     28348
             *********************************************************  *

LA2093       GGACGATGGACATGTTTTCGTTATTTTTCTTCTTTCAGTTTCAGTTTTGCTAGAGTTAGC     28442
E42          GGACGATGGACATGTTTTCGTTATTTTTCTTCTTTCAGTTTCAGTTTTGCTAGAGTTAGC     28444
HEINZ        GGACGATGGACATGTTTTCGTTATTTTTCTTCTTTCAGTTTCAGTTTTGCTAGAGTTAGC     28408
```

```
            ************************************************************

LA2093      AGGGCATGTCCCAGCATCTCTAGTCAGTTAGATGTTTATTTAAGACATAGCTAAAATCAA    28502
E42         AGGGTATGTCCCAGCATCTCTAGTCAGTTAGATGTTTATTTAAGACATAGCTAAAATCAA    28504
HEINZ       AGGGCATGTCCCAGCATCTCTAGTCAGTTAGATGTTTATTTAAGACATAGCTAAAATCAA    28468
            **** *******************************************************

LA2093      TTTTAGCTTTGAGTTTGATAATTCAATTGTTATTGAACTTACAATTTACATATTCACACA    28562
E42         TTTTAGCTTTGAGTTTGATAATTCAATTGTTATTGAACTTACAATTTACATATTCACACA    28564
HEINZ       TTTTAGCTTTGAGTTTGATAATTCAATTGTTATTGAACTTACAATTTACATATTCACACA    28528
            ************************************************************

LA2093      GTGTTGGGTATTCCTCATTTCACTTAATATGATGGTTTTAAGCTTCCGCACAATATTTCT    28622
E42         GTGTTAGGTATTCCTCATTTCACTTAATATGATGGTTTTAAGCTTCCGCACAATATTTCT    28624
HEINZ       GTGTTGGGTATTCCTCATTTCACTTAATATGATGGTTTTAAGCTTCCGCACAATATTTCT    28588
            ***** ******************************************************

LA2093      TAATTCAGTTTATCTTTAAGTATACCTTTGATATGCAAATTCAGGGTTAGCTTGGGGTCA    28682
E42         TAATTCAGTTTATCTTTAAGTATACCTTTGATATGCAAATTCAGGGTTAGCTTGGGGTCA    28684
HEINZ       TAATTCAGTTTATCTTTAAGTATACCTTTGATATGCAAATTCAGGGTTAGCTTGGGGTCA    28648
            ************************************************************

LA2093      CTCGTGATCCTAGGTCCCGTGTTACGTCAAGGGGGTAGCCTCGGGGCGTTGCATGGCTCT    28742
E42         CTCGTGATCCTAGGTCCCGTGTTACGTCAAGGGGGTAGCCTCGGGGCGTTGCATGGCTCT    28744
HEINZ       CTCGTGATCCTAGGTCCCGTGTTACGTCAAGGGGGTAGCCTCGGGGCGTTGCATGGCTCT    28708
            ************************************************************

LA2093      CAACCACTTCATTGAACATTGGGCCACACCCTTAGGTGCCATTAAGAATGTTTTTCTACA    28802
E42         CAACCACTTCATTGAACATTGGGCCACACCCTTAGGTGCCATTAAGAATGTTTTTCTACA    28804
HEINZ       CAACCACTTCATTGAACATTGGGCCACACCCTTAGGTGCCATTAAGAATGTTTTTCTACA    28768
            ************************************************************

LA2093      TAGTGATCTTGAGGGAAAAGTCTATATGGAGAAACTACCTACTTATGTTGCTTAAGGGGA    28862
E42         TAGTGATCTTGAGGGAAAAGTCTATATGGAGAAACTACCTACTTATGTTGCTTAAGGGGA    28864
HEINZ       TAGTGATCTTGAGGGAAAAGTCTATATGGAGAAACTACCTACTTATGTTGCTTAAGGGGA    28828
            ************************************************************

LA2093      ATTTAGAAGCCTTGTATGTCGACAATTTTGAATAGTAACCACGAGTTTAGTTTGGAAAGC    28922
E42         ATTTAGAAGCCTTGTATGTCGACAATTTTGAATAGTAACCACGAGCTTAGTTTGGAAAGT    28924
HEINZ       ATTTAGAAGCCTTGTATGTCGACAATTTTGAATAGTAACCACGAGTTTAGTTTGGAAAGT    28888
            ********************************************* ************* 

LA2093      TCGCATAGTACTTCAAGATTTTGGCATAACCCATTGTGGATTTGATCACTATGTTTTATC    28982
E42         TCGCATAGTACTTCAAGATTTTGGCATAACCCATTGTGGATTTGATCACTATGCTTTATC    28984
HEINZ       TCGCATAGTACTTCAAGATTTTGGCATAACCCATTGTGGATTTGATCACTATGTTTTATC    28948
            ***************************************************** ******

LA2093      AACATTCTACACCGAATCTATATATTTATTTGGTGATTTATGTTGACAATAACATTACCA    29042
E42         AACATTCTACACCGAATCTATATATTTATTTGGTGATTTATGTTGACAATAACATTACCA    29044
HEINZ       AACATTCTACACCGAATCTATATATTTATTTGGTGATTTATGTTGACAATAACATTACCA    29008
            ************************************************************

LA2093      CATGCAATAAACAAAATGGTATCACCAATTTGAAGCAACATCTCTTTCACCAATTCTGTA    29102
E42         CATGCAATAAACAAAATGGTATCACCAATTTGAAGCAACATCTCTTTCACCAATTCTGTA    29104
HEINZ       CATGCAATAAACAAAATGGTATCACCAATTTGAAGCAACATCTCTTTCACCAATTCTGTA    29068
            ************************************************************

LA2093      CTAATGACCTCGGCAACATGAAGTATAATCTTGGTATTGAAGTTGTTCAATCCAAATAAA    29162
E42         CTAATGACCTCGGCAACATGAAGTATAATCTTGGTATTGAAGTTGTTCAATCCAAATAAA    29164
HEINZ       CTAATGACCTCGGCAACATGAAGTATAATCTTGGTATTGAAGTTGCTCAATCCAAATAAA    29128
            ********************************************* **************

LA2093      GTATGGCTTGGACATTCTTGAGGAGACAAAAATGATAGGTACCTATTGACACTCCTATGG    29222
E42         GTATGGCTTGGACATTCTTGAGGAGACAAAAATGATAGGTACCTATTGACACTCCTATGG    29224
HEINZ       GTATGGCTTGGACATTCTTGAGGAGACAAAAATGATAGGTACCTATTGACACTCCTATGG    29188
            ************************************************************

LA2093      ATCCAAATGCTAACCCCTACAATAGTAGGGGAGCCACTTAGTGATCTTGGAAGGTATAGG    29282
E42         ATCCAAATGCTAACCCCTACAATAGTAGGGGAGCCACTTAGTGATCTTGGAAGGTATAGG    29284
HEINZ       ATCCAAATGCTAACCCCTACAATAGTAGGGGAGCCACTTAGTGATCTTGGAAGGTATAGG    29248
            ************************************************************
```

```
LA2093      TTGGAAAGTTCAATTATCCCAGAGTGGCTAGACCGGATATCTCTTTTACTGTGAGTGTGT     29342
E42         TTGGAAAGTTCAATTATCCCAGAGTGGCTAGACCGGATATCTCTTTTACTGTGAGTGTGT     29344
HEINZ       TTGGAAAGTTCAATTATCCCAGAGTGGCTAGACCGGATATCTCTTTTACTGTGAGTGTGT     29308
            ************************************************************

LA2093      TATAAGTAAGTGTATGACTTCCGCTGTGATTTTATTGGGATGCAGTTGTTTGAATTTTGT     29402
E42         TATAAGTCAGTTTATGACTTCCGCTGTGATTTTATTGGGATGCAGTTGTTTGAATTTTGT     29404
HEINZ       TATAAGTAAGTGTATGACTTCCGCTGTGATTTTATTGGGATGCAGTTGTTTGAATTTTGT     29368
            ******* *** ************************************************

LA2093      GACAGATAAAGTTAGATCCAAGTAAAGGCCTACTCTTGGAGGATCGAGGTCATGAGCATA     29462
E42         GACAGATAAAGTTAGATCCAAGTAAAGGCCTACTCTTGGAGGATCGAGGTCATGAGCATA     29464
HEINZ       GACAGATAAAGTTAGATCCAAGTAAAGGCCTACTCTTGGAGGATCGAGGTCATGAGCATA     29428
            ************************************************************

LA2093      TCATTGAATATACAAACGTTCATTATAC-AGAGCACCCTCTGATAGACATTTTATATCCA     29521
E42         TCATTGAATATACAAACGTTCATTATACAAGAGCACCCTCTGATAGACATTTTATATCCA     29524
HEINZ       TCATTGAATATACAAACGTTCATTATAC-AGAGCACCCTCTGATAGACATTTTATATCCA     29487
            **************************** *******************************

LA2093      AATATTGGGTTTTAGTAGGAAGTAATTTGGTGTTCTACAAAAGCAAGAAACAGAGTGTGG     29581
E42         AATATTGGGTTTTAGTAGAAAGTAATTTGGTGTTCTACAAAAGCAAGAAACAGAGTGTGG     29584
HEINZ       AATATTGGGTTTTAGTAGGAAGTAATTTGGTGTTCTACAAAAGCAAGAAACAGAGTGTGG     29547
            ****************** *****************************************

LA2093      TTGCTTGAAGCATAATATCAAGCAATGGTTGTAGCAACTTGTGAGCTAGTTTGGATCAAA     29641
E42         TTGCTTGAAGCATAATATCAAGCAATGGTTGTAGCAACTTGTGAGCTAGTTTGGATCAAA     29644
HEINZ       TTGCTTGAAGCATAATATCAAGCAATGGTTGTAGCAACTTGTGAGCTAGTTTGGATCAAA     29607
            ************************************************************

LA2093      CAATTGTTGAAGAATTAAAATTTGGAGAAATCAATCATATGGAATTTGTGTATGATAATC     29701
E42         CAATTGTTGAAGAATTAAAATTTGGAGAAATCAATCATATGGAATTTGTGTATGATAATC     29704
HEINZ       CAATTGTTGAAGAATTAAAATTTGGAGAAATCAATCATATGGAATTTGTGTATGATAATC     29667
            ************************************************************

LA2093      AACCAGGCTTTCATATCGCACCAAATTAAGTATTTCACGAAAAGGCTAAGCACATTGAGA     29761
E42         AACCAGGCTTTCATATCGCACCAAATTAAGTATTTCACGAAAGGGCTAAGCACATTGAGA     29764
HEINZ       AACCAGGCTTTCATATCGCACCAAATTAAGTATTTCACGAAAAGGCTAAGCACATTGAGA     29727
            ****************************************** *****************

LA2093      TTGATTGTCACCTTGTAAGAGAAAAGATACTCTTAGGAGATATTGTTACAAAATTTGTGA     29821
E42         TTGATTGTCACTTTGTAAGAGAAAAGATACTCTTAGGAGATATTGTTACAAAATTTGTGA     29824
HEINZ       TTGATTGTCACCTTGTAAGAGAAAAGATACTCTTAGGAGATATTGTTACAAAATTTGTGA     29787
            *********** ************************************************

LA2093      ATTCAAGTGATGAACCTTCAAATATATTAATCGAGTTCTTCACTGGTTCTCATATCAATT     29881
E42         AGTCAAGTGATGAACCTTCAAATATATTAATCGAGTTCTTCACTGGTTCTCATATCAATT     29884
HEINZ       ATTCAAGTGATGAACCTTCAAATATATTAATCGAGTTCTTCACTGGTTCTCATATCAATT     29847
            * **********************************************************

LA2093      ACATCTGCAAAAAGCCTGGTACACAGAACTTATATGCACCCGCTTGAGAGGATTGTTTGA     29941
E42         ACATCTGCAAAAAGCCTGGTACACAGAACTTATATGCACCCGCTTGAGAGGATTGTTTGA     29944
HEINZ       ACATCTGCAAAAAGCCTGGTACACAGAACTTATATGCACCCGCTTGAGAGGATTGTTTGA     29907
            ************************************************************

LA2093      ATAGGAATAGGAACAAGAAATCATATAGAATGCTACTTGGGAAAGGATTTGTAATATAGC     30001
E42         ATAGGAATAGGAACAAGAAATCATATAGAATGCTACTTGGGAAAGGATTTGTAATATAGC     30004
HEINZ       ATAGGAATAGGAACAAGAAATCATATAGAATGCTACTTGGGAAAGGATTTGTAATATAGC     29967
            ************************************************************

LA2093      ATCTATAAATAGTATCTCAATTTAATAATATAGATACACAATTCAATAATATTTATATTT     30061
E42         ATCTATAAATAGTATCTCAATTTAATAATATAGATACACAATTCAATAATATTTATATTT     30064
HEINZ       ATCTATAAATAGTATCTCAATTTAATAATATAGATACACAATTCAATAATATTTATATTT     30027
            ************************************************************

LA2093      CTCACATGGGCTTATTAATAAACAGTGTTGACTGTTTAGAATGTGTAAATTAGTTTCTTA     30121
E42         CTCACATGGGCTTATTAATAAACAGTGTTGACTGTTTAGAATGTGTAAATTAGTTTCTTA     30124
HEINZ       CTCACATGGGCTTATTAATAAACAGTGTTGACTGTTTAGAATGTGTAAATTAGTTTCTTA     30087
            ************************************************************

LA2093      AGTAATCATTAGAACAGTTGAACTCTTTTTCAAATCTAATAAAGGGTTTGTTTATTATTT     30181
E42         AGTAATCATTAGAACAGTTGAACTCTTTTTCAAATCTAATAAAGGGTTTGTTTATTATTT     30184
```

```
HEINZ      AGTAATCATTAGAACAGTTGAACTCTTTTTCAAATCTAATAAAGGGTTTGTTTATTATTT    30147
           ************************************************************

LA2093     TTTAAAAAAATTAAGTTGGTGCACTTTACATGGGTACAAAAGAAAAATGTTGTACTTTAT    30241
E42        TTAAAAAAAATTAAGTTGGTGCACTTTACATGGGTACAAAAGAAAAATGTTGTACTTTAT    30244
HEINZ      TTTAAAAAAATTAAGTTGGTGCACTTTACATGGGTACAAAAGAAAAATGTTGTACTTTAT    30207
           ** *********************************************************

LA2093     ATTTATTAGATTAGTTTTGCTAAGTGGTGTCCTCCCCTAGGAGGGTGTTGTACAAATGCT    30301
E42        ATTTATTAGATTAGTTTTGCTAAGTGGTGTCCTCCCCTGGGAGGGTGTTGTACAAATGCT    30304
HEINZ      ATTTATTAGATTAGTTTTGCTAAGTGGTGTCCTCCCCTAGGAGGGTGTTGTACAAATGCT    30267
           ************************************** *********************

LA2093     ACAGCCATCATAGGCATTGGCCTTATGTCCTTAAATTCATATTGAAGAGTAAGAC----G    30357
E42        ACAGCCATCATAGGCATTGGCCTTATGTCCTTAAATTCATATTGAAGAGTAAGACTTGTG    30364
HEINZ      ACAGCCATCATAGGCATTGGCCTTATGTCCTTAAATTCATATTGAAGAGTAAGAC----G    30323
           *******************************************************    *

LA2093     TGTATGAAGCATCCTGCGTTCACGCAGGATCCACGAAAGGAATAAAAAGTCATTTCATGA    30417
E42        TGTATGAAGCATCCTGCGTTCACGCAGGATCCACGAAAGGAATAAAAAGTCATTTCGTGA    30424
HEINZ      TGTATGAAGCATCCTGCGTTCACGCAGGATCCACGAAAGGAATAAAAAGTCATTTCATGA    30383
           ******************************************************** ***

LA2093     AAATTATAGAACACAAATTCATGTATTCAAGTGATATTTTTAGTGAAGGGGTATGAACTG    30477
E42        AAATTATAGAACACAAATTCATGTATTCAAGTGATATTTTTAGTGAAGGGGTATGAACTG    30484
HEINZ      AAATTATAGAACACAAATTCATGTATTCAAGTGATATTTTTAGTGAAGGGGTATGAACTG    30443
           ************************************************************

LA2093     ACCTGTTACCAAAGCACGAATGTCGAGCAGAGATGCTTATGTTTATTGTGCGAATGTTAT    30537
E42        ACCTGTTACCAAAGCACGAATGTCGAGCAGAGATGCTTATGTTTATTGTGCGAATGTTAT    30544
HEINZ      ACCTGTTACCAAAGCACGAATGTCGAGCAGAGATGCTTATGTTTATTGTGCGAATGTTAT    30503
           ************************************************************

LA2093     GGCGCAATCTAGCTTCCTCGGGCAGTAACAAGTACCCCTAAAAATAAATGGTAATACCCA    30597
E42        GGCGCAATCTAGCTTCCTCGGGCAGTAACAAGTACCCCTAAAAATAAATGGTAATACCCA    30604
HEINZ      GGCGCAATCTAGCTTCCTCGGGCAGTAACAAGTACCCCTAAAAATAAATGGTAATACCCA    30563
           ************************************************************

LA2093     CGAATTTACAAAAAGACAAACTTTAGCTTTTCATCAACAACTGGAAAAGATGCATAAATT    30657
E42        CGAATTTACAAAAAGACAAACTTTAGCTTTTCATCAACAACTGGAAAAGATGCATAAATT    30664
HEINZ      CGAATTTACAAAAAGACAAACTTTAGCTTTTCATCAACAACTGGAAAAGATGCATAAATT    30623
           ************************************************************

LA2093     AAATACAGTAAGGACCACAAAGCAATAGTAGTACCTTCTCTTTGGTGTATAAATAATTAG    30717
E42        AAATACAGTAAGGACCACAAAGCAATAGTAGTACCTTTTCTTTGGTGTATAAATAATTAG    30724
HEINZ      AAATACAGTAAGGACCACAAAGCAATAGTAGTACCTTCTCTTTGGTGTATAAATAATTAG    30683
           ************************************* **********************

LA2093     GTTCAAATGCATTGCTTCTTGTCTCAAGCCACTGCACTACCAGGTCAAATAAAGATCATA    30777
E42        GTTCAAATGCATTGCTTCTTGTCTCAAGCCACTGCACTACCAGGTCAAATAAAGATCATA    30784
HEINZ      GTTCAAATGCATTGCTTCTTGTCTCAAGCCACTGCACTACCAGGTCAAATAAAGATCATA    30743
           ************************************************************

LA2093     ACCATCACAAGATAATTCAACAAAACAACCAAAAACAGTTAAATAACTAAGGGGTCGTTT    30837
E42        ACCATCACAAGATAATTCAACAAAACAACCAAAAACAGTTAAATAACTAAGGGGTCGTTT    30844
HEINZ      ACCATCACAAGATAATTCAACAAAACAACCAAAAACAGTTAAATAACTAAGGGGTCGTTT    30803
           ************************************************************

LA2093     GGTAGAGTGTATAAGAATAATGTTAAATAGAGTGTATTAGTAATGCTAGCATTAGCAATG    30897
E42        GGTAGAGTGTATAAGAATAATGTTAAATAGAGTGTATTAGTAATGCTAGCATTAGCAATG    30904
HEINZ      GGTAGAGTGTATAAGAATAATGTTAAATAGAGTGTATTAGTAATGCTAGCATTAGCAATG    30863
           ************************************************************

LA2093     CATGTATTAGTTATGTTTGCATTAATTATACATATATTATTTTTATGCATCATTTGGTTT    30957
E42        CATGTATTAGTTATGTTTGCATTAATTATACATATATTATTTTTATGCATCATTTGGTTT    30964
HEINZ      CATGTATTAGTTATGTTTGCATTAATTATACATATATTATTTTTATGCATCATTTGGTTT    30923
           ************************************************************

LA2093     GATGCATAAAAAAATAGCATGCATTGCATAATAAATTTAATTTACAAAGATAACCTCCAC    31017
E42        GATGCATAAAAAAATAGCATGCATTGCATAATAAATTTAATTTACAAAGATAACCTCCAC    31024
HEINZ      GATGCATAAAAAAATAGCATGCATTGCATAATAAATTTAATTTACAAAGATAACCTCCAC    30983
           ************************************************************
```

```
LA2093      TATTATGGTGGAAAGGATGTAAAAAAAGGTTTTAAGATGCAATTGGTCTTTAATCATGCT     31077
E42         TATTATGGTGGAAAGGATGTAAAAAAAGGTTTTAAAATGCAATTGGTCTTTAATCATGCT     31084
HEINZ       TATTATGGTGGAAAGGATGTAAAAAAAGGTTTTAAGATGCAATTGGTCTTTAATCATGCT     31043
            *********************************** ************************

LA2093      AATGCATGCATTAAAATCATATCTAGAAATCAATGGTATTAGCAATATACATCGTAATAC     31137
E42         AATGCATGCATTAAAATCATATCTAGAAATCAATGGTATTAGCAATATACATCGTAATAC     31144
HEINZ       AATGCATGCATTAAAATCATATCTAGAAATCAATGGTATTAGCAATATACATCGTAATAC     31103
            ************************************************************

LA2093      ACTATATAGTGTTATACATAGGTTGGAAAAGAGTACCAAACAAGGTACTAGTAATACACA     31197
E42         ACTATAGAGTGTTATACATAGGTTGGAAAAGAGTACCAAACAAGGTACTAGTAATACACA     31204
HEINZ       ACTATATAGTGTTATACATAGGTTGGAAAAGAGTACCAAACAAGGTACTAGTAATACACA     31163
            ****** *****************************************************

LA2093      AGGCTAATGCATGCATTATTTTTTCTAAGAAACTCTACCAAACGACCCCTAACTCGTGAA     31257
E42         AGGCTAATGCATGCATTATTTTTTCTAAGAAACTCTACCAAACGACCCCTAACTCGTGAA     31264
HEINZ       AGGCTAATGCATGCATTATTTTTTCTAAGAAACTCTACCAAACGACCCCTAACTCGTGAA     31223
            ************************************************************

LA2093      TTTGATCCTTCAATAACTATAACAGCCTCAGTCAGCTCAAGTAACAAAAGTAATATTACA     31317
E42         TTTGATCCTTCAATAACTATAACAGCCTCAGTCAGCTCAAGTAACAAAAGTAATATTACA     31324
HEINZ       TTTGATCCTTCAATAACTATAACAGCCTCAGTCAGCTCAAGTAACAAAAGTAATATTACA     31283
            ************************************************************

LA2093      GGTATGCCTTTGACTTTTATAGAGATACATAAATATCAAGGAATCCAAAGCCATACATTA     31377
E42         GGTATGCCTTTGACTTTTATAGAGATACATAAATATCAAGGAATCCAAAGCCATACATTA     31384
HEINZ       GGTATGCCTTTGACTTTTATAGAGATACATAAATATCAAGGAATCCAAAGCCATACATTA     31343
            ************************************************************

LA2093      AGATTATAGGTGCTTGACGACAGATCTGACAGGCAATGGTCTTTAAAATGTAGGTCAGCC     31437
E42         AGATTATAGGTGCTTGACGACAGATCTGACAGGCAATGGTCTTTAAAATGTAGGTCAGCC     31444
HEINZ       AGATTATAGGTGCTTGACGACAGATCTGACAGGCAATGGTCTTTAAAATGTAGGTCAGCC     31403
            ************************************************************

LA2093      CATGAAAATAGTCCAATTCATATATTTCCTCTCCAAGTCTTCGCTAATTCTTCCCCCTCT     31497
E42         CATGAAAATAGTCCAATTCATATATTTCCTCTCCAAGTCTTCGCTAATTCCTCCCCCTCT     31504
HEINZ       CATGAAAATAGTCCAATTCATATATTTCCTCTCCAAGTCTTCGCTAATTCTTCCCCCTCT     31463
            ************************************************** *********

LA2093      ACTTCAAACGAGTGTTTCTTTCCCCTTTTCCTACTCCTTTATATTGATGATGGGAGTTTT     31557
E42         ACTTCAAACGAGTGTTTCTTTCCCCTTTTCCTACTCCTTTATATTGATGATGGGAGTTTT     31564
HEINZ       ACTTCAAACGAGTGTTTCTTTCCCCTTTTCCTACTCCTTTATATTGATGATGGGAGTTTT     31523
            ************************************************************

LA2093      CCATTCCCATCATTGTATTAACACGAACAAAGCACGAGGAAATAGGGACACAAGTATAAC     31617
E42         CCATTCCCATCATTGTATTAACACGAACAAAGCACGAGGAAATAGGGACACAAGTATAAC     31624
HEINZ       CCATTCCCATCATTGTATTAACACGAACAAAGCACGAGGAAATAGGGACACAAGTATAAC     31583
            ************************************************************

LA2093      AAGCAACAAAATATACCTAGTGTGATCAGGTACAAGTATATCAAGCTCCATATAAATTTG     31677
E42         AAGCAACAAAATATACCTAGTGTGATCAGGTACAAGTATATCAAGCTCCATATAAATTTG     31684
HEINZ       AAGCAACAAAATATACCTAGTGTGATCAGGTACAAGTATATCAAGCTCCATATAAATTTG     31643
            ************************************************************

LA2093      GTGGGTGGTTTAAGTCTTTTAGAGAGGGGGGCTGGGAGGGGGGGGTCACAAGCCTTTAGTTG     31737
E42         GTGGGTGGTTTAAGTCTTTTAGAGGGGGGGGCTGGGAGGGGGGGGTCACAAGCCTTTAGTTG     31744
HEINZ       GTGGGTGGTTTAAGTCTTTTAGAGAGGGGGGCTGGGAGGGGGGGGTCACAAGCCTTTAGTTG     31703
            ************************ ***********************************

LA2093      CACCTACATTGCATCCAATGTACCTGAACATCAGATTGGCCAGCTTTATCTGATTCAAGC     31797
E42         CACCTACATTGCATCCAATGTACCTGAACATCAGATTGGCCAGCTTTATCTGATTCAAGC     31804
HEINZ       CACCTACATTGCATCCAATGTACCTGAACATCAGATTGGCCAGCTTTATCTGATTCAAGC     31763
            ************************************************************

LA2093      TGAATTTCGTTACTACATATAATGTTTAATTACTCTATACATCACTAACAAACCACTAGA     31857
E42         TGAATTTCGTTACTACATATAATGTTTAATTACTCTATACATCACTAACAAACCACTAGA     31864
HEINZ       TGAATTTCGTTACTACATATAATGTTTAATTACTCTATACATCACTAACAAACCACTAGA     31823
            ************************************************************

LA2093      ACGGGAGCCAAAGGAAAAGAAGGGGGTTATAAGTGTAGAATCAGAAGATGAAAATTTATT     31917
```

```
E42           ACGGGAGCCAAAGGAAAAGAAGGGGGTTATAAGTGTAGAATTAGAAGATGAAAATTTATT    31924
HEINZ         ACGAGAGCCAAAGGAAAAGAAGGGGGTTATAAGTGTAGAATCAGAAGATGAAAATTTATT    31883
              *** ************************************ *******************

LA2093        TTCCGCTAAAAATGTATCAAAATAGACATATTAGAAACTCTAACATCTCAAAACATTTTA    31977
E42           TTCCGCTAAAAATGTATCAAAATAGACATGTTAGAAACTCTAACATCTCAAAACATTTTA    31984
HEINZ         TTCCGCTAAAAATGTATCAAAATAGACATATTAGAAACTCTAACATCTCAAAACATTTTA    31943
              ***************************** ******************************

LA2093        ACCTAATAGATGTATGTCAAATTAAGGCCTACTTGAATGAAGTGCCTTCACACAAAATAA    32037
E42           ACCTAATAGATGTATGTCAAATTAAGGCCTACTTGAATGAAGTGCCTTCACACAAAATAA    32044
HEINZ         ACCTAATAGATGTATGTCAAATTAAGGCCTACTTGAATGAAGTGCCTTCACACAAAATAA    32003
              ************************************************************

LA2093        GTACCCAAGTCTGTCATATAGTGGCCTCTATATAGTTTGCAAAATAAGCATCTTGTACGT    32097
E42           GTACCCAAGTCTGTCATATAGTGGCCTCTATATAGTTTGCAAAATAAGCATCTTGTACGT    32104
HEINZ         GTACCCAAGTCTGTCATATAGTGGCCTCTATATAGTTTGCAAAATAAGCATCTTGTACGT    32063
              ************************************************************

LA2093        AAGTAGAAAACCAAGAATGATAGCTACAATTCATCAAAACTACAAATGATAGAATGGAGG    32157
E42           AAGTAGAAAACCAAGAATGATAGCTACAATTCATCAAAACTACAAATGATAGAATGGAGG    32164
HEINZ         AAGTAGAAAACCAAGAATGATACCTACAATTCATCAAAACTACAAATGATAGAATGGAGG    32123
              ********************** *************************************

LA2093        GTAGCAGAGCCGGATGATAATGGTTCAACTCTTAGAAACAAACAAATTAAACCAACCACG    32217
E42           GTAGCAGAGCCGGATGATAATGGTTCAACTCTTAGAAACAAACAAATTAAACCAACCACG    32224
HEINZ         GTAGCAGAGCCGGATGATAATGGTTCAACTCTTAGAAACAAACAAATTAAACCAACCACG    32183
              ************************************************************

LA2093        GCAAGAAGTTTAAAAAATTAATTTCCCAACAAAATTATGCGCAAAAGATTAGGTTGTTAT    32277
E42           GCAAGAAGTTTAAAAAATTAATTTCCCAACAAAATTATGCGCAAAAGATTAGGTTGTTAT    32284
HEINZ         GCAAGAAGTTTAAAAAATTAATTTCCCAACAAAATTATGCGCAAAAGATTAGGTTGTTAT    32243
              ************************************************************

LA2093        GTTTTTTTGAAATATAAAAATAAATAAAAGTATCTTGCGATGTTTTCTTTAAGGAAAATC    32337
E42           GTTTTTTTGAAATATAAAAATAAATAAAAGTATCTTGCGATGTTTTCTTTAAGGAAAATC    32344
HEINZ         GTTTTTTTGAAATATAAAAATAAATAAAAGTATCTTGCGATGTTTTCTTTAAGGAAAATC    32303
              ************************************************************

LA2093        AAGCTAACTAATGTAAGAACATTGCACCAAAAGGGTGTGGCCTATTGGTCTATAAGGTGG    32397
E42           AAGCTAACTAATGTAAGAACATTGCACCAAAAGGGTGTGGCCTATTGGTCTATAAGGTGG    32404
HEINZ         AAGCTAACTAATGTAAGAACATTGCACCAAAAGGGTGTGGCCTATTGGTCTATAAGGTGG    32363
              ************************************************************

LA2093        TTGAGAACCCCAAGGTTTCAGGTTCGAAAATCAACAGAAACAAGAAAACACTCCCATCTG    32457
E42           TTGAGAACCCCAAGGTTTCAGGTTCGAAAATCAACAGAAACAAGAAAACACTCCCATCTG    32464
HEINZ         TTGAGAACCCCAAGGTTTCAGGTTCGAAAATCAACAGAAACAAGAAAACACTCCCATCTG    32423
              ************************************************************

LA2093        TCCAAGCCTTAATGGCAGAGTTACCTGGTACTTGTTGGTGGTGGGAGGTGACAGGTATCT    32517
E42           TCCAAGCCTTAATGGCAGAGTTACCTGGTACTTGTTGGTGGTGGGAGGTGACAGGTATCT    32524
HEINZ         TCCAAGCCTTAATGGCAGAGTTACCTAGTACTTGTTGGTGGTGGGAGGTGACAGGTATCT    32483
              ************************** *********************************

LA2093        CGTGGAATTAGTCAAGGTGCACGAAAGTTGATCCGAACAACACGGTTATCAAAAAAAATT    32577
E42           CGTGGAATTAGTCGAGGTGCACGAAAGTTGATCCGAACAACACGGTTATCAAAAAAAATT    32584
HEINZ         CGTGGAATTAGTCAAGGTGCACGAAAGTTGATCCGAACAACACGGTTATCAAAAAAAATT    32543
              ************* **********************************************

LA2093        AAGAACATTCTAAACAATATATTTACTACAAAAGCCTAAAACTCCTTGTAGAATTGAAAA    32637
E42           AAGAACATTCTAAACAATATATTTACTACAAAAGCCTAAAACTCCTTGTAGAATTGAAAA    32644
HEINZ         AAGAACATTCTAAACAATATATTTACTACAAAAGCCTAAAACTCCTTGTAGAATTGAAAA    32603
              ************************************************************

LA2093        ATGTCATCATACATGATCCACAATCAGCTTTAGAGGTTGGTTGTTCATAAACCTCAAAGT    32697
E42           ATGTCATCATACATGATCCACAATTAGCTTTAGAGGTTGGTTGTTCATAAACCTCAAAGT    32704
HEINZ         ATGTCATCATACATGATCCACAATCAGCTTTAGAGGTTGGTTGTTCATAAACCTCAAAGT    32663
              ************************ ***********************************

LA2093        TTTTTACTGGTCTAAGCCATGGTTACAGTGGCCCTTCAAAACTGGGAAGAAGTCGCGATT    32757
E42           TTTTTACTGGTCTAAGCCATGGTTACAGTGGCCCTTCAAAACTGGGAAGAAGTCGCGATT    32764
HEINZ         TTTTTACTGGTCTAAGCCATGGTTACAGTGGCCCTTCAAAACTGGGAAGAAGTCGCGATT    32723
```

```
            ************************************************************

LA2093      CAGGTAAAGAGCAGAAGTAGGTTGAGAATCATACTAGTCATCATATGATGGACAGTTTGG     32817
E42         CAGGTAAAGAGCAGAAGTAGGTTGAGAATCATACTAGTCATCATATGATGGACAGTTTGG     32824
HEINZ       CAGGTAAAGAGCAGAAGTAGGTTGAGAATCATACTAGTCATCATATGATGGACAGTTTGG     32783
            ************************************************************

LA2093      GAAGAGAGGAATGATAGGTGTTTTGAGAACGTAGAGAATAGTATAGAACAAATCAAGTTC     32877
E42         GAAGAGAGGAATGATAGGTGTTTTGAGAACGTAGAGAATAGTATAGAACAAATCAAGTTG     32884
HEINZ       GAAGAGAGGAATGATAGGTGTTTTGAGAACGTAGAGAATAGTATAGAACAAATCAAGTTC     32843
            ***********************************************************

LA2093      ATTGTATCTAGATTTTGTGTTTTTTGGTGTAACCAAATTTGGTCTAGTGATCCTGTATTT     32937
E42         ATTGTATCTAGATTTTGTGTTTTTTGGTGTAACCAAATTTGGTCTAGTGATCCTGTATTT     32944
HEINZ       ATTGTATCTAGATTTTGTGTTTTTTGGTGTAACCAAATTTGGTCTAGTGATCCTGTATTT     32903
            ************************************************************

LA2093      TTTATTCGATTCATTATAGACATAGGATCTGGATTTACTTGTGAATATGGCTATAGTAGT     32997
E42         TTTATTCGATTCATTATAGACATAGGATCTGGATTTACTTGTGAATATGGCTATAGTAGT     33004
HEINZ       TTTATTCGATTCATTATAGACATAGGATCTGGATTTACTTGTGAATATGGCTATAGTAGT     32963
            ************************************************************

LA2093      TAAATATAGTAGTTTAATGTTATTTTTAGGACGACAAAAATGCCCAACAACAGGAGGTGA     33057
E42         TAAATATAGTAGTTTAATGTTATTTTTAGGACGACAAAAATGCCCAACAACAGGAGGTGA     33064
HEINZ       TAAATATAGTAGTTTAATGTTATTTTTAGGACGACAAAAATGCCCAACAACAGGAGGTGA     33023
            ************************************************************

LA2093      ATACAATGGTTCCATTGTACAAGAATAAGGGTGATATCCTAATCTATAACAATTACACGG     33117
E42         ATACAATGGTTCCATTGTACAAGAATAAGGGTGATATCCTAATCTATAACAATTACACGG     33124
HEINZ       ATACAATGGTTCCATTGTACAAGAATAAGGGTGATATCCTAATCTATAACAATTACACGG     33083
            ************************************************************

LA2093      GTATCAAATTGCTATTTCACATTATGAAGGTTTGGGAGAGAGCGGTGGATATGAAGGTGA     33177
E42         GTATCAAATTGCTATTTCACATTATGAAGGTTTGGGAGAGAGCGGTGGATATGAAGGTGA     33184
HEINZ       GTATCAAATTGCTATTTCACATTATGAAGGTTTGGGAGAGAGCGGTGGATATGAAGGTGA     33143
            ************************************************************

LA2093      GGAAAGTGGAGTCCATTTCTAAGAATCAATTTGGATTCATGCCAAGACGGTCAACTACTA     33237
E42         GGAAAGTGGAGTCCATTTCTAAGAATCAATTTGGATTCATGCCAAGACGGTCAACTACTA     33244
HEINZ       GGAAAGTGGAGTCCATTTCTAAGAATCAATTTGGATTCATGCCAAGACGGTCAACTACTA     33203
            ************************************************************

LA2093      ACGCTATCCATCTTGTGCGGAGAGGGGTGCAGAAATATAGGTAAAGAAAGAGGAACCTAC     33297
E42         ACGCTATCCATCTTGTGCGGAGAGGGGTGCAGAAATATAGGTAAAGAAAGAGGAACCTAC     33304
HEINZ       ACGCTATCCATCTTGTGCGGAGAGGGGTGCAGAAATATAAGTAAAGAAAGAGGAACCTAC     33263
            **************************************** *******************

LA2093      ATATGGACTGACTTTGAAAAAGCATATGACAAATTCCCGAGGGATGTCCCCGAAGGTGCT     33357
E42         ATATGGACTGACTTTGAAAAAGCATATGACAAATTCCCGAGGGATGTCCCCGAAGGTGCT     33364
HEINZ       ATATGGACTGACTTTGAAAAAGCATATGACAAATTCCCGAGGGATGTCCCCGAAGGTGCT     33323
            ************************************************************

LA2093      TGGAGACTAGAGGTGCTCCTATGACTCATATTAGGATGATGAAGGACATGTAGATGGATC     33417
E42         TGGAGACTAGAGGTGCTCCTATGACTCATATTAGGATGATGAAGGACATGTAGATGGATC     33424
HEINZ       TGGAGACTAGAGGTGCTCCTATGACTCATATTAGGATGATGAAGGACATGTAGATGGATC     33383
            ************************************************************

LA2093      CACGATACAGGTTAGAATGGTGGCAGGAGACTTGGAGCACTTCCTAGTTGGGATGGGGTT     33477
E42         CACGATACAGGTTAGAATGGTGGCAGGAGACTCGGAGCACTTCCTAGTTGGGATGGGGTT     33484
HEINZ       CACGATACAGGTTAGAATGGTGGCAGGAGACTCGGAGCACTTCCTAGTTGGGATGGGGTT     33443
            ******************************** ***************************

LA2093      GCACTAGGGATCAGTTCTTAGCCCTTTCCTATTTGCCTTGGTGTTGGATGAGTTGATGCA     33537
E42         GCACTAGGGATCAGTTCTTAGCCCTTTCCTATTTGCCTTGGTGTTGGATGAGTTGATGCA     33544
HEINZ       GCACTAGGGATCAGTTCTTAGCCCTTTCCTATTTGCCTTGGTGTTGGATGAGTTGATGCA     33503
            ************************************************************

LA2093      GTCGATTCATAAGGAAATTCCATGTGTACGTTATTCTCGGACGACATAATGATGATTGAT     33597
E42         GTCGATTCATAAGGAAATTCCATGTGTACGTTATTCTCGGACGACATAATGATGATTGAT     33604
HEINZ       GTCGATTCATAAGGAAATTCCATGTGTACGTTATTCTCGGACGACATAATGATGATTGAT     33563
            ************************************************************
```

```
LA2093      GAGACACGGGATAAATTTCATGTTAGGTTGGAGGTTCGGATACAAACTACAAAGTCCAAA     33657
E42         GAGACACGGGATAAATTTCATGTTAGGTTGGAGGTTCGGATACAAACTACAAAGTCCAAA     33664
HEINZ       GAGACACGGGATAAATTTCATGTTAGGTTGGAGGTTCGGATACAAACTACAAAGTCCAAA     33623
            ************************************************************

LA2093      GGGTTCAGGTTGAGCAAGACCAAAACGGATATTTAGGGTGCAAATTCAGTGTTGCATTGG     33717
E42         GGGTTCAGGTTGAGCAAGACCAAAACGGATATTTAGGGTGCAAATTCAGTGTTGCATTGG     33724
HEINZ       GGGTTCAGGTTGAGCAAGACCAAAACGGATATTTAGGGTGCAAATTCAGTGTTTCATTGG     33683
            ***************************************************** ******

LA2093      ATGAAGCAAACGTAGAAGTGACGCTCGCCACATAGACTATTCGTAAGAGCTTGAGTCTAT     33777
E42         ATGAAGCAAACGTAGAAGTGACGCTCGCCACATAGACTATTCGTAAGAGCTTGAGTCTAT     33784
HEINZ       ATGAAGCAAACGTAGAAGTGACGCTCGCCACATAGACTATTCGTAAGAGCTTGAGTCTAT     33743
            ************************************************************

LA2093      AATCCAAAGAAGTAGGGACATTGACAATGATGTCACACATCTTATTGGGTAGCATGAAAT     33837
E42         AATCCAAAGAAGTAGGGACATTGACAATGATGTCACACATCTTATTGGGTAGCATGAATT     33844
HEINZ       AATCCAAAGAAGTAGGGACATTGACAATGATGTCACACATCTTATTGGGTAGCATGAAAT     33803
            ********************************************************** *

LA2093      TAATGGAGACTTGCCTCGAAAGTCTGATAATAAAGTACCATCGACACATAATTAAGAACT     33897
E42         TAATGGAGACTTGCCTCGAAAGTCTGATAATAAAGTACCATCGACACATAATTAAGAACT     33904
HEINZ       TAATGGAGACTTGCCTCGAAAGTCTGATAATAAAGTACCATCGACACATAATTAAGAACT     33863
            ************************************************************

LA2093      CCATCCTGCGAGATGTAAAGATGGTTGAAATACTAAGAAGTATTTCCTTCTTTCTCAAAT     33957
E42         CCATCCTGCGAGATGTAAAGATGGTTGAAATACTAAGAAGTATTTCCTTCTTTCTCAAAT     33964
HEINZ       CCATCCTGCGAGATGTAAAGATGGTTGAAATACTAAGAAGTATTTCCTTCTTTCTCAAAT     33923
            ************************************************************

LA2093      TTTACATGGTATCAAAGCAATTGCTACCTTTTAAAGTGAGTTTCCCTCCCTAGAAGAATT     34017
E42         TTTACATGGTATCAAAGCAATTGCTACCTTTTAAAGTGAGTTTCCCTCCCTAGAAGAATT     34024
HEINZ       TTTACATGGTATCAAAGCAATTGCTACCTTTTAAAGTGAGTTTCCCTCCCTAGAAGAATT     33983
            ************************************************************

LA2093      AGAGCTCTTTTTTTCTTAAAAAAAGGTCGAGTTTTCTCTCCTTTTTCCAATGCTTTTTCC     34077
E42         AGAGCTCTTTTTTTCTTAAAAAAAGGTCGAGTTTTCTCTCCTTTTTCCAATGCTTTTTCC     34084
HEINZ       AGAGCTCTTTTTTTCTTAAAAAAAGGTCGAGTTTTCTCTCCTTTTTCCAATGCTTTTTCC     34043
            ************************************************************

LA2093      AATCTTTACTTCTTTTGTCCAATCGATCATAACCACTATCTACATTCCATTTGATTGTGC     34137
E42         AATCTTTACTTCTTTTGTCCAATCGATCATAACCACTATCTACATTCCATTTGATTGTGC     34144
HEINZ       AATCTTTACTTCTTTTGTCCAATCGATCATAACCACTATCTACATTCCATTTGATTGTGC     34103
            ************************************************************

LA2093      ACCACAAATACGAGCTAATAAAGAGACCAACAATTACTTAGAATGATATCGGGATTCTTG     34197
E42         ACCACAAATACGAGCTAATAAAGAGACCAACAATTACTTAGAATGATATCACGATTCTTG     34204
HEINZ       ACCACAAATACGAGCTAATAAAGAGACCAACAATTACTTAGAATGATATCGCGATTCTTG     34163
            **************************************************  ********

LA2093      GTGGAAAAAAATGATTTGTTGAGGCAGAAAAATAGATATCAAATTTCTACCTAGATATCTG     34257
E42         GTGGAAAAAAATGATTTGTTGAGGCAGAAAAATAGATATCAAATTTCTACCTAGATATCTG     34264
HEINZ       GTGGAAAAAAATGATTTGTTGAGGCAGAAAAATAGATATCAAATTTCTACCTAGATATCTG     34223
            ************************************************************

LA2093      AAAGTTCTAACTTGATGACTCCATCATCTATGATTCAGTTGCAAAAACTTTTGTATACGT     34317
E42         AAAGTTCTAACTTGATGACTCCATCATCTATGATTCAGTTGCAAAAACTTTTGTATACGT     34324
HEINZ       AAAGTTCTAACTTGATGACTCCATCATCTATGATTCAGTTGCAAAAACTTTTGTATACGT     34283
            ************************************************************

LA2093      ATCTAATATCTTAACCAAATTCTTTAGGGTTAAAGAATTTCTTTCTAGTTGCTCTAGAAC     34377
E42         ATCTAATATCTTAACCAAATTCTTTAGGGTTAAAGAATTTCTTTCTAGTTGCTCTAGAAC     34384
HEINZ       ATCTAATATCTTAACCAAATTCTTTAGGGTTAAAGAATTTCTTTCTAGTTGCTCTAGAAC     34343
            ************************************************************

LA2093      AATTAGGAGATTTTGTAGAAGAAATATGGGGTTTTGCTTTTTGGCGGTAACATGTGTGGT     34437
E42         AATTAGGAGATTTTGTAGAAGAAATATGGGGTTTTGCTTTTTGGCGGTAACATGTGTGGT     34444
HEINZ       AATTAGGAGATTTTGTAGAAGAAATATGGGGTTTTGCTTTTTGGCGGTAACATGTGTGGT     34403
            ************************************************************

LA2093      CCGCTCAGGGGTCAAATCAATACGTTCAAGAAGAAAAATGGAATATCAATCTGATCAAGA     34497
E42         TCGCTCAGGGGTCAAATCAATACGTTCAAGAAGAAAATTGGAATATCAATATGATCAAGA     34504
```

```
HEINZ         CCGCTCAGGGGTCAAATCAATACGTTCAAGAAGAAAAATGGAATATCAATCTGATCAAGA     34463
               ************************************ ************ *********

LA2093        TCTTTTTTCCTGATTTGCGGTCCTATATAATATAGCTTAATCAATACTATAGTAAAAAAG     34557
E42           TCTTTTTTCCTGATTTGCGGTCCTATATAATATAGCTTAATCAATACTATAGTAAAAAAG     34564
HEINZ         TCTTTTTTCCTGATTTGCGGTCCTATATAATATAGCTTAATCAATACTATAGTAAAAAAG     34523
              ************************************************************

LA2093        GATGCAATTGGATTTGAGTTGACATAGCTTCCTGGATTTAAAAAATGTCAAGGAAAGGTG     34617
E42           GATGCAATTGGATTTGAGTTGACATAGCTTCCTGGATTTAAAAAATGTCAAGGAAAGGTG     34624
HEINZ         GATGCAATTGGATTTGAGTTGACATAGCTTCCTGGATTTAAAAAATGTCAAGGAAAGGTG     34583
              ************************************************************

LA2093        TGCAAACCGAAGAAAGCCTTGTACAAAAAGAAGCTATCCCTTAGAGCTTGGTTTGATAGA     34677
E42           TGCAAACCGAAGAAAGCCTTGTACAAAAAGAAGCTATCCCTTAGAGCTTGGTTTGATAGA     34684
HEINZ         TGCAAACCGAAGAAAGCCTTGTACAAAAAGAAGCTATCCCTTAGAGCTTGGTTTGATAGA     34643
              ************************************************************

LA2093        TCCTCCAATGTGTTGCTCTCTTTCAGTTACAACAAAGTAATGTTGATCACACCCTCTTCA     34737
E42           TCCTCCAATGTGTTGCTCTCTTTCAGTTACAACAAAGTAATGTTGATCACACCCTCTTCA     34744
HEINZ         TCCTCCAATGTGTTGCTCTCTTTCAGTTACAACAAAGTAATGTTGATCACACCCTCTTCA     34703
              ************************************************************

LA2093        TAAGACATCAATAGGGTAAACACTCTTCTCATTATTTATGTTGATGACATAATAATGTCA     34797
E42           TAAGACATCAATAGGGTAAACACTCTTCTCATTATTTATGTTGATGACATAATAATGTCA     34804
HEINZ         TAAGACATCAATAGGGTAAACACTCTTCTCATTATTTATGTTGATGACATAATAATGTCA     34763
              ************************************************************

LA2093        GGAGATGACCCGATTGAAGTTGTCAACACAAAAAAAAAAAGTCAACGATCGAGGGAAGTT     34857
E42           GGAGATGACCCGATTGAAGTTGTCAACAC-AAAAAAAAAAGTCAACGATCGAGGGAAGTT     34863
HEINZ         GGAGATGACCCGATTGAAGTTGTCAACAC-AAAAAAAAAAGTCAACGATCGAGGGAAGTT     34822
              ***************************** ******************************

LA2093        ATTTCTTAGGGATTGAGGTTGCTGGATCAAAAAGAGGAACTTTTATTAAAAAGCTTGCAA     34917
E42           ATTTCTTAGGGATTGAGGTTGCTGGATCAAAAAGAGGAACTTTTATTAAAAGGCTTGCAA     34923
HEINZ         ATTTCTTAGGGATTGAGGTTGCTGGATCAAAAAGAGGAACTTTTATTAAAAAGCTTGCAA     34882
              *************************************************** ********

LA2093        GCTTTTACTAATTTGGACTGCACTTCATCTTAAATGATAGAAGACCTTATCCGAGTATCA     34977
E42           GCTTTTACTAATTTGGACTGCACTTCATCTTAAATGATAGAAGACCTTATCCGAGTATCA     34983
HEINZ         GCTTTTACTAATTTGGACTGCACTTCATCTTAAATGATAGAAGACCTTATCCGAGTATCA     34942
              ************************************************************

LA2093        TACACTCGGAAGAGAGAACCTAGTTACTTGAAGAAGTAAGAAGGGAAGTGTAGTTGCAAG     35037
E42           TACACTCGGAAGAGAGAACCTAGTTACTTGAAGAAGTAAGAAGGGAAGTGTAGTTGCAAG     35043
HEINZ         TACACTCGGAAGAGAGAACCTAGTTACTTGAAGAAGTAAGAAGGGAAGTGTAGTTGCAAG     35002
              ************************************************************

LA2093        ATCAAGTAAGGAAGCAAAATATAGAGCTATGGCCTAAGGTGTTAATGAGCATCTTTCGCC     35097
E42           ATCAAGTAAGGAAGCAAAATATAGAGCTATGGCCTAAGGTGTTAATGAGCATCTTTGGCC     35103
HEINZ         ATCAAGTAAGGAAGCAAAATATAGAGCTATGGCCTAAGGTGTTAATGAGCATCTTTCGCC     35062
              ******************************************************** ***

LA2093        ACAAAAGTTACATGGAAGAATTGAGCTTGTCTGAAAAAGGAAAGTCTTGTGCAGCAACAA     35157
E42           ACAAAAGTTACATGGAAGAATTGAGCTTGTCTGAAAAAGGAAAGTCTTGTACAGCAACAA     35163
HEINZ         ACAAAAGTTACATGGAAGAATTGAGCTTGTCTGAAAAAGGAAAGTCTTGTGCAGCAACAA     35122
              ************************************************** *********

LA2093        CAAAGTTGTAATCAGTATAGCTCAGAATCCTGTTCAACATGACCGAAGGCAAACAAAACA     35217
E42           CAAAGTTGTAATCAGTATAGCTCAGAATCCTGTTCAACATGACCGAAGGCAAACAAAACA     35223
HEINZ         CAAAGTTGTAATCAGTATAGCTCAGAATCCTGTTCAACATGACCGAAGGCAAACAAAACA     35182
              ************************************************************

LA2093        TATATTATTGATTGACACTTTATTTAAGAGAAAGCTACTAGTGGCTCCTTGAGTTTGCTT     35277
E42           TATATTATTGATTGACACTTTATTTAAGAGAAAGCTACTAGTGGCTCCTTGAGTTTGCTT     35283
HEINZ         TATATTATTGATTGACACTTTATTTAAGAGAAAGCTACTAGTGGCTCCTTGAGTTTGCTT     35242
              ************************************************************

LA2093        CATGTACCGTCAAACGAGCAGCTAGAAAATGTGTTTAAAAATGGCCTCAGCAAAAGGACT     35337
E42           CATGTACCGTCAAACGAGCAGCTAGAAAATGTGTTTAAAAATGGCCTCAGCAAAAGGACT     35343
HEINZ         CATGTACCGTCAAACGAGCAGCTAGAAAATGTGTTTAAAAATGGCCTCAGCAAAAGGACT     35302
              ************************************************************
```

```
LA2093      TTTCATACTTTGGTTTGCAAGTAGGGCATGTGCTACATCTTTGCTCCCACTTGACAACTT     35397
E42         TTTCATACTTTGGTTTGCAAGTAGGGCATGTGCTACATCTTTGCTCCCACTTGACAACTT     35403
HEINZ       TTTCATACTTTGGTTTGCAAGTAGGGCATGTGCTACATCTTTGCTCCCACTTGACAACTT     35362
            ************************************************************

LA2093      GAGAGGGAATGTTGATTGTGTTAACTAATTAGCATGATTTAGGTGACTGTACTTTTCCA     35457
E42         GAGAGGAAATGTTGATTGTGTTAACTAATTAGCATGATTTAGGTGACTGTACTTTTCCA     35463
HEINZ       GAGAGGGAATGTTGATTGTGTTAACTAATTAGCATGATTTAGGTGACTGTACTTTTCCA     35422
            ****** *****************************************************

LA2093      TTTTCTAGAGTAATCATCATAGGATTAGAATATTTCATATTATTTGTAATTGATTGTCTT     35517
E42         TTTTCTAGAGTAATCATCATAGGATTAGAATATTTCATATTATTTGTAATTGATTGTCTT     35523
HEINZ       TTTTCTAGAGTAATCATCATAGGATTAGAATATTTCATATTATTTGTAATTGATTGTCTT     35482
            ************************************************************

LA2093      TCTTTGATTGATCAGGATTCACTTGAATAATACCTCTTCTTATGTAATATAAAGACATAC     35577
E42         TCTTTGATTGATCAGGATTCACTTGAATAATACCTCTTCTTATGTAATATAAAGACATAC     35583
HEINZ       TCTTTGATTGATCAGGATTCACTTGAATAATACCTCTTCTTATGTAATATAAAGACATAC     35542
            ************************************************************

LA2093      TGAAATACAGAGAAGTCTTTTCTTCTTGCTCAATTCTACATAACATTCAAAAAATGAGCA     35637
E42         TGAAATACAGAGAAGTCTTTTCTTCTTGCTCAATTCTACATAACATTCAAAAAATGAGCA     35643
HEINZ       TGAAATACAGAGAAGTCTTTTCTTCTTGCTCAATTCTACATAACATTCAAAAAATGAGCA     35602
            ************************************************************

LA2093      GTTAAATACCAACTCAGTAACCTTTATCACCATCCAATATTGGGAAGAAACGGCATAAAA     35697
E42         GTTAAATACCAACTCAGTAACCTTTATCACCATCCAATATTGGGAAGAAACGGCATAAAA     35703
HEINZ       GTTAAATACCAACTCAGTAACCTTTATCACCATCCAATATTGGGAAGAAACGGCATAAAA     35662
            ************************************************************

LA2093      AGAAACTCATATTTTTTCTATACTAAGATTAGCTCATTGTGTAACTGCAGGTACTGACAG     35757
E42         AGAAACTCATATTTTTTCTGTACTAAGATTAGCTCATTGTGTAACTGCAGGTACTGACAG     35763
HEINZ       AGAAACTCATATTTTTTCTATACTAAGATTAGCTCATTGTGTAACTGCAGGTACTGACAG     35722
            ******************* ****************************************

LA2093      AATTAATCAAAGTTAGATAGATACCAGCATAAGAGTTTAGCTTGTCCAAATGAAGCGTCT     35817
E42         AATTATTCAAAGTTAGATAGATACCAGCATAAGAGTTTAGCTTGTCCAAATGAAGCGTCT     35823
HEINZ       AATTAATCAAAGTTAGATAGATACCAGCATAAGAGTTTAGCTTGTCCAAATGAAGCGTCT     35782
            ***** ******************************************************

LA2093      TGAGCAACTTGGGAATGTCAATATGTAGATGTATACCAGCAATATCCTGCACATGGAACT     35877
E42         TGAGCAACTTGGGAATGTCAATATGTAGATGTATACCAGCAATATCCTGCACATGGAACT     35883
HEINZ       TGAGCAACTTGGGAATGTCAATATGTAGATGTATACCAGCAATATCCTGCACATGGAACT     35842
            ************************************************************

LA2093      TTAATCTGAGAACCAGGTTAAAGCAGCAACAGAATGTGCTTAGCTCTTAAATTTACAAGG     35937
E42         TTAATCTGAGAACCACGTTAAAGCAGCAACAGAATGTGCTTAGCTCTTAAATTTACAAGG     35943
HEINZ       TTAATCTGAGAACCACGTTAAAGCAGCAACAGAATGTGCTTAGCTCTTAAATTTACAAGG     35902
            *************** ********************************************

LA2093      AAAAACTGAAAAGAAACGAAATATGCATTTGCTTATTGAATTTGACTGAAAAACTAACAG     35997
E42         AAAAACTGAAAAGAAACGAAATATGCATTTGCTTATTGAATTTGACTGAAAAACTAACAG     36003
HEINZ       AAAAACTGAAAAGAAACGAAATATGCATTTGCTTATTGAATTTGACTGAAAAACTAACAG     35962
            ************************************************************

LA2093      CTCGTAACCAAGTTGGATGCCACCTATATATAAGAGTGTACAGACAAGTAATGCATCATT     36057
E42         CTCGTAACCAAGTTGGATGCCACCTATATATAAGAGTGTACAGACAAGTAATGCATCATT     36063
HEINZ       CTCGTAACCAAGTTGGATGCCACCTATATATAAGAGTGTACAGACAAGTAATGCATCATT     36022
            ************************************************************

LA2093      TCTGTGGAAAAATATCAGGTATTTAATATCTAGCAACACAATGTCTCAAGCCCAACAGCC     36117
E42         TCTGTGGAAAAATATCAGGTATTTAATATCTAGCAACACAATGTCTCAAGCCCAACAGCC     36123
HEINZ       TCTGTGGAAAAATATCAGGTATTTAATATCTAGCAACACAATGTCTCAAGCCCAACAGCC     36082
            ************************************************************

LA2093      CAAGCACTACCATAAATATGATCAGCAGAGTAGACCCCAAGTTAGACGTTCTTATCAGTC     36177
E42         CAAGCACTACCATAAATATGATCAGCAGAGTAGACCCCAAGTTAGACGTTCTTATCAGTC     36183
HEINZ       CAAGCACTACCATAAATATGATCAGCAGAGTAGACCCCAAGTTAGACGTTCTTATCAGTC     36142
            ************************************************************

LA2093      AGTAACCATAGAGCTCAAACAAAACTTCCGCAAAACACAATTAGGTAATAGCTGTTCTAA     36237
```

```
E42             AGTAATCATAGAGCTCAAACAAAACTTCCGCAAAACACAATTAGGTAATAGCTGTTCTAA     36243
HEINZ           AGTAACCATAGAGCTCAAATAAAACTTCCGCAAAACACAATTAGGTAATAGCTGTTCTAA     36202
                ***** ************* ****************************************

LA2093          ACAATTTCATAAAAACAACATAGGGTAATCTTAGTAGCTCAGTTGGTTGGCTGGCTACCT     36297
E42             ACAATTTCATAAAAACAACATAGGGTAATCTTAGTAGCTCAGTTGGTTGGCTGGCTACCT     36303
HEINZ           ACAATTTCATAAAAACAACATAGGGTAATCTTAGTAGCTCAGTTGGTTGGCTGGCTACCT     36262
                ************************************************************

LA2093          GAACTCCCACCTTGTTGGTGAGGGTTCGATTCCCCACATTGTAATCCCCATCCATTACCC     36357
E42             GAACTCCCACCTTGTTGGTGAGGGTTCGATTCCCCACATTGTAATCCCCATCCATTACCC     36363
HEINZ           GAACTCCCACCTTGTTGGTGAGGGTTCGATTCCCCACATTGTAATCCCCATCCATTACCC     36322
                ************************************************************

LA2093          TTTCCCCTACCCTTAATTGTTTTGCTAAAAAAAAAACATAGGGCAAACATTGGCACTAGT     36417
E42             TTTCCCCTACCCTTAATTGTTTTGCTAAAAAAAAAACATAGGGCAAACATTGGCACTAGT     36423
HEINZ           TTTCCCCTACCCTTAATTGTTTTGCTAAAAAAAAAACATAGGGCAAACATTGGCACTAGT     36382
                ************************************************************

LA2093          CTGCAATTCAATCAATGACCTGCGATACTCCAGAACCCAAAATGTGCAGAAGAGAGCAAC     36477
E42             CTGCAATTCAATCAATGACCTGCGATACTCCAGAACCCAAAATGTGCAGAAGAGAGCAAC     36483
HEINZ           CTGCAATTCAATCAATGACCTGCGATACTCCAGAACCCAAAATGTGCAGAAGAGAGCAAC     36442
                ************************************************************

LA2093          CGAAACTGAAAGATGTAACAAAGCAATCATAATTTTACAATGTGATTTGTAATTTACATC     36537
E42             CGAAACTGAAAGATGTAACAAAGCAATCATAATTTTACAATGTGATTTGTAATTTACATC     36543
HEINZ           CGAAACTGAAAGATGTAACAAAGCAATCATAATTTTACAATGTGATTTGTAATTTACATC     36502
                ************************************************************

LA2093          CAAACATTAAAATCTTTTATTCAAATGCGTATCTCATTAAAAACTCAACTTATGTTGCAG     36597
E42             CAAACATTAAAATCTTTTATTCAAATGCATATCTCATTAAAAACTCAACTTATGTTGCAG     36603
HEINZ           CAAACATTAAAATCTTTTATTCAAATGCGTATCTCATTAAAAACTCAACTTATGTTGCAG     36562
                **************************** *******************************

LA2093          GTAAAAAAAACTGTGAATGACGATACAACATAACACCTGATCTCCCCAGTTCCTTAAGAA     36657
E42             GTAAAAAAAACTGTGACTGACGATACAACATAACACCTGATCTCCCCAGTTCCTTAAGAA     36663
HEINZ           GTAAAAAAAACTGTGAATGACGATACAACATAACACCTGATCTCCCCAGTTCCTTAAGAA     36622
                **************** *******************************************

LA2093          TAACTAACAAATACAATAAACCTTCCATGTAGAATTTTAATAAATTGGGAAAAAATGCAAT     36717
E42             TAACTAACAAATACAATAAACCTTCCATGTAGAATTTTAATAAATTGGGAAAAAATGCAAT     36723
HEINZ           TAACTAACAAATACAATAAACCTTCCATGTAGAATTTTAATAAATTGGGAAAAAATGCAAT     36682
                ************************************************************

LA2093          AGCAATCACCTTCTTCTCTACATACTGTAATCCTTCCAAAAGATAAGCACAGAAATGCAT     36777
E42             AGCAATCTCCTTCTTCTCTACATACTGTAATCCTTCCAAAAGATAAGCACAGAAATGCAT     36783
HEINZ           AGCAATCACCTTCTTCTCTACATACTGTAATCCTTCCAAAAGATAAGCACAGAAATGCAT     36742
                ******* ****************************************************

LA2093          AAAACTACAATGTTATAACACAATGAGAAACATCATTAAACATCTAGGTAGCAGAAGTAG     36837
E42             AAAACTACAATGTTATAACACAATGAGAAACATCATTAAACATCTAGGTAGCAGAAGTAG     36843
HEINZ           AAAACTACAATGTTATAACACAATGAGAAACATCATTAAACATCTAGGTAGCAGAAGTAG     36802
                ************************************************************

LA2093          GCAGGTATGTGATGTTCAGATTCATTTTCTTAATTAACTCCCTTCATAAGAGATAACTAT     36897
E42             GCAGGTATGTGATGTTCAGATTCATTTTCTTAATTAACTCCCTTCATAAGAGATAACTAT     36903
HEINZ           GCAGGTATGTGATGTTCAGATTCATTTTCTTAATTAACTCCCTTCATAAGAGATAACTAT     36862
                ************************************************************

LA2093          ACTTAGTCTACCAAGTACAAACTCAACAGTGCTTCCTCGTTAAAGGCGGGCAAAATATGT     36957
E42             ACTTAGTCTACCAAGTACAAACTCAACAGTGCTTCCTCGTTAAAGGCGGGCAAAATATGT     36963
HEINZ           ACTTAGTCTACCAAGTACAAACTCAACAGTGCTTCCTCGTTAAAGGCGGGCAAAATATGT     36922
                ************************************************************

LA2093          TTGGTGGTCGAAATAGAAGGTTCAATGGCAAAGAGTTGCATATGAAGCAATAAATACATG     37017
E42             TTGGTGGTCGAAATAGAAGGTTCAATGGCAAAGAGTTGCATATGAAGCAATAAATACATG     37023
HEINZ           TTGGTGGTCGAAATAGAAGGTTCAATGGCAAAGAGTTGCATATGAAGCAATAAATACATG     36982
                ************************************************************

LA2093          GGAAATCCACAAATAAAGCTAGTCTTCCTAAGTGTATTTCTAGTCTGTTAATTGGGAAAT     37077
E42             GGAAATCCACAAATAAAGCTAGTCTTCCTAAGTGTATTTCTAGTCTGTTAATTGGGAAAT     37083
HEINZ           GGAAATCCACAAATAAAGCTAGTCTTCCTAAGTGTATTTCTAGTCTGTTAATTGGGAAAT     37042
```

```
               ************************************************************

LA2093         ATCACAATCTAGGATTAAGACCACTCAATATGACCTAGGATCTTCATTACATCTAAAAGA     37137
E42            ATCACAATCTAGGATTAAGACCACTCAATATGACCTAGGATCTTCATTACATCTAAAAGA     37143
HEINZ          ATCACAATCTAGGATTAAGACCACTCAATATGACCTAGGATCTTCATTACATCTAAAAGA     37102
               ************************************************************

LA2093         CTAATAAAAGATGACATAATTCGATGCAATAAAGGTGTAAATACTTCATAACAGTATTCA     37197
E42            CTAATAAAAGATGACATAATTCGATGCAATAAAGGTGTAAATACTTCATAACAGTATTCA     37203
HEINZ          CTAATAAAAGATGACATAATTCGATGCAATAAAGGTGTAAATACTTCATAACAGTATTCA     37162
               ************************************************************

LA2093         CTTTGTTGTTCCTCAAATAAGGGAATCCCCAATATGCCAAAATAAAGTATCAAAGAAGAT     37257
E42            CTTTGTTGTTCCTCAAATAAGGGAATCCCCAATATGCCAAAATAAAGTATCAAAGAAGAT     37263
HEINZ          CTTTGTTGTTCCTCAAATAAGGGAATCCCCAATATGCCAAAATAAAGTATCAAAGAAGAT     37222
               ************************************************************

LA2093         GCAAAGAATTCCTCCGAGCAAGTTTACACTTATCCAAACTTACCCATAATCCTCCAAGGA     37317
E42            GCAAAGAATTCCTCCGAGCAAGTTTACACTTATCCAAACTTACCCATAATCCTCCAAGGA     37323
HEINZ          GCAAAGAATTCCTCCGAGCAAGTTTACACTTATCCAAACTTACCCATAATCCTCCAAGGA     37282
               ************************************************************

LA2093         CTCGCTTGGAAACTCTCCAAATGAAAGACAAGCGCCCATGCCATTTTTGGCCAATTCGAA     37377
E42            CTCGCTTGGAAACTCTCCAAATGAAAGACAAGCGCCCATGCCATTTTTGGCCAATTCGAA     37383
HEINZ          CTCGCTTGGAAACTCTCCAAATGAAAGACAAGCGCCCATGCCATTTTTGGCCAATTCGAA     37342
               ************************************************************

LA2093         TTATAGCTGCTTTTAGTGATTCCTTTGGCGAGATGAAAAATCGTGCACGCAAAACTTCCT     37437
E42            TTATAGCTGCTTTTAGTGATTCCTTCGGCGAGATGAAAAATCGTGCACGCAAAACATCCT     37443
HEINZ          TTATAGCTGCTTTTAGTGATTCCTTCGGCGAGATGAAAAATCGTGCACGCAAAACTTCCT     37402
               ************************* ***************************** ****

LA2093         TACTGATTTTGGGGACCACACCATCACCCACTGACTCACTGTTCTTTGAACTTCCCAGTG     37497
E42            TACTGATTTTGGGGACCACACCATCACCCACTGACTCACTGTTCTTTGAACTTCCCAGTG     37503
HEINZ          TACTGATTTTGGGGACCACACCATCACCCACTGACTCACTGTTCTTTGAACTTCCCAGTG     37462
               ************************************************************

LA2093         AAGACTCAGAGGGCTCATTGTTTGAATATAATAGAGAGTTACCACTCTTAGCTCCATCAC     37557
E42            AAGACTCAGAGGGCTCATTGTTTGAATATAATAGAGAGTTACCACTCTTAGCTCCATCAC     37563
HEINZ          AAGACTCAGAGGGCTCATTGTTTGAATATAATAGAGAGTTACCACTCTTAGCTCCATCAC     37522
               ************************************************************

LA2093         TTAACCAGTCCAACCATGGTTTTGTAGCAACAGTAACTGAACTATCTTCATTCGCATGTT     37617
E42            TTAACCAGTCCAACCATGGTTTTGTAGCAACAGTAACTGAACTATCTTCATTCGCATGTT     37623
HEINZ          TTAACCAGTCCAACCATGGTTTTGTAGCAACAGTAACTGAACTATCTTCATTCGCATGTT     37582
               ************************************************************

LA2093         TCAGGTTCTCATCAATAGGTTCTTCATTGGCAACATTTACAACATCATAGTTGGTTTGAT     37677
E42            TCAGGTTCTCATCAATAGGTTCTTCATTGGCAACATTTACAACATCATAGTTGGTTTGAT     37683
HEINZ          TCAGGTTCTCATCAATAGGTTCTTCATTGGCAACATTTACAACATCATAGTTGGTTTGAT     37642
               ************************************************************

LA2093         TTTGTGACCACAAGCCAGCACTAGTTATCTGTAGGAGAAAACAGAGTCAGCTGAATCTTT     37737
E42            TTTGTGACCACAAGCCAGCACTAGTTATCTGTAGGAGAAAACAGAGTCAGCTGAATCTTT     37743
HEINZ          TTTGTGACCACAAGCCAGCACTAGTTATCTGTAGGAGAAAACAGAGTCAGCTGAATCTTT     37702
               ************************************************************

LA2093         CAATAACTGGCACATGTGAGTATTCACAAATTTACGAAAAAACCTTTACAACTAGCAGCT     37797
E42            CAATAACTGGCACATGTGAGTGTTCACAAATTTACGAAACAACCTTTACAACTAGCAGCT     37803
HEINZ          CAATAACTGGCACATGTGAGTATTCACAAATTTACGAAAAAACCTTTACAACTAGCAGCT     37762
               ********************* ***************** ********************

LA2093         GCAATAGTGCAGTTGATGTACGAAATCGCAGAATAATAGATTCATGAAAATGCTAAACGT     37857
E42            GCAATAGTGCAGTTGATGTACGAAATCGCAGAATAATAGATTCATGAAAATGCTAAACGT     37863
HEINZ          GCAATAGTGCAGTTGATATACGAAATCGCAGAATAATAGATTCATGAAAATGCTAAACGT     37822
               ***************** ******************************************

LA2093         TCTGCGAGAACTCATCCTTCAATATTAGTCAACTCAGTTTATCATCGATATTGACCTTGA     37917
E42            TCTGCGAGAACTCATCCTTCAATATTAGTCAACTCAGTTTATCATCGATATTGACCTTGA     37923
HEINZ          TCTGCGAGAACTCATCCTTCAATATTAGTCAACTCAGTTTATCATCGATATTGACCTTGA     37882
               ************************************************************
```

```
LA2093      ACCAGGTCATTTTTCACTGTTCATATTTCTATCAATTTTTTAGAACAATTTGTCAAACT    37977
E42         ACCAGGTCATTTTTCACTGTTCATATTTCTATCAATTTTTTAGAACAATTTGTCAAACT    37983
HEINZ       ACCAGGTCATTTTTCACTGTTCATATTTCTATCAATTTTTTAGAACAATTTGTCAAACT    37942
            ************************************************************

LA2093      TCTTTGTTTGAGAAGGTAAAATATATTATATAATATCAGTTGCACAATGGATGCGCACAG    38037
E42         TCTTTGTTTGAGAAGGTAAAATATATTATATAATATCAGTTGCACAATGGATGCGCACAG    38043
HEINZ       TCTTTGTTTGAGAAGGTAAAATATATTATATAATATCAGTTGCACAATGGATGCGCACAG    38002
            ************************************************************

LA2093      CATATTTACATTCCAAAAGAAAGCAAAATGATCTCCTTGCTCTTCTTACAGTTTGTTGAA    38097
E42         CATATTTACATTCCAAAAGAAAGCAAAATGATCTCCTTGCTCTTCTTACAGTTTGTTGAA    38103
HEINZ       CATATTTACATTCCAAAAGAAAGCAAAATGATCTCCTTGCTCTTCTTACAGTTTGTTGAA    38062
            ************************************************************

LA2093      CTTTATTAGCATACAATTTCTTTTGCCAAGAAATAATGTGAATTAGTTCCAATTAGATAA    38157
E42         CTTTATTAGCATACAATTTCTTTTGCCAAGAAATAATGTGAATTAGTTCCAATTAGAAAA    38163
HEINZ       CTTTATTAGCATACAATTTCTTTTGCCAAGAAATAATGTGAATTAGTTCCAATTAGATAA    38122
            ********************************************************* **

LA2093      AAATGCAAAATAAACAAACAACCAAAAAACATAGCTTCTTAAGATGTCATGTGAATCATT    38217
E42         AAATGCAAAATAAACAAACAACCAAAAAACATAGCTTCTTAAGATGTTATGTGAATCATT    38223
HEINZ       AAATGCAAAATAAACAAACAACCAAAAAACATAGCTTCTTAAGATGTCATGTGAATCATT    38182
            *********************************************** ************

LA2093      AACTGTACAAGATCTAACCTCTTTTATATATTTCTTCGGATAACAGTAGCATCTTGGCCA    38277
E42         AACTGTACAAGATCTAACCTCTTTTATATATTTCTTCGGATAACAGTAGCATCTTGGCCA    38283
HEINZ       AACTGTACAAGATCTAACCTCTTTTATATATTTCTTCGGATAACAGTAGCATCTTGGCCA    38242
            ************************************************************

LA2093      ACAAGCACACCTCAACTAATCTACCAATACTTGTTACCCACCACCAACACATGTACTAGA    38337
E42         ACAAGCACACCTCAACTAATCTACCAATACTTGTTACCCACCACCAACACATGTACTAGA    38343
HEINZ       ACAAGCACACCTCAACTAATCTACCAATACTTGTTACCCACCACCAACACATGTACTAGA    38302
            ************************************************************

LA2093      TAACGCCTTTATTTATGATTCTGGTCTGGGTCAGCTTTTGTGTATCCCGGGTAACTCTGT    38397
E42         TAACGCCTTTTTTTATGATTCTAGTCTGGGTCAGCTTTTGTGTATCCCGGGTAACTCTGT    38403
HEINZ       TAACGCCTTTATTTATGATTCTGGTCTGGGTCAGCTTTTGTGTATCCCGGGTAACTCTGT    38362
            ********** *********** *************************************

LA2093      CTAAAATGTGGCACATATACATAC--ATACATATATATATATATATATATGACATATACC    38455
E42         CTAAAATGTGGCACATATACATACATATATATATATATATATATATATATGACATATACC    38463
HEINZ       CTAAAATGTGGCACATATACATACATACATATATATATATATATATATATGACATATACC    38422
            ************************  * * ******************************

LA2093      CAAAAATCAAACCACATACCTAATCAATACTTCACGGTCTAAAATGTGGTCAAAGTACTC    38515
E42         CAAAAATCAAACCACATACCTAATCAATACTTCACGGTCTAAAATGTGGTCAAAGTACTC    38523
HEINZ       CAAAAATCAAACCACATACCTAATCAATACTTCACGGTCTAAAATGTGGTCAAAGTACTC    38482
            ************************************************************

LA2093      AAGATTAGTTGACCAACTCAGGTATGGATCCGATACTCATGTCATTTCAACGCAGGTGGG    38575
E42         AAGATTAGTTGACCAAATCAGGTATGGATCCGATACTCATGTCATTTCAACGCAGGTGGG    38583
HEINZ       AAGATTAGTTGACCAACTCAGGTATGGATCCGATACTCATGTCATTTCAACGCAGGTGGG    38542
            **************** *******************************************

LA2093      ATGGAGATTTTGAAGAGTTAGCACAACATAGAAAGGACAACATAAGTCATACATTTAATA    38635
E42         ATGGAGATTTTGAAGAGTTAGCACAACATAGAAAGGACAACATAAGTCATACATTTAATA    38643
HEINZ       ATGGAGATTTTGAAGAGTTAGCATAACATAGAAAGGACAACATAAGTCATACATTTAATA    38602
            *********************** ************************************

LA2093      AGTTCTCACCATAACATGAAGAGGATTATTTGTGTTTAGCCCATTGTGATCTATTAATTT    38695
E42         AGTTCTCACCATAACATGAAGAGGATTATTTGTGTTTAGCCCATTGTGATCTATTAATTT    38703
HEINZ       AGTTCTCACCATAACATGAAGAGGATTATTTGTGTTTAGCCCATTGTGATCTATTAATTT    38662
            ************************************************************

LA2093      ACATCACATTTCTAGATGTAGATACTTCCAATATGATCCAACATGTGGCTTACTTTGCTT    38755
E42         ACATCACATTTCTAGATGTAGATACTTCCAATATGATCCAACATGTGGCTTACTTTGCTT    38763
HEINZ       ACATCACATTTCTAGATGTAGATACTTCCAATATGATCCAACATGTGGCTTACTTTGCTT    38722
            ************************************************************

LA2093      GCTTCCAGGCTAAAAACTTTTCAACTAAAGTTGTTACTTCCCAAACACAAAATCTAAACA    38815
E42         GCTTCCAGGCTAAAAACTTTTCAACTAAAGTTGTTACTTCCCAAACACAAAATCTAAACA    38823
```

```
HEINZ          GCTTCCAGGCTAAAAACTTTTCAACTAAAGTTGTTACTTCCCAAACACAAAATCTAAACA     38782
               ************************************************************

LA2093         CACAAAACAGTGAAGACTTTTTTTCTGCTTTAAATTTTCTTTCAACAACCCAAAACCTAA     38875
E42            CACAAAACAGTGAAGACTTCTTTTCTGCTTTAAATTTTCTTTCAACAACCCAAAACCTAA     38883
HEINZ          CACAAAACAGTGAAGACTTTTTTTCTGCTTTAAATTTTCTTTCAACAACCCAAAACCTAA     38842
               ******************* ****************************************

LA2093         ATGCATACAAAATTATTATTGAGAAAATAAAAAAGGTACAAGTTTTTCAGTATCAGCGGA     38935
E42            ATGCATACAAAATTATTATTGAGAAAATAAAAAAGGTACAAGTTTTTCAGTATCAGCGGA     38943
HEINZ          ATGCATACAAAATTATTATTGAGAAAATAAAAAAGGTACAAGTTTTTCAGTATCAGCGGA     38902
               ************************************************************

LA2093         TGAGATCACTCACCTTGATTTGAATGAGTTGTGCATCTGAGATGTTCACTCTAGACAATT     38995
E42            TGAGATCACTCACCTTGATTTGAATGAGTTGTGCATCTGAGATGTTCACTCTAGACAATT     39003
HEINZ          TGAGATCACTCACCTTGATTTGAATGAGTTGTGCATCTGAGATGTTCACTCTAGACAATT     38962
               ************************************************************

LA2093         CACTTGAACAGCCAGGCTACACAGAACAAATTATAGTCATTACTTAAAAAATTTGCATGT     39055
E42            CACTTGAACAGCCAGGCTACACAGAACAAATTATAGTCATTACTTAAAAAATTTGCATGT     39063
HEINZ          CACTTGAACAGCCAGGCTACACACAACAAATTATAGTCATTACTTAAAAAATTTGCATGT     39022
               *********************** ************************************

LA2093         AAGGAAATGAAACACCCACCCCCAAACAACCCCACCATATCCAGAAAGAAGAGAAATCTA     39115
E42            AAGGAAATGAAACACCCACCCCCAAACAACCCCACCATATCCAGAAAGAAGAGAAATCTA     39123
HEINZ          AAGGAAATGAAACACCCACCCCCAAACAACCCCACCATATCCAGAAAGAAGAGAAATCTA     39082
               ************************************************************

LA2093         CCTAAAGAAGCATTAAGAGAGTTGCAAAATTGATAGCTGAAGAACAACATTTTTATTCTA     39175
E42            CCTAAAGAAGCATTAAGAGAGTTGCAAAATTGATAGCTGAAGAACAACATTTTTATTCTA     39183
HEINZ          CCTAAAGAAGCATTAAGAGAGTTGCAAAATTGATAGCTGAAGAACAACATTTTTATTCTA     39142
               ************************************************************

LA2093         AAAAATTAAAAGGTAAATGATTTTATTAATTATGAAACAATCTGGCATAGAGAAATAGAA     39235
E42            AAAAATTAAAAGGTAAATGATTTTATTAATTATGAAACAATCTGGCATAGAGAAATAGAA     39243
HEINZ          AAAAATTAAAAGGTAAATGATTTTATTAATTATGAAACAATCTGGCATAGAGAAATAGAA     39202
               ************************************************************

LA2093         GTAAGATTCTAAACAATCAATACCCAATCTTGTTAAAAAAAACAAGGGTAGTCCTGAAAT     39295
E42            GTAAGATTCTAAACAATCAATACCCAATCTTGTTAAAAAAAACAAGGGTAGTCCTGAAAT     39303
HEINZ          GTAAGATTCTAAACAATCAATACCCAATCTTGTTAAAAAAAACAAGGGTAGTCCTGAAAT     39262
               ************************************************************

LA2093         GTGCATAATTATTCTCCATTTCTTTGAAAGCTCTAATATTTAGTGCTTGTGGGTAATCAA     39355
E42            GTGCATAATTATTCTCCATTTCTTTGAAAGCTCTAATATTTAGTGCTTGTGGGTAATCAA     39363
HEINZ          GTGCATAATTATTCTCCATTTCTTTGAAAGCTCTAATATTTAGTGCTTGTGGGTAATCAA     39322
               ************************************************************

LA2093         GAAGACCTAACCGTAGATTGTTTCACTTAGTGTCAGTAACTGAATATGTATAGGAGGGAG     39415
E42            GAAGACCTAACCGTAGATTGTTTCACTTAGTGTCAGTAACTGAATATGTATAGGAGGGAG     39423
HEINZ          GAAGACCTAACCGTAGATTGTTTCACTTAGTGTCAGTAACTGAATATGTATAGGAGGGAG     39382
               ************************************************************

LA2093         CAGGTCAGGCATAAACATAGGTAATCCTATTTTTTCCCTGAAAGAAACAATTATTAAAAA     39475
E42            CAGGTCAGGCATAAACATAGGTAATCCTATTTTTTCCCTGAAAGAAACAATTATTAAAAA     39483
HEINZ          CAGGTCAGGCATAAACATAGGTAATCCTATTTTTTCCCTGAAAGAAACAATTATTAAAAA     39442
               ************************************************************

LA2093         CTTAAGGAAAATTGTAATAATTTGATAAACCTGTTGAACGTAATTGGCGTGCATAACAAC     39535
E42            CTTAAGGAAAATTGTAATAATTTGATAAACCTGTTGAACGTAATTGGCGTGCATAACAAC     39543
HEINZ          CTTAAGGAAAATTGTAATAATTTGATAAACCTGTTGAACGTAATTGGCGTGCATAACAAC     39502
               ************************************************************

LA2093         GAGTATACAGAAGAGGGTTACGGCCATGAAGAGATACAAATACTCGAGAGTAGCTCTGAT     39595
E42            GAGTATACAGAAGAGGGTTACGGCCATGAAGAGATACAAATACTCGAGAGTAGCTCTGAT     39603
HEINZ          GAGTATACAGAAGAGGGTTACGGCCATGAAGAGATACAAATACTCGAGAGTAGCTCTGAT     39562
               ************************************************************

LA2093         CCTGGGCGTCAGCATCTGAGCAAATCGTTCTTGAACCCGTATAAACGTCTGCTCTGGATC     39655
E42            CCTGGGCGTCAGCATCTGAGCAAATCGTTCTTGAACCCGTATAAACGTCTGCTCTGGATC     39663
HEINZ          CCTGGGCGTCAGCATCTGAGCAAATCGTTCTTGAACCCGTATAAACGTCTGCTCTGGATC     39622
               ************************************************************
```

```
LA2093      CATCTAAACATGCATACAACTATGATTTGGGATTTTCAGTCTTCTCGTCAGCTCTTCACA     39715
E42         CATCTAAACATGCATACAACTATGATTTGGGATTTTCAGTCTTCTCGTCAGCTCTTCACA     39723
HEINZ       CATCTAAACATGCATACAACTATGATTTGGGATTTTCAGTCTTCTCGTCAGCTCTTCACA     39682
            ************************************************************

LA2093      ATCAACCTTGTCATATCAATTGGCGAATTAAATTTTATGGGAACGAATACTCTTATTTGG     39775
E42         ATCAACCTTGTCATATCAATTGGCGAATTAAATTTTATGGGAACGAAAACTCTTATTTGG     39783
HEINZ       ATCAACCTTGTCATATCAATTGGCGAATTAAATTTTATGGGAACGAATACTCTTATTTGG     39742
            *********************************************** ************

LA2093      AAACCATCGTATAAGTTAGAAGTTGTGATGCGATGCTCATAGATAGGGTA       39825
E42         AAACCATCGTATAAGTTAGAAGTTGTGATGCGATGCTCATAGATAGGGTA       39833
HEINZ       AAACCATCGTATAAGTTAGAAGTTGTGATGCGATGCTCATAGATAGGGTA       39792
            **************************************************
```

Solyc04g076270

```
HEINZ       TAGTTATACTTCCTAGGCCTACCTCTTTTCTTAGGTCCTGTCGACATTGGTCTGGGAGCA   60
E42         TAGTTATACTTCCTAGGCCTACCTCTTTTCTTAGGTCCTGTCGACATTGGTCTGGGAGCA   60
LA2093      TAGTTATACTTCCTAGGCCTACCTCTTTTCTTAGGTCCTGTCGACATTGGTCTGGGAGCA   60
            ************************************************************

HEINZ       GCAGCAGCAGCAGCAGGAGGAGGTGGAGGAGCCTGAGGCCGCGCTGGCGCTGGATTCGTT   120
E42         GCAGC------------AGGAGGTGGAGGAGCCTGAGGCCGCGCTGGCGCTGGATTCGTT   108
LA2093      GCAGCAGC---AGCAGGAGGAGGTGGAGGAGCCTGAGGCCGCGCTGGCGCTGGATTCGTT   117
            *****            *******************************************

HEINZ       ACCTGAACCGTAGCTGGAATTGGAGTAGCATTTTGTTGATTATTCGCAGCATTTTGAGTC   180
E42         ACCTGAACCGTAGCTGGAATTGGAGTAGCATTTTGTTGATTATTCGCAGCATTTTGAGTC   168
LA2093      ACCTGAACCGTAGCTGGAATTGGAGTAGCATTTTGTTGATTATTCGCAGCATTTTGAGTC   177
            ************************************************************

HEINZ       GAAAACATCGGTGGCATCCAAGTTGGAAATGCACCAGTAGTTGCAGCAGCAGCAGCAGCT   240
E42         GAAAACATCGGTGGCATCCAAGTTGGAAATGCACCAGTAGTTGCAGCAGCAGCAGCAGCT   228
LA2093      GAAAACATCGGTGGCATCCAAGTTGGAAATGCACCAGTAGTTGCAGCAGCAGCAGCAGCT   237
            ************************************************************

HEINZ       CCTTTCACAGTACCCAAATCCTCCAAATTCCCATTCACAAAACCCATTGGAAAAAAACCCC   300
E42         CCTTTCACAGTACCCAAATCCTCCAAATTCCCATTCACAAAACCCATTGGAAAAAAACCCC   288
LA2093      CCTTTCACAGTACCCAAATCCTCCAAATTCCCATTCACAAAACCCATTGGAAAAAAACCCC   297
            ************************************************************

HEINZ       CAAATGCAATAATAAGCTTCTTTCCCTGGCACCATTGGTGGCATTGACAAAATCTCAGTA   360
E42         CAAATGCAATAATAAGCTTCTTTCCCTGGCACCATTGGTGGCATTGACAAAATCTCAGTA   348
LA2093      CAAATGCAATAATAAGCTTCTTTCCCTGGCACCATTGGTGGCATTGACAAAATCTCAGTA   357
            ************************************************************

HEINZ       GCAGTAAAAGCCCTTTTACATTTATCACATCGTAAACAACACCCTTCATAAACCCTAGGA   420
E42         GCAGTAAAAGCCCTTTTACATTTATCACATCGTAAACAACACCCTTCATAAACCCTAGGA   408
LA2093      GCAGTAAAAGCCCTTTTACATTTATCACATCGTAAACAACACCCTTCATAAACCCTAGGA   417
            ************************************************************

HEINZ       TACTCATACAAATTGTAACAATACGGACACGCCGTCCACAAATTCACCACCCTTTGCCCT   480
E42         TACTCATACAAATTGTAACAATACGGACACGCCGTCCACAAATTCACCACCCTTTGCCCT   468
LA2093      TACTCATACAAATTGTAACAATACGGACACGCCGTCCACAAATTCACCACCCTTTGCCCT   477
            ************************************************************

HEINZ       CTCATCGCACCACTCATCGGCGCCGGCGCCGTAGCCTTCGGCGGCGCACTATGATAACTC   540
E42         CTCATCGCACCACTCATCGGCGCCGGCGCCGTAGCCTTCGGCGGCGCACTATGATAACTC   528
LA2093      CTCATCGCACCACTCATCGGCGCCGGCGCCGTAGCCTTCGGCGGCGCACTATGATAACTC   537
            ************************************************************

HEINZ       TTCTGACTCACCGGACTGGGATTAGGGTTCTGATTTTGACCCGAACCCGAATTACTCGCT   600
E42         TTCTGACTCACCGGACTGGGATTAGGGTTCTGATTTTGACCCGAACCCGAATTACTCGCT   588
LA2093      TTCTGACTCACCGGACTGGGATTAGGGTTCTGATTTTGACCCGAACCCGAATTACTCGCT   597
            ************************************************************

HEINZ       CGACTACTCCTCCTCACAGGTAACTTCGCATCTCCTTTCTTCTGCTTATTATAACTCCCT   660
E42         CGACTACTCCTCCTCACAGGTAACTTCGCATCTCCTTTCTTCTGCTTATTATAACTCCCT   648
LA2093      CGACTACTCCTCCTCACAGGTAACTTCGCATCTCCTTTCTTCTGCTTATTATAACTCCCT   657
            ************************************************************

HEINZ       CTAGGTCTTCCTCGTCGAGCAGAAGGAGTCAATTTAACTCTACCAAAAAGGGAAAGTTCA   720
E42         CTAGGTCTTCCTCGTCGAGCAGAAGGAGTCAATTTAACTCTACCAAAAAGGGAAAGTTCA   708
LA2093      CTAGGTCTTCCTCGTCGAGCAGAAGGAGTCAATTTAACTCTACCAAAAAGGGAAAGTTCA   717
            ************************************************************

HEINZ       TTATCATAAAGACCCTTTTTTGATGGATCTGAAAGAACAGCCCAAGCATCAGCAACAAGG   780
E42         TTATCATAAAGACCCTTTTTTGATGGATCTGAAAGAACAGCCCAAGCATCAGCAACAAGG   768
LA2093      TTATCATAAAGACCCTTTTTTGATGGATCTGAAAGAACAGCCCAAGCATCAGCAACAAGG   777
            ************************************************************

HEINZ       CCGAAAGCAGTATCTGAAGATGGGTATTTGTTTTTATCAGGATGAAGAAGAAGAGCAAGA   840
E42         CCGAAAGCAGTATCTGAAGATGGGTATTTGTTTTTATCAGGATGAAGAAGAAGAGCAAGA   828
LA2093      CCGAAAGCAGTATCTGAAGATGGGTATTTGTTTTTATCAGGATGAAGAAGAAGAGCAAGA   837
            ************************************************************
```

```
HEINZ       CGACGATACTGTTTCTTGATAAGCTCTGAATCTTCAGTACGACCTTGGATTTGAAGAATA    900
E42         CGACGATACTGTTTCTTGATAAGCTCTGAATCTTCAGTACGACCTTGGATTTGAAGAATA    888
LA2093      CGACGATACTGTTTCTTGATAAGCTCTGAATCTTCAGTACGACCTTGGATTTGAAGAATA    897
            ************************************************************

HEINZ       GAGTACCAATCATGGTGATTGTTGATTCGTTTAACAGAAGCTAAAAGAACTTCAGCAATT    960
E42         GAGTACCAATCATGGTGATTGTTGATTCGTTTAACAGAAGCTAAAAGAACTTCAGCAATT    948
LA2093      GAGTACCAATCATGGTGATTGTTGATTCGTTTAACAGAAGCTAAAAGAACTTCAGCAATT    957
            ************************************************************

HEINZ       GCTAAGATCTGGTCAGGTCCTTCAAGTAAAGGCTCAGTTTCTTGAGCAAGAAGTGCAAAA    1020
E42         GCTAAGATCTGGTCAGGTCCTTCAAGTAAAGGCTCGGTTTCTTGAGCAAGAAGTGCAAAA    1008
LA2093      GCTAAGATCTGGTCAGGTCCTTCAAGTAAAGGCTCGGTTTCTTGAGCAAGAAGTGCAAAA    1017
            *********************************** ************************

HEINZ       TCTTTACAAGGGGCAAATTCTTTATCTCTTAACAGCTTTTCTGCAACTCCTAACAGTCTC    1080
E42         TCTTTACAAGGGGCAAATTCTTTATCTCTTAACAGCTTTTCTGCAACTCCTAACAGTCTC    1068
LA2093      TCTTTACAAGGGGCAAATTCTTTATCTCTTAACAGCTTTTCTGCAACTCCTAACAGTCTC    1077
            ************************************************************

HEINZ       TCTGCTTCTGCTCTGCTTGTAGTTGTATGATGCTCCAT    1118
E42         TCTGCTTCTGCTCTGCTTGTAGTTGTATGATGCTCCAT    1106
LA2093      TCTGCTTCTGCTCTGCTTGTAGTTGTATGATGCTCCAT    1115
            **************************************
```

Solyc04g077430

```
E42             CAGAGAAACAAAAACGCGAAGTTGAAAGAAGAAGAAAATAGAGAAAATACAAAGTACAAT     60
HEINZ           CAGAGAAACAAAAACGCGAAGTTGAAAGAAGAAGAAAATAGAGAAAATACAAAGTACAAT     60
LA2093          CAGAGAAACAAAAACGCGAAGTTGAAAGAAGAAGAAAATAGAGAAAATACAAAGTACAAT     60
                ************************************************************

E42             TTCATCTAAAGTCGGCGACGGCGTAGAGAAGAAGGAGGAACGATTATATAAAGCACACAG     120
HEINZ           TTCATCTAAAGTCGGCGACGGCGTAGAGAAGAAGGAGGAACGATTATATAAAGCACACAG     120
LA2093          TTCATCTAAAGTCGGCGACGGCGTAGAGAAGAAGGAGGAACGATTTTATTAAGCACACAG     120
                ********************************************* *** **********

E42             CATAGTATGTTTCATCGGCTCTTTTTCCAGTTTATTACAGTGATTACAATTCAAAGCTAA     180
HEINZ           CATAGTATGTTTCATCGGCTCTTTTTCCAGTTTATTACAGTGATTACAATTCAAAGCTAA     180
LA2093          CATAGTATGATTCATCGGCTCTTTTTCCAGTTTATTACAGTGATTACAATTCAAAGCTAA     180
                ********* **************************************************

E42             ATAAATTATGGGTAACGAACAAGACGATGAAGACAATAGGGGAAAACCTTATCAAGAAAA     240
HEINZ           ATAAATTATGGGTAACGAACAAGGCGATGAAGACAATAGGGGAAAACCTTATCAAGAAAA     240
LA2093          ATAAATTATGGGTAACGAACAAGGCGATGAAGACAATAGGGGAAAACCTTATCAAGAAAA     240
                *********************** ************************************

E42             AGATGAGATGAAACTTTGGGGTATTTTTGTTTTTGCATTGATCGGAGCTACTGCCACAAC     300
HEINZ           AGATGAGATGAAACTTTGGGGTATTTTTGTTTTTGCATTAATCGGAGCTACTGCCACAAC     300
LA2093          AGATGAGATGAAACTTTGGGGTATTTTTGTTTTTGCATTAATCGGAGCTACTGCCACAAC     300
                *************************************** ********************

E42             TTTTGCGGTAGGTTCTTACCCCATCTTTTTTCTTTTTTTTTTTTGTTTCTCTACTTTTTT     360
HEINZ           TTTTGCGGTAGGTTCTTACCCCATCTTTTTTCTTTTTTTTTTTTGTTTCTCTACTTTTTT     360
LA2093          TTTTGCGGTAGGTTCTTACCCCCATCTTTTT---TTTTTTTTTTGTTTCTCTACTTTTTT     357
                **********************    *****   **************************

E42             TCATTTATTTGAATTTGGTTTTGCAATTCAGTGGGTTCTGTAAAGTTAGAAGCTTTATTG     420
HEINZ           TCATTTATTTGAATTTGGTTATGCAATTCAGTGGGTTCTGTAAAGTTAGAAGCTTTATTG     420
LA2093          TCATTTATTTGAATTTGGTTTTGCAATTCAGTGGGTTCTGTAAAGTTAGAAGCTTTATTG     417
                ******************** ***************************************

E42             AGTAATCTTGAAATCTATGATCTGTAATATCAGATATGATGTGATAATTGGGCATTTAGG     480
HEINZ           AGTAATGTTGAAATCTATGATCTGTAATATCAGATATGATGTGATAATTGGGCATTTAGG     480
LA2093          AGTAATCTTGAAATCTATGATCTGTAATATCAGATATGATGTGATAATTGGGCATTTAGG     477
                ****** *****************************************************

E42             GAAAATGGGTTGTGAAGAAACCAAAGTGTATGCTTGATCAAACTGGGAAAGAAAGAAACT     540
HEINZ           GAAAATGGGTTGTGAAGAAACCAAAGTGTATGCTTGATCAAACTGGGAAAGAAAGAAACT     540
LA2093          GAAAATGGGTTGTGAAGAAACCAAAGTGTATGCTTGATCAAACTGGGAAAGAAAGAAACT     537
                ************************************************************

E42             TGGGGATATTTAGGTTTCTAATTTTTTTATGCTCAGTTGTGAACAAAATTTGCCTTGAT     600
HEINZ           TGGGGATATTTAGGTTTCTAATTTTTTTATGCTCAGTTGTGAACAAAATTTGCCCTGAT     600
LA2093          TGGGGATATTTAGGTTTCTAATTTTTTTATGCTCAGTTGTGAACAAAATTTGCCCTGAT     597
                ******************************************************* ****

E42             TAATTACCATAAGTAGTAGTTATTCTGCCATTTGAGTTAATTATTTTTGAAAACCACAAT     660
HEINZ           TAATTACCATAAGTAGTAGTTATTCTGCCATTTGAGTTAATTATATTCGAAAACCACAAT     660
LA2093          TAATTACCATAAGTAGTAGTTATTCTGCCATTTGAGTTAATTATATTCGAAAACCACAAT     657
                ******************************************** ** ************

E42             GATGCTAATTTGCTTACAGGTTAATATGTTGTAACTTTGGGGATGACCATCATTTGTCAA     720
HEINZ           GATGCTAATTTGCTTACAGGTTAATATGTTGTAACTTTGGGGATGACCATCATTTGTCAA     720
LA2093          GATGCTAATTTGCTTACAGGTTAATATGTTGTAACTTTGGGGATGACCATCATTTGTCAA     717
                ************************************************************

E42             TGTTTTTATTGAAACCCCCTTACTGTATTTGAAAAAAATTTGCTCGTCTCATTACCATAA     780
HEINZ           TGTTTTTATTGAAACCCCCTTACTGTATTTGAAAAAAATTTGCTCGTCTCATTACCATAA     780
LA2093          TGTTTTTATTGAAACCCCCTTACTGTATTTGAAAAAAATTTGCTCGTCTCATTACCATAA     777
                ************************************************************

E42             ATAATATATTCGTTTCCTGGATTAGGAGCCAAGGTGTTTGAGCATTATGTCCTACACCAT     840
HEINZ           ATAATATATTCGTTTCCTGGATTAGGAGCCAAGGTGTTTGAGCATTATGTCCTACACCAT     840
LA2093          ATAATATATTCGTTTCCTGGATTAGGAGCCAAGGTGTTTGAGCATTATGTCCTACACCAT     837
                ************************************************************
```

```
E42            CAAATTCCGTGTAACTCTGCAGCTAAGATGTGATTTAGATATTCAAAGTCACCAGTGAAC     900
HEINZ          CAAATTCCGTGTAACTCTGCAGCTAAGATGTGATTTAGATATTCAAAGTCACCAGTGAAC     900
LA2093         CAAATTCCGTGTAACTCTGCAGCTAAGATGTGATTTAGATATTCAAAGTCACCAGTGAAC     897
               ************************************************************

E42            TTGTAGTGTAAAGTGGTGTTTTTTGTAGAGGGGAATCTTTCTAAGAAGTGGCAGGGTGTG     960
HEINZ          TTGTAGTGTAAAGTGGTGTTTTTTGTAGAGGGGAATCTTTCTAAGAAGTGGCAGGGTGTG     960
LA2093         TTGTAGTGTAAAGTGGTGTTTTTTGTAGAAGGGAATCTTTCTAAGAAGTGGCAGGGTGTG     957
               ***************************** ******************************

E42            GGACCACCATAGATGGTAGGTGCTGGTTATATCTATCCAGAATGAGAATGAGCTTCTGTT     1020
HEINZ          GGACCACCATAGATGGTAGGTGCTGGTTATATCTATCCAGAATGAGAATGAGCTTCTGTT     1020
LA2093         GGACCACCATAGATGGTAGGTGCTGGTTATATCTATCCAGAATGAGAATGAGCTTCTGTT     1017
               ************************************************************

E42            TTCAGTTTCAGCTGATGAAACAAAAAGCCAGAGATGATACCTGAGGTCATGTTCCTTTGA     1080
HEINZ          TTCAGTTTCAGCTGATGAAACAAAAAGCCAGAGATGATACCTGAGGTCATGTTCCTTTGA     1080
LA2093         TTCAGTTTCAGCTGATGAAACAAAAAGCCAGAGATGATACCTGAGGTCATGTTCCTTTGA     1077
               ************************************************************

E42            TGGGTTCAACAGAAAGATTCAGTCACCAAAGCTGGTTTGCTGTTTGCAAGTTAGGTGTAC     1140
HEINZ          TGGGTTCAACAGAAAGATTCAGTCACCAAAGCTGGTTTGCTGTTTGCAAGTTAGGTGTAC     1140
LA2093         TGGGTTCAACAGAAAGATTCAGTCACCAAAGCTGGTTTGCTGTTTGCAAGTTAGGTGTAC     1137
               ************************************************************

E42            GTCATGGTTCGAACCATGTCGTCGCAGGTAAATTTTGTTATTTAAGTAGAGACGGGTAAA     1200
HEINZ          GTCATGGTTCGAACCATGTCGTCGCAGGTAAATTTTGTTATTTAAGTAGAGACGGGTAAA     1200
LA2093         GTCATGGTTCGAACCATGTCGTCGCAGGTAAATTTTGTTATTTAAGTAGAGACGGGTAAA     1197
               ************************************************************

E42            GGTGCAGGCCCATTATTTGCCTACTTTCTGACCGTGCGCTAGTGACCCTTGGAGGTTTCT     1260
HEINZ          GGTGCAGGCCCATTATTTGCCTAGTTTCTGACCGTGCGCTAGTGACCCTTGGAGGTTTCT     1260
LA2093         GGTGCAGGCCCATTATTTGCCTAGTTTCTGACCGTGCGCTAGTGACCCTTGGAGGTTTCT     1257
               *********************** ************************************

E42            CGATTATAAAAGAAGGATGGCAATACGATATCTTTTGGTGGAGAAGAAAATATCTCTCTA     1320
HEINZ          CGATTATAAAAGAAGGATGGCAATACGATATCTTTTGGTGGAGAAGAAAATATCTCTCTA     1320
LA2093         CGATTATAAAAGAAGGATGGCAATACGATATCTTTTGGTGGAGAAGAAAATATCTCTCTA     1317
               ************************************************************

E42            TGGTTTGGGGGAGGGTTGGAGGATTGGGGAAACAAATAATCATCTCTTCCTGCATTGTAA     1380
HEINZ          TGGTTTGGGGGAGGGTTGGAGGATTGGGGAAACAAATAATCATCTCTTCCTGCACTGTAA     1380
LA2093         TGGTTTGGGGGAGGGTTGGAGGATTGGGGAAACAAATAATCATCTCTTCCTGCACTGTAA     1377
               ****************************************************** *****

E42            GTTTACTGCTCAAATTTGGAACTTGTTTCTCAACATCACAAGCATGAACTGGACAATGCC     1440
HEINZ          GTTTACTGCTCAAATTTGGAACTTGTTTCTCAACATCACAAGCATGAACTGGACAACGCC     1440
LA2093         GTTTACTGCTCAAATTTGGAACTTGTTTCTCAACATCACAAGCATGAACTGGACAACGCC     1437
               ******************************************************** ***

E42            AGAACATACATCAGATTTGTTGAGCTGTTGGATTAGGAGGGGAGGCAGTAAGAGTCAAAA     1500
HEINZ          AGAACATACATCAGATTTGTTGAGCTGTTGGATTAGGAGGGGAGGCAGTAAGAGTCAAAA     1500
LA2093         AGAACATACATCAGATTTGTTGAGCTGTTGGATTAGGAGGGGAGGCAGTAAGAGTCAAAA     1497
               ************************************************************

E42            GAGATGGTGGAAGCTAATACCATCATGTGTATGGTGGTCAGTCTGGAAAGAAAGAAATGG     1560
HEINZ          GAGATGGTGGAAGCTAATATCATCATGTGTATGGTGGTCAGTCTGGAAAGAAAGAAATGG     1560
LA2093         GAGATGGTGGAAGCTAATATCATCATGTGTATGGTGGTCAGTCTGGAAAGAAAGAAATGG     1557
               ******************* ****************************************

E42            AAAATGTTCTGAAGATAGATCCAATTCCATCCACAAAGTAAAATGGAGTTGTATAGTATC     1620
HEINZ          AAAATGTTTTGAAGATAGATCCAATTCCATCCACAAAGTAAAATGGAGTTGTATAGTATC     1620
LA2093         AAAATGTTTTGAAGATAGATCCAATTCCATCCACAAAGTAAAATGGAGTTGTATAGTATC     1617
               ******** ***************************************************

E42            TCTACTTTTTTGGTGTAAACAACTTTGTATAGATGATATAGACCAGATTATAGATTTGAT     1680
HEINZ          TCTACTTTTTTGGTGTAAACAACTTTGTATAGATGATATAGACCAGATTATAGATTTGAT     1680
LA2093         TCTACTTTTTTGGTGTAAACAACTTTGTATAGATGATATAGACCAGATTATAGATTTGAT     1677
               ************************************************************

E42            TGGAAATCTGTAATTATTCCTTTTTGGAGGATTAGTGATGAAGAGATCGAAATGATTTAA     1740
```

```
HEINZ      TGGAAATCTGTAATTATTCCTTTTTGGAGGATTAGTGATGAAGAGATCGAAATGATTTAA    1740
LA2093     TGGAAATCTGTAATTATTCCTTTTTGGAGGATTAGTGATGAAGAGATCGAAATGATTTAA    1737
           ************************************************************

E42        TATGGCACTAATGGAAAAATGTCTTTTTTACATAGAGACATTATCAGGAAGTCGTTGAAT    1800
HEINZ      TATGGCACTAATGGAAAAATGTCTTTTTTACATAGAGACATTATCAGGAAGTCGTTGAAT    1800
LA2093     TATGGCACTAATGGAAAAATGTCTTTTTTACATAGAGACATTATCAGGAAGTCGTTGAAT    1797
           ************************************************************

E42        GTTGCCAGATACAAATTGTCAGGTAGTGGAAGATAAGATGACCTTACGAGTTTTCTATTT    1860
HEINZ      GTTGCCAGATACAAATTGTCAGGTAGTGGAAGATAAGATGACCTTACGAGTTTTCTATTT    1860
LA2093     GTTGCCAGATACAAATTGTCAGGTAGTGGAAGATAAGATGACCTTACGAGTTTTCTATTT    1857
           ************************************************************

E42        ATTGATAAGTTTGTGAAAAGTTTGTCTCCCGGCAACGGAATGATATAACCTTTGTTATCT    1920
HEINZ      ATTGATAAGTTTGTGAAAAGTTTGTCTCCCGGCAACGGAATGATATAACCTTTGTTATCT    1920
LA2093     ATTGATAAGTTTGTGAAAAGTTTGTCTCCCGGCAACGGAATGATATAACCTTTGTTATCT    1917
           ************************************************************

E42        TTTGGTCACTAGAACAAAGGAGATTGTGAAGGGAAAAGATGATTTTACAAAGTTCATTGT    1980
HEINZ      TTTGGTCATTAGAACAAAGGAGATTGTGAAGGGAAAAGATGATTTTACAAAGTTCATTGT    1980
LA2093     TTTGGTCATTAGAACAAAGGAGATTGTGAAGGGAAAAGATGATTTTACAAAGTTCATTGT    1977
           ******** ***************************************************

E42        CATGACAATGACCCTTAATGTGGAACTGTCGGGACTCGGAGTGACAACCATCTACGTTTT    2040
HEINZ      CATGACAATGACCCTTAATGTGGAACTGTCGGGACTCGGAGTGACAACCATCTACGTTTT    2040
LA2093     CATGACAATGACCCTTAATGTGGAACTGTCGGGACTCGGAGTGACAACCATCTACGTTTT    2037
           ************************************************************

E42        TGGTTGGATAGCTTGTGTGGAGACGCTGAAGTATGCATATACTTCGGGTGTTAGCCATTC    2100
HEINZ      TGGTTGGATAGCTTGTGTGGAGACGCTGAAGTATGCATATACTTCGGGTGTTAGCCATTC    2100
LA2093     TGGTTGGATAGCTTGTGTGGAGACGCTGAAGTATGCATATACTTCGGGTGTTAGCCATTC    2097
           ************************************************************

E42        TACATAGATTGGGTAATATATCCGAGTTTGTCACATCTCACAAGAAACATGTAGTAATAT    2160
HEINZ      TACATAGATTGGGTAATATATCCGAGTTTGTCACATCTCACAAGAAACATGTAGTAATAT    2160
LA2093     TACATAGATTGGGTAATATATCCGAGTTTGTCACATCTCACAAGAAACATGTAGTAATAT    2157
           ************************************************************

E42        CTCAGAAATTTGTTTCAAAAGAGTAATTTTGTATTGGGAATGAGAAGTGGAGTTCATGCA    2220
HEINZ      CTCAGAAATTAGTTTCAAAAGAGTAATTTTGTATTGGGAATGAGAAGTGGAGTTCATGCA    2220
LA2093     CTCAGAAATTAGTTTCAAAAGAGTAATTTTGTATTGGGAATGAGAAGTGGAGTTCATGCA    2217
           ********** *************************************************

E42        AAGGAGTATGGGAGATAAGTTAGATAGATTGGAATGGAGGGCTTTGGGTATTTGCATGTA    2280
HEINZ      AAGGAGTATGGGAGATAAGTTAGATAGATTGGAATGGAGGGCTTTGGGTATTTGCATGTA    2280
LA2093     AAGGAGTATGGGAGATAAGTTAGATAGATTGGAATGGAGGGCTTTGGGTATTTGCATGTA    2277
           ************************************************************

E42        TTGACGAAAGGCATCCTCTAAGAAGAAGGAGGGGGGGGGGGGGGGGGGGGGCCAAAGGTG    2340
HEINZ      TTGACGAAAGGCATCCTCTAAGAAGAAGGAAGGGGGGGGAGGGGGGGGGGGGCAAAGGTG    2340
LA2093     TTGACGAAAGGCATCCTCTAAGAAGAAGGAAGGGGGGGGA----GGGGGGGGCAAAGGTG    2333
           ****************************** ********     ******* ********

E42        GAAAAATATATGGATATTTTTTATGACAAGGGATCCTGCAACCGATATTTTTTGGGTGCA    2400
HEINZ      GAAAAATATATGGATATTTTTTATGACAAGGGATCCCGCAACCGATATTTTTTGGGTGCA    2400
LA2093     GAAAAATATATGGATATTTTTTATGACAAGGGATCCCGCAACCGATATTTTTTGGGTGCA    2393
           ************************************ ***********************

E42        CACAGGGTAAACCTCGCTCCACTGTAATAGCCCGCTAACCTCACAGGAGAGATAAACCGC    2460
HEINZ      CACAGGGTAAACCTCGCTCCACTGTAATAGCCCGCTAACCTCACAGGAGAGATAAACCGC    2460
LA2093     CACAGGGTAAACCTCGCTCCACTGTAATAGCCCGCTAACCTCACAGGAGAGATAAACCGC    2453
           ************************************************************

E42        ACTAGGAAATCCCTTACTACACGCTCAACCAAGAAAGCATGCAAGGGGTTTCAAGCCTAA    2520
HEINZ      ACTAGGAAATCCCTTACTACACGCTCAACCAAGAAAGCATGCAAGGGGTTTCAAGCCTAA    2520
LA2093     ACTAGGAAATCCCTTACTACACGCTCAACCAAGAAAGCATGCAAGGGGTTTCAAGCCTAA    2513
           ************************************************************

E42        GACCCATCATTGGGAGATTTCCTGCTCAACCAACTCGGCCACCTTTACAGGTGGAAGAAG    2580
HEINZ      GACCCATCATTGGGAGATTTCCTGCTCAACCAACTCGGCCACCTTTACAGGTGGAAGAAG    2580
LA2093     GACCCATCATTGGGAGATTTCCTGCTCAACCAACTCGGCCACCTTTACAGGTGGAAGAAG    2573
```

```
            ************************************************************

E42         ATACGAATAGAAAATCATATGTCCTTAGTGTATTTCAACTCTTTGTTATTAAGGATGGCG    2640
HEINZ       ATACGAATAGAAAATCATATGTCCTTAGTGTATTTCAACTCTTTGTTATTAAGGATGGCG    2640
LA2093      ATACGAATAGAAAATCATATGTCCTTAGTGTATTTCAACTCTTTGTTATTAAGGATGGCG    2633
            ************************************************************

E42         TACTATTCAGTGACATTTGTATAGTCTCCTTCTCTTAGAAACTTGTTTAGTGGATTCTAC    2700
HEINZ       TACTATTCAGTGACATTTGTATAGTCTCCTTCTCTTAGAAACTTGTTCAGTGGATTCTAC    2700
LA2093      TACTATTCAGTGACATTTGTATAGTCTCCTTCTCTTAGAAACTTGTTCAGTGGATTCTAC    2693
            *********************************************** ************

E42         AAATTGAAGTTGATTAGGTCTGATATTTTT-TTCCCGGGTGTGGTTCGTTGTTGCTTCTG    2759
HEINZ       AAATTGAAGTTGATTAGGTCTGATATTTTTTTTCCCGGGTGTGGTTCGTTGTTGCTTCTG    2760
LA2093      AAATTGAAGTTGATTAGGTCTGATATTTTTTTTCCCGGGTGTGGTTCGTTGTTGCTTCTG    2753
            ****************************** *****************************

E42         ACCACTTTAAACTTTCATGCTCTTCTGTGTTCTGTTAGAACAATGAAAAAGTTTTAGAGG    2819
HEINZ       ACCACTTTAAACTTTCATGCTCTTCTGTGTTCTGTTAGAACAATGAAAAAGTTTTAGAGG    2820
LA2093      ACCACTTTAAACTTTCATGCTCTTCTGTGTTCTGTTAGAACAATGAAAAAGTTTTAGAGG    2813
            ************************************************************

E42         AATTTTGTAGCTTTCCTTGCTTCTTCCTTTTTTCCGCCTCTGGGTTGCTTCTGATTATTA    2879
HEINZ       AATTTTGTAGCTTTCCTTGCTTCTTCCTTTTTTCCGCCTCTGGGTTGCTTCTGATTATTA    2880
LA2093      AATTTTGTAGCTTTCCTTGCTTCTTCCTTTTTTCCGCCTCTGGGTTGCTTCTGATTATTA    2873
            ************************************************************

E42         ATTCACAGCCTGAGAAATTTGGCAGGTTACACAATTGCGAAGTACAGTGGATTTTGTCTA    2939
HEINZ       ATTCACAGCCTGAGAAATTTGGCAGGTTACACAATTGCGAAGTACAGTGGATTTTGTCTA    2940
LA2093      ATTCACAGCCTGAGAAATTTGGCAGGTTACACAATTGCGAAGTACAGTGGATTTTGTCTA    2933
            ************************************************************

E42         CTCTCAGGTAATGAGCTTTTTTGTTCAATCTCATTGGGTAAATGTAGAATTTCAGTTTAC    2999
HEINZ       CTCTCAGGTAATGAGCTTTTTTGTTCAATCTCATTGGGTAAATGTAGAATTTCAGTTTAC    3000
LA2093      CTCTCAGGTAATGAGCTTTTTTGTTCAATCTCATTGGGTAAATGTAGAATTTCAGTTTAC    2993
            ************************************************************

E42         TCCTGAGTTACCCTCCCTGCATGTGGTCTATGTTATGACCTATTTGCTTGTTGCTTTTGC    3059
HEINZ       TCCTGAGTTACCCTCCCTGCATGTGGTCTATGTTATGACCTATTTGCTTGTTGCTTTTGC    3060
LA2093      TCCTGAGTTACCCTCCCTGCATGTGGTCTATGTTATGACCTATTTGCTTGTTGCTTTTGC    3053
            ************************************************************

E42         ATAATCATAGTGAGCTACCTTTGCTTCTTCTTTTGACTACCTCTTATCTGGTTTTCTTGT    3119
HEINZ       ATAATCATAGTGAGCTACCTTTGCTTCTTCTTTTGACTACCTCTTATCTGGTTTTCTTGT    3120
LA2093      ATAATCATAGTGAGCTACCTTTGCTTCTTCTTTTGACTACCTCTTATCTGGTTTTCTTGT    3113
            ************************************************************

E42         CTTTTTCTTGTAATTTTTATGTAGCACCAAATAGGACAGCAAAAATAAGGGAAGATATGA    3179
HEINZ       CTTTTTCTTGTAATTTTTATGTAGCACCAAATAGGACAGCAAAAATAAGGGAAGATATGA    3180
LA2093      CTTTTTCTTGTAATTTTTATGTAGCACCAAATAGGACAGCAAAAATAAGGGAAGATATGA    3173
            ************************************************************

E42         GAAAAGGCAAAAATGAGAGAGAGAGAGAGAGAGAGGAAGGAGAGGGGGTGGATAGGAGAAGG    3239
HEINZ       GAAAAGGCAAAAATGAGAGAGAGAGAGAGAGAGAGGAAGGAGAGGGGGTGGATAGGAGAAGG    3240
LA2093      GAAAAGGCAAAAATGAGAGAGAGAGAGAGAGAGAGGAAGGAGAGGGGGTGGATAGGAGAAGG    3233
            ************************************************************

E42         GAGCAGGAATAAGAGGATAAAGAGAAGAATTTTTATATGCAATCAATTAATACAATATTA    3299
HEINZ       GAGCAGGAATAAGAGGATAAAGAGAAGAATTTTTATATGCAATCAATTAATACAATATTA    3300
LA2093      GAGCAGGAATAAGAGGATAAAGAGAAGAATTTTTATATGCAATCAATTAATACAATATTA    3293
            ************************************************************

E42         TTAGTCTCTCGACCAAAATGCACTAGGGTCCAATTTATAAATAGGGAATATTGAGCAAGT    3359
HEINZ       TTAGTCTCTCGACCAAAATGCACTAGGGTCCAATTTATAAATAGGGAATACTGAGCAAGT    3360
LA2093      TTAGTCTCTCGACCAAAATGCACTAGGGTCCAATTTATAAATAGGGAATACTGAGCAAGT    3353
            ************************************************** *********

E42         GAAAACTGGAGACAACAAAACCTATTTCTCTAACTAAAACTGAAAGATAAGACTATTGCT    3419
HEINZ       GAAAACTGGAGACAACAAAACCTATTTCTCTAACTAAAACTGAAAGATAAGACTATTGCT    3420
LA2093      GAAAACTGGAGACAACAAAACCTATTTCTCTAACTAAAACTGAAAGATAAGACTATTGCT    3413
            ************************************************************
```

```
E42        AACCAACAGATACGACCTTTTAATGAAAAAATAGTATTATATCTAACTCAGAATTCTTTT    3479
HEINZ      AACCAACAGATATGACCTTTTAATGAAAAAATAGTATTATATCTAACTCAGAATTCTTTT    3480
LA2093     AACCAACAGATATGACCTTTTAATGAAAAAATAGTATTATATCTAACTCAGAATTCTTTT    3473
           ************ ***********************************************

E42        TTGCCTTCTAAAAATTAAGAAGATTGACTTGCTCAAACAAAAAAATTAAGAAGATTGGCA    3539
HEINZ      TTGCCTTCTAAAAATTAAGAAGATTGACTTGCTCAAACAAAAAAATTAAGAAGATTGGCA    3540
LA2093     TTGCCTTCTAAAAATTAAGAAGATTGACTTGCTCAAACAAAAAAATTAAGAAGATTGGCA    3533
           ************************************************************

E42        TGGCGCATGCACAAAAAATGACACCCATAAATCGAGATGATGTTTAATCTTAAATTAGAA    3599
HEINZ      TGGCGCATGCACAAAAAATGACACCCATAAATCGAGATGATGTTTAATCTTAAATTAGAA    3600
LA2093     TGGCGCATGCACAAAAAATGACACCCATAAATCGAGATGATGTTTAATCTTAAATTAGAA    3593
           ************************************************************

E42        GAAAGAGATTAATGTCAAAGGCAGGAAGATGTGATATTGATGCATCGATCCCTCTTCTAC    3659
HEINZ      GAAAGAGATTAATGTCAAAGGCAGGAAGATGTGATATTGATGCATCGATCTCTCTTCTAC    3660
LA2093     GAAAGAGATTAATGTCAAAGGCAGGAAGATGTGATATTGATGCATCGATCTCTCTTCTAC    3653
           ************************************************** *********

E42        ATATTTGCTCTTGCTGATGTTCTAGATCTTCGAAGTATGTGAGTCAGTTTTAGCTCACGT    3719
HEINZ      ATATTTGCTCTTGCTGATGTTCTAGATCTTCGAAGTATGTGAGTCAGTTTTAGCTCACGT    3720
LA2093     ATATTTGCTCTTGCTGATGTTCTAGATCTTCGAAGTATGTGAGTCAGTTTTAGCTCACGT    3713
           ************************************************************

E42        CTAAACATAATTCACCACTCAGAAGATGATTGAAAAATGACTAAAAATTAGTTCTCCAAA    3779
HEINZ      CTAAACATAATTCACCACTCAGAAGATGATTGAAAAATGACTAAAAATTAGTTCTCCAAA    3780
LA2093     CTAAACATAATTCACCACTCAGAAGATGATTGAAAAATGACTAAAAATTAGTTCTCCAAA    3773
           ************************************************************

E42        GTCTTTGGTTTAGTGATGAGAGTTAAGCAATATGTGGATTAGCCACACGTAATTAGTTTG    3839
HEINZ      GTCTTTGGTTTAGTGATGAGAGTTAAGCAATATGTGGATTAGCCACACGTAATTAGTTTG    3840
LA2093     GTCTTTGGTTTAGTGATGAGAGTTAAGCAATATGTGGATTAGCCACACGTAATTAGTTTG    3833
           ************************************************************

E42        AAAGTCGTGGCAATCTGAAGAATGGTATTTAATCAGAGAAGGGTACGGAGACAGTCTCAT    3899
HEINZ      AAAGTCGTGGCAATCTGAAGAATGGTATTTAATGAGAGAAGGGTACGGAGACAGTCTCAT    3900
LA2093     AAAGTCGTGGCAATCTGAAGAATGGTATTTAATCAGAGAAGGGTACGGAGACAGTCTCAT    3893
           ********************************* **************************

E42        GATACAACGAGTTTCAAACTGTGGGCCATCTCGCCCTCGGATATTTCTTAGTCATAAAGA    3959
HEINZ      GATACAACGAGTTTCAAACTGTGGGCCATCTCGCCCTCGGATATTTCTTAGTCATAAAGA    3960
LA2093     GATACAACGAGTTTCAAACTGTGGGCCATCTCGCCCTCGGATATTTCTTAGTCATAAAGA    3953
           ************************************************************

E42        AAAAAATTAAACTGATAAATCGCTTTTTTTTAATAAGAATGAGGGGTTCAGGGAAGATCA    4019
HEINZ      AAAAAATTAAACTGATAAATCGCTTTTTTTTAATAAGAATGAGGGGTTCAGGGAAGATCA    4020
LA2093     AAAAAATTAAACTGATAAATCGCTTTTTTTTAATAAGAATGAGGGGTTCAGGGAAGATCA    4013
           ************************************************************

E42        TCAGATTCGAATTTGACATTGATTTTTAAATTTTGGGAAATAAACTTTTGCATATTTATT    4079
HEINZ      TCAGATTCGAATTTGACATTGATTTTTAAATTTTGGGAAATAAACTTTTGCATATTTATT    4080
LA2093     TCAGATTCGAATTTGACATTGATTTTTAAATTTTGGGAAATAAACTTTTGCATATTTATT    4073
           ************************************************************

E42        TACTACAGAGAAAGTTTTATAACTCAGCTTTAGAGTTAAAAAATATCAGAAAATAATTGT    4139
HEINZ      TACTACAGAGAAAGTTTTATAACTCAGCTTTAGAGTTAAAAAATATCAGAAAATAATTGT    4140
LA2093     TACTACAGAGAAAGTTTTATAACTCAGCTTTAGAGTTAAAAAATATCAGAAAATAATTGT    4133
           ************************************************************

E42        AAAAGAAACTCTTAATAATCGTGTTATTTCCTTCTGATACAGGGAGTATATGCTTACTCT    4199
HEINZ      AAAAGAAACTCTTAATAATCGTGTTATTTCCTTCTGATACAGGGAGTATATGCTTACTCT    4200
LA2093     AAAGGAAACTCTTAATAATCGTGTTATTTCCTTCTGATACAGGGAGTATATGCTTACTCT    4193
           *** ********************************************************

E42        ATGAAATGAGATGTTGCCACAAAACTAATATTGTGCAAATAATTGATTTTGCTGTTTTCA    4259
HEINZ      ATGAAATGAGATGTTGCCACAAAACTAATATTGTGCAAATAATTGATTTTGCTGTTTTCA    4260
LA2093     ATGAAATGAGATGTTGCCACAAAACTAATATTGTGCAAATAATTGATTTTGCTGTTTTCA    4253
           ************************************************************

E42        TGCAATTGTCCTGGGTGCAAGTTCAATACTAATTAACTGTTGCATGCATAAGATGAAATA    4319
HEINZ      TGCAATTGTCCTGGGTGCAAGTTCAATACTAATTAACTGTTGCATGCATAAGATGAAATA    4320
```

```
LA2093          TGCAATTGTCCTGGGTGCAAGTTCAATACTAATTAACTGTTGCATGCATAAGATGAAATA     4313
                ************************************************************

E42             CATCAAATGAAGTTGCTTATAATTATTGACATTTGTGCTTAAAGAAGGATTCTGCTCTAG     4379
HEINZ           CATCAAATGAAGTTGCTTATAATTATTGACATTTGTGCTTAAAGAAGGATTCTGCTCTAG     4380
LA2093          CATCAAATGAAGTTGCTTATAATTATTGACATTTGTGCTTAAAGAAGGATTCTGCTCTAG     4373
                ************************************************************

E42             TTGTTCTGATAAATACTTTGCATGGGGGAGGAAAGAAATGTTTTTCTTGAGCTAATAACT     4439
HEINZ           TTGTTCTGATAAATACTTCGCATGGGGGAGGAAAGAAATGTTTTTCTTGAGCTAATAACT     4440
LA2093          TTGTTCTGATAAATACTTTGCATGGGGGAGGAAAGAAATGTTTTTCTTGAGCTAATAACT     4433
                ****************** *****************************************

E42             ACTTATTTGAATTGAAAAAGTTGAGGCAGTCAGCACAGAGAAGCCGAGCTGGCGGTTCTT     4499
HEINZ           ACTTATTTGAATTGAAAAAGTTGAGGCAGTCAGCACAGAGAAGTCGAGCTGGCAGTTCTT     4500
LA2093          ACTTATTTGAATTGAAAAAGTTGAGGCAGTCAGCACAGAGAAGTCGAGCTGGCGGTTCTT     4493
                ******************************************* ********* ******

E42             TTAGGACAAGTTTTCAGGAGGAAGCATGGAAAAGATACAATCGCAGGATGCGAGAGGAGT     4559
HEINZ           TTAGGACAAGTTTTCAGGAGGAAGCATGGAAAAGATACAATCGCAGGATGCGAGAGGAGT     4560
LA2093          TTAGGACAAGTTTTCAGGAGGAAGCATGGAAAAGATACAATCGCAGGATGCGAGAGGAGT     4553
                ************************************************************

E42             ATGAAGAAGAAATGGAGAGAGTGGTGAGCTACATTTTTCATCTTCAACAATTTTACTCTT     4619
HEINZ           ATGAAGAAGAAATGGAGAGAGTGGTGAGCTACATTTTTCATCTTCAACAATTTTACTCTT     4620
LA2093          ATGAAGAAGAAATGGAGAGAGTGGTGAGCTACATTTTTCATCTTCAACAATTTTACTCTT     4613
                ************************************************************

E42             TATTTCCTTATATCTAATTGAAAGAGAATATCGTCAAAGTTTATTTCAACTAGAAGGAAG     4679
HEINZ           TATTTCCTTATATCTAATTGAAAGAGAATATCGTCAAAGTTTATTTCAACTAGAAGGAAG     4680
LA2093          TATTTCCTTATATCTAATTGAAAGAGAATATCGTCAAAGTTTATTTCAACTAGAAGGAAG     4673
                ************************************************************

E42             TGAAGACATTGGTGATATTTGCTTTATGAAATCCAGGCCCCTATGTGTAATCATTTATAA     4739
HEINZ           TGAAGACATTGGTGATATTTGCTTTATGAAATCCAGGCCCCTATGTGTAATCATTTATAA     4740
LA2093          TGAAGACATTGGTGATATTTGCTTTATGAAATCCAGGCCCCTATGTGTAATCATTTATAA     4733
                ************************************************************

E42             TGTGTGCCGTCTCTGAATATAATTTGGCCGCGTCGAGAAATTAACTACCATTTATTTTGA     4799
HEINZ           TGTGTGCCGTCTCTGAATATAATTTGGCCGCGTCGAGAAATTAACTACCATTTATTTTGA     4800
LA2093          TGTGTGCCGTCTCTGAATATAATTTGGCCGCGTCGAGAAATTAACTACCATTTATTTTGA     4793
                ************************************************************

E42             GAAGGAGAATTTAACTACAATTTGTTCTTCTGGACATAAGCATAACTTGCTTTCTTGGAC     4859
HEINZ           GAAGGAGAATTTAACTACAATTTGTTCTTCTGGACATAAGCATAACTTGCTTTCTTGGAC     4860
LA2093          GAAGGAGAATTTAACTACAATTTGTTCTTCTGGACATAAGCATAACTTGCTTTCTTGGAC     4853
                ************************************************************

E42             AAGCTGTGCGAAATTTTTTATGGAAAATCTTATAGCTAGAAGGTTGGAGGACGGACATAA     4919
HEINZ           AAGCTGTGGGAAATTTTTTATGGAAAATCTTATAGCTAGAAGGTTGGAGGACGGACATAA     4920
LA2093          AAGCTGTGCGAAATTTTTTATGGAAAATCTTATAGCTAGAAGGTTGGAGGACGGACATAA     4913
                ******** ***************************************************

E42             AATTTACCCATATTTCTTCATCTGCAATAATACACTGAAGTTGTACAGAACCATTGGATA     4979
HEINZ           AATTTACCCATATTTCTTCATCTGCAATAATACACTGAAGTTGTACAGAACCATTGGATA     4980
LA2093          AATTTACCCATATTTCTTCATCTGCAATAATACACTGAAGTTGTACAGAACCATTGGATA     4973
                ************************************************************

E42             TTTAACAAAGTTGAACACTCGTCTTATTTGTATTTCTTTCAAGTGAAATGTCACTCTTAC     5039
HEINZ           TTTAACAAAGTTGAACACTCGTCTTATTTGTATTTCTTTCAAGTGAAATGTCACTCTTAC     5040
LA2093          TTTAACAAAGTTGAACACTCGTCTTATTTGTATTTCTTTCAAGTGAAATGTCACTCTTAC     5033
                ************************************************************

E42             CCTATGTTTTCTTTGCCTGATTGAACTGTTGTGCTTTATTTTCTATCCCATAATTTGTGA     5099
HEINZ           CCTATGTTTTCTTTGCCTGATTGAACTGTTGTGCTTTATTTTCTATCCCATAATTTGTGA     5100
LA2093          CCTATGTTTTCTTTGCCTGATTGAACTGTTGTGCTTTATTTTCTATCCCATAATTTGTGA     5093
                ************************************************************

E42             CCTTCACAAAGACAACTACAGATAGCAGATCTAAAGACTTACATATCTGAGCTAAGGAAA     5159
HEINZ           CCTTCACAAAGACAACTACAGATAGCAGATCTAAAGACTTACATATCTGAGCTAAGGAAA     5160
LA2093          CCTTCACAAAGACAACTACAGATAGCAGATCTAAAGACTTACATATCTGAGCTAAGGAAA     5153
                ************************************************************
```

```
E42         AACATAATGAGATCACAGATAAACTTCCATTTGTGAGTTTACTCTTGACCTAATATCCTG     5219
HEINZ       AACATAATGAGATCACAGATAAACTTCCATTTGTGAGTTTACTCTTGACCTAATATCCTG     5220
LA2093      AACATAATGAGATCACAGATAAACTTCCATTTGTGAGTTTACTCTTGACCTAATATCCTG     5213
            ************************************************************

E42         TGCTTTTCGTAGGAGCGCATTCGCCGTATGCAAAGTGTTTTCAACAGGGAGAGAAACAAA     5279
HEINZ       TGCTTTTCGTAGGAGCGCATTCGCCGTATGCAAAGTGTTTTCAACAGGGAGAGAAACAAA     5280
LA2093      TGCTTTTCGTAGGAGCGCATTCGCCGTATGCAAAGTGTTTTCAACAGGGAGAGAAACAAA     5273
            ************************************************************

E42         TATAAGAGGAGCTATGAGAGCTGGCAAGAAAATGGGCAAAGTGCATATCATCAGCACTTC     5339
HEINZ       TATAAGAGGAGCTATGAGAGCTGGCAAGAAAATGGGCAAAGTGCATATCATCAGCACTTC     5340
LA2093      TATAAGAGGAGCTATGAGAGCTGGCAAGAAAATGGGCAAAGTGCATATCATCAGCACTTC     5333
            ************************************************************

E42         CAAAGAAATGACTGGTACTGGAAGGCTGATACATCGTTCAGAGACCGGGGAACTAATTTC     5399
HEINZ       CAAAGAAATGACTGGTACTGGAAGGCTGATACATCGTTCAGAGACCGGGGAACTAATTTC     5400
LA2093      CAAAGAAATGACTGGTACTGGAAGGCTGATACATCGTTCAGAGACCGGGGAACTAATTTC     5393
            ************************************************************

E42         AGGGAAGCTCCTAGGGCCAATGCAAGCAACCCTCTGTCGCATCATTACTCAGTTTTGGGT     5459
HEINZ       AGGGAAGCTCCTAGGGCCAATGCAAGCAACCCTCTGTCGCATCATTACTCAGTTTTGGGT     5460
LA2093      AGGGAAGCTCCTAGGGCCAATGCAAGCAACCCTCTGTCGCATCATTACTCAGTTTTGGGT     5453
            ************************************************************

E42         CTTGATAGGTACTAATTTTTTTCAGATGTTGAATTGATCTATATTTATACTGGTTACGCA     5519
HEINZ       CTTGATAGGTACTAACTTTTTTCAGATGTTGAATTGATCTATATTTATACTGGTTACGCA     5520
LA2093      CTTGATAGGTACTAACTTTTTTCAGATGTTGAATTGATCTATATTTATACTGGTTACGCA     5513
            *************** ********************************************

E42         TGTTATCGCTAATCAGATATGCTGTTTCAGTCTTCCAATTGTCTTAGCTTTAGCATCCTT     5579
HEINZ       TGTTATCGCTAATCAGATATGCTGTTTCAGTCTTCCAATTGTCTTAGCTTTAGCATCCTT     5580
LA2093      TGTTATCGCTAATCAGATATGCTGTTTCAGTCTTCCAATTGTCTTAGCTTTAGCATCCTT     5573
            ************************************************************

E42         AAGGATATCAGTGTAGTAGTGAAGTTGCTTCTTTCAATGATTTAGGTTCTTTTATCTCTT     5639
HEINZ       AAGGATATCAGTGTAGTAGTGAAGTTGCTTCTTTCAATGATTTAGGTTCTTTTATCTCTT     5640
LA2093      AAGGATATCAGTGTAGTAGTGAAGTTGCTTCTTTCAATGATTTAGGTTCTTTTATCTCTT     5633
            ************************************************************

E42         AGTGCCGAGTGTTATTCTTTTGGCAGTCCAGACTGGTTTCTTCATTGCATTAGCTAGGTG     5699
HEINZ       AGTGCCGAGTGTTATTCTTTTGGCAGTCCAGACTGGTTTCTTCATTGCATTAGCTAGGTG     5700
LA2093      AGTGCCGAGTGTTATTCTTTTGGCAGTCCAGACTGGTTTCTTCATTGCATTAGCTAGGTG     5693
            ************************************************************

E42         AGGGGTAAGTGTTAAATGTCAAATGAATCATTAAGAATGATTATGCTATAATTATTTGGA     5759
HEINZ       AGGGGTAAGTGTTAAATGTCAAATGAATCATTAAGAATGATTATGCTATAATTATTTGGA     5760
LA2093      AGGGGTAAGTGTTAAATGTCAAATGAATCATTAAGAATGATTATGCTATAATTATTTGGA     5753
            ************************************************************

E42         CTTTGATGAATTAGGAAGCTAGTTTTGTTTCATGTAGCTCAAGGTCTTAAACCAAATTTT     5819
HEINZ       CTTTGATGAATTAGGAAGCTAGTTTTGTTTCATGTAGCTCAAGGTCTTGAACCAAATTTT     5820
LA2093      CTTTGATGAATTAGGAAGCTAGTTTTGTTTCATGTAGCTCAAGGTCTTAAACCAAATTTT     5813
            ************************************************ ***********

E42         TTTGCTGTTCTCTGTAGTATGTTCTTGCTTATTGTTAGAATAATAAAACTATGAGAAGCT     5879
HEINZ       TTTGCTGTTCTCTGTAGTATGTTCTTGCTTATTGTTAGAATAATAAAACTGTGAGAAGCT     5880
LA2093      TTTGCTGTTCTCTGTAGTATGTTCTTGCTTATTGTTAGAATAATAAAACTGTGAGAAGCT     5873
            ************************************************** *********

E42         GCCTCTTTCTTATTTATTGTCAGTATGGAATACTATCAAAAGCTAAATCATAGTTTGTTT     5939
HEINZ       GCCTCTTTCTTATTTATTGTCAGTATGGAATACTATCAAAAGCTAAATCATAGTTTGTTT     5940
LA2093      GCCTCGTTCTTATTTATTGTCAGTATGGAATACTATCAAAAGCTAAATCATAGTTTGTTT     5933
            ***** ******************************************************

E42         GTTTTCCTTTTAACTTTTTAGTGTATAGATAGCTGACTGGATTTTCTAGAAGCCTTATGC     5999
HEINZ       GTTTTCCTTTTAACTTTTTAGTGTATAGATAGCTGACTGGATTTTCTAGAAGCCTTATGC     6000
LA2093      GTTTTCCTTTTAACTTTTTAGTGTATAGATAGCTGACTGGATTTTCTAGAAGCCTTATGC     5993
            ************************************************************

E42         ATATTTTTATCAAAACACCAACTTGGTTATTTGTGTCTAGGGACTTTTTCAGTGGTATTC     6059
```

```
HEINZ       ATATTTTTATCAAAACACCAACTTGGTTATTTGTGTCTAGGGACTTTTTCAGTGGTATTC     6060
LA2093      ATATTTTTATCAAAACACCAACTTGGTTATTTGTGTCTAGGGACTTTTTCAGTGGTATTC     6053
            ************************************************************

E42         ACTCCTTTCTATTTCCTTCTTTGATGTTATTCTCGATTCCTTCTTTGATGTTATCCCCGA     6119
HEINZ       ACTCCTTTCTATTTCCTTCTTTGATGTTATTCTCGATTCCTTCTTTGATGTTATCCCCGA     6120
LA2093      ACTCCTTTCTATTTCCTTCTTTGATGTTATTCTCGATTCCTTCTTTGATGTTATCCCCGA     6113
            ************************************************************

E42         TTTCATCAGAAGTCTCATTTCAATCAATAATTCGGTGAACATCTTATCACTTAGAATTAG     6179
HEINZ       TTTCATCAGAAGTCTCATTTCAATCAATAATTCGGTGAACATCTTATCACTTAGAATTAG     6180
LA2093      TTTCATCAGAAGTCTCATTTCAATCAATAATTCGGTGAACATCTTATCACTTAGAATTAG     6173
            ************************************************************

E42         TCCATGTTTACTGAGACTGATGATGTCAGTTCATTTGGATGGACCTGTCAAGTCAGCACT     6239
HEINZ       TCCATGTTTACTGAGACTGATGATGTCAGTTCATTTGGATGGACCTGTCAAGTCAGCACT     6240
LA2093      TCCATGTTTACTGAGACTGATGATGTCAGTTCATTTGGATGGACCTGTCAAGTCAGCACT     6233
            ************************************************************

E42         TTGCATGTGTCTGGTCATGCATCTACATAAGCTAATACTGGCTTAGGCTCAGTAATTAGC     6299
HEINZ       TTGCATGTGTCTGGTCATGCATCTACATAAGCTAATACTGGCTTAGGCTCAGTAATTAGC     6300
LA2093      TTGCATGTGTCTGGTCATGCATCTACATAAGCTAATACTGGCTTAGGCTCAGTAATTAGC     6293
            ************************************************************

E42         AAATCATTCCTCTCTAATGAGTTGGGAAAGACATACCAATTTTGGTTGCTTGGTCAGAAC     6359
HEINZ       AAATCATTCCTCTCTAATGAGTTGGGAAAGACGTACCAATTTTGGTTGCTTGGTCAGAAC     6360
LA2093      AAATCATTCCTCTCTAATGAGTTGGGAAAGACATACCAATTTTGGTTGCTTGGTCAGAAC     6353
            ******************************** ***************************

E42         CTCCTAGAAGACCTTACTGAGTGGTTGTATTCATGAAGAAGATAGCTGTTGGTGTCTGAC     6419
HEINZ       CTCCTAGAAGACCTTACTGAGTGGTTGTATTCATGAAGAAGATAGCTGTTGGTGTCTGAC     6420
LA2093      CTCCTAGAAGACCTTACTGAGTGGTTGTATTCATGAAGAAGATAGCTGTTGGTGTCTGAC     6413
            ************************************************************

E42         CGATAATATGGCAGTTAACAGTAATGGTGTAAACATTATCCACAAGAGCGCAAAAGCAAA     6479
HEINZ       CGATAATATGGCAGTTAACAGTAATGGTGTAAACATTACCCACAAGAGCGCAAAAGCAAA     6480
LA2093      CGATAATATGGCAGTTAACAGTAATGGTGTAAACATTATCCACAAGAGCGCAAAAGCAAA     6473
            ************************************** *********************

E42         CCCTATTTCTTTACTCTCCAGCTGACAGTTAAAACATAATAGGCCCTCTTTGCATGTCCT     6539
HEINZ       CCCTATTTCTTTACTCTCCAGCTGACAGTTAAAACATAATAGGCCCTCTTTGCATGTCCT     6540
LA2093      CCCTATTTCTTTACTCTCCAGCTGACAGTTAAAACATAATAGGCCCTCTTTGCATGTCCT     6533
            ************************************************************

E42         CTAATTTAACACAGGCCAAAAGTGAACTCCTTCTGCAAAGGCTGACAAAATTTGCACCTA     6599
HEINZ       CTAATTTAACACAGGCCAAAAGTGAACTCCTTCTGCAAAGGCTGACAAAATTTGCACCTA     6600
LA2093      CTAATTTAACACAGGCCAAAAGTGAACTCCTTCTGCAAAGGCTGACAAAATTTGCACCTA     6593
            ************************************************************

E42         GCGTATTCCTTTATATATAGCTATATGGTTATAGAGGAGTCTTGCGTATTACCTGATGCA     6659
HEINZ       GCGTATTCCTTTATATATAGCTATATGGTTATAGAGGAGTCTTGCGTATTACCTGATGCA     6660
LA2093      GCGTATTCCTTTATATATAGCTATATGGTTATAGAGGAGTCTTGCGTATTACCTGATGCA     6653
            ************************************************************

E42         TCACCTTATCCTGGGGTCTACTAATGCCTTCCAACGGCTTTGCAAAAACTGCTAGAACAT     6719
HEINZ       TCACCTTATCCTGGGGTCTACTAATTCCTTCCAACGGCTTTGCAAAAACTGCTAGAACAT     6720
LA2093      TCACCTTATCCTGGGGTCTACTAATGCCTTCCAACGGCTTTGCAAAAACTGCTAGAACAT     6713
            ************************* **********************************

E42         AGAGTAGTGCATTGGAAAATGAAGGTTGGAAGGCTACCGATCAACTGGCAGAACTCCGCC     6779
HEINZ       AGAGTAGTGCATTGGAAAATGAAGGTTGGAAGGCTACCGATCAACTGGCAGAACTCCGCC     6780
LA2093      AGAGTAGTGCATTGGAAAATGAAGGTTGGAAGGCTACCGATCAACTGGCAGAACTCCGCC     6773
            ************************************************************

E42         ATGCAGCTAGCAGATGTGGTAAATATACAGATATGAGGAGGCAAATTACAACCACATTTA     6839
HEINZ       ATGCAGCTAGCAGATGTGGTAAATATACAGATATGAGGAGGCAAATTACAACCACATTTA     6840
LA2093      ATGCAGCTAGCAGATGTGGTAAATATACAGATATGAGGAGGCAAATTACAACCACATTTA     6833
            ************************************************************

E42         CCAATGAAATTTAAGTGGAAGTTGGAGGGAGAATCCAAGTCAAAGTTTTAAGGAATTTTG     6899
HEINZ       CCAATGAAATTTAAGTGGAAGTTGGAGGGAGAATCCAAGTCAAAGTTTTAAGGAATTTTG     6900
LA2093      CCAATGAAATTTAAGTGGAAGTTGGAGGGAGAATCCAAGTCAAAGTTTTAAGGAATTTTG     6893
```

```
            ************************************************************

E42         GGGGCCCACTTTTAACTCATTTTGGTGCATATACTACTGCATTCTTTGGAAACATTTCAC    6959
HEINZ       GGGGCCCACTTTTAACTCATTTTGGTGCTTATACTACTGCATTCTTTGGAAACATTTCAC    6960
LA2093      GGGGCCCACTTTTAACTCATTTTGGTGCCTATACTACTGCATTCTTTGGAAACATTTCAC    6953
            **************************** *******************************

E42         TTGGTATGTTGTTGTATATGACGCAGTTCCACGACTAGATACTAAACTTATAAGACGAAT    7019
HEINZ       TTGGTATGTTGTTGTATATGACGCAGTTCCACGACTAGATACTAAACATATAAGACGAAT    7020
LA2093      TTGGTATGTTGTTGTATATGACGCAGTTCCACGACTAGATACTAAACATATAAGACGAAT    7013
            *********************************************** ************

E42         TCATGTAAAAGTATGTGCTACCTCAGTTTCATTTTATATTACAATGTTTAACTGGGTTTG    7079
HEINZ       TCATGTAAAAGTATGTGCTACCTCAGTTTCATTTTATATTACAATGTTTAACTGGGTTTG    7080
LA2093      TCATGTAAAAGTATGTGCTACCTCAGTTTCATTTTATATTACAATGTTTAACTGGGTTTG    7073
            ************************************************************

E42         GAGTTGAGATTTTAGTTTGACTTATATAGCATATTAGTGATGATAAAAGAGAATAATGAA    7139
HEINZ       GAGTTGAGATTTTAGTTTGACTTATATAACATATTAGTGATGATAAAAGAGAATAATGAA    7140
LA2093      GAGTTGAGATTTTAGTTTGACTTATATAACATATTAGTGATGATAAAAGAGAATAATGAA    7133
            **************************** *******************************

E42         GTAGTGGGTGAATAGAGAGCAAATATCACATCAAAGTTGAAGTGTGTATATTTAATGATT    7199
HEINZ       GTAGTGGGTGAATAGAGAGCAAATATCACATCAAAGTTGAAGTGTGTATATTTAATGATT    7200
LA2093      GTAGTGGGTGAATAGAGAGCAAATATCACATCAAAGTTGAAGTGTGTATATTTAATGATT    7193
            ************************************************************

E42         AAAATTCTTGCTAGTCGTGAAAGTTGCAGTAAAATAGAATGGTGTCAAACGTTTTGTTAC    7259
HEINZ       AAAATTCTTGCTAGTCGTGAAAGTTGCAGTAAAATAGAATGGTGTCAAACGTTTTGTTAC    7260
LA2093      AAAATTCTTGCTAGTCGTGAAAGTTGCAGTAAAATAGAATGGTGTCAAACGTTTTGTTAC    7253
            ************************************************************

E42         GTCCATTACAAAGATGATAGTCCAAAAATCAAAGTTGTTTAGGACACGTTACTGTTCATT    7319
HEINZ       GTCCATTACAAAGATGATAGTCCAAAAATCAAAGTTGTTTAGGACACGTTACTGTTCATT    7320
LA2093      GTCCATTACAAAGATGATAGTCCAAAAATCAAAGTTGTTTAGGACACGTTACTGTTCATT    7313
            ************************************************************

E42         TATTATGGTTTGAGGCATATGTATGTGCCTGAGGCATGCATTTTGGCCAATTCTTT-TCT    7378
HEINZ       TATTATGGTTTGAGGCATATGTATGTGCCTGAGGCATGCATTTTGGCCAATTCTTTTGCT    7380
LA2093      TATTATGGTTTGAGGCATATGTATGTGCCTGAGGCATGCATTTTGGCCAATTCTTTTGCT    7373
            ********************************************************  **

E42         AGGGAATTCTGCATCCAAGCAGAAAGAAGGGTTTACAAAGAAGAAAAATAACTAATTTGC    7438
HEINZ       AGGGAATTCTGCATCCAAGCAGAAAGAAGGGTTTACAAAGAAGAAAAATAACTAATTTGC    7440
LA2093      AGGGAATTCTGCATCCAAGCAGAAAGAAGGGTTTACAAAGAAGAAAAATAACTAATTTGC    7433
            ************************************************************

E42         ATTCTTTTTTTGGCAACTCAAGGACCAGAGCATACACCTCAGCCCTATAAGAATATGCCT    7498
HEINZ       ATTCTTTTTTTGGCAACTCAAGGACCAGAGCATACACCTCAGCCCTATAAGAATATGCCT    7500
LA2093      ATTCTTTTTTTGGCAACTCAAGGACCAGAGCATACACCTCAGCCCTATAAGAATATGCCT    7493
            ************************************************************

E42         CATAGGATGCAGCTTACCAATGCCGTGGGCTCTTCTATTTGAAGTCACCATGGACTTCTA    7558
HEINZ       CATAGGATGCAGCTTACCAATGCCGTGGGCTCTTCTATTTGAAGTCACCATGGACTTCTA    7560
LA2093      CATAGGATGCAGCTTACCAATGCCGTGGGCTCTTCTATTTGAAGTCACCATGGACTTCTA    7553
            ************************************************************

E42         ACAAGGACATAGATTGGAACACTTCTAGTTTTATTTTCGACCTATTCACTCACTGCAATC    7618
HEINZ       ACAAGGACATAGATTGGAACACTTCTAGTTTTGTTTTCGACCTATTCACTCACTGCAATC    7620
LA2093      ACAAGGACATAGATTGGAACACTTCTAGTTTTGTTTTCGACCTATTCACTCACTGCAATC    7613
            ******************************** ***************************

E42         TAATAAGGAGATAATGGTACTGTTGTGGATGCATTTGTGGGCAAGACATATGTGCGACAG    7678
HEINZ       TAATAAGGAGATAATGGTACTGTTGTGGATGCATTTGTGGGCAAGACATATGTGCGACAG    7680
LA2093      TAATAAGGAGATAATGGTACTGTTGTGGATGCATTTGTGGGCAAGACATATGTGCGACAG    7673
            ************************************************************

E42         AGAGCCAAATAACTTGATTTAATTATAACGCTTGTAGTTCTCTTATTATTCTAGAATAGG    7738
HEINZ       AGAGCCAAATAACTTGATTTAATTATAACGCTTGTAGTTCTCTTATTATTCTAGAATAGG    7740
LA2093      AGAGCCAAATAACTTGATTTAATTATAACGCTTGTAGTTCTCTTATTATTCTAGAATAGG    7733
            ************************************************************
```

```
E42        AAGACTTTAATTATATTATTAGTAGTTCTCTTATCAGTTTAGAATAGGATTTATGTTTCT    7798
HEINZ      AAGACTTTAATTATATTAGTAGTAGTTCTCTTATTAGTTTAGAATAGGATTTATGTTTCT    7800
LA2093     AAGACTTTAATTATATTAGTAGTAGTTCTCTTATTAGTTTAGAATAGGATTTATGTTTCT    7793
           ***************** *************** **************************

E42        TAGTTTCATAAGACTTCCTATTACTGTAAGAAGACTCCTATTTAAAGGAGGATTTGAGGA    7858
HEINZ      TAGTTTCATAAGACTTCCTATTACTGTAAGAAGACTCCTATTTAAAGGAGGATTTGAGGA    7860
LA2093     TAGTTTCATAAGACTTCCTATTACTGTAAGAAGACTCCTATTTAAAGGAGGATTTGAGGA    7853
           ************************************************************

E42        ATAAGAAGTATCGCAGATTTTTATCAGAAAACAAGAGATAGGAGTTCTCTCTTTTAATGA    7918
HEINZ      ATAAGAAGTATCGCAGATTTTTATCAGAAAACAAGAGATAGGAGTTCTCTCTTTTAATGA    7920
LA2093     ATAAGAAGTATCGCAGATTTTTATCAGAAAACAAGAGATAGGAGTTCTCTCTTTTAATGA    7913
           ************************************************************

E42        ACTCTAAGGTTTCGGCTTCCCTTTGATGAGCTGAATTCATACACAAAAATAGGTCGATCT    7978
HEINZ      ACTCTAAGGTTTCGGCTTCCCTTTGATGAGCTGAATTCATACACAAAAATAGGTCGATCT    7980
LA2093     ACTCTAAGGTTTCGGCTTCCCTTTGATGAGCTGAATTCATACACAAAAATAGGTCGATCT    7973
           ************************************************************

E42        AGGAATTTGAACAAAAGCAAGGAAAAATCGAAGGCAGTTGAATTTGATTTCCCTGTTCAT    8038
HEINZ      AGGAATTCGAACAAAAGCAAGGAAAAATCGAAGGCAGTTGAATTTGATTTCCCTGTTCAT    8040
LA2093     AGGAATTCGAACAAAAGCAAGGAAAAATCGAAGGCAGTTGAATTTGATTTCCCTGTTCAT    8033
           ******* ****************************************************

E42        TGTCTTGTGACTCGATCCTTCTCTAAGAATCCTAACAGGTACCTTATGATGTGAATGGTG    8098
HEINZ      TGTCTTGTGACTCGATCCTTCTCTAAGAATCCTAACAGGTACCTTATGATGTGAATGGTG    8100
LA2093     TGTCTTGTGACTCGATCCTTCTCTAAGAATCCTAACAGGTACCTTATGATGTGAATGGTG    8093
           ************************************************************

E42        CAACCTATCAAGTGTGGCATGTATCATACTTACATGAAGGTGAAGATTTGTTTTACAGAC    8158
HEINZ      CAACCTATCAAGTGTGGCATGTATCATACTTACATGAAGGTGAAGATTTGTTTTACAGAC    8160
LA2093     CAACCTATCAAGTGTGGCATGTATCATACTTACATGAAGGTGAAGATTTGTTTCACAGAC    8153
           ***************************************************** ******

E42        TTTGGATAGCTTCCATTTATGTGATTAATCTCATATTGAATTTTACTTTCACCTGGGTAA    8218
HEINZ      TTTGGATAGCTTCCATTTATGTGATTAATCTCATATTGAATTTTACTTTCACCTGGGTAA    8220
LA2093     TTTGGATAGCTTCCATTTATGTGATTAATCTCATATTGAATTTTACTTTCACCTGGGTAA    8213
           ************************************************************

E42        ATCATGACATTGAAAATTATTTTCTAAAACATGGTTTTGCCTATGATGAGGTGGTCTAGG    8278
HEINZ      ATCATGACATTGAAAATTATTTTCTAAAACATGGTTTTGCCTATGATGAGGTGGTCTAGG    8280
LA2093     ATCATGACATTGAAAATTATTTTCTAAAACATGGTTTTGCCTATGATGAGGTGGTCTAGG    8273
           ************************************************************

E42        CTTTGCTGCTGTAAAAGGGTATTACTTCTTCTGTTAGTTACAGATACTTATTGTTCTTCT    8338
HEINZ      CTTTGCTGCTGTAAAAGGGTATTACTTCTTCTGTTAGTTACAGATACTTATTGTTCTTCT    8340
LA2093     CTTTGCTGCTGTAAAAGGGTATTACTTCTTCTGTTAGTTACAGATACTTATTGTTCTTCT    8333
           ************************************************************

E42        CTAGTTTTAGTTTTCTCTTACGTGTACTAGTTTTCATGTGGCCAAGAGGTCCATATTTAG    8398
HEINZ      CTAGTTTTAGTTTTCTCTTACGTGTACTAGTTTTCATGTGGCCAAGAGGTCCATATTTAG    8400
LA2093     CTAGTTTTAGTTTTCTCTTACGTGTACTAGTTTTCATGTGGCCAAGAGGTCCATATTTAG    8393
           ************************************************************

E42        AATGTCAGGTGTTTAATGTGGATCTGTTTAGTACTTTGGCAATGATACTGACATTGACAT    8458
HEINZ      AATGTCAGGTGTTTAATGTGGATCTGTTTAGTACTTTGGCAATGATACTGACATTGACAT    8460
LA2093     AATGTCAGGTGTTTAATGTGGATCTGTTTAGTACTTTGGCAATGATACTGACATTGACAT    8453
           ************************************************************

E42        GAAAATTGACATGTTTGATCCTTCCATTATGACAGGTCTAGGACGAAGCCTTACACGGAT    8518
HEINZ      GAAACTTGACATGTTTGATCCTTCCATTATGACAGGTCTAGGACGAAGCCTTACACGGAT    8520
LA2093     GAAACTTGACATGTTTGATCCTTCCATTATGACAGGTCTAGGACGAAGCCTTACACGGAT    8513
           **** *******************************************************

E42        GACGAGATAAAGGTACTTATCCTCTCTGGCATGTCCAAAAATTCTTCCAGTATTAAATGA    8578
HEINZ      GACGAGATAAAGGTACTTATCCTCTCTGGCATGTCCAAAAATTCTTCCAGTATTAAATGA    8580
LA2093     GACGAGATAAAGGTACTTATCCTCTCTGGCATGTCCAAAAATTCTTCCAGTATTAAATGA    8573
           ************************************************************

E42        AAATAGTGTTGTTGTGCAGTTTACCTCCCTTTTTTTTTCTTTTTATCTTTATACCTTCCA    8638
HEINZ      AAATAGTGTTGTTGTGCAGTTTACCTCCCTTTTTTTTTCTTTTTATCTTTATACCTTCCA    8640
```

```
LA2093       AAATAGTGTTGTTGTGCAGTTTACCTCCCTTTTTTTTTCTTTTTATCTTTATACCTTCCA     8633
             ************************************************************

E42          TTGTTCTAAGATTCCCTGATCCCACCTCTTCCTTGTGGAGGAGAATGATTTAGAGAACTA     8698
HEINZ        TTGTTCTAAGATTCCCTGATCCCACCTCTTCCTTGTGGAGGAGAATGATTTAGAGAACTA     8700
LA2093       TTGTTCTAAGATTCCCTGATCCCACCTCTTCCTTGTGGAGGAGAATGATTTAGAGAACTA     8693
             ************************************************************

E42          AATAGCGAAGAAAAGCTTTTTCTGAGTGATACTACCCCTGCCTCCCTGCATGACTTGAAG     8758
HEINZ        AATAGCGAAGAAAAGCTTTTTCTGAGTGATACTACCCCTGCCACCCTGCATGACTTGAAG     8760
LA2093       AATAGCGAAGAAAAGCTTTTTCTGAGTGATACTACCCCTGCCACCCTGCATGACTTGAAG     8753
             ****************************************** *****************

E42          CCTTGAAACATTTATTTCCGTTCTGAATTTTCTGAATAAAGAAATATTTTCCCTTTGGGT     8818
HEINZ        CCTTGAAACATTTATTTCCGTTCTGAATTTTCTGAATAAAGAAATATTTTCCCTTTGGGT     8820
LA2093       CCTTGAAACATTTATTTCCGTTCTGAATTTTCTGAATAAAGAAATATTTTCCCTTTGGGT     8813
             ************************************************************

E42          GATACCACCCTACTCTGAATGACAGAGTTAACTTATCTCCTTTGTGCTGCTCCTATTTTT     8878
HEINZ        GATACCACCCTACTCTGAATGACAGAGTTAACTTATCTCCTTTGTGCTGCTCCTATTTTT     8880
LA2093       GATACCACCCTACTCTGAATGACAGAGTTAACTTATCTCCTTTGTGCTGCTCCTATTTTT     8873
             ************************************************************

E42          CTGAATGAAACGTATGTGGGTTTATATTTGGCATTAGGCAGGAAAAGAGTGATCAAAATC     8938
HEINZ        CTGAATGAAACGTATGTGGGTTTATATTTGGCATTAGGCAGGAAAAGAGTGATCAAAATC     8940
LA2093       CTGAATGAAACGTATGTGGGTTTATATTTGGCATTAGGCAGGAAAAGAGTGATCAAAATC     8933
             ************************************************************

E42          ATCTGTGACCTCTTAAAGAAAACTACTGTCTTGATAAAGGTAAAAATAAAATGAAATGAT     8998
HEINZ        ATCTGTGACCTCTTAAAGAAAACTACTGTCTTGATAAAGGTAAAAATAAAATGAAATGAT     9000
LA2093       ATCTGTGACCTCTTAAAGAAAACTACTGTCTTGATAAAGGTAAAAATAAAATGAAATGAT     8993
             ************************************************************

E42          ACTCCCCGGATAAATTTTTTGTATCACCAACATTTTCGTAGCTAGAAGCTCTGTTCATTA     9058
HEINZ        ACTCCCCGGATAAATTTTTTGTATCACCAACATTTTCGTAGCTAGAAGCTCTGTTAATTA     9060
LA2093       ACTCCCCGGATAAATTTTTTGTATCACCAACATTTTCGTAGCTAGAAGCTCTGTTAATTA     9053
             ******************************************************* ****

E42          CTC---AACTTTACTTTTCTTTGTTCCTTGTTTACTTAGAGACTGCAAGCCGAGCTGCTT     9115
HEINZ        CTCAACAACTTTACTTTTCTTTGTTCCTTGTTTACTTAGAGACTGCAAGCCGAGCTGCTT     9120
LA2093       CTCAACAACTTTACTTTTCTTTGTTCCTTGTTTACTTAGAGACTGCAAGCCGAGCTGCTT     9113
             ***   ******************************************************

E42          CAATTCTGTTACCTGCCACCATGTCTCTTATACACAAAAAAACTTCGTGGAATATAAACA     9175
HEINZ        CAATTCTGTTACCTGCCACCATGTCTCTTATACACAAAAAAACTTCGTGGAATATAAACA     9180
LA2093       CAATTCTGTTACCTGCCACCATGTCTCTTATACACAAAAAAACTTCGTGGAATATAAACA     9173
             ************************************************************

E42          TATTTCCAACGGACTAAGATCCAGGCGTATTGCTTTGTTTTGCAAGATTATGTACTAGCC     9235
HEINZ        TATTTCCAATGGACTAAGATCCAGGCGTATTGCTTTGTTTTGCAAGATTATGTACTAGCC     9240
LA2093       TATTTCCAATGGACTAAGATCCAGGCGTATTGCTTTGTTTTGCAAGATTATGTACTAGCC     9233
             ********* **************************************************

E42          TTTTCTAGAATAGAACACTTTTGAGTAGCTTCAGGTCTGAGTTTACTGCCGTGCTACAAC     9295
HEINZ        TTTTCTAGAATAGAACACTTTTGAGTAGCTTCAGGTCTGAGTTTACTGCCGTGCTACAAC     9300
LA2093       TTTTCTAGAATAGAACACTTTTGAGTAGCTTCAGGTCTGAGTTTACTGCCGTGCTACAAC     9293
             ************************************************************

E42          AAAGACACTTGAGCAGTTCCCTACAAATTTCAGTTAAAAACATTACAGAACTAGCATGAA     9355
HEINZ        AAAGACACTTGAGCAGTTCCCTACAAATTTCAGTTAAAAACATTACAGAACTAGCATGAA     9360
LA2093       AAAGACACTTGAGCAGTTCCCTACAAATTTCAGTTAAAAACATTACAGAACTAGCATGAA     9353
             ************************************************************

E42          TTGTCTGCTCGGCAAAGTCGTGTTTGATAGAATCCAAAAAGGAGTGTCTTTTGGGATTGG     9415
HEINZ        TTGTCTGCTCGGCAAAGTCGTGTTTGATAGAATCCAAAAAGGAGTGTCTTTTGGGATTGG     9420
LA2093       TTGTCTGCTCGGCAAAGTCGTGTTTGATAGAATCCAAAAAGGAGTGTCTTTTGGGATTGG     9413
             ************************************************************

E42          GAAATGGATATCCACAAGGACTTTCATATGTTAAGTTGCTTCTCCTTTATTTCTAATCGA     9475
HEINZ        GAAATGGATATCCACAAGGACTTTCATATGTTAAGTTGCTTCTCCTTTATTTCTAATCGA     9480
LA2093       GAAATGGATATCCACAAGGACTTTCATATGTTAAGTTGCTTCTCCTTTATTTCTAATCGA     9473
             ************************************************************
```

```
E42         ATGTGCATCTTTCCTTTCCAATATTTGTTGGAGTGATCCTCACTAATTTAGGCTACCCGT     9535
HEINZ       ATGTGCATCTTTCCTTTCCAATATTTGTTGGAGTGATCCTCACTAATTTAGGCTACCCGT     9540
LA2093      ATGTGCATCTTTCCTTTCCAATATTTGTTGGAGTGATCCTCACTAATTTAGGCTACCCGT     9533
            ************************************************************

E42         TAAAACATATTTTTACTTCACAAAAAGTGATATTGGACATGTTTAATCTTTTCAGGAAAC     9595
HEINZ       TAAAACATATTTTTACTTCACAAAAAGTGATATTGGACATGTTTAATCTTTTCAGGAAAC     9600
LA2093      TAAAACATATTTTTACTTCACAAAAAGTGATATTGGACATGTTTAATCTTTTCAGGAAAC     9593
            ************************************************************

E42         TTTACTCCTTATTCGATTGATCAAGAACCTAAAAGTCCGCACATCCCGACTTGTCTCTTA     9655
HEINZ       TTTACTCCTTATTCAATTGATCAAGAACCTAAAAGTCCGCACATCCCGACTTGTCTCTTA     9660
LA2093      TTTACTCCTTATTCGATTGATCAAGAACCTAAAAGTCCGCACATCCCGACTTGTCTCTTA     9653
            ************** *********************************************

E42         GCAATATTTATACATTTTCAACTCTATGAAAAAAACACTAGGAAATAGTGAGCATAGTTG     9715
HEINZ       GCAATATTTATACATTTTCAACTCTATGAAAAAACCACTAGGAAATAGTGAGCATAGTTG     9720
LA2093      GCAATATTTATACATTTTCAACTCTATGAAAAAACCACTAGGAAATAGTGAGCATAGTTG     9713
            ********************************** *************************

E42         GTAGGAAATTGAAATGAACAACTGACTAAGAGCCTGTTTGGATTGACTTATTTTAAGTGC     9775
HEINZ       GTAGGAAATTGAAATGAACAACTGACTAAGAGCCTGTTTGGATTGACTTATTTTAAGTGC     9780
LA2093      GTAGGAAATTGAAATGAACAACTGACTAAGAGCCTGTTTGGATTGACTTATTTTAAGTGC     9773
            ************************************************************

E42         TTTTAAGTCAATTTAGCTTTTAAATTTTTTTGTAGTGTTTGGATATAATTAATAAGTGCT     9835
HEINZ       TTTTAAGTCAATTTAGCTTTTAAATTTTTTTGTAGTGTTTGGATATAATTAATAAGTGCT     9840
LA2093      TTTTAAGTCAATTTAGCTTTTAAATTTTTTTGTAGTGTTTTGATATAATTAATAAGTGCT     9833
            **************************************** *******************

E42         TTTAAGCACTTGTCCTTAAGGGGGAGGGATAAAAATAAGTCAAAAGCCATGTGTTAGAAT     9895
HEINZ       TTTAAGCACTTGTCCTTAAGGGGGAGGGATAAAAATAAGTCAAAAGCCATGTGTTAGAAT     9900
LA2093      TTTAAGCACTTGTCCTTAAGGGGGAGGGATAAAAATAAGTCAAAAGCCATGTGTTAGAAT     9893
            ************************************************************

E42         TTTTAACTTACGGGTTTTGGCTTATAAGCTAAAAGCTATAAGCCCATTCAAGCAGGCTCT     9955
HEINZ       TTTTAACTTACGGGTTTTGGCTTATAAGCTAAAAGCTATAAGCCCATTCAAGCAGGCTCT     9960
LA2093      TTTTAACTTACGGGTTTTGGCTTATAAGCTAAAAGCTATAAGCCCATTCAAGCAGGCTCT     9953
            ************************************************************

E42         AATACTATTTCTTAACTGCCTCGTCAAATTTAAGAAGTTGATGCCTAATATTATTGGGTG     10015
HEINZ       AATACTATTTCTTAACTGCCTCGTCAAATTTAAGAAGTTGATGCCTAATATTATTGGGTG     10020
LA2093      AATACTATTTCTTAACTGCCTCGTCAAATTTAAGAAGTTGATGCCTAATATTATTGGGTG     10013
            ************************************************************

E42         GTTCTGGCATTGCCTATTACAAGGAGATTTTATGAAGCTGCTTATTTATTGATCATACTT     10075
HEINZ       GTTCTGGCATTGCCTATTACAAGGAGATTTTATGAAGCTGCTTATTTATTGATCATACTT     10080
LA2093      GTTCTGGCATTGCCTATTACAAGGAGATTTTATGAAGCTGCTTATTTATTGATCATACTT     10073
            ************************************************************

E42         GTACACACTGATCTCTGCTAAAATTATCTTAATATGATAAATCCTCCAACTTGAGCTATT     10135
HEINZ       GTACACACTGATCTCTGCTAAAATTATCTTAATATGATAAATCCTCCAACTTGAGCTATT     10140
LA2093      GTACACACTGATCTCTGCTAAAATTATCTTAATATGATAAATCCTCCAACTTGAGCTATT     10133
            ************************************************************

E42         TGCTCGGGCGTCTGTAGTTGACATCGCAATGGTCCTTCAGTTTCGACTATCTGTTCTGCT     10195
HEINZ       TGCTCGGGCGTCTGTAGTTGACATCGCAATGGTCCTTCAGTTTCGACTATCTGTTCTGCT     10200
LA2093      TGCTCGGGCGTCTGTAGTTGACATCGCAATGGTCCTTCAGTTTCGACTATCTGTTCTGCT     10193
            ************************************************************

E42         TCTTTCCCTGTCTTTCATGGGCACATGTGAAAACATTAACTTGTATTGAGAAGTTTCTTT     10255
HEINZ       TCTTTCCCTGTCTTTCATGGGCACATGTGAAAACATTAACTTGTATTGAGAAGTTTCTTT     10260
LA2093      TCTTTCCCTGTCTTTCATGGGCACATGTGAAAACATTAACTTGTATTGAGAAGTTTCTTT     10253
            ************************************************************

E42         TTTTGAACTTGCAGTCAGCATTTAGGGAAAAGGCAAAGCAGTTTCACCCTGATCAGAATC     10315
HEINZ       TTTTGAACTTGCAGTCAGCATTTAGGGAAAAGGCAAAGCAGTTTCACCCTGATCAGAATC     10320
LA2093      TTTTGAACTTGCAGTCAGCATTTAGGGAAAAGGCAAAGCAGTTTCACCCTGATCAGAATC     10313
            ************************************************************

E42         AGCAGAACAAAGGTGGATTGTGTTAGTCTGGTCTATTTGATATTTTATCCTTGTATTACC     10375
```

```
HEINZ     AGCAAAACAAAGGTGGATTGTGTTAGTCTGGTCTATTTGATATTTTATCCTTGTATTACC    10380
LA2093    AGCAAAACAAAGGTGGATTGTGTTAGTCTGGTCTATTTGATATTTTATCCTTGTATTACC    10373
          **** *******************************************************

E42       TATTTCAAATTCAAGAAAAACAAAAGGAAAGAAATGCTACTACCTGCATCATGTGATAAA    10435
HEINZ     TATTTCAAATTCAAGAAAAACAAAAGGAAAGAAATGCTACTACCTGCATCATGTGATAAA    10440
LA2093    TATTTCAAATTCAAGAAAAACAAAAGGAAAGAAATGCTACTACCTGCATCATGTGATAAA    10433
          ************************************************************

E42       TATCTGATATTGTAACATGGTTGCAGATACTGCTGAAGCGAAGTTTAAGGAAGTCATGAA    10495
HEINZ     TATCTGATATTGTAACATGGTTGCAGATACTGCTGAAGCGAAGTTTAAGGAAGTCATGAA    10500
LA2093    TATCTGATATTGTAACATGGTTGCAGATACTGCTGAAGCGAAGTTTAAGGAAGTCATGAA    10493
          ************************************************************

E42       GTCATATGAGGCTATAAAGTCGGAAAGGAAGAATGGCACGAGCAGATAAAGAGCCATATG    10555
HEINZ     GTCATATGAGGCTATAAAGTCGGAAAGGAAGAATGGCACGAGCAGATAAAGAGCCATATG    10560
LA2093    GTCATATGAGGCTATAAAGTCGGAAAGGAAGAATGGCACGAGCAGATAAAGAGCCATATG    10553
          ************************************************************

E42       ATAAATGCCTGAAATTTTCTGTAACTTCTAAAAGATCATGTCCACTTGATGTTGTATATT    10615
HEINZ     ATAAATGCCTGAAATTTTCTGTAACTTCTAAAAGATCATGTCCACTTGATGTTGTATATT    10620
LA2093    ATAAATGCCTGAAATTTTCTGTAACTTCTAAAAGATCATGTCCACTTGATGTTGTATATT    10613
          ************************************************************

E42       AGTCTAGCTAATCCAAGTGGTCCTTCATGATGGATTCCTGGGAGACTATCGAGCCATTTG    10675
HEINZ     AGTCTAGCTAATCCAAGTGGTCCTTCATGATGGATTCCTGGGAGACTATCGAGCCATTTG    10680
LA2093    AGTCTAGCTAATCCAAGTGGTCCTTCATGATGGATTCCTGGGAGACTATCGAGCCATTTG    10673
          ************************************************************

E42       CTTCCAGCCTTGTGCTAGTTGACTGGTACACGTAGTCTCTTGACATTCTTGGCTGCTTAT    10735
HEINZ     CTTCCAGCCTTGTGCTAGTTGACTGGTACACGTAGTCTCTTGACATTCTTGGCTGCTTAT    10740
LA2093    CTTCCAGCCTTGTGCTAGTTGACTGGTACACGTAGTCTCTTGACATTCTTGGCTGCTTAT    10733
          ************************************************************

E42       CAAGGTTTTCAGGATACGCTTTGCAATAGGTTCTTGTTTCTTTTTCTTCAATTGACGAGT    10795
HEINZ     CAAGGTTTTCAGGATACGCTTTGCAATAGGTTCTTGTTTCTTTTTCTTCAATTGACGAGT    10800
LA2093    CAAGGTTTTCAGGATACGCTTTGCAATAGGTTCTTGTTTCTTTTTCTTCAATTGACGAGT    10793
          ************************************************************

E42       TTTAAGAGTACTTCTACAGCTAGGATTGTTGCTTCTATAATCCCTCCATGGACTTCCAAC    10855
HEINZ     TTTAAGAGTACTTCTACAGCTAGGATTGTTGCTTCTATAATCCCTCCATGGACTTCCAAC    10860
LA2093    TTTAAGAGTACTTCTACAGCTAGGATTGTTGCTTCTATAATCCCTCCATGGACTTCCAAC    10853
          ************************************************************

E42       TTCCTGGAAGAGCGCAATTTGTGTCGTGGGATAAGCTGACATACGGATTTACATTGTATA    10915
HEINZ     TTCCTGGAAGAGCGCAATTTGTGTCGTGGGATAAGCTGACATACGGATTTACATTGTATA    10920
LA2093    TTCCTGGAAGAGCGCAATTTGTGTCGTGGGATAAGCTGACATACGGATTTACATTGTATA    10913
          ************************************************************

E42       TCACACAGGCTTTAGTACACTGTAAGTTAGCTCAAGATGTCAAATAAAGAGAGAAAAGGC    10975
HEINZ     TCACACAGGCTTTAGTACACTGTAAGTTAGCTCAAGATGTCAAATAAAGAGAGAAAAGGC    10980
LA2093    TCACACAGGCTTTAGTACACTGTAAGTTAGCTCAAGATGTCAAATAAAGAGAGAAAAGGC    10973
          ************************************************************

E42       TGTAATCAGCCTATGAATTTTGCCTTGTATTAAATTTGAGTGGCCAAGTTTGAATGGCCC    11035
HEINZ     TGTAATCAGCCTATGAATTTTGCCTTGTATTAAATTTG--------------AATGGCCC    11026
LA2093    TGTAATCAGCCTATGAATTTTGCCTTGTATTAAATTTG--------------AATGGCCC    11019
          **************************************              ********

E42       ATGCAAGTGGTTGATTATGTTATATTCTTGCTTGGTAGAAAAGATATGTAAGAAACTTCT    11095
HEINZ     ATGCAAGTGGTTGATTATGTTATATTCTTGCTTGGTAGAAAAGATATGTAAGAAACTTCT    11086
LA2093    ATGCAAGTGGTTGATTATGTTATATTCTTGCTTGGTAGAAAAGATATGTAAGAAACTTCT    11079
          ************************************************************

E42       TGAGATTGTTCATTGTTGCATCACAAAAGGTCGAAATTCAGAGAAAAAAACAAATTATAT    11155
HEINZ     TGAGATTGTTCATTGTTGCATCACAAAAGGTCGAAATTCAGAGAAAAAAACAAATTATAT    11146
LA2093    TGAGATTGTTCATTGTTGCATCACAAAAGGTCGAAATTCAGAGAAAAAAACAAATTATAT    11139
          ************************************************************

E42       GCTATTATTTTGTCAAGATATATGCCATAGCACTCACTTTTACATTACATTAATTTCAAT    11215
HEINZ     GCTATTATTTTGTCAAGATATATGCCATAGCACTCACTTTTACATTACATTAATTTCAAT    11206
LA2093    GCTATTATTTTGTCAAGATATATGCCATAGCACTCACTTTTACATTACATTAATTTCAAT    11199
```

```
            ************************************************************

E42         CTCAAGCTTCGAGCCTTTAAAGGATCAGTTGTTATGCTTCCTCAATTTCTACTCCATTGT    11275
HEINZ       CTCAAGCTTCGAGCCTTTAAAGGATCAGTTGTTATGCTTCCTCAATTTCTACTCCATTGT    11266
LA2093      CTCAAGCTTCGAGCCTTTAAAGGATCAGTTGTTATGCTTCCTAAATTTCTACTCCATTGT    11259
            ****************************************** *****************

E42         CGTCAAACTCAAATCTTCGGTATCAGTAATTATTGCCAAATGTGGGAGAACACAAGAAGA    11335
HEINZ       CGTCAAACTCAAATCTTCGGTATCAGTAATTATTGCCAAATGTGGGAGAACACAAGAAGA    11326
LA2093      CGTCAAACTCAAATCTTCGGTATCAGTAATTATTGCCAAATGTGGGAGAACACAAGAAGA    11319
            ************************************************************

E42         AAAGGTAAAAAGGAGAAAAAAATGAATGTACAGAGAAGAGATAAGGTATACTTTTACAAC    11395
HEINZ       AAAGGTAAAAAGGAGAAAAAAATGAATGTACAGAGAAGAGATAAGGTATACTTTTACAAC    11386
LA2093      AAAGGTAAAAAGGAGAAAAAAATGAATGTACAGAGAAGAGATAAGGTATACTTTTACAAC    11379
            ************************************************************

E42         TTATATTTACTTTGCAGTTAATAGTGTAATCAATCATGGGAACAAAGGAAAAAGATTACA    11455
HEINZ       TTATATTTACTTTGCAGTTAATAGTGTAATCAATCATGGGAACAAAGGAAAAAGATTACA    11446
LA2093      TTATATTTACTTTGCAGTTAATAGTGTAATCAATCATGGGAACAAAGGAAAAAGATTACA    11439
            ************************************************************

E42         AGGGAAGACTAGAAACCAAAAATTGTGCAACAGTTGGACTAAACTCTTGAAACTCACAAC    11515
HEINZ       AGGGAAGACTAGAAACCAAAAATTGTGCAACAGTTGGACTAAACTCTTGAAACTCACAAC    11506
LA2093      AGGGAAGACTAGAAACCAAAAATTGTGCAACAGTTGGACTAAACTCTTGAAACTCACAAC    11499
            ************************************************************

E42         CAGAATACTGATGGAGGTGGTA    11537
HEINZ       CAGAATACTGATGGAGGTGGTA    11528
LA2093      CAGAATACTGATGGAGGTGGTA    11521
            **********************
```

Solyc05g050820

```
E42         CCCAGGCATGATTCAACAGATGCAACATGTTTGTTACGAGTGCCAAGGCTCAGGTAATGA    60
HEINZ       CGAAGGCATGATTCAACAGATGCAACATGTTTGTTACGAGTGCCAAGGCTCAGCTAATGA    60
LA2093      CCCAGGCATGATTCAACAGATGCAACATGTTTGTTACGAGTGCCAAGGCTCAGGTAATGA    60
            *  ************************************************** ******

E42         GATTTCCCGGTAATTCTAATTTTCAGTCTTTACATGTGGAGTGAAAAACACTGCTTGTAA    120
HEINZ       GATTTCCCGGTAATTCTAATTTTCAGTCTTTACATGTGGAGTGAAAAACACTGCTTGTAA    120
LA2093      GATTTCCCGGTAATTCTAATTTTCAGTCTTTACATGTGGAGTGAAAAACACTGCTTGTAA    120
            ************************************************************

E42         CTATGAATTTTTCCTTGTTAATACAGGAGAGGTTATAACTGAGTTGGATAGGTGTCCTCA    180
HEINZ       CTATGAATTTTTCCTTGTTAATACAGGAGAGGTTATAACTGAGTTGGATAGGTGTCCTCA    180
LA2093      CTATGAATTTTTCCTTGTTAATACAGGAGAGGTTATAACTGAGTTGGATAGGTGTCCTCA    180
            ************************************************************

E42         GTGCAATGGAAATAAAATTACACAAGAAAAGAAAGTATTGAATGTGAATGTTGAGAAAGG    240
HEINZ       GTGCAATGGAAATAAAATTACACAAGAAAAGAAAGTATTGAATGTGAATGTTGAGAAAGG    240
LA2093      GTGCAATGGAAATAAAATTACACAAGAAAAGAAAGTATTGAATGTGAATGTTGAGAAAGG    240
            ************************************************************

E42         GATGTTACACGGTCAGAAGATTGTTTTCAACGCGGAAGCTGATGAAGCTGTAAAGAGTTT    300
HEINZ       GATGTTACACGGTCAGAAGATTGTTTTCAACGCGGAAGCTGATGAAGCTGTAA-GAGTTT    299
LA2093      GATGTTACACGGTCAGAAGATTGTTTTCAACGCGGAAGCTGATGAAGCTGTAA-GAGTTT    299
            ***************************************************** ******

E42         TTTCGCTATGCAAATTAGCTCAGCTTTTTGTAATTCCCCAGTGTACATTTTAAATTGTGC    360
HEINZ       TTTCGCTATGCAAATTAGCTCAGCTTTTTGTAATTCCCCAGTGTACATTTTAAATTGTGC    359
LA2093      TTTCGCTATGCAAATTAGCTCAGCTTTTTGTAATTCCCCAGTGTACATTTTAAATTGTGC    359
            ************************************************************

E42         CTCAAAAACTTGATCTTTCTTCTCAAGTCTAAATTTTGAAGTCAGTGCAGGACTTTGAAT    420
HEINZ       CTCAAAAACTTGATCTTTCTTCTTAAGTCTAAATTTTGAAGTCAGT----------GAAT    409
LA2093      CTCAAAAACTTGGTCTTTCTTCTTAAGTCTAAATTTTGAAGTCAGT----------GAAT    409
            ************ ********** **********************          ****

E42         TGACTTCCTCTTACATTTTGGCAAGTGATATTTCGTTTCTCATAGTTGTTTTGTGGTGTT    480
HEINZ       TGACTTCCTCTTACATTTTGGCAAGTGATATTTCGTTTCTCATAGTTGTTTTGTGGTGTT    469
LA2093      TGACTTCCTCTTACATTTTGGCAAGTGATATTTCGTTTCTCATAGTTGTTTTGTGGTGTT    469
            ************************************************************

E42         ATATGCAGCCAGATACCATCACCGGCAATGTTATTTTTGTATTACAACAGAAGAGTCACT    540
HEINZ       ATATGCAGCCAGATACCATCACCGGCAATGTTATTTTTGTATTACAACAGAAGAGTCACT    529
LA2093      ATCTGCAGCCAGATACCATCACCGGCAATGTTATTTTTGTATTACAACAGAAGAGTCACT    529
            ** *********************************************************

E42         CAAAGTTCAGGCGCAACTCTGATGATCTTTACATGGAACACAATATCAGTTTGACAGAAG    600
HEINZ       CAAAGTTCAGGCGCAACTCTGATGATCTTTACATGGAACACAATATCAGTTTGACAGAAG    589
LA2093      CAAAGTTCAGGCGCAACTCTGATGATCTTTACATGGAACACAATATCAGTTTGACAGAAG    589
            ************************************************************

E42         CTCTCTGTGGCTTTCAGTTTGTCCCGACTCATCTTGACGGCAGGCAGCTTCAGATCAAAT    660
HEINZ       CTCTCTGTGGCTTTCAGTTTGTCCTGACTCATCTTGACGGCAGGCAGCTTCAGATCAAAT    649
LA2093      CTCTCTGTGGCTTTCAGTTTGTCCTGACTCATCTTGACGGCAGGCAGCTTCAGATCAAAT    649
            ************************ ***********************************

E42         CTAGCCCCGGAGAAGTTATAAAGCCTGGTAATTAGTGATGGTTGTTAGTTTGTGCTTTAG    720
HEINZ       CTAGCCCCGGAGAAGTTATAAAGCCTGGTAATTAGTGATGGTTGTTAGTTTGTGCTTTAG    709
LA2093      CTAGCCCCGGGGAAGTTATAAAGCCTGGTAATTAGTGATGGTTGTTAGTTTGTGCTTTAG    709
            ********** *************************************************

E42         TTACCTTGTGGCGTGAAATGTGATATTTATATATGGGGGGATATTTCGTTCTCTCTTGCA    780
HEINZ       TTACCTTGTGGCGTGAAATGTGATATTTATATATGGGGGGATATTTCGTTCTCTCTTGCA    769
LA2093      TTACCTTGTGGCGTGAAATGTGACATTTATATATGGGGGGATATTTCGTTCTCTCTTGCA    769
            *********************** ************************************

E42         GATCAATACAAGGCGATAAATGATGAAGGAATGCCCCGTTATGGTAGGCCATTCATTAAG    840
HEINZ       GATCAATACAAGGCGATAAATGATGAAGGAATGCCCCGTTATGGTAGGCCATTCATTAAG    829
LA2093      GATCAATACAAGGCGATAAATGATGAAGGAATGCCCCGTTATGGTAGGCCGTTCATTAAG    829
            ************************************************** *********
```

```
E42         GGTCAGCTTTACATCCATTTTAATGTGATATTTCCAGAATCTGGATTTCTTTCTCTCGAT    900
HEINZ       GGTCAGCTTTACATCCATTTTAATGTGATATTTCCAGAATCTGGATTTCTTTCTCTCGAT    889
LA2093      GGTCAGCTTTACATCCATTTTAATGTGATATTTCCAGAATCTGGATTTCTTTCTCTCGAT    889
            ************************************************************

E42         AAATGTCGTGCTCTTGAGGCTATTCTGCCAACGAGACTAGGGAAACGCTCATCAGGTATA    960
HEINZ       AAATGTCGTGCTCTTGAGGCTATTCTGCCAACGAGACTAGGGAAACGCTCATCAGGTATA    949
LA2093      AAATGTCGTGCTCTTGAGGCTATTCTGCCAACGAGACTAGGGAAACGCTCATCAGGTATA    949
            ************************************************************

E42         GAGTTGGAGAAGTGTGAGGAAACAACTATGCACGACGTCAACATTGATGAAGCAATGAGA    1020
HEINZ       GAGTTGGAGAAGTGTGAGGAAACAACTATGCACGACGTCAACATTGATGAAGAAATGAGA    1009
LA2093      GAGTTGGAGAAGTGTGAGGAAACAACTATGCACGACGTCAACATTGATGAAGC-------    1002
            ****************************************************

E42         CGCAATGAGCAGCGTCATCACAGGCAAGAGGCTTATGATACAGATGATGAAATCAATGCA    1080
HEINZ       CGCAATGAGCAGCGTCATCACAGGCAAGAGGCTTATGATACAGATGATGAAATCAATGCA    1069
LA2093      ---AATGAGCAGCGTCATCACAGGCAAGAGGCTTATGATACAGATGATGAAATCAATGCA    1059
               *********************************************************

E42         AATCTGCATAGCTTGGGTTGTAACCAACAATAGAAGTGAAGTTCCCAGATAGTCTGGTCT    1140
HEINZ       AATCTGCATAGCTTGGGTTGTAACCAACAATAGAGTTGAAGTTCCCAGATAGTGTGGTCT    1129
LA2093      AATCTGCATAGCTTGGGTTGTAACCAACAATAGAATTGAAGTTCTCAGATAGTGTGGTCT    1119
            **********************************  ******** ******** ******

E42         TTTAGTTGATTCCCCAATTTTGTCTAATGCTCTGTTGGCTGTTTTGATTAGTAACTAACT    1200
HEINZ       TTTAGTTGATTCCCCAATTTTGTCTAATGCTCTGTTGGCTGTTTTGATTAGTAACTAACT    1189
LA2093      TTTAGTTGATTCCCCAATTTTGTCTAATGCTCTGTTGGCTGTTTTGATTAGTAACTAACT    1179
            ************************************************************

E42         GATTTTCTTTTTTGTTCTGCTTTTCAGTATAATGAAATGCATATATTTGGATGGTTGGTA    1260
HEINZ       GATTTTCTTTTTTGTTCTGCTTTTCAGTATAATGAAATGCATATATTTGGATGGTTGGTA    1249
LA2093      GATTTTCTTTTTTGTTCTGCTTTTCAGTATAATGAAATGCATATATTTGGATGGTTGGTA    1239
            ************************************************************

E42         AGTATC    1266
HEINZ       AGTATC    1255
LA2093      AGTATC    1245
            ******
```

Solyc05g051140

```
HEINZ       CCATCTCCTACTATGAGTAGAAGACGAGGTAGATCTGACAGTAAATAGTCTTTAGATTAG    60
E42         CCATCTCCTACTATGAGTAGAAGACGAGGTAGATCTGACAGTAAATAGTCTTTAGATTAG    60
LA2093      CCATCTCCTACTATGAGTAGAAGACGAGGTAGATCTGACAGTAAATAGTCTTTAGATTAG    60
            ************************************************************

HEINZ       GTATCCAATGGTTTTCTCGGTAATGTCAACGAAGAACGATCAACATTGGTTTCTTCATCT    120
E42         GTATCCAATGGTTTTCTCGGTAATGTCAACGAAGAACGATCAACATTGGTTTCTTCATCT    120
LA2093      GTATCCAATGGTTTTCTCGGTAATGTCAACGAAGAACGATCAACATTGGTTTCTTCATCT    120
            ************************************************************

HEINZ       TGTTCAGGTAAAGTTAGCAAACTCAAGTGAAAAGTTCTGATTTTTCTAATAGAAAGTTGA    180
E42         TGTTCAGGTAAAGTTAGCAGACTCAAGTGAAAAGTTCTGATTTTTCTAATAGAAAGTTGA    180
LA2093      TGTTCAGGTAAAGTTAGCAGACTCAAGTGAAAAGTTCTGATTTTTCTAATAGAAAGTTGA    180
            ******************* ****************************************

HEINZ       TTCTGAAGTGGTTAGAAGTAAAATTCGAGAAGAGGAGTTATTCCAGATGAGATTATTTAT    240
E42         TTCTGAAGTGGTTAGAAGTAAAATTCGAGAAGAGGAGATATTCCAGATGAGATTATTTAT    240
LA2093      TTCTGAAGTGGTTAGAAGTAAAATTCGAGAAGAGGAGTTATTCCAGATGAGATTATTTAT    240
            ************************************* **********************

HEINZ       GTATAAAAAGAGTCAATTTCTGAGGCTTTAGTTGCATTGATGGTTGAATTTTCGTCTAAC    300
E42         GTATAAAAAGAGTCAATTTCTGAGGCTTTAGTTGCATTGATGGTTGAATTTTCGTCTAAC    300
LA2093      GTATAAAAAGAGTCAATTTCTGAGGCTTTAGTTGCATTGATGGTTGAATTTTCGTCTAAC    300
            ************************************************************

HEINZ       TCGTTAAAGGATGAAAAAATTGAATCTAGGGTTGTTGGTGTGGTTGGCAGGATATACCAA    360
E42         TCGTTAAAGGATGAAAAAATTGAATCTAGAGTTGTTGGTGTAGTTGGCAGGATATACCAA    360
LA2093      TCGTTAAAGGATGAAAAAATTGAATCTAGAGTTGTTGGTGTAGTTGGCAGGATATACCAA    360
            ***************************** *********** ******************

HEINZ       TGTCACTATATTTTAGTTAGAACACATTATTACAAAATGGCGTGAAAATGTTTCGATTTG    420
E42         TGTCACTATATTTTAGTTAGAACACATTATTACAAAATGGTGTGAAAATGTTTCGATTTG    420
LA2093      TGTCACTATATTTTAGTTAGAACACATTATTACAAAATGGTGTGAAAATGTTTCGATTTG    420
            **************************************** *******************

HEINZ       GGTGACAAACTATATGTTTGGTAATGTTATACATGAATGGTGTAGTGAGTTATTAAATTC    480
E42         GGTGACAAACTATATGTTTGGTAATGTTATACATGAATGGTGTAGTGAGTTATTAAATTC    480
LA2093      GGTGACAAACTATATGTTTGGTAATGTTATACATGAATGGTGTAGTGAGTTATTAAATTC    480
            ************************************************************

HEINZ       TGCCTATAACTATTTGTTATCATACATATATAGTAATTTTGTGTTGGCATTGACAATTAA    540
E42         TGCCTATAACTATTTGTTATCATACATATATAGTAATTTTGTGTTGGCATTGACAATTAA    540
LA2093      TGCCTATAACTATTTGTTATCATACATATATAGTAATTTTGTGTTGGCATTGACAATTAA    540
            ************************************************************

HEINZ       GAACATGATTCCAGCTGCGCCAAGTAAAGACATAGTGATTGTTATTGGGGTAAGTCTTTC    600
E42         GAACATGATTCTAGCTGCGCCAAGTAAAGACATAGTGATTGTTATTGGGGTAAGTCTTTC    600
LA2093      GAACATGATTCTAGCTGCGCCAAGTAAAGACATAGTGATTGTTATTGGGGTAAGTCTTTC    600
            *********** ************************************************

HEINZ       TTGGTTGGCGACTGTATGATAATTGATGTTATTTGGGTTTGAGGTTATTGTTTTGGAGGG    660
E42         TTGGTTGGCGACTGTATGATAATTGATGTTATTTGGGTTTGAGGTTATTGTTTTGGAGGG    660
LA2093      TTGGTTGGCGACTGTATGATAATTGATGTTATTTGGGTTTGAGGTTATTGTTTTGGAGGG    660
            ************************************************************

HEINZ       ACGAAAGCGTGCAGGTGGTAGGTGTATACAAAAAATATGGAATGAGGAAATAAGGTGGTA    720
E42         ACGAAAGCGTGCAGGTGGTAGGTGTATACAAAAAATATGGAATGAGGAAATAAGGTGGTA    720
LA2093      ACGAAAGCGTGCAGGTGGTAGGTGTATACAAAAAATATGGAATGAGGAAATAAGGTGGTA    720
            ************************************************************

HEINZ       GTTGTTGATCTAAGAGGGAGTGTTTTGACAGGTACACTAGGAAATCTACTTTGTTTGTTG    780
E42         GTTGTTGATCTAAGAGGGAGTGTTTTGACAGGTACACTAGGAAATCTACTTTGTTTGTTG    780
LA2093      GTTGTTGATCTAAGAGGGAGTGTTTTGACAGGTACACTAGGAAATCTACTTTGTTTGTTG    780
            ************************************************************

HEINZ       GCTTAATGGTTGTCGTATACACTTCATAAGGTGAGAGATCAATGTCCATTCAGTCGTGCT    840
E42         GCTTAATGGTTGTCGTATACACTTCATAAGGTGAGAGATCAATATCCATTCAGTCGTGCT    840
LA2093      GCTTAATGGTTGTCGTATACACTTCATAAGGTGAGAGATCAATATCCATTCAGTCGTGCT    840
            ******************************************* ****************
```

```
HEINZ       GATGGAAAGCCCGTAGATAAGTATTTGGATAAAAAAGGTGAAGGCTGCTTACTATGA       897
E42         GATGGAAAGCCCGTAGATAAGTATTTGGATAAAAAAGGTGAAGGCTGCTTACTATGA       897
LA2093      GATGGAAAGCCCGTAGATAAGTATTTGGATAAAAAAGGTGAAGGCTGCTTACTATGA       897
            *********************************************************
```

Solyc05g053850

```
LA2093       CATCTCATGTAATAAACAAAAATTGAGCTTATTAATTATAATTGAGAAGAAAAAAAATCA   60
E42          CATCTCATGTAATAAACAAAAATTGAGCTTATTAATTATAATTGAGAAGAAAAAAAATCA   60
HEINZ        CATCTCATGTAATAAACAAAAATTGAGCTTATTAATTATAATTGAGAAGAAAAAAAATCA   60
             ************************************************************

LA2093       TGCCTAGAGATCCTTTAATAGTTTCTGGAGTTGTTGGAGATGTTGTTGATCCATTCACAA   120
E42          TGCCTAGAGATCCTTTAATAGTTTCTGGAGTTGTTGGAGATGTTGTTGATCCATTCACAA   120
HEINZ        TGCCTAGAGATCCTTTAATAGTTTCTGGAGTTGTTGGAGATGTTGTTGATCCATTCACAA   120
             ************************************************************

LA2093       GATGTGTAGACTTTGGTGTGGTTTACAACAATAGGGTGGTCTACAATGGATGTTCCTTGA   180
E42          GATGTGTAGACTTTGGTGTGGTTTACAACAATAGGGTGGTCTACAATGGATGTTCCTTGA   180
HEINZ        GATGTGTAGACTTTGGTGTGGTTTACAACAATAGGGTGGTCTATAATGGATGTTCCTTGA   180
             ******************************************* ****************

LA2093       GGCCTTCACAAGTTGTCAATCAACCTAGGGTTGACATTGATGGAGACGATCTTCGTACTT   240
E42          GGCCTTCACAAGTTGTCAATCAACCTGGGGTTGACATTGCTGGAGACGATCTTCGTACTT   240
HEINZ        GGCCTTCACAAGTTGTCAATCAACCTAGGGTTGACATTGATGGAGACGATCTTCGTACTT   240
             ************************** ************ ********************

LA2093       TTTACACTCTGGTATAAACTCATCGTTTTATTTCATATGATATACATATATATATATA--   298
E42          TTTACACTCTGGTATAAACTCATCGTTTTATTTCATATGATATACATATATATATATATA   300
HEINZ        TTTACACTCTGGTATAAACTCATCGTTTTATTTCATATGATATACATATATATATATATA   300
             **********************************************************

LA2093       --------TATATATATATATTTTCTTTCTATTTATACATTTTAATATCTCTAAATTATT   350
E42          TATATATATATATATATATATTTTCTTTCTATTTATAAATTTTAATATCTCTAAATTATT   360
HEINZ        TATATATATATATATATATATTTTCTTTCTATTTATACATTTTAATATCTCTAAATTATT   360
                     ***************************** **********************

LA2093       AACCTTTTGTCAATTGATTATGAGTAGAAGATCAAAAGGACAATATGTGCAAAGGCTTCT   410
E42          AACCTTTTGTCAATTGATTATGAGTAGAAGATCAAAAGGACAATATGTGCAAAGGCTTCT   420
HEINZ        AACCTTTTGTCAATTGATTATGAGTAGAAGATCAAAAGGACAATATGTGCAAAGGCTTCT   420
             ************************************************************

LA2093       AATTATGTGAATTTGTGTTAGTTTTAATTTTGATTCACCATCTAAGTACTTGTTTTGTGG   470
E42          AATTATGTGAATTTGTGTTAGTTTTAATTTTGATTCACCATCTAAGTACTTGTTTTATGG   480
HEINZ        AATTATGTGAATTTGTGTTAGTTTTAATTTTGATTCACCATCTAAGTACTTGTTTTGTGG   480
             ******************************************************** ***

LA2093       TTTTTATTTGAATTTGAGAACTCATAACATACTATTTATGATAATAAAAAATGTTAGTAA   530
E42          TTTTTATTTGAATTTGAGAACTCATAACATACTATTTATGATAATAAAAAATGTTAGTAA   540
HEINZ        TTTTTATTTGAATTTGAGAACTCATAACATACTATTTATGATAATAAAAAATGTTAGTAA   540
             ************************************************************

LA2093       CATGTATGTTTAATATTGCAAGCTTGAAAATATACAATATTTTTAAATTACTAATAATGT   590
E42          CATGTATGTTTAATATTGCAAGCTTGAAAATATACAATATTTTTAAATTACTAATAATGT   600
HEINZ        CATGTATGTTTAATATTGCAAGCTTGAAAATATACAATATTTTTAAATTACTAATAATGT   600
             ************************************************************

LA2093       CATGTAAAACATTTGGATATACAATATGGAAAATTATTTTTCCTAATTCTCAAAATATTT   650
E42          CATGTAATACATTTGGATATACAATATGGAAAATTATTTTTCCTAATTTTCAAAATATTT   660
HEINZ        CATGTAATACATTTGGATATACAATATGGAAAATTATTTTTCCTAATTTTCAAAATATTT   660
             ******* **************************************** ***********

LA2093       GAAATGTTTCTTTTCTTTTTGGAAGATTATGGTGGATCCTGATGCTCCAAATCCTAGCAA   710
E42          GAAATGTTTCTTTTCTTTTTGGAAGATTATGGTGGATCCTGATGCTCCAAACCCTAGCAA   720
HEINZ        GAAATGTTTCTTTTCTTTTTGGAAGATTATGGTGGATCCTGATGCTCCAAACCCTAGCAA   720
             *************************************************** ********

LA2093       CCCAAACCTGAGGGAATATTTGCACTGGTAAGTCATCTAGCTTTTATATATATATATATA   770
E42          CCCAAACCTGAGGGAATATTTGCACTGGTAAGTCATCTAGCTTATATATATATATATATA   780
HEINZ        CCCAAACCTGAGGGAATATTTGCACTGGTAAGTCATCTAGCTTATATATATATATATATA   780
             ******************************************* ****************

LA2093       TATATATATATATATATATATATATATATATATATATATATATATATATATATATATATA   830
E42          TATATAT-----------------------------------------------------A   788
HEINZ        TATATAT-----------------------------------------------------A   788
             *******                                                    *
```

```
LA2093      TATATATATATATTATAAATAGATAAAAATATTCATTTTGTTATATACTTCTTATTTCTC    890
E42         TATATATATATTATATAAATAGATAAAAATATTCATTTTGTTATATACTTCTTATTTCTC    848
HEINZ       TATATATATATTATATAAATAGATAAAAATATTCATTTTGTTATATACTTCTTATTTCTC    848
            ***********  ***********************************************

LA2093      TTAAATCAATCGTCGATAGCAAAGACAAAAATGTATGTGAGATTATATAAGAACCTAAGG    950
E42         TTAAATCAATCGTCGATAGCGAAGACAAAAATGTATGTGAGATTATATAAGAACCTAAGG    908
HEINZ       TTAAATCAATCGTCGATAGCGAAGACAAAAATGTATGTGAGATTATATAAGAACCTAAGG    908
            ******************** ***************************************

LA2093      AAAGTATTATTTCATAAAATGATAACTTTCTGATACACAAATTAATCAATATTTCAAATA    1010
E42         AAAGTATTATTTCATAAAATGATAACTTTCTGATACACAAATTAATCAATATTTCAAATA    968
HEINZ       AAAGTATTATTTCATAAAATGATAACTTTCTGATACACAAATTAATCAATATTTCAAATA    968
            ************************************************************

LA2093      AATATCAAATATCGAATAACAACGTATAAAAATAATAACTATTATCGATTGCTTAATCCC    1070
E42         AATACCAAATATCGAATAACAACCTAAAAATATAATAACTATTATCGATTGCTTAATCCC    1028
HEINZ       AATACCAAATATCGAATAACAACGTAAAAAAATAATAACTATTATCGATTGCTTAATCCC    1028
            **** ****************** ** *** *****************************

LA2093      CTTACAATTAATGTACCTAAACCTCTTTTTTTTTTTAAAAAAAATAATAATAATAATAAT    1130
E42         CTTACAATTAATGTACCTAAACCTCTTTTTTTTTTTAAA------------AATAATAAT    1076
HEINZ       CTTACAATTAATGTACCTAAACCTCTTTTTTTTTTTAAAAAAA-------AAATAATAAT    1081
            ***************************************            *********

LA2093      AATAATGTTTAACACATTATTTTTTTAATAGGTTGGTCACAGATATCCCAGCAACCACAG    1190
E42         AATAATGTTTAACACATTATTTTTTTAATAGGTTGGTCACAGATATCCCAGCAACCACAG    1136
HEINZ       AATAATGTTTAACACATTATTTTTTTAATAGGTTGGTCACAGATATCCCAGCAGCCACAG    1141
            ***************************************************** ******

LA2093      GAGCAACCTTTGGTAAGTTTTTCTTACATTATTACCTAATGGCTCGTAATTACGCAGTGA    1250
E42         GAGCAACCTTTGGTAAGTTTTTCTTACATTATTACCTAATGGCTCATAATTACGCAGTGA    1196
HEINZ       GAGCAACCTTTGGTAAGTTTTTCTTACATTATTACCTAATGGCTCGTAATTACGCAGTGA    1201
            ********************************************* **************

LA2093      CGAAGCAAGAAATTTAAATATACTTTATATTTACAATACATTGTATCCGTATCACTACAT    1310
E42         CGAAACAAGAAATTTAAATATACTTTATATTTACGATACATTGTATCCGTATCACTACAT    1256
HEINZ       CGAAGCAAGAAATTTAAATATACTTTATATTTACGATACATTGTATCCGTATCACTACAT    1261
            **** ***************************** *************************

LA2093      TTTTAATATAAGACGGTTAGTAATATACAAAATACAACTTGTATCATCATCACCTTAGTA    1370
E42         TTTTAATATAAGACGGTTAGTAATATACAAAATACAACTTGTATCATCATCACCTTAGTA    1316
HEINZ       TTTTAATATAAGACGGTTAGTAATATACAAAATACAACTTGTATCATCATCACCTTAGTA    1321
            ************************************************************

LA2093      GTACATTATTAGTACTATAGGCCCAATTATTACTACTAATAAAATAAGACTTAAAAAGAA    1430
E42         GTACATTATTAGTACTATAGGCCCAATTATGACTACTAATAAAATAAGACTTAAAAAGAA    1376
HEINZ       GTACATTATTAGTACTATAGGCCCAATTATGACTACTAATAAAATAAGACTTAAAAAGAA    1381
            ****************************** *****************************

LA2093      ACATAAAATCAAAATGAAGTATATACTATGTATATAAATGTTTTTGAAACAAGGAAAATA    1490
E42         ACATAAAATCAAAATGAAGTATATACTATGTATATAAATGTTTTTGAAACAAGGAAAATA    1436
HEINZ       ACATAAAATCAAAATGAAGTATATACTATGTATATAAATGTTTTTGAAACAAGGAAAATA    1441
            ************************************************************

LA2093      CGCGTATTGAATGTCTTTGTTACTAAACTCAAACTCTCGTTATACAGGCAATGAAGTCGT    1550
E42         CGCGTATTGAATGTCTTTGTTACTAAACTCAAACTCTCGTTATACAGGCAATGAAGTCGT    1496
HEINZ       CGCGTATTGAATGTCTTTGTTACTAAACTCAAACTCTCGTTATACAGGCAATGAAGTCGT    1501
            ************************************************************

LA2093      GGGCTACGAGAGCCCACGACCCTCGATGGGAATCCATCGTTATATTTTCGTGTTGTATCG    1610
E42         GGGCTACGAGAGCCCACGACCCTCGATGGGAATCCATCGTTATATTTTCGTGTTGTATCG    1556
HEINZ       GGGCTACGAGAGCCCACGACCCTCAATGGGAATCCATCGTTATATTTTCGTGTTGTATCG    1561
            ************************ ***********************************

LA2093      ACAATTGGGCCGCGATGCCATCGATGCACCGGACATAATCGATTCTAGACAAAATTTCAA    1670
E42         ACAATTGGGCCGCGATGCCATCGATGCACAGGACATAATCGATTCTAGACAAAATTTCAA    1616
HEINZ       ACAATTGGGCTGCGATGCCATCGATGCACCGGACATAATCGATTCTAGACAAAATTTCAA    1621
            ********** ****************** ******************************

LA2093      CACAAGAGACTTTGCTAGGTTTCACAATCTAGGTTTGCCTGTTGCTGCTGTTTACTTCAA    1730
```

```
E42       CACAAGAGACTTTGCTAGGTTTCACAATCTAGGTTTGCCTGTTGCTGCTGTTTACTTCAA  1676
HEINZ     CACAAGAGACTTTGCTAGGTTTCACAATCTAGGTTTGCCTGTTGCTGCTGTTTACTTCAA  1681
          ************************************************************

LA2093    TTGCAATAGGGAAGGTGGTACCGGTGGTCGTCGCCTATAAATCACCCCCCTCTCCTCGGG  1790
E42       TTGCAATAGGGAAGGTGGTACCGGTGGTCGTCGCCTATAAATCACCCCCCTCTCCTCGGG  1736
HEINZ     TTGCAATAGGGAAGGTGGTACCGGTGGTCGTCGCCTATAAATCACCCCCCTCTCCTCGGG  1741
          ************************************************************

LA2093    GTGCGATCCGTTCTCGAACTCTGTGTCAATGTCAGATGTTTTGTATAACGGATTTTTCTG  1850
E42       GTGCGATCCGTTCTCGAACTCTGTGTCAATGTCAGATGTTTTGTATAACGGATTTT----  1792
HEINZ     GTGCGATCCGTTCTCGAACTCTGTGTCAATGTCAGATGTTTTGTGTAACGGATTTT----  1797
          ******************************************** ***********

LA2093    TATCTATCGTTATTATTACTATATATCGATATCATATATGAGTTGATTTTGTTTGATAGT  1910
E42       ------------------------------------------------TTGTTTGATAGT  1804
HEINZ     ------------------------------------------------TTGTTTGATAGT  1809
                                                          ************

LA2093    CACTCAGCTAAATTGCTTATTAACTCAGAAAGTCATTTTTCTTTTTATCGAAGAAAATTG  1970
E42       CACTCAGCTAAATTGCTTATTAACTCAGAAAGTCATTTTTCTTTTTATCGAAGAAAATTG  1864
HEINZ     CACTCAGCTAAATTGCTTATTAACTCAGAAAGTCATTTTTCTTTTTATCGAAGAAAATTG  1869
          ************************************************************

LA2093    AAATTATGAGATAATAGCTATTATAGTTGAGTGATAATCTGAAAAAAAAAATCAACCATA  2030
E42       AAATTATGAGATAATAGCTATTATAGTTGAGTGATAATCTGAAAAAAAAAATCAACCATA  1924
HEINZ     AAATTATGAGATAATAGCTATTATAGTTGAGTGATAATCTGAAAAAAAAAATCAACCATA  1929
          ************************************************************

LA2093    ATATATATTATATATTATATATATACCCAAAAAATAAAAGTCTTGTTTGGTATATATTGA  2090
E42       ATATATATTATATATTATATATATACCCAAAAAATAAAAGTCTTGTTTGGTATATATTGA  1984
HEINZ     ATATATATTATATATTATATATATACCCAAAAAATAAAAGTCTTGTTTGGTATATATTGA  1989
          ************************************************************

LA2093    TATATATGTATATGTGTTTTATATTTTATTACTGAATTTTAATTTGTATGTATACGAGTC  2150
E42       TATATATGTATATGTGTTTTATATTTTATTACTGAATTTTAATTTGTATGTATACGAGTC  2044
HEINZ     TATATATGTATATGTGTTTTATATTTTATTACTGAATTTTAATTTGTATGTATACGAGTC  2049
          ************************************************************

LA2093    TACATGTAAAAGTGTGGATATATTTACTATATCTA  2185
E42       TACATGTAAAAGTGTGGATATATTTACTATATCTA  2079
HEINZ     TACATGTAAAAGTGTGGATATATTTACTATATCTA  2084
          ***********************************
```

Solyc05g053760

```
HEINZ      TGTGGATCAAACCCAAGAAAAAACACA-TAAAAAAAAAAAGTTTCAAAATCTGCAATCAA   59
E42        TGTGGATCAAACCCAAGAAAAAACACATAAAAAAAAAAAAGTTTCAAAATCTGCAATCAA   60
LA2093     TGTGGATCAAACCCAAGAAAAAACACATAAAAAAAAAAAAGTTTCAAAATCTGCAATCAA   60
           ***************************  *******************************

HEINZ      AAAGAGAGAAAAGATGTGTTGCAACTCCAATGGCGTAATCCCTACAAGCGAACCTCGCTT   119
E42        AAAGAGAGAAAAGATGTGTTGCAACTCCAATGGCGTAATCCCTACAAGCGAACCTCGCTT   120
LA2093     AAAGAGAGAAAAGATGTGTTGCAACTCCAATGGCGTAATCCCTACAAGCGAACCTCGCTT   120
           ************************************************************

HEINZ      GCCC-TTTTCTCCACCCACCCACCAACAATTTCTCCAAATCCTCGTCTATTTTTCCTTAA   178
E42        GCCCCTTTTCTCCACCCACCCACCAACAATTTCTCCAAATCCTCGTCTATTTTTCCTTAA   180
LA2093     GCCCCTTTTCTCCACCCACCCACCAACAATTTCTCCAAATCCTCGTCTATTTTTTCTTAA   180
           **** ************************************************* *****

HEINZ      CAATCCTTCGAATCATGGAGTTTTGAGGACAAAATTCGTTTCATACAAAGCTAAATCGAA   238
E42        CAATCCTTCGAATCATGGAGTTTTGAGGACAAAATTCGTTTCATACAAAGCTAAATCGAA   240
LA2093     CAATCCTTCGAATCATGGAGTTTTGAGGACAAAATTCGTTTCATACAAAGCTAAATCGAA   240
           ************************************************************

HEINZ      CCTAAACGATGTCGTTTCTTATACCGACACCGGTAAGAGCTTTTACGATCTTCTGGGTAT   298
E42        CCTAAACGATGTCGTTTCTTATACCGACACCGGTAAGAGCTTTTACGATCTTCTGGGTAT   300
LA2093     CCTAAACGATGTCGTTTCCTATACCGACACCGGTAAGAGCTTTTACGATCTTCTGGGTAT   300
           ****************** *****************************************

HEINZ      ACCGGAAAATGGATCTTTGTTGGAAATCAAGCAAGCGTACAAGCAATTAGTAAGGAAGTA   358
E42        ACCGGAAAATGGATCTTTGTTGGAAATCAAGCAAGCGTACAAGCAATTAGTAAGGAAGTA   360
LA2093     ACCGGAAAATGGATCTTTGTTGGAAATCAAGCAAGCGTACAAGCAATTAGTAAGGAAGTA   360
           ************************************************************

HEINZ      TCACCCGGATGTTTCACCTCCAGATCGGGTTGAAGAGTATACACAGAGGTTTATTAGGGT   418
E42        TCACCCGGATGTTTCACCTCCAGATCGGGTTGAAGAGTATACACAGAGGTTTATTAGGGT   420
LA2093     TCACCCGGATGTTTCACCTCCAGATCGGGTTGAAGAGTATACACAGAGGTTTATTAGGGT   420
           ************************************************************

HEINZ      TCAGGAAGCTTATGAAACGTTATCAGATCCTGGAATGAGAGCTTTGTATGATATAGATAT   478
E42        TCAGGAAGCTTATGAAACGTTATCAGATCCTGGAATGAGAGCTTTGTATGATATAGATAT   480
LA2093     TCAGGAAGCTTATGAAACGTTATCAGATCCTGGAATGAGAGCTTTGTATGATATAGATAT   480
           ************************************************************

HEINZ      GGCTAAAGGACTTCACTTTGGTTTCTCTGCTCGTTCACATGAGGTGTGTTTGCTTTTTCT   538
E42        GGCTAAAGGACTTCACTTTGGTTTCTCTGCTCGTTCACATGAGGTGTGTTTGCTTTTTCT   540
LA2093     GGCTAAAGGACTTCACTTTGGTTTCTCTGCTCGTTCACATGAGGTGTGTTTGCTTTTTCT   540
           ************************************************************

HEINZ      TTTTCAATTTGTGTCTTTCTTTAATTATTGAATGCATGAAAAATACCCCCAATTTGACTG   598
E42        TTTTCAATTTGTGTCTTTCTTTAATTATTGAATGCATGAAAAATACCCCCAATTTGACTG   600
LA2093     TTTTCAATTTGTGTCTTTCTTTAATTATTGAATGCATGAAAAATACCCCCAATTTGACTG   600
           ************************************************************

HEINZ      CAATTTTGCCTTCTCTGTCTATATTGAATGTTTATTTTGATATATTCTAGCTATACTTTA   658
E42        CAATTTTGCCTTCTCTGTCTATATTGAATGTTTATTTTGATATATTCTAGCTATACTTTA   660
LA2093     CAATTTTGCCTTCTCTGTCTATATTGAATGTTTATTTTGATATATTCTAGCTATACTTTA   660
           ************************************************************

HEINZ      GCTCAAAGCACAAACTTTAGTTTATGAAGTTAAAATCAGACAAACAAATTAAAACCCTAA   718
E42        GCTCAAAGCACAAAATTTAGTTTATGAAGTTAAAATCAGACAAACAAATTAAAACCCTAA   720
LA2093     GCTCAAAGCACAAAATTTAGTTTATGAAGTTA-AATCAGACAAACAAATTAAAACCCTAA   719
           ************** ***************** ***************************

HEINZ      TGGGATTGTAGAGAGTTTGACTTCTCATGTAATTACTCAGTTATGTAGTTATTATCTTAG   778
E42        TGGGATTGTAGAGAGTTTGACTTCTCATGTAATTACTCAGTTATGTAGTTATTATCTTAG   780
LA2093     TGGGATTGTAGAGAGTTTGACTTCTCATGTAATTACTCAGTTATGTAGTTATTATCTTAG   779
           ************************************************************

HEINZ      AAAGTCATGTTCTTTGTTGAAAAAACATTTGAAAAATGAAGAAGAAGAAAGTGACTTTGT   838
E42        AAAGTCATGTTCTTTGTTGAAAAAACATTTGAAAAATGAAGAAGAAGAAAGTGACTTTGT   840
LA2093     AAAGTCATGTTCTTTGTTGAAAAAACATTTGAAAAATGAAGAAGAAGAAAGTGACTTTGT   839
           ************************************************************
```

```
HEINZ       GAAATAATAATTATTACTAGTTGAGCGGCCATATGAGAAATCAACTCGAGTTCCGGGGTT    898
E42         GAAATAATAATTATTACTAGTTGAGCGGCCATATGAGAAATCAACTCGAGTTCCGGGGTT    900
LA2093      GAAATAATAATTATTACTAGTTGAGCGGCCATATGAGAAATCAACTCGAGTTCCGGGGTT    899
            ************************************************************

HEINZ       TCATTTATATTTGGGTCTGGTTAGGCATGATTCACTTATTTTATGCTGATAGTTGAAAAG    958
E42         TCATTTATATTTGGGTCTGGTTAGGCATGATTCACTTATTTTATGCTGATAGTTGAAAAG    960
LA2093      TCATTTATATTTGGGTCTGGTTAGGCATGATTCACTTATTTTATGCTGATAGTTGAAAAG    959
            ************************************************************

HEINZ       GATTAAGGGTATTTGCACATAAAGGTCAATGAAAAAGTAGGCATAGTAGAGTATGAAATT    1018
E42         GATTAAGGGTATTTGCACATAAAGGTCAATGAAAAAGTAGGCATAGTAGAGTATGAAATT    1020
LA2093      GATTAAGGGTATTTGCACATAAAGGTCAATGAAAAAGTAGGCATAGTAGAGTATGAAATT    1019
            ************************************************************

HEINZ       CTGAAGATCCATCCACTTGGATATTGGTTTTATTTTATTTGTTCTGTTTCTGTTTATTAC    1078
E42         CTGAAGATCCATCCACTTGCATATTGGTTTTATTTTATTTGTTCTGTTTCTGTTTATTAC    1080
LA2093      CTGAAGATCCATCCACTTGCATATTGGTTTTATTTTATTTGTTCTGTTTCTGTTTATTAC    1079
            ******************* ****************************************

HEINZ       CGGTAGTGTCCGAGCAGTTTGCATGCACCTCGACTAATCAACAGAGTGTGTGCTACTCTC    1138
E42         CGGTAGTGTCCGAGCAGTTTGCATGCACCTCGACTAATCAACAGAGTGTGTGCTACTCTC    1140
LA2093      CGGTAGTGTCCGAGCAGTTTGCATGCACCTCGACTAATCAACAGAGTGTGTGCTACTCTC    1139
            ************************************************************

HEINZ       TCTTTCCACTGAGGTTTAGGCAGATGGGACTACTGGTGTGACCTTTACTGGAATTCATAC    1198
E42         TCTTTCCACTGAGGTTTAGGCAGATGGGACTACTGGTGTGACCTTTACTGGAATTCATAC    1200
LA2093      TCTTTCCACTGAGGTTTAGGCAGATGGGACTACTGGTGTGACCTTTACTGGAATTCATAC    1199
            ************************************************************

HEINZ       CCAAGGTTATGAGACTTATCCACATCATAAAGGATTATATGCTGAAGATAAGAATTTGCG    1258
E42         CCAAGGTTATGAGACTTATCCACATCATAAAGGATTATATGCTGAAGATAAGAATTTGCG    1260
LA2093      CCAAGGTTATGAGACTTATCCACATCATAAAGGATTATATGCTGAAGATAAGAATTTGCG    1259
            ************************************************************

HEINZ       TATATGTTCTAAAAAAATGTAGAATCTTAGTATATTTGGCTAACTGTTGAGTTCAGAAGGG    1318
E42         TATATGTTCTAAAAAAATGTAGAATCTTAGTATATTTGGCTAACTGTTGAGTTCAGAAGGG    1320
LA2093      TATATGTTCTAAAAAAATGTAGAATCTTAGTATATTTGGCTAACTGTTGAGTTCAGAAGGG    1319
            ************************************************************

HEINZ       ATATTATGTCAAGATTGTGGAATATTTTCTGGAAAAAAAGTCACTTGTCTTTAAATTGTC    1378
E42         ATATTATGTCAAGATTGTGGAATATTTTCTGAAAAAAAAGTCACTTGTCTTTAAATTGTC    1380
LA2093      ATATTATGTCAAGATTGTGGAATATTTTCTGGAAAAAAAGTCACTTGTCTTTAAATTGTC    1379
            ******************************* ****************************

HEINZ       GGTATCTAGATGCCTGAGGAATGACAAGTCTTAAAATGATCGTGCTCGGGGTCAACTTAG    1438
E42         GGTATCTAGATGCCTGAGGAATGACAAGTCTTAAAATGATCGTGCTCGGGGTCAACTTAG    1440
LA2093      GGTATCTAGATGCCTGAGGAATGACAAGTCTTAAAATGATCGTGCTCGGGGTCAACTTAG    1439
            ************************************************************

HEINZ       GTGGGTTCCCCTTTTGGTCGGATGACTGCATAATATAACCAACAACAAAAAAAAAACACT    1498
E42         GTGGGTTCCCCTTTTGGTCGGATGACTGCATAATATAACCAACAACAAAA-AAAAACACT    1499
LA2093      GTGGGTTCCCCTTTTGGTCGGATGACTGCATAATATAACCAACAACAAAA-AAAAACACT    1498
            ************************************************** *********

HEINZ       TTATTGAGGCATCTACTTTAGCATTAGGCAGAAACTACATGTATTTTAATGGGGTATGAC    1558
E42         TTATTGAGGCATCTACTTTAGCATTAGGCAGAAACTACATGTATTTTAATGGGGTATGAC    1559
LA2093      TTATTGAGGCATCTACTTTAGCATTAGGCAGAAACTACATGTATTTTAATGGGGTATGAC    1558
            ************************************************************

HEINZ       AGAACTGCTGGCAACTAGGAATATATGGTTTAGTCATTTGGTCTTTTTCATATAACTTTG    1618
E42         AGAACTGCTGGCAATTAGGAATATATGGTTTAGTCATTTGGTCTTTTTCATATAACTTTG    1619
LA2093      AGAACTGCTGGCAATTAGGAATATATGGTTTAGTCATTTGGTCTTTTTCATATAACTTTG    1618
            ************** *********************************************

HEINZ       GTGTTCAATGGTGGTATTCCCTTGCAATGTTGATGCTTGTAATCTTTGGTGTTTCCACTG    1678
E42         GTGTTCAATGGTGGTATTCCCTTGCAATGTTGATGCTTGTAATCTTTGGTGTTTCCACTG    1679
LA2093      GTGTTCAATGGTGGTATTCCCTTGCAATGTTGATGCTTGTAATCTTTGGTGTTTCCACTG    1678
            ************************************************************

HEINZ       TTTTCTCTATTTGATGGACTAAATGAATGTTATCATGATAGATCTCATTGATCAAGTATG    1738
```

```
E42         TTTTCTCTATTTGATGGACTAAATGAATGTTATCATGATAGATCTCATTGATCAAGTATG    1739
LA2093      TTTTCTCTATTTGATGGACTAAATGAATGTTATCATGATAGATCTCATTGATCAAGTATG    1738
            ************************************************************

HEINZ       AGATAGTCTACTGTTTCGTGGAATAACTCAATTAGTGGAATCTTAAAGGAGAAGTATGAA    1798
E42         AGATAGTCTACTGTTTCGTGGAATAACTCAATTAGTGGAATCTTAAAGGAGAAGTATGAA    1799
LA2093      AGATAGTCTACTGTTTCGTGGAATAACTCAATTAGTGGAATCTTAAAGGAGAAGTATGAA    1798
            ************************************************************

HEINZ       AATACTTCATTTTTTCCAATTCTTGGGACAAGAGGAGTGAATGGGTGTAATCTTGGTTGA    1858
E42         AATACTTCATTTTTTCCAATTCTTGGGACAAGAGGAGTGAATGGGTGTAATCTTGGTTGA    1859
LA2093      AATACTTCATTTTTTCCAATTCTTGGGACAAGAGGAGTGAATGGGTGTAATCTTGGTTGA    1858
            ************************************************************

HEINZ       TTCCAATAGACTTTAATATGCGATGTTCTTTTGCGTCAGATCCAACGTTTAAATGATAAC    1918
E42         TTCCAATAGACTTTAATATGCGATGTTCTTTTGCGTCAGATCCAACGTTTAAATGATAAC    1919
LA2093      TTCCAATAGACTTTAATATGCGATGTTCTTTTGCGTCAGATCCAACGTTTAAATGATAAC    1918
            ************************************************************

HEINZ       AAGTTGTTTGACCTTAGCATACTACTTCTGCCACTACATGTCTATATCCAAATCACTTTC    1978
E42         AAGTTGTTTGACCTTAGCATACTACTTCTGCCACTACATGTCTATATCCAAATCACTTTC    1979
LA2093      AAGTTGTTTGACCTTAGCATACTACTTCTGCCACTACATGTCTATATCCAAATCACTTTC    1978
            ************************************************************

HEINZ       GATTCTATTTATCTCATCAAAACTTGTCAACATAAGATTTTTCTTAGACAGCCACTCTGT    2038
E42         GATTCTATTTATCTCATCAAAACTTGTCAACATAAGATTTTTCTTAGACAGCCACTCTGT    2039
LA2093      GATTCTATTTATCTCATCAAAACTTGTCAACATAAGATTTTTCTTAGACAGCCACTCTGT    2038
            ************************************************************

HEINZ       TCTTTCGACATTCATGTACTTGATTGCTTGTACGCTCTTATGACATGTTTTATGTTCCAG    2098
E42         TCTTTCGACATTCATGTACTTGATTACTTGTACGCTCTTATGACATGTTTTATGTTCCAG    2099
LA2093      TCTTTCGACATTCATGTACTTGATTGCTTGTACGCTCTTATGACATGTTTTATGTTCCAG    2098
            ************************* **********************************

HEINZ       AATCTAGGTATCCTCTTTGCTAATTCGAGTTTTTTGTTATGCTTTAGGCGATGGAGGAGA    2158
E42         AATCTAGGTATCCTCTTTGCTAATTCGAGTTTTTTGTTATGCTTTAGGCGATGGAGGAGA    2159
LA2093      AATCTAGGTATCCTCTTTGCTAATTCGAGTTTTTTGTTATGCTTTAGGCGATGGAGGAGA    2158
            ************************************************************

HEINZ       GAGGTGAGTGGAAAAAACCGTTGGCAGTCTCAGCTATCAGAGCTCAAGAGACGAAGAACAT    2218
E42         GAGGTGAGTGGAAAAAACCGTTGGCAGTCTCAGCTATCAGAGCTCAAGAGACGAAGAACAT    2219
LA2093      GAGGTGAGTGGAAAAAACCGTTGGCAGTCTCAGCTATCAGAGCTCAAGAGACGAAGAACAT    2218
            ************************************************************

HEINZ       ACAAGGAATCCAGTAACAGTATGTCTTGGGGAGCACGGATGCGCAAGCAAAGGGATGATG    2278
E42         ACAAGGAATCCAGTAACAGTATGTCTTGGGGAGCACGGATGCGCAAGCAAAGGGATGATG    2279
LA2093      ACAAGGAATCCAGTAACAGTATGTCTTGGGGAGCACGGATGCGCAAGCAAAGGGATGATG    2278
            ************************************************************

HEINZ       ATGCATAATCTTAATCGATAATAACTAATACATCTTATTTTATTTTGGATATACAGGAAT    2338
E42         ATGCATAATCTTAATCGATAATAACTAATACATCTTATTTTATTTTGGATATACAGGAAT    2339
LA2093      ATGCATAATCTTAATCGATAATAACTAATACATCTTATTTTATTTTGGATATACAGGAAT    2338
            ************************************************************

HEINZ       GTTTTTAGTTTTCTGCTAGTACTAACCATATATGAATCACCTTATAGGCAGTGCATATTG    2398
E42         GTTTTTAGTTTTCTGCTAGTACTAACCATAT------------ATAGGCAGTGCATATTG    2387
LA2093      GTTTTTAGTTTTCTGCTAGTACTAACCATATATGAATCACCTTATAGGCAGTGCATATTG    2398
            *******************************             *****************

HEINZ       TATATAAAGTATATGAATCAAGAATATCATTTCCGTTGGTACACTAGTTTCTGGTACCTT    2458
E42         TATATAAAGTATATGAATCAAGAATATCATTTCCGTTGGTACACTAGTTTCTGGTACCTT    2447
LA2093      TATATAAAGTATATGAATCAAGAATATCATTTCCGTTGGTACACTAGTTTCTGGTACCTT    2458
            ************************************************************

HEINZ       GTTACTTTCAGAAAAGACTGTTGTTTTACAGTAGTATATTTAAGCAAGTTGGTGGCTTTC    2518
E42         GTTACTTTCAGAAAAGACTGTTGTTTTACAGTAGTATATTTAAGCAAGTTGGTGGCTTTC    2507
LA2093      GTTACTTTCAGAAAAGACTGTTGTTTTACAGTAGTATATTTAAGCAAGTTGGTGGCTTTC    2518
            ************************************************************

HEINZ       ATTGATGTTGGCTGTATATCATAAATTGTGCGGGCTCTTCACTTTTATTGACATGTT---    2575
E42         ATTGATGTTGGCTGTATATCATAAATTGTGCGGGCTCTTCACTTTTATTGACATGCTCAT    2567
LA2093      ATTGATGTTGGCTGTATATCATAAATTGTGCGGGCTTTTCACTTTTATTGACATGCTCAT    2578
```

```
             ************************************ ****************** *

HEINZ        ---TTCAAAAACTACTTTACTTTTGGAG-AATTCAACGCATGTTGTCATTGAAGAGTTCG    2631
E42          GTTTTCAAAAACTACACTACTTTTGGAGAATTC-AACGCATGTTGTCATTGAAGAGTTCG    2626
LA2093       GTTTTCAAAAACTACACTACTTTTGGAGAATTCTAACGCATGTTGTCATTGAAGAGTTCG    2638
                ************  *********** * *  **************************

HEINZ        AGCAACATAGTATATATATCTATATCATTT    2661
E42          AGCAACATAGTATATAAATCTATATCATTT    2656
LA2093       AGCAACATAGTATATATATCTATATCATTT    2668
             **************** *************
```

Solyc05g055660

```
E42         TTGAAATTTTGACGCCATCCTGGGGGATACACTGTTTCACGTCCCAATTGTCGAAATAAA     60
HEINZ       TAGAAATTTTGACGCCATCCTGGGGGATACACTGTTTCACGTCCCAATTGTCGAAATAAA     60
LA2093      TTGAAATTTTGACGCCATCCTGGGGGATACACTGTTTCACGTCCCAATTGTCGAAATAAA     60
            * **********************************************************

E42         ACCAAAACGAATCGATGAATTCCCATCGTTGGCGTTGGATTCTCGTAGCATACTATTTCA     120
HEINZ       ACCAAAACGAATCGATGAATTCCCATCGTTGGCGTTGGATTCTCGTAGCATACTATTTCA     120
LA2093      ACCAAAACGAATCGATGAATTCCCATCGTTGGCGTTGGATTCTCGTAGCATACTATTTCA     120
            ************************************************************

E42         TTTCCTAGTATAAGGTAAAATAATCGAATAATTAATGACGATATAAAAAAGATTTTTAAA     180
HEINZ       TTTCCTAGTATAAGGTAAAATAATCGAATAATTAATGACGACATAAAAAAGATTTTTAAA     180
LA2093      TTTCCTAGTATAAGGTAAAATAATCGAATAATTAATGACGATATAAAAAAGATTTTTAAA     180
            ***************************************** ******************

E42         ATTTACGATCTTTTCGTGATTTTTCTAGCTAGGTTATTGACCATATATGTGTGTGTATAT     240
HEINZ       ATTTACGATCCTTTCGTGATTTTTCTAGCTAGGTTATTGACCATATATGTGTGTGTATAT     240
LA2093      ATTTACGATCTTTTCGTGATTTTTCTAGCTAGGTTATTGACCATATATGTGTGTGTATAT     240
            ********** *************************************************

E42         AAGAGATAAGTTATCATATACTATATGATTTATTTTCTCAAAAACTGTTACTTTATAGTG     300
HEINZ       GAGAGATAAGTTATCATATACTATATGATTTATTTTCTCAAAAACTGTTACTTTATAGTG     300
LA2093      AAGAGATAAGTTATCATATACTATATGATTTATTTTCTCAAAAACTGTTACTTTATAGTG     300
             ***********************************************************

E42         TCAATACATAGAACTTTGTGTAAATGACAAAACAAATATATTTATAATTAAAAGGATTTA     360
HEINZ       TCAATACATAGAACTTTGTGTAAATGACAAAACAAATATATTTATAATTAAAAGGATTTA     360
LA2093      TCAATACATAGAACTTTGTGTAAATGACAAAACAAATATATTTATAATTAAAAGGATTTA     360
            ************************************************************

E42         CCAAAGCTTGTATCTGTAGTTGCTGGGATATCTGTGACCAGCCTGAAAAAAAT-ATATTA     419
HEINZ       CCAAATCTTGTATCTGTAGTTGCTGGGATATCTGTGACCAGCCTGAAAAAAAATATATTA     420
LA2093      CCAAAGCTTGTATCTGTAGTTGCTGGGATATCTGTGACCAGCCTGAAAAA-AATATATTA     419
            ***** ******************************************** *  ******

E42         AATAAAGTGTTAAA---ATAATAATAATAATAATATATTTAGTATAATTATATAAGTGGT     476
HEINZ       AATAAAGTGTTAAAATAATAATAATAATAATAATATATTTAGTATAATTATATAAGTGGT     480
LA2093      AATAAAGTGTTAAAATAATAATAATAATAATAATATATTTAGTATAATTATATAAGTGGT     479
            **************   *******************************************

E42         GTTTAGGTAGAGTGTTATGTGTATTTACGTACGATATATCTGCATTGTGAAGATAGAGAA     536
HEINZ       GTTTAGGTAGAGTGTTATGTGTATTTACGTACGATATATCTGCATTGTGAAGGTAGAGAA     540
LA2093      GTTTAGGTAGAGTGTTATGTGTATTTACGTACGATATATCTGCATTGTGAAGGTAGAGAA     539
            **************************************************** *******

E42         ATTGTTTTTGATAGACTCTTGATTTAAGTAGCATGTATGAATAGGGAATATAGTAGTGAA     596
HEINZ       ATTGTTTTTGATAGACTCTTGATTTAAGTAGCATGTATGAATAGGGAATATAGTAGTGAA     600
LA2093      ATTGTTTTTGATAGACTCTTGATTTAAGTAGCATGTATGAATAGGGAATATAGTAGTGAA     599
            ************************************************************

E42         GAAGTTATGATGAAAATAATCGAGAAAACAATACAATAAACAGAGAAAAAGAAATAATAT     656
HEINZ       GAAGTTATGATGAAAATAATCGAGAAAACAATACAATAAACAGAGAAAAAGAAATAACGT     660
LA2093      GAAGTTATGATGAAAATAATCGAGAAAACAATACAATAAACAGAGAAAAAGAAATAACGT     659
            *********************************************************  *

E42         ATTACCATAAAAATCGAAGAATAAAATATACAAGAGATAATAAAAATAACAATAATAAGG     716
HEINZ       ATTACCATAAAAATCGAAGAATAAAATATACGAGAGATAATAAAAATAACAATAATAACG     720
LA2093      ATTACCATAAAAATCGAAGAATAAAATATACAAGAGATAATAAAAATAACAATAATAACG     719
            ******************************* ************************** *

E42         ACGATGATGATGATTAGGAAAACTAGAGTAATAGGATTTTCAGTACACATGACATTATGA     776
HEINZ       ACGATGATGATGATTAGGAAAACTAGAGTAATAGGATTTTCAGTACACATGACATTATGA     780
LA2093      ACGATGATGATGATTAGGAAAACTAGAGTAATAGGATTTTCAGTACACATGACATTATGA     779
            ************************************************************

E42         TTTTATGTTATCAATTGTGGGTTTTCACTTTGCATTATGAGCCTAACCAAGTTTCTTGAT     836
HEINZ       TTTTATGTTATCAATTGTGGGTTTTCACTTTGCATTATGAGCCTAACCAAGTTTCTTGAT     840
LA2093      TTTTATGTTATCAATTGTGGGTTTTCACTTTGCATTATGAGCCTAACCAAGTTTCTTGAT     839
            ************************************************************
```

```
E42      TCGAGTTTCATCGTCATTATTAATTCAAAAAAAA-AAAAGAATTTTTACGTGTTTGATCT   895
HEINZ    TCGAGTTTCATCGTCATTATTAATTCAAAAAAAAAAAAAGAATTTTTACGTGTTTGATCT   900
LA2093   TCGAGTTTCATCGTCATTATTAATTCAAAAAAA--AAAAGAATTTTTACGTGTTTGATCT   897
         *********************************  *************************

E42      AATAAGATCGAAAATACTAACCAGTGTAGATACTCCCTCAAGTTAGGATTGCTTGGGCTT   955
HEINZ    AATAAGATCGAAAATACTAACCAGTGTAGATACTCCCTCAAGTTAGGATTGCTTGGGCTT   960
LA2093   AATAAGATCGAAAATACTAACCAGTGTAGATACTCCCTCAAGTTAGGATTGCTTGGGCTT   957
         ************************************************************

E42      GGAGCATCAGGATCCACCATAATCTTCATAAAAAAACAATTACAAAAGAATCTCAAACAA   1015
HEINZ    GGAGCATCAGGATCCACCATAATCTTCATAAAAAAACAATTACAAAAGAATCTCAAACAA   1020
LA2093   GGAGCATCAGGATCCACCATAATCTTCAAAAAAAAACAATTACAAAAGAATCTCAAACAA   1017
         **************************** *******************************

E42      CAATGTAATCAAAACATAAATAAAGAAAAATTAAAAGAAAGTAAATACTATCTCCAAATA   1075
HEINZ    CAATGTAATCAAAACATAAATAAAGAAAAATTAAAAGAAAGTAAATACTATCTCCAAATA   1080
LA2093   CAATGTAATCAAAACATAAATAAAGAAAAATTAAAAGAAAGTAAATACTATCTCCAAATA   1077
         ************************************************************

E42      TTAATAGCGTCACAAAAAATAGAACAGAGAGAGTCAGAGAAAAGTAATAGTCAATAGCTC   1135
HEINZ    TTAATAGCGTCACAAAAAATAGAACCGAGAGAGTCAGAGAAAAGTAATAGTCAATAGCTC   1140
LA2093   TTAATAGCGTCACAAAAAATAGAACAGAGAGAGTCAGAGAAAAGTAATAGTCAATAGCTC   1137
         ************************* **********************************

E42      ATGAAAACAGTGAAAACATATCTAATACCGTCTTGATAATAGAAAATGAAGCCTTTGTTT   1195
HEINZ    ATGAAAACAGTGAAAACATATCTAATACCGTCTTGATAATAGAAAATGAAGCCTTTGTTT   1200
LA2093   ATGAAAACAGTGAAAACATATCTAATACCGTCTTGATAATAGAAAATGAAGCCTTTGTTT   1197
         ************************************************************

E42      AACTCTCAGAATAGTAATATTATCACCTAAAGTAAAAATGAACGAGCGAATATATAAAAA   1255
HEINZ    AACTCTCAGAATAGTAATATTATCACCTAAAGTAAAAATGAACGAGCGAATATATAAAAA   1260
LA2093   AACTCTCAGAATAGTAATATTATCACCTAAAGTAAAAATGAACGAGCGAATATATAAAAA   1257
         ************************************************************

E42      ATAATATAAGCGATAATAGTTAATAATAACAAAAACATTAGAAAAGAGTAAAAAACCTCT   1315
HEINZ    ATAATATAAGCGATAATAGTTAATAATAACAAAAACATTAGAAAAGAGTAATAAACCTCT   1320
LA2093   ATAATATAAGCGATAATAGTTAATAATAACAAAAACATTAGAAAAGAGTAATAAACCTCT   1317
         *************************************************** ********

E42      TATAGCTTCACATAAAATAAATAAAAAGAGTGAATTTTCTTTTTT---TTTTACTTAAGA   1372
HEINZ    TATAGCTTCACATAAAATAAATAAAAAGAGTGAATTTTCTTCTTTTTTTTTTACTTAAGA   1380
LA2093   TATAGCTTCACATAAAATAAATAAAAAGAGTGAATTTTCTTT--TTTTTTTTACTTAAGA   1375
         *****************************************   *   ************

E42      GTAGTTAATAGCACATGAAATTAACATTAAAAATAATATCTTAAATCGTCTTGATCGTTG   1432
HEINZ    GTAGTTAATAGCTCATGAAATTAACAT-TAAAATAATATCTTAAATCGTCTTGATCGTTG   1439
LA2093   GTAGTTAATAGCTCATGAAATTAACAT-TAAAATAATATCTTAAATCGTCTTGATCGTTG   1434
         ************ **************  *******************************

E42      AAAATTAAACCTAAGTTTTAACTCTCATAATAGTAATAGTACCACGTAAGATAATGCAAG   1492
HEINZ    AAAATTAAACCTAAGTTTTAACTCTCAGAATAGTAATAGTACCACGTAAGATAATGCAAG   1499
LA2093   AAAATTAAACCTAAGTTTTAACTCTCAGAATAGTAATAGTACCACGTAAGATAATGCAAG   1494
         *************************** ********************************

E42      TGAGGATGAATATATAATATATAATTAATAATCCAAAAAATTAGATAAAAAATATTAAA-   1551
HEINZ    TGAGGATGAATATATAATATATAATTAATAATCCAAAAAATTAGATAAAAAATATTAAA-   1558
LA2093   TGAGGATGAATATATAATATATAATTAATAATCCAAAAAATTAGATAAAAAATATTAAAC   1554
         ***********************************************************

E42      CCTTTGTGACGATCTCCAAATGATAATAGCGTCACATAAAATAAGAGAGAACAAAAAAGA   1611
HEINZ    CCTTTGTGACGATCTCCAAATGATAATAGCGTCACATAAAATAAGAGAGAACAAAAAAGA   1618
LA2093   CTTTTGTGACGATCTCCAAATGATAATAGCGTCACATAAAATAAAGAGAACAAAAAAGA    1614
         * ****************************************** ***************

E42      AATACTATAATTAATAATATAAGAAACAATAGTCGATAATTCAGAACATTAAACCTCTAT   1671
HEINZ    AATACTATAATTAATAATATAAGAAACAATAGTCGATAATTCAGAACATTAAACCTCTAT   1678
LA2093   AATACTATAATTAATAATATAAGAAACAATAGTCGATAATTCAGAACATTAAACCTCTAT   1674
         ************************************************************

E42      AACTCTCCAAATAGTATATAATAGTGTCACATAAAATACAATAGAACAAGACAGAGCGAG   1731
```

```
HEINZ       AACTCTCCAAATAGTATATAATAGTGTCACATAAAATACAATAGAACAAGACAGAGCGAG    1738
LA2093      AACTCTCCAAATAGTATATAATAGTGTCACATAAAATACAATAGAACAAGACAGAGCGAG    1734
            ************************************************************

E42         TAAAATTTAAAATTTACCAGAGTGTAAAAGGTGCGAAGATCGTCCCCTCCGATATAAACC    1791
HEINZ       TAAAATTTAAAATTTACCAGAGTGTAAAAGGTGCGAAGATCGTCCCCTCCGATATAAACC    1798
LA2093      TAAAATTTAAAATTTACCAGAGTGTAAAAGGTGCGAAGATCGTCCCCTCCGATATAAACC    1794
            ************************************************************

E42         TTAGGTTGCATAACAACTTGTGAAGGTTTCAACACACATGCATTGTTCACTTCTCTATTA    1851
HEINZ       TTAGGTTGCATAACAACTTGTGAAGGTTTCAACACACATGCATTGTTCACTTCTCTATTA    1858
LA2093      TTAGGTTGCATAACAACTTGTGAAGGTTTCAACACACATGCATTGTTCACTTCTCTATTA    1854
            ************************************************************

E42         TTATAAACAACTCTAAGATCAACAGACCTAGTGAATGGATCTAAAACTTCACCTATCACA    1911
HEINZ       TTATAAACAACTCTAAGATCAACAGACCTAGTGAATGGATCTAAAACTTCACCTATCACA    1918
LA2093      TTATAAACAACTCTAAGATCAACAGACCTAGTGAATGGATCTAAAACTTCACCTATCACA    1914
            ************************************************************

E42         CGACCAACTATCAATGGATCAACTCTAGGCAT  1943
HEINZ       CGACCAACTATCAATGGATCAACTCTAGGCAT  1950
LA2093      CGACCAACTATCAATGGATCAACTCTAGGCAT  1946
            ********************************
```

Solyc07g021000

```
E42         TGACCCCTATGATAATGGAAGGGGATGACTACTTCTTGCCCGGTGGAAATGGAGGGTTAG    60
HEINZ       TGACCCCTATGATAATGGAAGGGGATGACTACTTCTTGCCCGGTGGAAATGGAGGGTTAG    60
LA2093      TGACCCCTATGATAATGGAAGGGGATGACTACTTCTTGCCCGGTGGAAATGGAGGGTTAG    60
            ************************************************************

E42         TTCATGCCCTAACTAAAAATGTGCCTACTATTTTTGAAAAAATTGTACATGCCACTAGTT    120
HEINZ       TTCATGCCCTAACTAAAAATGTGCCTTCTATTTTTGAAAAAATTATACATGCCACTAGTT    120
LA2093      TTCATGCCCTAACTAAAAATGTGCCTTCTATTTTTGAAAAAATTATACATGCCACTAGTT    120
            ************************** ***************** ***************

E42         ATAATAGAAATAGTTTGAAGGTGATTAATGGAGGCCAATTATTTGAGGGAGATGTGACAT    180
HEINZ       ATAATAGAAATAGTTTGAAGGTGATTAATGGAGGCCAATTATTTGAGGGAGATGTGACAT    180
LA2093      ATAATAGAAATAGTTTAAAGGTGATTAATGGAGGCCAATTATTTGAGGGAGATGTGACAT    180
            **************** *******************************************

E42         TTCCACATTTCCTCTTAGAGTTTTGA 206
HEINZ       TTCCACATTTCCTCTTAGAGTTTTGA 206
LA2093      TTCCACATTTCCTCTTAGAGTTTTGA 206
            **************************
```

Solyc07g026810

```
LA2093      TTTGAAAATCCAACCGTTACGAATTTCACACAGAGGCAAAGAAATAAACCATCATACGAA    60
E42         TTTGAAAATCCAACCGTTACGAATTTCACACAGAAGCAAAGAAATAAACCATCAGACGAA    60
HEINZ       TTTGAAAATCCAACCGTTACGAATTTCACACAGAGGCAAAGAAATAAACCATCATACGAA    60
            ********************************** ******************* *****

LA2093      AG-TTGTAATCGTAACTCTTTTTGCTTGGAGTCCACATTTCATTTTCAACAGGCATCTCT    119
E42         AGTTTGTAATCGTAACTCTTTTTGCTTGGAGTCCACATTTCATTTTCAACAGGCATCTCT    120
HEINZ       AG-TTGTAATCGTAACTCTTTTTGCTTGGAGTCCACATTTCATTTTCAACAGGCATCTCT    119
            ** *********************************************************

LA2093      CTCATGCTCTCTTTTCCCAACTCAGTATAAAGGACAGGGTAGGAACATTTTACCCAGAAT    179
E42         CTCATGCTCTCTTTTCTCAACTCAGTATAAAGGACAGGGTAGGAACATTTTACCCAGAAT    180
HEINZ       CTCATGCTCTCTTTTCCCAACTCAGTATAAAGGACAGGGTAGGAACATTTTACCCAGAAT    179
            **************** *******************************************

LA2093      GTGAGACTACAATTTATTTGTCAACTTTTGGGCAATCTATTTTAAACTGACCCAGAAAAT    239
E42         GTGAGACTACAATTTATTTGTCAACTTTTGGGCAATCTATTTTAAACTGACCCAGAAAAT    240
HEINZ       GTGAGACTACAATTTATTTGTCAACTTTTGGGCAATCTATTTTAAACTGACCCAGAAAAT    239
            ************************************************************

LA2093      GCTTAGACGACGATTCTGCGTCTAACAGAAATCATGGGGTCAAGTC--------------    285
E42         GCTTAGACGACGATTCTGCGTCTAACAGAAATCATGGGGTCAAGTCAATTCCAGGCTAGT    300
HEINZ       GCTTAGACGACGATTCTGCGTCTAACAGAAATCATGGGGTCAAGTCAATTCCAGGCTAGT    299
            **********************************************

LA2093      --------------------------------------------AATTCAAGGAGGCAGG    301
E42         ATCAGCTAATTTTGGCTCTCATCTGGCCGTCTCAGTCCTTGAATAATTCAAGGAGGCAGG    360
HEINZ       ATCAGCTAATTTTGGCTCTCATCTGGCCGTCTCAGTCCTTGAATAATTCAAGGAGGCAGG    359
                                                        ****************

LA2093      ATCAGAGTGAGCTTCATACTACCATTGCTTCTTCCCCTTTTTCTTTCTCTTGTTTCCACT    361
E42         ATCAGAGTGAGCTTCATACTACCATTGCTTCTTCCCCTTTTTCTTTCTCTTGTTTCCACT    420
HEINZ       ATCAGAGTGAGCTTCATACTACCATTGCTTCTTCCCCTTTTTCTTTCTCTTGTTTCCACT    419
            ************************************************************

LA2093      TCCACTACCGTTGCTTGTACTACTACTACTACTCTTGGAGGTACTTCCAGCTGGTGTAGA    421
E42         TCCACTACCGTTGCTTGTACTACTACTACTACTCTTGGAGGTACTTCCAGCTGGTGTAGA    480
HEINZ       TCCACTACCGTTGCTTGTACTACTACTACTACTCTTGGAGGTACTTCCAGCTGGTGTAGA    479
            ************************************************************

LA2093      TGGGCTCTGTGGCGTGCTGCCATCAAAATCATCAAACATACCCGCTTGTACAGCATTCTG    481
E42         TGGGCTCTGTGGCGTGCTTCCATCAAAATCATCAAACATACCCGCTTGTACAGCATTCTG    540
HEINZ       TGGGCTCTGTGGCGTGCTGCCATCAAAATCATCAAACATACCCGCTTCTACAGCATTCTG    539
            ****************** **************************** ************

LA2093      TAGCCACTCGACAAATTCTTCCTCGGTCATGCTCTCTTCCATATTTGGCATTGGCATCCC    541
E42         TAGCCACTCGACAAATTCTTCCTCGGTCATGCTCTCTTCCATATTTGGCATTGGCATCCC    600
HEINZ       TAGCCACTCGACAAATTCTTCCTCGGTCATGCTCTCTTCCATATTTGGCATTGGCATCCC    599
            ************************************************************

LA2093      ACTTTTTTGTCCTGAGCTACTTCTCTTCCAATTGCTGTTCTTCAAGGTTACACTAGTATT    601
E42         ACTTTTTTGTCCTGAGCTACTTCTCTTCCCATTGCTGTTCTTCAAGGTTACACTAGTATT    660
HEINZ       ACTTTTTTGTCCTGAGCTACTTCTCTTCCAATTGCTGTTCTTCAAGGTTACACTAGTATT    659
            ***************************** ******************************

LA2093      TACATGGAAGCTTGGTTTGTGACTGTTGACCGGACATCTCATTCCCTGCACAACCAGCAT    661
E42         TACATGGAAGCTTGGTTTGTGACTGTTGACCGGACATCTCATTCCCTGCACAACCAGCAT    720
HEINZ       TACATGGAAGCTTGGTTTGTGACTGTTGACCGGACATCTCATTCCCTGCACAACCAGCAT    719
            ************************************************************

LA2093      AATGTTGAATTCTTCAAGCACATAATGTAGATACAGAGGTACATCCGCTCAGTTAAATTA    721
E42         AATGTTGAATTCTTCAAGCACATAATGTAGATACAGAGGTACATCCGCTCAGTTAAATTA    780
HEINZ       AATGTTGAATTCTTCAAGCACATAATGTAGATACAGAGGTACATCCGCTCAGTTAAATTA    779
            ************************************************************

LA2093      GGTCATTTTGATGATTAACCAACCTTAAAAGATCTAGAGGCACTCTTCCATTTAGAACTA    781
E42         GGTCATTTTGATGATTAACCAACCTTAAAAGATCTAGAGGCACTCTTCCATTTAGAACTA    840
HEINZ       GGTCATTTTGATGATTAACCAACCTTAAAAGATCTAGAGGCACTCTTCCATTTAGAACTA    839
            ************************************************************
```

```
LA2093      CAACGTAAGGAGGCATACATGGTGTGTTTAAAACATTTTTTTTTCATTGTTCAGGCAAAC    841
E42         CAACGTAAGGAGGCATACATGGTGTGTTTAAAACATTTTTTTTTCATTGTTCAGGCAAAC    900
HEINZ       CAACGTAAGGAGGCATACATGGTGTGTTTAAAACATTTTTTTTTCATTGTTCAGGCAAAC    899
            ************************************************************

LA2093      TTAAGAAGAGACAAAATTACAAGTTGTTTGGGCTTAAGAATTGTCACAATATGGTGAAGT    901
E42         TTAAGAAGAGACAAAATTACTAGTTGTTTGGGCTTAAGAATTGTCACAATATGGTGAAGT    960
HEINZ       TTAAGAAGAGACAAAATTACAAGTTGTTTGGGCTTAAGAATTGTCACAATATGGTGAAGT    959
            ******************** ***************************************

LA2093      GAAGCCACTGAGATCTTCCTTGACATGTCAGTTAACAAACTTATAGTGGAAACATAGAAG    961
E42         GAAGCCACTGAGATCTTCCTTGACATGTCAGTTAACAAACTTATAGTGGAAACATAGAAG    1020
HEINZ       GAAGCCACTGAGATCTTCCTTGACATGTCAGTTAACAAACTTATAGTGGAAACATAGAAG    1019
            ************************************************************

LA2093      CAAACAGGGGATCCATATTTTTTGATAAGACCCATTCTATGATTCTACACTGCTAATTAT    1021
E42         CAAACAGGGGATCCATATTTTTTGATAAGACCCATTCTATGATTCTACACTGCTAATTAT    1080
HEINZ       CAAACAGGGGATCCATATTTTTTGATAAGACCCATTCTATGATTCTACACTGCTAATTAT    1079
            ************************************************************

LA2093      TCCTGTATATGAGCATTTTCTGTCCACTCCTCATCCATGGGAGTTGATCTTTCCTAAATA    1081
E42         TCCTGTATATGAGCATTTTCTGTCCACTCCTCATCCATGGGAGTTGATCTTTCCTAAATA    1140
HEINZ       TCCTGTATATGAGCATTTTCTGTCCACTCCTCATCCATGGGAGTTGATCTTTCCTAAATA    1139
            ************************************************************

LA2093      TGGAGAAAAGAACAGTATAGTACCCATGGAAGATAAAACTGAAGTTGGTCCAAGCTTAAC    1141
E42         TGGAGAAAAGAACAGTATAGTACCCATGGAAGATAAAACTGAAGTTGGTCCAAGCTTAAC    1200
HEINZ       TGGAGAAAAGAACAGTATAGTACCCATGGAAGATAAAACTGAAGTTGGTCCAAGCTTAAC    1199
            ************************************************************

LA2093      AAGTAACTATGATGGCATTAGAATCAGTCAGACAGAAGCCCTTCTTCCCCCAAAAAATAA    1201
E42         AAGTAACTATGATGGCATTAGAATCAGTCAGACAGAAGCCCTTCTTCCCCCAAAAAATAA    1260
HEINZ       AAGTAACTATGATGGCATTAGAATCAGTCAGACAGAAGCCCTTCTTCCCCCAAAAAATAA    1259
            ************************************************************

LA2093      GAGACATACAAT-AAACCTTACTGAATTAATATAGGTGGGACCACGAAATAGAGATATGA    1260
E42         GAGACATACAATAAAACCTTACTGAATTAATATAGGTGGGACCACGAAATAGAGATATGA    1320
HEINZ       GAGACATACAAT-AAACCTTACTGAATTAATATAGGTGGGACCACGAAATAGAGATATGA    1318
            ************ ***********************************************

LA2093      TTTAATCAATAAATTAATGTTTATTGATTTAAAGAGTTTCGACACCGCAAAATTCATTTG    1320
E42         TTTAATCAATAAATTAATGTTTATTGATTTAAAGAGTTTCGACACCGCAAAATTCATTTG    1380
HEINZ       TTTAATCAATAAATTAATGTTTATTGATTTAAAGAGTTTCGACACCGCAAAATTCATTTG    1378
            ************************************************************

LA2093      GAATGAAATCTCAGTGAATATAAATGAATCCGCTTAAGAAAACGTCATTCATGAACTTGA    1380
E42         GAATGAAATCTCAGTGAATATAAATGAATCCGCTTAAGAAAACGTCATTCATGAACTTGA    1440
HEINZ       GAATGAAATCTCAGTGAATATAAATGAATCCGCTTAAGAAAACGTCATTCATGAACTTGA    1438
            ************************************************************

LA2093      TCATGATTAATGATATCGGTTACCGCAATAAAATGGACGTTAATAACTTGTTGAATGGGT    1440
E42         TCATGATTAATGATATCGGTTACCGCAATAAAATGGACGTTAATAACTTGTTGAATGGGT    1500
HEINZ       TCATGATTAATGATATCGGTTACCGCAATAAAATGGACGTTAATAACTTGTTGAATGGGT    1498
            ************************************************************

LA2093      GAAAATGATACACGTTCATAGGTCCAGAGCTCATAAGAAATTGTGGATATCATTAAAAAA    1500
E42         GAAAATGATACACGTTCATAGGTCCAGAGCTCATAAGAAATTGTGGATATCATTAAAAAA    1560
HEINZ       GAAAATGATACACGTTCATAGGTCCAGAGCTCATAAGAAATTGTGGATATCATTAAAAAA    1558
            ************************************************************

LA2093      AACATTTTGATGATAAAGCAGAAGATGATACAATACCTTTAGAACAAGCTACGGTCAAGA    1560
E42         AACATTTTGATGATAAAGCAGAAGATGATACAATACCTTTGGAACAAGCTACGGTCAAGA    1620
HEINZ       AACATTTTGATGATAAAGCAGAAGATGATACAATACCTTTAGAACAAGCTACGGTCAAGA    1618
            **************************************** *******************

LA2093      AACACTTATTACATTTAGAACTCTTCCTCAATTTTATGATGCAGTTCAGAAAGACAACAC    1620
E42         AACACTTATTACTTTTAGAACTCTTCCTCAATTTTATGATGCAGTTCAGAAAGACAACAC    1680
HEINZ       AACACTTATTACATTTAGAACTCTTCCTCAATTTTATGATGCAGTTCAGAAAGACAACAC    1678
            ************ ***********************************************

LA2093      AAACTTTTGAATGCAAGAAAATAAATTAGATGGGATTCAACAAGATTTAAATTTTAACC-    1679
```

```
E42         AAACTTTTGAATGCAAGAAAATAAATTAGATGGGATTCAACAAGATTTAAATTTTAACCA    1740
HEINZ       AAACTTTTGAATGCAAGAAAATAAATTAGATGGGATTCAACAAGATTTAAATTTTAACC-    1737
            ***********************************************************

LA2093      AAAAAAAATAGAACACAAGACAATCATGTTTCACTAAATTATCTTAGATATATTTGTACT    1739
E42         AAAAAAAATAGAACACAAGACAATCATGTTTCACTAAATTATCTTAGATATATTTGTACT    1800
HEINZ       AAAAAAAATAGAACACAAGACAATCATGTTTCACTAAATTATCTTAGATATATTTGTACT    1797
            ************************************************************

LA2093      TTTAATGAATTATTAA-TTTTATAATATTAATGGGACCATATATTTATATAGGGCTCTTC    1798
E42         TTTAATGAATTATTAA--TTTATAATATTAATGGGACCATATATTTATATAGGGCTCTTC    1858
HEINZ       TTTAATGAATTATTAATTTTTATAATATTAATGGGACCATATATTTATATAGGGCTCTTC    1857
            ****************  ******************************************

LA2093      AGAAAATATATTATCTTATTGAAACGAGTGATATTTTCCATTATATATCTTATTGAAATG    1858
E42         AGAAAATATATTATCTTATTGAAACGAGTGATATTTTCCATTATATATCTTATTGAAATG    1918
HEINZ       AGAAAATATATTATCTTATTGAAACGAGTGATATTTTCCATTATATATCTTATTGAAATG    1917
            ************************************************************

LA2093      AGTGATATTTTCCATTGGCCCAAGTCGAGACCAGAGAAAAATATTATCTTAGATATTATT    1918
E42         AGTGATATTTTCCAGTGGCCCAAGTCGAGACCAGAGAAAAATATTATCTTAGATATTATT    1978
HEINZ       AGTGATATTTTCCATTGGCCCAAGTCGAGACCAGAGAAAAATATTATCTTAGATATTATT    1977
            ************** *********************************************

LA2093      AATAAATCGAGTATTAATTTAGTGAAGTTTTACCGTAAATGACTATTCAATTGGGATATG    1978
E42         AATAAATCGAGTATTAATTTAGTGAAGTTTTACCGTAAATGACTATTCAATTGGGATATG    2038
HEINZ       AATAAATCGAGTATTAATTTAGTGAAGTTTTACCGTAAATGACTATTCAATTGGGATATG    2037
            ************************************************************

LA2093      TGAGCCCAACAACATACCCAGGGAAATCCCACTAGGTGGGGAATTGGGATATGTGAGCCC    2038
E42         TGAGCCCAACAACATACCCAGGGAAATCCCACTAGGTGGGGAATTGGGATATGTGAGCCC    2098
HEINZ       TGAGCCCAACAACATACCCAGGGAAATCCCACTAGGTGGGGAATTGGGATATGTGAGCCC    2097
            ************************************************************

LA2093      AAATCACACAATTAACTTCAGTTCTCCCATGAGATTTGCAGAAAGCTAGCGAAGTCCACA    2098
E42         AAATCACACAATTAACTTCAGTTCTCCCATGAGATTTGCAGAAAGCTAGCGAAGTCCACA    2158
HEINZ       AAATCACACAATTAACTTCAGTTCTCCCATGAGATTTGCAGAAAGCTAGCGAAGTTCACA    2157
            ******************************************************* ****

LA2093      CCTTACCGTGATGATAAAAAGCGAAAAAACTCAATGATAACTACAGTAATGCACTTTGAC    2158
E42         CCTTACCGTGATGATAAAAAGCGAAAAAACTCAATGATAACTACAGTAATGCACTTTGAC    2218
HEINZ       CCTTACCGTGATGATAAAAAGCGAAAAAACTCAATGATAACTACAGTAATGCACTTTGAC    2217
            ************************************************************

LA2093      CTTTATAGTGCTATCAATAAGTTCTTCTCAATTCCCAGACCAATTCAATTCCACATCCAC    2218
E42         CTTTATAGTGCTATCAATAAGTTCTTCTCAATTCCCAGACCAATTCAATTCCACATCCAC    2278
HEINZ       CTTTATAGTGCTATCAATAAGTTCTTCTCAATTCCCAGACCAATTCAATTCCACATCCAC    2277
            ************************************************************

LA2093      GGAACATCGAAAACTTTTAAATGATGAGAAGGAAAACTCTACCTGACATATATACCATTC    2278
E42         GGAACATCGAAAACTTTTAAATGATGAGAAGGAAAACTCTACCTGACATATATACCATTC    2338
HEINZ       GGAACATCGAAAACTTTTAAATGATGAGAAGGAAAACTCTACCTGACATATATACCATTC    2337
            ************************************************************

LA2093      AGTTGCGTTATAGATCCTGCTATCAGCACAAACATAGGCAACAGGATTATCTACCTGCGC    2338
E42         AGTTGCGTTATAGATCCTGCTATCAGCACAAACATAGGCAACAGGATTATCTACCTGCGC    2398
HEINZ       AGTTGCGTTATAGATCCTGCTATCAGCACAAACATAGGCAACAGGATTATCTACCTGCGC    2397
            ************************************************************

LA2093      AGATTGGTGGAGATCACTAGGGTAACATATACACAAGATGACAGATAGAGGAAATAACTG    2398
E42         AGATTGGTGGAGATCACTAGGGTAACATATACACAAGATGACAGATAGAGGAAATAACTG    2458
HEINZ       AGATTGGTGGAGATCACTAGGGTAACATATACACAAGATGACAGATAGAGGAAATAACTG    2457
            ************************************************************

LA2093      AAGAGAACAAAATATTTAAAGAAGAGATTCATCATGAAAGAAGTCTCCTCAAAAGCTTTT    2458
E42         AAGAGAACAAAATATTTAAAGAAGAGATTCATCATGAAAGAAGTCTCCTCAAAAGCTTTT    2518
HEINZ       AAGAGAACAAAATATTTAAAGAAGAGATTCATCATGAAAGAAGTCTCCTCAAAAGCTTTT    2517
            ************************************************************

LA2093      TTCTTTCTTGCTTTTTGATCAAATTAATTGATACCTTCAGTAGCATTCCGAAAAAGAATG    2518
E42         TTCTTTCTTGCTTTTTGATCAAATTAATTGATACCTTCAGTAGCATTCCGAAAAAGAATG    2578
HEINZ       TTCTTTCTAGCTTTTTGATCAAATTAATTGATACCTTCAGTAGCATTCCGAAAAAGAATG    2577
```

```
            ******** ***************************************************

LA2093      GATGTGAAGGCTGTTCAACCCATCCATCTCCGTCTTTTGCTGGATGAAAATCTTTGCATT    2578
E42         GATGTGAAGGCTGTTCAACCCATCCATCTCCGTCTTTTGCTGGATGAAAATCTTTGCATT    2638
HEINZ       GATGTGAAGGCTGTTCAACCCATCCATCTCCGTCTTTTGCTGGATGAAAATCTTTGCATT    2637
            ************************************************************

LA2093      CCTATGAATGGAAGAGAAAAATAAATGTCCTTGCAAGAACCTAAGTTGAAGAGGAGGACA    2638
E42         CCTATGAATGGAAGAGAAAAATAAATGTCCTTGCAAGAACCTAAGTTGAAGAGGAGGACA    2698
HEINZ       CCTATGAATGGAAGAGAAAAATAAATGTCCTTGCAAGAACCTAAGTTGAAGAGGAGGACA    2697
            ************************************************************

LA2093      ACCTATGTATGCTAAAGTGCACAGAATTCAAGAATTAATAACACGCTTGTACTGTGACTG    2698
E42         ACCTATGTATGCTAAAGTGCACAGAATTCAAGAATTAATAACACGCTTATACTGTGACTG    2758
HEINZ       ACCTATGTATGCTAAAGTGCACAGAATTCAAGAATTAATAACACGCTTATACTGTGACTG    2757
            ************************************************ ***********

LA2093      GAGGGAAACCTGGCACCATCTTGCTTTGGACTTAGTTTTCTTAGTGACAACCCAGACATG    2758
E42         GAGGGAAACCTGGCACCATCTTGCTTTGGACTTAGTTTTCTTAGTGACAACCCAGACATG    2818
HEINZ       GAGGGAAACCTGGCACCATCTTGCTTTGGACTTAGTTTTCTTAGTGACAACCCAGACATG    2817
            ************************************************************

LA2093      AAAGTTGCCACACTTTTTGCAAGTAATTCGCCGAGCTTCTCCAAGTGGATCCTCGCCCGC    2818
E42         AAAGTTGCCACACTTTTTGCAAGTAATTCGCCGAGCTTCTCCAAGTGGATCCTCGCCCGC    2878
HEINZ       AAAGTTGCCACACTTTTTGCAAGTAATTCGCCGAGCTTCTCCAAGTGGATCCTCGCCCGC    2877
            ************************************************************

LA2093      AGCTTCTGTCTGAGTGAAACCAGAGGTGAAGAATCCATAGCCTCTACCCTGAAATTATTT    2878
E42         AGCTTCTGTCTGAGTGAAACCAGAGGTGAAGAATCCATAGCCTCTACCCTGAAATTATTT    2938
HEINZ       AGCTTCTGTCTGAGTGAAACCAGAGGTGAAGAATCCATAGCCTCTACCCTGAAATTATTT    2937
            ************************************************************

LA2093      GAGATAAGGGTTAAGCATGACCCACAGTCAAGTAAAAGTATAATGAAACATTACAATCCC    2938
E42         GAGATAAGGGTTAAGCATGACCCACAGTCAAGTAAAAGTATAATGAAACATTACAATCCC    2998
HEINZ       GAGATAAGGGTTAAGCATGACCCACAGTCAAGTAAAAGTATAATGAAACATTACAATCCC    2997
            ************************************************************

LA2093      ACTATCAAGAGTACTTTCCAAAAATATTTCCTAATATAAGATGATACTTATCTAAAATAC    2998
E42         ACTATCAAGAGTACTTTCCAAAAATATTTCCTAATATAAGATGATACTTATCTAAAATAC    3058
HEINZ       ACTATCAAGAGTACTTTCCAAAAATATTTCCTAATATAAGATGATACTTATCTAAAATAC    3057
            ************************************************************

LA2093      AACTTGGGACCTAAAAATAAGAAGTTGACATAAAACAGCATTTAAAGGAAGACATTTACT    3058
E42         AACTTGGGACCTAAAAATAAGAAGTTGACATAAAACAGCATTTAAAGGAAGACATTTACT    3118
HEINZ       AACTTGGGACCTAAAAATAAGAAGTTGACATAAAACAGCATTTAAAGGAAGACATTTACT    3117
            ************************************************************

LA2093      AATAGATGTCATTATTTATATGATGAACCTATTGGAGCTAATTCATATACATTCGAGCCC    3118
E42         AATAGATGTCATTATTTATATGATGAACCTATTGGAGCTAATTCATATACATTCGAGCCC    3178
HEINZ       AATAGATGTCATTATTTATATGATGAACCTATTGGAGCTAATTCATATACATTCGAGCCC    3177
            ************************************************************

LA2093      AGCTGGCAAAAAGGCAAAAGAGTAAAGACTTAAAGACTGCATGAGAAACCCATTATTACT    3178
E42         AGCTGGCAAAAAGGCAAAAGAGTAAAGACTTAAAGACTGCATGAGAAACCCATTATTACT    3238
HEINZ       AGCTGGCAAAAAGGCAAAAGAGTAAAGACTTAAAGACTGCATGAGAAACCCATTATTACT    3237
            ************************************************************

LA2093      TAAAGTTGTTATTGTTAAGATACGTTTCACCAGATGGGTTCTTCAGCAAAAGGTGAGACT    3238
E42         TAAAGTTGTTATTGTTAAGATACGTTTCACCAGATGGGTTCTTCAGCAAAAGGTGAGACT    3298
HEINZ       TAAAGTTGTTATTGTTAAGATACGTTTCACCAGATGGGTTCTTCAGCAAAAGGTGAGACT    3297
            ************************************************************

LA2093      AATGTGACATCCCGCCACAATTAGAGCCTGATTCCGCGTCCGGAAAGGGGCAGATTGCTC    3298
E42         AATGTGACATCCCGCCACAATTAGAGCCTGATTCCGCGTCCGGAAAGGGGCAGATTGCTC    3358
HEINZ       AATGTGACATCCCGCCACAATTAGAGCCTGATTCCGCGTCCGGAAAGGGGCAGATTGCTC    3357
            ************************************************************

LA2093      TAGAGTTGTACGGAGAACTCTAGAACATTTTAGAACTCTCAAGAAGGCTAGAGAAGGTCC    3358
E42         TAGAGTTGTACGGAGAACTCTAGAACATTTTAGAACTCTCAAGAAGGCTAGAGAAGGTCC    3418
HEINZ       TAGAGTTGTACGGAGAACTCTAGAACATTTTAGAACTCTCAAGAAGGCTAGAGAAGGTCC    3417
            ************************************************************
```

```
LA2093      CAAAGTAGTGTAGAATTCTCTAGAAAAGGGACCAGAGTGTAAATATTTTAGGGACTTGTA    3418
E42         CAAAGTAGTGTAGAATTCTCTAGAAAAGGGACCAGAGTGTAAATATTTTAGGGACTTGTA    3478
HEINZ       CAAAGTAGTGTAGAATTCTCTAGAAAAGGGACCAGAGTGTAAATATTTTAGGGACTTGTA    3477
            ************************************************************

LA2093      AAGTAAATATTAATTGTACTTTAGTCCCTAGGAAGTAGTATAAATAGAGGTGCCCCTCAT    3478
E42         AAGTAAATATTAATTGTACTTTAGTCCCTAGGAAGTAGTATAAATAGAGGTGCCCCTCAT    3538
HEINZ       AAGTAAATATTAATTGTACTTTAGTCCCTAGGAAGTAGTATAAATAGAGGTGCCCCTCAT    3537
            ************************************************************

LA2093      TTGCAAAGGCACCAAGCAAGTTGTAAAGCAATCTTCCAAAGCAATACAAAAGTCTTCTTC    3538
E42         TTGCAAAGGCACCAAGCAAGTTGTAAAGCAATCTTCCAAAGCAATACAAAAGTCTTCTTC    3598
HEINZ       TTGCAAAGGCACCAAGCAAGTTGTAAAGCAATCTTCCAAAGCAATACAAAAGTCTTCTTC    3597
            ************************************************************

LA2093      AAAGCTCTCTTTCTTTTCTTTCTAAGTCTTTCTTTGCATTTTCTTAGCGTTTCGAGTCTT    3598
E42         AAAGCTCTCTTTCTTTTCTTTCTAAGTCTTTCTTTGCATTTTCTTAGCGTTTCGAGTCTT    3658
HEINZ       AAAGCCCTCTTTCTTTTCTTTCTAAGTCTTTCTTTGCATTTTCTTAGCGTTTCGAGTCTT    3657
            ***** ******************************************************

LA2093      AAAGGCTTACTTGAGCTTACAAATCGTGAAAGATTCGTGAGTAAGTTGTCAAGTGCTGCA    3658
E42         AAAGGCTTACTTGAGCTTACAAATCGTGAAAGATTCGTGAGTAAGTCGTCAAGTGCTGCA    3718
HEINZ       AAAGGCTTACTTGAGCTTACAAATCGTGAAAGATTCGTGAGTAAGTTGTCAAGTGCTGCA    3717
            ********************************************** *************

LA2093      CGGAGTCTTAGCAAATACTCTAAGTCCGTGACAAAGTGGTATTAGAGCGAGGTTTGATAC    3718
E42         CGGAGTCTTAGCAAATACTCTAAGTCCGTGACAAAGTGGTATTAGAGCGAGGTTTGATAC    3778
HEINZ       CGGAGTCTTAGCAAATACTCTAAGTCCGTGACAAAGTGGTATTAGAGCGAGGTTTGATAC    3777
            ************************************************************

LA2093      TAAGGGAATGGCTACCGAAGGAGACGTAAACGGCACTAGCACCACTAACGTCCGTCTAGA    3778
E42         TAAGGGAATGGCTACCGAAGGAGACGTAAACGGCACTAGCACCACTAATGTCCGTCTAGA    3838
HEINZ       TAAGGGAATGGCTACCGAAGGAGACGTAAACGGCACTAGCACCACTAACGTCCGTCTAGA    3837
            ************************************************ ***********

LA2093      TGGTGGTAAGAAGAATCGAAAGGGGAAGAACATCAAAAGGATAATGAGGAGGTGTTGCCC    3838
E42         TGGTGGTAAGAAGAATCGAAAGGGGAAGAACATCAAAAGGATAATGAGGAGGTGTTGCCC    3898
HEINZ       TGGTGGTAAGAAGAATCGAAAGGGGAAGAACATCAAAAGGATAATGAGGAGGTGTTGCCC    3897
            ************************************************************

LA2093      GAACTCACACCTAGCGAGGCGCTAACATCTCATCCACCCTCGACTGCCGAGGCAAGTGAC    3898
E42         GAACTCACACCTAGCGAGGCGCTAACATCTCATCCACCCTCGACTGCCGAGGCAAGTGAT    3958
HEINZ       GAACTCACACCTAGCGAGGCGCTAACATCTCATCCACCCTCGACTGCCGAGGCAAGTGAC    3957
            ***********************************************************

LA2093      GAAGATGCCATAGACGTCACCGCTGGAGAAGAATGGGTCGCAAGGGTGGAAGTGATGAGA    3958
E42         GAAGATGCCATAGACGTCACCGCTGGAGAAGAATGGGTCGCAAGGGTGGAAGTGATGAGA    4018
HEINZ       GAAGATGCCATAGACGTCACCGCTGGAGAAGAATGGGTCGCAAGGGTGGAAGTGATGAGA    4017
            ************************************************************

LA2093      CAGGTCGTCGAGATCTTATGCCCGCGCATGAACATGGAAGACGGCAAGTTCAAGACCCTT    4018
E42         CAGGTCGTCGAGATCTTATGCCCGCGCATGAACATGGAAGACGGCAAGTTCAAGACCCTT    4078
HEINZ       CAGGTCGTCGAGATCTTATGCCCGCGCATGAACATGGAAGACGGCAAGTTCAAGACCCTT    4077
            ************************************************************

LA2093      GAGGAGACCAAGAACATCTGTAAGGAGTTGGAAGGTCGCCAGCGGGCCGAGTTCGAGATG    4078
E42         GAGGAGACCAAGAACATCTGTAAGGAGTTGGAAGGTCGCCAGCGGGCCGAGTTCGAGATG    4138
HEINZ       GAGGAGACCAAGAACATCTGTAAGGAGTTGGAAGGTCGCCAGCGGGCCGAGTTCGAGATG    4137
            ************************************************************

LA2093      GAGGAGGCCATCACTTCCTTGGAGTGTCGGCTTATGGATGTGCTTAACACGAAGGAGACC    4138
E42         GAGGAGGCCATCACTTCCTTGGAGTGTCGGCTTATGGATGTGCTTAACACGAAGGAGACC    4198
HEINZ       GAGGAGGCCATCACTTCCTTGGAGTGTCGGCTTATGGATGTGCTTAACACGAAGGAGACC    4197
            ************************************************************

LA2093      ATCACTTCCTTGGAGAGTCAGCTTACAGAAGCACTTAGCACGATCGAGACCTTGAAGGCC    4198
E42         ATCACTTCCTTGGAGAGTCAGCTTACAGAAGCACTTAGCACGATCGAGACCTTGAAGGCC    4258
HEINZ       ATCACTTCCTTGGAGAGTCAGCTTACAGAAGCACTTAGCACGATCGAGACCTTGAAGGCC    4257
            ************************************************************

LA2093      GAAATAAAAGCACTCAAGGAAAGCGTAGATGTTGGAGGATTTGCATCACCTGACCGCGAT    4258
E42         GAAATAAAAGCACTCAAGGAAGGCGTAGATGTTGGAGGATTTGCATCACCTGACCGCGAT    4318
```

```
HEINZ         GAAATAAAAGCACTCAAGGAAGGCGTAGATGTTGGAGGATTTGCATCACCTGACCGCGAT    4317
              ********************* **************************************

LA2093        AGGGAGACCATGGTCGAGGCTCCCAACCCACCTATATTCAAGGGTGTCCGTGACGCTCAA    4318
E42           AGGGAGACCATGGTCGAGGCTCCCAACCCACCTATATTCAAGGGTGTCCGTGACGCTCAA    4378
HEINZ         AGGGAGACCATGGTCGAGGCTCCCAACCCACCTATATTCAAGGGTGTCCGTGACGCTCAA    4377
              ************************************************************

LA2093        GAGGTGGAAAACTTTCTATGGCACTTGGAGAATTGCTTCAAGTGTAGTCGAGTGAGAAGC    4378
E42           GAGGTGGAAAACTTTCTATGGCACTTGGAGAATTGCTTCAAGTGTAGTCGAGTGAGAAGC    4438
HEINZ         GAGGTGGAAAACTTTCTATGGCATTTGGAGAATTGCTTCAAGTGTAGTCGAGTGAGAAGC    4437
              *********************** ************************************

LA2093        GACGAGAACAAGATCAACACTGTCGTGTTGTACTTGTCCGAGATGGCTATGCTATGGTTG    4438
E42           GACGAGAACAAGATCAACACTGTCGTGTTGTACTTGTCCGAGATGGCTATGCTATGGTTG    4498
HEINZ         GACGAGAACAAGATCAACACTGTCGTGTTGTACTTGTCCGAGATGGCTATGCTATGGTTG    4497
              ************************************************************

LA2093        AGGCGCAAAGAGGTCGAGATCGGGAAGGGGACGCGCACCATCAACACGTGGGAGCAATTC    4498
E42           AGGCGCAAAGAGGTCGAGATCGGGAAGGGGACGCACACCATCAACACGTGGGAGCAATTC    4558
HEINZ         AGGCGCAAAGAGGTCGAGATCGGGAAGGGGACGCGCACCATCAACACGTGGGAGCAATTC    4557
              ********************************** *************************

LA2093        CGCGAAGAATTCAAGAAAGCCTTCTTCCCTAACAACGTCGTTTACGAGGTTAAGCGCAAG    4558
E42           CGCGAAGAATTCAAGAAAGCCTTCTTCCCTAACAACGTCGTTTACGAGGTTAAGCGCAAG    4618
HEINZ         CGCGAAGAATTCAAGAAAGCCTTCTTCCCTAACAACGTCGTTTACGAGGTTAAGCGCAAG    4617
              ************************************************************

LA2093        TTCTGGGAATTGAGGCAAACGAGAAGCATTTGGGCGAATGTGAAGGAGTTCACAACTCTA    4618
E42           TTCCGGGAATTGAGGCAAACGAGAAGCATTTGGGCGAATGTGAAGGAGTTCACAACTCTA    4678
HEINZ         TTCCGGGAATTGAGGCAAACGAGAAGCATTTGGGCGAATGTGAAGGAGTTCACAACTCTA    4677
              *** ********************************************************

LA2093        ACGCTTCAAATTTCCAACCTCACGGACGAAGACATGTTGTTCCACTTCATGGACGCACTG    4678
E42           ACGCTTCAAATTTCCAACCTCACGGACGAAGACATGTTGTTCCACTTCATGGACGCACTG    4738
HEINZ         ACGCTTCAAATTTCCAACCTCACGGACGAAGACATGTTGTTCCACTTCATGGACGCACTG    4737
              ************************************************************

LA2093        CAGAACTGGGCCAAGACGGTGTTGGAGCGGCGTCAGGTCAAAACCATAGACGAAGCCATC    4738
E42           CAGAACTGGGCCAAGACGGTGTTGGAGCGGCGTCAGGTCAAAACCATAGACGAAGCCATC    4798
HEINZ         CAGAACTGGGCCAAGACGGTGTTGGAGCGGCATCAGGTCAAAACCATAGACGAAGCCATC    4797
              ******************************* ****************************

LA2093        ACACAAGCCGAGTCCTCGACGGACTTCAAGCATGAACGACATGACAAGGCGAAGGGCAGA    4798
E42           ACACAAGCCGAGTCCTCGACGGACTTCAAGCATGAACGACATGACAAGGCGAAGGGCAGA    4858
HEINZ         ACACAAGCCGAGTCCTCGACGGACTTCAAGCATGAACGACATGACAAGGCGAAGGGCAGA    4857
              ************************************************************

LA2093        AATGCAAGAAGTTGTCATGCCAAAGGTGGGGAGACCGTGGCCGAGGCAAGGAGCAGCAGG    4858
E42           AATGCAAGAAGTTGTCATGCCAAAGGTGGGGAGACCGTGGCCGAGGCAAGGAGTAGCAGG    4918
HEINZ         AATGCAAGAAGTTGTCATGCCAAAGGTGGGGAGACCGTGGCCGAGGCAAGGAGCAGCAGG    4917
              ***************************************************** ******

LA2093        CACACCCCAAGCAACACGACCCCCACAAGCCGGACATCAGGCGATTCGTGCGCAAGAATT    4918
E42           CACACCCCAAGCAACACGACCCCCACAAGCCGGACATCAGGCGATTCGTGCGCAAGAATT    4978
HEINZ         CACACCCCAAGCAACACGACCCCCACAAGCCGGACATCAGGCGATTCGTGCGCAAGAATT    4977
              ************************************************************

LA2093        ATACGGAGAAACGGGCGCAGACCAGCTAAAGAGATGGGTACTACATATGCGGTAGACCGC    4978
E42           ATACGGAGAAACGGGCGCAGACCAGCTAAAGAGATGGGTACTACATATGCGGTAGACCGC    5038
HEINZ         ATACGGAGAAACGGGCGCAGACCAGCTAAAGAGATGGGTACTACATATGCGGTAGACCGC    5037
              ************************************************************

LA2093        ACATCTATGCCAGGTGCCCCGAAATGAAGAACCTCGGTGCCATCCTACGCAAACGGAAGG    5038
E42           ACGTCTATGCCAGGTGCCCCGAAATGAAGAACCTCGGTGCCATCCTACGCGAACGGAAGG    5098
HEINZ         ACATCTATGCCAGGTGCCCCGAAATGAAGAACCTCGGTGCCATCCTACGCAAACGGAAGG    5097
              ** *********************************************** *********

LA2093        AGAAGTACGCACAAGATCAAGGACATGATGCGGGCACGACGCAGTTGGGCATGGTTGGAT    5098
E42           AGAAGTACGCACAAGATCAAGGACATGATGCGGGCACGACGCAGTTGGGCATGGTTGGAT    5158
HEINZ         AGAAGTACGCACAAGATCAAGGACATGATGCGGGCACGACGCAGTTGGGCATGGTTGGAT    5157
              ************************************************************
```

```
LA2093      TGTGTGGTGCGATAGCCAAGAGAACCGAGAAGCTAGGAGACTTCAGCACGCAGTATGTAG    5158
E42         TGTGTGGTGCGATAGCCAAGAGAACCGAGAAGCCAGGAGACTTCAGCACGCAGTATGTAG    5218
HEINZ       TGTGTGGTGCGATAGCCAAGAGAACCGAGAAGCCAGGAGACTTCAGCACGCAGTATGTAG    5217
            ********************************* **************************

LA2093      ACATCTCCATAAATGGAAGACCAGCTCGTGCCATGGTAGACTCCGGAGCAGAAGTCAACA    5218
E42         ACATCTCCATAAATGGAAGACCAGCTCGTGCCATGGTAGACTCCGGAGCAGAAGTCAACA    5278
HEINZ       ACATCTCCATAAATGGAAGACCAGCTCGTGCCATGGTAGACTCCGGAGCAGAAGTCAACA    5277
            ************************************************************

LA2093      TCATGACCAAGACGGCAGCAGAGAGGTTAAGGCTGAACTTTGTGCCAAGCAATACCCACC    5278
E42         TCATGACCAAGACGGCAGCAGAGAGGTTAAGGCTGAACTTTGTGCCAAGCAATACCCACC    5338
HEINZ       TCATGACCAAGACGGCAGCAGAGAGGTTAAGGCTGAACTTTGTGCCAAGCAATACCCACC    5337
            ************************************************************

LA2093      TCAAGACAGTTAATGCCCCATCGACTCCCGTATGCGGGG-TCGCCCAAGGAGTGAGCATC    5337
E42         TCAAGACAGTTAATGCCCCATCGACTCCCGTATGCGGGGTTCGCCCAAGGAGTGAGCATC    5398
HEINZ       TCAAGACAGTTAATGCCCCATCGACTCCCGTATGCGGGG-TCGCCCAAGGAGTGAGCATC    5396
            *************************************** ********************

LA2093      ACGTTGGGAAAGTGGCAAGGTAAAACAAACTTTACCTCGCTCATTTAGACATAATTGATA    5397
E42         ACGTTGGGAAAGTGGCAAGGTAAAACAAACTTTACCTCGCTCATTTAGACATAATTGATA    5458
HEINZ       ACGTTGGGAAAGTGGCAAGGTAAAACAAACTTTACCTCGCTCATTTAGACATAATTGATA    5456
            ************************************************************

LA2093      TCATTCTCAGGCAAGAGTTCTTCCAGCGCTGCCACACGATGATCGACCCCTACCTCCAAC    5457
E42         TCATTCTCAGGCAAGAGTTCTTCCAGCGCTGCCACACGATGATCGACCCCTACCTCCAAC    5518
HEINZ       TCATTCTCAGGCAAGAGTTCTTCCAGCGCTGCCACACGATGATCGACCCCTACCTCCAAC    5516
            ************************************************************

LA2093      AACTCATGGTGATGAAACAGGAAGGGTCATGCATGGTACCTCTAGTCAAGGTACCAAAGA    5517
E42         AACTCATGGTGATGAAACAGGAAGGGTCATGCATGGTACCTCTAGTCAAGGTACCAAAGA    5578
HEINZ       AACTCATGGTGATGAAACAGGAAGGGTCATGCGTGGTACCTCTAGTCAAGGTACCAAAGA    5576
            ******************************** ***************************

LA2093      AGGAAGGACACACCCACTTATCGGCCATGCGGATCGTGAAAGGCCTAAAGGAAGGAGAAC    5577
E42         AGGAAGGACACACCCACTTATCGGCCATGAGGATCGTGAAAGGCCTAAAGGAAGGAGAAC    5638
HEINZ       AGGAAGGACACACCCACTTATCGGCCATGCGGATCGTGAAAGGCCTAAAGGAAGGAGAAC    5636
            ***************************** ******************************

LA2093      AGACGTTCTTAGCTACCATTGCAAGTTCGAAGGAAGACAATGGTACTATAGAGTCCTTGC    5637
E42         AGACGTTCTTAGCTACCATTGCAAGTTCGAAGGAAGACAATGGTACTATAGAGTCCTTGC    5698
HEINZ       AGACGTTCTTAGCTACCATTGCAAGTTCGAAGGAAGACAATGGTACTATAGAGTCCTTGC    5696
            ************************************************************

LA2093      CACCGATCATAGAGACTGTCCTTGAAGAGAACAAGAACGTAATGCCGGACGAGCTGCCGA    5697
E42         CACCGATCATAGAGACTGTCCTTGAAGAGAACAAGAACGTGATGCCGGACGAGCTGCCGA    5758
HEINZ       CACCGATCATAGAGACTGTCCTTGAAGAGAACAAGAACGTGATGCCGGACGAGCTGCCGA    5756
            **************************************** *******************

LA2093      AGACTACCTCCGAGGCGCGAGGTAGATCATAAGATAGAGTTGGAGGTCGAAGCCAAGCCA    5757
E42         AGACTACCTCCGAGGCGCGAGGTAGATCATAAGATAGAGTTGGAGGTCGAAGCCAAGCCA    5818
HEINZ       AGACTACCTCCGAGGCGCGAGGTAGATCATAAGATAGAGTTGGAGGTCGAAGCCAAGCCA    5816
            ************************************************************

LA2093      CCCGCACATGCACCTTACCGTATGGCTCCACCCGAGTTAGAGGAGCTAAGGAAGCAATTG    5817
E42         CCCGCACATGCACCTTACCGTATGGCTCCACCCGAGTTAGAGGAGCTAAGGAAGCAATTG    5878
HEINZ       CCCGCACATGCACCTTACCGTATGGCTCCACCCGAGTTAGAGGAGCTAAGGAAGCAATTG    5876
            ************************************************************

LA2093      AAGGAGCTCCTCGAGGCCGGTCACATTCGTCCATCCAAGGCACCTTATGGCGCGCCAGTG    5877
E42         AAGGAGCTCCTCGAGGCCGGTCACATTCGTCCATCCAAGGCACCTTATGGCGCGCCAGTG    5938
HEINZ       AAGGAGCTCCTCGAGGCCGGTCACATTCGTCCATCCAAGGCACCTTATGGCGCGCCAGTG    5936
            ************************************************************

LA2093      TTGTTCCAAAAGAAAAAAGACGCATCGTTGCGCTTATGCATCGATTATCGTGCGCTCAAT    5937
E42         TTGTTCCAAAAGAAAAAAGACGCATCGTTGCGCTTATGCGTCGATTATCGGGCGCTCAAT    5998
HEINZ       TTGTTCCAAAAGAAAAAAGACGCATCGTTGCGCTTATGCATCGATTATCGTGCGCTCAAT    5996
            *************************************** ********** *********

LA2093      AAGGTCACAATTAAGAACAAATATCTTATCCCGCTAATTGCAGATTTGTTTGATAGACTT    5997
```

```
E42        AAGGTCACAATTAAGAACAAATATCTTATCCCGCTAATTGCAGATTTGTTTGATAGACTT    6058
HEINZ      AAGGTCACAATTAAGAACAAATATCTTATCCCGCTAATTGCAGATTTGTTTGATAGACTT    6056
           ************************************************************

LA2093     GGGCAGGCCAAGTACTTCACCAAGATGGACCTCTGGAAAGGCTACTACCAAGTGCGCATC    6057
E42        GGGCAGGCCAAGTACTTCACCAAGATGGACCTCCGGAAAGGCTACTACCAAGTGCGCATC    6118
HEINZ      GGGCAGGCCAAGTACTTCACCAAGATGGACCTCTGGAAAGGCTACTACCAAGTGCACATC    6116
           ********************************* ********************* ****

LA2093     GCAGAGGGAGATGAGCCAAAGACAGCGTGCATGACTAGATACGGAGCATAAGAGTGGTTG    6117
E42        GCAGAGGGAGATGAGCCAAAGACAGCGTGCATGACTAGATACGGAGCATAAGAGTGGTTG    6178
HEINZ      GCAGAGGGAGATGAGCCAAAGACAGCGTGCATGACTAGATACGGAGCATAAGAGTGGTTG    6176
           ************************************************************

LA2093     GTGATGCCCTTTGGCTTAACAAACGCACCCGCCACCTTTTGCACGCTGATGAACGAAATC    6177
E42        GTGATGCCCTTTGGCTTAACAAACGCACCCGCCACCTTTTGCACGCTGATGAACGAAATC    6238
HEINZ      GTGATGCCCTTTGGCTTAACAAACGCACCCGCCACCTTTTGCACGCTGATGAACGAAATC    6236
           ************************************************************

LA2093     CTTCATCCCTACTTGGACAAGTTCTTAGT-GGTGTACTTGGATGACAGTCATATATAGCA    6236
E42        CTTCATCCCTACTTGGACAAGTTCTTAGT-GGTGTACTTGGATGACAGTCATATATAGCA    6297
HEINZ      CTTCATCCCTACTTGGACAAATTCTTAGTGGGTGTACTTGGATGACAGTCATATATAGCA    6296
           ******************** ******** ******************************

LA2093     ACACCTTAGAAGAACACGTGGAGCACCTGAATAAAGTCTTTTTAGTCTTATGAGAGAACC    6296
E42        ACACCTTAGAAGAACACGTGGAGCACCTGAATAAAGTCTTTTTAGTCTTATGAGAGAACC    6357
HEINZ      ACACCTTAGAAGAACACGTGGAGCACCTGAATAAAGTCTTTTTAGTCTTATGAGAGAACC    6356
           ************************************************************

LA2093     AGCTTTACGTCAAGCGGGAGAAGTGCGAGTTCGCCCAGCCCAAGGTGTACTTCTTAGGCC    6356
E42        AGCTTTACGTCAAGCGGGAGAAGTGCGAGTTCGCCCAGCCCAAGGTGTACTTCTTAGGCC    6417
HEINZ      AGCTTTACGTCAAGCGGGAGAAGTGCGAGTTTGCCCAGCCCAAGGTGTACTTCTTAGGCC    6416
           ******************************* ****************************

LA2093     ATGTTATCAGCCACGGCGAACTACGGATGGACGAGTCGAAGATTAGGGCAATCCAAGAAT    6416
E42        ATGTTATCAGCCACGGCGAACTACGGATGGACGAGTCGAAGATTAGGGCAATCCAAGAAT    6477
HEINZ      ATGTTATCAGCCACGGCGAACTACGGATGGACGAGTCGAAGATTAGGGCAATCCAAGAAT    6476
           ************************************************************

LA2093     GGGAGGCACCAACGAAGGTGATCGAGCTGCGATCATTCCTTGGACTTGCGAACTACTATC    6476
E42        GGGAGGCACCAACGAAGGTGATCGAGCTGCGATCATTCCTTGGACTTGCGAACTACTATC    6537
HEINZ      GGGAGGCACCAACGAAGGTGATCGAGCTGCGATCATTCCTTGGACTTGCGAACTACTATC    6536
           ************************************************************

LA2093     GCAGGTTCATCAGTGGCTACTTCGCTAAAGCCGCTCCACTGACTGAGTTGTTAAAGAAGA    6536
E42        GCAGGTTCATCAGTGGCTACTTCGCTAAAGCCGCTCCACTGACTGAGTTGTTAAAGAAGA    6597
HEINZ      GCAGGTTCATCAGTGGCTACTTCGCTAAAGCCGCTCCACTGACTGAGTTGTTAAAGAAGA    6596
           ************************************************************

LA2093     ATAAGCCATGGGTTTAGAGCAAGGAGTGACAAAAGGCATTAAGGCCGCAGTGACAGAAGA    6596
E42        ATAAGCCATGGGTTTAGAGCAAGGAGTGACAAAAGGCGTTAAGGCCGCAGTGACAGAAGA    6657
HEINZ      ATAAGCCATGGGTTTAGAGCAAGGAGTGACAAAAGGCGTTAAGGCCGCAGTGACAGAAGA    6656
           ************************************* **********************

LA2093     GTCGGTCTTGACGTTACCTGACTTCTCCAAGACCTTCGAAATACATACTGATGCCTCAGA    6656
E42        GTCGATCTTGACGTTACCTGACTTCTCCAAGGCCTTCGAAATACATACGGATGCCTCAGA    6717
HEINZ      GTCGGTCTTGACGTTACCTGACTTCTCCAAGACCTTCGAAATACATACGGATGCCTCAGA    6716
           **** ************************** **************** ***********

LA2093     CTTTGCCATTGGTGGGGGAGTATCGCATTCGAAAGCCGCAAGTTAAACGAGACTGAACGG    6716
E42        CTTTGCCATTGGTGGGGGAGTATCGCATTCGAAAGCCGCAAGTTAAACGAGACCGAACGG    6777
HEINZ      CTTTGCCATTGGTGGGGGAGTATCGCATTCGAAAGCCGCAAGTTAAACGAGACCGAACGG    6776
           ***************************************************** ******

LA2093     TATTATACAGTGAAGGAAAAAGAGATGACGGCCATTGTACATTGCCTACACACGTGGAGA    6776
E42        TATTATACGGTGAAGGAAAAAGAGATGACGGCCATTGTACATTGCCTACACACGTGGAGA    6837
HEINZ      TATTATACGGTGAAGGAAAAAGAGATGACGGCCATTGTACATTGCCTACACGCGTGGAGA    6836
           ******** ****************************************** ********

LA2093     CACTACTTATTAGACCGACAACGTTGCCACTAGCTACTTCCAATCTCAAAAGAAAATCTC    6836
E42        CACTACTTATTAGACCGACAACGTTGCCACTAGCTACTTCCAATCTCAAAAGAAGATCTC    6897
HEINZ      CACTACTTATTAGACCGACAACGTTGCCACTAGCTACTTCCAATCTCAAAAGAAGATCTC    6896
```

```
              ****************************************************** *****

LA2093        CCCAACGCAAGCTAGATGACAAGACTTCTTGGCAGAATATGACTACGTCCTGGAGTAAAG    6896
E42           CCCAACGCAAGCTAGATGACAAGACTTCTTGGCAGAATATGACTACGTCCTGGAGTAAAG    6957
HEINZ         CCCAACGCAAGCTAGATGACAAGACTTCTTGGCAGAATATGACTACGTCCTGGAGTAAAG    6956
              ************************************************************

LA2093        CCAGGAAGGGGCAACATCGTAGCCGACGCACTAAGTAGGAAGGCCGAGCTTGCTGCCATC    6956
E42           CCAGGAAGGGGCAACATCGTAGCCGACGCACTAAGTAGGAAGGCCGAGCTTGCTGCCATC    7017
HEINZ         CCAGGAAGGGGCAACATCGTAGCCGACGCACTAAGTAGGAAGGCCGAGCTTGCTGCCATC    7016
              ************************************************************

LA2093        ACTACAGCTCACTGTGACTTTCAAGATGCAATCAAGGATGGCATGCAACACGATCCAGAG    7016
E42           ACTACAGCTCACTGTGACATTCAAGATGCAATCAAGGATGGCATGCAACACGATCCAGAG    7077
HEINZ         ACTACAGCTCACTGTGACTTTCAAGATGCAATCAAGGATGGCATGCAACACGATCCAGAG    7076
              ****************** *****************************************

LA2093        GCCAAGAAGCTGATGGAGTTAGCTGTCCAAGGAAAAACCAGGCGTTTTTGGGTAGAAAAT    7076
E42           GCCAAGAAGCTGATGGAGTTAGCTGTCCAAGGAAAAACCAGGCGTTTTTGGGTAGAAAAT    7137
HEINZ         GCCAAGAAGCTGATGGAGTTAGCTGTCCAAGGAAAAACCAGGCGTTTTTGGGTAGAAAAT    7136
              ************************************************************

LA2093        GGCTTCTTGCTCAATACCGGTCGAAGGGTTTGTGTACCCAAGTTCGTGTCTATCAGGCGG    7136
E42           GGCTTCTTGCTCAATACCGATCGAAGGGTTTGTGTACCCAAGTTCGTGTCTATCAGGCGG    7197
HEINZ         GGCTTCTTGCTCAATACCGGTCGAAGGGTTTGTGTACCCAAGTTCGTGTCTATCAGGCGG    7196
              ******************* ****************************************

LA2093        CGCATCATAAAGGAAAGCCACGACACACCGTGAGCTGGGCATCCGGGACAGCGACGTAAG    7196
E42           CGCATCATAAAGGAAAGCCACGACACACCGTGAGCTGGGCATCCGGGACAGCGACGTAAG    7257
HEINZ         CGCATCATAAAGGAAAGCCACGACACACCGTGAGCTGGGCATCCGGGACAGTGACGTAAG    7256
              *************************************************** ********

LA2093        AGGGCGCTGATCGAGGCTATTTAATTCTGCCACGCATGCGGGACGATATATTGTGCTATG    7256
E42           AGGGCGCTGATCGAGGCTATTTAATTCTGCCACGCATGCGGGACGATATATTGTGCTATG    7317
HEINZ         AGGGCGCTGATCGAGGCTATTTAATTCTGCCACGCATGCGGGACGATATATTGTGCTATG    7316
              ************************************************************

LA2093        TGCAGACTTGTCTTATGTGCCAACAAGACAAGGTGGAGTAAAGGCAACCGGGAGGGTTAC    7316
E42           TGCAGACTTGTCTTATGTGCCAACAAGACAAGGTGGAGTAAAGGCAACCGGGAGGGTTAC    7377
HEINZ         TGCAGACTTGTCTTATGTGCCAACAAGACAAGGTGGAGTAAAGGCAACCGGGAGGGTTAC    7376
              ************************************************************

LA2093        TAGAACCACTACCCGTAGCAGAACGCCCATGAGAGAGCGTGACCATGGACTTCATCACTT    7376
E42           TAGAACCACTACCCGTAGCAGAACGCCCATGAGAGAGCGTGACCATGGACTTCATCACTT    7437
HEINZ         TAGAACCACTACCCGTAGCAGAACGCCCATGAGAGAGCGTGACCATGGACTTCATCACTT    7436
              ************************************************************

LA2093        CCTTGCCGAAGTCCGACGGTTTTGGTACTATCATGGTCGTGGTGGACAGGTTTTCAAAGT    7436
E42           CCTTGCCGAAGTCCGACGGTTTTGGTACTATCATGGTCGTGGTGGACAGGTTTTCAAAGT    7497
HEINZ         CCTTGCCGAAGTCCGACGGTTTTGGTACTATCATGGTCGTGGTGGACAGGTTTTCAAAGT    7496
              ************************************************************

LA2093        ATGCTACCTTTTTGCCCGCCACAGCCGGTTGCACGGCAAAAAAGGCTGCTCGATTGTTCT    7496
E42           ATGCTACCTTTTTGCCCGCCACAGCCGGTTGCACGGCAAAAAAGGCTGCTCGATTGTTCT    7557
HEINZ         ATGCTACCTTTTTGCCCGCCACAGCCGGTTGCACGGCAAAAAAGGCTGCTCGATTGTTCT    7556
              ************************************************************

LA2093        TTAAGAACGTGGTGAAATATTGGGGGCTGCTGAGACATTCATTAGCAATCAAGACCCCCG    7556
E42           TTAAGAACGTGGTGAAATATTGGGGGTTGCTGAGACATTCATTAGCAATCAAGACCCCCG    7617
HEINZ         TTAAGAACGTGGTGAAATATTGGGGGCTGCTGAGACATTCATTAGCAATCAAGACCCCCG    7616
              ************************** *********************************

LA2093        CTTTACCGGGTACTTTTGGAGAGTTGTTCCTGATACTGGGCACGGAGCTTCACTTCTCCA    7616
E42           CTTTACCGGGTACTTTTGGAGAGTTGTTCCTGATACTGGGCACGGAGCTTCACTTCTCCA    7677
HEINZ         CTTTACCGGGTACTTTTGGAGAGTTGTTCCTGATACTGGGCACGGAGCTTCACTTCTCCA    7676
              ************************************************************

LA2093        CAAGTTTCCACCAG------------CAGACCAACGGCCAGACCAAACGCGTCAACATCC    7664
E42           CAAGTTTCCACCAG------------CAGACCAACGGCCAGACCAAACGCGTCAACATCC    7725
HEINZ         CAAGTTTCCACCAGCAGACCAACGGCCAGACCAACGGCCAGACCAAACGCGTCAACATCC    7736
              **************            **********************************
```

```
LA2093      TACTAGAGTGCTATCTAAGGCACTATGTTAGTGCGTATCAAAAGGATTGGGCCAAACTCC    7724
E42         TACTAGAGTGCTATCTAAGGCACTATGTTAGTGCGCATCAAAAGGATTGGGCCAAACTCC    7785
HEINZ       TACTAGAGTGCTATCTAAGGCACTATGTTAGTGCGTATCAAAAGGATTGGGCCAAACTCC    7796
            *********************************** ************************

LA2093      TAGATATGGTGCAGTTTTCCTACAATTTGCAAAGGAGCGAGTCCACCGAGCGCACACCGT    7784
E42         TAGATATGGTGCAGTTTTCCTACAATTTGCAAAGGAGCGAGTCCACCGAGCGCACACCGT    7845
HEINZ       TAGATATGGTGCAGTTTTCCTACAATTTGCAAAGGAGCGAGTCCACCGAGCGCACACCGT    7856
            ************************************************************

LA2093      TTGAGTTGGCGATAGGCCAACAACCCCAAACTCCGCATTGGTTGCCGACTGCTTTTGAGG    7844
E42         TTGAGTTGGCGATAGGCCAACAACCCCAAACTCCGCATTGGTTGCCGACTGCTTTTGAGG    7905
HEINZ       TTGAGTTGGCGATAGGCCAACAACCCCAAACTCCGCATTGGTTGCCGACTGCTTTTGAGG    7916
            ************************************************************

LA2093      GAAAAAGTTTGGGTGCCTACCGTCTTGCCAAGGGATGGAGGAGCAGTTTAACACTGCCA    7904
E42         GAAAAAGTTTGGGTGCCTACCGTCTTGCCAAGGGATGGAGGAGCAGTTTGACACTGCCA    7965
HEINZ       GAAAAAGTTTGGGTGCCTACCGTCTTGCCAAGGGATGGAGGAGCAGTTTGACACTGCCA    7976
            ************************************************* **********

LA2093      AGTCCTACTTGGACAAGGCAGCCAAAAAGATGAAGAAGTTCGCCGACCGCAAGCGTCGTC    7964
E42         AGTCCTACTTGGACAAGGCAGCCAAAAAGATGAAGAAGTTCGCCGACCGCAAGCGTCGTC    8025
HEINZ       AGTCCTACTTGGACAAGGCAGCCAAAAAGATGAAGAAGTTCGCCGACCGCAAGCGTCGTC    8036
            ************************************************************

LA2093      CCACGCACTATAAAGAAGGCAACATGGTCTTGGTAAAATTCAATCCAAGACAGTTCAAGG    8024
E42         CCACGCACTATAAAGAAGGCAACATGGTCTTGGTAAAATTCAATCCAAGACAGTTCAAGG    8085
HEINZ       CCACGCACTATAAAGAAGGCAACATGGTCTTGGTAAAATTCAATCCAAGACAGTTCAAGG    8096
            ************************************************************

LA2093      CACTAAGAGGCATTGGTCAAAATTTAGTGCGTAAATATGAGGGTCCATTCAAGATCGTTG    8084
E42         CACTAAGAGGCATTGGTCAAAATTTAGTGCGTAAATATGAGGGTCCATTCAAGATCGTTG    8145
HEINZ       CACTAAGAGGCATTGGTCAAAATTTAGTGCGTAAATATGAGGGTCCATTCAAGATCGTTG    8156
            ************************************************************

LA2093      CCAAGGTGGGCAAGATCTCATACAAGTTGGAGTTACTTCCACACTTCAAGATCCACCCTG    8144
E42         CCAAGGTGGGCAAGATCTCATACAAGTTGGAGTTACTTCCACACTTCAAGATCCACCCTG    8205
HEINZ       CCAAGGTGGGCAAGATCTCATACAAGTTGGAGTTACTTCCACACTTCAAGATCCACCCTG    8216
            ************************************************************

LA2093      TATTTCATGCAAGCGTCCTCAAGCCATACCATGAGGACAAGGAGGATCCTAACCGGAACC    8204
E42         TATTTCATGCAAGCGTCCTCAAGCCATACCATGAGGACAAGGAGGATCCTAACCGGAACC    8265
HEINZ       TATTTCATGCAAGCGTCCTCAAGCCATACCATGAGGACAAGGAGGATCCTAACCGGAACC    8276
            ************************************************************

LA2093      GATCACAACGGGCTCCCATTACTGTCACTGCTTCGCAGGATCGGGAGATCGAAGCTATCA    8264
E42         GATCACAACGGGCTCCCATTACTGTCACTGCTTCGCAGGATCGGGAGATCGAAGCTATCA    8325
HEINZ       GATCACAACGGGCTCCCATTACTGTCACTGCTTCGCAGGATCGGGAGATCGAAGCTATCA    8336
            ************************************************************

LA2093      TGGATTATCAAGCCAAACGAAAGTGAGGTCAACAAGCCAGCGCCATGTTTTTTGAGCATT    8324
E42         TGGATTATCAAGCCAAACGAAAGTGAGGTCAACAAGCCAGCGCCATGTTTTTTGAGCATT    8385
HEINZ       TGGATTATCAAGCCAAACGAAAGTGAGGTCAACAAGCCAGCGCCATGTTTTTTGAGCATT    8396
            ************************************************************

LA2093      GGAAGGGACAAACTCAGAAAGAGACCACATGGGAGAAATATGAAGATTTGTGGCAGTTCA    8384
E42         GGAAGGGACAAACTCAGAAAGAGACCACATGGGAGAAATATGAAGATTTGTGGCAGTTCA    8445
HEINZ       GGAAGCGACAAACTCAGAAAGAGACCACATGGGAGAAATATGAAGATTTGTGGCAGTTCA    8456
            ***** ******************************************************

LA2093      AGGACAAAGTCCAGGAGTTCTTACAGCAATGCGCCGCGATTGTCGCATCATCAGGTGGGG    8444
E42         AGGACAAAGTCCAGGAGTTCTTACAAAAATGCGCCGCGATTGTCGCATCATCAGGTGGGG    8505
HEINZ       AGGACAAAGTCCAGGAGTTCTTACAGCAATGCGCCGCGATTGTCGCATCATCAGGTGGGG    8516
            *************************  *********************************

LA2093      GAGCGTGTGACGTCCCGCCACAATTTCAGCCTGATTCCGCGTCCGGAAAGGGGCAGATTG    8504
E42         GAGCGTGTGACGTCCCGCCACAATTTCAGCCTGATTCCGCGTCCGGAAAGGGGCAGATTG    8565
HEINZ       GAGCGTGTGACGTCCCGCCACAATTTCAGCCTGATTCCGCGTCCGGAAAGGGGCAGATTG    8576
            ************************************************************

LA2093      CTCTAGAGTTGTGCGGAGAACTCTAGAACATTTTATAACTCTCAAGAAGGCTAGAGAAGG    8564
E42         CTCTAGAGTTGTGCGGAGAACTCTAGAACATTTTATAACTCTCAAGAAGGCTAGAGAAGG    8625
```

```
HEINZ      CTCTAGAGTTGTGCGGAGAACTCTAGAACATTTTATAACTCTCAAGAAGGCTAGAGAAGG    8636
           ************************************************************

LA2093     TCCCAAAGTAATTTAGAATTCTCTAGAAAAGAGACCAAAGTGTAAATATTTTAGGGACTT    8624
E42        TCCCAAAGTAATTTAGAATTCTCTAGAAAAGAGACCAAAGTGTAAATATTTTAGGGACTT    8685
HEINZ      TCCCAAAGTAATTTAGAATTCTCTAGAAAAGAGACCAAAGTGTAAATATTTTAGGGACTT    8696
           ************************************************************

LA2093     GTAAAGTAAATATTAATTGCACTTTAGTCCCTAGGAAGTAGTATAAACAGAGGCGCCCCT    8684
E42        GTAAAGTAAATATTAATTGCACTTTAGTCCCTAGGAAGTAGTATAAACAGAGGCGCCCCT    8745
HEINZ      GTAAAGTAAATATTAATTGCACTTTAGTCCCTAGGAAGTAGTATAAACAGAGGCGCCCCT    8756
           ************************************************************

LA2093     CATTTGCAAAGACACCAAGCAAGTTGAAAGCAATCTTTCAAAGCTCTCTTTCTTTTTTTC    8744
E42        CATTTGCAAAGACACCAAGCAAGTTGAAAGCAATCTTTCAAAGCTCTCTTTCTTTTTTTC    8805
HEINZ      CATTTGCAAAGACACCAAGCAAGTTGAAAGCAATCTTTCAAAGCTCTCTTTCTTTTTTTC    8816
           ************************************************************

LA2093     TGAGTCTTTCTTTGCATTCTTTAAGTGTTCCAAGTCTTAAAGGCTTACTTGAGCTTACAA    8804
E42        TCAGTCTTTCTTTGCATTCTTTAAGTGTTCCAAGTCTTAAAGGCTTACTTGAGCTTACAA    8865
HEINZ      TCAGTCTTTCTTTGCATTCTTTAAGTGTTCCAAGTCTTAAAGGCTTACTTGAGCTTACAA    8876
           * **********************************************************

LA2093     GGTCGTGAAAGATTCGTGAGTAAGTTGTCAAGTGTCGCACAGAGTCTTAGCAAATACTCT    8864
E42        GGTCGTGAAAGATTCGTGAGTAAGTTGTCAAGTGTCGCACAGAGTCTTAGCAAATACTCT    8925
HEINZ      GGTCGTGAAAGATTCGTGAGTAAGTTGTCAAGTGTCGCACAGAGTCTTAGCAAATACTCT    8936
           ************************************************************

LA2093     AAGTCTGTGACACTAAGCTCTCTTTTTCGAAAGAAAACGAAGCACTAAAGATATTTCACA    8924
E42        AAGTATGTGACACTAAGCTCTCTTTTTCGAAAGAAAACGAAGCACTAAAGATATTTCACA    8985
HEINZ      AAGTCTGTGACACTAAGCTCTCTTTTTCGAAAGAAAACGAAGCACTAAAGATATTTCACA    8996
           **** *******************************************************

LA2093     AAACATGAGATTTGTCAAGGACTTGACCATTCTCATGTTTCAATTAGATATTATTAGAGT    8984
E42        AAACATGAGATTTGTCAAGGACTTGACCATTCTCATGTTTCAATTAGATATTATTAGAGT    9045
HEINZ      AAACATGAGATTTGTCAAGGACTTGACCATTCTCATGTTTCAATTAGATATTATTAGAGT    9056
           ************************************************************

LA2093     TTGTTTCTCCTATAGAAAAGGACCTTTGACGCAAAGTAAATAGCACAAATTGAAACAAAA    9044
E42        TCGTTTCTCCTATAGAAAAGGACCTTTGACGCAAAGGAAATAGCACAAATTGAAACAAAA    9105
HEINZ      TCGTTTCTCCTATAGAAAAGGACCTTTGACGCAAAGTAAATAGCACAAATTGAAACAAAA    9116
           * ********************************** ***********************

LA2093     AATCTTTTTGCAGGTCTCACTGTGTATTTGGCTTATACATAAAATGTAAAAGATCTAACT    9104
E42        AATCTTTTTGCAGGTCTCACTGTGTATTTGGCTTATACATAAAATGTAAAAGATCTAACT    9165
HEINZ      AATCTTTTTGCAGGTCTCACTGTGTATTTGGCTTATACATAAAATGTAAAAGATCTAACT    9176
           ************************************************************

LA2093     TCCGATGAACAACTTAAATGACTCAATTATATTAATTCCAACCTTTTGAGAAGGACTTTG    9164
E42        TCCGATGAACAACTTAAATGACTCAATTATATTAATTCCAACCTTTTGAGAAGGACTTTG    9225
HEINZ      TCCGATGAACAACTTAAATGACTCAATTATATTAATTCCAACCTTTTGAGAAGGACTTTG    9236
           ************************************************************

LA2093     GAACCGATGAAGGTAATTTACCAGCTCTTCCCTCCTCAATTCATCATCGTATGCTTTTCG    9224
E42        GAACCGATGAAGGTAATTTACCAGCTCTTCCCTCCTCAATTCATCATCGTATGCTTTTCG    9285
HEINZ      GAACCGATGAAGGTAATTTACCAGCTCTTCCCTCCTCAATTCATCATCGTATGCTTTTCG    9296
           ************************************************************

LA2093     TTTAAAAGAATCAAGTAAAACCTGAAACAATCCAATTCTTATCAAGTGCAGGATCAATAC    9284
E42        TTTAAAAGAATCAAGTAAAACCTGAAACAATCCAATTCTTATCAAGTGCAGGATCAATAC    9345
HEINZ      TTTAAAAGAATCAAGTAAAACCTGAAACAATCCAATTCTTATCAAGTGCAGGATCAATAC    9356
           ************************************************************

LA2093     TAGATAAAAAACTCAAATCACAGGACAAAACCTGTACCTCATAGGCGTTTTGAAGTTTCA    9344
E42        TAGATAAAAAACTCAAATCACAGGACAAAACCTGTACCTCATAGGCGTTTTGAAGTTTCA    9405
HEINZ      TAGATAAAAAACTCAAATCACAGGACAAAACCTGTACCTCATAGGCGTTTTGAAGTTTCA    9416
           ************************************************************

LA2093     TAAAAGCTTCAGCAGCCTTTTCATTACCCATATTTTTGTCAGGATGGACCAGCATTGCCT    9404
E42        TAAAAGCTTCAGCAGCCTTTTCATTACCCATATTTTTGTCAGGATGGACCAGCATTGCCT    9465
HEINZ      TAAAAGCTTCAGCAGCCTTTTCATTACCCATATTTTTGTCAGGATGGACCAGCATTGCCT    9476
           ************************************************************
```

```
LA2093        AATAGGAGATATTATTTAGCTGATTAGTTATTTCACTTTGATAGTAGGGCATTTCTAATG     9464
E42           AATAGGAGATATTATTTAGCTGATTAGTTATTTCACTTCGATAGTAGGGCA-TTCTAATG     9524
HEINZ         AATAGGAGATATTATTTAGCTGATTAGTTATTTCACTTTGATAGTAGGGCATTTCTAATG     9536
              ************************************** ************ ********

LA2093        AAAGCATTATCAGACTTCAAAAAGCATGATCTCCAGAAATATCAGAGACAGATATTTTTA     9524
E42           AAAGCATTATCAGACTTCAAAAAGCATGATCTCCAAAAATATCAGAGACAGATATTTTTA     9584
HEINZ         AAAGCATTATCAGACTTCAAAAAGCATGATCTCCAGAAATATCAGAGACAGATATTTTTA     9596
              *********************************** ************************

LA2093        GTGAATTATTTTCAAGAAGATATAAACAACCACCTTTTTCCTATATTCTCTCTTGAGAAC     9584
E42           GTGAATTATTTTCAAGAAGATGTAAACAATCACCTTTTTCCTATATTCTCTCTTGAGAAC     9644
HEINZ         GTGAATTATTTTCAAGAAGATATAAACAATCACCTTTTTCCTATATTCTCTCTTGAGAAC     9656
              ********************* ******* ******************************

LA2093        TGAAGCATCAGTATTCTGAAATCGCATTAGGCCCAGTGCTGAATAGTGATTTGTACTGTT     9644
E42           TGAAGCATCAGTATTCTGAAATCGCATTAGGCCCAGTGCTGAATAGTGATTTGTACTGTT     9704
HEINZ         TGAAGCATCAGTATTCTGAAATCGCATTAGGCCCAGTGCTGAATAGTGATTTGTACTGTT     9716
              ************************************************************

LA2093        TAATAATCTGAGAACTTCATCTTCGGAAGTCATCTCAACATCAGATCCACTAGTTGAAGG     9704
E42           TAATAATCTGAGAACTTCATCTTCGGAAGTCATCTCAACATCAGATCCACTAGTTGAAGG     9764
HEINZ         TAATAATCTGAGAACTTCATCTTCGGAAGTCATCTCAACATCAGATCCACTAGTTGAAGG     9776
              ************************************************************

LA2093        GATGCCAGTGTCACGATCAACAGGATGAACATACCCATCATCAGTAGATGGAGGCACTGA     9764
E42           GATGCCAGTGTCACGATCAACAGGATGAACATACCCATCATCAGTAGATGGAGGCACTGA     9824
HEINZ         GATGCCAGTGTCACGATCAACAGGATGAACATACCCATCATCAGTAGATGGAGGCACTGA     9836
              ************************************************************

LA2093        TCCACGCGAACAGAAGCTAGGTTCACCTTGTACTCCAGTTGTTTGATCAGGAAAGGTGTG     9824
E42           TCCACGCGAACAGAAGCTAGGTTCACCTTGTACTCCAGTTGTTTGATCAGGAAAGGTGTG     9884
HEINZ         TCCACGCGAACAGAAGCTAGGTTCACCTTGTACTCCAGTTGTTTGATCAGGAAAGGTGTG     9896
              ************************************************************

LA2093        GGCCCTTCTTTGCTCATTTACATTGTTTCTTAAGAAGAATATGAGAATATCACTCGAAAT     9884
E42           GGCCCTTCTTTGCTCATTTACATTGTTTCTTAAGAAGAATATGAGAATATCACTCGAAAT     9944
HEINZ         GGCCCTTCTTTGCTCATTTACATTGTTTCTTAAGAAGAATATGAGAATATCACTCGAAAT     9956
              ************************************************************

LA2093        AAATGACAAATTCAATATGAAGATTAAACCTATCCATCCAACATATGTCCATGCGCAAAA     9944
E42           AAATGACAAATTCAATATGAAGATTAAACCTATCCATCCAACATATGTCCATGCGCAAAA     10004
HEINZ         AAATGACAAATTCAATATGAAGATTAAACCTATCCATCCAACATATGTCCATGCGCAAAA     10016
              ************************************************************

LA2093        TACAGAATACGAAGTTATGACAAACACTGCAAGCCGTTCATGTTTCAACCTATAAAGAGC     10004
E42           TACAGAATACGAAGTTATGACAAACACTGCAAGCCGTTCATGTTTCAACCTATAAAGAGC     10064
HEINZ         TACAGAATACGAAGTTATGACAAACACTGCAAGCCGTTCATGTTTCAACCTATAAAGAGC     10076
              ************************************************************

LA2093        TCCTACATTTAAAAAAAAAACACTTGGTGGATTAAACTATAAAGTAAAAAGAAATCCTCA     10064
E42           TCCTACATTT-AAAAAAAAACACTTGGTGGATTAAACTATAAAGTAAAAAGAAATCCTCA     10123
HEINZ         TCCTACATTT-AAAAAAAAACACTTGGTGGATTAAACTATAAAGTAAAAAGAAATCCTCA     10135
              ********** *************************************************

LA2093        ACACTAAGGGTGGCAAACAGGCGGGTCGAGTCGGACATGGATTGATCAAAAATGAGTCAA     10124
E42           ACACTAAGGGTGGCAAACAGGCGGGTCGAGTCGGACATGGATTGATCAAAAATGAGTCAA     10183
HEINZ         ACACTAAGGGTGGCAAACAGGCGGGTCGAGTCGGACATGGATTGATCAAAAATGAGTCAA     10195
              ************************************************************

LA2093        GTGAAAATAAATAAAATATTTGACCCGACCCATATTTGATATGGATAAAATTAGGCAACC     10184
E42           GTGAAAATAAATAAAATATTTGACCCGACCCATATTTGATATGGATAAAATTAGGCAACC     10243
HEINZ         GTGAAAATAAATAAAATATTTGACCCGACCCATATTTGATATGGATAAAATTAGGCAACC     10255
              ************************************************************

LA2093        CCAATGGATCATGAGTACTTCCAAATTACAAGTTTTTTTCTTTCTCTTTTTCTCCTTTTA     10244
E42           CCAATGGATCATGAGTACTTCCAAATTACAAGTTTTTTTCTTTCTCTTTTTCTCCTTTTA     10303
HEINZ         CCAATGGATCATGAGTACTTCCAAATTACAAGTTTTTTTCTTTCTCTTTTTCTCCTTTTA     10315
              ************************************************************

LA2093        GGGTGTGTTTGGTTGTGAAGGAAATATTTCCATGTTTCCCTGGAAAATATTTTCCTGATT     10304
```

```
E42         GGGTGTGTTTGGTTGTGAAGGAAATATTTCCATGTTTCCCTGGAAAATATTTTCCTGATT    10363
HEINZ       GGGTGTGTTTGGTTGTGAAGGAAATATTTCCATGTTTCCCTGGAAAATATTTTCCTGATT    10375
            ************************************************************

LA2093      TAAGGGAAGTTTTAAATTTTTTTCCTCACTTAATGTATGGAAGTCATTTTTCTTATATTA    10364
E42         TAAGGGAAGTTTTAAATTTTTTTCCTCACTTAATGTATGGAAGTCATTTTTCTTATATTA    10423
HEINZ       TAAGGGAAGTTTTAAATTTTTTTCCTCACTTAATGTATGGAAGTCATTTTTCTTATATTA    10435
            ************************************************************

LA2093      GAGGAATGTAAGTCCAGTTGAAAACAAAATTCCAAAACATTTAAATAAACCAACATGAGA    10424
E42         GAGGAATGTAAGTCCAGTTGAAAACAAAATTCTAAAACATTTAAATAAACCAACATGAGA    10483
HEINZ       GAGGAATGTAAGTCCAGTTGAAAACAAAATTCCAAAACATTTAAATAAACCAACATGAGA    10495
            ******************************** ***************************

LA2093      AAATTGGAAAAAAAAATTTCTTCATAACAACACACCCTTAATAAGCGAACTTTTTAGAGGT    10484
E42         AAATTGGAAAAAAAAATTTCTTCATAACAACACACCCTTAATAAGCGAACTTTTTAGAGGT    10543
HEINZ       AAATTGGAAAAAAAAATTTCTTCATAACAACACACCCTTAATAAGCGAACTTTTTAGAGGT    10555
            ************************************************************

LA2093      TCCTCCATTCTTATATAATTTAAGAGGATTGTAATTGGTGAATATCTATTTTCTTATCCA    10544
E42         TCCTCCATTCTTATATAATTTAAAAGGATTGTAATTGGTGAATATCTATTTTCTTATCCA    10603
HEINZ       TCCTCCATTCTTATATAATTTAAGAGGATTGTAATTGGTGAATATCTATTTTCTTATCCA    10615
            *********************** ************************************

LA2093      TATTTTATAATGGATTATATGGGATATATATATTTGATCCACTTTTAAATAATTGGATAA    10604
E42         TATTTTATAATGGATTATATGGGATATATATATTTGATCCACTTTTAAATAATTGGATAA    10663
HEINZ       TATTTTATAATGGATTATATGGGATATATATATTTGATCCACTTTTAAATAATTGGATAA    10675
            ************************************************************

LA2093      CCAACCCATTCAAAATGAGTTGGATCGAGTGGATAACCATAAATTTTACCCATTTTGCCA    10664
E42         CCAACCCATTCAAAATGAGTTGGATCGAGTGGATAACCATAAATTTTACCCATTTTGCCA    10723
HEINZ       CCAACCCATTCAAAATGAGTTGGATCGAGTGGATAACCATAAATTTTACCCATTTTGCCA    10735
            ************************************************************

LA2093      TCCCTACTCAACACTAAACTTGAAAACAATCACCTCCACTCAAGACCAGAAGAACTGCTG    10724
E42         TCCCTACTCAACACTAAACTTGAAAACAATCACCTCCACTCAAGACCAGAAGAACTGCTG    10783
HEINZ       TCCCTACTCAACACTAAACTTGAAAACAATCACCTCCACTCAAGACCAGAAGAACTGCTG    10795
            ************************************************************

LA2093      CCCAAAATCTTCCATAGAACCACAAGTAACTGATACTCACTGCAGCAACTCCTAGAAATG    10784
E42         CCCAAAATCTTCCATAGAACCACAAGTAACTGATACTCACTGCAGCAACTCCTAGAAATG    10843
HEINZ       CCCAAAATCTTCCATAGAACCACAAGTAACTGATACTCACTGCAGCAACTCCTAGAAATG    10855
            ************************************************************

LA2093      TGATGATGAACCCAGTAAGAAGTCCAATAACAGCAACTACTGCCTGAATGTTAAGACGAC    10844
E42         TGATGATGAACCCAGTAAGAAGTCCAATAACAGCAACTACTGCCTGAATGTTAAGACGAC    10903
HEINZ       TGATGATGAACCCAGTAAGAAGTCCAATAACAGCAACTACTGCCTGAATGTTAAGACGAC    10915
            ************************************************************

LA2093      TTCATTACAGCAAGATATTGAAATGTTCCAGAGAAATTATGCAGTTTAAAGGAAGGAATC    10904
E42         TTCATTACAGCAAGATATTGAAATGTTCCAGAGAAATTATGCAGTTTAAAGGAAGGAATC    10963
HEINZ       TTCATTACAGCAAGATATTGAAATGTTCCAGAGAAATTATGCAGTTTAAAGGAAGGAATC    10975
            ************************************************************

LA2093      TAATCAAGAATTAGAAAGAAGACACAAAGCCAGAGCGTGACCGCGATAACTCACAAAATC    10964
E42         TAATCAAGAATTAGAAAGAAGACACAAAGCCAGAGCGTGACCGCGATAACTCACAAAATC    11023
HEINZ       TAATCAAGAATTAGAAAGAAGACACAAAGCCAGAGCGTGACCGCGATAACTCACAAAATC    11035
            ************************************************************

LA2093      AATATACTAATTGAACAAAGTTAAAGTCTAAGACACTATTGTGACCACCTTCAGTAATTC    11024
E42         AATATACTAATTGAACAAAGTTAAAGTCTAAGACACTATTGTGACCACCTTCAGTAATTC    11083
HEINZ       AATATACTAATTGAACAAAGTTAAAGTCTAAGACACTATTGTGACCACCTTCAGTAATTC    11095
            ************************************************************

LA2093      AATTATCAACTGAAGGAAACGGAACAAAGTATACAGAATCAAAATCAGCAAAACACAACT    11084
E42         AATTATCAACTGAAGGAAACGGAACAAAGTATACAGAATCAAAATCAGCAAAACACAACT    11143
HEINZ       AATTATCAACTGAAGGAAACGGAACAAAGTATACAGAATCAAAATCAGCAAAACACAACT    11155
            ************************************************************

LA2093      AAAGAACCGTAAAGCATGAAATGGCCAAAGTCAAAAAGTGAAATACAATGATTAATGTCC    11144
E42         AAAGAACCGTAAAGCATGAAATGGCCAAAGTCAAAAAGTGAAATACAATGATTAATGTCC    11203
HEINZ       AAAGAACCGTAAAGCATGAAATGGCCAAAGTCAAAAAGTGAAATACAATGATTAATGTCC    11215
```

```
            ************************************************************

LA2093      AATTTTAAAAAAAAAAAATTTCACACAATTTATAAGTCGCTTAATTCAGTGACTTCAATA     11204
E42         AATTTTAAAAAAAAAAAA-TTCACACAATTTATAAGTCGCTTAATTCAGTGACTTCAATA     11262
HEINZ       AATTTTAAAAATAAAAAA-TTCACACAATTTATAAGTCGCTTAATTCAGTGACTTCAATA     11274
            *********** ****** *****************************************

LA2093      ATAAGAAAAAACCAGAGCTCCTCAAAACTATTTTTGGTTCAGCAGGAAATCATTCATTAG     11264
E42         ATAAGAAAAAACCAGAGCTCCTCAAAACTATTTTTGGTTCAGCAGGAAATCATTCATTAG     11322
HEINZ       ATAAG-AAAAACCAGAGCTCCTCAAAACTATTTTTGGTTCAGCAGGAAATCATTCATTAG     11333
            ***** ******************************************************

LA2093      TCAACACAATGTTGAGAAGCAATTAGGGTTATATATTTATGCTTACAAACACCTTTTATC     11324
E42         TCAACACAATGTTGAGAAGCAATTAGGGTTATATATTTATGCTTACAAACACCTTTTATC     11382
HEINZ       TCAACACAATGTTGAGAAGCAATTAGGGTTATATATTTATGCTTACAAACACCTTTTATC     11393
            ************************************************************

LA2093      ATCTCAGGGGTAGATAATGTTGCTATCCACACAAGAACAGAAGATAGATGCTTACCAAAA     11384
E42         ATCTCAGGGGTAGATAATGTTGCTATCCACACAAGAACAGAAGATAGATGCTTACCAAAA     11442
HEINZ       ATCTCAGGGGTAGATAATGTTGCTATCCACACAAGAACAGAAGATAGATGCTTACCAAAA     11453
            ************************************************************

LA2093      TCAATAGAAACTTGAATTTAGCGGCTGCAATCAAAGAAAGAACACCACACCATATGACTG     11444
E42         TCAATAGAAACTTGAATTTAGCGGCTGCAATCAAAGAAAGAACACCACACCATATGACTG     11502
HEINZ       TCAATAGAAACTTGAATTTAGCGGCTGCAATCAAAGAAAGAACACCACACCATATGACTG     11513
            ************************************************************

LA2093      AGAGAATTGATGTCATCCTCATACACAGAAAGGACTCAATTCCTCTAAGGGCACAATCCA     11504
E42         AGAGAATTGATGTCATCCTCATACACAGAAAGGACTCAATTCCTCTAAGGGCACAATCCA     11562
HEINZ       AGAGAATTGATGTCATCCTCATACACAGAAAGGACTCAATTCCTCTAAGGGCACAATCCA     11573
            ************************************************************

LA2093      ACCAAACCATTGATAACAAAAGCATTATATTTCCAAAATGCATGATCCACCTAAACACTA     11564
E42         ACCAAACCATTGATAACAAAAGCATTATATTTCCAAAATGCATGATCCACCTAAACACTA     11622
HEINZ       ACCAAACCATTGATAACAAAAGCATTATATTTCCAAAATGCATGATCCACCTAAACACTA     11633
            ************************************************************

LA2093      TAGGGTATGCTTGCTGACCCTTTTCTTGAACATAACCAATTGCAACTAATAAATTGCTCT     11624
E42         TAGGGTATGCTTGCTGACCCTTTTCTTGAACATAACCAATTGCAACTAATAAATTGCTCT     11682
HEINZ       TAGGGTATGCTTGCTGACCCTTTTCTTGAACATAACCAATTGCAACTAATAAATTGCTCT     11693
            ************************************************************

LA2093      TTAAAGGGTCAAGCAAAGGTTTCCACCTCTCTAACCACTGAATTGATACCTTTGAGGCAG     11684
E42         TTAAAGGGTCAAGCAAAGGTTTCCACCTCTCTAACCACTGAATTGATACCTTTGAGGCAG     11742
HEINZ       TTAAAGGGTCAAGCAAAGGTTTCCACCTCTCTAACCACTGAATTGATACCTTTGAGGCAG     11753
            ************************************************************

LA2093      ACAAAGCCAACGTCATCAAGTTCCTAAAAACAACCATCATAGACACTTTAAAATTTTCTA     11744
E42         ACAAAGCCAACGTCATCAAGTTCCTAAAAACAACCATCATAGACACTTTAAAATTTTCTA     11802
HEINZ       ACAAAGCCAACGTCATCAAGTTCCTAAAAACAACCATCATAGACACTTTAAAATTTTCTA     11813
            ************************************************************

LA2093      ATGTATTGTCTCCATCTAGTCCTGTTCCTAAGTGACCAAAACATCCCTTTGAACTATCTG     11804
E42         ATGTATTGTCTCCATCTAGTCCTGTTCCTAAGTGACCAAAACATCCCTTTGAATTATCTG     11862
HEINZ       ATGTATTGTCTCCATCTAGTCCTGTTCCTAAGTGACCAAAACATCCCTTTGAATTATCTG     11873
            ****************************************************** *****

LA2093      AGTGCTTACTAGAGTTTGGAGATGTATCAATTCCTTTTCCTGACTCAGAATCCACCCCTC     11864
E42         AGTGCTTACTAGAGTTTGGAGATGTATCAATTCCTTTTCCTGACTCAGAATCCACCCCTC     11922
HEINZ       AGTGCTTACTAGAGTTTGGAGATGTATCAATTCCTTTTCCTGACTCAGAATCCACCCCTC     11933
            ************************************************************

LA2093      TACTTTTACAATTTTGGAAAATTCCTGCATCAGTGTCTTCTGGCCCTGTTTCCTCTGGAA     11924
E42         TACTTTTACAATTTTGGAAAATTCCTGCATCAGTGTCTTCTGGCCCTGTTTCCTCTGGAA     11982
HEINZ       TACTTTTACAATTTTGGAAAATTCCTGCATCAGTGTCTTCTGGCCCTGTTTCCTCTGGAA     11993
            ************************************************************

LA2093      TCAACAATGTATCAATACCTTCCTTTTCCTTTTTAAGTGATCTTCTAGATTTGTTCTTGC     11984
E42         TCGACAATGTATCAATACCTTCCTTTTCCTTTTTAAGTGATCTTCTAGATTTGTTCTTGC     12042
HEINZ       TCGACAATGTATCAATACCTTCCTTTTCCTTTTTAAGTGATCTTCTAGATTTGTTCTTGC     12053
            ** *********************************************************
```

```
LA2093       TTTTCTTCTTGCCTTCACTTTCATGTTTATTGTTTAAGCTCTCTGTTATAGATATGCCAG    12044
E42          TTTTCTTCTTGCCTTCACTTTCATGTTTATTGTTTAAGCTCTCTGTTATAGATATGCCAG    12102
HEINZ        TTTTCTTCTTGCCTTCACTTTCATGTTTATTGTTTAAGCTCTCTGTTATAGATATGCCAG    12113
             ************************************************************

LA2093       AAAGGTTACCATTAAAAGGTTCTTCAGCATTTTTGCCTTCTGCCTCAGTCAGATTACTGT    12104
E42          AAAGGTTACCATTAAAAGGTTCTTCAGCATTTTTGCCTTCTGCCTCAGTCAGATTACTGT    12162
HEINZ        AAAGGTTACCATTAAAAGGTTCTTCAGCATTTTTGCCTTCTGCCTCAGTCAGATTACTGT    12173
             ************************************************************

LA2093       TTTCATTCACATTTAACAATCCAGATCCAGAATCTGAAACTCCTTTTCTGTATTTCTGTC    12164
E42          TTTCATTCTCATTTAACAATCCAGATCCAGAATCTGAAACTCCTTTTCTGTATTTCTGTC    12222
HEINZ        TTTCATTCTCATTTAACAATCCAGATCCAGAATCTGAAACTCCTTTTCTGTATTTCTGTC    12233
             ******** ***************************************************

LA2093       GGTTTCCTTTGCGAGCCATTGACTATATATGGTATCAACAAACTCCAGCGACAAGCCAGT    12224
E42          GGTTTCCTTTGCGAGCCATTGACTATATATGGTATCAACAAACTCCAGCGACAAGCCAGT    12282
HEINZ        GGTTTCCTTTGCGAGCCATTGACTATATATGGTATCAACAAACTCCAGCGACAAGCCAGT    12293
             ************************************************************

LA2093       TCAACTCATCTCCAGTGCCTCGTCATATATCAAAAGTCCTGTGTCACAAGCAATACGAAA    12284
E42          TCAACTCATCTCCAGTGCCTCGTCATATATCAAAAGTCCTGTGTCACAAGCAATACGAAA    12342
HEINZ        TCAACTCATCTCCAGTGCCTTGTCATATATCAAAAGTCCTGTGTCACAAGCAATACGAAA    12353
             ******************** ***************************************

LA2093       GTAAGCAAATTTACAGGAGATTTCAAGCATCAACAATAGTTTAAAGGTGCCACCACTTAG    12344
E42          GTAAGCAAATTTACAGGAGATTTCAAGCATCAACAATAGTTTAAAGGTGCCACCACTTAG    12402
HEINZ        GTAAGCAAATTTACAGGAGATTTCAAGCATCAACAATAGTTTAAAGGTGCCACCACTTAG    12413
             ************************************************************

LA2093       ATAATCAAACAATGAACACACATACAGGCATATCTCATTCCTAATGCATGCAACTCAGTG    12404
E42          ATAATCAAACAATGAACACACATACAGGCATATCTCATTCCTAATGCATGCAACTCAGTG    12462
HEINZ        ATAATCAAACAATGAACACACATACAGGCATATCTCATTCCTAATGCATGCAACTCAGTG    12473
             ************************************************************

LA2093       AAATTAGCTTTCTTCACAATTTCGGAGCCGTGACTGTAAAAGAAACAACATTCTCCTCAT    12464
E42          AAATTAGCTTTCTTCACAATTTCGGAGCCGTGACTGTAAAAGAAACAACATTCTCCTCAT    12522
HEINZ        AAATTAGCTTTCTTCACAATTTCGGAGCCGTGACTGTAAAAGAAACAACATTCTCCTCAT    12533
             ************************************************************

LA2093       GCGTATTCCCTCAACTATCGTCCATTCACGCGTAATTAATCAGAAACCTATCGCTCATTA    12524
E42          GCGTATTCCCTCAACTATCGTCCATTCACGCGTAATTAATCAGAAACCTATCGCTCATTA    12582
HEINZ        GCGTATTCCCTCAACTATCGTCCATTCACGCGTAATTAATCAGAAACCTATCGCTCATTA    12593
             ************************************************************

LA2093       TTAACCTTCTTTCTTTGCAATATACTGTCATCTTCCCCGAGATTTTCCTTGATCTAGCCA    12584
E42          TTAACCTTCTTTCTTTGCAATATACTGTCATCTTCCCCGAGATTTTCCTTGATCTAGCCA    12642
HEINZ        TTAACCTTCTTTCTTTGCAATATACTGTCATCTTCCCCGAGATTTTCCTTGATCTAGCCA    12653
             ************************************************************

LA2093       TGTTATCAGTAAAACACTGTCTGTTCACTGCTCTCAATTAATATCAATTTCCTTTAAGCA    12644
E42          TGTTATCAGTAAAACACTGTCTGTTCACTGCTCTCAATTAATATCAATTTCCTTTAAGCA    12702
HEINZ        TGTTATCAGTAAAACACTGTCTGTTCACTGCTCTCAATTAATATCAATTTCCTTTAAGCA    12713
             ************************************************************

LA2093       GGACATACATAAACTAATCTAATAAAAGGGCAATACAAGGAATTAATAATGTCATGCATC    12704
E42          GGAAATACATAAACTAATCTAATAAAAGGGCAATACAAGGAATTAATAATGTCATGCATC    12762
HEINZ        GGAAATACATAAACTAATCTAATAAAAGGGCAATACAAGGAATTAATAATGTCATGCATC    12773
             *** ********************************************************

LA2093       CAACATCACCAGTTTATGGGTTTCTGCAACGTCTTCTACTTGAAACAACAAATACAAGAG    12764
E42          CAACATCACCAGTTTATGGGTTTCTGCAACGCCTTCTACTTGAAACAACAAATACAAGAG    12822
HEINZ        CAACATCACCAGTTTATGGGTTTCTGCAACGTCTTCTACTTGAAACAACAAATACAAGAG    12833
             ******************************* ****************************

LA2093       ATATTATTGGTTATCTTTTAAAATCATATTGAACTTTGTGAGAAATATAGGAAAAGAATA    12824
E42          ATATTATTGGTTATCTTTTAAAATCATATTGAACTTTGTGAGAAATATAGGAAAAGAATA    12882
HEINZ        ATATTATTGGTTATCTTTTAAAATCATATTGAACTTTGTGAGAAATATAGGAAAAGAATA    12893
             ************************************************************

LA2093       TTATTGAATTGTTGTTGTGTCTACATTATTACATGAGACTCTATTTATAGACCATAGAAT    12884
E42          TTATTGAATTGTTGTTGTGTCTACATTATTACATGAGACTCTATTTATAGACCATAGAAT    12942
```

```
HEINZ        TTATTGAATTGTTGTTGTGTCTACATTATTACATGAGACTCTATTTATAGACCATAGAAT    12953
             ************************************************************

LA2093       ACAATCCTTTATCAAGTAGGATACTATTTATTTATTTTTGAGAAAGCAGTAGGATACTA    12944
E42          ACAATCCTTTATCAAGTAGGATACTATTTATTTATTTTTGAGAAAGCAGTAGGATACTA    13002
HEINZ        ACAATCCTTTATCAAGTAGGATACTATTTATTTATTTTTGAGAAAGCAGTAGGATACTA    13013
             ************************************************************

LA2093       TTTAGTATTCCTATTCCTATTCTAATTGGATTGTACAAACATATTCCTATTCTAATAGGA    13004
E42          TTTAGTATTCCTATTCCTATTCTAATTGGATTGTACAAACA-------------------    13043
HEINZ        TTTAGTATTCCTATTCCTATTCTAATTGGATTGTACAAACA-------------------    13054
             *****************************************

LA2093       TTGTATAAACCTATTCCTATTCTAATAGGATTGTATAAACCTATTCCTATTCAAATAGGA    13064
E42          -----------TATCCCTATTCTAATAGGATTGTATAAACCTATTCCTATTCAAATAGGA    13092
HEINZ        -----------TATTCCTATTCTAATAGGATTGTATAAACCTATTCCTATTCAAATAGGA    13103
                        *** *********************************************

LA2093       TCTGTATAAACCTATTCCTATTCAAATAGGATCTATATAAACCTATTCCTATTTCAATAG    13124
E42          TCTGTATAAACCTATTCCTATTCAAATAGGATCTGTATAAACCTATTCCTATTTCAATAG    13152
HEINZ        TCTGTATAAACCTATTCCTATTCAAATAGGATCTATATAAACCTATTCCTATTTCAATAG    13163
             ********************************** *************************

LA2093       GATTATATAAACCTATTCCTATTCCAACACTCCCCCTCAAGCTAGTGCATACAATTCATG    13184
E42          GATTATATAAACCTATTCCTATTCCAACACTCCCCCTCAAGCTAGTGCATACAATTCATG    13212
HEINZ        GATTATATAAACCTATTCCTATTCCAACACTCCCCCTCAAGCTAGTGCATACAATTCATG    13223
             ************************************************************

LA2093       TACCTAGCTTGTTACAAATGTAATTAACACGAGGACCGATGAGGGGCTTGGTGTGAGATA    13244
E42          TACCTAGCTTGTTACAAATGTAATTAACACGAGGACCGATGAGGGGCTTGGTGTGAGATA    13272
HEINZ        TACCTAGCTTGTTACAAATGTAATTAACACGAGGACCGATGAGGGGCTTGGTGTGAGATA    13283
             ************************************************************

LA2093       TTCACAAGTTCATCAGTCGACTTCACAAAATTGATAGTCAATCTCAATGTGATTGTCTTC    13304
E42          TTCACAAGTTCATCAGTCGACTTCACAAAATTGACAGTCAATCTCAATGTGATTGTCTTC    13332
HEINZ        TTCACAAGTTCATCAGTCGACTTCACAAAATTGATAGTCAATCTCAATGTGATTGTCTTC    13343
             ********************************** *************************

LA2093       TCATTAAATACTGGATTTGATGCATAATGTCAGTAAACACAGATTAAATTTCAGTGTTGA    13364
E42          TCATTAAATACTGGATTTGATGCATAATGTCAGTAAACACAGATTAAATTTCAGTGTTGA    13392
HEINZ        TCATTAAATACTGGATTTGATGCATAATGTCAGTAAACACAGATTAAATTTCAGTGTTGA    13403
             ************************************************************

LA2093       ACTTCCCAAACAAAACCTGCAAAGACTGCTTCAAACTATAGAGTGACTCATATAATCGAC    13424
E42          ACTTCCCAAACAAAACCTGCAAAGACTGCTTCAAACTATAGAGTGACTCATATAATCGAC    13452
HEINZ        ACTTCCCAAACAAAACCTGCAAAGACTGCTTCAAACTATAGAGTGACTCATATAATCGAC    13463
             ************************************************************

LA2093       ATACAAGGAGAGTGACTCACATAATCGACATACAAGGCTACTGAACTCCCCCTGAGCAAC    13484
E42          ATACAAGGAGAGTGACTCACATAATCGACATACAAGGCTACTGAACTCCCCCTGAGCAAC    13512
HEINZ        ATACAAGGAGAGTGACTCACATAATCGACATACAAGGCTACTGAACTCCCCCTGAGCAAC    13523
             ************************************************************

LA2093       AAAGTGCTCAAAACCTAGTATAAAGTATATGGATCATATTGGAATCAATTGATGAGTCGA    13544
E42          AAAGTGCTCAAAACCTAGTATAAAGTATATGGATCATATTGGAATCAATTGATGAGTCGA    13572
HEINZ        AAAGTGCTCAAAACCTAGTATAAAGTATATGGATCATATTGGAATCAATTGATGAGTCGA    13583
             ************************************************************

LA2093       TATTAAACAAGTCACCGAAAAACTGGCTCGAACATGCTCATATGTCGGAGTATTAGGTTT    13604
E42          TATTAAACAAGTCACCGAAAAAATGGCTCGAACATGCTCATATGTCGGAGTATTAGGTTT    13632
HEINZ        TATTAAACAAGTCACCGAAAAACTGGCTCGAACATGCTCATATGTCGGAGTATTAGGTTT    13643
             ********************** *************************************

LA2093       GATGGCTAGCAGTGGTCACAATCTAAGAAATAAAATTATATAGGTAAGGTCAGAATGATG    13664
E42          GATGGCTAGCAGTGGTCACAATCTAAGAAATAAAATTATATAGGTAAGGTCGGAATGATG    13692
HEINZ        GATGGCTAGCAGTGGTCACAATCTAAGAAATAAAATTATATAGGTAAGGTCAGAATGATG    13703
             *************************************************** ********

LA2093       TCACGACCCAACTAGAAAATAAAAATTAAATAGGATAGTTCGAATTGATGGAACGACCCC    13724
E42          TCACGACCCAACTAGAAAATAAAAATTAAATAGGATAGTTCGAATTGATGGAACGACCCC    13752
HEINZ        TCACGACCCAACTAGAAAATAAAAATTAAATAGGATAGTTCGAATTGATGGAACGACCCC    13763
             ************************************************************
```

```
LA2093      ATTGAAAAAGAGAAATAATATGGGTAGGATCGAATTGATGGCACAACCCTACGCAAATCA    13784
E42         ATTGAAAAAGAGAAATAATATGGGTAGGATCGAATTGATGGCACAACCCTACGCAAATCA    13812
HEINZ       ATTGAAAAAGAGAAATAATATGGGTAGGATCGAATTGATGGCACAACCCTACGCAAATCA    13823
            ************************************************************

LA2093      AATTTGAGTCACCAGAAAGACAAATGGAACACGCAAATCAAATCTGAGTCACTGAAAAGA    13844
E42         AATTTGAGTCACCAGAAAGACAAATGGAACACGCAAATCAAATCTGAGTCACTGAAAAGA    13872
HEINZ       AATTTGAGTCACCAGAAAGACAAATGGAACACGCAAATCAAATCTGAGTCACTGAAAAGA    13883
            ************************************************************

LA2093      CACACAGTGCTATGCAACTCATATATGAATAAGTAGTTCGGAAAAAAAACCCATGGTACTA    13904
E42         CACACAGTGCTATGCAACTCATATATGAATAAGTAGTTCGGAAAAAAAACCCATGGTACTA    13932
HEINZ       CACACAGTGCTATGCAACTCATATATGAATAAGTAGTTCGGAAAAAAAACCCATGGTACTA    13943
            ************************************************************

LA2093      CGCATATTAAATATGAAAAGTAGTCCAGAGAGATGCACGGTGCAATGCAAATAAGATATG    13964
E42         CGCATATTAAATATGAAAAGTAGTCCGGAGAGATGCACGGTGCAATGCAAATAAGATATG    13992
HEINZ       CGCATATTAAATATGAAATGTAGTCCAGAGAGATGCACGGTGCAATGCAAATAAGATATG    14003
            ****************** ******* *********************************

LA2093      CAAAAAAAAGTAGTCACTAGAAGGATGGCATGGTGCCTACACAAATTAAATATGAAAAAG    14024
E42         CAAAAAAAAGTAGTCACTAGAAGGATGGCATGGTGCCTACACAAATTAAATATGAAAAAG    14052
HEINZ       CAAAAAAAAGTAGTCACTAGAAGGATGGCATGGTGCCTACACAAATTAAATATGAAAAAG    14063
            ************************************************************

LA2093      TAGTCACCAGAATGATGGTGCGGTGCTACACAAATTAGATATGAGAAAATAGTCACCAGA    14084
E42         TAGTCACCAGAATGATGGTGTGGTGCTACACAAATTAGATATGAGAAAATAGTCACCAGA    14112
HEINZ       TAGTCACCAGAATGATGGTGCGGTGCTACACAAATTAGATATGAGAAAATAGTCACCAGA    14123
            ******************** ***************************************

LA2093      ATGATACAGGGTGACCCAACTTTTGCTAACATAATCATTAGCCGGACGGGCGGCACATAT    14144
E42         ATGATACAGGGTGACCCAACTTTTGCTAACATAATCATTAGCCGGACGGGCGGCACATAT    14172
HEINZ       ATGATACAGGGTGACCCAACTTTTGCTAACATAATCATTAGCCGGACGGGCGGCACATAT    14183
            ************************************************************

LA2093      GAGTAATAGCCGCCCGCCGACAGCAGAAGCTCTTTAATTGTTTACCAAGATCGTAGAGGC    14204
E42         GAGTAATAGCCGCCCGCCGACAGCAGAAGCTCTTTAATTGTTTACCAAGATCGTAGAGGC    14232
HEINZ       GAGTAATAGCCGCCCACCGACAGCAGAAGCTCTTTAATTGTTTACCAAGATCGTAGAGGC    14243
            *************** ********************************************

LA2093      TTTGATACCATGTGAGAAATATAGGAAAAGAATATTATTGAATTGTTGTTGTGTCTACAT    14264
E42         TTTGATACCATGTGAGAAATATAGGAAAAGAATATTATTGAATTGTTGTTGTGTCTACAT    14292
HEINZ       TTTGATACCATGTGAGAAATATAGGAAAAGAATATTATTGAATTGTTGTTGTGTCTACAT    14303
            ************************************************************

LA2093      TATTACATGAGACTCTATTTATAGACCATAGAATACAATCCTTTATAAGTAGGATACTAT    14324
E42         TATTACATGAGACTCTATTTATAGACCATAGAATACAATCCTTTATAAGTAGGATACTAT    14352
HEINZ       TATTACATGAGACTCTATTTATAGACCATAGAATACAATCCTTTATAAGTAGGATACTAT    14363
            ************************************************************

LA2093      TTAGTATTCCTATTCCAATTTCTATTGGATTGTATAAACATATTCCTATTCTAATAGGAT    14384
E42         TTAGTATTCCTGTTCCAATTTCTATTGGATTGTATAAACATATTCCTATTCTAATAGGAT    14412
HEINZ       TTAGTATTCCTATTCCAATTTCTATTGGATTGTATAAACATATTCCTATTCTAATAGGAT    14423
            *********** ************************************************

LA2093      TGTATACACCTATTCCAATTCTAATAGGATTGTATAAACATATTCCTATTTCAATAGGAT    14444
E42         TGTATACACCTATTCCAATTCTAATAGGATTGTATAAACATATTCCTATTTCAATAGGAT    14472
HEINZ       TGTATACACCTATTCCAATTCTAATAGGATTGTATAAACATATTCCTATTTCAATAGGAT    14483
            ************************************************************

LA2093      TATATAAACCTATTGCTATTCTAACAAACTTTAAGCATACGGAATCTTGTCATTCTATAT    14504
E42         TATATAAACCTATTGCTATTCTAACAAACTTTAAGCATACGGAATCTTGTCATTCTATAT    14532
HEINZ       TATATAAACCTATTGCTATTCTAACAAACTTTAAGCATACGGAATCTTGTCATTCTATAT    14543
            ************************************************************

LA2093      TGAATTCAAAGGAAGGTAATCTTTTAGAATTTACAAAATAAGGCCAATCTTTGGGGTAAT    14564
E42         TGAATTCAAAGGAAGGTAATCTTTTAGAATTTACAAAATAAGGCCAATCTTTGGGGTAAT    14592
HEINZ       TGAATTCAAAGGAAGGTAATCTTTTAGAATTTACAAAATAAGGCCAATCTTTGGGGTAAT    14603
            ************************************************************

LA2093      CCAATCAGGTCTACAAAGCAGGATGGAGGAACCAAATTTAAAAGAGGGAATAACTTAAGC    14624
```

```
E42         CCAATCAGGTCTACAAAGCAGGATGGAGGAACCAAATTTAAAAGAGGGAATAACTTAAGC     14652
HEINZ       CCAATCAGGTCTACAAAGCAGGATGGAGGAACCAAATTTAAAAGAGGGAATAACTTAAGC     14663
            ************************************************************

LA2093      ATAAGAATTTGGAGATATATCAATGTGAATTGTGCAGTAGAACATTTTGCAAGGCCACAT     14684
E42         ATAAGAATTTGGAGATATATCAATGTGAATTGTGCAGTAGAACATTTTGCAAGGCCACCT     14712
HEINZ       ATAAGAATTTGGAGATATATCAATGTGAATTGTGCAGTAGAACATTTTGCAAGGCCACCT     14723
            ********************************************************** *

LA2093      GACGCTTGCTTCCTAAAATCAGAAAATGAACTGGCCAGAATTTGAGTCTAGTTGAGTGT     14744
E42         GACGCTTGCTTCCTAAAATCAGAAAATGAACTGGCCAGAATTTGAGTCTAGTTGAGTGC     14772
HEINZ       GACGCTTGCTTCCTAAAATCAGAAAATGAACTGGCCAAAATTTGAGTCTAGTTGAGTGT     14783
            ************************************* *********************

LA2093      CTAAGGAAGAAAGAAAATTTACGGCAGTTCCTATCTTAACAACAAAAAGAAAAAAGCAAA     14804
E42         CTAAGGAAGAAAGAAAATTTACGGCAGTTCCTATCTTAACAACAAAAAGAAAAAAGCAAA     14832
HEINZ       CTAAGGAAGAAAGAAAATTTACGGCAGTTCCTATCTTAACAACAAAAAGAAAAAAGCAAA     14843
            ************************************************************

LA2093      TACCGGAATATCTACTCTGGATTACATCTTTGAATATCATGTACCAGGGAAATCATTTGA     14864
E42         TACCGGAATATCTACTCTGGATTACATCTTTGAATATCATGTACCAGGGAAATCATTTGA     14892
HEINZ       TACCGGAATATCTACTCTGGATTACATCTTTGAATATCATGTACCAGGGAAATCATTTGA     14903
            ************************************************************

LA2093      GGAAGGATAAACTCTTCGAAGTGAACTAAGCAACTATTTAAGACATATGCAAAGAAATTC     14924
E42         GGAAGGATAAACTCTTCGAAGTGAACTAAGCAACTATTTAAGACATATGCAAAGAAATTC     14952
HEINZ       GGAAGGATAAACTCTTCGAAGTGAACTAAGCAACTATTTAAGACATATGCAAAGAAATTC     14963
            ************************************************************

LA2093      TACCAGTAATTAAAGATGTCCCAAAAGAAAAGATTTTTAAATTAAAACTAACGAATAAAA     14984
E42         TACCAGTAATTAAAGATGTCCCAAAAGAAAAGATTTTTAAATTAAAACTAATGAATAAAA     15012
HEINZ       TACCAGTAATTAAAGATGTCCCAAAAGAAAAGATTTTTAAATTAAAACTAATGAATAAAA     15023
            *************************************************** ********

LA2093      TCATTCCATATGAATATATATCAAATGCCTAGATTCTCTCTAGCATGAAATCCATCAAAA     15044
E42         TCATTCCATATGAATATATATCAAATGCCTAGATTCTCTCTAGCATGAAATCCATCAAAA     15072
HEINZ       TCATTCCATATGAATATATATCAAATGCCTAGATTCTCTCTAGCATGAAATCCATCAAAA     15083
            ************************************************************

LA2093      ACTGCTTAGACTGTAGTAAATCATTTTTATCCAAATTAACTCTTCTAAAATCAGCAGCAT     15104
E42         ACTGCTTAGACTGTAGTAAATCATTTTTATCCAAATTAACTCTTCTAAAAGCAGCAGCAT     15132
HEINZ       ACTGCTTAGACTGTAGTAAATCATTTTTATCCAAATTAACTCTTCTAAAATCAGCAGCAT     15143
            ************************************************** *********

LA2093      CAACAGGCAAAACTACAACTAAACTATGAAATTAATATCAAAATGCGGGATACAAGATGA     15164
E42         CAACAGGCAAAACTACAACTAAACTATGAAATTAATATCAAAATGCGGGATACAAGATGA     15192
HEINZ       CAACAGGCAAAACTACAACTAAACTATGAAATTAATATCAAAATGCGGGATACAAGATGA     15203
            ************************************************************

LA2093      CCAAACAAATTAAGGAAATTAATCACTATAACTAACACATACCTCTTTTCCGGTGAACAA     15224
E42         CCAAACAAATTAAGGAAATTAATCACTATAACTAACACATACCTCTTTTCCGGTGAACAA     15252
HEINZ       CCAAACAAATTAAGGAAATTAATCACTATAACTAACACATACCTCTTTTCCGGTGAACAA     15263
            ************************************************************

LA2093      AAGGAACAACAAAGAAAGTGAATACTCCCTAACGTACTCAAAATTATTCTACACCAACTT     15284
E42         AAGGAACAACAAAGAAAGTGAATACTCCCTAACGTACTCAAAATTATTCTACACCAACTT     15312
HEINZ       AAGGAACAACAAAGAAAGTGAATACTCCCTAACGTACTCAAAATTATTCTACACCAACTT     15323
            ************************************************************

LA2093      TCTACTATAGTAGAAGCATGGGTGGCAGCACTTGGTATCAACATGGCTTGTTTTTTGTCA     15344
E42         TCTACTATAGTAGAAGCATGGGTAGCAGCACTTGGTATCAACATGGCTTGTTTTTTGTCA     15372
HEINZ       TCTACTATAGTAGAAGCATGGGTAGCAGCACTTGGTATCAACATGGCTTGTTTTTTGTCA     15383
            ********************** *************************************

LA2093      TTAAAAATTAAGACAAGGGTAATTTGAACTTTTCACTGTAACTTATATGTACGTGTCTTT     15404
E42         TTAAAAATTAAGACAAGGGTAATTTTAACTTTTCACTGTAACCTATATGTACGTGTCTTT     15432
HEINZ       TTAAAAATTAAGACAAGGGTAATTTGAACTTTTCATTGTAACTTATATGTACGTGTCTTT     15443
            ************************* ********* ****** *****************

LA2093      CTCTCAGCATTTGGAAAGCCACGTAATGTGCCTAGAGGCGGATCTAGAATTCGGAGGCTG     15464
E42         CTCTCAGCATTTGGAAAGCCACGTAATGTGCCTAGAGGCGGATCTAGAATTCGGAGGCTG     15492
HEINZ       CTCTCAGCATTTGGAAAGCCACGTAATGTGCCTAGAGGCGGATCTAGAATTCGGAGGCTG     15503
```

```
            ************************************************************

LA2093      GGGGTGCCATATCATCTAAATTGTAAACGGGTTGGTTCCTTCAATTTTAGGTTATGATTA     15524
E42         GGGGTGCCATATCATCTAAATTGTAAACGGGTTGGTTCCTTCAATTTTAGGTTATGAGTA     15552
HEINZ       CGGGTGCCATATCATCTAAATTGTAAACGGGTTGGTTCCTTCAATTTTAGGTTATGATTA     15563
             ******************************************************** **

LA2093      GGATTATTTAGTTTTTTACGATTTCTATAAATAAATTGATCAAATATACATATTTAGTAA     15584
E42         GGATTA-TTAGTTTTTTACGATTTCTATAAATAAATTGATCAAATATACATATTTAGTAA     15611
HEINZ       GGATTATTTAGTTTTTTACGATTTCTATAAATAAATTGATCAAATATACATATTTAGTAA     15623
            ****** *****************************************************

LA2093      TGTTTTTTTTTCTTTTAAATATTTTGATACTAAAGATATACAAATAAATAATTAGTTTTT     15644
E42         TGTTTTTTTTTCTTTTAAATATTCTGATACTAAAGATATACAAATAAATAATTAGTTTTT     15671
HEINZ       TG-TTTTTTTTCTTTTAAATATTCTGATACTAAAGATATACAAATAAATAATTAGTTTTT     15682
            ** ******************** ************************************

LA2093      CATAATTTTTTTTAGGAATAAGAGACCTTATTTGTAACATTGCGAAAATAAGAGATTTTT     15704
E42         CATAATTTTTTTTAGGAATAAGAGACCTTATTTGTAACATTGCGAAAATAAGAGATTTTT     15731
HEINZ       CATAATTTTTTTTAGGAATAAGAGACCTTATTTGTAACATTGCGAAAATAAGAGATTTTT     15742
            ************************************************************

LA2093      TTCACCTCAAAAATTCAATTTGTACACGTCATCTACATTCTAATACCACATAGTTGAGAA     15764
E42         TTCACCTCAAAAATTCAATTTGTACACGTCATCTACATTCTAATACCACATAGTTGAGAA     15791
HEINZ       TTCACCTCAAAAATTCAATTTGTACACGTCATCTACATTCTAATACCACATAGTTGAGAA     15802
            ************************************************************

LA2093      GAAAAAAATTACAGCTTTAATCCAGTCATCACCACTTAGTGTGACATTAAAAATTGAAAA     15824
E42         GAAAAAAATTACAGCTTTAATCCAGTCATCACCACTTAGTGTGACATTAAAAATTGAAAA     15851
HEINZ       GAAAAAAATTACAGCTTTAATCCAGTCATCACCACTTAGTGTGACATTAAAAATTGAAAA     15862
            ************************************************************

LA2093      AAAAA----AAATTTCAAAATCTTTAACATAATGATATCCATCATTTCAAAAAAAAAATAT     15880
E42         AAAAAATATAAATTTCAAAATCTTTAACATAATGATATCCATCATTTCAAAAAAAAAATAT     15911
HEINZ       AAAAA--TTAAATTTCAAAATCTTTAACATAATGATATCCATCATTTCAAAAAAAAAATAT     15920
            *****    ***************************************************

LA2093      TTAAAAATACTACTTAAACATATTTTTCTTAATATTTATGTGACTTGTAAAATTTAATAT     15940
E42         TTAAAAATACTACTTAAACATATTTTTCTTAATATTTATGTGACTTGTAAAATTTAATAT     15971
HEINZ       TTAAAAATACTACTTAAACATATTTTTCTTAATATTTATGTGACTTGTAAAATTTAATAT     15980
            ************************************************************

LA2093      TAATCTATAATTAATATAGAATTTCACGCAATATACACATCAAATTAACGATAGATAAAT     16000
E42         TAATCTATAATTAATATAGAATTTCACGCAATCTACACATCAAATTAAAGATAGATAAAT     16031
HEINZ       TAATCTATAATTAATATAGAATTTCACGCAATATACACATCAAATTAACGATAGATAAAT     16040
            ******************************** *************** ***********

LA2093      AAGTAACGACTAGCAAATAAAGGAACTAATTGAAGATGGCAAGAAACTGAAACAAAGACA     16060
E42         AAGTAACGACTAGCAAATAAAGGAACTATTTGAAGATGGCAAGAAACTGAAACAAAGACA     16091
HEINZ       AAGTAACGACTAGCAAATAAAGGAACTAATTGAAGATGGCAAGAAACTGAAACAAAGACA     16100
            **************************** *******************************

LA2093      AAACAAAAAGAGAGGGAAAAAAAAGGGAGCTCAAAAAGGATACAATAATGATAGTGCCCT     16120
E42         AAACAAAAAGAGAGGGAAAAAAAAGGGAGCTCAAAAAGGATACAATAATGATAGTGCCCT     16151
HEINZ       AAACAAAAAGAGAGGGAAAAAAAAGGGAGCTCAAAAAGGATACAATAATGATAGTGCCCT     16160
            ************************************************************

LA2093      CATGGAGGTCCGAACTCTCGATGCACTCATTGAGACTTGCGACCTTCGCCAGTTGAATAT     16180
E42         CATGGAGGTCCGAACTCTCGATGCACTCATTGAGATTTGCGACCTTCGCCAGTTGAATAT     16211
HEINZ       CATGGAGGTCCGAACTCTCGATGCACTCATTGAGACTTGCGACCTTCGCCAGTTGAATAT     16220
            *********************************** ************************

LA2093      TCAACTGATTGTTCGCTGGGTGGCAAATGTAATATATGTATTAAATATGTCATACTTATA     16240
E42         TCAACTGATTGTTCGCTGGGTGGCAAATGTAATATATGTATTAAATATGTCATACTTATA     16271
HEINZ       TCAACTGATTGTTCGCTGGGTGGCAAATGTAATATATGTATTAAATATGTCATACTTATA     16280
            ************************************************************

LA2093      CATATATATACAACGTTTAATCGAGGCCATTGGGTGCCGTTGCAGCCCACCCAAACAGCT     16300
E42         CATATATATACAACGTTTAATCGAGGCCATTGGGTGCCGTTGCAGCCCACCCAAACAGCT     16331
HEINZ       CATATATATACAACGTTTAATCGAGGCCATTGGGTGCCGTTGCAGCCCACCCAAACAGCT     16340
            ************************************************************
```

```
LA2093      AGATCCGCCCCTGAATGTGACAATTTCCTATTTTTCTTCTCCAAATCTAGAATCTTCCAA     16360
E42         AGATCCGCCCCTGAATGTGACAATTTCCTATTTTTCTTCTCCAAATCTAGAATCTTCCAA     16391
HEINZ       AGATCCGCCCCTGAATGTGACAATTTCCTATTTTTCTTCTCCAAATCTAGAATCTTCCAA     16400
            ************************************************************

LA2093      AACTTACTCTCTGTTCTCTCCAAAAATGGCATCAGAGACATAGAGGAGACCACCTACCGC     16420
E42         AACTTACTCTCTGTTCTCTCCAAAAATGGCATCAGAGACATAGAGGAGACCACCTACCGC     16451
HEINZ       AACTTACTCTCTGTTCTCTCCAAAAATGGCATCAGAGACATAGAGGAGACCACCTACCGC     16460
            ************************************************************

LA2093      TGAGTTTCCTGCTTTCTTACTACTTTCCACAAAATCATTGTTTAGAGAACCATCAATTG     16480
E42         CGAGTTTCCTGCTTTCTTACTACTTTCCACAAAATCATTGTTTAGAGAACCATCAATTG     16511
HEINZ       TGAGTTTCCTGCTTTCTTACTACTTTCCACAAAATCATTGTTTAGAGAACCATCAATTG     16520
             ***********************************************************

LA2093      AGTAGCTAAGTGTCTTATGTGCAACTTTTTACAAAGTATTATTTTAGTTCTATTTTACTT     16540
E42         AGTAGCTAAGTGTCTTATGTGCAACTTTTTACAAAGTATTATTTTAGTTCTATTTTACTT     16571
HEINZ       AGTAGCTAAGTGTCTTATGTGCAACTTTTTACAAAGTATTATTTTAGTTCTATTTTACTT     16580
            ************************************************************

LA2093      TCCTTATATACTTTGTTTTTGTTGTTTCTGGTTCTAATATATTAGCGGGTCGTTGCATTT     16600
E42         TCCTTATATACTTTGTTTTTGTTGTTTCTGGTTCTAATATATTAGCGGGTCGTTGCATTT     16631
HEINZ       TCCTTATATACTTTGTTTTTGTTGTTTCTGGTTCTAATATATTAGCGGGTCGTTGCATTT     16640
            ************************************************************

LA2093      AAGTTACTTATAGTTGTATGTGTGTAGTTTTCCGTCACTCTAAATTTTATGAGAAGCAAA     16660
E42         AAGTTACTTATAGTTGTATGTGTGTAGTTTTCCGTCACTCTAAATTTTATGAGAAGCAAA     16691
HEINZ       AAGTTACTTATAGTTGTATGTGTGTAGTTTTCCGTCACTCTAAATTTTATGAGAAGCAAA     16700
            ************************************************************

LA2093      AATTAATTATATCTTCCTACTAATTTGATTTGTAAAAATAGATTTTGCTTGTGAAGCTTG     16720
E42         AATTAATTATATCTTCCTACTAATTTGATTTGTAAAAATAGATTTTGCTTGTGAAGCTTG     16751
HEINZ       AATTAATTATATCTTCCTACTAATTTGATTTGTAAAAATAGATTTTGCTTGTGAAGCTTG     16760
            ************************************************************

LA2093      ATTTTGAAAATAAGCTCTTAAGTACTCACCATTGTTGTCTTCTTTTCAAATTGTGCTACT     16780
E42         ATTTTGAAAATAAGCTCTTAAGTACTCACCATTGTTGTCTTCTTTTCAAATTGTGCTACT     16811
HEINZ       ATTTTGAAAATAAGCTCTTAAGTACTCACCATTGTTGTCTTCTTTTCAAATTGTGCTACT     16820
            ************************************************************

LA2093      CAGGTTGTCATTTCACCTTTTTGGTAGTTGTTCTTGCAAATTATAGACGAGCATTTTGAT     16840
E42         CAGGTTGTCATTTCACCTTTTTGGTAGTTGTTCTTGCAAATTATAGACGAGCATTTTGAT     16871
HEINZ       CAGGTTGTCATTTCACCTTTTTGGTAGTTGTTCTTGCAAATTATAGACGAGCATTTTGAT     16880
            ************************************************************

LA2093      CATCCAATTTATTTATTATAGCCATTAACTATTTTACTACTTTCAATGTTACTATGTATG     16900
E42         CATCCAATTTATTTATTATAGCCATTAACTATTTTACTACTTTCAATGTTACTATGTATG     16931
HEINZ       CATCCAATTTATTTATTATAGCCATTAACTATTTTACTACTTTCAATGTTACTATGTATG     16940
            ************************************************************

LA2093      TGCCTTATTTCAGGTCAAAACTGAAATTCTGCAAAAGTAAGTTATAGGCCTAAAATTGAG     16960
E42         TGCCTTATTTCAGGTCAAAACTGAAATTCTGCAAAAGTAAGTTATAGGCCTAAAATTGAG     16991
HEINZ       TGCCTTATTTCAGGTCAAAACTGAAATTCTGCAAAAGTAAGTTATAGGCCTAAAATTGAG     17000
            ************************************************************

LA2093      TTGGTTAGTTTTTGCGCTAGATATCTATGTTAATTTACAATTCACTAAATTACCCATAGG     17020
E42         TTGGTTAGTTTTTGCGCTAGATATCTATGTTAATTTACAATTCACTAAATTACCCATAGG     17051
HEINZ       TTGGTTAGTTTTTGCGCTAGATATCTATGTTAATTTACAATTCACTAAATTACCCATAGG     17060
            ************************************************************

LA2093      TTTTTGGTACTTGATGGTACTAATTCATCTACTCAAAAGCATCAACTCCTTTAGCATATA     17080
E42         TTTTTGGTACTTGATGGTACTAATTCATCTACTCAAAAGCATCAACTCCTTTAGCATATA     17111
HEINZ       TTTTTGGTACTTGATGGTACTAATTCATCTACTCAAAAGCATCAACTCCTTTAGCATATA     17120
            ************************************************************

LA2093      TTTTCTACTAACTTAATGGTTGTATCTGTTGTGTAATTAGATCGCAAATATGTTTCCCGC     17140
E42         TTTTCTACTAACTTAATGGTTGTATCTGTTGTGTAATTAGATCGCAAATATGTTTCCCGC     17171
HEINZ       TTTTCTACAAACTTAATGGTTGTATCTGTTGTGTAATTAGATCGCAAATATGTTTCCCGC     17180
            ******** ***************************************************

LA2093      AATTTCATAGATTACTTATTCCTGATTTATTCTTATACCTAATTAAGATACATAGAATGC     17200
E42         AATTTCATAGATTACTTATTCCTGATTTATTCCTATACCTAATTAAGATACATAGAATGC     17231
```

```
HEINZ        AATTTCATAGATTACTTATTCCTGATTTATTCTTATACCTAATTAAGATACATAGAATGC    17240
             ******************************** ***************************

LA2093       ATCAAAATTATCTTTAATCTTAAGATCGTAAATATGTGAGGTGGAAAGTTAATGTACCAA    17260
E42          ATCAAAATTATCTTTAATCTTAAGATCGTAAATATGTGAGGTGGAAAGTTAATGTACCAA    17291
HEINZ        ATCAAAATTATCTTTAATCTTAAGATCGTAAATATGTGAGGTGGAAAGTTAATGTACCAA    17300
             ************************************************************

LA2093       AATGTCTTTAATCTTATAAATGTGTTTGGTGTTTCCTTAGAAAGAAATATTTAAATCTGT    17320
E42          AATGTCTTTAATCTTATAAATGTGTTTGGTGTTTCCTTAGAAAGGAATATTTAAATCTGT    17351
HEINZ        AATGTCTTTAATCTTATAAATGTGTTTGGTGTTTCCTTAGAAAGGAATATTTAAATCTGT    17360
             ******************************************** ***************

LA2093       CAATTCACTAGCCTTTTGATCAATAATGCGCAAGAACGCAAAAAGAAAAAAACTACCTCA    17380
E42          CAATTCACTAGCCTTTTGATCAATAATGCGCAAGAACGCAAAAAGAAAAAAACTACCTCA    17411
HEINZ        CAATTCACTAGCCTTTTGATCAATAATGCGCAAGAACGCAAAAAGAAAAAAACTACCTCA    17420
             ************************************************************

LA2093       TTGATTCTTTGTATTAGTGGGTCATAGATTGCACAAGTAGTGCAACATGAATTACCCGAT    17440
E42          TTGATTCTTTGTATTAGTGGGTCATAGATTGCACAAGTAGTGCAACATGAATTACCCGAT    17471
HEINZ        TTGATTCTTTGTATTAGTGGGTCATAGATTGCACAAGTAGTGCAACATGAATTACCCGAT    17480
             ************************************************************

LA2093       TGGTTGAAAGTACTATATCATCCTATTTTAGATGAATAAACCGTAATATTTCACTGGTCT    17500
E42          TGGTTGAAAGTACTATATCATCCTATTTTAGATGAATAAACCGTAATATTTCATTGGTCT    17531
HEINZ        TGGTTGAAAGTACTATATCATCCTATTTTAGATGAATAAACCGTAATATTTCACTGGTCT    17540
             ***************************************************** ******

LA2093       AAGCACCATGAGGCCGAAAATAATTAGGATTCAACAAAGCGATGAAATCTTAAAGTTCTG    17560
E42          AAGCACCATGAGGCCGAAAATAATTAGGATTCAACAAAGCGATGAAATCTTAAAGTTCTG    17591
HEINZ        AAGCACCATGAGGCCGAAAATAATTAGGATTCAACAAAGCGATGAAATCTTAAAGTTCTG    17600
             ************************************************************

LA2093       -TTTTTTCAAGTGTCATTAGAGGAATATTCTGTCAATCTCACAAGATCTATCAAGGTTTT    17619
E42          TTTTTTTCAAGTGTCATTAGAGGAATATTCTGTCAATCTCACAAGATCTATCAAGGTTTT    17651
HEINZ        -TTTTTTCAAGTGGCATTAGAGGAATATTCTGTCAATCTCACAAGATCTATCAAGGTTTT    17659
              ************ **********************************************

LA2093       GTCCAGGTATATAATCGATCAAGCATTTGGGAGGAGGAAGAGATATTTCCCAAAGATTGA    17679
E42          GTCCAGGTATATAATCGATCAAGCATTTGGGAGGAGGAAGAGATATTTCCCAAAGATTGA    17711
HEINZ        GTCCAGGTATATAATCGATCAAGCATTTGGGAGGAGGAAGAGATATTTCCCAAAGATTGA    17719
             ************************************************************

LA2093       TTAATAGTAATGAAAGCTAATTTATCATTACATCAGGTTAATTTCACTTTTAACCTGAAA    17739
E42          TTAATAGTAATGAAAGCTATTTTATCATTACATCAGGTTAATTTCACTTTTAACCTGAAA    17771
HEINZ        TTAATAGTAATGAAAGCTAATTTATCATTACATCAGGTTAATTTCACTTTTAACCTGAAA    17779
             ******************* ****************************************

LA2093       AGGATGTTATTTTAGATTGCTAGTTGTTAAAAAAAAAATTATGTTCATCTATCCATTTCA    17799
E42          AGGATGTTATTTTAGATTGCTAGTTGTTAAAAAAAAAATTCTGTTCATCTATCCATTTCA    17831
HEINZ        AGGATGTTATTTTAGATTGCTAGTTGTTAAAAAAAAAATTATGTTCACCTATCCATTTCA    17839
             **************************************** ****** ************

LA2093       TTTGTTTGGCCACTACATTGTCTTCTAAGGATTCGAAATGTCTTTTTGGATGAAATGATG    17859
E42          TTTGTTTGGCCACTACATTGTCTTCTAAGGATTCGAAATGTCTTTTTGGATGAAATGATG    17891
HEINZ        TTTGTTTGGCCACTACATTGTCTTCTAAGGATTCGAAATGTCTTTTTGGATGAAATGATG    17899
             ************************************************************

LA2093       CCAAACTACTGCCTGCATGTACGCAGTGGAGTGCCAACATTGTCTAGACAAAAGTGTTCA    17919
E42          CCAAACTACTGCCTGCATGTACGCAGTGGAGTGCCAACATTGTCTAGACAAAAGTGTTCA    17951
HEINZ        CCAAACTACTGCCTGCATGTACGCAGTGGAGTGCCAACATTGTCTAGACAAAAGTGTTCA    17959
             ************************************************************

LA2093       CATTTTTTAGCATAAAATTTATTGAGCCCTTTTGGATATATATCTGCTTTGGGCTCTCAT    17979
E42          CATTTTTTAGCATAAAATTTATTGAGCCCTTTTGGATATATATCTGCTTTGGGCTCTCAT    18011
HEINZ        CATTTTTTAGCATAAAATTTATTGAGCCCTTTTGGATATATATCTGCTTTGGGCTCTCAT    18019
             ************************************************************

LA2093       AGAACACTTGAGGCTCTTCATGGGATGAAATTTATATCCTCGTGTGGTAAAGAGATTCAT    18039
E42          AGAACACTTGAGGCTCTTCATGGGATGAAATTTATATCCTCATGTGGTAAAGAGATTCAT    18071
HEINZ        AGAACACTTGAGGCTCTTCATGGGATGAAATTTATATCCTCATGTGGTAAAGAGATTCAT    18079
             ***************************************** ******************
```

```
LA2093       TTTCTAACAGTAGGTAATGCTTGTGAGATCCTCGATAGAAATTATCAATCTTGTGCTTGG     18099
E42          TTTCTAACAGTAGGTAATGCTTGTGAGATCCTCGATAGAAATTATCAATCTTGTGCTTGG     18131
HEINZ        TTTCTAACAGTAGGTAATGCTTGTGAGATCCTCGATAGAAATTATCAATCTTGTGCTTGG     18139
             ************************************************************

LA2093       TGAATTTTGATTTGTTCTAACTACGATGCTAACGAAATAAATTGGTTGTAGTGTTGTTTC     18159
E42          TGAATTTTGATTTGTTCTAACTACGATGCTAACGAAATAAATTGGTTGTAGTGTTGTTTC     18191
HEINZ        TGAATTTTGATTTGTTCTAACTACGATGCTAACGAAATATATTGGTTGTAGTGTTGTTTC     18199
             *************************************** ********************

LA2093       TATTGCTTATGTGTACTTTTTTCAACATTTGATTCTTGTAGATAGAAAATGAGACAACTT     18219
E42          TATTGCTTATGTGTACTTTTTTCAACATTTGATTCTTGTAGATAGAAAATGAGACAACTT     18251
HEINZ        TATTGCTTATGTGTACTTTTTTCAACATTTGATTCTTGTAGATAGAAAATGAGACAACTT     18259
             ************************************************************

LA2093       TTTTTCCGCAAATATGAGATTGTGAGGCCCTCTTATCAGTTTAGACCATTCAATCTTGGA     18279
E42          TTTTTCCGCAAATATGAGATTGTGAGGCCCTCTTATCAGTTTAGACCATTCAATCTTGGA     18311
HEINZ        TTTTTCCGCAAATATGAGATTGTGAGGCCCTCTTATCAGTTTAGACCATTCAATCTTGGA     18319
             ************************************************************

LA2093       TTTATGTATGTCAATAAATAGTACGAGTTCCACGCAATGTAGTTTGAGTAGTGTTGAAGC     18339
E42          TTTATGTATGTCAATAAATAGTACGAGTTCCACGCAATGTAGTTTGAGTAGTGTTGAAGC     18371
HEINZ        TTTATGTATGTCAATAAATAGTACGAGTTCCACGCAATGTAGTTTGAGTAGTGTTGAAGC     18379
             ************************************************************

LA2093       CTTTAGATATTCTTACATCTAAATTAACATATGCGGTGTATTCTTAGGAGAGGTTCTTTA     18399
E42          CTTTAGATATTCTTACATCTAAATTAACAAATGCGGTGTATTCTTAGGAGAGGTTCTTTA     18431
HEINZ        CTTTAGATATTCTTACATCTAAATTAACATATGCGGTGTATTCTTAGGAGAGGTTCTTTA     18439
             ***************************** ******************************

LA2093       CTTTTGCCAGCAGATATTTAGATATTTTTGTTTATAAATGTGTATTTTCTTTTCTCTCGT     18459
E42          CTTTTGCCAGCAGATATTTAGATATTTTTGTTTATAAATGTGTATTTTCTTTTCTCTCGT     18491
HEINZ        CTTTTGCCAGCAGATATTTAGATATTTTTGTTTATAAATGTGTATTTTCTTTTCTCTCGT     18499
             ************************************************************

LA2093       CATTTATTCCTCTCATTTGTTTTGCCAAAAGATTTGGCTGTTATCTTTGGTCCCTGAACT     18519
E42          CATTTATTCCTCTCATTTGTTTTGCCAAAAGATTTGGCTGTTATCTTTGGTCCCTGAAAT     18551
HEINZ        CATTTATTCCTCTCATTTGTTTTGCCAAAAGATTTGGCTGTTATCTTTGGTCCCTGAACT     18559
             ********************************************************** *

LA2093       ATCTTTTTGAAGTGTCAAATTGAAGAGAGCTCCAATGCTTTAAAAAAGTACACGACCAGT     18579
E42          ATCTTTTTGAAGTGTCAAATTGAAGAGAGCTCCAATGCTTTAAAAAAGTACACGACCAGT     18611
HEINZ        ATCTTTTTGAAGTGTCAAATTGAAGAGAGCTCCAATGCTTTAAAAAAGTACACGACCAGT     18619
             ************************************************************

LA2093       CGTCCAAACATTTCTCCCACTTCAACATAAAGCCTCCAACATATATTTTGCCAGTCTTGA     18639
E42          CGTCCAAACATTTCTCCCACTTCAACATAAAGCCTCCAACATATATTTTGCCAGTCTTGA     18671
HEINZ        CGTCCAAACATTTCTCCCACTTCAACATAAAGCCTCCAACATATATTTTGCCAGTCTTGA     18679
             ************************************************************

LA2093       TGTCACAAAATTACATAGGTAATTAAGAAAATCAAGATCACTACCACCAACATAGTAACG     18699
E42          TGTCACAAAATTACATAGGTAATTAAGAAAATCAAGATCACTACCACCAACATAGTAACG     18731
HEINZ        TGTCACAAAATTACATAGGTAATTAAGAAAATCAAGATCACTACCACCAACATAGTAACG     18739
             ************************************************************

LA2093       GACTGTGTTACTATTTCACCTTTTGTGACACCAGCTTCTGTGACGATGCAAGGGCTATAG     18759
E42          GACTGTGTTACTATTTCACCTTTTGTGACACCAGCTTCTGTGACGATGCAAGGGCTATAG     18791
HEINZ        GACTGTGTTACTATTTCACCTTTTGTGACACCAGCTTCTGTGACGATGCAAGGGCTATAG     18799
             ************************************************************

LA2093       TTCAGTTGGTGTGTACATATACTGACAGTCTTTTACTATGACACGATCTTCAACTACTAC     18819
E42          TTCAGTTGGTGTGTACATATACTGACAGTCTTTTACTATGACACGATCTTCAACTACTAC     18851
HEINZ        TTCAGTTGGTGTGTACATATACTGACAGTCTTTTACTATGACACGATCTTCAACTACTAC     18859
             ************************************************************

LA2093       TTTATGCTGATTTTTCAGTTGACGCCTTACAAGTTTTTCTAAATTTTGTTAGCATCATAT     18879
E42          TTTATGCTGATTTTTCAGTTGACGCCTTACAAGTTTTTCTAAATTTTGTTAGCATCATAT     18911
HEINZ        TTTATGCTGATTTTTCAGTTGACGCCTTACAAGTTTTTCTAAATTTTGTTAGCATCATAT     18919
             ************************************************************

LA2093       ATGTTGGGCCCATACACACAAGGGCAACCTCTATCTAGTAATTCTGAGAAATGGCAGCAA     18939
```

```
E42         ATGTTGGGCCCATACACACAAGGGCAACCTCTATCTAGTAATTCTAAGAAATGGCAGCAA    18971
HEINZ       ATGTTGGGCCCATACACACAAGGGCAACCTCTATCTAGTAATTCTGAGAAATGGCAGCAA    18979
            ********************************************** *************

LA2093      AGAAACACTGTATTGAAGATAATCATAATAACGAGAACACATGACCCGTAAAGCAGACTC    18999
E42         AGAAACACTGTATTGAAGATAATCATAATAACGAGAACACATGACCCGTAA---------    19022
HEINZ       AGAAACACTGTATTGAAGATAATCATAATAACGAGAACACATGACCCGTAAAGCAGACTC    19039
            ***************************************************

LA2093      GAACAAATAGCCTCCAAAAGATTAAGACATGCCAAGGTTCAAATGGACTGATTAACAGTA    19059
E42         -AACAAATAGCCTTCAAAAGATCAAGACATGCCAAGGTTCAAATGGACTGATTAACAGTA    19081
HEINZ       GAACAAATAGCCTTCAAAAGATTAAGACATGCCAAGGTTCAAATGGACTGATTAACAGTA    19099
             ************ ******** *************************************

LA2093      AATAGCAAAAGCTTACTGAAAACTAACAAGGGGCAAATTTAATATCAACAAAGCTTTATC    19119
E42         CATAGCAAAAGCTTACTGAAAACTAACAAGGGGCAAATTTAATATCAACAAAGCTTTATC    19141
HEINZ       AATAGCAAAAGCTTACTGAAAACTAACAAGGGGCAAATTTAATATCAACAAAGCTTTATC    19159
             ***********************************************************

LA2093      ATCACTTTAAAACCTACAGAATCAACAGGAATTCAGGAAAAAACTTAACAAAATGCATCC    19179
E42         ATCACTTTAAAACCTACAGAATCAACAGGAATTCAGGAAAAAACTTAACAAAATGCATCC    19201
HEINZ       ATCACTTTAAAACCTACAGAATCAACAGGAATTCAGGAAAAAACTTAACAAAATGCATCC    19219
            ************************************************************

LA2093      GAAGTCAATACAACAGACTGACTGATCAACAAAGAAAAAGAAAAAGAAATGAGATGTAAC    19239
E42         GAAGTCAATACAACACACTGACTGATCAACAAAGAAAAAGAAAAAGAAATGAGATGTAAC    19261
HEINZ       GAAGTCAATACAACACACTGACTGATCAACAAAGAAAAAGAAAAAGAAATGAGATGTAAC    19279
            *************** ********************************************

LA2093      CTTTTCCTGGGGCAAGACGAAGTTGAGAGATACTAAAATCTAGACACCAAACCTCTTGAA    19299
E42         CTTTTCCTGGGGCAAGACGAAGTTGAGAGATACTAAAATCTAGACACCAAACCTCTTGAA    19321
HEINZ       CTTTTCCTGGGGCAAGACGAAGTTGAGAGATACTAAAATCTAGACACCAAACCTCTTGAA    19339
            ************************************************************

LA2093      TTAGGGCTTGAAAACAGGATAAGGATCAGGGAAATTGAAATTTGAAACTCATATGCTGGA    19359
E42         TTAGGGCTTGAAAACAGGATAAGGATCAGGGAAATTGAAATTTGAAACTCATATGCTGGA    19381
HEINZ       TTAGGGCTTGAAAACAGGATAAGGATCAGGGAAATTGAAATTTGAAACTCATATGCTGGA    19399
            ************************************************************

LA2093      TAGATTCGAACGAATTCTCAGAGAAATCAGTCTCACCGCTGGGTCAAGAAGAAGAAGAAG    19419
E42         TAGATTCGAACGAATTCTCAGAGAAATCAGTCTCACCGCTGGGTCAAGAAGAAGAAGAAG    19441
HEINZ       TAGATTCGAACGAATTCTCAGAGAAATCAGTCTCACCGCTGGGTCAAGAAGAAGAAGAAG    19459
            ************************************************************

LA2093      ATGAGGGTCGGTCCAGTTGTGTATGGCTTCTCCGAATTGTATTCTTCTCTGTATTGGGTT    19479
E42         ATGAGGGTCGGTCCAGTTGTGTATGGCTTCTCCGAATTGTATTCTTCTCTGTATTGGGTT    19501
HEINZ       ATGAGGGTCGGTCCAGTTGTGTATGGCTTCTCCGAATTGTATTCTTCTCTGTATTGGGTT    19519
            ************************************************************

LA2093      TTGT        19483
E42         TTGT        19505
HEINZ       TTGT        19523
            ****
```

Solyc07g039220

```
E42       CAATAAGTAAACATTAAATACAAAGGCATTCCTACAGGATCAAGCACAAAGAAACCTCGC   60
HEINZ     CAATCAGTAAACATTAAATACAAAGGCATTCCTACAGGATCAAGCACAAAGAAACCTCGC   60
LA2093    CAATCAGTAAACATTAAATACAAAGGCATTCCTACAGGATCAAGCACAAAGAAACCTCGC   60
          **** *******************************************************

E42       AAACAACGACCTCTCTTTTCTGTCAAGAGAAAAGCAGTAATTAGATGAGAGAACGTGAGA   120
HEINZ     AAACAGCGACCTCTCTTTTCTGTCAAGAGAAAAGCAGTAATTAGATGAGAGAACCTGAGA   120
LA2093    AAACAGCGACCTCTCTTTTCTGTCAAGAGAAAAGCAGTAATTAGATGAGAGAGCCTGAGA   120
          ***** ********************************************** * *****

E42       TGTTGGGATATAGATATTTTCACTACTTTGTCAGCCTCTTTTTCTTCCACTTTAGGCTTG   180
HEINZ     TGTTGGGATATAGATATTTTCACTACTTTGTCAGCCTCTTCTTCTTCCACTTTAGGCTTG   180
LA2093    TGTTGGGATATAGATATTTTCACTACTTTGTCAGCCTCTTCTTCTTCCACTTTAGGCTTG   180
          **************************************** *******************

E42       TAAACAGACTTGAATCCTTTTAACCATTGTAAGAACCTCAGGTCTACATAATTATCAGAA   240
HEINZ     TAAACAGACTTTAATCCTTTTACCCATTGTAAGAACCTCAGGTCTACATAATTATCATAA   240
LA2093    TAAACAGACTTGAATCCTTTTACCCATTGTAAGAACCTCAGGTCTACATAATTATCATAA   240
          *********** ********** ********************************** **

E42       GCATCCACTATTTCAACAATATACCTTTCAGCTGAGAGATCCAATTTTTATATACCGCCC   300
HEINZ     GCATCCACTATTTCAACAATATACCTTTCAGCTGAGAGATCCAGTTTTTATATACCGCCC   300
LA2093    GCATCCACTATTTCAACAATATACCTTTCAGCTGAGAGATCCAGTTTTTATATACCGCCC   300
          ******************************************* ****************

E42       AGACTTCACCTTTTAAAGGGATGATCTTGTACACACCCCTCTTCACGTTCTGCTCCTAGT   360
HEINZ     AGACTTCACCTTTTAAAGGGATGATCTTGTACACACCCCTCTTCACGTTCTGCTCCTAGT   360
LA2093    AGACTTCACCTTTTAAAGGGATGATCTTGTACACACCCCTCTTCACGTTCTGCTCCTAGT   360
          ************************************************************

E42       ACATGTGAAAGGTTGTTGGTTACACTATATTTGTTCAGCTTTCTATTTTCACAATTAAAT   420
HEINZ     ACATGTGAAAGGTTGTTGGTTACACTATATTTGTTCAGCTTTCTATTTTCACAATTAAAT   420
LA2093    ACATGTGAAAGGTTGTTGTTTACACTATATTTGTTCAGCTTTCTATTTTCACAATTAAAT   420
          ****************** *****************************************

E42       AGTACACAACTAATTGTCATTGTTATATTATTCCACTGTATTGTACTTTTAGGAAGTGGA   480
HEINZ     AGTACACAACTAATTGTCATTGTTATATTATGCCACTGTATTGTACTTTTAGGAAGTGGA   480
LA2093    AGTACACAACTAATTGTCATTGTTATATTATGCCACTGTATTGTACTTTTAGGAAGTGGA   480
          ******************************* ****************************

E42       CACGCATAAAACCATGTCACATGCAATACAAACTCAGGGAGAAGATTGATCTTCTTTAAT   540
HEINZ     CACGCATAAAACCATGTCACATGCAATACAAACTCAGGGAGAAGATTGATCTTCTTTAAT   540
LA2093    CACGCATAAAACCATGTCACATGCAATACAAACTCAGGGAGAAGATTGATCTTCTTTAAT   540
          ************************************************************

E42       AGGTCATTGTACCGGGGAAACACATCTTCATTACTGCATATTTCTCAATACTGACCAATT   600
HEINZ     AGGTCATTGTACCGGGGAAACACATCTTCCTTACTGCATATTTCCCAATACTGACCAATT   600
LA2093    AGGTCGTTGTACCGGTGAAACACATCTTCCTTACTGCATATTTCCCAATACTGACCAATT   600
          ***** ********* ************* ************** ***************

E42       TGAAATATCTCCGGGGATCCCTCAGTAGCAAATCTGTAAAACACAGGTTCAGGAACCTGA   660
HEINZ     TGAAATATCTCCGGGGATCCCTCAGTAGCAAATCTGTAAAACACAGGTTCAGGAACCTGA   660
LA2093    TGAAATATCTCCGGGGATCCCTCAGTAGCAAATCTGTAAAACACAGGTTCAGGAACCTGA   660
          ************************************************************

E42       TTAGTACCGATGCAACCCAGTTTTCAGGAGAATTTACTGAATACAATAAATTGCCTCTCT   720
HEINZ     TTAGTACCGATGCAACCCAGTTTTCAGGAGAATTTACTGAATACAATAAATTGCCTCTCT   720
LA2093    TTAGTACCGATGCAACCCAGTTTTCAGGAGAATTTACTGAATACAATAAATTGCCTCTCT   720
          ************************************************************

E42       TTGGTCTATGGATGGAGAAGAAATATTTATTCGTTCAGCAGTTAAAGAGGCATGGTCTAG   780
HEINZ     TTGGTCTATGGATGGAGAAGAAATATTTATTCGTTCAGTAGTTAAAGAGGCATGGTCTAG   780
LA2093    TTGGTCTATGGATGGAGAAGAAATATTTATTCGTTCAGTAGTTAAAGAGGCATGGTCTAG   780
          ************************************** *********************

E42       TTCACAGGATCCTTCAGGAATACCATCCTTCTCCATCCCTGTCATCTTTATAGAAGGAAT   840
HEINZ     TTCACAGGATCCTTCAGGAATACCATCCTTCTCCATCCCTGTCATCTTTATAGAAGGAAT   840
LA2093    TTCACAGGATCCTTCAGGAATACCATCCTTCTCCATCCCTGTCATCTTTATAGAAGGAAT   840
          ************************************************************
```

```
E42        TCTATGAGAGAATCTAAACATATCCTTTGCTGGACTAGAAATAAGTCTCTTGCCTCTATG     900
HEINZ      TCTATGAGAGAATCTAAACATATCCTTTGCTGGACTAGAAATAAGTCTCTTGCCTCTATG     900
LA2093     TCTATGAGAGAATCTAAACATATCCTTTGCTGGACTAGAAATAAGTCTCTTGCCTCTATG     900
           ************************************************************

E42        AAAGAGACATGTGAAGCCTTTTAATTTATTCATGTACGCAACATGCACACTAATAGAATC     960
HEINZ      AAAGAGACATGTGAAGCCTTTTACTTTATTCAAGTACGCAACATGCACACTAATAGAATC     960
LA2093     AAAGAGACATGTGAAGCCTTTTACTTTATTCAAGTACGCAACATGCACACTAATAGAATC     960
           *********************** ******** ***************************

E42        ACCATAACCTGACAAGACCTGAACAAACTCGAAGTTGAATTTTTTATTACTCCCAAGATG     1020
HEINZ      ACCATAACCTGACAAGACCTAAACAAACTCGAAGTTGAATTTTTTATTACTCCCAAGATG     1020
LA2093     ACCATAACCTGACAAGACCTAAACAAACTCGAAGTTGAATTTTTTATTACTCCCAAGATG     1020
           ******************** ***************************************

E42        AGGACACCACTTCAGATCCAAATCTTTTAAGAAAGCCCAATTTATCCTTCCAATAGAAAT     1080
HEINZ      AGGACACCACTTCAGATCCAAATCTTTTAAGAAAGCCCAATTTATCCTTCCAATAGAAAT     1080
LA2093     AGGACACCACTTCAGATCCAAATCTTTTAAGAAAGCCCAATTTATCCTTCCAATAGAAAT     1080
           ************************************************************

E42        ATCTTTATTTCAGCGCAGATGTTTCCACTCATTGGACACACCAAACGTGAGAACATAGGA     1140
HEINZ      ATCTTTATTTCAGCGCAGATGTTTCCACTCATTGGACACACCAAACGTGAGAACATAGGA     1140
LA2093     ATCTTTATTTCAGCGCAGATGTTTCCACTCATTGGACACACCAAACGTGAGAACATAGGA     1140
           ************************************************************

E42        TGATCATCAAGGTCTTCTGAGTTTCCTAGTCTGAACCTACCACAAGAAGCAGGAAAGCCC     1200
HEINZ      TGATCATCAAGGTCTTCTGAGTTTCCTAGTCTGAACCTACCACAAGAAGAAGGAAAGCCC     1200
LA2093     TGATCATCAAGGTCTTCTGAGTTTCCTAGTCTGAACCTACCACAAGAAGAAGGAAAGCCC     1200
           ************************************************* **********

E42        TCAGATAGCCATTTTGCTTCATCTTCATTCAGCGGCTCTGGCTCTAACCATTTTATGCAC     1260
HEINZ      TCAGATAGCCATTTTGTTTCATCTTCATTCAGCGGCTCTGGCTCTAACCATTTTATGCAC     1260
LA2093     TCAGATAGCCATTTTGTTTCATCTTCATTCAGCGGCTCTGGCTCTAACCATTTTATGCAC     1260
           **************** *******************************************

E42        AACTTTAATGAAGGAGATAAAATCTTTCTAATGACGGGATAAAATCGTGGAATGACATCC     1320
HEINZ      AACTTTAATGAAGGAGATAAAATCTTTCTAATGACGGGATAAAATCGTGGAATGACATCC     1320
LA2093     AACTTTAATGAAGGAGATAAAATCTTTCTAATGACGGGATAAAATCGTGGAATGACATCC     1320
           ************************************************************

E42        ATAGTATCATAAACAGCCCATACATGCCCAACCTTAAAGCAAGTTTCTTCCGTTTCCATG     1380
HEINZ      ATAGTATCATAAACAGCCCATACATGCCCAACCTTAAAGCAAGTTTCTTCCGTTTCCTTG     1380
LA2093     ATAGTATCATAAACAGCCCATACATGCCCAACCTTAAAGCAAGTTTCTTCCGTTTCCTTG     1380
           ********************************************************* **

E42        TCAAAAACACTAAATATGTGCATCTTCATATTCAAATGTTTCGGACATAGCCAT   1434
HEINZ      TCAAAAACACT-AATATGTGCATCTTCATATTCAAATGTTTCGGACATAGCCAT   1433
LA2093     TCAAAAACACT-AATATGTGCATCTTCATATTCAAATGTTTCGGACATAGCCAT   1433
           *********** ******************************************
```

Solyc07g043560

```
LA2093      AAAAGGGCAACCAAAGGGAAGCTCAGTAAGTCAGTAACATTGTTGCCTGTATAGATTCAC   60
E42         CAAGGGGCAACCAAAGGGAAGCTCAGTAAGTCAGTAACATTGTTGCCGGTATAGATTCAC   60
HEINZ       AAAGGGGCAACCAAAGGGAAGCTCAGTAAGTCAGTAACATTGTTGCCTGTATAGATTCAC   60
             ** ******************************************* ************

LA2093      AGAAATTAGAAATGTGTAATCTAAGGGCAGGAGTGTATTCAAAGCTAACAAAATTCTATT   120
E42         AGAAATTAGAAATGTGTAATCTAACGGCAGGAGTGTATTCAAAGCTAACAAAATTCTATT   120
HEINZ       AGAAATTAGAAATGTGTAATCTAACGGCAGGAGTGTATTCAAAGCTAACAAAATTCTATT   120
            ************************ ***********************************

LA2093      CTGCAGCCAAAATGGCGCATGAATATGTTCTCAATGTTCTACATTAATTTAGTGCTACAG   180
E42         CTGCAGCCAAAATGGCGCATGAATATGTTCTCAATGTTCTACATTAATTTAGTGCTACAG   180
HEINZ       CTGCAGCCAAAATGGCGCATGAATATGTTCTCAATGTTCTACATTAATTTAGTGCTACAG   180
            ************************************************************

LA2093      AGAATGCTGATCAAGTGTAACAACCCATAGCATGTACTATTTGAACCCGAGACAGCCCGG   240
E42         AGAATGCTGATCAAGTGTAACAACCCATAGCATGTACTATTTGAACCCGAGACAGCCCGG   240
HEINZ       AGAATGCTGATCAAGTGTAACAACCCATAGCATGTACTATTTGAACCCGAGACAGCCCGG   240
            ************************************************************

LA2093      AGAGTGAGGACTTCCAAAGCATGGCAAGAAAAAGCATCCTATGGTTTCACTCAGTATATA   300
E42         AGAGTGAGGACTTCCAAAGCATGGCAAGAAAAAGCATCCTATGGTTTCACTCAGTATATA   300
HEINZ       AGAGTGAGGACTTCCAAAGCATGGCAAGAAAAAGCATCCTATGGTTTCACTCAGTATATA   300
            ************************************************************

LA2093      CTCTACAAATTAAGTTTCCGTCTCTTCCTCTTGGCAATTTGCATATATCTAGCCTTCAAT   360
E42         CTCTACAAATTAAGTTTCCGTCTCTTCCTCTTGGCAATTTGCATATATCTAGCCTTCAAT   360
HEINZ       CTCTACAAATTAAGTTTCCGTCTCTTCCTCTTGGCAATTTGCATATATCTAGCCTTCAAT   360
            ************************************************************

LA2093      GTATCGTTGTAGGATAATAATCATAACTCATCATGATCCGAGTTTTCATCTGCAGATGGC   420
E42         GTATCGTTGTAGGATAATAATCATAACTCATCATGATCCGAGTTTTCATCTGCAGATGGC   420
HEINZ       GTATCGTTGTAGGATAATAATCATAACTCATCATGATCCGAGTTTTCATCTGCAGATGGC   420
            ************************************************************

LA2093      TTCTCTGCCTCTGAAGCTGTTTGTTCTTTCTGGGAGGTACCCTCCTCAGAAGAGGATTTG   480
E42         TTCTCTGCCTCTGAAGCTGTTTGTTCTTTCTGGGAGGTACCCTCCTCAGAAGAGGATTTG   480
HEINZ       TTCTCTGCCTCTGAAGCTGTTTGTTCTTTCTGGGAGGTACCCTCCTCAGAAGAGGATTTG   480
            ************************************************************

LA2093      GTAGTGTCTGCTTTCTCCTTGCTGTTTTCAGTTTCATTTTTCAAAGGCTTCTCAACCTTA   540
E42         GTAGTGTCTGCTTTCTCCTTGCTGTTTTCAGTTTCATTTTTCAAAGGCTTCTCAACCTTA   540
HEINZ       GTAGTGTCTGCTTTCTCCTTGCTGTTTTCAGTTTCATTTTTCAAAGGCTTCTCAACCTTA   540
            ************************************************************

LA2093      GGCTTTGGCTTTGGTATTTTATTTACCTTGTTAACCTGATCCATGAAAAAGAGAGAGTGA   600
E42         GGCTTTGGCTTTGGTATTTTATTTACCTTGTTAACCTGATCCATGAAAAAGAGAGAGTGA   600
HEINZ       GGCTTTGGCTTTGGTATTTTATTTACCTTGTTAACCTGATCCATGAAAAAGAGAGAGTGA   600
            ************************************************************

LA2093      CGATCAAGCACTGAAAGATAAAGCTTCATATCTGGAATGAGTTGACTACCAATTTCTTGT   660
E42         CGATCAAGCACTGAAAGATAAAGCTTCATATCTGGAATGAGTTGACTACCAATTTCTTGT   660
HEINZ       CGATCAAGCACTGAAAGATAAAGCTTCATATCTGGAATGAGTTGACTACCAATTTCTTGT   660
            ************************************************************

LA2093      TCAACTCTATCACTAGACCAAATTAACAAAGATTTAGATCACGCACCTTGTCTTGAAGAT   720
E42         TCAACTCTATCACTAGACCAAATTAACAAAGATTTAGATCACGCACCTTGTCTTGAAGAT   720
HEINZ       TCAACTCTATCACTAGACCAAATTAACAAAGATTTAGATCACGCACCTTGTCTTGAAGAT   720
            ************************************************************

LA2093      CAAAAACCTTTACATATACTTCTTCAGAAGTAAATGCAGGCTTGTCGGATCCAGGAGTGC   780
E42         CAAAAACCTTTACATATACTTCTTCAGAAGTAAATGCAGGCTTGTCGGATCCAGGAGTGC   780
HEINZ       CAAAAACCTTTACATATACTTCTTCAGAAGTAAATGCAGGCTTGTCGGATCCAGGAGTGC   780
            ************************************************************

LA2093      TGTAGGAAAAGAAAAGATGTAACAGAGTGTATGAGAAAGGGGATAAAAAGGTGGTAGCAA   840
E42         TGTAGGAAAAGAAAAGATGTAACAGTGTGTATGAGAAAGGGGATAAAAAGGTGGTAGCAA   840
HEINZ       TGTAGGAAAAGAAAAGATGTAACAGTGTGTATGAGAAAGGGGATAAAAAGGTGGTAGCAA   840
            ************************* **********************************
```

```
LA2093      TATTTTCAGTTTCAAAAACAGAATAAAAATTTTCTTAAAACAACATTTATAGAATAGAGT     900
E42         TATTTTCAGTTTCAAAAACAGAATAAAAATTTTCTTAAAACAACATTTATAGAATAGAGT     900
HEINZ       TATTTTCAGTTTCAAAAACAGAATAAAAATTTTCTTAAAACAACATTTATAGAATAGAGT     900
            ************************************************************

LA2093      GGACTTTCTCAAACTTGCAAGCCAGACAGTAGGATATATGGGAAATAATTTTATACCCAT     960
E42         GGACTTTCTCAAACTTGCAAGCCAGACAGTAGGATATATGGGAAATAATTTTATACCCAT     960
HEINZ       GGACTTTCTCAAACTTGCAAGCCAGACAGTAGGATATATGGAAAATAATTTTATACCCAT     960
            ***************************************** ******************

LA2093      GACCCAATAATATTATACGGAAATTCTAGGTAATACTATTATGGATGGGG-GGGTGGAGA     1019
E42         GACCCAATAATATTATACGGAAATTCTAGGTAATACTATTATGGATGGGGGGGGTGGAGA     1020
HEINZ       GACCCAATAATATTATACGGAAATTCTAGGTAATACTATTATGGATGGGGGGGGTGGAGA     1020
            ************************************************** *********

LA2093      ACCAGCCAGCAAAGTATTATTTTGCCACCAGAAATTGTAGGTATAACAGCTCTAGCCACA     1079
E42         ACCAGCCAGCAAAGTATTATTTTGCCACCAGAAATTGTAGGTATAACAGCTCTAGCCACA     1080
HEINZ       ACCAGCCAGCAAAGTATTATTTTGCCACCAGAAATTGTAGGTATAACAGCTCTAGCCACA     1080
            ************************************************************

LA2093      TCGCACCCTACAGCTAACGCACACGGCATGTGATGGGTCCTTGTCACTGAACAAAATTCC     1139
E42         TCGCACCCTACAGCTAACGCACACGGCATGTGATGGGTCCTTGTCACTGAACAAAATTCC     1140
HEINZ       TCGCACCCTACAGCTAACGCACACGGCATGTGATGGGTCCTTGTCACTGAACAAAATTCC     1140
            ************************************************************

LA2093      CAGGGGGCAACATCTTAGAACACCCATTTTCTTCATTTTTCTAGTCTCTCCATATATCTT     1199
E42         CAGGGGGCAACATCTTAGAACACTCATTTTCTTCATTTTTCTAGTCTCTCCATATATCTT     1200
HEINZ       CAGGGGGCAACATCTTAGAACACTCATTTTCTTCATTTTTCTAGTCTCTCCATATATCTT     1200
            *********************** ************************************

LA2093      TCATTCCAAGGTCCCAAATGAATAGGGCAAATGTTAAATGAAAAAACTGCAAAGTTCCAC     1259
E42         TCATTCTAAGGTCCCAAATGAATAGGGCAAATGTTAAATGAAAAAACTACAAAGTTCCAC     1260
HEINZ       TCATTCTAAGGTCCCAAATGAATAGGGCAAATGTTAAATGAAAAAACTACAAAGTTCCAC     1260
            ****** ***************************************** ***********

LA2093      CTTTCTCCCTCCTAATTCAGTTGAACGAAATACAGAAACAACTAAGAATACACAAACACG     1319
E42         CTTTCTCCCTCCTAATTCAGTTGAACGAAATACAGAAACAACTAAGAATACACAAACACG     1320
HEINZ       CTTTCTCCCTCCTAATTCAGTTGAACGAAATACAGAAACAACTAAGAATACACAAACACG     1320
            ************************************************************

LA2093      TGTATATACAATTTGCAAATGTTCAAATCAAAGTGTATATGCTTCAACACACACACACGT     1379
E42         TGTATATACAATTTGCAAATGTTCAAATCAAAGTGTATATGCTTCAACACACACACACGT     1380
HEINZ       TGTATATACAATTTGCAAATGTTCAAATCAAAGTGTATATGCTTCAACACACACACACGT     1380
            ************************************************************

LA2093      ATCCACTAGATTTATTCGTTACAGATGATATATCAGTCAGGATCACTTTCGGGAGTTCAA     1439
E42         ATCCACTAGATTTATTCTTTACAGATGATATATCAGTCAGGATCACTTTCGGGAGTTCAA     1440
HEINZ       ATCCACTAGATTTATTCTTTACAGATGATATATCAGTCAGGATCACTTTCGGGAGTTCAA     1440
            **************** *******************************************

LA2093      TGGCTCAATATTTTATTGAACAAGATGTTCCAACAGGAAACAACTTGCTATGTTCACTGC     1499
E42         TGGCTCAATATTTTATTGAACAAGATGTTCCAACAGGAAACAACTCGCTATGTTCACTGC     1500
HEINZ       TGGCTCAATATTTTATTGAACAAGATGTTCCAACAGGAAACAACTCGCTATGTTCACTGC     1500
            ******************************************** ***************

LA2093      TTAAAGCGGCAGTGGAAACAAACAGAGTAAGTACGAAAAGAAAAAGGGGAGAGGCAATAA     1559
E42         TTAAAGCGGCAGTGGAAACAAACAGAGTAAGTACGAAAAGAAAAAGGGGAGAGGCGATAA     1560
HEINZ       TTAAAGCGGCAGTGGAAACAAACAGAGTAAGTACGAAAAGAAAAAGGGGAGAGGCAATAA     1560
            ******************************************************* ****

LA2093      CATGGAAAGTGAACGCACAAATGACCATATGAAAATAGGAAACAGGTAAGAGAAATGAGA     1619
E42         CATGGAAAGTGAACGCACAAATGACCATATGAAAATAGGAAACAGGTAAGAGAAATGAGA     1620
HEINZ       CATGGAAAGTGAACGCACAAATGACCATATGAAAATAGGAAACAGGTAAGAGAAATGAGA     1620
            ************************************************************

LA2093      AAGAAAAAGAACTTACTTTTTCTGTTCAGCCTCCTTCTGATTTAACCAATTCTTCACTTT     1679
E42         AAGAAAAAGAACTTACTTTTTCTGTTCAGCCTCCTTCTGATTTAACCAATTCTTCACTTT     1680
HEINZ       AAGAAAAAGAACTTACTTTTTCTGTTCAGCCTCCTTCTGATTTAACCAATTCTTCACTTT     1680
            ************************************************************

LA2093      CTCAGATTCATTTAGAACCTGCAAACATGACCCTTTTAAAACTTCCCCAGTACGAACATA     1739
```

```
E42       CTCAGATTCATTTAGAACCTGCAAACATGACCCTTTTAAAACTTCCCCGGTACGAACATA   1740
HEINZ     CTCAGATTCATTTAGAACCTGCAAACATGACCCTTTTAAAACTTCCCCGGTACGAACATA   1740
          ************************************************ ***********

LA2093    AAGAAGAGATGCCTTAGCTTCTATTTATAAAATCAGAAATATGCACAACAACTGAAAAGA   1799
E42       AAGAAGAGATGCCTTAGCTTCTATTTATAAAATCAGAAATATGCACAACAACTGAAAAGA   1800
HEINZ     AAGAAGAGATGCCTTAGCTTCTATTTATAAAATCAGAAATATGCACAACAACTGAAAAGA   1800
          ************************************************************

LA2093    GTACCTCATCTATTTTTCCCTTAGGAAGCCACGATTTGTTGGTTTCCCATCCACGCACAA   1859
E42       GTACCTCATCTATTTTTCCCTTAGGAAGCCACGATTTGTTGGTTTCCCATCCACGCACAA   1860
HEINZ     GTACCTCATCTATTTTTCCCTTAGGAAGCCACGATTTGTTGGTTTCCCATCCACGCACAA   1860
          ************************************************************

LA2093    TCTGAAGGTATCCAAGAAAGAAATAAAAGGTTAACTGCAAGGGTCAAAGGCAATAAAAGA   1919
E42       TCTGAAGGTATCCAAGAAAGAAATAAAAGGTTAACTGCAAGGGTCAAAGGCAATAAAAGA   1920
HEINZ     TCTGAAGGTATCCAAGAAAGAAATAAAAGGTTAACTGCAAGGGTCAAAGGCAATAAAAGA   1920
          ************************************************************

LA2093    TACAAAACTTCCCTAGCTGGCTAACAAGCTTTGAAAATCACAGAAGGATCATTGCAAAGG   1979
E42       TACAAAACTTCCCTAGCTGGCTAACAAGCTTTGAAAATCACAGAAGGATTATTGCAAAGG   1980
HEINZ     TACAAAACTTCCCTAGCTGGCTAACAAGCTTTGAAAATCACAGAAGGATTATTGCAAAGG   1980
          ************************************************* **********

LA2093    AAGCCAGTTAATAAGTTCAACATCAACAGTGTAGTGGAATCTTCTACTAATGCCACAATG   2039
E42       AAGCCAGTTAATAAGTTCAACATCAACAGTGTAGTTGAATCTTCTACTAATGCCACAATG   2040
HEINZ     AAGCCAGTTAATAAGTTCAACATCAACAGTGTAGTTGAATCTTCTACTAATGCCACAATG   2040
          *********************************** ************************

LA2093    ACTGTTATGGTACCAAATGTGAAATCTCTAAAATACAGATGCAGGTTAATATCAATA---   2096
E42       ACTGTTATGGTACCAAATGTGAAATCTCTAAAATACAGATGCAGGTTAATATCAATTTCA   2100
HEINZ     ACTGTTATGGTACCAAATGTGAAATCTCTAAAATACAGATGCAGGTTAATATCAATTTCA   2100
          ********************************************************

LA2093    TCAATATGCCTTCTGCACTTCAAATCTAGAAAATACCCATGGGAATTATCACCTCAAACA   2156
E42       TCAATATGCCTTCTGCACTTCAAATCTAGAAAATACCCATGGGAATTATCACCTCAAACA   2160
HEINZ     TCAATATGCCTTCTGCACTTCAAATCTAGAAAATACCCATGGGAATTATCACCTCAAACA   2160
          ************************************************************

LA2093    GTGCCTCAAAACATACAGCTTATTTTCCCACATCTTCGCTTGAAGAACTTCACTGCCGAC   2216
E42       GTGCCTCAAAACATACAGCTTATTTTCCCACATCTTCGCTTGAAGAACTTCACTGCCGAC   2220
HEINZ     GTGCCTCAAAACATACAGCTTATTTTCCCACATCTTCGCTTGAAGAACTTCACTGCCGAC   2220
          ************************************************************

LA2093    TTACAATTCGCTGTGCGGGTGCAAACTAGTAGTTTTTTCTTAAACATACTAATTTCATCA   2276
E42       TTACAATTCGCTGTGCTGGTGCAAACTAGTAGTTTTTTCTTAAACATACTAATTTCACCA   2280
HEINZ     TTACAATTCGCTGTGCTGGTGCAAACTAGTAGTTTTTTCTTAAACATACTAATTTCACCA   2280
          **************** **************************************** **

LA2093    TTTCCTTGAGAAAACAAACAAAGAGATACTCCCTCTGTTTCAATTTGCTTGTCTTAGTTT   2336
E42       TTTCCTTGAGAAAACAAACAAAGAGATACTCCCTCTGTTTCAATTTGCTTGTCTTAGTTT   2340
HEINZ     TTTCCTTGAGAAAACAAACAAAGAGATACTCCCTCTGTTTCAATTTGCTTGTCTTAGTTT   2340
          ************************************************************

LA2093    GACTCGGCACGGAGTTTGAGAAAATAAAGAAAAAATTTAGAATTTGGTGGTCTTAAACTAA   2396
E42       GACTCGGCACGGAGTTTGAGAAAATAAAGAAAAAATTTAGAATTTGGTGGTCTTAAACTAA   2400
HEINZ     GACTCGGCACGGAGTTTGAGAAAATAAAGAAAAAATTTAGAATTTGGTGGTCTTAAACTAA   2400
          ************************************************************

LA2093    ATATGTGTAATGTACCAAAACGCCCTATGAATTTGTGGTCTTAAATATGCCATGTATGAT   2456
E42       ATATGTGTAATGTACCAAAACGCCCTATGAATTTGTGATCTTAAATATGCCATGTATGAT   2460
HEINZ     ATATGTGTAATGTACCAAAACGCCCTATGAATTTGTGATCTTAAATATGCCATGTATGAT   2460
          ************************************* **********************

LA2093    ATAGGGTGTAAAGAGTAACTGAATAAAGAAAAACATTCTTTTTGAAACAGATCTAATGCA   2516
E42       ATTGGGTGTAAAGAGTAACTGAATAAAGAAAAACATTCTTTTTGAAACAGATCTAACGCA   2520
HEINZ     ATTGGGTGTAAAGAGTAACTGAATAAAGAAAAACATTCTTTTTGAAACAGATCTAACGCA   2520
          ** ***************************************************** ***

LA2093    AGACAAACGAATTGACACGGAGGGAGTCCTATTTATGTCCAGCTATCTATAGATACCACC   2576
E42       AGACAAACGAATTGACACGGAGGGAGTCCTATTTATGTCCAGCTATCTATAGATACCACC   2580
HEINZ     AGACAAACGAATTGACACGGAGGGAGTCCTATTTATGTCCAGCTATCTATAGATACCACC   2580
```

```
            ************************************************************

LA2093      ATAACACAAATAAGACACTCTTTCAACTAACAAAATGCACTTCATGATGCAAAGAGTTTG    2636
E42         ATAACACAAATAAGACACTCTTTCAACTAACAAAATGCACTTCATGATGCAAAGAGTTTG    2640
HEINZ       ATAACACAAATAAGACACTCTTTCAACTAACAAAATGCACTTCATGATGCAAAGAGTTTG    2640
            ************************************************************

LA2093      ACCAAATAGAGCAGAAGAGTGTTACGGGAACAAGAAATTAAACTTCCTACCTGTTGCACT    2696
E42         ACCAAATAGAGCAGAAGAGTGTTACGGGAACAAGAAATTAAACTTCCTACCTGTTGCACT    2700
HEINZ       ACCAAATAGAGCAGAAGAGTGTTACGGGAACAAGAAATTAAACTTCCTACCTGTTGCACT    2700
            ************************************************************

LA2093      TCATTAAGGTATTTGCGAGCATGATCAGATGAAGCAGGGCGTGCAGTAAGTTCTTTATGT    2756
E42         TCATTAAGGTATTTGCGAGCATGATCAGATGAAGCAGGGCGTGCAGTAAGTTCTTTATGT    2760
HEINZ       TCATTAAGGTATTTGCGAGCATGATCAGATGAAGCAGGGCGTGCAGCAAGTTCTTTATGT    2760
            ********************************************** *************

LA2093      CTGCATTAAAAATTACATTCAGCATAACTACCGAACCATGTACAAAAGGTAACTACAATT    2816
E42         CTGCATTAAAAATTACATTCAGCATAACTACCGAACCATGTACAAAAGGTAACTACAATT    2820
HEINZ       CTGCATTAAAAATTACATTCAGCATAACTACCGAACCATGTACAAAAGGTAACTACAATT    2820
            ************************************************************

LA2093      TAGCCTTTGTCTGCAGTTGATACTAGGAGCAGACCTGAAAAATATGGGATCCCCAATAGC    2876
E42         TAGCCTTTGTCTGCAGTTGATACTAGGAGCAGACCTGAAAAATATGGGATCCCCAATAGC    2880
HEINZ       TAGCCTTTGTCTGCAGTTGATACTAGGAGCAGACCTGAAAAATATGGGATCCCCAATAGC    2880
            ************************************************************

LA2093      TTTCAACTTATCTAAATGTTCTTGAAACTGCTTGGCAGAAGCATCTTCACCATCTGTGTA    2936
E42         TTTCAACTTATCTAAATGTTCTTGAAACTGCTTGGCAGAAGCATCTTCACCATCTGTGTA    2940
HEINZ       TTTCAACTTATCTAAATGTTCTTGAAACTGCTTGGCAGAAGCATCTTCACCATCTGTGTA    2940
            ************************************************************

LA2093      CAACCATTCTTGTACCTGAAAAACTCCATTTAGTATATTTAAGATAAACACATGACAAAA    2996
E42         CAACCATTCTTGTACCTGAAAAACTCCATTTAGTATATTTAAGATAAACACATGACAAAA    3000
HEINZ       CAACCATTCTTGTACCTGAAAAACTCCATTTAGTATATTTAAGATAAACACATGACAAAA    3000
            ************************************************************

LA2093      CATGGAAAAAAGGAAATATGCAAACCTCATCAAGTTTCTGAATAAAGGACTGGCGCTCTT    3056
E42         CATGGAAAAAAGGAAATATGCAAACCTCATCAAGTTTCTGAATAAAGGACTGGCGCTCTT    3060
HEINZ       CATGGAAAAAAGGAAATATGCAAACCTCATCAAGTTTCTGAATAAAGGACTGGCGCTCTT    3060
            ************************************************************

LA2093      GACTGGTTGATATTGTCACAAAATCTCCAGATTCAAGCTGGTACATGAGCACATTCACCA    3116
E42         GACTGGTTGATATTGTCACAAAATCTCCAGATTCAAGCTGGTACATGAGCATATTCACCA    3120
HEINZ       GACTGGTTGATATTGTCACAAAATCTCCAGATTCAAGCTGGTACATGAGCACATTCACCA    3120
            *************************************************** ********

LA2093      TTAAAAAAAAGTGATCAATTAATCAGTTCCATGTGATCAATCAGCGAATAAAAATGTTGG    3176
E42         TTAAAAAAAAGTGATCAATTAATCAGTTCCATGTGATCAATCAGCGAATAAAAATGTTGG    3180
HEINZ       TTAAAAAAAAGTGATCAATTAATCAGTTCCATGTGATCAATCAGCGAATAAAAATGTTGG    3180
            ************************************************************

LA2093      TGCAAAACAGTGATGCCACTGGAAGCTAAATGATATGAAGTGCATTACAAGAAGAAAAAG    3236
E42         TGCAAAACAGTGATGCCACTGGAAGCTAAATGATATGAAGTGCATTACAAGAAGAAAAAG    3240
HEINZ       TGCAAAACAGTGATGCCACTGGAAGCTAAATGATATGAAGTGCATTACAAGAAGAAAAAG    3240
            ************************************************************

LA2093      AGACCAAGAAGCTAACAGTGATCACAAGGTTATAACATCTAGGAAACAATATCACCTGAG    3296
E42         AGACCAAGAAGCTAACAGTGATCACAAGGTTGTAACATCTAGGAAACAATATCACCTGAG    3300
HEINZ       AGACCAAGAAGCTAACAGTGATCACAAGGTTGTAACATCTAGGAAACAATATCACCTGAG    3300
            ******************************* ****************************

LA2093      CAACAGAGTAAGTTCTGCATCTGTGTAAGCATCAGTTTAACAAAATGATGTTTCAGAAGA    3356
E42         CAACAAAGTAAGTTCTGCATCTGTGTAAGCATCAGTTTAACAAAATGATGTTTCAGGAGA    3360
HEINZ       CAACAAAGTAAGTTCTGCATCTGTGTAAGCATCAGTTTAACAAAATGATGTTTCAGGAGA    3360
            ***** ************************************************** ***

LA2093      ATCTTGAAGCATGGGTATGTGCTTAAAAACTATTGCAAGAAAGATTTGTCTAGCTGCAAA    3416
E42         ATCTTGAAGCATGGGTATGTGCTTAAAAGCTATTGCAAGAAAGATTTGTCTAGCTGCAAA    3420
HEINZ       ATCTTGAAGCATGGGTATGTGCTTAAAAGCTATTGCAAGAAAGATTTGTCTAGCTGCAAA    3420
            **************************** *******************************
```

```
LA2093      GCCGTTACTCAAAGCTAGAGGAAAAACACATATTAACAAATCGAACAAGTGTATGCAACT     3476
E42         GCCGTTACTCAAAGCTAGAGGAAAAACACATATTAACAAATCGAACAAGTGTATGCAACT     3480
HEINZ       GCCGTTACTCAAAGCTAGAGGAAAAACACATATTAACAAATCGAACAAGTGTATGCAACT     3480
            ************************************************************

LA2093      GGAACGAAAAATGAGAAAATCAAGAGTACACACAAGGCATTCAAGTTTACAGAGCTGAAC     3536
E42         GGAACGAAAAATGAGAAAATCAAGAGTACACACAAGGCATTCAAGTTTACAGAGCTGAAC     3540
HEINZ       GGAACGAAAAATGAGAAAATCAAGAGTACACACAAGGCATTCAAGTTTACAGAGCTGAAC     3540
            ************************************************************

LA2093      AGATCAGGTAACTACATAATCACAGGCATGAGCAAGAGTTAGAGATATAAGCCCTCGAAC     3596
E42         AGATCAGGTAACTACATAATCACAGGCATGAGCAAGAGTTAGAGATATAAGCCCTCGAAC     3600
HEINZ       AGATCAGGTAACTACATAATCACAGGCATGAGCAAGAGTTAGAGATATAAGCCCTCGAAC     3600
            ************************************************************

LA2093      CTTGTCTCTCGTATCATATATGTATCCTTCCAAGCTATTCTTCAATTCAGCTGTTCTTCT     3656
E42         CTTGTCTCTCGTATCATATATGTATCCTTCCAAGCTATTCTTCAATTCAGCTGTTCTTCT     3660
HEINZ       CTTGTCTCTCGTATCATATATGTATCCTTCCAAGCTATTCTTCAATTCAGCTGTTCTTCT     3660
            ************************************************************

LA2093      TCTTTCTTCATCTTTTTTGTCCAGTGCTTCCAATTTGCTTTTAGCTTCACTAAAAGACTC     3716
E42         TCTTTCTTCATCTTTTTTGTCCAGTGCTTCCAATTTGCTTTTAGCTTCACTAAAAGACTC     3720
HEINZ       TCTTTCTTCATCTTTTTTGTCCAGTGCTTCCAATTTGCTTTTAGCTTCACTAAAAGACTC     3720
            ************************************************************

LA2093      TTTTGAAAGAGGTGCTCCTGGCCCAGCAGTCTTCTCATCAATCTACATGAACAAAGAAGT     3776
E42         TTTTGAAAGAGGTGCTCCTGGCCCAGCAGTCTTCTCATCAATCTACATGAACAAAGAAGT     3780
HEINZ       TTTTGAAAGAGGTGCTCCTGGCCCAGCAGTCTTCTCATCAATCTACATGAACAAAGAAGT     3780
            ************************************************************

LA2093      CTCAAGGATAAGACTGGCTAAAAACATCATAACACTACGATATTTCTAAAATAACATTTT     3836
E42         CTCAAGGATAAGACTGGCTAAAAACATCATAACACTACGATATTTCTAAAATAACATTTT     3840
HEINZ       CTCAAGGATAAGACTGGCTAAAAACATCATAACACTACGATATTTCTAAAATAACATTTT     3840
            ************************************************************

LA2093      TTCCTGGCACGTAATGGAAATCGATAAAGAAGGGGATGCAAAATAGATTCCCCTGCAAAT     3896
E42         TTCCTGGCACGTAATGGAAATCGATAAAGAAGGGGATGCAAAATAGATTCCCCTGCAAAT     3900
HEINZ       TTCCTGGCACGTAATGGAAATCGATAAAGAAGGGGATGCAAAATAGATTCCCCTGCAAAT     3900
            ************************************************************

LA2093      GGATAGGTTTAGAAGACCTTAAGTGGAACTCGGAAAGTCCGTTTCTTCAGCTTCTTCTCT     3956
E42         GGATAGGTTTAGAAGACCTTAAGTGGAACTCGGAAAGTCCGTTTCTTCAGCTTCTTCTCT     3960
HEINZ       GGATAGGTTTAGAAGACCTTAAGTGGAACTCGGAAAGTCCGTTTCTTCAGCTTCTTCTCT     3960
            ************************************************************

LA2093      GTAACGGGACTTATGGTGCTAGAATCATTTGCACCAGAATCAGAGGTGTTACTATTAACA     4016
E42         GTAACGGGACTTATGGTGCTAGAATCATTTGCACCAGAATCAGAGGTGTTACTATTAACA     4020
HEINZ       GTAACGGGACTTATGGTGCTAGAATCATTTGCACCAGAATCAGAGGTGTTACTATTAACA     4020
            ************************************************************

LA2093      ATGTCTGGGTTCAACTTTTCATCACTTTCCTCTGTACTGCTGGGGCCACTTTCAGTTGAT     4076
E42         ATGTCTGGGTTCAACTTTTCATCACTTTCCTCTGTATTGCTGGGGCCACTTTCAGTTGAT     4080
HEINZ       ATGTCTGGGTTCAACTTTTCATCACTTTCCTCTGTATTGCTGGGGCCACTTTCAGTTGAT     4080
            ************************************ ***********************

LA2093      GTGTTTGCAGAAGCAGAGGTCGAGTTGTCCACTGTCAGATTCTTTACTGGAACTTCGACC     4136
E42         GTGTTTGCAGAAGCAGAGGTCGAGTTGTCCACTGTCAGATTCTTTACTGGAACTTCGACC     4140
HEINZ       GTGTTTGCAGAAGCAGAGGTCGAGTTGTCCACTGTCAGATTCTTTACTGGAACTTCGACC     4140
            ************************************************************

LA2093      CATTCAGTTATTTCAATAACAGCATCTGCTCGATCTAATGAAAATATTCCACTTCTACTA     4196
E42         CATTCAGTTATTTCAATAACAGCATCTGCTCGATCTAATGAAAATATTCCACTTCTACTA     4200
HEINZ       CATTCAGTTATTTCAATAACAGCATCTGCTCGATCTAATGAAAATATTCCACTTCTACTA     4200
            ************************************************************

LA2093      AGTGAGAAATGTAAATTAGCCTTGACTGGGGCAGAAAGATTCCGTGATGCATACCTAAAA     4256
E42         AGTGAGAAATGTAAATTAGCCTTGACTGGGGCAGAAAGATTCCGTGATGCATACCTAAAA     4260
HEINZ       AGTGAGAAATGTAAATTAGCCTTGACTGGGGCAGAAAGATTCCGTGATGCATACCTAAAA     4260
            ************************************************************

LA2093      AAAGAAAAGCACAAAGCTCAGAATAATAGATCACCAAACACCATCTGATCCAGATATCAG     4316
E42         AAAGAAAAGCACAAAGCTCAGAATAATAGATCACCAAACACCATCTGATCCAGATATCAG     4320
```

```
HEINZ        AAAGAAAAGCACAAAGCTCAGAATAATAGATCACCAAACACCATCTGATCCAGATATCAG    4320
             ************************************************************

LA2093       TGATTCTTACTTTTCGCTAGCATCAGTAAGACCTGACACTGCGTACTGAGCAAATGTACG    4376
E42          TGATTCTTACTTTTCGCTAGCATCAGTAAGACCTGACACTGCGTACTGAGCAAATGTGCG    4380
HEINZ        TGATTCTTACTTTTCGCTAGCATCAGTAAGACCTGACACTGCGTACTGAGCAAATGTACG    4380
             ********************************************************* **

LA2093       TGAAGTAGTTCCAGGTGGCAAGAAGTCATCGCTTTCATACGCTAGTGAAACTTCAAAATC    4436
E42          TGAAGTAGTTCCAGGTGGCAAGAAGTCATCGCTTTCATACGCTAGTGAAACTTCAAAATC    4440
HEINZ        TGAAGTAGTTCCAGGTGGCAAGAAGTCATCGCTTTCATACGCTAGTGAAACTTCAAAATC    4440
             ************************************************************

LA2093       CTTCTTGTGAACAATAGATCTGAACATCTGCAGATCAATCACACAATCGGACATAGAACT    4496
E42          CTTCTTGTGAACAATAGATCTGAACATCTGCAGATCAATCACACAATCGGACATAGAACT    4500
HEINZ        CTTCTTGTGAACAATAGATCTGAACATCTGCAGATCAATCACACAATCGGACATAGAACT    4500
             ************************************************************

LA2093       GTCATGTGTTAAGCATGTTCTTTGATAGCCTATTGTTCACCAACAATATACAGACAAAAC    4556
E42          GTCATGTGTTATGTATGTTCTTTGATAGCCTATTGTTCACCAACAATATACAGACAAAAC    4560
HEINZ        GTCATGTGTTATGTATGTTCTTTGATAGCCTATTGTTCACCAACAATATACAGACAAAAC    4560
             *********** * **********************************************

LA2093       AAAATTCTGATATACCTTGCTAGGCAGTTTCTTCATACGTGGTATGGTCAACTGTTTCGT    4616
E42          AAAATTCTGATATACCTTGCTAGGCAGTTTCTTCATACGTGGTATGGTCAACTGTTTCGT    4620
HEINZ        AAAATTCTGATATACCTTGCTAGGCAGTTTCTTCATACGTGGTATGGTCAACTGTTTCGT    4620
             ************************************************************

LA2093       GCTTTCATCTTTAGGAAGGTCCGGACCATCTACTTCAATCACATATCCATATGGAGAACC    4676
E42          GCTTTCATCTTTAGGAAGGTCCGGACCATCTACTTCAATCACATATCCATATGGAGAACC    4680
HEINZ        GCTTTCATCTTTAGGAAGGTCCGGACCATCTACTTCAATCACATATCCATATGGAGAACC    4680
             ************************************************************

LA2093       ATCAATCATTCCCAGTTTACGATTCAATTTGATTCCATCACTTATATTAGCAGCATGCAA    4736
E42          ATCAATCATTCCCAGTTTACGATTCAATTTGATTCCATCACTTATATTAGCAGCATGCAA    4740
HEINZ        ATCAATCATTCCCAGTTTACGATTCAATTTGATTCCATCACTTATATTAGCAGCATGCAA    4740
             ************************************************************

LA2093       TGAGGCCCCAAGTGCAATTGCTTCATCGGAGTCCAAGTGTCTGTCCAGTTCTTTCCTTCC    4796
E42          TGAGGCCCCAAGTGCAATTGCTTCATCGGAGTCCAAGTGTCTGTCCAGTTCTTTCCTTCC    4800
HEINZ        TGAGGCCCCAAGTGCAATTGCTTCATCGGAGTCCAAGTGTCTGTCCAGTTCTTTCCTTCC    4800
             ************************************************************

LA2093       AAGAAATTCCTGTAGCTTAGCCTGCAAGCAAGGAATGTAAATTTTATTTTCAAGAAAACA    4856
E42          AAGAAATTCCTGTAGCTTAGCCTGCAAGCAAGGAATGTAAATTTTATTTTCAAGAAAACA    4860
HEINZ        AAGAAATTCCTGTAGCTTAGCCTGCAAGCAAGGAATGTAAATTTTATTTTCAAGAAAACA    4860
             ************************************************************

LA2093       ATCAGTGTTCTGACAACTCCTAAATCGATCATGTTACAAGCAATGATGTCGAAATCGACA    4916
E42          ATCAGTGTTCTGACAATTCCTAAATCGATCATGTTACAAGCAATGATGTCGAAATCGACA    4920
HEINZ        ATCAGTGTTCTGACAATTCCTAAATCGATCATGTTACAAGCAATGATGTCGAAATCGACA    4920
             **************** *******************************************

LA2093       TTGACTCGACTGCAAGAAACTCCACCAACCAACAAGAAAATATTTATGTTATCCTAATTA    4976
E42          TTGACTCGACTGCAAGAAACTCCACCAACCAACAAGAAAATATTTATTTTATCCTAATTA    4980
HEINZ        TTGACTCGACTGCAAGAAACTCCACCAACCAACAAGAAAATATTTATTTTATCCTAATTA    4980
             *********************************************** ************

LA2093       TATGTAATTAGTGCCGGAAAAATGAGCTCATATTTTGATGACCCGCCTAACCCGCCCATA    5036
E42          TATGTAATTAGTGCCGGAAAAATGAGCTCATATTTTGATGACCCGCCTAACCCGCCCATA    5040
HEINZ        TATGTAATTAGTGCCGGAAAAATGAGCTCATATTTTGATGACCCGCCTAACCCGCCCATA    5040
             ************************************************************

LA2093       TTTTAACGGGTTGGGTGAGTGATTTTTCACATGGGTAAAATATGGGTAATATATGAATTA    5096
E42          TTTTAACGGGTTGGGTGAGTGATTTTTCACATGGGTAAAATATGGGTAATATATGAATTA    5100
HEINZ        TTTTAACGGGTTGGGTGAGTGATTTTTCACATGGGTAAAATATGGGTAATATATGAATTA    5100
             ************************************************************

LA2093       GTCAAATGGGTTAAGCTTCTAACTTTTATTCACTCAAAATTCATAATATCACTCTAAATA    5156
E42          GTCAAATGGGTTAAGCTTCTAACTTTTATTCACTCAAAATTCATAATATCACTCTAAATA    5160
HEINZ        GTCAAATGGGTTAAGCTTCTAACTTTTATTCACTCAAAATTCATAATATCACTCTAAATA    5160
             ************************************************************
```

```
LA2093      GTGTAACTTCTCTTCCTTTTTATGTTATTCTATAGAACTTAT------------------   5198
E42         GTGTAACTTCTCTTCCTTTTTATGTTATTCTATAGAACTAACTATTCTTCTTTATAATTG   5220
HEINZ       GTGTAACTTCTCTTCCTTTTTATGTTATTCTATAGAACTAACTATTCTTCTTTATAATTG   5220
            *************************************** *

LA2093      ------------------------------------------------------------   5198
E42         AAAAGGTGAAGTCTTTGACACTCCAAAAATATATAATTCAAATGTATATTTCTTTTGTTA   5280
HEINZ       AAAAGGTGAAGTCTTTGACACTCCAAAAATATATAATTCAAATGTATGTTTCTTTTGTTA   5280


LA2093      ------------------------------------------------------------   5198
E42         ATTATAATACATTTATTTATTTAATTTTATATTGAATAATCAATAATAGTATAAAATAAG   5340
HEINZ       ATTATAATACATTTATTTATTTAATTTTATATTGAATAATCAATAATAGTATAAAATAAG   5340


LA2093      ---------------------------------------------GTTTTAGTCCTAAAA   5213
E42         CAAATCATGTATTAAAATTGACATTGCTTTAAGATCGTTAAGCATGTTTTAGTCCTAAAA   5400
HEINZ       CAAATCATGTATTAAAATTGACATTGCTTTAAGATCGTTAAGCATGTTTTAGTCCTAAAA   5400
                                                         ***************

LA2093      TACAAATATTCATTTACTTTGAAATTAAAAATATTTTCATCTTGTTATTACATAAATCTA   5273
E42         TACAAATATTCATTTACTTTGAAATTAAAAATATTTTCATCTTGTTATTACATAAATCTA   5460
HEINZ       TACAAATATTCATTTACTTTGAAATTAAAAATATTTTCATCTTGTCATTACATAAATCTA   5460
            ********************************************* **************

LA2093      TTTTATATTGAATTCTTTTAATGATACAATAAAACTAAATGAAGCACGTTCAACATTTTT   5333
E42         TTTTATATTGAATTCTTTTAATGATACAATAAAACTAAATGAAGCACGTTCAACATTTTT   5520
HEINZ       TTTTATATTGAATTCTTTTAATGATACAATAAAACTAAATGAAGCACGTTCAACATTTTT   5520
            ************************************************************

LA2093      CATCTAAAAAAGACTAAAAAATCTTCTCCAATCAATTTTTAACCATATCAATTATGGGTG   5393
E42         CATCTAAAAAAGACTAAAAAATCTTCTCCAATCAATTTTTAACCATATCAATTATGGGTG   5580
HEINZ       CATCTAAAAAAGACTAAAAAATCTTCTCCAATCAATTTTTAACCATATCAATTATGGGTG   5580
            ************************************************************

LA2093      GGTTGAATATTACCCATTTTATGTTTGCCCATTTTCAACCCGCCTACATTTGACCCAACC   5453
E42         GGTTGAATATTACCCATTTTATGTTTGCCCATTTTCAACCCGCCTACATTTGACCCAACC   5640
HEINZ       GGTTGAATATTACCCATTTTATGTTTGCCCATTTTCAACCCGCCTACATTTGACCCAACC   5640
            ************************************************************

LA2093      CGCCCATTTTCAACCCGCATATATGTAAATCATAAGCAACCAAAAAATTACCTTGTGTTT   5513
E42         AGCCCATTTTCAACCCGCATATATGTAAATCATAAGCAACCAAAAAATTACCTTGTGTTT   5700
HEINZ       CGCCCATTTTCAACCCGCATATATGTAAATCATAAGCAACCAAAAAATTACCTTGTGTTT   5700
             ***********************************************************

LA2093      ACAAGTATATGACAACTGCAAATATGTATATTTTTACTGTGGTTAGACCTTAACTTTGTT   5573
E42         ACAAGTATATGACAACTGCAAATATGTATATTTTTACTGTGGTTAGACCTTAACTTTGTT   5760
HEINZ       ACAAGTATATGACAACTGCAAATATGTATATTTTTACTGTGGTTAGACCTTAACTTTGTT   5760
            ************************************************************

LA2093      AAGAAAAGAGCAGCGACTCAGACCTGCAATTTTGGCACCCTAGTGGCACCTCCAATCAAC   5633
E42         AAGAAAAGAGCAGCGACTCAGACCTGCAATTTTGGCACCCTAGTGGCACCTCCAATCAAC   5820
HEINZ       AAGAAAAGAGCAGCGACTCAGACCTGCAATTTTGGCACCCTAGTGGCACCTCCAATCAAC   5820
            ************************************************************

LA2093      TCCACTGCATAAATATCTTCAATTTTCAAACCAGAATGAGTAAGAACTTCCTTTAATGGT   5693
E42         TCCACTGCATAAATATCTTCAATTTTCAAACCAGAATGAGTAAGAACTTCCTTTAATGGT   5880
HEINZ       TCCACTGCATAAATATCTTCAATTTTCAAACCAGAATGAGTAAGAACTTCCTTTAATGGT   5880
            ************************************************************

LA2093      ACAAGAGCTTTTTCCCACAAATCGGCACATAACTCTTCAAACTTTTCACGGGTTATTGAG   5753
E42         ACAAGAGCTTTTTCCCACAAATCGGCACATAACTCTTCAAACTTTTCACGGGTTATTGAG   5940
HEINZ       ACAAGAGCTTTTTCCCACAAATCGGCACATAACTCTTCAAACTTTTCACGGGTTATTGAG   5940
            ************************************************************

LA2093      CTCCTGCGACAAAATAAATTCCTGATGAGCGAATAAATGTAAAACAAGTGAAAAATACTA   5813
E42         CTCCTGCGACAAAATAAATTCCTGATGAGCGAATAAATGTAAAACAAGTGAAAAATACTA   6000
HEINZ       CTCCTGCGACAAAATAAATTCCTGATGAGCGAATAAATGTAAAACAAGTGAAAAATACTA   6000
            ************************************************************

LA2093      CTGAATATCACTCTTCCGCCTCTTCCAGATGTGTAAGCATGCCAGGGCATACACATAGTC   5873
```

```
E42         CTGAATATCACTCTTCCGCCTCTTCCAGATGTGTAAGCATGCCAGGGCATACACATAGTC    6060
HEINZ       CTGAATATCACTCTTCCGCCTCTTCCAGATGTGTAAGCATGCCAGGGCATACACATAGTC    6060
            ************************************************************

LA2093      ACACATAAGAGGAAGCGTACTCCTATATGTATTAGATAGAAACACTATTCATGGGGGTAG    5933
E42         ACACATAAGAGGAAGCGTACTCCTATATGTATTAGATAGAAACACTATTCATGGGAGTAG    6120
HEINZ       ACACATAAGAGGAAGCGTACTCCTATATGTATTAGATAGAAACACTATTCATGGGGGTAG    6120
            ******************************************************* ****

LA2093      AACTTTCTTATCAAAGACGGAATTGCATTTGATCATGTCAACTAGAAATAAACATAATCT    5993
E42         AACTTTCTTATCAAAGACGGAATTGCATTTGATCATGTCAACTAGAAATAAACATAATCT    6180
HEINZ       AACTTTCTTATCAAAGACGGAATTGCATTTGATCATGTCAACTAGAAATAAACATAATCT    6180
            ************************************************************

LA2093      GTTGTCATATTAACTAAAAATATGAAGAAAGACATGCAAACATGGGGAAACACAAACGAG    6053
E42         GTTGTCATATTAACTAAAAATATGAAGAAAGACATGCAAATATGGGGAAACACAAACGAG    6240
HEINZ       GTTGTCATATTAACTAAAAATATGAAGAAAGACATGCAAACATGGGGAAACACAAACGAG    6240
            **************************************** *******************

LA2093      AAACAAACAATTTACAGAGCACTCCAGTTTGATGTATTTCCCCATAGAATCAGTTTTAAA    6113
E42         AAACAAACAATTTACAGAGCACTCCAGTTTGATGTATTTCCCCATAGAATCAGTTTTAAA    6300
HEINZ       AAACAAACAATTTACAGAGCACTCCAGTTTGATGTATTTCCCCATAGAATCAGTTTTAAA    6300
            ************************************************************

LA2093      TTAATCACATAAGTTATACAGCACAGAATAAGAGAGAATGCAAGAGCATCAACTTTCAAC    6173
E42         TTAATCACATAAGTTATACAGCACAGAATAAGAGAGAATGCAAGAGCATCAACTTTCAAC    6360
HEINZ       TTAATCACATAAGTTATACAGCACAGAATAAGAGAGAATGCAAGAGCATCAACTTTCAAC    6360
            ************************************************************

LA2093      TAAACCTCGCCCTCAAGCCCTATTTCACCTTTCACATATGCCATTTTGATTTTATTTAAG    6233
E42         TAAACCTCGCCCTCAAGCCCTATTTCACCTTTCACATATGCCATTTTGATTTTATTTAAG    6420
HEINZ       TAAACCTCGCCCTCAAGCCCTATTTCACCTTTCACATATGCCATTTTGATTTTATTTAAG    6420
            ************************************************************

LA2093      AATAGAAACATTTTCTTCATTATTTTGTTCGCCTATCAAATTCTTTACTCTCTCATAAGC    6293
E42         AATAGAAACATTTTCTTCATTATTTTGTTCGCCTATCAAATTCTTTACTCTCTCATAAGC    6480
HEINZ       AATAGAAACATTTTCTTCATTATTTTGTTCGCCTATCAAATTCTTTACTCTCTCATAAGC    6480
            ************************************************************

LA2093      TGAACAGTTGATCCTTCACCTCAACTACATGTGTTAATCATTATTACTTGCATATCATGT    6353
E42         TGAACAGTTGATCCTTCACCTCAACTACATGTGTTAATCATTATTACTTGCATATCATGT    6540
HEINZ       TGAACAGTTGATCCTTCACCTCAACTACATGTGTTAATCATTATTACTTGCATATCATGT    6540
            ************************************************************

LA2093      TAAATTTGAGTAACCAAAAGTTCTGAAAAAGTAAACAAAATAGAAAGAATCACAGGGACC    6413
E42         TAAATTTGAGTAACCAAAAGTTCTGAAAAAGTAAACAAAATAGAAAGAACCACAGGAACC    6600
HEINZ       TAAATTTGAGTAACCAAAAGTTCTGAAAAAGTAAACAAAATAGAAAGAATCACAGGAACC    6600
            ************************************************* ****** ***

LA2093      AAAAAATGTAAAATCAAGTAAAGTTGCTTTTGAGTAATCCTCCCATAGCAAGATCAGCTT    6473
E42         AAAAAATGTAAAATCAAGTAAAGTTGCTTTTGAGTAATCCTCCCATAGCAAGATCAGCTT    6660
HEINZ       AAAAAATGTAAAATCAAGTAAAGTTGCTTTTGAGTAATCCTCCCATAGCAAGATCAGCTT    6660
            ************************************************************

LA2093      GAGAAATAACATGGCAGCAGAAGTGTTTGACCAAACCCTATCCTAGGAATTGGATCGATG    6533
E42         GAGAAATAACATGGCAGCAGAAGTGTTTGACCAAACCCTATCCTAGGAATTGGATCGATG    6720
HEINZ       GAGAAATAACATGGCAGCAGAAGTGTTTGACCAAACCCTATCCTAGGAATTGGATCGATG    6720
            ************************************************************

LA2093      TCAATTTAATGAGGCTTACTTCATGCACAAAGTGCTAATTCACTGATTTGGGGTTAAGGG    6593
E42         TCAATTTAATGAGGCTTACTTCATGCACAAAGTGCTAATTCACTGATTTGGGGTTAAGGG    6780
HEINZ       TCAATTTAATGAGGCTTACTTCATGCACAAAGTGCTAATTCACTGATTTGGGGTTAAGGG    6780
            ************************************************************

LA2093      ACGAGTGAGTGTAACCATATAGGGTTACATTTCTATCTAAGCAAGAGATTTCTTTAACCA    6653
E42         ACGAGTGAGTGTAACCATATAGGGTTACATTTCTATCTAAGCAAGAGATTTCTTTGACCA    6840
HEINZ       ACGAGTGAGTGTAACCATATAGGGTTACATTTCTATCTAAGCAAGAGATTTCTTTGACCA    6840
            ******************************************************* ****

LA2093      ATTTTTCTTGCTTAGCCAAAGTTGATATTAGACCTAGCCTGGTCATATCTCTGGCAAGCT    6713
E42         ATTTTTCTTGCTTAGCCAAAGTTGATATTAGACCTAGCCTGGTCATATCTCTGGCAAGCT    6900
HEINZ       ATTTTTCTTGCTTAGCCAAAGTTGATATTAGACCTAGCCTGGTCATATCTCTGGCAAGCT    6900
```

```
            ************************************************************

LA2093      ACAGTGGTTAAGGTGCCAAAAGTCCTGGCCCCTATGTGCCGATTAATAATCCATCTCAGG    6773
E42         ACAGTGGTTAAGGTGCCAAAAGTCCTGGCCCCTATGTGCCGATTAATAATCCATCTCAGG    6960
HEINZ       ACAGTGGTTAAGGTGCCAAAAGTCCTGGCCCCTATGTGCCGATTAATAATCCATCTCAGG    6960
            ************************************************************

LA2093      GTACATGCACTTATCCAATGTACCCTAAATTTAGTGAAAAGCTGAAAAAAAGAAAAGAAA    6833
E42         GTACATGCACTCATCCAATGTACCCTAAATTTAGTGAAAAGCTGAAAAAAAGAAAAGAAA    7020
HEINZ       GTACATGCACTCATCCAATGTACCCTAAATTTAGTGAAAAGCTGAAAAAAAGAAAAGAAA    7020
            *********** ************************************************

LA2093      TTGCAACAGTAATGCTTAAGTTTTAAAGAAGCAGTTACAGCAAGAACATATTCCTTTGAA    6893
E42         TTGCAACAGTAATGCTTAAGTTTTAAAGAAGCAGTTACAGCAAGAACATATTCCTTTGAA    7080
HEINZ       TTGCAACAGTAATGCTTAAGTTTTAAAGAAGCAGTTACAGCAAGAACATATTCCTTTGAA    7080
            ************************************************************

LA2093      AAGCAGCTCATCACAAGCAAAACATGAATTAGCATAGCTTATGGGTATACAGAACTAAGT    6953
E42         AAGCAGCTCATCACAAGCAAAACATGAATTAGCATAGCTTATGGGTATACAGAACTAAGT    7140
HEINZ       AAGCAGCTCATCACAAGCAAAACATGAATTAGCATAGCTTATGGGTATACAGAACTAAGT    7140
            ************************************************************

LA2093      CTCTCACCCAGAATCAGCATAAAGTTTCCCCAAGCCTTACATATAGTAGACAGGATTATT    7013
E42         CTCTCACCCAGAATCAGCATAAAGTTTCCCCAAGCCTTACATATAGTAGACAGGATTATT    7200
HEINZ       CTCTCACCCAGAATCAGCATAAAGTTTCCCCAAGCCTTACATATAGTAGACAGGATTATT    7200
            ************************************************************

LA2093      CTGATCATCTTTCTCTGGATAAGGTGAAATCATTCACTTATTAACAGCACATAGCAGAGC    7073
E42         CTGATCATCTTTCTCTGGATAAGGTGAAATCATTCACTTATTAACAGCACATAGCAGAGC    7260
HEINZ       CTGATCATCTTTCTCTGGATAAGGTGAAATCATTCACTTATTAACAGCACATAGCAGAGC    7260
            ************************************************************

LA2093      TAGAGATAAATTTCAGAACTAAAACTACCAATTAATTGATGAACTACAAGAAAACAAGAA    7133
E42         TAGAGATAAATTTCAGAACTAAAACTACCAATTAATTGATGAACTACAAGAAAACAAGAA    7320
HEINZ       TAGAGATAAATTTCAGAACTAAAACTACCAATTAATTGATGAACTACAAGAAAACAAGAA    7320
            ************************************************************

LA2093      AGCGTGTGGATAAAAATCAAGCGACAATAAACTTACCTAAAATCCCGATCATCATAAATA    7193
E42         AGCTTGTGGATAAAAATCAAGCGACAATAAACTTACCTAAAATCCCGATCATCATAAATA    7380
HEINZ       AGCTTGTGGATAAAAATCAAGCGACAATAAACTTACCTAAAATCCCGATCATCATAAATA    7380
            *** ********************************************************

LA2093      GATTCAACTGAAATTGGAGCTGCTGTGTTTGCACTAAGAATTTCCTTTGTTCTTTTGACT    7253
E42         GATTCAACTGAAATTGGAGCTGCTGTGTTTGCACTAAGAATTTCCTTTGTTCTTTTGACT    7440
HEINZ       GATTCAACTGAAATTGGAGCTGCTGTGTTTGCACTAAGAATTTCCTTTGTTCTTTTGACT    7440
            ************************************************************

LA2093      TGCTTCTTCAACTTTGCCATTGCCTTTGGAGACTTCCTAATGTCAACCCCATTTCCTACC    7313
E42         TGCTTCTTCAACTTTGCCATTGCCTTTGGAGACTTCCTAATGTCAACCCCATTTCCTACC    7500
HEINZ       TGCTTCTTCAACTTTGCCATTGCCTTTGGAGACTTCCTAATGTCAACCCCATTTCCTACC    7500
            ************************************************************

LA2093      TGTTTATTGAACTCATCTGCAAAGTGTTCCACCAATCTTAGTTCCATATGTTCTCCTCCC    7373
E42         TGTTTATTGAACTCATCTGCAAAGTGTTCCACCAATCTTAGTTCCATATGTTCTCCTCCC    7560
HEINZ       TGTTTATTGAACTCATCTGCAAAGTGTTCCACCAATCTTAGTTCCATATGTTCTCCTCCC    7560
            ************************************************************

LA2093      AGTTCCGCATTCCATCTAACATCCTTGACCTGAAATTGGTTAGCTGATACAGTCTTCCCG    7433
E42         AGTTCCGCATTCCATCTAACATCCTTGACCTGAAATTGGTTAGCTGATACAGTCTTCCCG    7620
HEINZ       AGTTCCGCATTCCATCTAACATCCTTGACCTGAAATTGGTTAGCTGATACAGTCTTCCCG    7620
            ************************************************************

LA2093      AACTCCTTAGTGTTATAAGCAGAGAAATACACCAAGGCAGCATATGTACTACCCGCACCC    7493
E42         AACTCCTTAGTGTTATAAGCAGAGAAATACACCAAGGCAGCATATGTACTACCCGCACCC    7680
HEINZ       AACTCCTTAGTGTTATAAGCAGAGAAATACACCAAGGCAGCATATGTACTACCCGCACCC    7680
            ************************************************************

LA2093      ATATCATAGAAAATCACATGCCTTGAACCATTAGAAAAATCTTTATCAATCCCATACTGC    7553
E42         ATATCATAGAAAATCACATGCCTTGAACCATTCGAAAAATCTTTATCAATCCCATACTGC    7740
HEINZ       ATATCATAGAAAATCACATGCCTTGAACCATTCGAAAAATCTTTATCAATCCCATACTGC    7740
            ******************************** ***************************
```

```
LA2093      AACGCTGCACCAGAATGTTCATTCACAAGCGCCAACACATTAATACCAGCCAATTCCGCA    7613
E42         AACGCTGCACCAGAATGTTCATTCACAAGCGCCAACACATTAATACCAGCCAATTCCGCA    7800
HEINZ       AACGCTGCACCAGAATGTTCATTCACAAGCGCCAACACATTAATACCAGCCAATTCCGCA    7800
            ************************************************************

LA2093      GCAACAAGCAGCCCTTTCCTTTCCGCTACTCCCATATACGGAGGCACAGTCACCACAGCA    7673
E42         GCAACAAGCAGCCCTTTCCTTTCCGCTACTCCCATATACGGAGGCACAGTCACCACAGCA    7860
HEINZ       GCAACAAGCAGCCCTTTCCTTTCCGCTACTCCCATATACGGAGGCACAGTCACCACAGCA    7860
            ************************************************************

LA2093      TCTTTCACCGGTGTTCCCCTAGTATGCGCTTCCGCCAATCCAAGAGCATACTTGAACAAC    7733
E42         TCTTTCACCGGTGTTCCCCTAGTATGCGCTTCCGCCAATCCAAGAGCATACTTGAACAAC    7920
HEINZ       TCTTTCACCGGTGTTCCCCTAGTATGCGCTTCCGCCAATCCAAGAGCATACTTGAACAAC    7920
            ************************************************************

LA2093      ATCGCTACCAACTCCTCCGCCGTAAAATTACCATTTTCAGTTTTAAAAACCGCCACATTC    7793
E42         ATCGCTACCAACTCCTCCGCCGTAAAATTACCATTTTCAGTTTTAAAAACCGCCACATTC    7980
HEINZ       ATCGCTACCAACTCCTCCGCCGTAAAATTACCATTTTCAGTTTTAAAAACCGCCACATTC    7980
            ************************************************************

LA2093      CGCGATTCCTCGGGAGAAATGTCGTAAGTCAGGTAAAGAGATTCTAGGGTTTTGGAAACG    7853
E42         CGCGATTCCTCGGGAGAAATGTCGTAAGTCAGGTAAAGAGATTCTAGGGTTTTGGAAACG    8040
HEINZ       CGCGATTCCTCGGGAGAAATGTCGTAAGTCAGGTAAAGAGATCCTAGGGTTTTGGAAACG    8040
            ****************************************** *****************

LA2093      TGGGGAAAGGGTTTTGATATTAGATCACGGAGATGAGAATAGACCTTGTTTGGATAGCGA    7913
E42         TGGGGAAAGGGTTTTGATATTAGATCACGGAGATGAGAATAGACCTTGTTTGGATAGCGA    8100
HEINZ       TGGGGAAAGGGTTTTGATATTAGATCACGGAGATGAGAATAGACCTTGTTTGGATAGCGA    8100
            ************************************************************

LA2093      GCGACGATTCCAGAAGCTTCTTCACCGATGAGGCGACTCTCGGAATGAAATGCGACGAGT    7973
E42         GCGACGATTCCAGAAGCTTCTTCACCGATGAGGCGACTCTCGGAATGAAATGCGACGAGT    8160
HEINZ       GCGACGATTCCAGAAGCTTCTTCACCGATGAGGCGACTCTCGGAATGAAATGCGACGAGT    8160
            ************************************************************

LA2093      GAGGGAGTTTTCCTTTTGGACATTTCGTTAATCGCTATAGAGATTGGAGGTTGTCCGGGT    8033
E42         GAGGGAGTTTTCCTTTTGGACATTTCGTTAATCGCTATAGAGATTGGAGGTTGTCCGGGT    8220
HEINZ       GAGGGAGTTTTCCTTTTGGACATTTCGTTAATCGCTATAGAGATTGGAGGTTGTCCGGGT    8220
            ************************************************************

LA2093      TTTAGGTTAACCACGGCGACTTTGAACCATTCTGATCCCAGATCTATGCTAGAAACTGCA    8093
E42         TTTAGGTTAACCACGGCGACTTTGAACCATTCTGATCCCAGATCTATGCTAGAAACTGCA    8280
HEINZ       TTTAGGTTAACCACGGCGACTTTGAACCATTCTGATCCCAGATCTATGCTAGAAACTGCA    8280
            ************************************************************

LA2093      GATTGAGATGGAATCGGATTTAGCAAAAACAGGGATAGAATTATCCCTATATGGAACAAC    8153
E42         GATTGAGATGGAATCGGATTTAGCAAAAACAGGGATAGAATTATCCCTATATGGAACAAC    8340
HEINZ       GATTGAGATGGAATCGGATTTAGCAAAAACAGGGATAGAATTATCCCTATATGGAACAAC    8340
            ************************************************************

LA2093      ATATTCTTCGCCGGCATCGGAAAATCGGAGTTAATCGGACAAAGATCTCCGGTGAAAATT    8213
E42         ATATTCTTCGCCGGCATCGGAAAATCGGAGTTAATCGGACAAAGATCTCCGGTGAAAATT    8400
HEINZ       ATATTCTTCGCCGGCATCGGAAAATCGGAGTTAATCGGACAAAGATCTCCGGTGAAAATT    8400
            ************************************************************

LA2093      CAAAAATTGGGACGACGA 8231
E42         CAAAAATTGGGACGACGA 8418
HEINZ       CAAAAATTGGGACGACGA 8418
            ******************
```

Solyc07g047690

```
LA2093      TGGGAACGAACATGGAGGAAGCATTGAAAGCTAAAACAAATGCTGAAAGGAGATTTGTGG     60
E42         TGGGAACGAACATGGAGGAAGCATTGAAAGCTAAAACAAATGCTGAAAGGAGATTCGTGG     60
HEINZ       TGGGAACGAACATGGAGGAAGCATTGAAAGCTAAAACAAATGCTGAAAGGAGATTCGTGG     60
            ******************************************************* ****

LA2093      AGAAAGACATTTTGAGTGCAAAAAATTATGCTCTGAAAGCTCAGATGTTGTACCCTCACT     120
E42         AGAAAGACATTTTGAGTGCAAAAAATTATGCTCTGAAAGCTCAGATGTTGTACCCTCACT     120
HEINZ       AGAAAGACATTTTGAGTGCAAAAAATTATGCTCTGAAAGCTCAGATGTTGTACCCTCACT     120
            ************************************************************

LA2093      TGGAAGGAATATCACAAATGGTAGCGACCTTTGGAGTCCTTAGTGCTGCAGAGACGAAGG     180
E42         TGGAAGGAATATCACAAATGGTAGCGACCTTTGGAGTCCTTAGTGCTGCAGAGACGAAGG     180
HEINZ       TGGAAGGAATATCACAAATGGTAGCGACCTTTGGAGTCCTTAGTGCTGCAGAGACGAAGG     180
            ************************************************************

LA2093      TTAATGGAGAATATGATTTCTATGCAATACTGGGTTTAGATTCCTCTGTTGACAAGGCCA     240
E42         TTAATGGAGAATATGATTTCTATGCAATACTGGGTTTAGATTCCTCTGTTGACAAGGCCA     240
HEINZ       TTAATGGAGAATATGATTTCTATGCAATACTGGGTTTAGATTCCTCTGTTGACAAGGCCA     240
            ************************************************************

LA2093      AGCTGAAGAAACAGTATAAGAAGATGGCTGTGTTACTCCATCCCGATAAGAACAAAAGTG     300
E42         AGCTGAAGAAACAGTATAAGAAGATGGCTGTGTTACTCCATCCCGATAAGAACAAAAGTG     300
HEINZ       AGCTGAAGAAACAGTATAAGAAGATGGCTGTGTTACTCCATCCCGATAAGAACAAAAGTG     300
            ************************************************************

LA2093      TTGGAGCTGATGGTGCATTTAGACTTGTCTCTGAAGCATGGACGGTGTTGTCTGATGTTT     360
E42         TTGGAGCTGATGGTGCATTTAGACTTGTCTCTGAAGCATGGACGGTGTTGTCTGATGTTT     360
HEINZ       TTGGAGCTGATGGTGCATTTAGACTTGTCTCTGAAGCATGGACGGTGTTGTCTGATGTTT     360
            ************************************************************

LA2093      CCAAAAGAAGCTCGTATGATCACAGGAGAAGTTTATTTACTCTGCATGGTTCTGGTGTTG     420
E42         CCAAAAGAAGCTCGTATGATCACAGGAGAAGTTTATTTACTCTGCATGGTTCTGGTGTTG     420
HEINZ       CCAAAAGAAGCTCGTATGATCACAGGAGAAGTTTATTTACTCTGCATGGTTCTGGTGTTG     420
            ************************************************************

LA2093      GGAGCTACGACAGTTACTCCAATTCATCAGTTTCTCATAACAGGCTGGATACATTTTGGA     480
E42         GGAGCTATGACAGTTACTCCAATTCATCAGTTTCTCATAACAGGCTGGATACATTTTGGA     480
HEINZ       GGAGCTATGACAGTTACTCCAATTCATCAGTTTCTCATAACAGGCTGGATACATTTTGGA     480
            ******* ****************************************************

LA2093      CCGTTTGTACCTCTTGTCACGTTCAGTATGAATATCTTAGGAAGTACCTGAACAAAAGGC     540
E42         CCGTTTGTACCTCTTGTCACGTTCAGTATGAATATCTTAGGAAGTACCTGAACAAAAGGC     540
HEINZ       CCGTTTGTACCTCTTGTCACGTTCAGTATGAATATCTTAGGAAGTACCTGAACAAAAGGC     540
            ************************************************************

LA2093      TGTCCTGTAAGAACTGTCGTGGGGTTTTCATAGCTGCTGAAACAGGATTGGCTCCGGTTA     600
E42         TGTCCTGTAAGAACTGTCGTGGGGTTTTCATAGCTGCTGAAACAGGATTGGCTCCGGTTA     600
HEINZ       TGTCCTGTAAGAACTGTCGTGGGGTTTTCATAGCTGCTGAAACAGGATTGGCTCCGGTTA     600
            ************************************************************

LA2093      ATGGTTCCTATTCCTATAGCTCTTGGTCTAATGGATATGGAAGCCATGGTTGCGGGGCTA     660
E42         ATGGTTCCTATTCCTATAGCTCTTGGTCTAATGGATATGGAAGCCATGGTTGCGGGGCTA     660
HEINZ       ATGGTTCCTATTCCTATAGCTCTTGGTCTAATGGATATGGAAGCCATGGTTGCGGGGCTA     660
            ************************************************************

LA2093      CATATGTCCCAACAACATCTGTTTATGCTGCAAATAATGGGGTCTCAGGACATCATTCCG     720
E42         CATATGTCCCAACAACATCTGTTTATGCTGCAAATAATGGGGTCTCAGGACATCATTCCG     720
HEINZ       CATATGTCCCAACAACATCTGTTTATGCTGCAAATAATGGGGTCTCAGGACATCATTCCG     720
            ************************************************************

LA2093      GACCTTGTTCCGAGCATGTCTCCAATTTGTCCTTTCAGTGGAGCTCCAGTGGAAATTCTG     780
E42         GACCTTGTTCCGAGCATGTCTCCAATTTGTCCTTTCAGTGGAGCTCCAGTGGAAATTCTG     780
HEINZ       GACCTTGTTCCGAGCATGTCTCCAATTTGTCCTTTCAGTGGAGCTCCAGTGGAAATTCTG     780
            ************************************************************

LA2093      CTCCAGTTTTAGATTCCAATGGATCATCCACAGCTGTCAGTTTTGCAAACCAAGCAAGTA     840
E42         CTCCAGTTTTAGATTCCAATGGATCATCCACAGCTGTCAGTTTTGCAAACCAAGCAAGTA     840
HEINZ       CTCCAGTTTTAGATTCCAATGGATCATCCACAGCTGTCAGTTTTGCAAACCAAGCAAGTA     840
            ************************************************************
```

```
LA2093      GGAAAATCACCAGAAGAAGAGGCAGAGGGAAGCATGAAGTGAAAAAGATGGTGAGCAACG    900
E42         GGAAAATCACCAGAAGAAGAGGCAGAGGGAAGCATGAAGTGAAAAAGATGGTGAGCAACG    900
HEINZ       GGAAAATCACCAGAAGAAGAGGCAGAGGGAAGCATGAAGTGAAAAAGATGGTGAGCAACG    900
            ************************************************************

LA2093      GGGTTCTTAACGGGCATACTGTGTGCAATGAACAAATCCCTCGCAGGCCTGGTAGACCTC    960
E42         GGGTTCTTAACGGGCATACTGTGTGCAATGAACAAATCCCTCGCAGGCCTGGTAGACCTC    960
HEINZ       GGGTTCTTAACGGGCATACTGTGTGCAATGAACAAATCCCTCGCAGGCCTGGTAGACCTC    960
            ************************************************************

LA2093      CTAAGAAGATAAAAATTGGCGTGGAAGGTACATACAGCTATAGTAATGGAGAAATGGCTC    1020
E42         CTAAGAAGATAAAAATTGGCGTGGAAGGTACATACAGCTATAGTAATGGAGAAATGGCTC    1020
HEINZ       CTAAGAAGATAAAAATTGGCGTGGAAGGTACATACAGCTATAGTAATGGAGAAATGGCTC    1020
            ************************************************************

LA2093      TAAAAACTGCTGGAGAAGTTAAAATGGCTGATGGAAATGGGGACGGGAATTTGAAACAAA    1080
E42         TAAAAACTGCTGGAGAAGTTAAAATGGCTGATGGAAATGGGGACGGGAATTTGAAACAAA    1080
HEINZ       TAAAAACTGCTGGAGAAGTTAAAATGACTGATGGAAATGGGGACGGGAATTTGAAACAAA    1080
            ************************** *********************************

LA2093      ATATTAAGCTTCTAAATCCTGCTGAAGCTTCAATAAAAAGACATTCCGCTGCTCCTGCAT    1140
E42         ATATTAAGCTTCTAAATCCTGCTGAAGCTTCAATAAAAAGACATTCCGCTGCTCCTGCAT    1140
HEINZ       ATATTAAGCTTCTAAATCCTGCTGAAGCTTCAATAAAAAGACATTCCGCTGCTCCTGCAT    1140
            ************************************************************

LA2093      TTGATGCAAGACGGTTACTAATTGACAAGGCAAGAGCAGAAATCCGCAAGAAACTGGAAG    1200
E42         TTGATGCAAGACGGTTACTAATTGACAAGGCAAGAGCAGAAATCCGCAAGAAACTGGAAG    1200
HEINZ       TTGATGCAAGACGGTTACTAATTGACAAGGCAAGAGCAGAAATCCGCAAGAAACTGGAAG    1200
            ************************************************************

LA2093      AGATCAAGTTGTCTTATGAAGCTGCTGCTGCAGAGACCGAGAAGAAGAGAAAGGCGGATG    1260
E42         AGATCAAGTTGGCTTATGAAGCTGCTGCTGCAGAGACCGAGAAGAAGAGAAAGGCGGATG    1260
HEINZ       AGATCAAGTTGGCTTATGAAGCTGCTGCTGCAGAGACCGAGAAGAAGAGAAAGGTGGATG    1260
            *********** ****************************************** *****

LA2093      CTGAGTTTGGTGAATCGGGTGAGAGACCTAAAGGAGCAGTGCAGGAAGTT---GTTCATC    1317
E42         CTGAGTTTGGTGAATCGGGTGAGAGACCTAAAGGAGCAGTGCAGGAAGTTGTTGTTCATC    1320
HEINZ       CTGAGTTTGGTGAATCGGGTGAGAGACCTAAAGGAGCAGTGCAGGAAGTTGTTGTTCATC    1320
            **************************************************   *******

LA2093      AATCAGAACTGGGGAAAACTGGGTCCATGACAATAGTAGTCCCAGATTCTGACTTCCATG    1377
E42         AATCAGAACTGGGGAAAACTGGGTCCATGACAATAGTAGTCCCAGATTCTGACTTCCATG    1380
HEINZ       AATCAGAACTGGGGAAAACTGGGTCCATGACAATAGTAGTCCCAGATTCTGACTTCCATG    1380
            ************************************************************

LA2093      ATTTTGACAAGGATAGATCAGAAGATTGCTTCAGACCTAAGCAGATATGGGCTGTATATG    1437
E42         ATTTTGACAAGGATAGATCAGAAGATTGCTTCAGACCTAAGCAGATATGGGCTGTATATG    1440
HEINZ       ATTTTGACAAGGATAGATCAGAAGATTGCTTCAGACCTAAGCAGATATGGGCTGTATATG    1440
            ************************************************************

LA2093      ATGAGGAAGATGGTATGCCTCGCTTGTATTGTTTGATTCGTGAAATCATATCTGTGAAAC    1497
E42         ATGAGGAAGATGGTATGCCTCGCTTGTATTGTTTGATTCGTGAAATCATATCTGTGAAAC    1500
HEINZ       ATGAGGAAGATGGTATGCCTCGCTTGTATTGTTTGATTCGTGAAATCATATCTGTGAAAC    1500
            ************************************************************

LA2093      CATTCAAAGTTCATATCAGTTACATAAGCTCAAAGACAGATAGCGAATTTGGGCTGGTCA    1557
E42         CATTCAAAGTTCATATCAGTTACATAAGCTCAAAGACAGATAGCGAATTTGGGCTGGTCA    1560
HEINZ       CATTCAAAGTTCATATCAGTTACATAAGCTCAAAGACAGATAGCGAATTTGGGCTGGTCA    1560
            ************************************************************

LA2093      ATTGGTTGGATTCTGGTTTTACAAAGTCATGTGGAAATTTTAGGGCCTTCAACTCTGAAA    1617
E42         ATTGGTTGGATTCTGGTTTTACAAAGTCATGTGGAAATTTTAGGGCCTTCAACTCTGAAA    1620
HEINZ       ATTGGTTGGATTCTGGTTTTACAAAGTCATGTGGAAATTTTAGGGCCTTCAACTCTGAAA    1620
            ************************************************************

LA2093      TTGTTGAGCATGTAAACATATTCTCTCACCTGCTAAGTAAGGAGAAAGTTGGAAGGGGAG    1677
E42         TTGTTGAGCATGTAAACATATTCTCTCACCTGCTAAGTAAGGAGAAAGTTGGAAGGGGAG    1680
HEINZ       TTGTTGAGCATGTAAACATATTCTCTCACCTGCTAAGTAAGGAGAAAGTTGGAAGGGGAG    1680
            ************************************************************

LA2093      GTAGTATTAGAATCTACCCAAAAAGTGGAGACATTTGGGCTGTATATCGAAATTGGTCAC    1737
```

```
E42         GTAGTATTAGAATCTACCCAAAAAGTGGAGACATTTGGGCTGTATATCGAAATTGGTCAC    1740
HEINZ       GTAGTATTAGAATCTACCCAAAAAGTGGAGACATTTGGGCTGTATATCGAAATTGGTCAC    1740
            ************************************************************

LA2093      CAGATTGGGACGGAACCACCCCAGCTGAAGTAAGGCACCAGTATGAAATGGTTGAGGTTC    1797
E42         CAGATTGGGACGGAACCACCCCAGCTGAAGTAAGGCACCAGTATGAAATGGTTGAGGTTC    1800
HEINZ       CAGATTGGGACGAAACCACCCCAGCTGAAGTAAGGCACCAGTATGAAATGGTTGAGGTTC    1800
            ************ ***********************************************

LA2093      TTGATGATTATTCTGAAGAGCTTGGTGTCTGTGTTACTCCTTTGGTTAAACTTGATGGGT    1857
E42         TTGATGATTATTCTGAAGAGCTTGGTGTCTGTGTTACTCCTTTGGTTAAACTTGATGGGT    1860
HEINZ       TTGATGATTATTCTGAAGAGCTTGGTGTCTGTGTTACTCCTTTGGTTAAACTTGATGGGT    1860
            ************************************************************

LA2093      TCAAGACAGTATACTCTAGAAACACTAACAAAGATGCAATTCGATTGATTCCAAGAAGAG    1917
E42         TCAAGACAGTATACTCTAGAAACACTAACAAAGATGCAATTCGATTGATTCCAAGAAGAG    1920
HEINZ       TCAAGACAGTATACTCTAGAAACACTAACAAAGATGCAATTCGATTGATTCCAAGAAGAG    1920
            ************************************************************

LA2093      AGATGTTACGATTTTCACATCAGGTACCATCTTGCTTACTGAAAGGTGAAAGAATGAACC    1977
E42         AGATGTTACGATTTTCACATCAGGTACCATCTTGCTTACTGAAAGGTGAAAGAATGAACC    1980
HEINZ       AGATGTTACGATTTTCACATCAGGTACCATCTTGCTTACTGAAAGGTGAAAGAATGAACC    1980
            ************************************************************

LA2093      TGCCTGAGGGGTGCTGGGATCTTGACCCTGCTGCAATTCCAGAAGATTTACTTCAGCGAG    2037
E42         TGCCTGAGGGGTGCTGGGATCTTGACCCTGCTGCAATTCCAGAAGATTTACTTCAGCGAG    2040
HEINZ       TGCCTGAAGGGTGCTGGGATCTTGACCCTGCTGCAATTCCAGAAGATTTACTTCAGCGAG    2040
            ******* ****************************************************

LA2093      TAAATGACGCCAAGGAGGAAAGAACTACTGAAGCTGAAAGCCCAGTGGGATTTGATCTAA    2097
E42         TAAATGACGCCAAGGAGGAAAGAACTACTGAAGCTGAAAGCCCAGTGGGATTTGATCTAA    2100
HEINZ       TAAATGACGCCAAGGAGGAAAGAACTACTGAAGCTGAAAGCCCAGTGGGATTTGATCTAA    2100
            ************************************************************

LA2093      ATGTGACATCTCAGGCTGAAACCAAAATGTTGATGGAAGAAGAACTTGGACAAACAGAAT    2157
E42         ATGTGACATCTCAGGCTGAAACCAAAATGTTGATGGAAGAAGAACTTGGACAAACAGAAT    2160
HEINZ       ATGTGACATCTCAGGCTGAAACCAAAATGTTGATGGAAGAAGAACTTGGACAAACAGAAT    2160
            ************************************************************

LA2093      ATCCTGGTGTTCCTGATGAGCTCCATGACTCTAGATGTGGTTTGCAGATTCAAGATATCT    2217
E42         ATCCTGGTGTTCCTGATGAGCTCCATGGCTCTAGATGTGGTTTGCAGATTCAAGATATCT    2220
HEINZ       ATCCTGGTGTTCCTGATGAGCTCCATGGCTCTAGATGTGGTTTGCAGATTCAAGATATCT    2220
            *************************** ********************************

LA2093      CGAATGATCCACAAAACCTGTTCAGGATTTCCACTGAGCTTCCTCAATCTGTAAAGGAAG    2277
E42         CGAATGATCCACAAAACCTGTTCAGGATTTCCACTGAGCTTCCTCAATCTGTAAAGGAAG    2280
HEINZ       CGAATGATCCACAAAACCTGTTCAGGATTTCCACTGAGCTTCCTCAATCTGTAAAGGAAG    2280
            ************************************************************

LA2093      TTCATAGTTGTGAAGAACCCAGCAGAATGGAAAAAAATTTCTCTGCTACACCAGGTGAGC    2337
E42         TTCATAGTTGTGAAGAACCCAGCAGAATGGAAAAAAATTTCTCTGCTACACCAGGTGAGC    2340
HEINZ       TTCATAGTTGTGAAGAACCCAGCAGAATGGAAAAAAATTTCTCTGCTACACCAGGTGAGC    2340
            ************************************************************

LA2093      ATTTTCGGGCTGTAACAGAGGTATAG2363
E42         ATTTTCGGGCTGTAACAGAGGTATAG2366
HEINZ       ATTTTCGGGCTGTAACAGAGGTATAG2366
            **************************
```

Solyc07g053615

```
HEINZ       TGTTTAAAACCAACTATGCTCAAGTGAAGAGAGCTACGAGGGTTATATCTGCTGAAGCTC     60
E42         TGTTTAAAACCAACTATGCTCAAGTGAAGAGAGCTACGAGGGTTATATCTGCTGAAGCTC     60
LA2093      TGTTTAAAACCAACTATGCTCAAGTGAAGAGAGCTACGAGGGTTATATCTGCTGAAGCTC     60
            ************************************************************

HEINZ       GTGAATATGAAAAGCTAGAACCAGATTATTCAGTTAAGGTAGGAAGCGGACATGCTAATT     120
E42         GTGAATATGAAAAGCTAGAACCAGATTATTCAGTTAAGGTAGGAAGCGGACATGCTAATT     120
LA2093      GTGAATATGAAAAGCTAGAACCAGATTATTCAGTTAAGGTAGGAAGCGGACATGCTAATT     120
            ************************************************************

HEINZ       CAGGACAAGGGCGTAATCGTGTAAGAGGAGGAGCTCATTGTCGCGTCCATGGAGGCAAAT     180
E42         CAGGACAAGGGCGTAATCGTGTAAGAGGAGGAGCTCATTGTCGCGTCCGTGGAGGCAAAT     180
LA2093      CAGGACAAGGGCGTAATCGTGTAAGAGGAGGAGCTCATTGTCGCGTCCGTGGAGGCAAAT     180
            ************************************************ ***********

HEINZ       CTCAGAGAAAAGTCAATGCATCCAGATCTGACTCTGCGCAGAGAAGTTCAACAAAGAACA     240
E42         CTCAGAGAAAAGTCAATGCATCCAGATCTGACTCTGCGCAGAGAAGTTCAACAAAGAACA     240
LA2093      CTCAGAGAAAAGTCAATGCATCCAGATCTGACTCTGCGCAGAGAAGTTCAACAAAGAACA     240
            ************************************************************

HEINZ       GTGACAGACTAGGCCACCTTCCTGCCTGGAAAGGTCAAGACCGCGGCAAAGGAAGACGTA     300
E42         GTGACAGACTAGGCCACCTTCCTGCCTGGAAAGGTCAAGACCGCGGCAAAGGAAGACGTA     300
LA2093      GTGATAGACTAGGCCACCTTCCTGCCTGGAAAGGTCAAGACCGCGGCAAAGGAAGACGTA     300
            **** *******************************************************

HEINZ       AAAGAGGTCGTCGCTCAGTTCGTAATAGACAAAAGCCAGTCAAGAATGTGGAAGAAGTTT     360
E42         AAAGAGGTCGTCGCTCAGTTCGTAATAGACAAAAGCCAGTCAAGAATGTGGAAGAAGTTT     360
LA2093      AAAGAGGTCGTCGCTCAGTTCGTAATAGACAAAAGCCAGTCAAGAATGTGGAAGAAGTTT     360
            ************************************************************

HEINZ       CGCCCGAAGAAGTGCCCATCACCAGTCAGCAAGATTGGAATGATGTTGAAGATGAAGAAA     420
E42         CGCCCGAAGAAGTGCCCATCACCAGTCAGCAAGATTGGAATGATGTTGAAGATGAAGAAA     420
LA2093      CGCCCGAAGAAGTGCCCATCACCAGTCAGCAAGATTGGAATGATGTTGAAGATGAAGAAA     420
            ************************************************************

HEINZ       CACCTCAATTTGAGGCGCCTGATAACGACAGTGATTCTGGAACATCAGGAAGTGAAGATT     480
E42         CACCTCAATTTGAGGCGCCTGATAACGACAGTGATTCTGGAACATCAGGAAGTGAAGATT     480
LA2093      CACCTCAATTTGAGGCGCCTGATAACGACAGTGATTCTGGAACATCAGGAAGTGAAGATT     480
            ************************************************************

HEINZ       ATAAAGGCCAAACAACCGTCAATGATTATGAAGACTTAATGGTTGCTGACTACGGTTCTT     540
E42         ATAAAGGCCAAACAACCGTCAATGATTATGAAGACTTAATGGTTGCTGACTACGGTTCTT     540
LA2093      ATAAAGGCCAAACAACCGTCAATGATTATGAAGACTTAATGGTTGCTGACTACGGTTCTT     540
            ************************************************************

HEINZ       TCAGTGGTAGAAATGATCACGCATCAACCAGTGTGAGTTACAACATCAGCCAACGTTATA     600
E42         TCAGTGGTAGAAATGATCACGCATCAACCAGTGTGAGTTACAACATCAGCCAACGTTATA     600
LA2093      TCAGTGGTAGAAATGATCACGCATCAACCAGTGTGAGTTACAACATCAGCCAACGTTATA     600
            ************************************************************

HEINZ       CTGAAACTGCTGAAGATGGTATTGGTGATTACGAGGATGACCATGATGAAGAGGACGAGG     660
E42         CTGAAACTGCTGAAGATGGTATTGGTGATTACGAGGATGACCATGATGAAGAGGACGAGG     660
LA2093      CTGAAACTGCTGAAGATGGTATTGGTGATTACGAGGATGACCATGATGAAGAGGACGAGG     660
            ************************************************************

HEINZ       AAGATGGATTGGCCAATAAAAACGTCCAAAGATACTTTGATGGCGAGTCCGATGATGAGG     720
E42         AAGATGGATTGGCCAATAAAAACGTCCAAAGATACTTTGATGGCGAGTCCGATGATGAGG     720
LA2093      AAGATGGATTGGCCAATAAAAACGTCCAAAGATACTTTGATGGCGAGTCCGATGATGAGG     720
            ************************************************************

HEINZ       GGGACAGATTTATGGATGAAGATCTTGTTGAAACCCCAAATAAAGATTCAGAATCATCAT     780
E42         GGGACAGATTTATGGATGAAGATCTTGTTGAAACCCCAAATAAAGATTCAGAATCATCAT     780
LA2093      GGGACAGATTTATGGATGAAGATCTTGTTGAAACCCCAAATAAAGATTCAGAATCATCAT     780
            ************************************************************

HEINZ       CCGAGTATAGTGATTGACAACAGTTATTGAACCCAAAGGTATTTTCTTCTCATGAGCGCG     840
E42         CCGAGTATAGTGATTGACAACAGTTATTGAACCCAAAGGTATTTTCTTCTCATGAGCGCG     840
LA2093      CCGAGTATAGTGATTGACAACAGTTATTGAAACCAAAGGTATTTTCTTCTCATGAGCGCG     840
            ******************************* ****************************
```

```
HEINZ       AAAGGCGTCTTTCAATCAGTGCTCGCGTTGACACAGTTGAAGAGCGCCAATGTTTGTTAT    900
E42         AAAGGCGTCTTTCAATCAGTGCTCGCGTTGACACAGTTGAAGAGCGCCAATGTTTGTTAT    900
LA2093      AAAGGCGTCTTTCAATCAGTGCTCGCGTTGACACAGTTGAAGAGCGCCAATGTTTGTTAT    900
            ************************************************************

HEINZ       GAAAGGATCATTAGTTCTAATTCTAACTGTTTATAGTTAGCATCCAACTTGAAAATAGTA    960
E42         GAAAGGATCATTAGTTCTAATTCTAACTGTTTATAGTTAGCATCCAACTTGAAAATAGTA    960
LA2093      GAAAGGATCATTAGTTCTAATTCTAACTGTTTATAGTTAGCATCCAACTTGAAAATAGTA    960
            ************************************************************

HEINZ       CAGTGCTT---CTCTTTACACTTAACATATGTATTACTATTACATGGCAAGTAACTTAAT    1017
E42         CAGTGCTTCTTCTCTTTACACTTAACATATGTATTACTATTACATGGCAAGTAACTTAAT    1020
LA2093      CAGTGCTTCTTCTCTTTACACTTAACATATGTATTACTATTACATGGCAAGTAACTTAAT    1020
            ********   *************************************************

HEINZ       GTGGTAATATATCTAGCGTTAAGTAATATATCTAGCGTTCTTAACCCGCGAATGTCATCG    1077
E42         GTGGTAAT-------------------ATATCTAGCGTTCTTAACCCGCGAATGTCATCG    1061
LA2093      GTGGTAAT-------------------ATATCTAGCGTTCTTAACCCGCGAATGTCATCG    1061
            ********                   *********************************

HEINZ       ATACTCACAGGTTGCAAAGTAAAGGAGGGTTGGTACACTTGAAGAATTCAATTTCATCAA    1137
E42         ATACTCACAGGTTGCAAAGTAAAGGAGGGTTGGTACACTTGAAGAATTCAATTTCATCAA    1121
LA2093      ATACTCACAGGTTGCAAAGTAAAGGAGGGTTGGTACACTTGAAGAATTCAATTTCATCAA    1121
            ************************************************************

HEINZ       GAAATTCACACATGCAGTTAACTTAAATCCAATCTCTCTATTGCTGAAAACATTCATAAC    1197
E42         GAAATTCACACATGCAGTTAACTTAAATCCAATCTCTCTATTGCTGAAAACATTCATAAC    1181
LA2093      GAAATTCACACATGCAGTTAACTTAAATCCAATCTCTCTATTGCTGAAAACATTCATAAC    1181
            ************************************************************

HEINZ       AGACAACATGTGAGATATCACTAATGATTCAACAACCAAAAGGCCTGTGAATTTTCAAGA    1257
E42         AGACAACATGTGAGATATCACTAATGATTCAACAACCAAAAGGCCTGTGAATTTTCAAGA    1241
LA2093      AGACAACATGTGAGATATCACTAATGATTCAACAACCAAAAGGCCTGTGAATTTTCAAGA    1241
            ************************************************************

HEINZ       CTTATTCCAAAAAAAGTAAATGTATTGCCTGTTGTTGAATGGATATACTGAACAATACCC    1317
E42         CTTATTCCAAAAAAAGTAAATGTATTGCCTGTTGTTGAATGGATATACTGAACAATACCC    1301
LA2093      CTTATTCCAAAAAAAGTAAATGTATTGCCTGTTGTTGAATGGATATACTGAACAATACCC    1301
            ************************************************************

HEINZ       ATAATAAACAGAAACAAAATTCTACTATGCTACGAAAGAGACTCGCCTACGAGCCTAAAC    1377
E42         ATAATAAACAGAAACAAAATTCTACTATGCTACGAAAGAGACTCGCCTACGAGCCTAAAC    1361
LA2093      ATAATAAACAGAAACAAAATTCTACTATGCTACGAAAGAGACTCGCCTACGAGCCTAAAC    1361
            ************************************************************

HEINZ       CAACGCCCCATCCCATAGCTCCAAATGGTTTAAAGAAAATATTTACACAGAACAAGACCA    1437
E42         CAACGCCCCATCCCATAGCTCCAAATGGTTTAAAGAAAATATTTACACAGAACAAGACCA    1421
LA2093      CAACGCCCCATCCCATAGCTCCAAATGGTTTAAAGAAAATATTTACACAGAACAAGACCA    1421
            ************************************************************

HEINZ       GCACCAGAGTTGTGCATAAAGCTCAAGAACCCAACAGCTCTTTGATACCAGATTTCTGAG    1497
E42         GCACCAGAGTTGTGCATAAAGCTCAAGAACCCAACAGCTCTTTGATACCAGATTTCTGAG    1481
LA2093      GCACCAGAGTTGTGCATAAAGCTCAAGAACCCAACAGCTCTTTGATACCAGATTTCTGAG    1481
            ************************************************************

HEINZ       CCACAGTGAGACGAGCAGGGAACTTGATGTCAAACTTGATCCTCAAATTCCCTTTCTTTG    1557
E42         CCACAGTGAGACGAGCAGGGAACTTGATGTCAAACTTGATCCTCAAATTCCCTTTCTTTG    1541
LA2093      CCACAGTGAGACGAGCAGGGAACTTGATGTCAAACTTGATCCTCAAATTCCCTTTCTTTG    1541
            ************************************************************

HEINZ       ACGGATCTTTTGGAAGCGGCATGCCTTCACCTGGGACGATATGTTCATAATTTGGTTGAA    1617
E42         ACGGATCTTTTGGAAGCGGCATGCCTTCACCTGGGACGATATGTTCATAATTTGGTTGAA    1601
LA2093      ACGGATCTTTTGGAAGCGGCATGCCTTCACCTGGGACGATATGTTCATAATTTGGTTGAA    1601
            ************************************************************

HEINZ       TCACGTTGTTGATTGGAATTGTTAAGTTCCTTCCATCCAGAGTAGTGAGTTGGACAGTAG    1677
E42         TCACGTTGTTGATTGGAATTGTTAAGTTCCTTCCATCCAGAGTAGTGAGTTGGACAGTAG    1661
LA2093      TCACGTTGTTGATTGGAATTGTTAAGTTCCTTCCATCCAGAGTAGTGAGTTGGACAGTAG    1661
            ************************************************************

HEINZ       TACCGGTTAAAGCTTCAGCCAAAGGAATCTTTTGTGTGACAATCAAGTCATTTCCATCAC    1737
```

```
E42            TACCGGTTAAAGCTTCAGCCAAAGGAATCTTTTGTGTGACAATCAAGTCATTTCCATCAC     1721
LA2093         TACCGGTTAAAGCTTCAGCCAAAGGAATCTTTTGTGTGACAATCAAGTCATTTCCATCAC     1721
               ************************************************************

HEINZ          GCGAGAAAACCTTATGAGGTTTCTCATCTATTATGAAGACCAGATCAGCTGGTATAACAC     1797
E42            GCGAGAAAACCTTATGAGGTTTCTCATCTATTATGAAGACCAGATCAGCTGGTATAACAC     1781
LA2093         GCGAGAAAACCTTATGAGGTTTCTCATCTATTATGAAGACCAGATCAGCTGGTATAACAC     1781
               ************************************************************

HEINZ          CTGGCTGCTCATTCCCTTTTTCTTGGAAGGTGATCTTTGTACCTTTCTTCCACCCAGGTT     1857
E42            CTGGCTGCTCATTCCCTTTTTCTTGGAAGGTGATCTTTGTACCTTTCTTCCACCCAGGTT     1841
LA2093         CTGGCTGCTCATTCCCTTTTTCTTGGAAGGTGATCTTTGTACCTTTCTTCCACCCAGGTT     1841
               ************************************************************

HEINZ          TAATTTCGATTGTCAGAATCTCTTGCACAATTCTCTTGCTGCACCCAACACAAGGATACA     1917
E42            TAATTTCGATTGTCAGAATCTCTTGCACAATTCTCTTGCTGCACCCAACACAAGGATACA     1901
LA2093         TAATTTCGATTGTCAGAATCTCTTGCACAATTCTCTTGCTGCACCCAACACAAGGATACA     1901
               ************************************************************

HEINZ          GGATAAGTTCAGTAGACAAACAGATATCAAAAGCACAATGTGAGTAAATCTAGTAAAGCC     1977
E42            GGATAAGTTCAGTAGACAAACAGATATCAAAAGCACAATGGGAGTAAATCTAGTAAAGCC     1961
LA2093         GGATAAGTTCAGTAGACAAACAGATATCAAAAGCACAATGGGAGTAAATCTAGTAAAGCC     1961
               **************************************** *******************

HEINZ          ACTGGCGAAACAACCACTTAAAATGAGAGCCTCAAATGGAATTCAGAGCGCAAATGATTA     2037
E42            ACTGGCGAAACAACCACTTAAAATGAGAGCCTCAAATGGAATTCAGAGCGCAAATGATTA     2021
LA2093         ACTGGCGAAACAACCACTTAAAATGAGAGCCTCAAATGGAATTCAGAGCGCAAATGATTA     2021
               ************************************************************

HEINZ          AATCGGAAATGCATCCTGATACGACAAATAACTAGTGCAAAGACACAGGAAATAAGTCAA     2097
E42            AATCGGAAATGCATCCTGATACGACAAATAACTAGTGCAAAGACACAGGAAATAAGTCAA     2081
LA2093         AATCGGAAATGCATCCTGATACGACAAATAACTAGTGCAAAGACACAGGAAATAAGTCAA     2081
               ************************************************************

HEINZ          CTTAATGACACTTGACAGACAACAATGTGTATTCAGACTCTCAAGAAATTGACCTCAAAA     2157
E42            CTTAATGACACTTGACAGACAACAATGTGTATTCAGACTCTCAAGAAATTGACCTCAAAA     2141
LA2093         CTTAATGACACTTGACAGACAACAATGTGTATTCAGACTCTCAAGAAATTGACCTCAAAA     2141
               ************************************************************

HEINZ          TTTTCCTTACAGAGATTTCAAAGACGTTATATTCACTTACAGCTTTCAATATAAATGAAT     2217
E42            TTTTCCTTACAGAGATTTCAAAGACGTTATATTCACTTACAGCTTTCAATATAAATGAAT     2201
LA2093         TTTTCCTTACAGAGATTTCAAAGACGTTATATTCACTTACAGCTTTCAATATAAATGAAT     2201
               ************************************************************

HEINZ          TTGAAGGACCCAACTCGACCTAAAATGCAATATCACTACATCTAGGCTTTATCGATCGTA     2277
E42            TTGAAGGACCCAACTCGACCTAAAATGCAATATCACTACATCTAGGCTTTATCGATCGTA     2261
LA2093         TTGAAGGACCCAACTCGACCTAAAATGCAATATCACTACATCTAGGCTTTATCGATCGTA     2261
               ************************************************************

HEINZ          GCTCAAACTGTAATCATGCATGATAGTACTCTTATTTCAACTAGACAAGATTTGACTGGC     2337
E42            GCTCAAACTGTAATCATGCATGATAGTACTCTTATTTCAACTAGACAAGATTTGACTGGT     2321
LA2093         GCTCAAACTGTAATCATGCATGATAGTACTCTTATTTCAACTAGACAAGATTTGACTGGC     2321
               ***********************************************************

HEINZ          TAAATAGTCACAAAATGGCACAAGTAACTTATCAAAGACTTCATGTTCTAAAAGGAAAAT     2397
E42            TAAATAGTCACAAAATGGCACAAGTAATTTATCAAAGACTTCACGTTCTAAAAGGAAAAT     2381
LA2093         TAAATAGTCACAAAATGGCACAAGTAATTTATCAAAGACTTCACGTTCTAAAAGGAAAAT     2381
               *************************** *************** ****************

HEINZ          CAAAGCAATTCGCAACTAACAAGGGAATAATCAACAGGTTTACCCTCAGCGGCCCAGCGC     2457
E42            CAAAGCAATTCGCAACTAACAAGGGAATAATCAACAGGTTTACCCTCAGCGGCCCAGCGC     2441
LA2093         CAAAGCAATTCGCAACTAACAAGGGAATAATCAACAGGTTTACCCTCAGCGGCCCAGCGC     2441
               ************************************************************

HEINZ          ACAAAGCCTCCCACATCCATTCAAAGTCTGGAGAAGTGCCACACCACAAGGGTGTGATGC     2517
E42            ACAAAGCCTCCCACATCCATTCAAAGTCTGGAGAAGTGCCACACCACAAGGGTGTGATGC     2501
LA2093         ACAAAGCCTCCCACATCCATTCAAAGTCTGGAGAAGTGCCACACCACAAGGGTGTGATGC     2501
               ************************************************************

HEINZ          AGACAGCCTACCCAAATGCAAGCATTAGTGGCTGCCTACCCTAATCTTTCAAATCTCTCA     2577
E42            AGACAGCCTACCCAAATGCAAGCATTAGTGGCTGCCTACCCTAATCTTTCAAATCTCTCA     2561
LA2093         AGACAGCCTACCCAAATGCAAGCATTAGTGGCTGCCTACCCTAATCTTTCAAATCTCTCA     2561
```

```
            ************************************************************

HEINZ       AAATTAATGACATAAGAAAACTACTACCCCAAGCATTCCATTATCCTCAATATTTTCCAG    2637
E42         AAATTAATGACATAAGAAAACTACTACCCCAAGCATTCCATTATCCTCAATATTTTCCAG    2621
LA2093      AAATTAATGACATAAGAAAACTACTACCCCAAGCATTCCATTATCCTCAATATTTTCCAG    2621
            ************************************************************

HEINZ       ACACTTGCGAAGAAATAGAAAGGGCACCCAAGGGTATGGACTAGTGGTCAATGAAGTGGT    2697
E42         ACACTTGCGAAGAAATAGAAAGGGCACCCAAGGGTATGGACTAGTGGTCAATGAAGTGGT    2681
LA2093      ACACTTGCGAAGAAATAGAAAGGGCACCCAAGGGTATGGACTAGTGGTCAATGAAGTGGT    2681
            ************************************************************

HEINZ       TGAGAGCCATGAGATCTCATGTTCAAATCCCAGAAATTTCAAATAATCATTCATCCAAAA    2757
E42         TGAGAGCCATGAGATCTCATGTTCAAATCCCAGTAATTCGAAATAATCATTCATCCAAAA    2741
LA2093      TGAGAGCCATGAGATCTCATGTTCAAATCCCAGTAATTCGAAATAATCATTCATCCAAAA    2741
            ********************************* ****  ********************

HEINZ       GTGCAACATTGCCAGAAGACAATTGAAGTTCCACATTTGATGGACAAGCAAGGCAAGACA    2817
E42         GTGCAACATTGCCAGAAGACAATTGAAGTTCCACATTTGATGGACAAGCAAGGCAAGACA    2801
LA2093      GTGCAACATTGCCAGAAGACAATTGAAGTTCCACATTTGATGGACAAGCAAGGCAAGACA    2801
            ************************************************************

HEINZ       CTAACCAGACTGTATGAACACCCCCATTACTTGCCCAATGCAATTTTCTAAGTAGAAAAT    2877
E42         CTAACCAGACTGTATGAACACCCCCATTACTTGCCCAATGCAATTTTCTAAGTAGAAAAT    2861
LA2093      CTAACCAGACTGTATGAACACCCCCATTACTTGCCCAATGCAATTTTCTAAGTAGAAAAT    2861
            ************************************************************

HEINZ       TGAAGTCAACATAAGAAGTGATTAACCTTGACCAGTAAAACGAATTGTAAGAACCTGATA    2937
E42         TGAAGTCAACATAAGAAGTGATTAACCTTGACCAGTAAAACGAATTGTAAGAACCTGATA    2921
LA2093      TGAAGTCAACATAAGAAGTGATTAACCTTGACCAGTAAAACGAATTGTAAGAACCTGATA    2921
            ************************************************************

HEINZ       CTTACCCACTCGAATCGGCAATTTCTCTAGAAATTTTCATCTTTTTGGTGGTACCCTTGT    2997
E42         CTTACCCACTCGAATCGGCAATTTCTCTAGAAATTTTCATCTTTTTGGTGGTACCCTTGT    2981
LA2093      CTTACCCACTCGAATCGGCAATTTCTCTAGAAATTTTCATCTTTTTGGTGGTACCCTTGT    2981
            ************************************************************

HEINZ       AGAGATCCTCAAGATTACAAGGCAAGTTCTGTTGTACCGGAGCCTCTTTCCTTGGGACTG    3057
E42         AGAGATCCTCAAGATTACAAGGCAAGTTCTGTTGTACCGGAGCCTCTTTCCTTGGGACTG    3041
LA2093      AGAGATCCTCAAGATTACAAGGCAAGTTCTGTTGTACCGGAGCCTCTTTCCTTGGGACTG    3041
            ************************************************************

HEINZ       ATGAATGCATTGGAACACCACCACCTCCTCCTCCTTCACCGAAGGATGCTGCGAATATAT    3117
E42         ATGAATGCATTGGAACACCACCACCTCCTCCTCCTTCACCGAAGGATGCTGCGAATATAT    3101
LA2093      ATGAATGCATTGGAACACCACCACCTCCTCCTCCTTCACCGAAGGATGCTGCGAATATAT    3101
            ************************************************************

HEINZ       CATCAGTGAACATATTACCAAACCTTGAGCCCCTACCCCCTCCAGGTCCATATGGAGTAG    3177
E42         CATCAGTGAACATATTACCAAACCTTGAGCCCCTACCCCCTCCAGGTCCATATGGAGTAG    3161
LA2093      CATCAGTGAACATATTACCAAACCTTGAGCCCCTACCCCCTCCAGGTCCATATGGAGTAG    3161
            ************************************************************

HEINZ       AAAAGCCAAAAAAATTCAGCAAATATGTCATCAGCATTTCTACTGTTGAATCTGAATGAAG    3237
E42         AAAAGCCAAAAAAATTCAGCAAATATGTCATCAGCATTTCTACTGTTGAATCTGAATGAAG    3221
LA2093      AAAAGCCAAAAAAATTCAGCAAATATGTCATCAGCATTTCTACTGTTGAATCTGAATGAAG    3221
            ************************************************************

HEINZ       TAGGGCCATCCCCAGTAGAAAAGTAGGTTGAACCGGCACCAGGACCACCAGCTCCAGGTG    3297
E42         TAGGGCCATCCCCAGTAGAAAAGTAGGTTGAACCGGCACCAGGACCACCAGCTCCAGGTG    3281
LA2093      TAGGGCCATCCCCAGTAGAAAAGTAGGTTGAACCGGCACCAGGACCACCAGCTCCAGGTG    3281
            ************************************************************

HEINZ       GTGGTACCCCGCCCTTGAGCCCTTCTTCACCATACTGGTCATACACAGCTTTTTTCTGGG    3357
E42         GTGGTACCCCGCCCTTGAGCCCTTCTTCACCATACTGGTCATACACAGCTTTTTTCTGGG    3341
LA2093      GTGGTACCCCGCCCTTGAGCCCTTCTTCACCATACTGGTCATACACAGCTTTTTTCTGGG    3341
            ************************************************************

HEINZ       AGTCGCTAAGCACCTAGTAAATATAATGCAGAAACACAATAGTCATCTATTTAACATTCA    3417
E42         AGTCGCTAAGCACCTAGTAAATATAATGCAGAAACACAATAGTCATCTATTTAACATTCA    3401
LA2093      AGTCGCTAAGCACCTAGTAAATATAATGCAGAAACACAATAGTCATCTATTTAACATTCA    3401
            ************************************************************
```

```
HEINZ       AGTCATATAATATCTAATAATAACACCAATAAAAGAGAATCTGTAAAGATAAATCATTGG    3477
E42         AATCATATAATATCTAATAATAACACCAATAAAAGAGAATCTGTAAAGATAAATCATTGG    3461
LA2093      AGTCATATAATATCTAATAATAACACCAATAAAAGAGAATCTGTAAAGATAAATCATTGG    3461
            * **********************************************************

HEINZ       AATCAGATTCGCCAGTTCAGATACTTGCTGCAAATGAAAACATCAATCCCTACTTCACAA    3537
E42         AATCAGATTCGCCAGTTCAGATACTTGCTGCAAATGAAAACATCAATCCCTACTTCACAA    3521
LA2093      AATCAGATTCGCCAGTTCAGATACTTGCTGCAAATGAAAACATCAATCCCTACTTCACAA    3521
            ************************************************************

HEINZ       TAATGTTTCAAGGACAGTGCCAGTTGTAGCTTTGGTTCAAAGATTGCATTTATTCTTAAA    3597
E42         TAATGTTTCAAGGACAGTGCCAGTTGTAGCTTTGGTTCAAAGATTGCATTTATTCTTAAA    3581
LA2093      TAATGTTTCAAGGACAGTGCCAGTTGTAGCTTTGGTTCAAAGATTGCATTTATTCTTAAA    3581
            ************************************************************

HEINZ       CATGCATCGAATACGTAAAAGATTATATAAATTTAGAATCCAAAACTAATAAACTTCATA    3657
E42         CATGCATCGAATACGTAAAAGATTATATAAATTTAGAATCCAAAACTAATAAACTTCATA    3641
LA2093      CATGCATCGAATACGTAAAAGATTATATAAATTTAGAATCCAAAACTAATAAACTTCATA    3641
            ************************************************************

HEINZ       TCCGGGTTCTGCCTCTAGTTTCGAGCTGCGAAAAACTGTTTCCTATCACAAGTGATTTTT    3717
E42         TCCGGGTTCTGCCTCTAGTTTCGAGCTGCGAAAAACTGTTTCCTATCACAAGTGATTTTT    3701
LA2093      TCCGGGTTCTGCCTCTAGTTTCGAGCTGCGAAAAACTGTTTCCTATCACAAGTGATTTTT    3701
            ************************************************************

HEINZ       TTTAACAACAAAAACATAAAACCTATCACTAGTGATCTTTCAATCTTTTCTTCCTACACC    3777
E42         TTTAACAACAAAAACATAAAACCTATCACTAGTGATCTTTCAATCTTTTCTTCCTACACC    3761
LA2093      TTTAACAACAAAAACATAAAACCTATCACTAGTGATCTTTCAATCTTTTCTTCCTACACC    3761
            ************************************************************

HEINZ       TTTCAACTATAAAATTTTGTGATATAAGTGCTTTTCTTCAGTAATAATTGATAGTTACAC    3837
E42         TTTCAACTATAAAATTTTGTGATATAAGTGCTTTTCTTCAGTAATAATTGATAGTTACAC    3821
LA2093      TTTCAACTATAAAATTTTGTGATATAAGTGCTTTTCTTCAGTAATAATTGATAGTTACAC    3821
            ************************************************************

HEINZ       AATTCAAACACATCAAACGTACACCAAATTAAAAATCAATAAACCCTAAATTCAATTCGA    3897
E42         AATTCAAACACATCAAACGTACACCAAATTAAAAATCAATAAACCCTAAATTCAATTCGA    3881
LA2093      AATTCAAACACATCAAACGTACACCAAATTAAAAATCAATAAACCCTAAATTCAATTCGA    3881
            ************************************************************

HEINZ       ACCTCCACAAAACTACCAAATTATGAAAAATGGTTCGGATCTAAAAGAATGAGATCATTT    3957
E42         ACCTCCACAAAACTACCAAATTATGAAAAATGGTTCGGATCTAAAAGAATGAGATCATTT    3941
LA2093      ACCTCCACAAAACTACCAAATTATGAAAAATGGTTCGGATCTAAAAGAATGAGATCATTT    3941
            ************************************************************

HEINZ       GCTTGGTGAATTCAGAATTCAAACAAGTGATTGATTAAGAACAAAGCAATCCAACAATTA    4017
E42         GCTTGGTGAATTCAGAATTCAAACAAGTGATTGATTAAGAACAAAGCAATCCAACAATTA    4001
LA2093      GCTTGGTGAATTCAGAATTCAAACAAGTGATTGATTAAGAACAAAGCAATCCAACAATTA    4001
            ************************************************************

HEINZ       AGTTAACCTATACAAGTTCATATCAACAAAAACATACAAATTAGGTTCCAAGAAAACACA    4077
E42         AGTTAACCTATACAAGTTCATATCAACAAAAACATACAAATTAGGTTCCAAGAAAACACA    4061
LA2093      AGTTAACCTATACAAGTTCATATCAACAAAAACATACAAATTAGGTTCCAAGAAAACACA    4061
            ************************************************************

HEINZ       AAACCGATCATTCTTTTTGCAAGATTATAAACACTAAACATTCTAAACCCTCAAATTAAT    4137
E42         AAACCGATCATTCTTTTTGCAAGATTATAAACACTAAACATTCTAAACCCTCAAATTAAT    4121
LA2093      AAACCGATCATTCTTTTTGCAAGATTATAAACACTAAACATTCTAAACCCTCAAATTAAT    4121
            ************************************************************

HEINZ       CAGCATTTAAACCAAGATTCAGAACCCCATTAGACTATAAACGAATAAAGATTGAAACTT    4197
E42         CAGCATTTAAACCAAGATTCAGAACCCCATTAGACTATAAACGAATAAAGATTGAAACTT    4181
LA2093      CAGCATTTAAACCAAGATTCAGAACCCCATTAGACTATAAACGAATAAAGATTGAAACTT    4181
            ************************************************************

HEINZ       TAAACAAAAAAACCCACAAGGAAAATTGGAAAACATACATCATACGCTTCAGAGATCTGTT    4257
E42         TAAACAAAAAAACCCACAAGGAAAATTGGAAAACATACATCATACGCTTCAGAGATCTGTT    4241
LA2093      TAAACAAAAAAACCCACAAGGAAAATTGGAAAACATACATCATACGCTTCAGAGATCTGTT    4241
            ************************************************************

HEINZ       TGAACTTAGCTTCAGCTTCTTTTTTGTTTTGTGGGTTCTTATCCGGATGCCATTTCATGG    4317
E42         TGAACTTAGCTTCAGCTTCTTTTTTGTTTTGTGGGTTCTTATCCGGATGCCATTTCATGG    4301
```

```
LA2093      TGAACTTAGCTTCAGCTTCTTTTTTGTTTTGTGGGTTCTTATCCGGATGCCATTTCATGG    4301
            ************************************************************

HEINZ       CAAGTTTTCTATAAGCTTTCTTCAAATCATCATCTGTAGCGTTCTTATCAACACCCAATA    4377
E42         CAAGTTTTCTATAAGCTTTCTTCAAATCATCATCTGTAGCGTTCTTATCAACACCCAATA    4361
LA2093      CAAGTTTTCTATAAGCTTTCTTCAAATCATCATCTGTAGCGTTCTTATCAACACCCAATA    4361
            ************************************************************

HEINZ       CTTTGTAATAATCAACCCCCATCTTCTCTACTACTGCTTCGTTTCTCTTCCTTTTCTTCA    4437
E42         CTTTGTAATAATCAACCCCCATCTTCTCTACTACTGCTTCGTTTCTCTTCCTTTTCTTCA    4421
LA2093      CTTTGTAATAATCAACCCCCATCTTCTCTACTACTGCTTCGTTTCTCTTCCTTTTCTTCA    4421
            ************************************************************

HEINZ       GTTTGAGAAATTTGGGTTTTTGAAGAAAAATGATTCTTGAAGATTGCTTCAGTATATGAA    4497
E42         GTTTGAGAAATTTGGGTTTTTGAAGAAAAATGATTCTTGAAGATTGCTTCAGTATATGAA    4481
LA2093      GTTTGAGAAATTTGGGTTTTTGAAGAAAAATGATTCTTGAAGATTGCTTCAGTATATGAA    4481
            ************************************************************

HEINZ       TTTGCGGAGAAAAAAAGAGAAGGAAAAGTGCAGGAGAAGACCGGAAAATGCAAAAACTAA    4557
E42         TTTGCGGAGAAAAAAAGAGAAGG-AAAGTGCAGGAGAAGACCGGAAAATGCAAAAACTAA    4540
LA2093      TTTGCGGAGAAAAAAAGAGAAGG-AAAGTGCAGGAGAAGACCGGAAAATGCAAAAACTAA    4540
            *********************** ************************************

HEINZ       CAGAGTCGAGAAAACTGTGGAAGATTCCAGAACCAGTTCATTATTAGAACTAGGGTTAGT    4617
E42         CAGAGTCGAGAAAACTGTGGAAGATTCCAGAACCAGTTCATTATTAGTACTAGGGTTAGT    4600
LA2093      CAGAGTCGAGAAAACTGTGGAAGATTCCAGAACCAGTTCATTATTAGAACTAGGGTTAGT    4600
            *********************************************** ************

HEINZ       GGACACTATTTTAGCCACGTCATTATAAGTTCAGGTTTTTCTCGAAATCGAATTAAAATT    4677
E42         GGACACTATTTTAGCCACGTCATTATAAGTTCAGGTTTTTCTCGAAATCGAATTAAAATT    4660
LA2093      GGACACTATTTTAGCCACGTCATTATAAGTTCAGGTTTTTCTCGAAATCGAATTAAAATT    4660
            ************************************************************

HEINZ       GATAATTT   4685
E42         GATAATTT   4668
LA2093      GATAATTT   4668
            ********
```

Solyc07g055710

```
HEINZ       AGCTTTAACTCAATATTGTTCAATATCACTAATCCTCTTATCAAAAATATAGATGAGTAC    60
E42         AGCTTTAACTCAATATTGTTCAATATCACTAATCCTCTTATCAAAAATATAGATGAGTAC    60
LA2093      AGCTTTAACTCAATATTGTTCAATATCACTAATCCTCTTATCAAAAATATGGATGAGTAC    60
            ************************************************** *********

HEINZ       CTTACTATAACTCTGATAATTACAAGCAAAGTCTACACTAAAAGTTCCACATAAAGTGGT    120
E42         CTTACTATAACTCTGATAATTACAAGCAAAGTCTACACTAAAAGTTCCACATAAAGTGGT    120
LA2093      CTTACTATAACTCTGATAATTACAAGCAAAGTCTACACTAAAAGTTCCACATAAAGTGGT    120
            ************************************************************

HEINZ       GTACTTTAACATCTAACTTCTAGTGAATGATCTCAGTATTACAACAACAAACCAACAAGT    180
E42         GTACTTTAACATCTAACTTCTAGTGAATGATCTCAGTATTACAACAACAAACCAACAAGT    180
LA2093      GTACTTTAACATCTAACTTCTAGTGAATGATCTCAGTATTACAACAACAAACCAACAAGT    180
            ************************************************************

HEINZ       GCTCTAATGTGACATATCTGATCATCAAAACTAGAAGAAAAAA--AAAAACAACAATAGC    238
E42         GCTCTAATGTGACATATCTGATCATCAAAACTAGAAGAAAAAAACAAAAACAACAATAGC    240
LA2093      GCTCTAATGTGACATATCTGATCATCAAAACTAGAAGAAAACAACAACAACAACAATAGC    240
            ***************************************** *  ** ************

HEINZ       TATAAAATGTCAAATACTGAATCAACTCAGCTTCCTGTTGCTGGACTACTAAGATGTCCC    298
E42         TATAAAATGTCAAATACTGAATCAACTCAGCTTCCTGTTGCTGGACTACTAAGATGTCCC    300
LA2093      TATAAAATGTCAAATACTGAATCAACTCAGCTTCCTGTTGCTGGACTACTAAGATGTCCC    300
            ************************************************************

HEINZ       ATTCTTTCAGCAAGATTTTCTAAATTAACTCCGCGATTCCACCAATATCTATGGCTATCC    358
E42         ATTCTTTCAGCAAGATTTTCTAAATTAACTCCGCGATTCCACCAATATCTATGGCTATCC    360
LA2093      ATTCTTTCAGCAAGATTTTCTAAATTAACTCCGCGATTCCACCAATATCTATGGCTATCC    360
            ************************************************************

HEINZ       CCTAATCTAATATCACGCGTTGATTCGTTTATCCCTTTGTTCTCAACTTGTTGTGGCTCA    418
E42         CCTAATCTAATATCACGCGTTGATTCGTTTATCCCTTTGTTCTCAACTTGTTGTGGCTCA    420
LA2093      CCTAATCTAATATCACGCGTTGATTCGTTTATCCCTTTGTTCTCAACTTGTTGTGGCTCA    420
            ************************************************************

HEINZ       GTGCAACCAGGCGTCTCTGTTAAGAACTGTTGCCAAAATACATCATTACCCGAGTTTGAT    478
E42         GTGCAACCAGGCGTCTCTGTTAAGAACTGTTGCCAAAATACATCATTACCCGAGTTTGAT    480
LA2093      GTGCAACCAGGCGTCTCTGTTAAGAACTGTTGCCAAAATACATCATTACCCGAGTTTGAT    480
            ************************************************************

HEINZ       ACTAATTCAGATGTTTTACTGGTCTTACACTCAATCTGATTATCAGATGGCTTAAGTTGA    538
E42         ACTAATTCAGATGTTTTACTGGTCTTACACTCAATCTGATTATCAGATGGCTTAAGTTGA    540
LA2093      ACTAATTCAGATGTTTTACTGGTCTTACACTCAATCTGATTATCAGATGGCTTAAGTTGA    540
            ************************************************************

HEINZ       CGTTCTAAATTTTCATAAGTTGATGACATGACAGGATTCAATGGCCCTAACTCGGAGGAG    598
E42         CGTTCTAAATTTTCATAAGTTGATGATATGACAGGATTCAATGGCCCTAACTCGGAGGAG    600
LA2093      CGTTCTAAATTTTCATAAGTTGATGACATGACAGGATTCAATGGCCCTAACTCGGAGGAG    600
            ************************** *********************************

HEINZ       GATGGTGAATGATCGATGGGAGAGTTTCGTTTGGCAGAATCATTAGATGATGTATGTGTA    658
E42         GATGGTGAATGATCGATAGGAGAGTTTCGTTTGGCAGAATCATTAGATGATGTATGTGTA    660
LA2093      GATGGTGAATGATCGATGGGAGAGTTTCGTTTGGCAGAATCATTAGATGATGTATGTGTA    660
            ***************** ******************************************

HEINZ       ACAATTGGGGAATGTGTATGCTCGAAATCTTGTGTTTGAACACCATACAGAAACCGCTCC    718
E42         ACAATCGGGGAATGTGTATGCTCGAAATCTTGTGTTTGAACACCATACAGAAACCGCTCC    720
LA2093      ACAATTGGGGAATGTGTATGCTCGAAATCTTGTGTTTGAACACCATACAGAAACCGCTCC    720
            ***** ******************************************************

HEINZ       CAAAAATTGATTGATGAATCCAACTTTTTAACCATTTCCAAGTCGAATTTTGGTGAGTTT    778
E42         CAAAAATTGATTGATGAATCCAACTTTTTAACCATTTCCAAGTCGAATTTTGGTGAGTTT    780
LA2093      CAAAAATTGATTGATGAATCCAACTTTTTAACCATTTCCAAGTCGAATTTTGGTGAGTTT    780
            ************************************************************

HEINZ       TCCTCGTCAATCAAGTAATTTGATATCAACAATCGTCTCTTCCTGCTCGCGCTTTGAGTG    838
E42         TCCTCGTCAATCAAGTAATTTGATATCAACAATCGTCTCTTCCTGCTCGCGCTTTGAGTG    840
LA2093      TCCTCGTCAATCAAGTAATTTGATATCAACAATCGTCTCTTCCTGCTCGCGCTTTGAGTG    840
            ************************************************************
```

```
HEINZ       AAATCAGATGAAAATCCAGGTGTTTGTAGTAATTGAGCTAAAAGAGAAATCAATTTCCCC    898
E42         AAATCAGATGAAAATCCAGGTGTTTGTAGTAATTGAGCTAAAAGAGAAATCAATTTCCCC    900
LA2093      AAATCAGATGAAAATCCAGGTGTTTGTAGTAATTGAGCTAAAAGAGAAATCAATTTCCCC    900
            ************************************************************

HEINZ       TGTCTATGAGCAACGTTCTGTAAACGTTGCTCCATAGACTTAATTCCACTTTCGTACTCC    958
E42         TGTCTATGAGCAACGTTCTGTAAACGTTGCTCCATAGACTTAATTCCACTTTCGTACTCC    960
LA2093      TGTCTATGAGCAACGTTCTGTAAACGTTGCTCCATAGACTTAATTCCACTTTCGTACTCC    960
            ************************************************************

HEINZ       CCATTGAATTTCAATTGATTCTCCGCAGACGACTGAAGAAGACTGTTTTCTCTCTTCAAC    1018
E42         CCATTGAATTTCAATTGATTCTCCGCAGACGACTGAAGAAGACTGTTTTCTCTCTTCAAC    1020
LA2093      CCATTGAATTTCAATTGATTCTCCGCAGACGACTGAAGAAGACTGTTTTCTCTCTTCAAC    1020
            ************************************************************

HEINZ       CTCTCGATTTCATCTTCATACTCCTGTCTCTCAGAATCCGTCAATGGAGCTACAGATTGC    1078
E42         CTCTCGATTTCATCTTCATACTCCTGTCTCTCAGAATCCGTCAATGGAGCTACAGATTGC    1080
LA2093      CTCTCGATTTCATCTTCATACTCCTGTCTCTCAGAATCCGTCAATGGAGCTACAGATTGC    1080
            ************************************************************

HEINZ       CCTGTTCCTGCTGCAGCAGAGTGGCTATGGATCGGCTTTCGTCTATAAATATTCTTCAAC    1138
E42         CCTGTTCCTGCTGCAGCAGAGTGGCTATGGATCGGCTTTCGTCTATAAATATTCTTCAAC    1140
LA2093      CCTGTTCCTGCTGCAGCAGAGTGGCTATGGATCGGCTTTCGTCTATAAATATTCTTCAAC    1140
            ************************************************************

HEINZ       AAATGTCTACGTCCTCTTAAAAAATCCTCGTTCGCAAACTCCCATTGTTCAGGATCAACC    1198
E42         AAATGTCTACGTCCTCTTAAAAAATCCTCGTTCGCAAACTCCCATTGTTCAGGATCAACC    1200
LA2093      AAATGTCTATGTCCTCTTAAAAAATCCTCGTTCGCAAACTCCCATTGTTCAGGATCAACC    1200
            ********* **************************************************

HEINZ       TTTCTAAACCCCTAATAGAAATAAATGAATATCGTCAAATAACAAAACACAATTGGTGAT    1258
E42         TTTCTAAACCCCTAATAGAAATAAATGAATATCGTCAAATAACAAAACACAATTGGTGAT    1260
LA2093      TTTCTAAACCCCTAATAGAAATAAATGAATATCGTCAAATAACAAAACACAATTGGTGAT    1260
            ************************************************************

HEINZ       GATTCGATAGAGATATTTTTACGAAATAATTTTACTTACATAAGTATTAAGTTGTCTGAT    1318
E42         GATTCAATAGAGATATTTTTACGAAATAATTTTACTTACATAAGTATTAAGTTGTCTGAT    1320
LA2093      GATTCGATAGAGATATTTTTACGAAATAATTTTACTTACATAAGTATTAAGTTGTCTGAT    1320
            ***** ******************************************************

HEINZ       AAAACTTGAGAAATTGTTATGCTTAAAGTATTTCGGAAGCAAATCTCTAGCGAATTCAGG    1378
E42         AAAACTTGAGAAATTGTTATGCTTAAAGTATTTCGGAAGCAAATCTCTAGCGAATTCAGG    1380
LA2093      AAAACTTGAGAAATTGTTATGCTTAAAGTATTTCGGAAGCAAATCTCTAGCGAATTCAGG    1380
            ************************************************************

HEINZ       TGGATTCCAAACAACGAAGCTACGTCCGTTATGGCTCCATGAAACAACTGGATTAGTATA    1438
E42         TGGATTCCAAACAACGAAGCTACGTCCGTTATGGCTCCATGAAACAACTGGATTAGTATA    1440
LA2093      TGGATTCCAAACAACGAAGCTACGTCCGTTATGGCTCCATGAAACAACTGGATTAGTATA    1440
            ************************************************************

HEINZ       CGAATCATCAACCAGTTCATAAGTTTTCAACAAAAAAGGCGCCGGAGAAGAAGAACTTGA    1498
E42         CGAATCATCAACCAGTTCATAAGTTTTCAACAAAAAAGGCGCCGGAGAAGAAGAACTTGA    1500
LA2093      CGAATCATCAACCAGTTCATAAGTTTTCAACAAAAAAGGCGCCGGAGAAGAAGAACTTGA    1500
            ************************************************************

HEINZ       TCCTCCATTACAGTTATCCATGACTATGCACACTCATATACTACTTTTAACGATTCTAAT    1558
E42         TCCTCCATTACAGTTATCCATGACTACGCACACTCATATACTATTTTTAACGATTCTAAT    1560
LA2093      TCCTCCATTACAGTTATCCATGACTATGCACACTCATATACTATTTTTAACGATTCTAAT    1560
            ************************** **************** ****************

HEINZ       GTGAATTGCGAAGATTATTATACTTTGAGTTTTCAATGAGAAAGGGGAAGAAAAATGATC    1618
E42         GTGAATTGCGAAGATTATTATACTTTGAGTTTTCAATGAGAAAGGGGAAGAAAAATGATC    1620
LA2093      GTGAATTGCGAAGATTATTATACTTTGAGTTTTCAATGAGAAAGGGGAAGAAAAATGATC    1620
            ************************************************************

HEINZ       AG 1620
E42         AG 1622
LA2093      AG 1622
            **
```

Solyc07g055720

```
E42        GCTGCAAGTGCCAATAATATCTAATCTGATTGCCGATATTTGTGGAATGAAAGATCTTTT   60
HEINZ      GCTGCAAGTGCCAATAATATCTAATCTGATTGCCGATATTTGTGGAATGAAAGATCTTTT   60
LA2093     GCTGCAAGTGCCAATAATATCTAATCTGATTGCCGATATTTGTGGAATGAAAGATCTTTT   60
           ************************************************************

E42        CCACCAAAAAAGTTAACTTCACAATATTATCATCATTCATATCACCATATCCAAAATTCC   120
HEINZ      CCACCAAAAAAGTTAACTTCACAATATTATCATCATTCATATCACCATATCCAAAATTCC   120
LA2093     CCACCAAAAAAGTTAACTTCACAATATTATCATCATTCATATCACCATATCCAAAATTCC   120
           ************************************************************

E42        TTTATCTTAAACAACATTTGTATCTCAAAGCAAGTAGAGGAACACTTTCTGTATTATATT   180
HEINZ      TTTATCTTAAACAACATTTGTATCTCAAAGCAAGTAGAGGAACACTTTCTGTATTATATT   180
LA2093     TTTATCTTAAACAACATTTGTATCTCAAAGCAAGTAGAGGAACACTTTCTGTATTATATT   180
           ************************************************************

E42        CACTCTGTTTTGGAGCAATGGAGAGTCAAATTGTTCGACGAAGAGTTAACATGATTACTG   240
HEINZ      CACTCTGTTTTGGAGCAATGGAGAGTCAAATTGTTCGACGAAGAGTTAACATGATTACTG   240
LA2093     CACTCTGTTTTGGAGCAATGGAGAGTCAAATTGTTCGACGAAGAGTTAACATGATTACTG   240
           ************************************************************

E42        CTCATTTAACTGCACATGATGATATTTCCGCCTCCGCTACTCATCTCTTTCCTATGGTAA   300
HEINZ      CTCATTTAACTGCACATGATGATATTTCCGCCTCCGCTACTCATCTCTTTCCTATGGTAA   300
LA2093     CTCATTTAACTGCACATGATGATATTTCCGCCTCCGCTACTCATCTCTTTCCTATGGTAA   300
           ************************************************************

E42        TCTCTTTCTCTGTCTCTACTCATAGTAATGTTAGCAGGGACGGAGTTACAGGTTCGATAG   360
HEINZ      TCTCTTTCTCTGTCTCTACTCATAGTAATGTTAGCAGGGACGGAGTTACAGGTTCGATAG   360
LA2093     TCTCTTTCTCTGTCTCTACTCATAGTAATGTTAGCAGGGACGGAGTTACAGATTCGATAG   360
           *************************************************** ********

E42        AACTAGTAGCTTTCGCCAGCATTTCGTATTTATGTTTAAAAAATTATGAATAGTATAATT   420
HEINZ      AACTAGTAGCTTTCGCCAGCATTTCGTATTTATGTTTAAAAAATTATGAATAGTATAATT   420
LA2093     AACTAGTAGCTTTCGCCAGCATTTCGTATTTATGTTTAAAAAATTATGAATAGTATAATT   420
           ************************************************************

E42        TATTTGAAGTAAAGTCAAAGGTGTCTTTACGAGAATCCAGAACCTCTGAATGGCAGGACT   480
HEINZ      TATTTGAAGTAAAGTCAAAGGTGTCTTTACGAGAATCCAGAACCTCTGAATGGTAGGACT   480
LA2093     TATTTGAAGTAAAGTCAAAGGTGTA---------ATCCAGAACCTCTGAATGGTAGGACT   471
           ************************          ******************* ******

E42        TGTCACGAGCTAATTCGAAAATAAAGTAATCTACTCATAGCCCTTTGGTTAAAGTCATAC   540
HEINZ      TGTCACGAGCTAATTTGAAAATAAAGTAATCTACTCATAGCCCTTTGGTTAAAGTCATAC   540
LA2093     TGTCACGAGCTAATTCGAAAATAAAGTAATCTACTCATAGCCCTTTGGTTAAAGTCATAC   531
           *************** ********************************************

E42        AACTATTCATTTCCCCTGTTTCTTTTAACTATATATCTATTGATGTGTTTCTATTTGATT   600
HEINZ      CACTATTCATTTCCCCTGTTTCTTTTAACTATATATCTATTGATGTGCTTCTATTTGATT   600
LA2093     CACTATTCATTTCCCCTGTTTCTTTTAACTATATATCTATTGATGTGCTTCTATTTGATT   591
            ********************************************** ************

E42        TTTCACTTTGTTTACTGTATAGAGCTGTAGCAGTAGCTTGAATTCTGCTATTCCGAGGAG   660
HEINZ      TTTCACTTTGTTTACTGTATAGAGCTGTAGCAGTAGCTTGAATTCTGCTATTCCGAGGAG   660
LA2093     TTTCACTTTGTTTACTGTATAGAGCTGTAGCAGTAGCTTGAATTCTGCTATTCCGAGGAG   651
           ************************************************************

E42        GTACGATAACAGAATGAACTATGCAAGACAAAGTTCCAGTTCTCAAGCTTGTTTCATGAG   720
HEINZ      GTACGATAACAGAATGAACTATGCAAGACAAAGTTCCAGTTCTCAAGCTTGTTTCATGAG   720
LA2093     GTACGATAACAGAATGAACTATGCAAGACAAAGTTCCAGTTCTCAAGCTTGTTTCATGAG   711
           ************************************************************

E42        GACAAGTGAACAGGTCTGGGAATTATTACTAATATAATTGACATTACTTTAGTTTCAATG   780
HEINZ      GACAAGTGAACAGGTCTGGGAATTATTACTAATATAATTGACATTACTTTAGTTTCAATG   780
LA2093     GACAAGTGAACAGGTCTGGGAATTATTACTAATATAATTGACATTACTTTAGTTTCAATG   771
           ************************************************************

E42        AGTTTGTAATATTCGTTCTGCAGGTGTTAGCTTTTTTCATTTTTTTTTCTTGCTTTGGGA   840
HEINZ      AGTTTGTAATATTCGTTCTGCAGGTGTTAGCTTTTTTCATTTTTTTTTCTTGCTTTGGGA   840
LA2093     AGTTTGTAATATTCGTCCTGCAGGTGTTAGCTTTTTTCATTTTTTTTTCTTGCTTTGGGA   831
           **************** *******************************************
```

```
E42       GCAGGGAAGCTGTACTGAATCCACTGCGGCTTTCAAGGCTAGTGATTATGCAAAGAAGAG   900
HEINZ     GCAGGGAAGCTGTACTGAATCCACTGCGGCTTTCAAGGCTAGTGATTATGCAAAGAAGAG   900
LA2093    GCAGGGAAGCTGTACTGAATCCACTGCGGCTTTCAAGGCTAGTGATTATGCAAAGAAGAG   891
          ************************************************************

E42       TTCTCGCGCCTTTGAAGGACCAATGTTTTCTAGACCTGCAAATAATTGCAAACACAATGG   960
HEINZ     TTCTCGCGCCTTTGAAGGACCAATGTTTTCTAGACCTGCAAATAATTGCAAACACAATGG   960
LA2093    TTCTCGCGCCTTTGAAGGACCAATGTTTTCTAGACCTGCAAATAATTGCAAACACAATGG   951
          ************************************************************

E42       AACTGTTGAAGAAGCTCCTAAATTTGCAAGGCCTCGTTTCCAATTGAAAGAAAGAAGAAA   1020
HEINZ     AACTGTTGAAGAAGCTCCTAAATTTGCTAGGCCTCGTTTCCAATTGAAAGAAAGAAGAAA   1020
LA2093    AACTGTTGAAGAAGCTCCTAAATTTGCTAGGCCTCGTTTCCAATTGAAAGAAAGAAGAAA   1011
          *************************** ********************************

E42       TGAACTTGAATCTAATGGTAAACAAACCTATGCTTTACTTTTGATTGTAATATCGCGTAA   1080
HEINZ     TGAACTTGAATCTAATGGTAAACAAACCTATGCTTTACTTTTGATTGTTATATCGCGTAA   1080
LA2093    TGAACTTGAATCTAATGGTAAACAAACCTATGCTTTACTTTTGATTGTTATATCGCGTAA   1071
          ************************************************ ***********

E42       TTGAGAGGTACGTCCTCTTTGGAATGTTGAAACATTTCCTTTTGTTTTTGCTAATGCAGG   1140
HEINZ     TTGAGAGGTACGTCCTCTTTGGAATGTTGAAACATTTCCTTTTGTTTTTGCTAATGCAGG   1140
LA2093    TTGAGAGGTACGTCCTCTTTGGAATGTTGAAACATTTCCTTTTGTTTTTGCTAATGCAGG   1131
          ************************************************************

E42       GAGTGAATGGTCTCCTAAGATGGATGTTGCAGAATCTGGAAGCATGTACGTCGTATCTAT   1200
HEINZ     GAGTGAATGGTCTCCTAAGATGGATGTTGCAGAATCTGGAAGCATGTACGTCGTATCTAT   1200
LA2093    GAGTGAATGGTCTCCTAAGATGGATGTTGCAGAATCTGGAAGCATGTACGTCGTATCTAT   1191
          ************************************************************

E42       AGAACTTCCCGGTGTCAATATAAATGATATAAAGGTCGAAGTCAGCCACAAAAGGTAAAT   1260
HEINZ     AGAACTTCCCGGTGTCAATATAAATGATATAAAGGTCGAAGTCAGCCACAAAAGGTAAAT   1260
LA2093    AGAACTTCCCGGTGTCAATATAAATGATATAAAGGTCGAAGTCAGCCACAAAAGGTAAAT   1251
          ************************************************************

E42       AAATGACAACTATCCATTAGTCTTTCTTTGATTTCTCGTATAGTCACTATTATCTCAGAA   1320
HEINZ     AAATGACAACTATCCATTAGTCTTTCTTTGATTTCTCGTATAGTCACTATTATCTCAGAA   1320
LA2093    AAATGACAACTATCCATTAGTCTTTCTTTGATTTCTCGTATAGTCACTATTATCTCAGAA   1311
          ************************************************************

E42       TGTCAATTTCTTTGTGAGATCAGCATGTGTTATAAAGTTCCATCTTCTGCAAAGATGAAT   1380
HEINZ     TGTCAATTTCTTTGTGAGATCAGCATGTGTTATAAAGTTCCATCTTCTGCAAAGATGAAT   1380
LA2093    TGTCAATTTCTTTGTGAGATCAGCATGTGTTATAAAGTTCCATCTTCTGCAAAGATGAAT   1371
          ************************************************************

E42       TCAATTTGCGTTGATCTAATAGACGCGTGTTATGTTCCTTGACAGCTTATTAGTTTCTGG   1440
HEINZ     TCAATTTGCGTTGATCTAATAGACGCGTGTTATGTTCCTTGACAGCTTAAAGTTTCTGG   1440
LA2093    TCAATTTGCGTTGATCTAATAGACGCGTGTTATGTTCCTTGACAGCTTAAAGTTTCTGG   1431
          ************************************************* **********

E42       AAATCGTTCCACTCAGTGTAAAGTGGCATCATACTTGAATGGCTTGGTATCAGCCTATCA   1500
HEINZ     AAATCGTTCCACTCAGTGTAAAGTGGCATCATACTTGAATGGCTTGGTATCAGCCTATCA   1500
LA2093    AAATCGTTCCACTCAGTGTAAAGTGGCATCATACTTGAATGGCTTGGTATCAGCCTATCA   1491
          ************************************************************

E42       CAAAAAGGAGATTGTTCAGGGACCATACCGTGTTTTCTGGCCTCTTCCAAGTAATGCTAA   1560
HEINZ     TAAAAAGGAGATTGTTCAGGGACCATACCGTGTTTTCTGGCCTCTTCCAAGTAATGCTAA   1560
LA2093    CAAAAAGGAGATTGTTCAGGGACCATACCGTGTTTTCTGGCCTCTTCCAAGTAATGCTAA   1551
           ***********************************************************

E42       TAAAAATCGCGTCTCAGCTGAGTTTGTGTAAGTTATCAATATTTGTGATTGTTTACCTAT   1620
HEINZ     TAAAAATCGCGTCTCAGCTGAGTTTGTGTAAGTTATCAATATTTGTGATTGTTTACCTAT   1620
LA2093    TAAAAATCGCGTCTCAGCTGAGTTTGTGTAAGTTATCAATATTTGTGATTGTTTACCTAT   1611
          ************************************************************

E42       ATATCGCCATGCTTAATGTTTTTAGTCGAGAAAAAACACTTCATTACTTATAGACTTGTG   1680
HEINZ     ATATCGCCATGCTTAATGTTTTTAGTCGAGAAAAAACACTTCGTTACATATAGACTTGTG   1680
LA2093    ATATCGCCATGCTTAATGTTTTTAGTCGAGAAAAAACACTTCGTTACATATAGACTTGTG   1671
          ****************************************** **** ************

E42       AAATTTCATGACTCTAAAACAATGCAGGGACGGACTTTTACAGATTACAATCCCGAAACT   1740
```

```
HEINZ        AAATTTCATGACTCTAAAACAATGCAGGGACGGACTTTTACAGATTACAATCCCGAAACT    1740
LA2093       AAATTTCATGACTCTAAAACAATGCAGGGACGGACTTTTACAGATTACAATCCCGAAACT    1731
             ************************************************************

E42          TTGAGAAACATGGCAAAGACAGATATATACGTATAGAATATATACTCGAAGCAGGATAAG    1800
HEINZ        TTGAGAAACATGGCAAAGACAGATATATACGTATAGAATATATACTCGAAGCAGGATAAG    1800
LA2093       TTGAGAAACATGGCAAAGACAGATATATACGTATAGAATATATACTCGAAGCAGGATAAG    1791
             ************************************************************

E42          CAACAGAAGTTACCATACAACATGAACCACTTATAAGTAATCGCACGCTATTTACTGAAC    1860
HEINZ        CAACAGAAGTTACCATACAACATGAACCACTTATAAGTAATCGCACGCTATTTACTGAAC    1860
LA2093       CAACAGAAGTTACCATACAACATGAACCACTTATAAGTAATCGCACGCTATTTACTGAAC    1851
             ************************************************************

E42          CAGTGTAAATTGTCATTTTTTATGAGCTATACTTGTAATGCTACATTAGTAGGTATATAC    1920
HEINZ        CAGTGTAAATTGTCATTTTTTATGAGCTATACTTGTAATGCTACATTAGTAGGTATATAC    1920
LA2093       CAGTGTAAATTGTCATTTTTTATGAGCTATACTTGTAATGCTACATTAGTAGGTATATAC    1911
             ************************************************************

E42          ATAAATGTTCATGTCATGTGTGTTTAAGTTATATATATATGGGGAATAGGTTCAAATTTA    1980
HEINZ        ATAAATGTTCATGTCATGTGTGTTTAAGTTATATATATATGGGGAATAGGTTCAAATTTA    1980
LA2093       ATAAATGTTCATGTCATGTGTGTTTAAGTTATATATATATGGGGAATAGGTTCAAATTTA    1971
             ************************************************************

E42          TATTTGATAAATTTGAACTATGTGAAATGATCAACCTTCTTCG      2023
HEINZ        TATTTGATAAATTTGAACTATGTGAAATGATCAACCTTCTTCG      2023
LA2093       TATTTGATAAATTTGAACTATGTGAAATGATCAACCTTCTTCG      2014
             *******************************************
```

Solyc07g065970

```
E42         CACCAACACTGATCCGTTTCCCACCTTCGGGTCGGGTAAAACTCTGACCCATTCCTACGA    60
HEINZ       CACCAACACTGATCCGTTTCCCACCTTCGGGTCGGGTAAAACTCTGACCCATTCCTACGA    60
LA2093      CACCAACACTGATCCGTTTCCCACCTTCGGGTCGGGTAAAACTCTGACCCATTCCTACGA    60
            ************************************************************

E42         ATTCCATCGCCGGAGCTCCCAAAACCTCGTCGGCCGGAGCTCATGTTTAGCTTGAGATCA    120
HEINZ       ATTCCATCGCCGGAGCTCCCAAAACCTCGTCGGCCGGAGCTCATGTTTAGCTTGAGATCA    120
LA2093      ATTCCATCGCCGGAGCTCCCAAAACCTCGTCGGCCGGAGCTCATGTTTAGCTTGAGATCA    120
            ************************************************************

E42         TAGAGATCTCTAGAAACAGGATCTGATAACGTAGCATAAGCTTCGTGAATCTCAATGAAA    180
HEINZ       TAGAGATCTCTAGAAACAGGATCTGATAACGTAGCATAAGCTTCGTGAATCTCAATGAAA    180
LA2093      TAGAGATCTCTAGAAACAGGATCTGATAACGTAGCATAAGCTTCGTGAATCTCAATGAAA    180
            ************************************************************

E42         TGGCGTCCGTCGGAAGATTCCTCCGACAGAGACGCAGCATCGGGATGGTATAGCTTAGCT    240
HEINZ       TGCCGTCCGTCGGAAGATTCCTCCGACAGAGACGCAGCATCGGGATGGTATAGCTTAGCT    240
LA2093      TGCCGTCCGTCGGAAGATTCCTCCGACAGAGACGCAGCATCGGGATGGTATAGCTTAGCT    240
            ** *********************************************************

E42         AAATTCCGGTAAGCTGTTTTTATCTCTTTGGGTGACGCATTTGATTTTACCCTTAGTACA    300
HEINZ       AAATTCCGGTAAGCTGTTTTTATCTCTTTGGGTGACGCATTTGATTTTACCCTTAGTACA    300
LA2093      AAATTCCGGTAAGCTGTTTTTATCTCTTTGGGTGACGCATTTGATTTTACCCTTAGTACA    300
            ************************************************************

E42         TCGTAGAGACTCGCCGGTGTTCTCCTTTGCAATTCCGGCGGTGCTTCCACAACGGCGGCG    360
HEINZ       TCGTAGAGACTCGCCGGTGTTCTCCTTTGCAATTCCGGCGGTGCTTCCGCAACGGCGGCG    360
LA2093      TCGTAGAGACTCGCCGGTGTTCTCCTTTGCAATTCCGGCGGTGCTTCCGCAACGGCGGCG    360
            ************************************************ ***********

E42         GCATATACCGCTGCTGTTCGATTATTCCTTCGGGGAAATGTGACTCCGGATCTGAAACCG    420
HEINZ       GCATATACCGCTGCTGTTCGATTATTCCTTCGGGGAAATGTGACTCCGGATCTGAAACCG    420
LA2093      GCATATACCGCTGCTGTTCGATTATTCCTTCGGGGAAATGTGACTCCGGATCTGAAACCG    420
            ************************************************************

E42         GCTGTGGAAGAAGGAAGGTTGTGTAGAGAAAAACGGAAAGTATTTCCGGCCGGTAGTGTT    480
HEINZ       GCTGTGGAAGAAGGAAGGTTGTGTAGAGAAAAACGGAAAGTATTTCCGGCCGGTAGTGTT    480
LA2093      GCTGTGGAAGAAGGAAGGTTGTGTAGAGAAAAACGGAAAGTATTTCCGGCCGGTAGTGTT    480
            ************************************************************

E42         AGGGATTGAACCAT    494
HEINZ       AGGGATTGAACCAT    494
LA2093      AGGGATTGAACCAT    494
            **************
```

Solyc07g066290

```
LA2093      ATCCACTAACTTCACATAAAGGTATTAGAAGGAAAGAATGTAGATCTCGTGTTTAAATAT    60
E42         ATCCACTAACTTCACATAAAGGTATTAGAAGGAAAGAATGTAGATCTCGTGTTTAAATAT    60
HEINZ       ATCCACTAACTTCACATAAAGGTATTAGAAGGAAAGAATGTAGATCTCGTGTTTAAATAT    60
            ************************************************************

LA2093      GTGGAATGAGCAGGATCAACAGTATGAATTGTCCTACAACTGCATTGCACAGAAGAGTAC    120
E42         GTGGAATGAGCAGGATCAACAGTATGAATTGTCCTACAACTGCATTGCACAGAAGAGTAC    120
HEINZ       GTGGAATGAGCAGGATCAACAGTATGAATTGTCCTACAACTGCATTGCACAGAAGAGTAC    120
            ************************************************************

LA2093      AATTT-TTTTCTCTGAAATTTATTACACCTCAAGTAGTCCAAGTCCAGGCAGGATCATCA    179
E42         AATTT-TTTTCTCTGAAATTTATTACACCTCAAGTAGTCCAAGTCCAGGCAGGATCATCA    179
HEINZ       AATTTTTTTTCTCTGAAATTTATTACACCTCAAGTAGTCCAAGTCCAGGCAGGATCATCA    180
            ***** ******************************************************

LA2093      GATGTACAAAGATTTGCTCAGTTTTGTTCCTCCTAATCAATTCAAAGGATCTAGCTCAAA    239
E42         GATGTACAAAGATTTGCTCAGTTTTGTTCCTCCTAATCAATTCAAAGGATCTAACTCAAA    239
HEINZ       GATGTACAAAGATTTGCTCAGTTTTGTTCCTCCTAATCAATTCAAAGGATCTAACTCAAA    240
            ***************************************************** ******

LA2093      ACTTGGCGTTGCAATAGATAAATGCTGCTGGATAATTAGGATACAACATATCTTAGTGTA    299
E42         ACTTGGCGTTGCAATAGATAAATGCTGCTGGATAATTAGGATACAACATATCTTAGTGTA    299
HEINZ       ACTTGGCGTTGCAATAGATAAATGCTGCTGGATAATTAGGATACAACATATCTTAGTGTA    300
            ************************************************************

LA2093      ATCCCACATAGTCTGGAAAGGGTGGTGTGTAAGCAAGCCGGATAATTAGGATGATATATA    359
E42         ATCCCACATAGTCTGGAAAGGGTGG-TTGTAAGCAAGCCGGATAATTAGGATGATATATA    358
HEINZ       ATCCCACATAGTCTGGAAAGGGTGG-TTGTAAGCAAGCCGGATAATTAGGATGATATATA    359
            *************************  *********************************

LA2093      CAAATGGATCAGCTAACCAGCACATTTATACTTGTTTAGCCAAACCAACAGATGAGCTCT    419
E42         CAAATGGATCAGCTAACCAGCACATTTATACTTGTTTAGCCAAACCAACAGATGAGCTCT    418
HEINZ       CAAATGGATCAGCTAACCAGCACATTTATACTTGTTTAGCCAAACCAACAGATGAGCTCT    419
            ************************************************************

LA2093      CCCCTGATGAGAAGTTTGGTCATCTCCATGTTGATGCATATAAAACCTTAAGATATCACA    479
E42         CCCCTGATGAGAAGTTTGGTCATCTCCATGTTGATGCATATGAAACCTTAAGATATCACA    478
HEINZ       CCCCTGATGAGAAGTTTGGTCATCTCCATGTTGATGCATATGAAACCTTAAGATATCACA    479
            ***************************************** ******************

LA2093      TCATGTCTTTTTATCCTCGTTTTTCATTTTGTCCTGATTTTAGGGGGCAGTGGATGTTGA    539
E42         TCATGTCTTTTTATCCTCGTTTTTCATTTTGTCCTGATTTTAGGGGGCAGTGGATGTTGA    538
HEINZ       TCATGTCTTTTTATCCTCGTTTTTCATTTTGTCCTGATTTTAGGGGGCAGTGGATGTTGA    539
            ************************************************************

LA2093      ATCATCACAAAAAATGGCATCTGAGCATCGTAACCGATTATGATGCATGTAACTCATGAAG    599
E42         ATCATCACAAAAAATGGCATCTGAGCATCGTAACCGATTATGATGCATGTAACTCATGAAG    598
HEINZ       ATCATCACAAAAAATGGCATCTGAGCATCGTAACCGATTATGATGCATGTAACTCATGAAG    599
            ************************************************************

LA2093      ACCTTCTCCATTGCTTCTTTCCTTTCATCTGATTCAACTTGAAAGGACTCCAAGTTTTTC    659
E42         ACCTTCTCCATTGCTTCTTTCCTTTCATCTGATTCAACTTGAAAGGACTCCAAGTTTTTC    658
HEINZ       ACCTTCTCCATTGCTTCTTTCCTTTCATCTGATTCAACTTGAAAGGACTCCAAGTTTTTC    659
            ************************************************************

LA2093      TCCGCTTTGGCGTCTCACAAAAAAAGTCCAGATGCTAGAGCTTGCTGGAGCCACAGCTCGA    719
E42         TCCGCTTTGGCGTCTCACAAAAAAAGTCCAGATGCTAGAGCTTGCTGGAGCCACAGCTCGA    718
HEINZ       TCCGCTTTGGCGTCTCACAAAAAAAGTCCAGATGCTAGAGCTTGCTGGAGCCACAGCTCGA    719
            ************************************************************

LA2093      ATTCTTCATCTTCATCTGTCATCTCAGCATCAAAATCCCAGGGGTACCTACTTGAATTGG    779
E42         ATTCTTCATCTTCATCTGTCATCTCAGCATCAAAATCCCAGGGGTACCTACTTGAATTGG    778
HEINZ       ATTCTTCATCTTCATCTGTCATCTCAGCATCAAAATCCCAGGGGTACCTACTTGAATTGG    779
            ************************************************************

LA2093      ATCTCTGGGATGATTTCTCTAATCCAACCATGTTCACATGGAAACTCGGTCTATGTGTAT    839
E42         ATCTCTGGGATGATTTCTCTAATCCAACCATGTTCACATGGAAACTCGGTCTATGTGTAT    838
HEINZ       ATCTCTGGGATGATTTCTCTAATCCAACCATGTTCACATGGAAACTCGGTCTATGTGTAT    839
            ************************************************************
```

```
LA2093      TTGGTCTACAAGCCATGCCCTGCACAGAGGAGATTCGTAATACAAAAACTAGCAGCAAAG    899
E42         TTGGTCTACAAGCCATGCCCTGCACAGAGGAGATTCGTAATACAAAAACTAGCAGCAAAG    898
HEINZ       TTGGTCTACAAGCCATGCCCTGCACAGAGGAGATTCGTAATACAAAAACTAGCAGCAAAG    899
            ************************************************************

LA2093      GCCATATTTCCATCTTTTTCATAATGACAGAATACTGACCTGACATATGGCCCATTCCGA    959
E42         GCCATATTTCCATCTTTTTCATAATGACAGAATACTGACCTGACATATGGCCCATTCCGA    958
HEINZ       GCCATATTTCCATCTTTTTCATAATGACAGAATACTGACCTGACATATGGCCCATTCCGA    959
            ************************************************************

LA2093      CACATCAAATATCTTGCCTTCAGCACAAACGAAAGCACGAGGTATCTCCGCCTGAATGAC    1019
E42         CACATCAAATATCTTGCCTTCAGCACAAACAAAAGCACGAGGTATCTCCGCCTGAATGAC    1018
HEINZ       CACATCAAATATCTTGCCTTCAGCACAAACGAAAGCACGAGGTATCTCCGCCTGAATGAC    1019
            ****************************** *****************************

LA2093      AAGTATATTTACAAACATTATGAAGAATGAGCATCTTATGGACGCACATCAAGAGAAAGC    1079
E42         AAGTATATTTACAAACATTATGAAGAATGAGCATCTTATGGACGCACATCAAGAGAAAGC    1078
HEINZ       AAGTATATTTACAAACATTATGAAGAATGAGCATCTTATGGACGCACATCAAGAGAAAGC    1079
            ************************************************************

LA2093      ATGATCAACTAAACTACAGCTGAAAGGAAGGGAAACCACTGTTCATGTGCAGCTAGATGC    1139
E42         ATGATCAACTAAACTACAGCTGAAAGGAAGGGAAACCACTGTTCATGTGCAGCTAGATGC    1138
HEINZ       ATGATCAACTAAACTACAGCTGAAAGGAAGGGAAACCACTGTTCATGTGCAGCTAGATGC    1139
            ************************************************************

LA2093      TGATTTCAACTCTTTGAAGTTGACATTTGACTAGTAGTGCCACACTAATTGGCCAGAGTA    1199
E42         TGATTTCAACTCTTTGAAGTTGACATTTGACTAGTAGTGCCACACTAATTGGCCAGAGTA    1198
HEINZ       TGATTTCAACTCTTTGAAGTTGACATTTGACTAGTAGTGCCACACTAATTGGCCAGAGTA    1199
            ************************************************************

LA2093      TTAATATTTGATTGTACCCTCTAAGCACATAAATACAGTAACTCATACAGTTAAAAAGTG    1259
E42         TTAATATTTGATTGTACCCTCTAAGCACATAAATACAGTAACTCATACAGTTAAAAAGTG    1258
HEINZ       TTAATATTTGATTGTACCCTCTAAGCACATAAATACAGTAACTCATACAGTTAAAAAGTG    1259
            ************************************************************

LA2093      GAGAACCACCCTATATACCATTAACTTGATCACATAAACCATGTTAAGATTTGACCAAAC    1319
E42         GAGAACCACCCTATATACCATTAACTTGATCACATAAACCATGTTAAGATTTGACCAAAC    1318
HEINZ       GAGAACCACCCTATATACCATTAACTTGATCACATAAACCATGTTAAGATTTGACCAAAC    1319
            ************************************************************

LA2093      CAGAGGAAAATCCTAGAGACAGATGATGGACTTGCTTCATTGCACCATCAACAATTTGAA    1379
E42         CAGAGGAAAATCCTAGAGACAGATGATGGACTTGCTTCATTGCACCATCAACAATTTGAA    1378
HEINZ       CAGAGGAAAATCCTAGAGACAGATGATGGACTTGCTTCATTGCACCATCAACAATTTGAA    1379
            ************************************************************

LA2093      GCTTGGTAATGCAGCCAAGGTAAACCATTAAGTACAAGAGTCCAAATATTTTGAATTGAT    1439
E42         GCTTGGTAATGCAGCCAAGGTAAACCATTAAGTACAAGAGTCCAAATATTTTGAATTGAT    1438
HEINZ       GCTTGGTAATGCAGCCAAGGTAAACCATTAAGTACAAGAGTCCAAATATTTTGAATTGAT    1439
            ************************************************************

LA2093      CTAAGTATTTATATTTTTTGATAAATTGCTTCCTACAATATCTATTGAAAATCGAACTTG    1499
E42         CTAAGTATTTATATTTTTTGATAAATTGCTTCCTACAATATCTATTGAAAATCGAACTTG    1498
HEINZ       CTAAGTATTTATATTTTTTGATAAATTGCTTCCTACAATATCTATTGAAAATCGAACTTG    1499
            ************************************************************

LA2093      ACAGTAATGATCTAGTATAACGTGCAGCTTTTTTAGTGACTATAATATATGTTTGGGGCC    1559
E42         ACAGTAATGATCTAGTATAACGTGCAGCTTTTTTTGTGACTATAATATATGTTTGGGGCC    1558
HEINZ       ACAGTAATGATCTAGTATAACGTGCAGCTTTTTTTGTGACTATAATATATGTTTGGGGCC    1559
            ********************************** *************************

LA2093      AAAACTTGACGGCAACACTTTCAAAGTTCAGAGATAATGGACCCAATCCTCTGCCCTTCC    1619
E42         AAAACTTGACGGCAACACTTTCAAAGTTCAGAGATAATGGACCCAATCCTCTGCCCTTCC    1618
HEINZ       AAAACTTGACGGCAACACTTTCAAAGTTCAGAGATAATGGACCCAATCCTCTGCCCTTCC    1619
            ************************************************************

LA2093      CACTTAAATGCCGAACTTAGTTTGCATGGCGCAGGACTCGAATTTGTGACCCAACTCACA    1679
E42         CACTTAAATGCCGAACTTAGTTTGCATGGCGCAGGACTCGAATTTGTGACCCAACTCACA    1678
HEINZ       CACTTAAATGCCGAACTTAGTTTGCATGGCGCAGGACTCGAATTTGTGACCCAACTCACA    1679
            ************************************************************

LA2093      AGTATAACATGGCATTAAAATGTTTGTCTTGCTTTAAGAACCCAGAATAAAAAATACTCT    1739
```

```
E42         AGTATAACATGGCATTAAAATGTTTGTCTTGCTTTAAGAACCCAGAATAAAAAATACTCT    1738
HEINZ       AGTATAACATGGCATTAAAATGTTTGTCTTGCTTTAAGAACCCAGAATAAAAAATACTCT    1739
            ************************************************************

LA2093      AATTCCAAGATTTTCAGGAAATGATAATCTGTTCCTAAATGACACTTTTATCTATCGAAC    1799
E42         AATTCCAAGATTTTCAGGAAATGATAATCTGTTCCTAAATGACACTTTTATCTATCGAAC    1798
HEINZ       AATTCCAAGATTTTCAGGAAATGATAATCTGTTCCTAAATGACACTTTTATCTATCGAAC    1799
            ************************************************************

LA2093      TGGTAAATGAACCAAATTGAACACTACTTCGCATGAATCTTTCAAGATAATATAAATTAC    1859
E42         TGGTAAATGAACCAAATTGGACACTACTTCGCATGAATCTTTCAAGATAATATAAATTAC    1858
HEINZ       TGGTAAATGAACCAAATTGGACACTACTTCGCATGAATCTTTCAAGATAATATAAATTAC    1859
            ******************* ****************************************

LA2093      TATGAAATTTAGATACCTTTTGTGGTCGATTGAAAACCAATGATCCTTTGTATTCTACCC    1919
E42         TATGAAATTTAGATACCTTTTGTGGTCGATTGAAAACCAATGATCCTTTGTATTCTACCC    1918
HEINZ       TATGAAATTTAGATACCTTTTGTGGTCGATTGAAAACCAATGATCCTTTGTATTCTACCC    1919
            ************************************************************

LA2093      ATCCATCCCCATCTTTGGCTTGGTGATATTGACAACAATCCTGTACACCAGCAGCTTTCA    1979
E42         ATCCATCCCCATCTTTGGCTTGGTGATATTGACAACAATCCTGTACACCAGCAGCTTTCA    1978
HEINZ       ATCCATCCCCATCTTTGGCTTGGTGATATTGACAACAATCCTGTACACCAGCAGCTTTCA    1979
            ************************************************************

LA2093      TTAAAATTTGAACAAGTGAAAGAGAGGAATAGCAAGTTAGCAACCATAGTAGACAATTTA    2039
E42         TTAAAATTTGAACAAGTGAAAGAGAGGAATAGCAAGTTAGCAACCATAGTAGACAATTTA    2038
HEINZ       TTAAAATTTGAACAAGTGAAAGAGAGGAATAGCAAGTTAGCAACCATAGTAGACAATTTA    2039
            ************************************************************

LA2093      ATGATTGCACAACCTGGCACCATCTTGCCTTGACCTTTGTCCTATTGGTGCAGATCCAAA    2099
E42         ATGATTGCACAACCTGGCACCATCTTGCCTTGACCTTTGTCCTATTGGTGCAGATCCAAA    2098
HEINZ       ATGATTGCACAACCTGGCACCATCTTGCCTTGACCTTTGTCCTATTGGTGCAGATCCAAA    2099
            ************************************************************

LA2093      TGTGTGAGTTGCCACATTTTGTGCAGTGGATACGCCTTGACTCTTCTGAGCAGTAGTTGG    2159
E42         TGTGTGAGTTGCCACATTTTGTGCAGTGGATACGCCTTGACTCTTCTGAGCAGTAGTTGG    2158
HEINZ       TGTGTGAGTTGCCACATTTTGTGCAGTGGATACGCCTTGACTCTTCTGAGCAGTAGTTGG    2159
            ************************************************************

LA2093      AGGATTCCTGCACCATACCAGATAAGCCATTTGTAAATATTAAGCCTTCCCTTTTAAGAT    2219
E42         AGGATTCCTGCACCATACCAGATAAGCCATTTGTAAATATTAAGCCTTCCCTTTTAAGAT    2218
HEINZ       AGGATTCCTGCACCATACCAGATAAGCCATTTGTAAATATTAAGCCTTCCCTTTTAAGAT    2219
            ************************************************************

LA2093      CGGAAGATCAACTTTGGCATCTTATTTATGACCTCAAAGAAATTGACATGGAACTGGTGG    2279
E42         CGGAAGATCAACTTTGGCATCTTATTTATGACCTCAAAGAAATTGACATGGAACTGGTGG    2278
HEINZ       CGGAAGATCAACTTTGGCATCTTATTTATGACCTCAAAGAAATTGACATGGAACTGGTGG    2279
            ************************************************************

LA2093      CTTGCAAGAAAGGCATCCGATATTTTTATTTTATTTTTTACATCCATCAATATTCAATAA    2339
E42         CTTGCAAGAAAGGCATCCGATATTTTTATTTTATTTTTTACATCCATCAATATTCAATAA    2338
HEINZ       CTTGCAAGAAAGGCATCCGATATTTTTATTTTATTTTTTACATCCATCAATATTCAATAA    2339
            ************************************************************

LA2093      CTATTTGCATGAGTTTCCCGTAGATCATAAAGCTGAACAGGTCTTAGATTGTGCCATTGA    2399
E42         CTATTTGCATGAGTTTCCCGTAGATCATAAAGCTGAACAGGTCTTAGATTGTGCCATTGA    2398
HEINZ       CTATTTGCATGAGTTTCCCGTAGATCATAAAGCTGAACAGGTCTTAGATTGTGCCATTGA    2399
            ************************************************************

LA2093      ACTTCTCATTCCAAAAGAAATTCTTTCTTCTGAAAATTCCAGGATACCATACAATTTCTG    2459
E42         ACTTCTCATTCCAAAAGAAATTCTTTCTTCTGAAAATTCCAGGATACCATACAATTTCTG    2458
HEINZ       ACTTCTCATTCCAAAAGAAATTCTTTCTTCTGAAAATTCCAGGATACCATACAATTTCTG    2459
            ************************************************************

LA2093      CATGCTCACCCGAAATTGTCGTTACACCTTGAAAGCTTATCATGCTTTGACATGGATCTT    2519
E42         CATGCTCACCCGAAATTGTCGTTACACCTTGAAAGCTTATCATGCTTTGACATGGATCTT    2518
HEINZ       CATGCTCACCCGAAATTGTCGTTACACCTTGAAAGCTTATCATGCTTTGACATGGATCTT    2519
            ************************************************************

LA2093      ATTGGTATTACATAATTTAATTTGGTGTCATCATTGCAAGAAATAGGGCTAAGACAGTTC    2579
E42         ATTGGTATTACATAATTTAATTTGGTGTCATCATTGCAAGAAATAGGGCTAAGACAGTTC    2578
HEINZ       ATTGGTATTACATAATTTAATTTGGTGTCATCATTGCAAGAAATAGGGCTAAGACAGTTC    2579
```

```
              ************************************************************

LA2093        AGGTGAAGGACAGGGCAGACACTGAATCTAGTCTTTCCCAGTTAAAGAGATACAACTAGC    2639
E42           AAGTGAAGGACAGGGCAGACACTGAATCTAGTCTTTCCCAGTTAAAGAGATACAACTAGC    2638
HEINZ         AAGTGAAGGACAGGGCAGACACTGAATCTAGTCTTTCCCAGTTAAAGAGATACAACTAGC    2639
              * **********************************************************

LA2093        TCTCATTGAAACTTTTCCCGCTTGGATAATTTTGTATGCAGGATAAGATGTTGAATTCTG    2699
E42           TCTCATTGAAACTTTTCCCGCTTGGATAATTTTGTATGCAGGATAAGATGTTGAATTCTG    2698
HEINZ         TCTCATTGAAACTTTTCCCGCTTGGATAATTTTGTATGCAGGATAAGATGTTGAATTCTG    2699
              ************************************************************

LA2093        ACAGGTTTACTGGAGATAAATGAAGTTCAGTCAGACACTTGAACAGACCTGGTTGGACGT    2759
E42           ACAGGTTTACTGGAGATAAATGAAGTTCAGTCAGACACTTGAACAGACCTGGTTGGACGT    2758
HEINZ         ACAGGTTTACTGGAGATAAATGAAGTTCAGTCAGACACTTGAACAGACCTGGTTGGACGT    2759
              ************************************************************

LA2093        GCTAGGTGATTTATGCAGTACACTCTTAGATTCTTCCTTTCTGAGTTGCTCATCATAGTC    2819
E42           GCTAGGTGATTTTTGCAGTACACTCTTAGATTCTTCCTTTCTGAGTTGCTCATCATAGTC    2818
HEINZ         GCTAGGTGATTTTTGCAGTACACTCTTAGATTCTTCCTTTCTGAGTTGCTCATCATAGTC    2819
              ************ ***********************************************

LA2093        TCGCTTCTTGACAGCATCAGAAAGAACCTAAAGGCAAGTGACAGTATTAGAAAACCATAG    2879
E42           TCGCTTCTTGACAGCATCAGAAAGAACCTAAAGGCAAGTGACAGTATTAGAAAACCATAG    2878
HEINZ         TCGCTTCTTGACAGCATCAGAAAGAACCTAAAGGCAAGTGACAGTATTAGAAAACCATAG    2879
              ************************************************************

LA2093        CAGCATGTTTGCATCTCAAACAAATAGGAAGCAAGCCAACAATAGTATTATAACAGCATA    2939
E42           CAGCATGTTTGCATCTCAAACAAATAGGAAGCAAGCCAACAATAGTATTATAACAGCATA    2938
HEINZ         CAGCATGTTTGCATCTCAAACAAATAGGAAGCAAGCCAACAATAGTATTATAACAGCATA    2939
              ************************************************************

LA2093        ACAGATAATTTAACTATTAAAATAGTGATCACCTCATATCCACATTGAAGTTTCTTAAAT    2999
E42           ACAGATAATTTAACTATTAAAATAGTGATCACCTCATATCCACATTGAAGTTTCTTAAAT    2998
HEINZ         ACAGATAATTTAACTATTAAAATAGTGATCACCTCATATCCACATTGAAGTTTCTTAAAT    2999
              ************************************************************

LA2093        GATTCACTGGCAAGTGAACTTCCCATGTTTTTGTCCGGGTGCACAAGCATTGCCTGCGCA    3059
E42           GATTCACTGGCAAGTGAACTTCCCATGTTTTTGTCCGGGTGCACAAGCATTGCCTGCGCA    3058
HEINZ         GATTCACTGGCAAGTGAACTTCCCATGTTTTTGTCCGGGTGCACAAGCATTGCCTGCGCA    3059
              ************************************************************

LA2093        TTTCATGTATGTAAAATTAGCATCAATCTGAACTTCGTCTAGACGGAAAGTAAATATATC    3119
E42           TTTCATGTATGTAAAATTAGCATCAATCTGAACTTCGTCTAGACGGAAAGTAAATATATC    3118
HEINZ         TTTCATGTATGTAAAATTAGCATCAATCTGAACTTCGTCTAGACGGAAAGTAAATATATC    3119
              ************************************************************

LA2093        ATCGGATCAGGCATGAAATTTGTTATCTTGCGTCAAAGAAATGGACACTTTGACGCCTAA    3179
E42           ATCGGATCAGGCATGAAATTTGTTATCTTGCGTCAAAGAAATGGACACTTTGACGCCTAA    3178
HEINZ         ATCGGATCAGGCATGAAATTTGTTATCTTGCGTCAAAGAAATGGACACTTTGACGCCTAA    3179
              ************************************************************

LA2093        CTCAAACCCATCAAACTAATCAAAAGATGGATGAGTAATCAACATCAATTTGATCTGACA    3239
E42           CTCAAACCCATCAAACTAATCAAAAGATGGATGAGTAATCAACATCAATTTGATCTGACA    3238
HEINZ         CTCAAACCCATCAAACTAATCAAAAGACGGATGAGTAATCAACATCAATTTGATCTGACA    3239
              *************************** ********************************

LA2093        CGTAAGGATATCAAAAAGATGGATGAGTAATCAACATCAATTTGACACGTTCTAATTTGT    3299
E42           CGTAAGGATATCAAAAAGATGGATGAGTAATCAACATCAATTTGACACGTTCTAATTTGT    3298
HEINZ         CGTAAGGATATCAAAAAGATGGATGAGTAATCAACATCAATTTGACACGTTCTAATTTGT    3299
              ************************************************************

LA2093        TAGGGTAAATTGAAAATGAATGAAACTCCGGTAGATGTCTCTCAAACCCATCACTTGGCT    3359
E42           TAGGGTAAATTGAAAATGAATGAAACTCCGGTAGATGTCTCTCAAACCCATCACTTGGCT    3358
HEINZ         TAGGGTAAATTGAAAATGAATGAAACTCCGGTAGATGTCTCTCAAACCCATCACTTGGCT    3359
              ************************************************************

LA2093        TTAAAGCCTGTCACATGATATATAATGCATAGGCATGATAAGAAAGTGGCACACCTTTTT    3419
E42           TTAAAGCCTGTCACATGATATATAATGCATAGGCATGATAAGAAAGTGGCACACCTTTTT    3418
HEINZ         TTAAAGCCTGTCACATGATATATAATGCATAGGCATGATAAGAAAGTGGCACACCTTTTT    3419
              ************************************************************
```

```
LA2093      ACGATATTCTTTTTTCAACAGTAAAGTGTCAATTTTCTTATGCCGAGAAACCCCTAGTGC     3479
E42         ACGATATTCTTTTTTCAACAGTAAAGTGTCAATTTTCTTATGCCGAGAAACCCCTAGTGC     3478
HEINZ       ACGATATTCTTTTTTCAACAGTAAAGTGTCAATTTTCTTATGCCGAGAAACCCCTAGTGC     3479
            ************************************************************

LA2093      TTCATAATGATCCGCACTATCTAATATCCGCTTCATCTCAATTATTGAACTTGCATCTTC     3539
E42         TTCATAATGATCCGCACTATCTAATATCCGCTTCATCTCAATTATTGAACTTGCATCTTC     3538
HEINZ       TTCATAATGATCCGCACTATCTAATATCCGCTTCATCTCAATTATTGAACTTGCATCTTC     3539
            ************************************************************

LA2093      TCTAACTACTGGCTTAGCTGGGGGTTCTATCTGTTTGGTTACAACTGATGAAGTAGAAGT     3599
E42         TCTAACTACTGGCTTAGCTGGGGGTTCTATCTGTTTGGTTACAACTGATGAAGTAGAAGT     3598
HEINZ       TCTAACTACTGGCTTAGCTGGGGGTTCTATCTGTTTGGTTACAACTGATGAAGTAGAAGT     3599
            ************************************************************

LA2093      AGAAGCAGCGTCGCTGAATGTTTTGCAAGCGTGAACCTTTTCAACTTTTTCAACCTTTTC     3659
E42         AGAAGCAGCGTCGCTGAATGTTTTGCAAGCGTGAACCTTTTCAACTTTTTCAACCTTTTC     3658
HEINZ       AGAAGCAGCGTCGCTGAATGTTTTGCAAGCGTGAACCTTTTCAACTTTTTCAACCTTTTC     3659
            ************************************************************

LA2093      AGGTTCATCCAGAGGAACTGATGACTCATAATCTGTGGAGAAGTCATCCTCAGTAAATGT     3719
E42         AGGTTCATCCAGAGGAACTGATGACTCATAATCTGTGGAGAAGTCATCCTCAGTAAATGT     3718
HEINZ       AGGTTCATCCAGAGGAACTGATGACTCATAATCTGTGGAGAAGTCATCCTCAGTAAATGT     3719
            ************************************************************

LA2093      TTCGGACTCTTTAAAATCTTCAACATGTGTGCCTTCACTCAAATTGTCACACCACTGGAG     3779
E42         TTCGGACTCTTTAAAATCTTCAACATGTGTGCCTTCACTCAAATTGTCACACCACTGGAG     3778
HEINZ       TTCGGACTCTTTAAAATCTTCAACATGTGTGCCTTCACTCAAATTGTCACACCACTGGAG     3779
            ************************************************************

LA2093      TAAGTAGTTCACTAAGTCATTTGAGAGGAAAGCGAGATTTATTGAGAGACAAACTCCAAG     3839
E42         TAAGTAGTTCACTAAGTCATTTGAGAGGAAAGCGAGATTTATTGAGAGACAAACTCCAAG     3838
HEINZ       TAAGTAGTTCACTAAGTCATTTGAGAGGAAAGCGAGATTTATTGAGAGACAAACTCCAAG     3839
            ************************************************************

LA2093      CCATCCAACTCGAACTTTAACACAATACATAGCATATATGGTAGCCATTAATACCACAAG     3899
E42         CCATCCAACTCGAACTTTAACACAATACATAGCATATATGTTAGCCATTAATACCACAAG     3898
HEINZ       CCATCCAACTCGAACTTTAACACAATACATAGCATATATGGTAGCCATTAATACCACAAG     3899
            **************************************** *******************

LA2093      TCTTGCATGGTTTAATGAAAACAGATAACCTACAAAACCACC-AAACAAGGATCAAATGA     3958
E42         TCTTGCATGGTTTAATGAAAACAGATAACCTACAAAACCACCAAGACAAGGATCAAATGA     3958
HEINZ       TCTTGCATGGTTTAATGAAAACAGATAACCTACAAAACCACCAAGACAAGGATCAAATGA     3959
            ****************************************** * ***************

LA2093      TGCATAACTTCTGAAAATACGGTATATCATACATACATGTGTTGCACTGGGAAATTAAAC     4018
E42         TGCATAACTTCTGAAAATACGGTATATCATACATACATGTGTTGCACTGGGAAATTAAAC     4018
HEINZ       TGCATAACTTCTGAAAATACGGTATATCATACATACATGTGTTGCACTGGGAAATTAAAC     4019
            ************************************************************

LA2093      TTGAGAAATATATCAGATAGAAGCAATACCATAAGGTGTGTAAAAACAAGTCAAAAAAGT     4078
E42         TTGAGAAATATATCAGATAGAAGCAATACCATAAGGTGTGTAAAAACAAGTCAAAAAAGT     4078
HEINZ       TTGAGAAATATATCAGATAGAAGCAATACCATAAGGTGTGTAAAAACAAGTCAAAAAAGT     4079
            ************************************************************

LA2093      AGGATCAATTCCATAATATCACCTTTCTTTCCTATTTTAAACTTTTCTATAAGTTGCATG     4138
E42         AGGATCAATTCCATAATATCACCTTTCTTTCCTATTTTAAACTTTTCTATAAGTTGCATG     4138
HEINZ       AGGATCAATTCCATAATATCACCTTTCTTTCCTATTTTAAACTTTTCTATAAGTTGCATG     4139
            ************************************************************

LA2093      TGTCTCCAGTACAGGGAAATTACAAACTAACAACATAGATGCAGTCCACACATCAAATAC     4198
E42         TGTCTCCAGTACAGGGAAATTACAAACTAACAACATAGATGCAGTCCACACATCAAATAC     4198
HEINZ       TGTCTCCAGTAGAGGGAAATTACAAACTAACAACATAGATGCAGTCCACACATCAAATAC     4199
            *********** ************************************************

LA2093      TCCAGGATCCTATGTCTACAGCAAGATCCACCTGGCTGGACTGATACAATACCTTTGAAA     4258
E42         TCCAGGATCCTATGTCTACAGCAAGATCCACCTGGCTGGACTGATACAATACCTTTGAAA     4258
HEINZ       TCCAGGATCCTATGTCTACAGCAAGATCCACCTGGCTGGACTGATACAATACCTTTGAAA     4259
            ************************************************************

LA2093      GTCACCAGTCGGCCTTTGAATGTACAAAATTATGCTTCCATGCAATTAAACTATCTACTT     4318
E42         GTCACCAGTCGGCCTTTGAATGTACAAAATTATGCTTCCATGCAATTAAACTATCTACTT     4318
```

```
HEINZ        GTCACCAGTCGGCCTTTGAATGTACAAAATTATGCTTCCATGCAATTAAACTATCTACTT    4319
             ************************************************************

LA2093       TTTTGCTAGCCCAACCCAATCTTAAAACAAATGCCTTGCTAAGTTTTCTTCCCCATCGGA    4378
E42          TTTTGCTAGCCCAACCCAATCTTAAAACAAATGCCTTGCTAAGTTTTCTTCCCCATCGGA    4378
HEINZ        TTTTGCTAGCCCAACCCAATCTTAAAACAAATGCCTTGCTAAGTTTTCTTCCCCATCGGA    4379
             ************************************************************

LA2093       ATGTTGTAGAACAAATAAAATAGTGATATATTTACTTTTGGTTGCATATAAATTTATAAT    4438
E42          ATGTTGTAGAACAAATAAAATAGTGATATATTTACTTTTGGTTGCATATAAATTTATAAT    4438
HEINZ        ATGTTGTAGAACAAATAAAATAGTGATATATTTACTTTTGGTTGCATATAAATTTATAAT    4439
             ************************************************************

LA2093       ATTTAAACAAATAATATTTAACCAAAATCTCAGGTAATGAAGACTTGAAGAGATTCAAGC    4498
E42          ATTTAAACAAATAATATTTAACCAAAATCTCAGGTAATGAAGACTTGAAGAGATTCAAGC    4498
HEINZ        ATTTAAACAAATAATATTTAACCAAAATCTCAGGTAATGAAGACTTGAAGAGATTCAAGC    4499
             ************************************************************

LA2093       TAGCAATAGTATCACCCTTATAACGGTGTGAAGCTATCTAGTCGTGCGTCCATTTATTAG    4558
E42          TAGCAATAGCATCACCCTTATAACGGTGTCAAGCTATCTAGTCGTGCGTCCATTTATTAG    4558
HEINZ        TAGCAATAGCATCACCCTTATAACGGTGTCAAGCTATCTAGTCGTGCGTCCATTTATTAG    4559
             ********* ******************* ******************************

LA2093       GTGATTTAGGTCTCACTTTTTACTGAATAGAATGGTTTCATCCATGAATGACAAGCATCA    4618
E42          GTGATTTAGGTCTCACTTTTTACGGAATAGAATGGTTTCATCCATGAATGACAAGCATCA    4618
HEINZ        GTGATTTAGGTCTCACTTTTTACGGAATAGAATGGTTTCATCCATGAATGACAAGCATCA    4619
             *********************** ************************************

LA2093       ATGCACAAGAAATTAAGCAATTTGGTTGAACTACAAAACAAACCTACCTCCAACTATAAA    4678
E42          ATGCACAAGAAATTAAGCAATTTGGTTGAACTACAAAACAAACCTACCTCCAACTATAAA    4678
HEINZ        ATGCACAAGAAATTAAGCAATTTGGTTGAACTACAAAACAAACCTACCTCCAACTATAAA    4679
             ************************************************************

LA2093       CAATACACCAGTAATCCAAAAGTTGGCATACATCCACAAAACTAAAATAGCGAAGAGCCC    4738
E42          CAATACACCAGTAATCCAAAAGTTGGCATACATCCACAAAACTAAAATAGCGAAGAGCCC    4738
HEINZ        CAATACACCAGTAATCCAAAAGTTGGCATACATCCACAAAACTAAAATAGCGAAGAGCCC    4739
             ************************************************************

LA2093       AACAATGAAAAGCCCAGGAGTGCAACCCAAATACTGAACAGCAGCCCCTGCAGCACCCTG    4798
E42          AACAATGAAAAGCCCAGGAGTGCAACCCAAATACTGAACAGCAGCCCCTGCAGCACCCTG    4798
HEINZ        AACAATGAAAAGCCCAGGAGTGCAACCCAAATACTGAACAGCAGCCCCTGCAGCACCCTG    4799
             ************************************************************

LA2093       AAAGGCAAATCAACATAACATATCATCAGCATGTCATTAGTGAGCATTTGAAAACAAAGT    4858
E42          AAAGGCAAATCAACATAACATATCATCAGCATGTCATTAGTGAGCATTTGAAAACAAAGT    4858
HEINZ        AAAGGCAAATCAACATAACATATCATCAGCATGTCATTAGTGAGCATTTGAAAACAAAGT    4859
             ************************************************************

LA2093       GATTCTGCATAAGAACACTGAACAGTCTCAAGAGACCATCTGAAATGGGAAATGAAACAA    4918
E42          GATTCTGCATAAGAACACTGAACAGTCTCAAGAGACCATCTGAAATGGGAAATGAAACAA    4918
HEINZ        GATTCTGCATAAGAACACTGAACAGTCTCAAGAGACCATCTGAAATGGGAAATGAAACAA    4919
             ************************************************************

LA2093       GTAGAATAGCATACTGAGTATATTTTTTCAACAAACAGAGTCCAATGAATAGTACTGGCA    4978
E42          GTAGAATAGCATACTGAGTATATTTTTTCAACAAACAGAGTCCAATGAATAGTACTGGCA    4978
HEINZ        GTAGAATAGCATACTGAGTATATTTTTTCAACAAACAGAGTCCAATGAATAGTACTGGCA    4979
             ************************************************************

LA2093       CAAGACTAAACAAGAAGCTAGAAGAGACTTTTTTCATCAACTATCGATGTTTACCATTTA    5038
E42          CAAGACTAAACAAGAAGCTAGAAGAGACTTTTTTCATCAACTATCGATGTTTACCATTTA    5038
HEINZ        CAAGACTAAACAAGAAGCTAGAAGAGACTTTTTTCATCAACTATCGATGTTTACCATTTA    5039
             ************************************************************

LA2093       TCATTCAAGAACTAGAGATGAAGTTGAACTTCAAGCACTCTAATTGAGCTATAAATTATA    5098
E42          TCATTCAAGAACTAGAGATGAAGTTGAACTTCAAGCACTCTAATTGAGCTATAAATTATA    5098
HEINZ        TCATTCAAGAACTAGAGATGAAGTTGAACTTCAAGCACTCTAATTGAGCTATAAATTATA    5099
             ************************************************************

LA2093       ATAACCTATTTGAGTTCTGAAAAGTTTGAATTTCAATACATACTTCAGATATCTGTATGG    5158
E42          ATAACCTATTTGAGTTCTGAAAAGTTTGAATTTCAATACATACTTCAGATATCTGTATGG    5158
HEINZ        ATAACCTATTTGAGTTCTGAAAAGTTTGAATTTCAATACATACTTCAGATATCTGTATGG    5159
             ************************************************************
```

```
LA2093      ACATCTGTATGGCCTAAAACACAGGGAAAAGAGTGGAACTTTGCTTTTATCTCTAGTACA    5218
E42         ACATCTGTATGGCCTAAAACACAGGGAAAAGAGTGGAACTTTGCTTTTATCTCTAGTACA    5218
HEINZ       ACATCTGTATGGCCTAAAACACAGGGAAAAGAGTGGAACTTTGCTTTTATCTCTAGTACA    5219
            ************************************************************

LA2093      ACCTTCAATGAGTTGGATATCTAAGTAGGTAAAAGTAAGACTCCAACATAATAAACTTGA    5278
E42         ACCTTCAATGAGTTGGATATCTAAGTAGGTAAAAGTAAGACTCCAACATAATAAACTTGA    5278
HEINZ       ACCTTCAATGAGTTGGATATCTAAGTAGGTAAAAGTAAGACTCCAACATAATAAACTTGA    5279
            ************************************************************

LA2093      ACATCACCAAGCAACATATAATTGCTCATGTGACAATAAGACAAAGTTAACTTATTTGAT    5338
E42         ACATCACCAAGCAACATATAATTGCTCATGTGACAATAAGACAAAGTTAACTTATTTGAT    5338
HEINZ       ACATCACCAAGCAACATATAATTGCTCATGTGACAATAAGACAAAGTTAACTTATTTGAT    5339
            ************************************************************

LA2093      TTATGAAGACTATGAAAAGATCATCACAAGAACCAAAAACTGTAGTACTTTCTCGTAGTT    5398
E42         TTATGAAGACTATGAAAAGATCATCACAAGAACCAAAAACTGTAGTACTTTCTCGTAGTT    5398
HEINZ       TTATGAAGACTATGAAAAGATCATCACAAGAACCAAAAACTGTAGTACTTTCTCGTAGTT    5399
            ************************************************************

LA2093      TATCCTGCAAAAGATACTAGTGCACAATTGCAGCATTCAATTCATGTCCAACTTGACCAA    5458
E42         TATCCTGCAAAAGATACTAGTGCACAATTGCAGCATTCAATTCATGTCCAACTTGACCAA    5458
HEINZ       TATCCTGCAAAAGATACTAGTGCACAATTGCAGCATTCAATTCATGTCCAACTTGACCAA    5459
            ************************************************************

LA2093      TGAGCTTTAGGTAAAATCAATGAACATGTAGATATGTTCAAGTTCTTATTTGATGTGTCA    5518
E42         TGAGCTTTAGGTAAAATCAATGAACATGTAGATATGTTCAAGTTCTTATTTGATGTGTCA    5518
HEINZ       TGAGCTTTAGGTAAAATCAATGAACATGTAGATATGTTCAAGTTCTTATTTGATGTGTCA    5519
            ************************************************************

LA2093      TTATATGTCTTACTAGTACAATGCATGTGCAATAAACCCAGGATCAAGCTAGCTAATCAG    5578
E42         TTATATGTCTTACTAGTACAATGCATGTGCAATAAACCCAGGATCAAGCTAGCTAATCAG    5578
HEINZ       TTATATGTCTTACTAGTACAATGCATGTGCAATAAACCCAGGATCAAGCTAGCTAATCAG    5579
            ************************************************************

LA2093      CAATTAATGAAGAGGAAGCAGATCTTGCTAAGTCACGAACCATACAGGAAATTAAAGAAA    5638
E42         CAATTAATGAAGAGGAAGCAGATCTTGCTACGTCACGAACCATACAGGAAATTAAAGAAA    5638
HEINZ       CAATTAATGAAGAGGAAGCAGATCTTGCTAAGTCACGAACCATACAGGAAATTAAAGAAA    5639
            ****************************** *****************************

LA2093      ATGGAATAGAATCATACCATACTAAGGAGCACGTAAACCAGACAAGACAATGATGTCAAA    5698
E42         ATGGAATAGAATCATACCATACTAAGGAGCACGTAAACCAGACAAGACAATGATGTCAAA    5698
HEINZ       ATGGAATAGAATCATACCATACTAAGGAGCACGTAAACCAGACAAGACAATGATGTCAAA    5699
            ************************************************************

LA2093      CTTAGAAAACAGCTCCACATAATAAGGAGCAATGCCGCGGATCCAAGTGCAAAGAACGAT    5758
E42         CTTAGAAAACAGCTCCACATAATAAGGAGCAATGCCGCGGATCCGAGTGCAAAGAACGAT    5758
HEINZ       CTTAGAAAACAGCTCCACATAATAAGGAGCAATGCCGCGGATCCAAGTGCAAAGAACGAT    5759
            ******************************************** ***************

LA2093      TTACAGCCCCTTACTAAACAATCCTTCCAGTATCTGAACAAGATAAAGAGCAATTTTCCT    5818
E42         TTACAGCCCCTTACTAAACAATCCTTCCAGTATCTGAACAAGATAAAGAGCAATTTTCCT    5818
HEINZ       TTACAGCCCCTTACTAAACAATCCTTCCAGTATCTGAACAAGATAAAGAGCAATTTTCCT    5819
            ************************************************************

LA2093      AGTATTGCACACCCACAGCAAACAATTGGCCAAATCCGCTCATTAAAGATTCCAACTTTA    5878
E42         AGTATTGCACACCCACAGCAAACAATTGGCCAAATCCGCTCATTAAAGATTCCAACTTTA    5878
HEINZ       AGTATTGCACACCCACAGCAAACAATTGGCCAAATCCGCTCATTAAAGATTCCAACTTTA    5879
            ************************************************************

LA2093      TCCCTAAAACAGCCAGCAGCTGTCCTTACAACCGAATAACAATCTTTCTTCGATTGCAGC    5938
E42         TCCCTAAAACAGCCAGCAGCTGTCCTTACAACCGAATAACAATCTTTCTTCGATTGCAGC    5938
HEINZ       TCCCTAAAACAGCCAGCAGCTGTCCTTACAACCGAATAACAATCTTTCTTCGATTGCAGC    5939
            ************************************************************

LA2093      CACATCCAAGCTTGTTTAAATAACCCAATGTCCTCCATTACTAAACTAAATTTACACACC    5998
E42         CACATCCAAGCTTGTTTAAATAACCCAATGTCCTCCATTACTAAACTAAATTTACACACC    5998
HEINZ       CACATCCAAGCTTGTTTAAATAACCCAATGTCCTCCATTACTAAACTAAATTTACACACC    5999
            ************************************************************

LA2093      TAACTGAACAGAACTACAACACCCAAACCCCAAATTACCAAAAATTGCTATTGGTACCCA    6058
```

```
E42        TAACTGAACAGAACTACAACACCCAAACCCCAAATTACCAAAAATTGCTATTGGTACCCA    6058
HEINZ      TAACTGAACAGAACTACAACACCCAAACCCCAAATTACCAAAAATTGCTATTGGTACCCA    6059
           ************************************************************

LA2093     TCCTTTTGATATGAGAACAACTCATTTGACCAAACCTGTGAAATCACCATATCATGAATA    6118
E42        TCCTTTTGATATGAGAACAACTCATTTGACCAAACCTGTGAAATCACCATATCATGAATA    6118
HEINZ      TCCTTTTGATATGAGAACAACTCATTTGACCAAACCTGTGAAATCACCATATCATGAATA    6119
           ************************************************************

LA2093     ACAGTCATTATCTGCTAATCTCACTAAAGAACCCCCTTTTGTGTCATATAAAACAAAACC    6178
E42        ACAGTCATTATCTGCTAATCTCACTAAAGAACCCCCTTTTGTGTCATATAAAACAAAACC    6178
HEINZ      ACAGTCATTATCTGCTAATCTCACTAAAGAACCCCCTTTTGTGTCATATAAAACAAAACC    6179
           ************************************************************

LA2093     CAGATCATAAATTCGTCAAATCCAAACACCCAAGTAGCAAAAACAAGTAAAGACAAGATC    6238
E42        CAGATCATAAATTCGTCAAATCCAAACACCCAAGTAGCAAAAACAAGTAAAGACAAGATC    6238
HEINZ      CAGATCATAAATTCGTCAAATCCAAACACCCAAGTAGCAAAAACAAGTAAAGACAAGATC    6239
           ************************************************************

LA2093     CAGAAGATTCCATCTTCACGCAGTAATAAATGGAGAGTGGCAGTTGTTAGATGAGAT       6295
E42        CAGAAGATTCCATCTTCACGCAGTAATAAATGGAGAGTGGCAGTTGTTAGATGAGAT       6295
HEINZ      CAGAAGATTCCATCTTCACGCAGTAATAAATGGAGAGTGGCAGTTGTTAGATGAGAT       6296
           *********************************************************
```

Solyc11g005400

```
LA2093      AGAAAAATATTATATCTTTTAAAAAAAACCATTATTATGTATAGTCAATGTATAATTTCC     60
E42         AGAAAAATATTGCATCTTTTTAAAAAAATCATTATTATGTATAGTCAATGTATAATTTCC     60
HEINZ       AGAAAAATATTATATCTTTTAAAAAAAACCATTATTATGTATAGTCAATGTATAATTTCC     60
            ***********  ******* ******* *******************************

LA2093      ACGATAGTTATGACTATGTAGTACACTTATAACTTCAAAATTCTGATGAAATTCGATGCG     120
E42         ACGATAGTTATGACT--GTAGTACACTTATAACTTCAAAATTCTGATGAAATTCGATGCG     118
HEINZ       ACGATAGTTATGACTATGTAGTACACTTATAACTTCAAAATTCTGATGAAATTCGATGCG     120
            ***************  *******************************************

LA2093      ATAATACAGGTATGATAACATCCTATTTTCAGAAACCAAGCAACTAGGGTATGTTATGCA     180
E42         ATAATACAAGTATGATCACATCCTATTTTCAGAAACCAAGCAACTAGGGTATGTTTTGCA     178
HEINZ       ATAATACAGGTATGATAACATCCTATTTTCAGAAACCAAGCAACTAGGGTATGTTATGCA     180
            ******** ******* ************************************** ****

LA2093      TGGATGTTTGGTTGTATACAAGACTCCAAAATTTTATTAAATACATTTTCATGACGACTA     240
E42         TGGATGTTTGGTTGTATACAAGACTCCAAAATTTTATTAAATACATTTTCATGACGACTC     238
HEINZ       TGGATGTTTGGTTGTATACAAGACTCCAAAATTTTATTAAATACATTTTCATGACGACTC     240
            *********************************************************** 

LA2093      -------------AGAGCCCCCATTATTGCAAGAGCTAAATTAGAACACATAACACTCAG     287
E42         TGCTGGGAAAGAAAGAGCCCCCATTATTGCAAGAGCTAAATTAGAACACATAACACTCAG     298
HEINZ       TGCTGGGAAAGAAAGAGCCCCCATTATTGCAAGAGCTAAATTAGAACACATAACACTCAG     300
                         ***********************************************

LA2093      --------CTAGCTATTATACTAAGAATGGTGATGTTGGCTTCTGAAACTCCCAAGAAAA     339
E42         CTAG----CTAGCTATTATACTAAGAATGGTGATGTTGGCTTCTGAAACTCCCAAGAAAA     354
HEINZ       CTAGCTAGCTAGCTATTATACTAAGAATGGTGATGTTGGCTTCTGAAACTCCCAAGAAAA     360
                    ****************************************************

LA2093      GAAGCCATAGATACGTCAGATAAAGGTTAAGCTGGGGAGAACGCGTTGCACAATGACTTG     399
E42         GAAGCCATAGATACGTCAGATAAAGGTTAAGCTGGGGAGAACGCGTTGCACAATGACTTG     414
HEINZ       GAAGCCATAGATACGTCAGATAAAGGTTAAGCTGGGGAGAACGCGTTGCACAATGACTTG     420
            ************************************************************

LA2093      TATGCAGTGACACAAGTTTTGAATTTCTCTTCTGCTGCTGCCTGCACAAATTCAATGACT     459
E42         TATGCAGTGACACAAGTTTTGAATTTCTCTTCTGCTGCTGCCTGCACAAATTCAATGACT     474
HEINZ       TATGCAGTGACACAAGTTTTGAATTTCTCTTCTGCTGCTGCCTGCACAAATTCAATGACT     480
            ************************************************************

LA2093      CGTCTCGTGAGGAGGACTCCAAGAATACGAATGAGTAGCTAAATTCCAAAACAAGGTTTC     519
E42         CGTCTCGTGAGGAGGACTCCAAGAATACGAATGAGTAGCTAAATTCCAAAACAAGGTTTC     534
HEINZ       CGTCTCGTGAGGAGGACTCCAAGAATACGAATGAGTAGCTAAATTCCAAAACAAGGTTTC     540
            ************************************************************

LA2093      AAGAAAGGAACCAGATAGGCTATTGTAAGTTGTCACGTAGGAGGACAAACAGAAAGTGTA     579
E42         AAGAAAGGAACCAGATAGGCTATTGTAAGTTGTCACGTAGGAGGACAAACAGAAAGTGTA     594
HEINZ       AAGAAAGGAACCAGATAGGCTATTGTAAGTTGTCACGTAGGAGGACAAACAGAAAGTGTA     600
            ************************************************************

LA2093      AAGCTAACCTGTGAAGGGCCTTGATGTTTATCAGGATGCCACTTTAATGCTGATAAGCGG     639
E42         AAGCTAACCTGTGAAGGGCCTTGATGTTTATCAGGATGCCACTTTAATGCTGATAAGCGG     654
HEINZ       AAGCTAACCTGTGAAGGGCCTTGATGTTTATCAGGATGCCACTTTAATGCTGATAAGCGG     660
            ************************************************************

LA2093      AAACTACAAGGACAATAATAGTGTTTACATTAGTCTTTAATATAATCGAGTATGGAGAGC     699
E42         AAACTGCAAGGACAATAATAGTGTTTACATTAGTCTTCAATATAATCGAGTATGGGGAGC     714
HEINZ       AAACTACAAGGACAATAATAGTGTTTACATTAGTCTTCAATATAATCGAGTATGGAGAGC     720
            ***** ******************************* ***************** ****

LA2093      ACAAAACTACTAGAACTTACGCATTCTTGACATCCTCAATCTTCAGAGGACCTCTTGGGG     759
E42         ACAAAACTACTAGAACTTACGCATTCTTGACATCCTCAATCTTCAGAGGACCTCTTGGGG     774
HEINZ       ACAAAACTACTAGAACTTACGCATTCTTGACATCCTCAATCTTCAGAGGACCTCTTGGGG     780
            ************************************************************

LA2093      GCAAACCGAGAATTGTTCTTTCTGAGGAAGAACCTATATGACACGATTCATATTTGGATT     819
E42         GCAAACCGAGAATTGTTCTTTCTGAGGAAGAACCTATATGACACGATTCATATTTGGATT     834
HEINZ       GCAAACCGAGAATTGTTCTTTCTGAGGAAGAACCTATATGACACGATTCATATTTGGATT     840
            ************************************************************
```

```
LA2093      CTGTATGTGTCTCGTTTTCAGTTTCATTATCCCATCTGTAATGCCTTTGTTCTGACCAGT    879
E42         CTGTATGTGTCTCGTTTTCAGTTTCATTATCCCATCTGTAATGCCTTTGTTCTGACCAGT    894
HEINZ       CTGTATGTGTCTCGTTTTCAGTTTCATTATCCCATCTGTAATGCCTTTGTTCTGACCAGT    900
            ************************************************************

LA2093      CTGTTTCTTGTCTTCGGGAGAACATATCTTCAAAAGTAGATGATTTACCAGGCCTAAAAG    939
E42         CTGTTTCTTGTCTTCGGGAGAACATATCTTCAAAAGTAGATGATTTACCAGGCCTAAAAG    954
HEINZ       CTGTTTCTTGTCTTCGGGAGAACATATCTTCAAAAGTAGATGATTTACCAGGCCTAAAAG    960
            ************************************************************

LA2093      ACCAAGTGTACCACTTATCACCAAAAGTTGCTTCGAATACTGATTCAGGATATTCATCGA    999
E42         AACAAGTGTACCACTTATCACCAAAAGTTGCTTCGAATACTGATTCAGGATATTCATCGA    1014
HEINZ       ACCAAGTGTACCACTTATCACCAAAAGTTGCTTCGAATACTGATTCAGGATATTCATCGA    1020
            * **********************************************************

LA2093      ATTCTTCATATAATCTCCGTTTCTTCATTTTTCCTAAAAACGAATAATTAACGGTCAATG    1059
E42         ATTCTTCATATAATCTCCGTTTCTTCATTTTTCCTAAAAACGAACAATTAACGGTCAATG    1074
HEINZ       ATTCTTCATATAATCTCCGTTTCTTCATTTTTCCTAAAAACGAATAATTAACGGTCAATG    1080
            ******************************************** ***************

LA2093      AAGGCTACCTGCATCCTTCAATAAGCTCAGTATTGCGTCACCATCAACCATGTAAAGATA    1119
E42         AAGGCTACCTGCATCCTTCAATAAGCTCAGTATTGCGTCACCATCAACCATGTAAAGATA    1134
HEINZ       AAGGCTACCTACATCCTTCAATAAGCTCAGTATTGCGTCACCATCAACCATGTAAAGATA    1140
            ********** *************************************************

LA2093      GATTAGCAATCAGAAAGCATAACAGAAGGTTATCTAAAGTTATGACATACATAACTTTGT    1179
E42         GATTAGCAATCAGAAAGCATAACAGAAGGTTATCTAAAGTTATGACATACATAACTTTGT    1194
HEINZ       GATTAGCAATCAGAAAGCATAACAGAAGGTTATCTAAAGTTATGACATACATAACTTTGT    1200
            ************************************************************

LA2093      GACAAACATAAAAAGTCCAATTTCCGTTCTTTAAAGACGCATTAATTCAAGTGTTCAAAT    1239
E42         GACAAACATAAAAAGTCCAATTTCCGTTCTTTAAAGACGCATTAATTCAAGTGTTCAAAT    1254
HEINZ       GACAAACATAAAAAGTCCAATTTCCGTTCTTTAAAGACGCATTAATTCAAGTGTTCAAAT    1260
            ************************************************************

LA2093      CCTTGCAATACAACTCCTAGATGGAAAAAATGCAAAGCAACGCAAGTTCATCCCATTTTA    1299
E42         CCTTGCAATACAACTCCTAGATGGAAAAAATGCAAAGCAACGCAAGTTCATCCCATTTTA    1314
HEINZ       CCTTGCAATACAACTCCTAGATGGAAAAAATGCAAAGCAACGCAAGTTCATCCCATTTTA    1320
            ************************************************************

LA2093      CTTAGAATGACAGCAACCAACTCACGGCCAATAAACCTTTTTTATTTGGAAGACAAGCAT    1359
E42         CTTAGAATGACAGCAACCAACTCACGGCCAATAAACCTTTTTTATTTGGAAGACAAGCAT    1374
HEINZ       CTTAGAATGACAGCAACCAACTCACGGCCAATAAACCTTTTTTATTTGGAAGACAAGCAT    1380
            ************************************************************

LA2093      TGGGATCAATCTGAAAAGGCATTGGTTTCGGCATGTTTCCTGATAGGTAACCCACTAGTC    1419
E42         TGGGATCAATCTGAAAAGGCATTGGTTTCGGCATGTTTCCTGATAGGTAACCCACTAGTC    1434
HEINZ       TGGGATCAATCTGAAAAGGCATTGGTTTCGGCATGTTTCCTGATAGGTAACCCACTAGTC    1440
            ************************************************************

LA2093      CAGCTAGTAGGTGGGCAGTAAAGGAGCTTTCGAAACTATCCCAGCGTCAAAGCACCAAAT    1479
E42         CAGCTAGTAGGTGGGCAGTAAAGGAGCTTTCGAAACTATCCCAGCGTCAAAGCACCAAAT    1494
HEINZ       CAGCTAGTAGGTGGGCAGTAAAGGAGCTTTCGAAACTATCCCAGCGTCAAAGCACCAAAT    1500
            ************************************************************

LA2093      TTCAATAGCAATATATATTTCAGTTATATCTGGTATTTAAGAATCTCAAGAATGGGGCCA    1539
E42         TTCAATAGCAATATATATTTCAGTTATATCTGGTATTTAAGAATCTCAAGAATGGGGCCA    1554
HEINZ       TTCAATAGCAATATATATTTCAGTCATATCTGGTATTTAAGAATCTCAAGAATGGGGCCA    1560
            ************************ ***********************************

LA2093      AAAGGTTGAACACACAGATATTTTTCAGTTTGATATAAGAATTTTCATTAAAAACAGAGA    1599
E42         AAAGGTTGAACACACTGATATTTTTCAGTTTGATATAAGAATTTTCATTAAAAACAGAGA    1614
HEINZ       AAAGGTTGAACACACTGATATTTTTCAGTTTGATATAAGAATTTTCATTAAAAACAGAGA    1620
            *************** ********************************************

LA2093      TCCAATTGTGAAATGAGAGTACAAACTCACGCATCCTTCTCCTTGAGCGATCTCTAAGAC    1659
E42         TCCAATTGTGAAATGAGAGTACAAACTCACGCATCCTTCTCCTTGAGCGATCTCTAAGAC    1674
HEINZ       TCCAATTGTGAAATGAGAGTACAAACTCACGCATCCTTCTCCTTGAGCGATCTCTAAGAC    1680
            ************************************************************

LA2093      GAGCAGCGGACTTATATTCACTTTTCTTCTCTGAACGATCATCCCCATCCATATCCCATC    1719
```

```
E42         GAGCAGCGGACTTATATTCACTTTTCTTCTCTGAACGATCATCCCGATCCATATCCCATC    1734
HEINZ       GAGCAGCGGACTTATATTCACTTTTCTTCTCTGAACGATCATCCCGATCCATATCCCATC    1740
            ********************************************* **************

LA2093      TATCATTTATTGTTGAAGATTCATTCTAGTAAATTATTCAACAAGAAAATAGCACATTGT    1779
E42         TATCATTTATTGTTGAAGATTCATTCTAGTAAATTATTCAACAAGAAAATAGCACATTGT    1794
HEINZ       TATCATTTATTGTTGAAGATTCATTCTAGTAAATTATTCAACAAGAAAATAGCACATTGT    1800
            ************************************************************

LA2093      CCATGAGTAGAACAAATATTGAGGTAAAAATAGTGAAATGAGAAGAGAATTTAATATAAA    1839
E42         CCATGAGTAGAACAAATATTGAGGTAAAAATAGTGAAATGAGAAGAGAATTTAATATAAA    1854
HEINZ       CCATGAGTAGAACAAATATTGAGGTAAAAATAGTGAAATGAGAAGAGAATTTAATATAAA    1860
            ************************************************************

LA2093      AAGAGTATTTGCATGCAATTGATCAGATACTAAAGTTCAGAAGCTGAACACATTATACTA    1899
E42         AAGAGTATTTGCATGCAATTGATCAGATACTAAAGTTCAGAAGCTGAACACATTATACTA    1914
HEINZ       AAGAGTATTTGCATGCAATTGATCAGATACTAAAGTTCAGAAGCTGAACACATTATACTA    1920
            ************************************************************

LA2093      TGCGGACTTTAAACCAGAAAAGGTGGTTCATGCTTTGGAGGCTTGGACCACAGAGGAAGT    1959
E42         TGCGGACTTTGAACCAGAAAAGGTGGTTCATGCTTTGGAGGCTTGGACCACAGAGGAAGT    1974
HEINZ       TGCGGACTTTAAACCAGAAAAGGTGGTTCATGCTTTGGAGGCTTGGACCACAGAGGAAGT    1980
            ********** *************************************************

LA2093      TAAGCAAGCACACTACAGTAGACCATAACTTCCTTAGAAAGCAGAAACCCACAAACAGAG    2019
E42         TAAGCAAGCACACTACAGTAGACCATAACTTCCTTAGAAAGCAGAAACCCACAAACAGAG    2034
HEINZ       TAAGCAAGCACACTACAGTAGACCATAACTTCCTTAGAAAGCAGAAACCCACAAACAGAG    2040
            ************************************************************

LA2093      TCAAGATGCAAGGGCGAAGAAAGGCAAAGCAGTAAAGATCAAAGACAAACTATACATAGG    2079
E42         TCAAGATGCAAGGGCGAAGAAAGGCAAAGCAGTAAAGATCAAAGACAAACTATACATAGG    2094
HEINZ       TCAAGATGCAAGGGCGAAGAAAGGCAAAGCAGTAAAGATCAAAGACAAACTATACATAGG    2100
            ************************************************************

LA2093      ATGAGGTATAAGTGAAAATTTCATCACTGCTTCTGTTACTCCATCTTTTCCTTCTATATA    2139
E42         ATGAGGTATAAGTGAAAATTTCATCACTGCTTCTGTTACTCCATCTTTTCCTTCTATATA    2154
HEINZ       ATGAGGTATAAGTGAAAATTTCATCACTGCTTCTGTTACTCCATCTTTTCCTTCTATATA    2160
            ************************************************************

LA2093      CGAGGAGATTCGTCTTACTACACTGAACTAGTGAACTTGGTCTATCCTCATTACACCTTG    2199
E42         CGAGGAGATTCGTCTTACTACACTGAACTAGTGAACTTGGTCTATCCTCATTACACCTTG    2214
HEINZ       CGAGGAGATTCGTCTTACTACACTGAACTAGTGAACTTGGTCTATCCTCATTACACCTTG    2220
            ************************************************************

LA2093      ATACCTTTGATCATTATGTGACTTAATGCTACAATTGCAAAATGAGTTGTTCCCTCATAG    2259
E42         ATACCTTTGATCATTATGTGACTTAATGCTACAATTGCAAAATGAGTTGTTCCCTCATAG    2274
HEINZ       ATACCTTTGATCATTATGTGACTTAATGCTACAATTGCAAAATGAGTTGTTCCCTCATAG    2280
            ************************************************************

LA2093      ATCCGCCTTTTATCCTCCTAGGATTCCTCAATTCACTTCAAAAGCAAACAGAGGACAAGT    2319
E42         ATCCGCCTTTTATCCTCCTAGGATTCCTCAATTCACTTCAAAAGCAAACAGAGGACAAGT    2334
HEINZ       ATCCGCCTTTCATCCTCCTAGGATTCCTCAATTCACTTCAAAAGCAAACAGAGGACAAGT    2340
            ********** *************************************************

LA2093      GAGATTCATTGAAAAGACAGAAGTTTTTGTGGTTGATTAGGACAGGTTTTTAGGTTACAT    2379
E42         GAGATTCATTGAAAAGACAGAAGTTTTTGTGGTTGATTAGGACAGGTTTTTAGGTTACAT    2394
HEINZ       GAGATTCATTGAAAAGACAGAAGTTTTTGTGGTTGATTAGGACAGGTTTTTAGGTTACAT    2400
            ************************************************************

LA2093      GAACAGATCAAGCGTCATATTCGGAAGCAGACATAAAATAGTTTTTTCTTTGGTTGGGGT    2439
E42         GAACAGATCAAGCGTCATATTCGGAAGCAGACATAAAATAGTTTTTTCTTTGGTTGGGGT    2454
HEINZ       GAACAGATCAAGCGTCATATTCGGAAGCAGACATAAAATAGTTTTTTCTTTGGTTGGGGT    2460
            ************************************************************

LA2093      GGGGTGAGGAAGCGGATATGTTCTAGAAGAAGAGAAGAGAAGAAACAAAAATAACACATA    2499
E42         GGGGTGAGGAAGCGGATATGTTCTAGAAGAAGATAAGAGAAGAAACAAAAATAACACATA    2514
HEINZ       GGGGTGAGGAAGCGGATATGTTCTAGAAGAAGAGAAGAGAAGAAACAAAAATAACACATA    2520
            ********************************* **************************

LA2093      AGAATAGGTCTCGAACTTCTCTCTCAGTTCTTTGGGAACTACGTTAGTATTTTCATGGAA    2559
E42         AGAATAGGTCTCGAACTTCTCTCTCAGTTCTTTGGGAACTACGTTAGTATTTTCATGGAA    2574
HEINZ       AGAATAGGTCTCGAACTTCTCTCTCAGTTCTTTGGGAACTACGTTAGTATT-TCATGGAA    2579
```

```
                   *************************************************** ********

LA2093      ATTCTTGTAAAGAGAATGTGACAGGGTTGAGATGCTTCTTGCTGGAGCTTTCTTTTGACA    2619
E42         ATTCTTGTAAAGAGAATGTGACAGGGTTGAGATGCTTCTTGCTGGAGCTTTCTTTTGACA    2634
HEINZ       ATTCTTGTAAAGAGAATGTGACAGGGTTGAGATGCTTCTTGCTGGAGCTTTCTTTTGACA    2639
            ************************************************************

LA2093      GTTGCGGAATGGCACCGGTGACACTTTTACTAGCGACCGGATGTTTGATGGCTGGAGCCA    2679
E42         GTTGCGGAATGGCACCGGTGACACTTTTACTAGCGACCGGATGTTTGATGGCTGGAGCCA    2694
HEINZ       GTTGCGGAATGGCACCGGTGACACTTTTACTAGCGACCGGATGTTTGATGGCTGGAGCCA    2699
            ************************************************************

LA2093      ATGTTGCAAAGGAAGAGGAACCTTTTCTATAAATTGTTTTTCGCTCATGTTTGGAGTATT    2739
E42         ATGTTGCAAAGGAAGAGGAACCTTTTCTATAAATTGTTTTTCGCTCATGTTTGGAGTATT    2754
HEINZ       ATGTTGCAAAGGAAAAGGAACCTTTTCTATAAATTGTTTTTCGCTCATGTTTGGAGTATT    2759
            ************** *********************************************

LA2093      TACTGGAAAGAAGTAGGGCATACCAAGAGATTAAGGCTTAGTCATGATGGTGTGCCTACA    2799
E42         TACTGGAAAGAAGTAGGGCATACCAAGAGATTAAGGCTTAGTCATGATGGTGTGCCTACA    2814
HEINZ       TACTGGAAAGAAGTAGGGCATACCAAGAGATTAAGGCTTAGTCATGATGGTGTGCCTACA    2819
            ************************************************************

LA2093      TTAGGTTCAATGTTGGCTAGGAGTCTTTTTAGTCTAGTTTAGACCTATAGTGTTGGTGAA    2859
E42         TTAGGTTCAATGTTGGCTAGGAGTCTTTTTAGTCTAGTTTAGACCTATAGTGTTGGTGAA    2874
HEINZ       TTAGGTTCAATGTTGGCTAGGAGTCTTTTTAGTCTAGTTTAGACCTATAGTGTTGGTGAA    2879
            ************************************************************

LA2093      CCTCTAAGTTATTGAGTATTGGGAAGAAATCAATCCAAATGGTAAGAAATGAATAATCAG    2919
E42         CCTCTAAGTTATTGAGTATTGGGAAGAAATCAATCCAAATGGTAAGAAATGAATAATCAG    2934
HEINZ       CCTCTAAGTTATTGAGTATTGGGAAGAAATCAATCCAAATGGTAAGAAATGAATAATCAG    2939
            ************************************************************

LA2093      GATTCACATAGTTGATCCAACAACACTTCATTTGGTGCAAAGATATTTCTTCCTTATATA    2979
E42         GATTCACATAGTTGATCCAACAACACTTCATTTGGTGCAAAGATATTTCTTCCTTATATA    2994
HEINZ       GATTCACATAGTTGATCCAACAACACTTCATTTGGTGCAAAGATATTTCTTCCTTATATA    2999
            ************************************************************

LA2093      ATGATGACATATCTTCTTGGAAGAACTTTCTTTATGATATTTCTTCCTTATATGATGATG    3039
E42         ATGATGACATATCTTCTTGGAAGAACTTTCTTTATGATATTTCTTCCTTATATGATGATG    3054
HEINZ       ATGATGACATATCTTCTTGGAAGAACTTTCTTTATGATATTTCTTCCTTATATGATGATG    3059
            ************************************************************

LA2093      ACATATCTTCTTGGAAGAACTTTCTTTCTTTAGTTGATTATTGCAGTTGGCTATTTGCTT    3099
E42         ACATATCTTTTTGGAAGAACTTTCTTTCTTTAGTTGATTATTGCAGTTGGCTATTTGCTT    3114
HEINZ       ACATATCTTCTTGGAAGAACTTTCTTTCTTTAGTTGATTATTGCAGTTGGCTATTTGCTT    3119
            ********* **************************************************

LA2093      TAATCCACTTTTTGGGGAAACTGGTTCCTCGCATTCCACTGTAGGTGGTAAATAATAATT    3159
E42         TAATCCACTTTTTGGGGAAACTGGTTCCTCGCATTCCACTGTAGGTGGTAAATAATAATT    3174
HEINZ       TAATCCACTTTTTGGGGAAACTGGTTCCTCGCATTCCACTGTAGGTGGTAAATAATAATT    3179
            ************************************************************

LA2093      GTTTCTTTTAGACACCGATACATATGTGAATGCAATCTTGGTGATAAGCTAATGATATCA    3219
E42         GTTTCTTTTAGACACCGATACATATGTGAATGCAATCTTGGTGATAAGCTAATGATATCA    3234
HEINZ       GTTTCTTTTAGACACCGATACATATGTGAATGCAATCTTGGTGATAAGCTAATGATATCA    3239
            ************************************************************

LA2093      CAAATGTAATCTATCAATAAAATACTATCTATTAGCCTGATTTTGGTAAAAGATGGAGAA    3279
E42         CAAATGTAATCTATCAATAAAATACTATCTATTAGCCTGATTTTGGTAAAAGATGGAGAA    3294
HEINZ       CAAATGTAATCTATCAATAAAATACTATCTATTAGCCTGATTTTGGTAAAAGATGGAGAA    3299
            ************************************************************

LA2093      CATAGGAACTCCTATTGGGAAGGACCATAAACAGAAAAAAATAGCAGAACTGATTTTCAGT    3339
E42         CATAGGAACTCCTATTGGGAAGGACCATAAACAGAAAAAAATAGCATAACTGATTTTCAGT    3354
HEINZ       CATAGGAACTCCTATTGGGAAGGACCATAAACAGAAAAAAATAGCAGAACTGATTTTCAGT    3359
            ********************************************** *************

LA2093      TTTCCAAGATACAAAATGACCTTGCGTTCAATGATTGAGGTCAGCAATAGCGATCTAGGA    3399
E42         TTTCCAAGATACAAAATGACCTTGCGTTCAATGATTGAGGTCAGCAATAGCGATCTAGGA    3414
HEINZ       TTTCCAAGATACAAAATGACCTTGCGTTCAATGATTGAGGTCAGCAATAGCGATCTAGGA    3419
            ************************************************************
```

```
LA2093     TGTATTCATGTCGTTTTGGAAGATGATTCCCTTGATAAATGTAGCGTTTGGTTATTTTCA     3459
E42        TGTATTCATGTCGTTTTGGAAGATGATTCCCTTGATAAATGTAGCGTTTGGTTATTTTCA     3474
HEINZ      TGTATTCATGTCGTTTTGGAAGATGATTCCCTTGATAAATGTAGCGTTTGGTTATTTTCA     3479
           ************************************************************

LA2093     CATGCTAAAAGGGCAAATGAATGTGTAAGTCGTCCCTTTTGCAAGTATTTCAACTTGTAC     3519
E42        CATGCTAAAAGGGCAAATGAATGTGTAAGTCGTCCCTTTTGCAAGTATTTCAACTTGTAC     3534
HEINZ      CATGCTAAAAGGGCAAATGAATGTGTAAGTCGTCCCTTTTGCAAGTATTTCAACTTGTAC     3539
           ************************************************************

LA2093     TTCATATCACTTCAAGTCTTCGACTACCAAGTAAATACTATTATCAACCTTCCATACTCA     3579
E42        TTCATATCACTTCAAGTCTTCAACTACCAAGTAAATACTATTATCAACCTTCCATACTCA     3594
HEINZ      TTCATATCACTTCAAGTCTTCAACTACCAAGTAAATACTATTATCAACCTTCCATACTCA     3599
           ******************** ***************************************

LA2093     AAATGATTACTGGAATGACACCCTGTACCACTTCTATTGTTGCCTCAGGTCACCTACCTT     3639
E42        AAATGATTACTGGAATGACACCCTGTACCACTTCTATTGTTGCCTCAGGTCACCTACCTT     3654
HEINZ      AAATGATTACTGGAATGACACCCTGTACCACTTCTATTGTTGCCTCAGGTCACCTACCTT     3659
           ************************************************************

LA2093     ATGTCCAACCAAACACCGGAAACTAATTCAGGAAACAAACTAATCCAAAGAAATTATTTT     3699
E42        ATGTCCAACCAAACACCGGAAACTAATTCAGGAAACAAACTAATCCAAAGAAATTATTTT     3714
HEINZ      ATGTCCAACCAAACACCGGAAACTAATTCAGGAAACAAACTAATCCAAAGAAATTATTTT     3719
           ************************************************************

LA2093     AAATGAAAACAATTTATGTCACTCCATAAAGAGAAAGAAATCCCTTATCAAAGACAGTTG     3759
E42        AAATGAAAACAATTTATGTCACTCCATAAAGAGAAAGAAATCCCTTATCAAAGACAGTTG     3774
HEINZ      AAATGAAAACAATTTATGTCACTCCATAAAGAGAAAGAAATCCCTTATCAAAGACAGTTG     3779
           ************************************************************

LA2093     ACCTAATATAACTAAGACTAAGATTTACCATATCTATGAAATGAAAGAAGATTAAATATA     3819
E42        ACCTAATATAACTAAGACTAAGATTTACCATATCTATGAAATGAAAGAAGATTAAATATA     3834
HEINZ      ACCTAATATAACTAAGACTAAGATTTACCATATCTATGAAATGAAAGAAGATTAAATATA     3839
           ************************************************************

LA2093     TACCTCAAAAGAGTTTCCAGATGCCCCATAAAAGAGAAGATTTTTCAAAGCTTTTTTTGA     3879
E42        TACCTCAAAAGAGTTTCCAGATGCCCCATAAAAGAGAAGATTTTTCAAAGCTTTTTTTGA     3894
HEINZ      TACCTCAAAAGAGTTTCCAGATGCCCCATAAAAGAGAAGATTTTTCAAAGCTTTTTTTGA     3899
           ************************************************************

LA2093     GTCAGCACGCTTTTGACGTGTTTCATATCTGATATAATTCTGAAAAATAAATGAAAAGCT     3939
E42        GTCAGCACGCTTTTGACGTGTTTCATATCTGATATAATTCTGAAAAATAAATGAAAAGCT     3954
HEINZ      GTCAGCACGCTTTTGACGTGTTTCATATCTGATATAATTCTGAAAAATAAATGAAAAGCT     3959
           ************************************************************

LA2093     GAATGATAAATAAGAAAATGAGGAGGACAAGAGAAGTTAATAGATGTATTACATGGAAAG     3999
E42        GAATGATAAATAAGAAAATGAGGAGGACAAGAGAAGTTAATAGATGTATTACATGGAAAG     4014
HEINZ      GAATGATAAATAAGAAAATGAGGAGGACAAGAGAAGTTAATAGATGTATTACATGGAAAG     4019
           ************************************************************

LA2093     AAAAAGAGATGTTAGGAAAAAGAATACATAACTGACCAGAGAAAACCAAGCGAAAAAACT     4059
E42        AAAAAGAGATGTTAGGAAAAAGAATACATAACTGACCAGAGAAAACCAAGCGAAAAAACT     4074
HEINZ      AAAAAGAGATGTTAGGAAAAAGAATACATAACTGACCAGAGAAAACCAAGCGAAAAAACT     4079
           ************************************************************

LA2093     CGTGAAGATATCTCTAATCCTAGCCAATTTCAAACACTATAATATTGGAAGCTGGTGAAG     4119
E42        CGTGAAGATATCTCTAATCCTAGCCAATTTCAAACACTATAATATTGGAAGCTGGTGAAG     4134
HEINZ      CGTGAAGATATCTCTAATCCTAGCCAATTTCAAACACTATAATATTGGAAGCTGGTGAAG     4139
           ************************************************************

LA2093     ATATTTACCTTAGTTGGCTGCTGACTTCCTCTAAAATCCTGCAGGAATAATCTATGAAAG     4179
E42        ATATTTACCTTAGTTGGCTGCTGACTTCCTCTAAAATCCTGCAGGAATAATCTATGAAAG     4194
HEINZ      ATATTTACCTTAGTTGGCTGCTGACTTCCTCTAAAATCCTGCAGGAATAATCTATGAAAG     4199
           ************************************************************

LA2093     GTCACATATGCAAACTGAAGAAAGATACTGCTTGAACTTAAAGGTAACAAGCATGAAAAT     4239
E42        GTCACATATGCAAACTGAAGAAAGATACTGCTTGAACTTAAAGGTAACAAGCATGAAAAT     4254
HEINZ      GTCACATATGCAAACTGAAGAAAGATACTGCTTGAACTTAAAGGTAACAAGCATGAAAAT     4259
           ************************************************************

LA2093     ACACTGTGAATGAGCAGAGAAAACTAAAACTGAGTGCAGAAACACAATAATTTCCTTAAA     4299
E42        ACACTGTGAATGAGCAGAGAAAACTAAAACTGAGTGCAGAAACACAATAATTTCCTTAAA     4314
```

```
HEINZ       ACACTGTGAATGAGCAGAGAAAACTAAAACTGAGTGCAGAAACACAATAATTTCCTTAAA    4319
            ************************************************************

LA2093      TCTACCAGTCTCAAATGGGAATATCGGAACCCTTTATTTGGGGGGTGGGGGTGGATAAGG    4359
E42         TCTACCAGTCTCAAATGGGAATATCGGAACCCTTTATTTGGGGGGTGGGGGTGGATAAGG    4374
HEINZ       TCTACCAGTCTCAAATGGGAATATCGGAACCCTTTATTTGGGGGGTGGGGGTGGATAAGG    4379
            ************************************************************

LA2093      GAGCATACTGACACTGCATATACAAGCAGACATAGAACAAACCACCTCCCTCCCCTCATA    4419
E42         GAGCATACTGACACTGCATATACAAGCAGACATAGAACAAACCACCTCCCTCCCCTCATA    4434
HEINZ       GAGCATACTGACACTGCATATACAAGCAGACATAGAACAAACCACCTCCCTCCCCTCATA    4439
            ************************************************************

LA2093      CTCAAGGAAAAACCTTTTCACTTAATACTACAATAGAGAATTGAAACATAGCACATATAG    4479
E42         CTCAAGGAAAAACCTTTTCACTTAATACTACAATAGAGAATTGAAACATAGCACATATAG    4494
HEINZ       CTCAAGGAAAAACCTTTTCACTTAATACTACAATAGAGAATTGAAACATAGCACATATAG    4499
            ************************************************************

LA2093      GAGGAAGAAGTATACTCCATTCTTGGTGTAATTTTAAGCAGTTTT-TTTTTCTCTAACCA    4538
E42         GAGGAAGAAGTATACTCCATTCTTGGTGTAATTTTAAGCAGTTTTTTTTTTCTCTAACCA    4554
HEINZ       GAGGAAGAAGTATACTCCATTCTTGGTGTAATTTTAAGCAGTTTTTTTTTTCTCTAACCA    4559
            ********************************************* **************

LA2093      TTACGCCTACCGAGGTAACATTTTAAAATTGGACAAATGAACTCCAAACCTTATCTTATT    4598
E42         CTACGCCTACCGAGGTAACATTTTAAAATTGGACAAACGAACTCCAAACCTTATCTTATT    4614
HEINZ       CTACGCCTACCGAGGTAACATTTTAAAATTGGACAAACGAACTCCAAACCTTATCTTATT    4619
             ************************************ **********************

LA2093      TCCTAACCAAAATAATTTAGCATATCTATTTCACCAAGAGCACGGTGAACTGGCGCCAAG    4658
E42         TCCTAACCAAAATAATTTAGCATATCTATTTCACCAAGAGCACGGTGAACTGGCGCCAAG    4674
HEINZ       TCCTAACCAAAATAATTTAGCATATCTATTTCACCAAGAGCACGGTGAACTGGCGCCAAG    4679
            ************************************************************

LA2093      ATTTAACCATGTTCAAACAAGAGACATGTGAGGTTTTGGCAACAATTAACTGGCTTAAAC    4718
E42         ATTTAACCATGTTCAAACAAGAGACATGTGAGGTTTTGGCAACAATTAACTGGCTTAAAC    4734
HEINZ       ATTTAACCATGTTCAAACAAGAGACATGTGAGGTTTTGGCAACAATTAACTGGCTTAAAC    4739
            ************************************************************

LA2093      CAGCCTGAAACTGGAAAAGAATGACAAAAACCTTTGATTTATTAACCAATTCCTGCTTTC    4778
E42         CAGCCTGAAACTGGAAAAGAATGACAAAAACCTTTGATTTATTAACCAATTCCTGCTTTC    4794
HEINZ       CAGCCTGAAACTGGAAAAGAATGACAAAAACCTTTGATTTATTAACCAATTCCTGCTTTC    4799
            ************************************************************

LA2093      AAATAATGAACTGGCCCCTAGAAATGGGTAAACAGTCTAAAACAGATTAGTAAACTTAAG    4838
E42         AAATAATGAACTGGCCCCTAGAAATGGGTAAACAGTCTAAAACAGATTAGTAAACTTAAG    4854
HEINZ       AAATAATGAACTGGCCCCTAGAAATGGGTAAACAGTCTAAAACAGATTAGTAAACTTAAG    4859
            ************************************************************

LA2093      TGGCAGCCAAACATTCTCCATTTCAGATCGTCTCATTGGAACCAATAAGAGGTGCTGAGT    4898
E42         TGGCAGCCAAACATTCTCCATTTCAGATCGTCTCATTGGAACCAATAAGAGGTGCTGAGT    4914
HEINZ       TGGCAGCCAAACATTCTCCATTTCAGATCGTCTCATTGGAACCAATAAGAGGTGCTGAGT    4919
            ************************************************************

LA2093      ATATTTCTGCTATGCATTATTGCCTGAGAAATTCTAGTGACTAAGGTGAGATAACATCAT    4958
E42         ATATTTCTGCTATGCATTATTGCCTGAGAAATTCTAGTGACTAAGGTGAGATAACATCAT    4974
HEINZ       ATATTTCTGCTATGCATTATTGCCTGAGAAATTCTAGTGACTAAGGTGAGATAACATCAT    4979
            ************************************************************

LA2093      TCTGTGTCCTCCAGAAATTGATCAATACTGGAAGAAATCGAAAGAACAATATGGGCAAGC    5018
E42         TCTGTGTCCTCCAGAAATTGATCAATACTGGAAGAAATCGAAAGAACAATATGGGCAAGC    5034
HEINZ       TCTGTGTCCTCCAGAAATTGATCAATACTGGAAGAAATCGAAAGAACAATATGGGCAAGC    5039
            ************************************************************

LA2093      CTCAATGTGCTTATAATTTATAAAGGACCTTTTTTCGCCAATTCAGGGAGTGAAAAGTTG    5078
E42         CTCAATGTGCTTATAATTTATAAAGGACCTTTTTTCGCCAATTCAGGGAGTGAAAAGTTG    5094
HEINZ       CTCAATGTGCTTATAATTTATAAAGGACCTTTTTTCGCCAATTCAGGGAGTGAAAAGTTG    5099
            ************************************************************

LA2093      CTATTAGCCAAAAGGACCAGATTGTCTACACCATGATAAATTGACAGCATA---------    5129
E42         CTATTAGCCAAAAGGACCAGATTGTCTACACCATGATAAATTGACAGCATAAGCCTTGCT    5154
HEINZ       CTATTAGCCAAAAGGACCAGATTGTCTACACCATGATAAATTGACAGCATAAGCCTTGCT    5159
            ***************************************************
```

```
LA2093       -------------------AGCCTTGTTGACTTAAGAGAAGACACATAGGTAGATTCAGA    5170
E42          TTTGATAACCAAAAGCATAAGCCTTGTTGACTTAAGAGAAGACACATAGGTAGATTCAGA    5214
HEINZ        TTTGATAACCAAAAGCATAAGCCTTGTTGACTTAAGAGAAGACACATAGGTAGATTCAGA    5219
                                *****************************************

LA2093       GACCGTCTCCAAAGGCTCTTACTTATTTCTAAATTTTGGAGGAGATACATTTGATGCTGA    5230
E42          GACCGTCTCCAAAGGCTCTTACTTATTTCTAAATTTTGGAGGAGATACATTTGATGCTGA    5274
HEINZ        GACCGTCTCCAAAGGCTCTTACTTATTTCTAAATTTTGGAGGAGATACATTTGATGCTGA    5279
             ************************************************************

LA2093       TGCTTAGACCTCCATATCTTTGCTTGCACGTAGAGGGATTGAGTGTTGGTGCAAGGAATA    5290
E42          TGCTTAGACCTCCATATCTTTGCTTGCACGTAGAGGGATTGAGTGTTGGTGCAAGGAATA    5334
HEINZ        TGCTTAGACCTCCATATCTTTGCTTGCACGTAGAGGGATTGAGTGTTGGTGCAAGGAATA    5339
             ************************************************************

LA2093       GAATCAAACAATTATTATCTATAAGAAAATCAACTACAGATAACTTGGTGGAGATTTAAT    5350
E42          GAATCAAACAATTATTATCTATAAGAAAATCAACTACAGATAACTTGGTGGAGATTTAAT    5394
HEINZ        GAATCAAACAATTATTATCTATAAGAAAATCAACTACAGATAACTTGGTGGAGATTTAAT    5399
             ************************************************************

LA2093       GGCTAAGTGCAGGGGTGGATGTAGGCTTTATGATATGTGTTCGACTGAACCAGTAACTTT    5410
E42          GGCTAAGTGCAGGGGTGGATGTAGGCTTTATGATATGTGTTCGACTGAACCAGTAACTTT    5454
HEINZ        GGCTAAGTGCAGGGGTGGATGTAGGCTTTATGATATGTGTTCGACTGAACCAGTAACTTT    5459
             ************************************************************

LA2093       GGTGTAATCACAATCTATGTTCCTAGAAATTCAGTAAATACGTACAAAAACTAAATTTAG    5470
E42          GGTGTAATCACTATCTATGTTCCTAGAAATTCAGTAAATACGTACAAGAACTAAATTTAG    5514
HEINZ        GGTGTAATCACTATCTATGTTCCTAGAAATTCAGTAAATACGTACAAGAACTAAATTTAG    5519
             *********** *********************************** ************

LA2093       AACTACTTAGAACTTGCCTTCGTTGTCCTACAATCCAGTGTCCATAAAATTCAAATCTTG    5530
E42          AACTACTTAGAACTTGCCTTCGTTGTCCTACAATCCAGTGTCCATAAAATTCAAATCTTG    5574
HEINZ        AACTACTTAGAACTTGCCTTCGTTGTCCTACAATCCAGTGTCCATAAAATTCAAATCTTG    5579
             ************************************************************

LA2093       GATTCTCCTCTAGCTAAGAGGTGTGGTTAAAGCCACTAAATATGACTGGAGAAAGAAAGG    5590
E42          GATTCTCCTCTAGCTAAGAGGTGTGGTTAAAGCCACTAAATATGACTGGAGAAAGAAAGG    5634
HEINZ        GATTCTCCTCTAGCTAAGAGGTGTGGTTAAAGCCACTAAATATGACTGGAGAAAGAAAGG    5639
             ************************************************************

LA2093       CATATGTAGCACGCCAAACACTGAACTATTTCAAATTTATTTAACTACTTCTACAGACAA    5650
E42          CATATGTAGCACGCCAAACACTGAACTATTTCAAATTTATTTAACTACTTCTACAGACAA    5694
HEINZ        CATATGTAGCACGCCAAACACTGAACTATTTCAAATTTATTTAACTACTTCTACAGACAA    5699
             ************************************************************

LA2093       AGGTTTCAGATCCTACTGAAGTCATTCCAAAAGAACACCTCCAAGGCTAGAAAAACTTAG    5710
E42          AGGTTTCAGATCCTACTGAAGTCATTCCAAAAGAACACCTCCAAGGCTAGAAAAACTTAG    5754
HEINZ        AGGTTTCAGATCCTACTGAAGTCATTCCAAAAGAACACCTCCAAGGCTAGAAAAACTTAG    5759
             ************************************************************

LA2093       TTAACTGCTTCCACCACACACTATTAAATCTCAAGTCATTGTAAGATACTTCCGCATGAA    5770
E42          TTAACTGCTTCCACCACACACTATTAAATCTCAAGTCATTGTAAGATACTTCCGCATGAA    5814
HEINZ        TTAACTGCTTCCACCACACACTATTAAATCTCAAGTCATTGTAAGATACTTCCGCATGAA    5819
             ************************************************************

LA2093       ATAAAACTGTTTAATCGCTCATTTCCTAGCTCTAAACCTATTAAGAGGTAAAAGCTTATC    5830
E42          ATAAAACTGTTTAATCGCTCATTTCCTAGCTCTAAACCTATTAAGAGGTAAAAGCTTATC    5874
HEINZ        ATAAAACTGTTTAATCGCTCATTTCCTAGCTCTAAACCTATTAAGAGGTAAAAGCTTATC    5879
             ************************************************************

LA2093       CTTTCTCCAAAACTTCTGAAAACAGGTAATCTAAATTAAAATGGGGAAACGTATTGTACA    5890
E42          CTTTCTCCAAAACTTCTGAAAACAGGTAATCTAAATTAAAATGGGGAAACGTATTGTACA    5934
HEINZ        CTTTCTCCAAAACTTCTGAAAACAGGTAATCTAAATTAAAATGGGGAAACGTATTGTACA    5939
             ************************************************************

LA2093       CGTGCAGGACGCACAAACTGATATAGTTACTGTCTTTATGCAAATGGAAATTCATAAGCA    5950
E42          CGTGCAGGACACACAAACTGATATAGTTACTGTCTTTTTGCAAATGGAAATTCATAAGCA    5994
HEINZ        CGTGCAGGACACACAAACTGATATAGTTACTGTCTTTTTGCAAATGGAAATTCATAAGCA    5999
             ********** ************************** **********************

LA2093       TGAATGACACTGTTTGCAAAAGTGCTGAAAGAAGGAAATCAATCCCCTTGAAATCACACT    6010
```

```
E42             TGAATGACACTGTTTGCAAAAGTGCTGAAAGAAGGAAATCAATCCCCTTGAAATCACACT     6054
HEINZ           TGAATGACACTGTTTGCAAAAGTGCTGAAAGAAGGAAATCAATCCCCTTGAAATCACACT     6059
                ************************************************************

LA2093          GTCACATAATTTTAGGATAAGCTTAAAATGGCGGAGCAGCTAAACTTAATGACGTAAAGC     6070
E42             GTCACATAATTTTAGGATAAGCTTAAAATGGCGGAGCAGCTAAACTTAATGACGTAAAGC     6114
HEINZ           GTCACATAATTTTAGGATAAGCTTAAAATGGCGGAGCAGCTAAACTTAATGACGTAAAAC     6119
                ********************************************************** *

LA2093          ACCTCTTGAGAATGATTATTGCACTTCCATATGAAAAAATGCACTAGTGCTCTCAGCAAA     6130
E42             ACCTCTTGAGAATGATTATTGCACTTCCATATGAAAAAATGCACTAGTGCTCTCAGCAAA     6174
HEINZ           ACCTCTTGAGAATGATTATTGCACTTCCATATGAAAAAATGCACTAGTGCTCTCAGCAAA     6179
                ************************************************************

LA2093          CAAAAACACTAGGTAATTTCTTCCCATATGCCTAGCTTAGTTGACAAAGTTACCTAGTTC     6190
E42             CAAAAACACTAGGTAATTTCTTCCCATATGCCTAGCTTAGTTGACAAAGTTACCTAGTTC     6234
HEINZ           CAAAAACACTAGGTAATTTCTTCCCATATGCCTAGCTTAGTTGACAAAGTTACCTAGTTC     6239
                ************************************************************

LA2093          CTATTGCTGGTGGGAGGTGTTAGCTATCTCGTGAAAACACGAAAGCTGG-CTAGAACACC     6249
E42             CTATTGCTGGTGGGAGGTGTTAGCTATCTCGTGAAAACACGAAAGCTGG-CTAGAACACC     6293
HEINZ           CTATTGCTGGTGGGAGGTGTTAGCTATCTCGTGAAAACACGAAAGCTGGTCTAGAACACC     6299
                ************************************************* **********

LA2093          ACAGCTATTAAAAAAAGGTTATTTTGCAGCTACTAGTCTACAACAATCAAAGATCAAATC     6309
E42             ACAGCTATTAAAAAAAGGTTATTTTGCAGCTACTAGTCTACAACAATCAAAGATCAAATC     6353
HEINZ           ACAGCTATTAAAAAAAGGTTATTTTGCAGCTACTAGTCTACAACAATCAAAGATCAAATC     6359
                ************************************************************

LA2093          TTTACTTTGCAGCTCCATGACTACAACAATCAAAGATCAAAACTTTATCCATTATTATCA     6369
E42             TTTACTTTGCAGCTCCATGACTACAACAATCAAAGATCAAAACTTTATCCATTATTATCA     6413
HEINZ           TTTACTTTGCAGCTCCATGACTACAACAATCAAAGATCAAAACTTTATCCATTATTATCA     6419
                ************************************************************

LA2093          CATATCTAAATCAAACAACAATTACATAACCTAAACTCTACGATATTGCCAACAAACTAA     6429
E42             CATATCTAAATCAAACAACAATTACATAACCTAAACTCTACGATATTGCCAACAAACTAA     6473
HEINZ           CATATCTAAATCAAACAACAATTACATAACCTAAACTCTACGATATTGCCAACAAACTAA     6479
                ************************************************************

LA2093          GCAAAAAAAAAAAACTCAATTTCATTGTGTTGTGCAACAACAAGAAAAGGCAAGAAAACT     6489
E42             GCAAAAA-AAAAAACTCAATTTCATTGTGTTGTGCAACAACAAGAAAAGGCAAGAAAACT     6532
HEINZ           GCAAAAA--AAAAACTCAATTTCATTGTGTTGTGCAACAACAAGAAAAGGCAAGAAAACT     6537
                *******  ***************************************************

LA2093          CACAGAATTCCACTTATTTTTCCATTTCTCAAAGCATATTGAGGTCGAATGGAAAGAGCT     6549
E42             CACAGAATTCCACTTATTTTTCCATTTCTCAAAGCATATTGAGGTCGAATGGAAAGAGCT     6592
HEINZ           CACAGAATTCCACTTATTTTTCCATTTCTCAAAGCATATTGAGGTCGAATGGAAAGAGCT     6597
                ************************************************************

LA2093          CAAATGGGTATTTTGTGATTGTGTAGAAGATACTATTCTTGTGGATTGAATAAGTGAATT     6609
E42             CAAATGGGTATTTTGTGATTGTGTAGAAGATACTATTCTTGTGGATTGAATAAGTGAATT     6652
HEINZ           CAAATGGGTATTTTGTGATTGTGTAGAAGATACTATTCTTGTGGATTGAATAAGTGAATT     6657
                ************************************************************

LA2093          CTTCAAGATCAAAACATTACGCCATTTGGGTATTACTTGCATTTTCTTAAAAATTTGAAA     6669
E42             CTTCAAGATCAAAACATTACGCCATTTGGGTATTACTTGCATTTTCTTAAAAATTTGAAA     6712
HEINZ           CTTCAAGATCAAAACATTACGCCATTTGGGTATTACTTGCATTTTCTTAAAAATTTGAAA     6717
                ************************************************************

LA2093          ACGACCCAAAAAGTGAAATACCAAAGATAAAAAACTGAACTTTTTTTTCCCCGATCTTTT     6729
E42             ACGACCCAAAAAGTGAAATACCAAAGATAAAAAACTGAACTTTTTTTTTCCCGATCTTTT     6772
HEINZ           ACGACCCAAAAAGTGAAATACCAAAGATAAAAAACTGAACTTTTTTTTTCCCGATCTTTT     6777
                ************************************************ ***********

LA2093          TGTACAAAGAATGAATCATCAATGGTG       6756
E42             TGTACAAAGAATGAATCATCAATGGTG       6799
HEINZ           TGTACAAAGAATGAATCATCAATGGTG       6804
                ***************************
```

Solyc11g008650

```
E42         TATTCGCAACGACGACCACCGGTGCCATTTTCCCTTTGGCAATTGAAGTAAACAGCAGCA    60
LA2093      TATTCGCAACGACGACCACCGGTGCCATTTTCCCTTTGGCAATTGAAGTAAACAGCAGCA    60
Heinz       TATTCGCAACGACGACCACCGGTGCCATTTTCCCTTTGGCAATTGAAGTAAACAGCAGCA    60
            ************************************************************

E42         ACAGGTAATCCAAGATTGTAGAGTTCAGCAAATTGTCTTGTATTGAAATTTTGACGCCAA    120
LA2093      ACAGGTAATCCAAGATTGTAGAGTTCAGCAAATTGTCTTGTATTGAAATTTTGACGCCAA    120
Heinz       ACAGGTAATCCAAGATTGTAGAGTTCAGCAAATTGCCTTGTATTGAAATTTTGACGCCAA    120
            *********************************** ************************

E42         TTTGGTGCATAAACAGTTTCTCTTCCCAATTGACGAAATAAAGAGAAAACAATTCTATGT    180
LA2093      TTTGGTGCATAAACAGTTTCTCTTCCCAATTGACGAAATAAAGAGAAAACAATTCTATGT    180
Heinz       TTTGGTGCATAAACAGTTTCTCTTCCCAATTGACGAAATAAAGAGAAAACAATTCTATGT    180
            ************************************************************

E42         ATTCCCATTGAAGGCCTTGGACTCTCATAACATATAACTTCAT      223
LA2093      ATTCCCATTGAAGGCCTTGGACTCTCATAACATATAACTTCAT      223
Heinz       ATTCCCTTTGAAGGCCTTGGACTCATAACATATAACTTC--AT      221
            ****** *****************  *  * ***       **
```

Solyc12g042560

```
LA2093      CACATTCTCAATCTGTTTAATCCTGCACCACTATTTCCAGTACCATAATCATCACCAGCA    60
E42         CACATTCTCAATCTGTTTAATCCTGCACCACTATTTCCAGTACCATAATCATCACCAGCA    60
HEINZ       CACATTCTCAATCTGTTTAATCCTGCACCACTATTTCCAGTACCATAATCATCACCAGCA    60
            ************************************************************

LA2093      CCACCTTGATACATTTTTCCAATGATTGGATTACACAACTTCTCTAACTCCTTCAATTTG    120
E42         CCACCTTGATACATTTTTCCAATGATTGGATTACACAACTTCTCTAACTCCTTCAATTTG    120
HEINZ       CCACCTTGATACATTTTTCCAATGATTGGATTACACAACTTCTCTAACTCCTTCAATTTG    120
            ************************************************************

LA2093      TTTTCAAATTCATCAACATCAGCCAATTGATTCCTATCCAACCATTCAATAGTTTCATCA    180
E42         TTTTCAAATTCATCAACTTCAGCCAATTGATTCCTATCCAACCATTCAATAGTTTCATCA    180
HEINZ       TTTTCAAATTCATCAACTTCAGCCAATTGATTCCTATCCAACCATTCAATAGTTTCATCA    180
            ***************** ******************************************

LA2093      ACAGCTTTCTCAATCTTCTGCTTCTCTGAAGAATCCAACTTCCCTGATATCTTTTCATCC    240
E42         ACAGCTTTCTCAATCTTCTGCTTCTCTGAAGAATCCAACTTCCCTGATATCTTTTCATCC    240
HEINZ       ACAGCTTTCTCAATCTTCTGCTTCTCTGAAGAATCCAACTTCCCTGATATCTTTTCATCC    240
            ************************************************************

LA2093      CTCACAGTATTCCTCATGTTATACGTGTAGTTCTCTAACGCGTTCTTCGCCTCCACTTTC    300
E42         CTCACAGTATTCCTCATGTTATACGTGTAGTTCTCTAACGCGTTCTTCGCCTCCACTTTC    300
HEINZ       CTCACAGTATTCCTCATGTTATACGTGTAGTTCTCTAACGCGTTCTTCGCCTCCACTTTC    300
            ************************************************************

LA2093      TTCTTCATTGCCTCATCCTCACACTTGTACCTCTCAGCTTTTTCAACCATTCTCTCAATC    360
E42         TTCTTCATTGCCTCATCCTCACACTTGTACCTCTCAGCTTTTTCAACCATTCTCTCAATC    360
HEINZ       TTCTTCATTGCCTCATCCTCACACTTGTACCTCTCAGCTTTTTCAACCATTCTCTCAATC    360
            ************************************************************

LA2093      TCTTCTTTACTTAGCCTCCCTTTGTCATTTGTAATAGTAATCTTGTTTTATCCAAACCAT    420
E42         TCTTCTTTACTTAGCCTCCCTTTGTCATTTGTAATAGTAATCTTGTTTTATCCAAACCAT    420
HEINZ       TCTTCTTTACTTAGCCTCCCTTTGTCATTTGTAATAGTAATCTTGTTTTATCCAAACCAT    420
            ************************************************************

LA2093      AAGCAATTGCAGCTGCAGTAGGTTCATTAATAATCCTCATTACATTCAACCCTGCAATAG    480
E42         AAGCAATTGCAGCTGCAGTAGGTTCATTAATAATCCTCATTACATTCAACCCTGCAATAG    480
HEINZ       AAGCAATTGCAGCTGCAGTAGGTTCATTAATAATCCTCATTACATTCAACCCTGCAATAG    480
            ************************************************************

LA2093      CACCAGCATCTTTAGTAGCTTGTCTTTGAGAATCATTAAAGTAAGCTGAACAGTAACCAC    540
E42         CACCAGCATCTTTAGTAGCTTGTCTTTGAGAATCATTAAAGTAAGCTGAACAGTAACCAC    540
HEINZ       CACCAGCATCTTTAGTAGCTTGTCTTTGAGAATCATTAAAGTAAGCTGAACAGTAACCAC    540
            ************************************************************

LA2093      AGCATTATCAACATTTTGTCCCAAAAAAGCCTCAGAAATTTCCTTCATTTTAATCAAGAC    600
E42         AGCATTATCAACATTTTGTCCCAAAAAAGCCTCAGAAATTTCCTTCATTTTAATCAAGAC    600
HEINZ       AGCATTATCAACATTTTGTCCCAAAAAAGCCTCAGAAATTTCCTTCATTTTAATCAAGAC    600
            ************************************************************

LA2093      CATTGAAGAAATCTCCTCTTCGTGCAAACTGTTTCTCCTCATTCTTGAAATTAAACAACA    660
E42         CATTGAAGAAATCTCCTCTTCGTGCAAACTGTTTCTCCTCATTCTTGAAATTAAACAACA    660
HEINZ       CATTGAAGAAATCTCCTCTTCGTGCAAACTGTTTCTCCTCATTCTTGAAATTAAACAACA    660
            ************************************************************

LA2093      ATCATAGGCTTGTCACCAGGTCCGGGAACAACCTTAAATGGCCAATGTTTCATATCAGAC    720
E42         ATCATAGGCTTGTCACCAGGTCCGGGAACAACCTTAAATGGCCAATGTTTCATATCAGAC    720
HEINZ       ATCATAGGCTTGTCACCAGGTCCGGGAACAACCTTAAATGGCCAATGTTTCATATCAGAC    720
            ************************************************************

LA2093      TGTACAGTTTGATCAGAGAAACGTCGAACCGATAAGACGTTTAGCATCAAAGACAGTGTT    780
E42         TGTACAGTTTGATCAGAGAAACGTCGAACCGATAAGACGTTTAGCATCAAAGATAGTGTT    780
HEINZ       TGTACAGTTTGATCAGAGAAACGTCGAACCGATAAGACGTTTAGCATCAAAGACAGTGTT    780
            ***************************************************** ******

LA2093      TTGGGGATTCATAGCAACTTGATTCTTGGCTGCATCACCAATAAAGACTTTCAGTATCTG    840
E42         TTGGGGATTCATAGCAACTTGATTCTTGGCTGCATCACCAATAAAGACTTTCAGTATCTG    840
HEINZ       TTGGGGATTCATAGCAACTTGATTCTTGGCTGCATCACCAATAAAGACTTTCAGTATCTG    840
            ************************************************************
```

```
LA2093      TGAATGCTACATAAGATGGAGTTGTTCTGTTGCCTTGATCATTTGGAATGATTTCAAACA    900
E42         TGAATGCTACATAAGATGGAGTTGTTCTGTTGCCTTGATCATTTGGAATGATTTCAAACA    900
HEINZ       TGAATGCTACATAAGATGGAGTTGTTCTGTTGCCTTGATCATTTGGAATGATTTCAAACA    900
            ************************************************************

LA2093      CGATCGTTTTGCCATACACCAACACAACTATAAGTTGTACCAAGATCAATCCCAATTGCT    960
E42         CGATCGTTTTGCCATACACCAACACAACTATAAGTTGTACCAAGATCAATCCCAATTGCT    960
HEINZ       CGATCGTTTTGCCATACACCAACACAACTATAAGTTGTACCAAGATCAATCCCAATTGCT    960
            ************************************************************

LA2093      TTTCCTTCAGATTTAGCCAT    980
E42         TTTCCTTCAGATTTAGCCAT    980
HEINZ       TTTCCTTCAGATTTAGCCAT    980
            ********************
```

Solyc12g042830

```
HEINZ         CACAAAACTTCAATTTCCTTAACATCTTTCTTAGGTTTGTCACTTTTAGGAATAGTAATT    60
E42           CACATAACTTCAATTTCCTGAACATCTTTCTTAGGTTTGTCACTTTTAGGAATAGTAATT    60
LA2093        CACATAACTTCAATTTCCTTAACATCTTTCTTAGGTTTGTCACTTTTAGGAATAGTAATT    60
              **** ************** ****************************************

HEINZ         GTCAAAACACCATCTTTCATCTCAGCTTTAATTTCATCAACCTTAGCATCTTGAGGCAAC    120
E42           GTCAAAACACCATCTTTCATCTCAGCTTTAATTTCATCAACCTTAGCATCTTGAGGCAAC    120
LA2093        GTCAAAACACCATCTTTCATCTCAGCTTTAATTTCATCAACCTTAGCATCTTGAGGCAAC    120
              ************************************************************

HEINZ         ACAATGCTATTATTATAGTAACCATAACTTGTTGATGACCAAAACTCATCATCTGATCCT    180
E42           ACAATGCTATTATTATAGTAACCATAACTTGTTGATGACCAAAACTCATCATCTGATCCT    180
LA2093        ACAATGCTATTATTATAGTAACCATAACTTGTTGATGACCAAAACTCATCATCTGATCCT    180
              ************************************************************

HEINZ         TCTTCTTCCTTCTCTTGTTTGTGTTCACCTTTTATTGTCAAAATTCCATCCTCAACCAT     239
E42           TCTTCTTCCTTCTCTTGTTTGTGTTCACCTTTTATTGTCAAAATTCCATCCTCAACCAT     239
LA2093        TCTTCTTCCTTCTCTTGTTTGTGTTCACCTTTTATTGTCAAAATTCCATCCTCAACCAT     239
              ***********************************************************
```

Solyc12g043120

```
HEINZ       TCTAGCTACAAGGAGCCAAACACCAGCTCAACAAATCATTTAGTGACCAACAGAAAATGT     60
E42         TCTAGCTACAAGGAGCCAAACACCAGCTCAACAAATCATTTAGTGACCAACAGAAAATGT     60
LA2093      TCTAGCTACAAGGAGCCAAACACCAGCTCAACAAATCATTTAGTGACCAACAGAAAATGT     60
            ************************************************************

HEINZ       CAGAGAACGGCAGCAGTTGTACATAACATCAAAACCCTCTGAAAAAACAGGTTTTTTGAG     120
E42         CAGAGAACGGCAGCAGTGTTACTTAACATCAAAACCCTCTGAGAAAACAGGTTTTTTGAG     120
LA2093      CAGAGAAC---AGCAGTGTTACTTAACATCAAAACCCTCTGAGAAAACAGGTTTTTTGAG     117
            ********   ******  *** ******************* *****************

HEINZ       AGTCGTTATCAAATAATATAACTTGACAAGACATCCGTATGGCCGAAATAGATCACAACA     180
E42         AGTCGTTATCAAATAATATAACTTGACAAGACATCTGTGTGGTCGAAATAGATCACAACA     180
LA2093      AGTCGTTATCAAATAATATAACTTGACAAGACATCTGTGTGGTCGAAATAGATCACAACA     177
            *********************************** ** *** *****************

HEINZ       ATTTAATGACAAAATCTTTCGACTGCTACTATAGGTCTGATCTTTTAAAATGTATAAAGG     240
E42         ATTTAATGACAAAATCTTTCGACTGCTACTATAGGTCTGATCTTTTAAAATGTATAAG-G     239
LA2093      ATTTAATGACAAAATCTTTCGACTGCTACTATAGGTCTGATCTTTTAAAATGTATAAG-G     236
            *********************************************************  *

HEINZ       GTGAAACGCAAAACGTAATACAAACCCAACAAAGTCTCTCTCTTCCTTCCAAATCTTCGA     300
E42         GTGAAACGCAAAAAGTAATACAAACCCAACAAAGTCTCTCTCTTCCTTCCAAATCTTTGA     299
LA2093      GTGAAACGCAAAAAGTAATACAAACCCAACAAAGTCTCTCTCTTCCTTCCAAATCTTTGA     296
            ************* ******************************************* **

HEINZ       GCATCAAACCATCAACTTAATTCATGATGCACTTGGCACA---TCAGATTTGTCCGTCTC     357
E42         GCATCAAACCATCAACTTAATTCATGATGCACTTGGCACAGTCTCAGATTTGTCTGTCTC     359
LA2093      GCATCAAACCATCAACTTAATTCATGATGCACTTGGCACAGTCTCAGATTTGTCTGTCTC     356
            ****************************************   *********** *****

HEINZ       CATTGGCTCGCCAGCAGGTGGTGCGCCACTGTCAGCACTATCACCCTCAGTAGCATTAGG     417
E42         CATTGGCTCGCCAGCAGGTGGTGCGCCACTGTCAGCACTATCACCCTCAGTAGCATTAGG     419
LA2093      CATTGGCTCGCCAGCAGGTGGTGCGCCACTGTCAGCACTATCACCCTCAGTAGCATTAGG     416
            ************************************************************

HEINZ       ACTCGCTGCACCCTGAGGTTGTTGCTCACCTCCTTGAGAAGATTGAGGTGACGGTGTTTC     477
E42         ACTCACTGCACCCTGAGGTTGTTGCTCACCTCCTTGAGAAGATTGAGGTGACGGTGTTTC     479
LA2093      ACTCACTGCACCCTGAGGTTGTTGCTCACCTCCTTGAGAAGATTGAGGTGACGGTGTTTC     476
            **** *******************************************************

HEINZ       AGGAGTTGCTGGCTTAGCTGGCTTAGGCTTTGTCATTATAGGCCTACAGACCCTGTAGAA     537
E42         AGGAGTTGCTGGCTTAGCTGGCTTAGGCTTTGTCATTATAGGCCTACAGACCCTGTAGAA     539
LA2093      AGGAGTTGCTGGCTTAGCTGGCTTAGGCTTTGTCATTATAGGCCTACAGACCCTGTAGAA     536
            ************************************************************

HEINZ       ACAAATAGTTAAAAAGTGGGTTAAGCAGACAGAAAAATATAGGGAAGGAAGAAATAAACA     597
E42         ACAAATAGTTAAAAAGTGGGCTAAGCAGACAGAAAAATATAGGGAAGGAAGAAATAAACA     599
LA2093      ACAAATAGTTAAAAAGTGGGCTAAGCAGACAGAAATATAGG--GAAGGAAGAAATAAACA     594
            ******************** ************** ***    *****************

HEINZ       AAGAA-ATGCTCTTATGTAGAAGGACAGCAACTAGCAAAGCAAGAAATGATATTGACAGA     656
E42         AAGAA-ATGCTCTTATGTAGAAGGACAGCAACTAGCAAAGCAAGAAATGATATCGACAGA     658
LA2093      AAGAAATGCTCTTTATGTAGAAGGACAGCAACTAGCAAAGCAAGAAATGATATCGACAGA     654
            *****       ***************************************** ******

HEINZ       CTTTCCAATAAAATTAGCCCAAATAATGCGATCTACAAGTTATGGCAAAATCCGACCAGA     716
E42         CTGTCAGATAAAATTAGCCCAAATAATGTGATCTACAAGTTATGGCAAAATCCGACCGGA     718
LA2093      CTGTCAGATAAAATTAGCCCAAATAATGTGATCTACAAGTTATGGCAAAATCCGACCGGA     714
            ** **  ********************* **************************** **

HEINZ       GGCAATAACAATAGCAACTATTCATTTGTCCAAACCTTGGTGTACCAAGATACCCAATAC     776
E42         GGCAATAACAATAACAACTTCTCATTTGTCCAAACCTTGTTGTACCAAGATACCCGATAC     778
LA2093      GGCAATAACAATAACAACTTCTCATTTGTCCAAACCTTGGTGTACCAAGATACCCGATAC     774
            ************* *****  ****************** *************** ****

HEINZ       CTTTGCTGGTGGAAGGTAGCAGGTACACTGTGGAAATACCACCTTCATCAAGAAAAAGAA     836
E42         CTTTGCTGGTGGAAGGTAGCAGGTACACTGTGGAAACACCACCTTCATCAAGAAAAAGAA     838
LA2093      CTTTGCTGGTGGAAGGTAGCAGGTACACTGTGGAAACACCACCTTCATCAAGAAAAAGAA     834
```

```
                ************************************ ***********************

HEINZ           AAGGAAACTCAATGAACAACAGACCTATCAAGTGCTTCTGCTTTCTTTCGAACATCAGCT     896
E42             AAGGAAACTCAATGAACAACAGACCTATCAAGTGCTTCTGCTTTCTTTCGAACATCAGCT     898
LA2093          AAGGAAACTCAATGAACAACAGACCTATCAAGTGCTTCTGCTTTCTTTCGAACATCAGCT     894
                ************************************************************

HEINZ           GACAAAAGAACAGGGTTGGCATATTTTGGAAGAGCATCCTGCTGCTGTTTTTTCTCTCTA     956
E42             GACAAAAGAACAGGGTTGGCATATTTTGAAAGAGCATCCTGCTGCTGTTTTTTCTCTCTA     958
LA2093          GACAAAAGAACAGGGTTGGCATATTTTGGAAGAGCATCCTGCTGCTGTTTTTTCTCTCTA     954
                **************************** *******************************

HEINZ           TACCAAGCCTCAGCTTCGACACACTCATTCAAGACCTAGGAATTGCAAGAGAAACATAAG     1016
E42             TACCAGGCCTCAGCTTCGACACACTCATTCAAGACCTAGGAATTGCAAGAGAAACATAAG     1018
LA2093          TACCAGGCCTCAGCTTCGACACACTCATTCAAGACCTAGGAATTGCAAGAGAAACATAAG     1014
                ***** ******************************************************

HEINZ           CATCAAACCACATTCTCAGGAACAAGATAGGCGAGCATATGATGATGAAAAGGCAAATAG     1076
E42             CATCAAACCACATTCTCAGGAACAAGATAGGCGAGCATATGATGATGAAAAGGCAAATAG     1078
LA2093          CATCAAACCACATTCTCAGGAACAAGATAGGCGAGCATATGATGATGAAAAGGCAAATAG     1074
                ************************************************************

HEINZ           CAAGATCAAGCACACCCATGCATACCTTCTGCTTCTCAGCTAAATCAATGTGATCAAACT     1136
E42             TAAGATCAAGCACACCCATGCATACCTTCTGCTTCTCTGCTAAATCAATGTGATCAAACT     1138
LA2093          TAAGATCAAGCACACCCATGCATACCTTCTGCTTCTCTGCTAAATCAATGTGATCAAACT     1134
                 ************************************ **********************

HEINZ           TGGGATCACTTGACACTGCCGCCTCTCTGTAACTATTTATGCAATAAATAAATTGATCAA     1196
E42             TGGGATCACTTGACACTGCCGCCTCTCTGTAACTATTTATGCAATAAACAAATTGATCAA     1198
LA2093          TGGGATCACTTGACACTGCCGCCTCTCTGTAACTATTTATGCAATAAACAAATTGATCAA     1194
                ************************************************ ***********

HEINZ           TCACAGGACCCCTCTCCGTGTGCTCTTTGTAGCGTTGCTCAATCGGGTCACCTTGCTGCA     1256
E42             TCACAGGACCCCTCTCCGTGTGCTCTTTGTAGCGTTGCTCAATCGGGTCACCTTGCTGCA     1258
LA2093          TCACAGGACCCCTCTCCGTGTGCTCTTTGTAGCGTTGCTCAATCGGGTCACCTTGCTGCA     1254
                ************************************************************

HEINZ           GATCCACACTAAATGAGCTCTATGTATGCCAAAAAGACTAGCAAAAAAGGAAATGCTAGA     1316
E42             GATCCACACTAAATCAGCTCTATGTATGCCAAAAAGACTAGCAAAAAAGGAAATGCTAGA     1318
LA2093          GATCCACACTAAATCAGCTCTATGTATGCCAAAAAGACTAGCAAAAAAGGAAATGCTAGA     1314
                ************** *********************************************

HEINZ           AACACAGACCTTTTTAAGCTCCTCAAGCTTGGCGATGTAAACACCCTTAGTTTCATCCTC     1376
E42             AACACCGACCTTTTTAAGCTCCTCAAGCTTGGCGATGTAAACACCCTTAGTTTCATCCTC     1378
LA2093          AACACCGACCTTTTTAAGCTCCTCAAGCTTGGCGATGTAAACACCCTTAGTTTCATCCTC     1374
                ***** ******************************************************

HEINZ           TCCATCTTCATACAACCAATCTTCCACTTCTTGAAGTACAGCCATAAATTGTTCTCTTTC     1436
E42             TCCATCTTCATACAACCAATCTTCCACTTCTTGAAGTACAACCATAAATTGTTCTCTTTC     1438
LA2093          TCCATCTTCATACAACCAATCTTCCACTTCTTGAAGTACAACCATAAATTGTTCTCTTTC     1434
                **************************************** *******************

HEINZ           TGAATCAGTTACAAACTCTTGATATTTATCTGAAAGCTGGATATAATAAGAAGAAAGTTG     1496
E42             TGAATCAGTTACAAACTCTTGATATTTATCTGAAAGCTGGATATATAAAGAAGAAAGTTG     1498
LA2093          TGAATCAGTTACAAACTCTTGATATTTATCTGAAAGCTGGATATATAAAGAAGAAAGTTG     1494
                *********************************************  *************

HEINZ           TCAGTGACATAACAAATGTTTTCATACAACCACCATAAAAAGCCAACTGATACCTTATTC     1556
E42             TCAGTGACATAACAAATGTTTT-ATACAACCACCATAAAAAGCCAACTGATACCTTATTC     1557
LA2093          TCAGTGACATAACAAATGTTTT-ATACAACCACCATAAAAAGCCAACTGATACCTTATTC     1553
                ********************** *************************************

HEINZ           CTCATGTCATAAACATAGGACTCAACAGCATTCTTTTTGTCCTTTGTCTCTTCCATAACA     1616
E42             CTCATGTCGTAAACATAGGACTCAACAGCATTCTTTTTGTCCTTTGTCTCTTCCATAACA     1617
LA2093          CTCATGTCGTAAACATAGGACTCAACAGCATTCTTTTTGTCCTTTGTCTCTTCCATAACA     1613
                ******** ***************************************************

HEINZ           CGGTCCTGAAGAGCCATTTCAAATTCTTTCTCAACAGCCTTCTGAACATCAGCAGCTGCC     1676
E42             CGGTCCTGAAGAGCCATTTCAAATTCTTTCTCAACAGCCTTCTGAACATCAGCAGCTGCC     1677
LA2093          CGGTCCTGAAGAGCCATTTCAAATTCTTTCTCAACAGCCTTCTGAACATCAGCAGCTGCC     1673
                ************************************************************
```

```
HEINZ           ATTGCACCATAAACAATCTCAGTCACTGGTACCGATGTCTTCTTGACCCTCTTCTTAGGG    1736
E42             ATTGCACCATAGACAATCTCAGTCACTGGTACCGATGTCTTCTTGACCTTCTTCTTAGGG    1737
LA2093          ATTGCACCATAGACAATCTCAGTCACTGGTACCGATGTCTTCTTGACCTTCTTCTTAGGG    1733
                *********** ************************************ ***********

HEINZ           GCTTCGACCTGAAAAAAAATGAACACAATAAATGCGA-----------CATCTTTGAGCT    1785
E42             GCTTCGACCTGAAAGAAAATGAACACAATAAATGCGA-----------CATCTTTGAGCT    1786
LA2093          GCTTCGACCTGAAAGAAAATGAACACAATAAATGCTAAGCTTTACCGACATCTTTGAGCT    1793
                ************** ******************** *           ************

HEINZ           AAACACACTGAAACAGAAGAAGAATTCTAAATATAACCCA-ACATTAATTAAGCAAGAAA    1844
E42             AAACACACTGAAACAGAAGAAGAATTCTAACTATAACCCA-ACATTAATTAAGCAAGAAA    1845
LA2093          TAACACACTGAAACAGAAGAAGAATTCTAACTATCACCCCAACATTAATTAAGCAAGAAA    1853
                 ***************************** *** ****  *******************

HEINZ           AGCACTACACTAA------TGCCGAATTAAAATATTGGTGAATTCATGAGTTTCCTGCAT    1898
E42             AGCACTACACTAA------TGCCGAATTAAAAGATAGGTGAATTCATGAGTTCCCTGCAT    1899
LA2093          AGCACTACACTAATGCCTTTCAAGAATTAAAAGATAGGTGAATTCATGAGTTCCCTGCAT    1913
                *************      *   ********* ** **************** *******

HEINZ           TTCATGCATAAATCAAATCCTGACAATTTGAACAGATGCCATAGGTTGATCTTATAACAA    1958
E42             TTCATGCATAAATCAAATCCTGACAATTTGAACAGATGCCATAGGTTGATCTTATAACAA    1959
LA2093          TTCATGCATAAATCAAATCCTGACAATTTGAACAGATGCCATAGGTTGATCTTATAACAA    1973
                ************************************************************

HEINZ           TCAAGTATGATTGATAGAGAAACATAAACTAGCTCAAGCAGTAATTAAAAGACGTGAAAG    2018
E42             TCAAGTATGATTGATAGAGAAACATAAACTAGCTCAAGCAGTAATTAAAAGACGTGAAAG    2019
LA2093          TCAAGTATGATTGATAGAGAAACATAAACTAGCTCAAGCAGTAATTAAAAGACGTGAAAG    2033
                ************************************************************

HEINZ           CGAGATGTTACCTTAGCATCTGACTCCATCTGGACAGGTTCATCTCCAGATTCTGGAACA    2078
E42             CGAGATGTTACCTTAGCATCTGATTCCATCTGGACAGGTTCATCTCCAGATTCTGGAACA    2079
LA2093          CGAGATGTTACCTTAGCATCTGATTCCATCTGGACAGGTTCATCTCCAGATTCTGGAACA    2093
                *********************** ************************************

HEINZ           CCATTCTCAGCCCCTGAAGCAGCTGCAGTTCCTTTAGCATCTTCCATATTCACATCACTT    2138
E42             CCATTCTCAGCCCCTGAAGCAGCTGCAGTTCCTTTAGCATCTTCCATATTCACATCACTT    2139
LA2093          CCATTCTCAGCCCCTGAAGCAGCTGCAGTTCCTTTAGCATCTTCCATATTCACATCACTT    2153
                ************************************************************

HEINZ           TCCGATGTAGTTGAAGGAGCAGCATCAGCTGAAGCTTCATCTGTTTCCATTCTGGCAGGT    2198
E42             TCCGATGTAGTTGAAGGAGCAGCATCAGCTGAAGCTTCATCTGTTTCCATTCTGGCAGGT    2199
LA2093          TCCGATGTAGTTGAAGGAGCAGCATCAGCTGAAGCTTCATCTGTTTCCATTCTGGCAGGT    2213
                ************************************************************

HEINZ           CCCTTAGCTGTCTCTTTCACAACTGGGACATCTACCTCTTCTTCTTCTAAGAGCTATATT    2258
E42             CCCTTAGCTGTCTCTTTCACAACTGGGACATCTACCTCTTCTTCTTCTAAGAGCTATATT    2259
LA2093          CCCTTAGCTGTCTCTTTCACAACTGGGACATCTACCTCTTCTTCTTCTAAGAGCTATATT    2273
                ************************************************************

HEINZ           ACAGAAAAAGCCATAAGATTAGATGATATTCTTTCGATTA-GAAAAAAACAAGAACGAGA    2317
E42             ACAGAAAAAGCCATAAGATAAGATGATATTCTTTCGATTTAGAAAAAAACAAGAACGAGA    2319
LA2093          ACAGAAAAAGCCATAAGATAAGATGATATTCTTTCGATTTAGAAAAAAACAAGAACGAGA    2333
                ******************* *******************  *******************

HEINZ           AAGCCACAAATCTCTGGACTTACAGTTGCGGACTCAACCGAGACAATACCATGCAGGGTT    2377
E42             AAGCCACAAATCTCTGGACTTACAGTTGCGGACTCAACCGAGACAATACCATGCAGGGTT    2379
LA2093          AAGCCACAAATCTCTGGACTTACAGTTGCGGACTCAACCGAGACAATACCATGCAGGGTT    2393
                ************************************************************

HEINZ           AGGCGTACTTTAACTTTCAGTTTGGCCCTTTCACCCTTTGAAGATTGGAATGGTCCAATC    2437
E42             AGGCGTACTTTAACTTTCAGTTTGGCCCTTTCACCCTTTGAAGATTGAAATGGTCCAATC    2439
LA2093          AGGCGTACTTTAACTTTCAGTTTGGCCCTTTCACCCTTTGAAGATTGAAATGGTCCAATC    2453
                *********************************************** ************

HEINZ           TGTCAGAAAGACCAAACAAAATCCAAGTCAGCCAAGGACTCGCCTGAAACTAGAGGACCT    2497
E42             TGTCAAAAAGACCAAACAAAATCCAAGTCAGCCAAGGACTCGCCTGAAACTAGAGGA-CT    2498
LA2093          TGTCAAAAAGACCAAACAAAATCCAAGTCAGCCAAGGACTCGCCTGAAACTAGAGGA-CT    2512
                ***** *************************************************** **

HEINZ           ATTATCCTCTAATTTTTAGAAAAGAAACCATAGACACTAATTAATGATTGAAAATCTGAA    2557
E42             ATTATCCTCTAATTTTTAGAAAAGAAACCATAGACACTAATTAATGATTGAGAATCTGAA    2558
```

```
LA2093      ATTATCCTCTAATTTTTAGAAAAGAAACCATAGACACTAATTAATGATTGAGAATCTGAA    2572
            *************************************************** ********

HEINZ       ATACCGTGTAAGTACTGATCTTTGCTGACGCCTGCAGTTCACTGACATCAGCATACTGTA    2617
E42         ATACCGTGTAATTACTGATCTTTGCTGGCGCCTGCAGTTCACTGACATCAGCATACTGTA    2618
LA2093      ATACCGTGTAAGTACTGATCTTTGCTGGCGCCTGCAGTTCACTGACATCAGCATACTGTA    2632
            *********** ***************** ******************************

HEINZ       CATCTGTTGTAAATGTGCCAGATCTGTAGAATGTCAGAGCTTTCACACTGGGTATGGGAT    2677
E42         CATCTGTTGTAAATGTGCCAGATCTGTAGAATGTCAGAGCTTTCACACTGGGTATGGGAT    2678
LA2093      CATCTGTTGTAAATGTGCCAGATCTGTAGAATGTCAGAGCTTTCACACTGGGTATGGGAT    2692
            ************************************************************

HEINZ       TCCCTTTGGGGAAAACAATTGTGCTCTGATGATTCTCTCCATTTTGTGCATCAGGTGAAG    2737
E42         TCCCTTTGGGGAAAACAATTGTGCTCTGATGATTCTCTCCATTTTGTGCATCAGGTGACG    2738
LA2093      TCCCTTTGGGGAAAACAATTGTGCTCTGATGATTCTCTCCATTTTGTGCATCAGGTGACG    2752
            ********************************************************** *

HEINZ       GCCCCTTCCATGATAATGCAATTGAGAAAGGGAAACTCTCATTGACCTACAATCAGCCAA    2797
E42         GCCCCTTCCATGATAATGCAATTGAGAAAGGGAAACTCTCATTGACCTACAATCAGCCAA    2798
LA2093      GCCCCTTCCATGATAATGCAATTGAGAAAGGGAAACTCTCATTGACCTACAATCAGCCAA    2812
            ************************************************************

HEINZ       CTATAACTCTTAGTACTTCTTACACTCATAAATCACCATAATTTCTTTCTTCTGGGAACT    2857
E42         CTCTAACTCTTAGTACTTCTTACACTCATTAATCACCATAATTTCTTTCTGCTGGGAACT    2858
LA2093      CTCTAACTCTTAGTACTTCTTACACTCATTAATCACCATAATTTCTTTCTGCTGGGAACT    2872
            ** ************************** ******************** *********

HEINZ       TTGATGTGTAATTAGTAAATAATCTTTTATTAAAGGTTAATAGTATAGCATCTGCTTCAA    2917
E42         TTGATGTGTAATTAGTAAATAATCTTTTAGTTAAGGTTAATAGTATAGCATTTGCTTCAA    2918
LA2093      TTGATGTGTAATTAGTAAATAATCTTTTAGTTAAGGTTAATAGTATAGCATTTGCTTCAA    2932
            ***************************** * ******************* ********

HEINZ       TAAAGATCTTCGTAGTTCTATGATAAATTATCCATTTCCTTTATCCTTCAAGACGACAAT    2977
E42         CAAAGATCTTCGTAGTTCTATGATAAATTATCCATTTCCTTCATCCTTCAAGACGACAAT    2978
LA2093      CAAAGATCTTCGTAGTTCTATGATAAATTATCCATTTCCTTCATCCTTCAAGACGACAAT    2992
             **************************************** ******************

HEINZ       TTCTTCTATCTCCAATTTGTCATTCATATAACCATAACCGGTCATACTCAAACCACTAAA    3037
E42         TTCTTCTATCTCCAATTTGTCATTCATACAACCATAACGGGTCATACTCAAACCACTAAA    3038
LA2093      TTCTTCTATCTCCAATTTGTCATTCATACAACCATAACGGGTCATACTCAAACCACTAAA    3052
            **************************** ********* *********************

HEINZ       TGAAACAAAAGGAAAAATCAAAAGCATTTTCTTAATTTATCTTCACATATTAAATTAGTG    3097
E42         TGAAACAAAAGGAAAAATCGAAAGCATTTTCTTAATTTATCTTCACATATTAAATTAGTG    3098
LA2093      TGAAACAAAGGAAAAATCGAAAGCATTTTTCTTAATTTATCTTCACATATTAAATTAGTG    3112
            ********* * ****    **    **********************************

HEINZ       CCTACTTAAAAAATAAGAACTAAGGATTATCAACAACATTGCAACACCTAATGAAATTGA    3157
E42         CCTACTTAAATAATAAGAACTAAGGATTATC---AACATTGCAACACGTAATGAAATTGA    3155
LA2093      CCTACTTATATAATAAGAACTAAGGATTATC---AACATTGCAACACGTAATGAAATTGA    3169
            ******** * ********************   ************* ************

HEINZ       AATCCCTCAATCCAAGATAACTATACAATAATATATCAGTCAAGAGAATGCAAGGTTGAC    3217
E42         AATCCCTCAATCCAAGATAACTATACAACAATATATCAGTCAAGAGAATGCAAGGTTGAC    3215
LA2093      AATCCCTCAATCCAAGATAACTATACAACAATATATCAGTCAAGAGAATGCAAGGTTGAC    3229
            **************************** *******************************

HEINZ       AAACTGTTACCTTGAATTCTCGCACTTTAAAAGTAGGGCTGAGAATAGCGCATTGCAGTG    3277
E42         AAACTGTTACCTTGAATTCTCGCACTTTAAAAGTAGGGCTGAGAATAGCGCATTGCAGTG    3275
LA2093      AAACTGTTACCTTGAATTCTCGCACTTTAAAAGTAGGGCTGAGAATAGCGCATTGCAGTG    3289
            ************************************************************

HEINZ       CAGCACCTTTGGCCACACATTCACTAGCATTCATGGTGCGCCTTGGTTCCTTACCAAAGA    3337
E42         CAGCACCTTTGGCCACACATTCACTAGCATTCATGGTGCGCCTTGGTTCCTTACCGAAGA    3335
LA2093      CAGCACCTTTGGCCACACATTCACTAGCATTCATGGTGCGCCTTGGTTCCTTACCGAAGA    3349
            ******************************************************* ****

HEINZ       ACTCCGTCAAAATCCTCATAATTGCGGGCACACGAGAGCTTGACCCAACAACCTCAACTG    3397
E42         ACTCCGTCAAAATCCTCATAATTGCGGGTACACGAGAGCTTGACCCAACAACCTCAACTG    3395
LA2093      ACTCCGTCAAAATCCTCATAATTGCGGGTACACGAGAGCTTGACCCAACAACCTCAACTG    3409
            **************************** *******************************
```

```
HEINZ         CATGAATGTTCTCAGTAGTAAGCCCAGCTTCAGCAAGAGCTTTCTCCAGTGGTTTCTTCA     3457
E42           CATGAATGTTCTCAGTAGTAAGCCCAGCTTCAGAAAGAGCTTTCTCCAGTGGTTTCTTCA     3455
LA2093        CATGAATGTTCTCAGTAGTAAGCCCAGCTTCAGAAAGAGCTTTCTCCAGTGGTTTCTTCA     3469
              ********************************* **************************

HEINZ         CTCTCTCCAGTATAGGGATGCTAATTTGCTCAAACTCATCCCTCTTGATAAACCCTCTGA     3517
E42           CTCTCTCCAGTATAGGGATGCTAATTTGCTCAAACTCATCCCTCTTGATAAACCCTCTGA     3515
LA2093        CTCTCTCCAGTATAGGGATGCTAATTTGCTCAAACTCATCCCTCTTGATAAACCCTCTGA     3529
              ************************************************************

HEINZ         CATCCTTCTCATCCATTAAACACTCTATATTCAAAGGTGCCTCAGGGTTTGCACTAAGAA     3577
E42           CATCCTTCTCATCCATTAAACACTCTATATTCAAAGGTGCCTCAGGGTTTGCACTGAGAA     3575
LA2093        CATCCTTCTCATCCATTAAACACTCTATATTCAAAGGTGCCTCAGGGTTTGCACTGAGAA     3589
              ******************************************************* ****

HEINZ         CCTTTTTCAACTTTTCACAAGCAGCTCGAAGTCTAATGCATGCCTTAGCATTTTGAAGAA     3637
E42           CCTTTTTCAACTTTTCACAAGCAGCTCGAAGTCTAATGCATGCCTTAGCATTTTGGAGAA     3635
LA2093        CCTTTTTCAACTTTTCACAAGCAGCTCGAAGTCTAATGCATGCCTTAGCATTTTGGAGAA     3649
              ******************************************************* ****

HEINZ         CATCAATTTTATATTCCTCCTTGAACTTTGCAGCAAAATGCTGGAAAAGAGCCTCATCAA     3697
E42           CATCAATTTTGTATTCCTCCTTGAACTTTGCAGCAAAATGCTGAAAAAGAGCCTCATCAA     3695
LA2093        CATCAATTTTGTATTCCTCCTTGAACTTTGCAGCAAAATGCTGAAAAAGAGCCTCATCAA     3709
              ********** ******************************** ****************

HEINZ         AATCCCTCCCACCAAGATTTCTGTCAAATGAATGAGCCAATATCTTCAATTGGCCTTTCT     3757
E42           AATCCCTCCCACCAAGATTTCTGTCAAATGAATGAGCCAATATCTTCAATTGGCCTTTCT     3755
LA2093        AATCCCTCCCACCAAGATTTCTGTCAAATGAATGAGCCAATATCTTCAATTGGCCTTTCT     3769
              ************************************************************

HEINZ         TGAAGCCAGCAATACAAACTTGCAAGCTTGCGTGCCCAACATCCACAAAAGCAACATTCA     3817
E42           TGAAGCCAGCAATACAAACTTGCAAGCTTGCGTGCCCAACATCCACAAAAGCAACATTCA     3815
LA2093        TGAAGCCAGCAATACAAACTTGCAAGCTTGCGTGCCCAACATCCACAAAAGCAACATTCA     3829
              ************************************************************

HEINZ         ATTGGTCATTTTCAGGTAAATCCGTCTTGTAAATACCATACGCTAATGCAGTAGCTGTTG     3877
E42           ATTGGTCATTTTCAGGTAAATCCGTCTTGTAAATACCATACGCTAATGCAGTAGCTGTTG     3875
LA2093        ATTGGTCATTTTCAGGTAAATCCGTCTTGTAAATACCATACGCTAATGCAGTAGCTGTTG     3889
              ************************************************************

HEINZ         TCTCGTGAATTAGATGCAAAGGATGCAAGCCAGCAATGGTGGCTGCGTCCATTACAGCTC     3937
E42           TCTCGTGAATTAGATGCAAAGGATGCAAGCCAGCAATGGTGGCTGCGTCCATTACAGCTC     3935
LA2093        TCTCGTGAATTAGATGCAAAGGATGCAAGCCAGCAATGGTGGCTGCGTCCATTACAGCTC     3949
              ************************************************************

HEINZ         TCCTCTGAAGATCAGTGAAATAAACTGGGATTCCAATGCAACAATCAACTACTGCTGCAT     3997
E42           TCCTCTGAAGATCAGTGAAATAAGCTGGGATTCCAATGCAACAATCAACTACTGCTGCAT     3995
LA2093        TCCTCTGAAGATCAGTGAAATAAGCTGGGATTCCAATGCAACAATCAACTACTGCTGCAT     4009
              *********************** ************************************

HEINZ         TGAGATTCTTCTCTGCTATAGTCTTCAGATCCGAAAACACCATTCCAACAACCTGGGTAG     4057
E42           TGAGATTCTTCTCTGCTATAGTCTTCAGATCCGAAAACACCATTCCAACAACCTGGGTAG     4055
LA2093        TGAGATTCTTCTCTGCTATAGTCTTCAGATCCGAAAACACCATTCCAACAACCTGGGTAG     4069
              ************************************************************

HEINZ         GGGTAAAAGTTCTCATTTCCCCCAAATAGCGTGCATGGATCAGGGGATATCCATCGGGCC     4117
E42           GGGTAAAAGTTCTCATTTCCCCCAAATAGCGTGCATGGATCAAGGGATATCCATCGGGCC     4115
LA2093        GGGTAAAAGTTCTCATTTCCCCCAAATAGCGTGCATGGATCAAGGGATATCCATCGGGCC     4129
              ****************************************** *****************

HEINZ         CTTCAGTTACTAAGAAGGGCAGTGCCTTAAGATCTCTTTGCAGCTCAGGATCTGAAAATT     4177
E42           CTTCAGTTACTAAGAAGGGCAGTGCCTTAAGATCTCTTTGCAGCTCAGGATCTGAAAATT     4175
LA2093        CTTCAGTTACTAAGAAGGGCAGTGCCTTAAGATCTCTTTGCAGCTCAGGATCTGAAAATT     4189
              ************************************************************

HEINZ         GTCGCCCTATTAGCCTCTTTATCTGTGAAATGGTGTTCTTTGGGTTCATCATGCTTGACG     4237
E42           GTCGCCCTATTAGCCTCTTTATCTGCGAAATGGTGTTCTTTGGGTTCATCATGCTTGACG     4235
LA2093        GTCGCCCTATTAGCCTCTTTATCTGCGAAATGGTGTTCTTTGGGTTCATCATGCTTGACG     4249
              ************************* **********************************

HEINZ         CTGCACCAGCAGTACCAAGAAATCGTTGCTTCTCTCCAAAGCAGACGATGGCTGGAGTTT     4297
```

```
E42        CTGCACCAGCAGTACCAAGAAATCGTTGCTTCTCTCCAAAGCAAACGATAGCTGGAGTTT   4295
LA2093     CTGCACCAGCAGTACCAAGAAATCGTTGCTTCTCTCCAAAGCAAACGATAGCTGGAGTTT   4309
           ******************************************* ***** **********

HEINZ      CCCTTTTGGATTCGTCATTAAGTACAACATCAATTCCTCGCTGCCTAGCAACCGCAACAA   4357
E42        CCCTTTTTGATTCGTCATTAAGTACAACATCAATTCCTCGCTGCCTAGCAACCGCAACAA   4355
LA2093     CCCTTTTTGATTCGTCATTAAGTACAACATCAATTCCTCGCTGCCTAGCAACCGCAACAA   4369
           ******* ****************************************************

HEINZ      CGCCACTTTCATTGCCGAAGTCAAAACCAACCACACTCATCCTTGCTGACTTCGGTCACT   4417
E42        CGCCACTTTCATTGCCGAAGTCAAAACCAACCACACTCATCCTTGCTGACTTCGGTCACT   4415
LA2093     CGCCACTTTCATTGCCGAAGTCAAAACCAACCACACTCATCCTTGCTGACTTCGGTCACT   4429
           ************************************************************

HEINZ      CAATATCCAACAGCACAAGCACGAAGCACGATAAAGTCCCTGGACAACATCAAAAGAAGT   4477
E42        CAATATCCAACAGCACAAG-------CACGATAAAGTCCCTGGACAACATCAAAAGAAGT   4468
LA2093     CAATATCCAACAGCACAAG-------CACGATAAAGTCCCTGGACAACATCAAAAGAAGT   4482
           *******************       **********************************

HEINZ      ATATAATCAACTAAACACCAGCATCCGATGTATCACATTAAGAAGCTACAACAACAACAA   4537
E42        ATATAATCAACTAAACACCAGCATCCGATGTATCACATTAAGAAGCTACAACAACAACAA   4528
LA2093     ATATAATCAACTAAACACCAGCATCCGATGTATCACATTAAGAAGCTACAACAACAACAA   4542
           ************************************************************

HEINZ      ACTTAGTGTAATCCTACAAGCAGGGTCTGAGAAGGTAAGTGTGTATGCAGACCATACCCC   4597
E42        ACCTAGTGTAATCCTACAAGCGGGGTCTGAGAAGGTAAGTGTGTATGCAGACCATACCCC   4588
LA2093     ACCTAGTGTAATCCTACAAGCGGGGTCTGAGAAGGTAAGTGTGTATGCAGACCATACCCC   4602
           ** ****************** **************************************

HEINZ      TGCCATGTGAAGGTAGAGAGGTTTTCCATGGACCCTCGGCTCAGGTATAAAATATATCAT   4657
E42        TGCCATGTGAAGGAAGAGAGGTTTTCCACGGACCCTCGGCTCAGGTATAAAATATCTCAT   4648
LA2093     TGCCATGTGAAGGAAGAGAGGTTTTCCACGGACCCTCGGCTCAGGTATAAAATATATCAT   4662
           ************* ************** ************************** ****

HEINZ      TAACAAAAGGAAATAACAAAGTGAAGAAGTCACGAATAAAATAGCGGAGAAAATAACCAT   4717
E42        TAACAAAAGGAAATAACAAAGTGAAGAAGTCACGAATAAAATAGCGGAGAAAATAACCAT   4708
LA2093     TAACAAAAGGAAATAACAAAGTGAAGAAGTCACGAATAAAATAGCGGAGAAAATAACCAT   4722
           ************************************************************

HEINZ      AGCAACAACAACAAAATAAAGATAACTGAAAGAAGGGAACATCAAGTAATAATGAAATCA   4777
E42        AGCAACAACAACAAAATAATGATAACTGAAAGAAGGGAACATCAAGTAATAATGAAATCA   4768
LA2093     AGCAACAACAACAAAATAATGATAACTGAAAGAAGGGAACATCAAGTAATAATGAAATCA   4782
           ******************* ****************************************

HEINZ      TTGACTAATAGAACCATCGACAAATAAGATAGTAGGATAGTAGGATAGTAATAATAACAG   4837
E42        TTGACGAATAGAACCATCGACAAATAAGATAGTAGGATA-------------GATAACAG   4815
LA2093     TTGACGAATAGAACCATCGACAAATAAGATAGTAGGAGA-------------GTTAACAG   4829
           ***** ******************************* *               ******

HEINZ      TACTGACAAGGGAGACTAGACAACGCTCGACTATGTACTAACCTTCTACCCTAATCCTGA   4897
E42        TACTGACAAGGAAGACTAGACAACGCTCGACTATGTACTAACCTTCTACCCTAATCCTGA   4875
LA2093     TACTGACAAGGAAGACTAGACAACGCTCGACTATGTACTAACCTTCTACCCTAATCCTGA   4889
           *********** ************************************************

HEINZ      TCCCATATGCCCCTTTCTAGGGAGCATGTCCTCGATAAGTTGAAGGTGCGTCATGGAAGA   4957
E42        TCCCATATGCCCCTTTCTAGGGAGCATGTCCTCGATAAGTTGCAGGTGCGTCATGGAAGA   4935
LA2093     TCCCATATGCCCCTTTCTAGGGAGCATGTCCTCGATAAGTTGCAGGTGCGTCATGGAAGA   4949
           ****************************************** *****************

HEINZ      AGCGATGGGTTTTAAATTGTAGCAACCAAGAGCGACTTCATAAAATGATTGATTTTTCAT   5017
E42        AGCGATGGGTTTTAAATTGTAGCAACCAAGAGCAACATCATAAAATGATTGATTTTTCAT   4995
LA2093     AGCGATGGGTTTTAAATTGTAGCAACCAAGAGCAACATCATAAAATGATTGATTTTTCAT   5009
           ********************************* ** ***********************

HEINZ      TTATAAGCTAAGAAAGAGGCTACCTCGTGGTGGGGCTACCCTATCCAGTAATGATGGATT   5077
E42        TTATAAGCTAACAAAGAGGCTCCCTCGTTGAGGGGCTAACCTATCCAGTACTGATGGATT   5055
LA2093     TTATAAGCTAACAAAGAGGCTCCCTCGTTGAGGGGCTAACCTATCCAGTACTGATGGATT   5069
           *********** ********* ****** * ******* *********** *********

HEINZ      CACAGAATAGAGCAAAGAAGTTCATTTTTCCATTTATGATTAGAGAGAGAAAACATAACC   5137
E42        CACAGAATAGAGCAAAGAAGTTCATTTTTCCATTTATGATTAGAGAGAGAAAACATAACC   5115
LA2093     CACAGAATAGAGCAAAGAAGTTCATTTTTCCATTTATGATTAGAGAGAGAAAACATAACC   5129
```

```
                ************************************************************

HEINZ           TGGCCTAACGTATTTAATGATCTCCAGACATGTCATTTAAAAAGTCAACAATCAAACCGG    5197
E42             TGGCCTAACATATTTAATAATCCCCAGACACGTCATTTAAAAAGTCAACACTCATACCGA    5175
LA2093          TGGCCTAACATATTTAATAATCCCCAGACACGTCATTTAAAAAGTCAACACTCATACCGA    5189
                ********* ******** *** ******* ******************* *** ****

HEINZ           CGTGGACTCCAAGTTCAAAGATGGTATCTTTTTTCTAATTTACACAATATCCAAAGCTCC    5257
E42             CGTGGACTCTAAGTTCAAAGATGGTATCTTTTTTCTAATTTACCCAATATCCAAAGCGCC    5235
LA2093          CGTGGACTCTAAGTTCAAAGATGGTATCTTTTTTCTAATTTACCCAATATCCAAAGCGCC    5249
                ********* ********************************* ************* **

HEINZ           AAGTATCCAAATTTTATCCAACTTCCCGCCAAGGAAACTTTCGTGCATGAATCTCATGCA    5317
E42             AAGTATCCAAATTTTATCCAACTTCCAGCCAAGGAAACTTTCATGCATGAATCTCATGCA    5295
LA2093          AAGTATCCAAATTTTATCCAACTTCCAGCCAAGGAAACTTTCATGCATGAATCTCATGCA    5309
                ************************** *************** *****************

HEINZ           CGTATCGATAAGACAGGAAAAGATAGTAATGCATCAACGAGAACTGAATCTTTTTTAATC    5377
E42             CGTATCGACAAGACAGGAAACAATTGTAATGCATCAACGAGAACTGAATCTTTTTTAATC    5355
LA2093          CGTATCGACAAGACAGGAAACAATTGTAATGCATCAACGAGAACTGAATCTTTTTTAATC    5369
                ******** ***********  ** ***********************************

HEINZ           TACTATCTCCCAATACACAAAATCGCCCAAAATTTACCCTATATCAATAAAACAGGGCTC    5437
E42             TACTATCTCCCAATACACAAAATCGCCCAAAATTCACCCTATATCAATAAAACAGGGCTC    5415
LA2093          TACTATCTCCCAATACACAAAATCGCCCAAAATTCACCCTATATCAATAAAACAGGGCTC    5429
                ********************************** *************************

HEINZ           TACATAGCCTCGTATAAACTAAACCAAACACATTTCCTTCCAATAATAAAAACTCAAACC    5497
E42             TACATTGCCTCGTATAAACTAAACCAAACACATTTCCTTCCAATAATAAAAACTCAAACC    5475
LA2093          TACATTGCCTCGTATAAACTAAACCAAACACATTTCCTTCCAATAATAAAAACTCAAACC    5489
                ***** ******************************************************

HEINZ           AAACACTGATCCAAAAAAGCCATTAGCTTGCTCTATTCTCCCACCCCCATCCCTCCCTAA    5557
E42             AAACACTGATCCAAAAAAGCCATTAGCTCGCTCTATTCTCCCACCCCCATCCCTCCCTAA    5535
LA2093          AAACACTGATCCAAAAAAGCCATTAGCTCGCTCTATTCTCCCACCCCCATCCCTCCCTAA    5549
                **************************** *******************************

HEINZ           GTGAATCTCTAATCTTAAGAATTGATCTGATTGAGGACAAACCAAAGACTTATCTACCTA    5617
E42             GTGAATCTCTAATCTTAAGAATTGATCTGATTGAGGACAAACCAAAGACTTATCTACCTA    5595
LA2093          GTGAATCTCTAATCTTAAGAATTGATCTGATTGAGGACAAACCAAAGACTTATCTACCTA    5609
                ************************************************************

HEINZ           GTAGATTACAACAGCACCCAATCTAGTCTACAGGATTTCCCATCCGCCCCAATTATCAAT    5677
E42             GTAGATTACAACAGCACCCAATCTAGTCTACAGGATTTCCCATCCGCCCCAATTATCAAT    5655
LA2093          GTAGATTACAACAGCACCCAATCTAGTCTACAGGATTTCCCATCCGCCCCAATTATCAAT    5669
                ************************************************************

HEINZ           CTAAAATCCACTCAGTTTCTTCATTTAATTATCATTTTTTCTCATTTGTCACATCAACCA    5737
E42             CTAAAATCCACTCAGTTTCTTCATTTAATTATCATTTTTTCTCATTTGTCACATCAACCA    5715
LA2093          CTAAAATCCACTCAGTTTCTTCATTTAATTATCATTTTTTCTCATTTGTCACATCAACCA    5729
                ************************************************************

HEINZ           ACAATGCAGGAACGATAATCTACTAATCATCAACCTATACAACAGCCTATATCAACAAAA    5797
E42             ACAATGCAGGAACGATAATCTACAAATCATCAACCTATACAACAGCCTATATCAACAAAA    5775
LA2093          ACAATGCAGGAACGATAATCTACAAATCATCAACCTATACAACAGCCTATATCAACAAAA    5789
                *********************** ************************************

HEINZ           CATACAACAGATTCAGTTTACACCTAACAAAACATGCAATTCACAAAGAGAACTCTAAAC    5857
E42             CATACAACAGATTCAGTTTACACCTAACAAAACATGCAATTCACAAAGAGAACTCTAAAC    5835
LA2093          CATACAACAGATTCAGTTTACACCTAACAAAACATGCAATTCACAAAGAGAACTCTAAAC    5849
                ************************************************************

HEINZ           ATCACCAAATCTAAATCAAATCATAGATCTACAAAAGAGTTTTGTGATTCAAACAATACC    5917
E42             ATCACCAAATCTAAATCAAATCATAGATCTACAAAAGAGTTTTGTGATTCAAACAATACC    5895
LA2093          ATCACCAAATCTAAATCAAATCATAGATCTACAAAAGAGTTTTGTGATTCAAACAATACC    5909
                ************************************************************

HEINZ           TTGAAGAGAAAGATATCTGTTCCACGGACTCCGGCTGTGTACGATTGATCTTTTTCCTGA    5977
E42             TTGAAGAGAAAGATATCTGTTCCACGGACTCCGGCTGTGTACGATTGATCTTTTTCCTGA    5955
LA2093          TTGAAGAGAAAGATATCTGTTCCACGGACTCCGGCTGTGTACGATTGATCTTTTTCCTGA    5969
                ************************************************************
```

```
HEINZ       AGGAACCAAACGGAGAATACTGATAAAACGGAAGGGTTTAAAGAAGAAGGGTTCACAGTT    6037
E42         AGGAACCAAACGGAGAATACTGATAAAACGGAAGGGTTTAAAGAAGAAGGGTTCACAGTT    6015
LA2093      AGGAACCAAACGGAGAATACTGATAAAACGGAAGGGTTTAAAGAAGAAGGGTTCACAGTT    6029
            ************************************************************

HEINZ       TTATACTTGAAAAAATAAAAAATGAGTC    6065
E42         TTATACTTGAAAAAATAAAAAATGAGTC    6043
LA2093      TTATACTTGAAAAAATAAAAAATGAGTC    6057
            ****************************
```
